# A modular synthesis of azetidines from reactive triplet imine intermediates using an intermolecular aza Paternò–Büchi reaction

**DOI:** 10.1038/s41929-025-01405-7

**Published:** 2025-09-05

**Authors:** Benedict A. Williams, Michael J. Tilby, Nicholas A. Parker, Mycah R. Uehling, J. Caleb Hethcox, Dipannita Kalyani, Michael C. Willis

**Affiliations:** 1 Department of Chemistry, University of Oxford, Oxford, UK; 2 Department of Chemistry, University of Manchester, Manchester, UK; 3 Discovery Chemistry, Merck & Co., Rahway, NJ USA; 4 Department of Process Research and Development, Merck & Co., Rahway, NJ USA

**Keywords:** Synthetic chemistry methodology, Photocatalysis

## Abstract

Azetidines are four-membered saturated N-heterocycles that are of interest in discovery chemistry. However, the implementation of these structures is limited by their synthetic intractability, resulting from their inherent ring strain. An approach that circumvents this is the intermolecular [2 + 2] photocycloaddition between imines and alkenes. However, this is unworkable with simple acyclic imines and non-activated alkenes, due to the inability to generate suitably reactive imine-derived triplet intermediates. Here we show that simple acyclic imines bearing N-sulfamoyl fluoride substituents generate reactive triplet imines that react with a broad range of alkenes to produce azetidine products in high yields. Mechanistic and computational studies confirm the key role of the sulfamoyl fluoride unit in dictating the [2 + 2] pathway. In addition, the sulfamoyl fluoride substituents offer a convenient reaction site for product functionalization or for traceless removal. The advent of synthetically useful imine-derived triplets should initiate further research and applications of these elusive reactive intermediates.

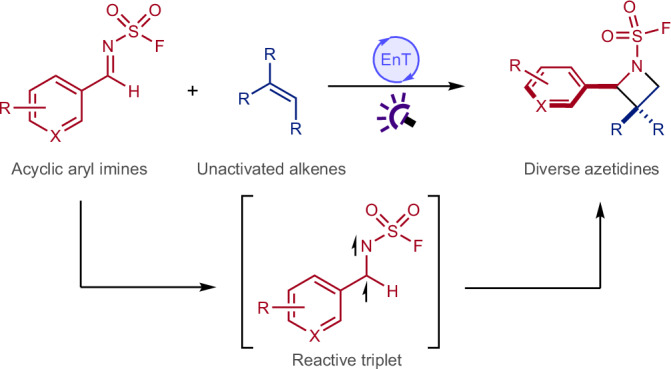

## Abstract (web summary)

Azetidines are four-membered saturated N-heterocycles that are of interest in drug discovery and medicinal chemistry. Here the authors report how sulfamoyl fluoride substituents tune the reactivity of acyclic imine-derived triplet intermediates for the synthesis of azetidines via a [2 + 2] photocycloaddition reaction with alkenes.

## Main

**Fig. 1:**
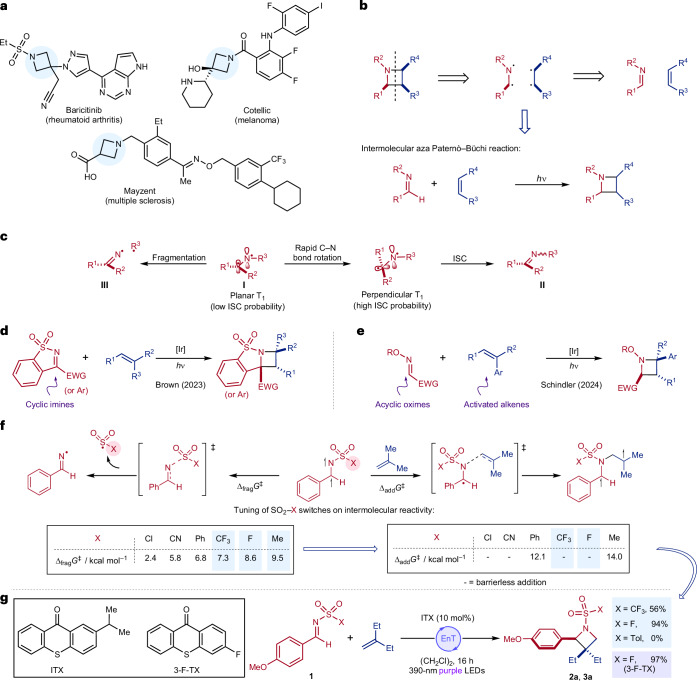
Background and an intermolecular aza Paternò–Büchi reaction using sulfamoyl fluoride imines. **a**, Examples of pharmaceutically relevant azetidines. **b**, Retrosynthetic analysis of azetidines and the aza Paternò–Büchi reaction. **c**, Established alternative modes of reactivity for imine derived triplet intermediates. **d**,**e**, Prior art for intermolecular aza Paternò–Büchi reactions using cyclic imines (**d**) and using excitable alkenes (**e**). **f**, Model system for study (DFT method: SMD(1,2-dichloroethane)-M06-2X-D3/def2-TZVP in kcal mol^−1^). **g**, Preliminary results for the development of an intermolecular aza Paternò–Büchi reaction. ISC, intersystem crossing.

Saturated N-heterocycles are ubiquitous in nature in the form of alkaloid natural products^1^ and are present in a multitude of biologically active molecules used in agrochemistry^2^ and medicinal chemistry. For example, >80% of all drugs approved by the US Food and Drug Administration (FDA) in the past 10 years incorporate at least one N-heterocycle^3^. Of these, the most common saturated examples are six-membered rings, reflecting the wide availability of piperidines and piperazines. By contrast, azetidines, the four-membered homologues of piperidines, occupy only a small fraction of chemical space, rank 26th on the list and account for only six approved drugs^3^ (see Fig. 1a for selected examples). As a result of their intrinsic small size and rigidity, azetidines present an atom-efficient three-dimensional scaffold with defined vectors to present pendant functionality^4^. Incorporating azetidines into bioactive molecules has been shown to impart myriad desirable properties, including improved stability to oxidative metabolism^5^, improved solubility^6^ and structural rigidity while simultaneously increasing the fraction of C*sp*^3^ centres^4^. Despite these attributes, azetidines remain underutilized scaffolds in drug design^7^, primarily due to the limitations of established synthetic methods^8^. For example, azetidines commonly used in drug design generally feature substituents predominantly at the 3-position, while other positions—and di-substituted variants—are rarely represented; this is due to the scarcity of convenient methods to access more complex substitution patterns. Recently, the application of modern synthetic methods to this task has resulted in improved syntheses, including processes based on strain release of aza-bicyclobutanes^9–12^, C–H amination^13^, photochemical cyclizations^14,15^ and hydrogen atom transfer catalysis^16^. Although these developments have partly alleviated this challenge, they still suffer from a principal limitation of earlier methods: the stringent requirement for complex, prefunctionalized substrates.

An appealing reaction to prepare azetidines would use electronically diverse feedstock olefins together with modular, readily synthesizable imines and directly couple them together via an intermolecular [2 + 2] cyclization; this general transformation, when achieved using light, is known as the aza Paternò–Büchi reaction^17^ (Fig. 1b). The parent transformation—the Paternò-Büchi reaction—refers to the [2 + 2] photocycloadditions of excited carbonyl compounds and alkenes and is a well-established transformation^18–21^. The aza variant represents a direct, atom-economical and modular synthesis of azetidines from two equally complex coupling partners. However, the aza Paternò–Büchi reaction remains underdeveloped. This is due to the stringent requirements placed on the imine reaction component^22,23^, which upon triplet sensitization must circumvent radiationless decay back to the ground-state (**I** → **II**; Fig. 1c), be resistant to photoreduction, and avoid fragmentation (**I** → **III**)^24–26^. The advancements in intermolecular aza Paternò–Büchi methodologies so far have relied on engineered substrates to prevent decay to the ground state, typically achieved by tethering the imine within a ring to prevent rotation around the carbon–nitrogen π-bond^27–31^ (Fig. 1d). An alternative strategy to skirt this challenge is to induce a formal aza Paternò–Büchi reaction by essentially reversing the reactivity and using activated alkenes that are excited in preference to the imine. A notable recent example of this strategy from the Schindler laboratory utilizes acyclic oximes as imine equivalents, combined with activated alkenes, enabling the matching of frontier molecular orbital energies between the two reaction components to achieve an efficient process^32^ (Fig. 1e). There are a small number of less general solutions, including matching substrates for exciplex formation with singlet excitation^33,34^, and the use of copper catalysis to activate bicyclic alkenes in combination with ultraviolet light^35^. Collectively, these strategies have enabled efficient reactions with broad scope; however, the need to engineer the imine structure, use imine equivalents or employ activated alkenes compromises structural diversity and thus undermines one of the major advantages of an idealized aza Paternò–Büchi reaction^17^.

As a simple and direct aza Paternò–Büchi reaction remains elusive, we sought to address this by designing acyclic imines that fulfil several key requirements. First, these components must have accessible excited states through photochemical or physical means. Second, the excited imines must be able to engage with the olefin component in a productive regioselective manner, favouring this pathway over the aforementioned deleterious alternatives. Finally, it is imperative that the modular, acyclic imines are readily synthesizable from commercial substrates in no more than one step. To achieve these specifications we targeted sulfonyl imines, primarily due to their known photophysics, and triplet excited states accessible through triplet energy transfer catalysis^36–38^. In selecting sulfonyl imines as our substrates of choice we were conscious of the rich chemistry that has been developed exploiting fragmentation of the derived triplets (that is, **I** → **III**; Fig. 1c), leading to many productive addition processes^24,26,37^. Nevertheless, we reasoned that the tunability of the sulfonyl substituent should enable effective partitioning between the competing pathways and establishment of a productive catalytic manifold. To implement this reaction design, we propose that controlling the electronic properties of the triplet imine can influence the fragmentation barrier; by minimizing this deleterious pathway, we aim to increase the likelihood of productive catalysis. A recent report has used sulfonyl imines in combination with excitable alkenes to achieve aza Paternò–Büchi products^39^.

Here, we show that acyclic sulfamoyl fluoride-substituted aryl imines deliver reactive triplet intermediates that engage as substrates in intermolecular aza Paternò–Büchi reactions using energy-transfer catalysis and visible light. A broad range of alkenes are used as reaction partners and provide substituted azetidines in high yields. We demonstrate the utility of this synthetic method by further manipulation of the N-sulfamoyl fluoride group present in the azetidine products, which can either be removed to provide the N–H heterocycles or be readily converted into a sulfamide unit.

## Results

### Computational and experimental reaction optimization

Using density functional theory (DFT)^40–43^, we assessed a series of imines substituted with a diverse range of –SO_2_–X functional groups and noted that the energy of the S–N σ* orbital of the triplet imine could be used to help qualitatively predict the degree of fragmentation (Supplementary Fig. 12), where an increase in the orbital energy correlated with a higher barrier to fragmentation (Fig. 1f). For the groups studied, the fragmentation energies of the various sulfonyl imines spanned a range of 2.4–9.5 kcal mol^−1^. For the desired bimolecular reaction to compete with the unimolecular fragmentation, the barrier to fragmentation must be appreciably high (**≳**4.0 kcal mol^−1^)—that is, greater than the diffusion limit—and therefore we selected the –SO_2_CF_3_-, –SO_2_Me- and –SO_2_F-substituted imines as promising candidates for further investigation. We next considered the corresponding activation energies for addition into isobutene. For several of the sulfonyl imines, we calculated barrierless additions, while the –SO_2_Me imine had a relatively large barrier of 14.0 kcal mol^−1^ and therefore would be unlikely to succeed in an intermolecular [2 + 2] addition. We therefore proceeded with the –SO_2_CF_3_- and –SO_2_F-substituted imines for experimental investigation.

Experimentally we designed our system around the metal-free photocatalyst 2-isopropyl thioxanthone (ITX) and the use of visible light to access the triplet via energy transfer catalysis^38,44^, coupled with 3-methylenepentane as a non-gaseous electron-neutral alkene, comparable to the gaseous isobutene used in our computational study. Consistent with the conclusions of our qualitative theoretical investigation both –SO_2_CF_3_- and –SO_2_F-substituted imines provided the corresponding azetidine products, in 56% and 94% yields, respectively, as single regioisomers (Fig. 1g). As a control, we also evaluated the –SO_2_Tol-derived imine, and as suggested from the calculations (on the –SO_2_Ph imine), this was an unproductive reaction. We decided to move forward with –SO_2_F-substituted imines because they not only yielded the most efficient reaction but also produced sulfamoyl fluorides—‘click reagents’ known for their excellent orthogonality to various chemistries^45^—and have been applied as covalent probes in chemical biology^46,47^. In addition, the required imines are accessible in a single step from the corresponding aldehydes using fluorosulfonyl isocyanate^24,48^. Before embarking on a study of the reaction scope, we revisited the choice of photocatalyst. Calculation of relevant reduction potentials suggested that the ITX catalyst may be too reducing to provide broad scope with respect to the imine components, so we switched to the less-reducing 3-fluoro variant, while maintaining a triplet energy matched to the imine^49^ (3-F-TX (−0.99 V) versus ITX (−1.16 V) calculated potentials, Supplementary Fig. 27; *E*_T_(3-F-TX) = 67.4 kcal mol^−1^, *E*_T_(imine) = 67.3 kcal mol^−1^, Supplementary Methods). For general reaction conditions, we used 10 equivalents of alkene to achieve full conversion of the imine; however, for some classes of alkene, this can be reduced to 3 equivalents, and in both cases, any unreacted alkene can be recovered after the reaction (Supplementary Methods).

### Imine reaction scope

**Fig. 2:**
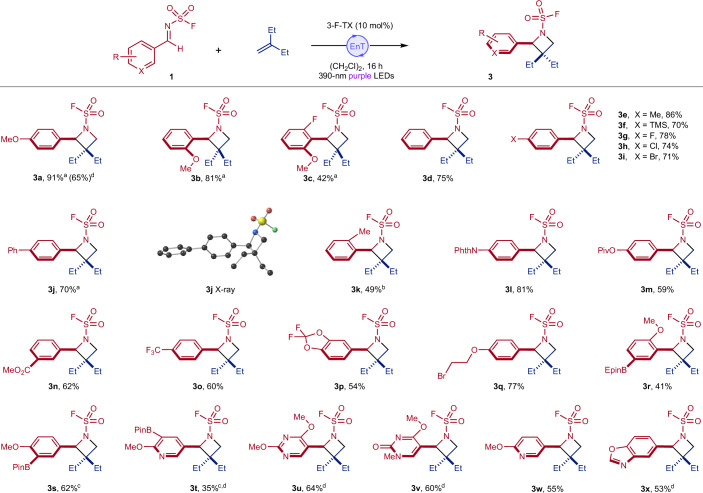
Scope of the imine component in aza Paternò–Büchi reactions. Reaction conditions: imine (0.20 mmol, 1.0 equiv.), alkene (2.0 mmol, 10 equiv.), (CH_2_Cl)_2_ (0.20 M) and 3-F-TX (0.020 mmol, 0.10 equiv.), 390 nm irradiation, 16 h. Isolated yields. ^a^ITX used as photocatalyst. ^b^3 equiv. alkene used. ^c^Isolated as the corresponding phenol after oxidation. ^d^Telescoped procedure (Supplementary Methods).

With optimized conditions for the aza Paternò–Büchi reaction in hand, we proceeded to assess the generality with respect to the imine component^24,37,50^ (Fig. 2). Encouragingly, the transformation is successful regardless of the electronics of the aryl imine; electron-rich, electron-poor and electron-neutral arenes, with a variety of substitution patterns, are well tolerated with reactions often proceeding in excellent yields. Sterically encumbered substrates can also be incorporated, with a number of bulky substituents successfully placed on the azetidine ring in spite of the proximity to a quaternary carbon (**3b**, **3c**, **3k**, **3r**, **3u** and **3v**). The mild nature of the conditions is highlighted by the tolerance of a range of sensitive functionalities including a selection of esters (**3m** and **3n**), primary alkyl bromides (**3q**) and boronic esters (**3r**–**3t**), which would probably not survive traditional basic conditions for forming azetidines via S_N_2 processes^51^. Alongside imines derived from arenes, a range of heteroaromatic imines can also be successfully used, including pyridines (providing access to four-membered derivatives of nicotine) (**3t** and **3w**), pyrimidines, pyrimidones (**3u** and **3v**) and a benzoxazole (**3x**). It is worth noting that heteroaromatic imines, or imine equivalents, are almost completely absent from the existing aza Paternò–Büchi literature. We also show that imines can be generated in situ from the corresponding aldehydes and used directly in the aza Paternò–Büchi reactions (**3a and** **3t**–**3v**). Imines featuring oxidatively labile groups on the aromatic ring, such as a phenol or sulfide, were not well tolerated and resulted in low yields (Supplementary Fig. 33).

### Alkene reaction scope

**Fig. 3:**
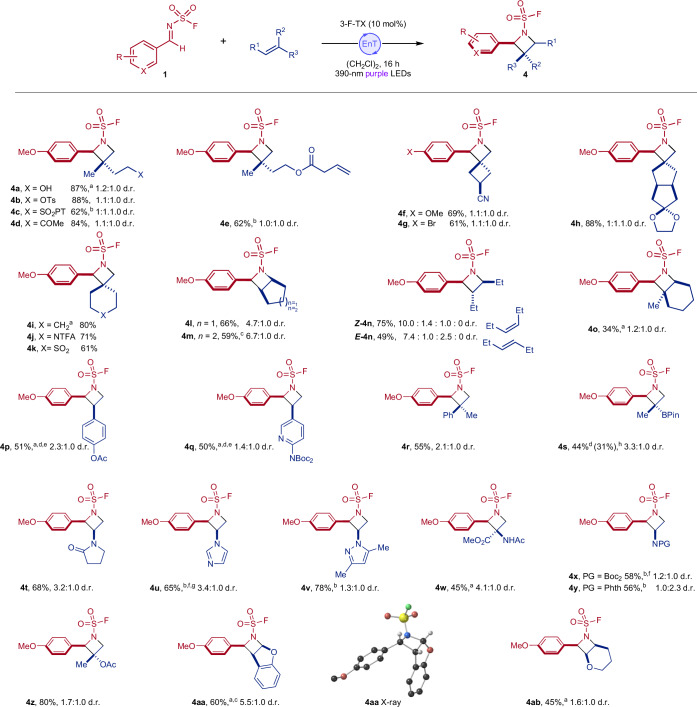
Scope of alkene component in aza Paternò–Büchi reaction. Reaction conditions: imine (0.20 mmol, 1.0 equiv.), alkene (2.0 mmol, 10 equiv.) and 3-F-TX (10 mol%) in (CH_2_Cl)_2_ (0.20 M), 390 nm irradiation, 16 h, isolated yields of both diastereomers. Diastereomeric ratios were determined by ^19^F NMR spectroscopic analysis of the crude reaction mixture. ^a^10 mol% ITX used as photocatalyst. ^b^3 equiv. of alkene used. ^c^Isolated yield of a single diastereomer. ^d^Yield determined using ^19^F NMR spectroscopy with 1,4-dFPh as an internal standard. ^e^Reaction time 3 days. ^f^Aldrithiol-2 (5 mol%) added. ^g^[Ir(dFCF_3_ppy)_2_dtbbpy][PF_6_] used and 427-nm light. ^h^Yield of alcohol after oxidative work-up. PT, 5-(1-phenyltetrazole). d.r., diastereomer ratio.

We next investigated the scope with respect to the alkene component. In addition to evaluating the generality of the chemistry, we also wanted to confirm that the reaction was not limited to specific substrate pairs, as is sometimes the case with singlet state reactivity, for example if exciplex formation is required^33^ (Fig. 3). Generally, the reaction works with a large range of alkene classes including, styrenes, di- and tri-alkyl substituted alkenes and B-, N- and O-heteroatom-substituted alkenes. Of note, this method is particularly adept at generating spirocyclic azetidines, which are attractive, three-dimensional scaffolds that often require long synthetic sequences to prepare (**4f**–**4k**)^52,53^. Tolerance of the chemistry to potentially reactive functionalities was also demonstrated here, including sulfonyl tetrazoles and phthalimides, groups that are labile under photoredox conditions (**4c** and **4y**)^54,55^, as well as groups with electrophilic centres such as ketones (**4d**), nitriles (**4f** and **4g**) and trifluoroacetamides (**4j**). Under these conditions, it is also possible to discriminate di-substituted and mono-substituted alkenes with excellent selectivity (**4e**, >20:1 regioisomer ratio). This selectivity arises because simple terminal alkenes are poorly reactive under the present conditions. Reactions with the matched *E-* and *Z*-isomers of 3-hexene converge to the same major diastereomer of product, which is consistent with the proposed triplet reactivity (***E***-**4n** and ***Z***-**4n**). With this methodology, it is possible to incorporate saturated and unsaturated heterocycles directly unto the azetidine core, including pyridine (**4q**), pyrrolidinone (**4t**), imidazole (**4u**) and pyrazine (**4v**), with the latter compound (**4v**) representing an azetidine derivative of crizotinib. It is also possible to use a dehydroalanine derivative to directly generate an α- and β-unnatural amino acid (**4w**), or an alkenyl boronic ester (**4s**), which both provide convenient groups for further functionalization. Owing to isolation challenges with imidazole-substituted azetidine **4u** and an impurity derived from the organic photocatalyst, this example was prepared using a commercially available Ir photocatalyst and 427 nm irradiation and serves to highlight the flexibility with regard to catalyst choice, which is useful for initiating specific optimization campaigns for a given substrate, as shown here. The generality of this method with respect to the alkene component is a direct consequence of the utilization of imine-derived triplet state intermediates, as opposed to activated alkene components, and enables the preparation of molecules that would otherwise require laborious multistep syntheses. However, simple terminal alkenes such as 1-octene, as well as alkenes conjugated with strong electron-withdrawing groups, remain challenging in this system and perform poorly (Supplementary Fig. 34).

### Product derivatization

**Fig. 4:**
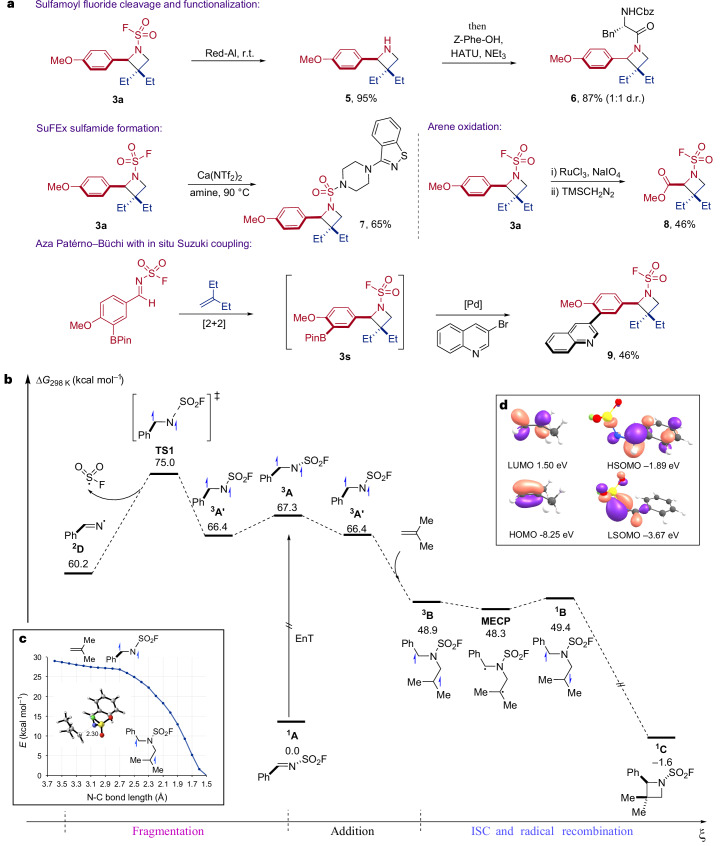
Derivatization of products and reaction profile. **a**, Derivatization of azetidine products (see Supplementary Methods for details). **b**, Reaction profile calculated using DFT SMD(1,2-dichloromethane)-M06-2X-D3/def2-TZVP in kcal mol^−1^. **c**, Potential energy surface calculated using DFT SMD(1,2-dichloromethane)-M06-2X-D3/def2QZVP//SMD(1,2-dichloromethane)-M06-2X-D3/def2-SVP. **d**, Frontier molecular orbitals of isobutene and ^**3**^**A′**. r.t., room temperature. HOMO, highest occupied molecular Orbital; HSOMO, highest singly occupied molecular orbital; LUMO, lowest unoccupied molecular orbital; LSOMO, lowest singly occupied molecular orbital.

To capitalize on the broad range of sulfamoyl fluoride-substituted azetidines available using the developed chemistry, we wanted to establish protocols that could further diversify the molecules that could be accessed (Fig. 4a). Azetidine **3a** was prepared on a gram scale (1.2 g, 77%) and used as a representative example. First, and most importantly, we were able to show that cleavage of the N–S bond in **3a**, effectively removing the –SO_2_–F substituent, could be achieved under mild conditions using Red-Al at room temperature. The corresponding N–H azetidine **5** was isolated in 95% yield, demonstrating the –SO_2_F functionality behaving as a traceless activating group in the [2 + 2] reaction and leaving no vestigial groups appended to the azetidine core. In place of isolating the free N–H azetidine, the crude reaction mixture following reaction with Red-Al could be subjected directly to peptide coupling conditions and joined with N-Boc phenyl alanine, to provide amide **6** in 87% yield. Sulfur–fluoride exchange reaction of the sulfamoyl fluoride present in **3a** was achieved using the Lewis-acid assisted method developed by Ball and colleagues^56,57^, providing sulfamide **7**. Given the vast range of amines that are commercially available, the ability to derivatize the aza Paternò–Büchi adducts directly with these building blocks suggests that library preparation around sulfamides of type **7** could be a profitable endeavour for medicinal chemistry applications. Using RuO_4_ facilitated oxidative cleavage of the paramethoxyphenyl group to deliver the carboxylic acid derivative **8**, which allows expedient access to synthetic proline homologues and provides opportunities for further decarboxylative functionalization^58^. The clean profile of the key [2 + 2] reaction simplifies the direct use of the crude reaction mixtures; for example, boronic ester containing azetidine **3s** was subjected directly to Suzuki–Miyaura coupling conditions with 3-Br quinoline, to yield biaryl **9**.

### Experimental and computational mechanistic investigation

To ascertain a detailed understanding of the key aza Paternò–Büchi reaction, we undertook a series of additional experimental and computational investigations. Stern–Volmer quenching studies confirmed that the imine is the most efficient quencher in the reaction (*K*_sv_ = 2.816 mM^−1^; Supplementary Fig. 6) and that the alkene is not quenched by 3-F-TX (Supplementary Fig. 8). Continuing our computational investigation, we calculated the full reaction profile for the reaction (Fig. 4b); following addition of triplet aldimine ^**3**^**A′** into isobutene, the resulting 1,3-diradical ^**3**^**B** undergoes intersystem crossing via an approximately (near) degenerate minimum energy crossing point to give singlet diradical ^**1**^**B**, which subsequently collapses to provide azetidine ^**1**^**C**. In this DFT study, we were unable to locate a transition state for the addition with isobutene leading to the observed regioisomer (Fig. 4c); however, Born–Oppenheimer molecular dynamics simulations provided multiple trajectories that directly led to formation of observed product, which provides support for a fast barrierless process. Our calculations also shed light on the excellent regioselectivity observed in the [2 + 2] reactions: while initial addition at nitrogen is barrierless, C-addition proceeds with a 7.4 kcal mol^−1^ energy barrier (Supplementary Methods). We propose this selectivity is a consequence of a nucleophilic alkene component donating electrons into the LSOMO of the electrophilic imine, with the lowest singly occupied molecular orbital (LSOMO) localized predominantly on N (Fig. 4d). Clearly, the sulfamoyl fluoride group plays a role beyond raising the S–N σ* energy and preventing fragmentation; it also contributes to the required reactivity and regiocontrol, enabling the successful development of the aza Paternò–Büchi reaction.

## Conclusions

In this study, we have shown that appropriately designed aryl imines are competent substrates for intermolecular aza Paternò–Büchi reactions using energy-transfer catalysis. The imines are required to feature an N-sulfamoyl fluoride substituent, but provided this condition is met, efficient aza Paternò–Büchi reactions are achieved. By design, the azetidine products from these reactions all feature an N-sulfamoyl fluoride; this group can be simply removed to provide the N–H heterocycles, or can be readily converted into a sulfamide unit. The scope of the aza Paternò–Büchi reactions with respect to both reaction components is broad, with aryl imines bearing a variety of substituents, as well as the first examples of heteroaryl imines, being successfully used. The alkenes used include 1,1- and 1,2-di-substituted, as well as trisubstituted alkenes, along with multiple examples of activated terminal alkenes bearing synthetically valuable groups such as amides and aza-heterocycles. Enol ethers, enamides, unsaturated amino acids, and an aromatic heterocycle also perform well under the reaction conditions. Some limitations remain with respect to the alkene component, and similarly, the imines must be aryl imines, as this is necessary to achieve effective energy transfer. Nevertheless, the reactions we report show exceptional scope for intermolecular aza Paternò–Büchi reactions that use acyclic imines. These transformations show that it is possible for correctly designed imines to generate synthetically useful triplet intermediates. With this precedent established, we anticipate many further applications of these elusive reactive intermediates.

## Methods

The general procedure for the energy-transfer-mediated intermolecular aza Paternò–Büchi reaction involved the following. Fluorosulfamoyl imine (0.20 mmol, 1.0 equiv.), 3-F-TX (4.6 mg, 0.020 mmol, 10 mol%), ITX (5.1 mg, 0.020 mmol, 10 mol%) or [Ir(dFCF_3_ppy)_2_(dtbbpy)][PF_6_] (2.2 mg, 0.002 mmol, 0.01 equiv.), aldrithiol-2 (0 or 2.2 mg, 0.00 or 0.01 mmol, 0 or 5 mol%) and (if solid) alkene (0.60–2.0 mmol, 3.0–10 equiv.) were added to an oven-dried tapered microwave vial equipped with a stirrer bar and a septum. The vial was evacuated and backfilled with nitrogen three times, followed by addition of anhydrous (CH_2_Cl)_2_ (1.0–3.0 ml, 0.067–0.20 M) and (if liquid) alkene (0.60–2.0 mmol, 3.0–10 equiv.). The reaction was then stirred under irradiation with 390-nm light-emmitting diode (LED) (3-F-TX and ITX) or 427-nm LED [Ir(dFCF_3_ppy)_2_(dtbbpy)][PF_6_] at ambient temperature for 16 h, with the fans turned on. The reaction mixture was diluted with CH_2_Cl_2_, and the solvent was removed under reduced pressure, followed by purification via flash column chromatography.

## Supplementary information

Supplementary Information Supplementary methods, Tables 1–24, Figs. 1–34 and references.

Supplementary Data 1 CIF file of the crystal structure of compound **3j**.

Supplementary Data 2 CIF file of the crystal structure of compound **4aa**.

Supplementary Data 3 Cartesian (*x*, *y*, *z*) of the computational section.

## Data Availability

Experimental procedures and characterization data are provided in the Supplementary Information. Crystallographic data for the structures reported in this Article have been deposited at the Cambridge Crystallographic Data Centre, under deposition numbers CCDC2375437 (**3j**) and 2375438 (**4aa**). Copies of the data can be obtained free of charge at https://www.ccdc.cam.ac.uk/structures/. Data are available from the corresponding author upon request.
