# Supplementary material for: A modular synthesis of azetidines from reactive triplet imine intermediates using an intermolecular aza Paternò–Büchi reaction
Source: Nat Catal. 2025 Sep 5;8(9):939–47. doi: 10.1038/s41929-025-01405-7 (PMC12460163; doi:10.1038/s41929-025-01405-7)
Supplement: Supplementary file 1 — Supplementary methods, Tables 1–24, Figs. 1–34 and references. [file 41929_2025_1405_MOESM1_ESM.pdf]

nature catalysis

Supplementary information

https://doi.org/10.1038/s41929-025-01405-7

A modular synthesis of azetidines from reactive triplet imine intermediates using an intermolecular aza Paternò–Büchi reaction

In the format provided by the authors and unedited

Contents

Supplementary Methods 1

- General Information 1
- Photochemical Reaction Setup 2
- Control experiments 3
- Screen of Sulfonyl Imines 3
- Photocatalyst screen 3
- Solvent Screen 4
- Concentration 4
- Equivalents of alkene 5
- Equivalents of alkene (cyclic) 5
- Light and photocatalyst controls 6
- Cyclic-Voltametry 6
- Stern-Volmer 8
- Computational Studies and Data 12
- Computational Methods 12
- Photochemistry of sulfonyl imines 13
  - Triplet energy 13
  - MECP 14
  - Isomerisation 15
- Fragmentation of triplet sulfonyl aldimines 16
- Addition of triplet sulfonyl aldimines into alkenes 17
  - Results and Discussion 17
  - Potential energy surfaces 18
    - Alternative Transition States 22
  - Regioselectivity of Addition 23
- Intersystem crossing 25
- Alternative Alkene 26
  - Addition of Imine 26

Intersystem Crossing 28
Calculated reduction potentials 29
Photochemistry and Electrochemical Studies on Thioxanthone Photocatalysts 31
Born-Oppenheimer Molecular Dynamics 31
Table of thermodynamic properties 34
Energy-transfer mediated intermolecular aza-Paternò-Büchi reaction (GP1) 37
Azetidine characterisation 38
Telescoped energy-transfer mediated intermolecular aza-Paternò-Büchi reaction from aldehyde 74
Azetidine characterisation 74
One-gram scale reaction and derivatisation of azetidine sulfamoyl fluorides 77
One-gram scale reaction 77
Cleavage of -SO_2F moiety 78
Telescoped amide coupling 78
Calcium Bistriflimide-mediated SuFEx 80
RuO_4 oxidative cleavage of aromatic group 80
Telescoped Suzuki coupling 81
Substrate Synthesis 82
Synthesis of sulfamoyl fluoride imines (GP2) 82
Imine product characterisation 82
Aldehydes 92
Alkenes 96
Characterisation of N-Triflyl system 102
Procedure for aza-Paternò-Büchi reaction of 2a 102
Preparation of imine I-43 103
Crystallographic Data 104
Unsuccessful Substrates 106
Imines 106
Alkenes 107
Spectroscopic Data 108
Azetidines 108
Derivitisation 223

Sulfamoyl fluoride Imines 229
Aldehydes 261
Alkenes 269
N-triflyl aldimine 283
Supplementary References 285

Supplementary Methods

General Information

All reactions were performed under an atmosphere of nitrogen with constant magnetic stirring, unless otherwise stated, using clean, oven dried glassware. All inert gases were sourced from the University of Oxford's internal supplies and dried through a $CaCl_2$ drying columns. Reactions were monitored by thin-layer chromatography (TLC) which was performed on Merck Millipore Kieselgel 60 PF254 pre-coated aluminium backed TLC sheets and visualized by UV lamp (λ = 254 nm and 365 nm) and/ or staining using $KMnO_4$, phosphomolybdic acid or anisaldehyde stain. All chemicals used were obtained from commercial sources, unless otherwise stated, including Sigma Aldrich, Fluorochem, Alfa Aesar and Strem. 5-vinyl-5H-thianthren-5-ium tetrafluoroborate,[1] 3-fluoro-9H-thioxanthen-9-one[2] and 1,1,2,2-tetraethylethylene glycol (EPin)[3] were prepared according to literature. $Pd(PPh_3)_4$ was triturated with MeOH before use and stored under N_2 at -20 °C. Dry solvents were obtained from the University of Oxford internal solvent drying system (Innovative Technology Inc. PS-400-7), wherein they are purified through dried alumina columns. Solvents used in the [2+2] photocyclisation were kept under an atmosphere of N_2 and stored over 3Å molecular sieves. Solvents used for purification, or not needed dry, were HPLC grade purchased from either Alfa Aesar or Sigma Aldrich. When referring to the solvent 'Petrol' and 'pet. ether', what is meant is the fractions of petroleum ether which boil within the range 40 – 60 °C. Flash column chromatography was performed by loading the compound as an oil or concentrated solution onto a column, using Geduran® Si 60, 40–63-micron silica gel, and then run with the indicated eluent system.

^{1}H NMR, ^{13}C NMR and ^{19}F NMR spectra were recorded on a Brüker AVIII spectrometers (400-700 MHz). Acquisitions were carried out at rt unless otherwise stated. Chemical shifts (δ/ ppm) are reported in parts per million (ppm) and referenced relative to the residual solvent peak; ^{1}H NMR: $CHCl_3$ (7.26 ppm), ^{13}C NMR: $CHCl_3$ (77.2 ppm). Coupling constants (*J*) are given in Hertz (Hz) and rounded to the nearest 0.5 Hz. ^{1}H NMR is recorded to two decimal places and ^{13}C NMR is recorded to 1 decimal place. Proton multiplicity is assigned using the following abbreviations: singlet (s), doublet (d), triplet (t), quartet (q), pentet (p), multiplet (m), broad (br). Assignments were made using the help of a variety of 2D NMR experiments (COSY, HSQC, HMBC and NOESY).

High resolution mass spectra were obtained via the University of Oxford in-house service, utilising using either a Thermo Exactive High-Resolution Orbitrap FTMS spectrometer or Waters BioAccord TOF spectrometer under electrospray ionisation conditions (ESI). Values quoted are a ratio of mass to charge in Daltons to four decimal places on the high resolution. For high resolution mass spectra, the mass found was compared to the mass calculated from the monoisotopic molecular formula, and all results were found to be within a 5 ppm error of the calculated values.

Melting point values were found using a Reich Melting Point Apparatus and are reported uncorrected. Infrared spectra were determined using neat samples with a Brüker Tensor 27 FT-IR spectrometer with an internal range of 600-4000 cm^{-1} and all absorptions are given in wavenumbers (cm^{-1}).

Crystals **3j** and **4aa** were grown by layer diffusion of pentane on top of a concentrated solution of EtOAc and vapour diffusion of pentane into a concentrated solution of 4aa in EtOAc.

All UV-vis absorption spectra were recorded at 298 K, with temperature control by a PTP-1 Peltier unit from Perkin Elmer, with baseline correction.

The HPLC data was obtained from an Agilent Technologies 1200 series HPLC Hypersol ODS, 5 µm column (100 × 4.0 MM) using an extended 20 min method H_2O (0.1 % v/v H_3PO_4): Acetonitrile 10-95% (17 min) to 100% (18.5 min) to 10% (20 min).

For the relative stereochemistry nomenclature of final ring diastereoisomers, the terms *R** and *S** have been used denote the configuration of a stereocenter, relative to that of a co-existing stereocenter, within a racemic compound. This is not to be confused with absolute stereochemical terms *R* and *S*, used to depict absolute configuration within an enantiopure compound. Accordingly, a compound of number ***A***, with a name prefixed x*R**,z*S** ((x*R**,z*S**)-***A***), will be the diastereoisomer of compound ***A***, prefixed with x*R**,z*R** ((x*R**,z*R**)-***A***), where x and z are integers representing the stereocenter position.

Photochemical Reaction Setup

427nm Blue LEDs and 390 nm Purple LEDs where specified, refer to a 45W Kessil PR160L – 427 nm lamp and 52W Kessil PR160L – 390 nm lamp respectively, mounted in an EvoluChem™ PhotoRedOx Box device. The reactions were run at 100% percent light intensity with the built-in fans on unless otherwise specified. Reactions were carried out in 0.5 – 2 mL tapered Microwave Vials, fitted with a PTFE septum.

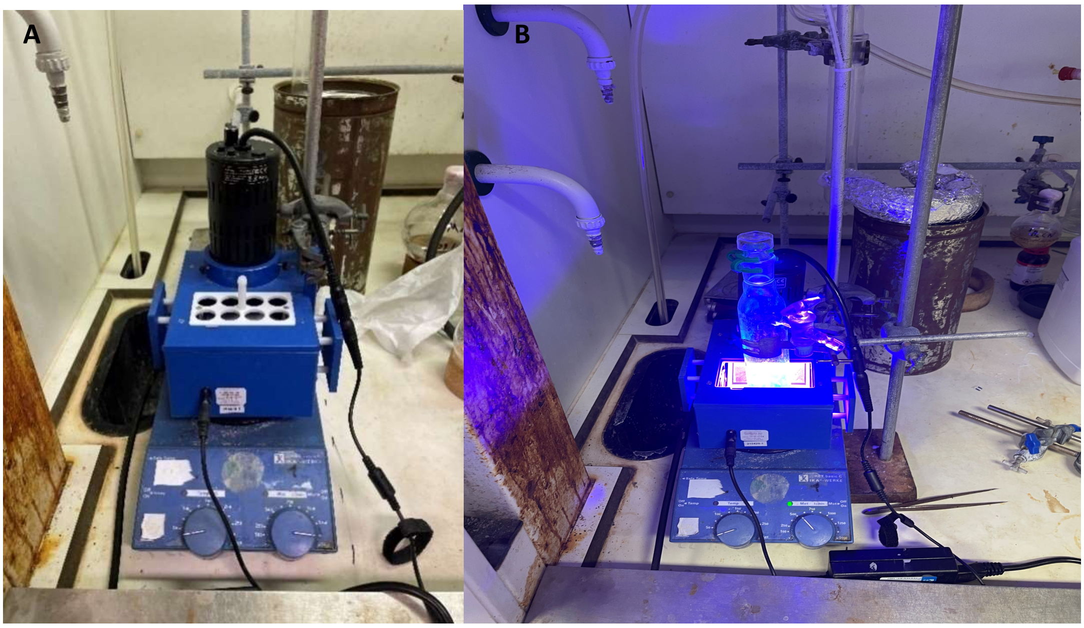

Supplementary Fig. 1A Set-up for 0.2 mmol photochemical reactions. **B** Set-up for 5 mmol photochemical reactions.

Control experiments

Screen of Sulfonyl Imines

Supplementary Table 1 Screen of sulfonyl aldimines.

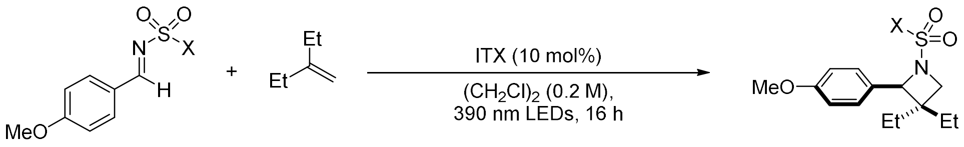

1.0 eq. 10 eq.

entry	X	SM remaining[a] / %	yield[a] / %
1	4-MePh	0	0
2	CF_3	0	56
3	F	0	94

Reaction conditions: Sulfamoyl fluoride imine (0.1 mmol), 3-methylenepentane (1.0 mmol) and ITX (10 mol%), CH_2Cl_2 (0.5 ml), rt, 16 h. [a]Quantities determined using $^1H/^{19}F$ NMR spectroscopy using 1,4-difluorobenzene as an internal standard

Photocatalyst screen

Supplementary Table 2 Screen of selected photocatalysts.

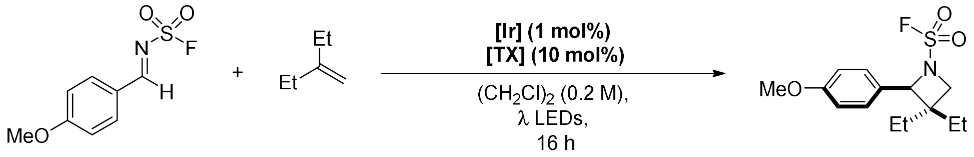

1, 1.0 eq. 10 eq. **3a**

entry	Photocatalyst	Triplet Energy / kcalmol^{-1}	λ /nm	1 remaining[a] / %	yield of 3a[a] / %
1	none	-	390	61	15
2	2-OMe-TX	57.8	390	38	33
3	ITX	63.5	390	0	94
4	TX	65.4	390	0	93
5	3-F-TX	67.4	390	0	97 (94)[b]
6	*fac*-Ir(ppy)$_3$	54.5	450-455	100	0
7	[Ir(dFCF$_3$ppy)$_2$(dtbbpy)]PF$_6$	60.1	450-455	0	95

Reaction conditions: Sulfamoyl fluoride imine (0.1 mmol), 3-methylenepentane (1.0 mmol) and ITX (10 mol%), CH_2Cl_2 (0.5 ml), rt, 16 h. [a]Quantities determined using ^{19}F NMR spectroscopy relative to 1,4-difluorobenzene as an internal standard [b]Isolated yield in parentheses.

Solvent Screen

Supplementary Table 3 Screen of selected solvents.

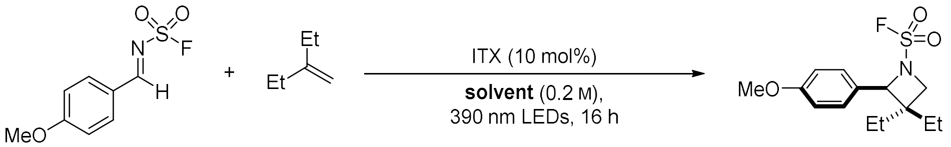

1, 1.0 eq. 10 eq. **3a**

entry	solvent	1 remaining[a] / %	yield of 3a[a] / %
1	$(CH_2Cl)_2$	0	94
2	CH_2Cl_2	0	92
3	MeCN	0	82
4	EtOAc	0	85
5	DMF	0	39
6	PhF	0	94
7	THF	0	50

Reaction conditions: Sulfamoyl fluoride imine (0.1 mmol), 3-methylenepentane (1.0 mmol) and ITX (10 mol%), CH_2Cl_2 (0.5 ml), rt, 16 h. [a]Quantities determined using ^{19}F NMR spectroscopy relative to 1,4-difluorobenzene as an internal standard

Concentration

Supplementary Table 4 Variation of concentration.

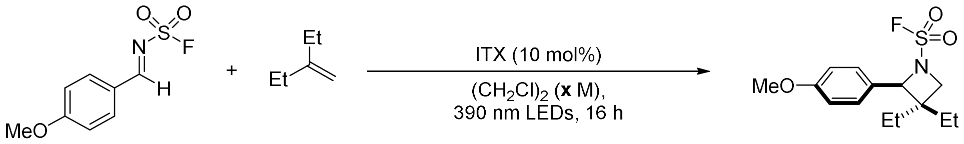

1, 1.0 eq. 10 eq. **3a**

entry	concentration / M	1 remaining[a] / %	yield of 3a[a] / %
1	0.1	0	92
2	0.2	0	94
3	0.4	0	91

Reaction conditions: Sulfamoyl fluoride imine (0.1 mmol), 3-methylenepentane (1.0 mmol) and ITX (10 mol%), CH_2Cl_2 (0.5 ml), rt, 16 h. [a]Quantities determined using ^{19}F NMR relative to 1,4-difluorobenzene as an internal standard

Equivalents of alkene

Supplementary Table 5 Screen of alkene equivalents.

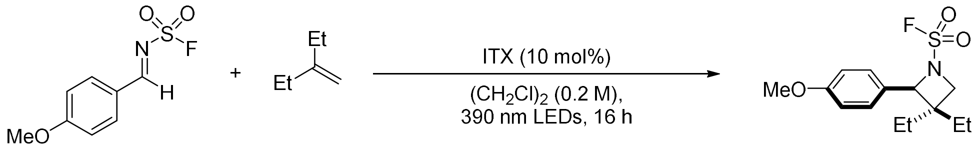

1, 1.0 eq. **x** eq. **3a**

entry	alkene loading (eq.)	1 remaining[a] / %	yield of 3a[a] / %
1	10	0	94
2	5.0	0	98
3	3.0	0	100
4	1.0	0	70

Reaction conditions: Sulfamoyl fluoride imine (0.1 mmol), 3-methylenepentane (1.0 mmol) and ITX (10 mol%), CH_2Cl_2 (0.5 ml), rt, 16 h. [a]Quantities determined using ^{19}F NMR spectroscopy relative to 1,4-difluorobenzene as an internal standard

Equivalents of alkene (cyclic)

Supplementary Table 6 Screen of alkene equivalents for an acyclic alkene.

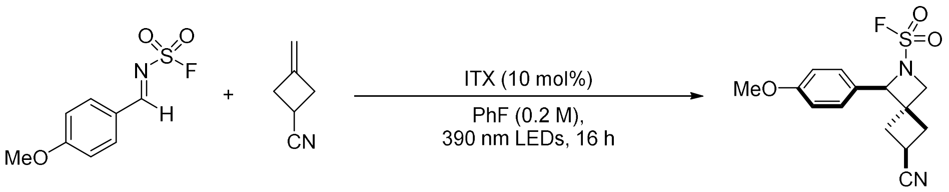

1, 1.0 eq. **x** eq. **4f**

entry	alkene loading / eq.	1 remaining[a] / %	yield of 4f[a] / %
1	12	0	73
2	7.5	0	73
3	3.0	0	68
4	1.0	0	58

Reaction conditions: Sulfamoyl fluoride imine (0.1 mmol), 3-methylenecyclobutane-1-carbonitrile (1.2 mmol) and ITX (10 mol%), fluorobenzene (0.5 ml), rt, 16 h. [a]Quantities determined using ^{19}F NMR spectroscopy relative to 1,4-difluorobenzene as an internal standard.

Light and photocatalyst controls

Supplementary Table 7 Control experiments.

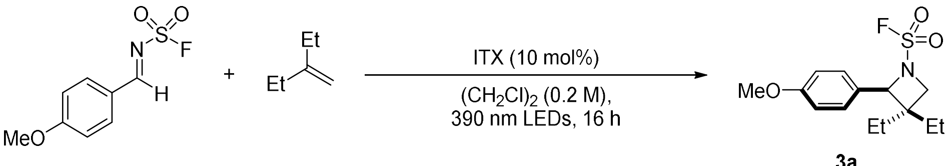

1, 1.0 eq. 10 eq. **3a**

entry	variations from conditions above	1 remaining[a] / %	yield of 3a[a] / %
1	none	0	94
2	no ITX	61	15
3	no light	100	0
4	no, light, no ITX, 80°C	68	0

Reaction conditions: Sulfamoyl fluoride imine (0.1 mmol), 3-methylenepentane (1.0 mmol) and ITX (10 mol%), CH_2Cl_2 (0.5 ml), rt, 16 h.[a]Quantities determined using ^{19}F NMR spectroscopy relative to 1,4-difluorobenzene as an internal standard

Cyclic-Voltametry

Cyclic voltammetry (CV) measurements were recorded on a Autolab PGSTAT101 Potentiostat, using a 3-electrode setup: a glassy carbon working electrode, a platinum wire counter electrode and a Ag|$AgNO_3$ (100 mM in MeCN) reference electrode. Parameters for CV: 25 mVs^{-1} scan rate. Measurements at a concentration of 0.05 M and were performed in MeCN with tetrabutylammonium hexafluorophosphate (Bu_4NPF_6, 0.1 M) as the electrolyte. Sample solutions were deoxygenated by purging with N_2 for 2 – 3 minutes before the measurements. Ferrocene was added after the experiment and the peaks reported are referenced to the Fc^+/Fc peak. The reported potentials were taken at the half-height of the cathodic peak of the compounds as all redox processes were irreversible. The reduction potential E(**1**/**1**$^-$) = -1.12 V and the oxidation potential E(**1**/**1**$^+$) = 2.04 V was obtained.

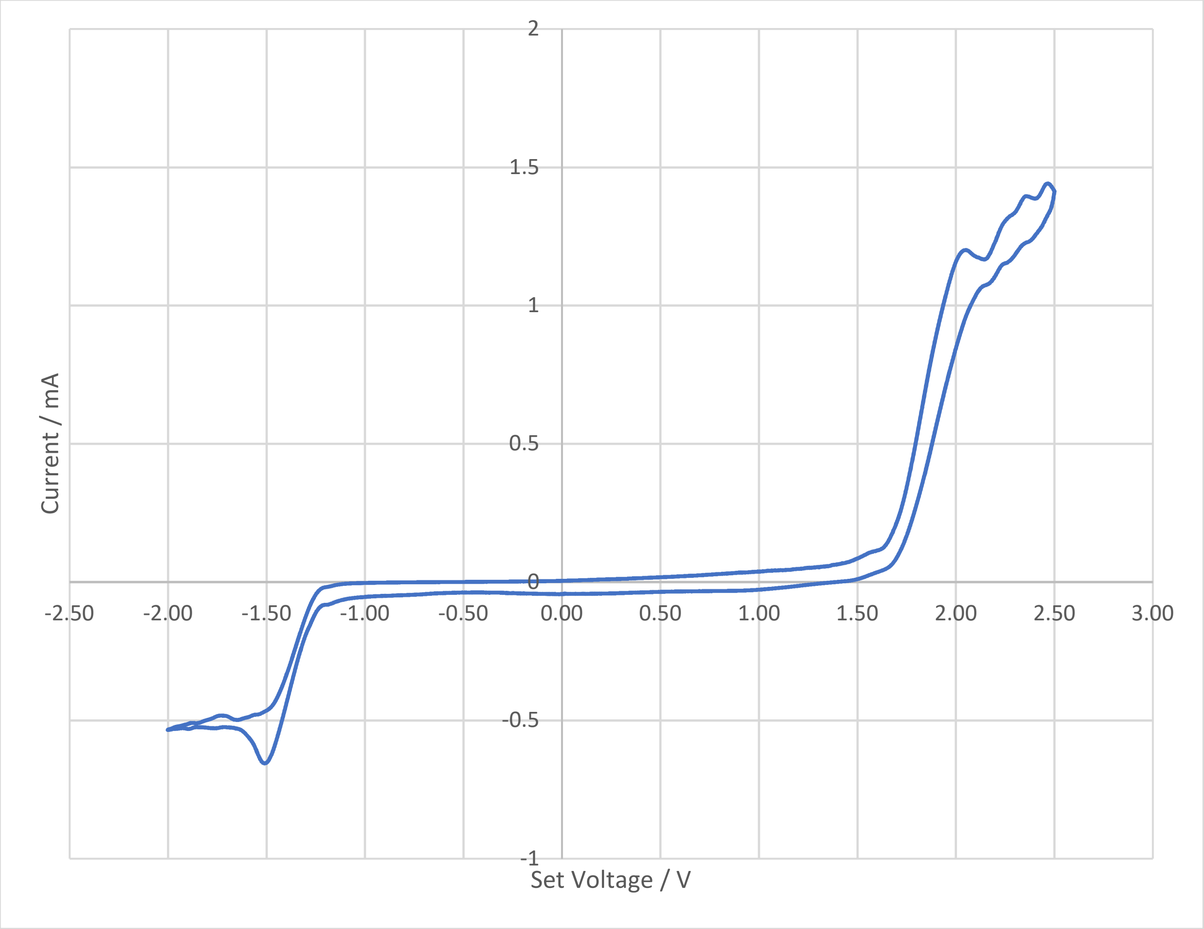

Supplementary Fig. 2 Cyclic voltammetry of **1** for reduction and oxidation in the absence of ferrocene.

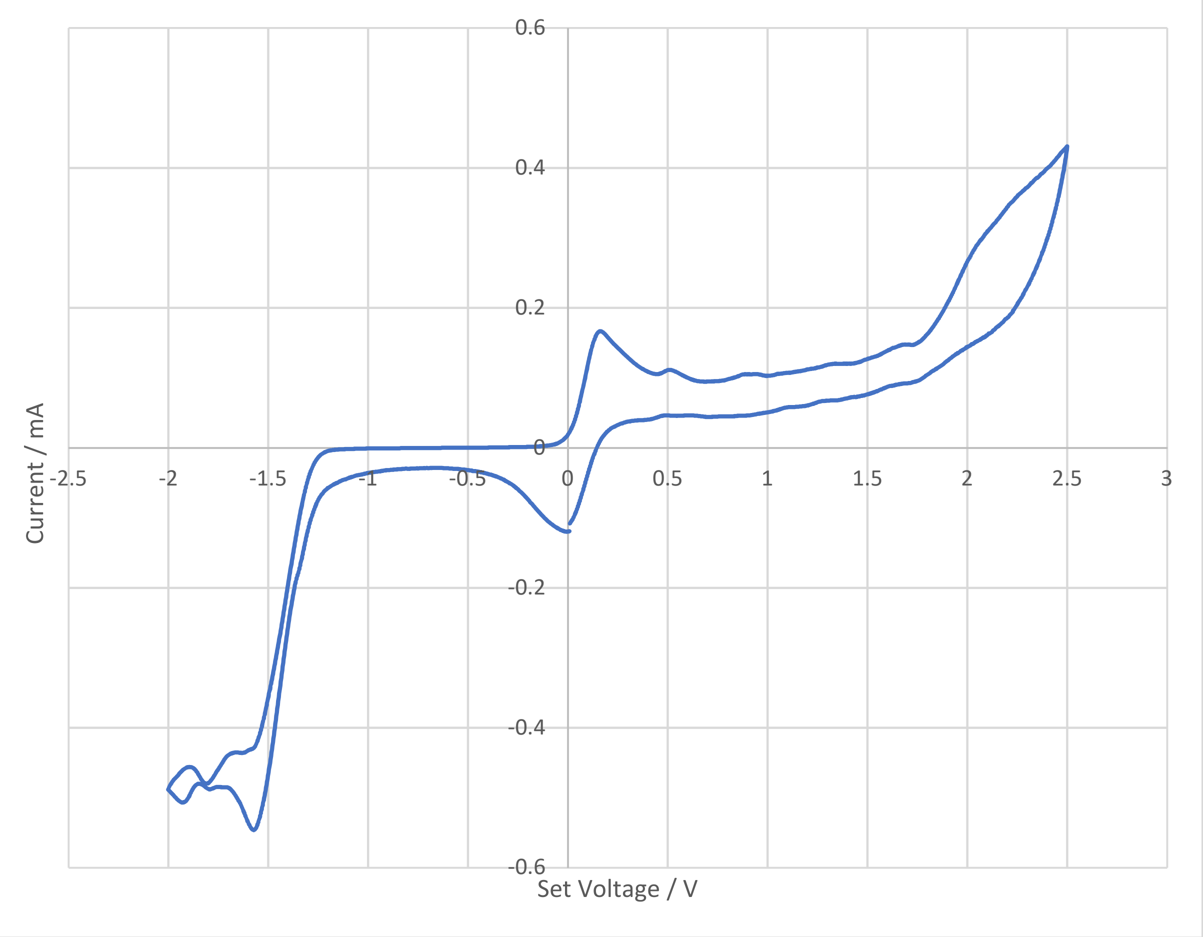

Supplementary Fig. 3 Cyclic voltammetry of **1** for reduction in the presence of ferrocene.

Stern-Volmer

Fluorescence spectra were acquired at 298 K using an Edinburgh Instruments FS5 spectrofluorometer operating Fluoracle® software, and equipped with a xenon arc lamp (providing 230–1000 nm excitation range), a thermostatic sample holder (SC-20) and an R13456 PMT detector (200–950 nm spectral coverage, Hamamatsu). Spectroscopic measurements were conducted using quartz cuvettes (10 mm path length, Starna Scientific Ltd).

Fluorescence quenching of [Ir(dFCF$_3$ppy)$_2$(dtbbpy)][PF$_6$] was investigated by measuring the measuring the emission spectrum of [Ir(dFCF$_3$ppy)$_2$(dtbbpy)][PF$_6$] in the presence of the reaction components and varying the concentration under an atmosphere of N_2. A solution of [Ir(dFCF$_3$ppy)$_2$(dtbbpy)][PF$_6$] (2 μM) was treated with (0.05 – 0.25 mM) of 3-methylene pentane and 1. The samples were irradiated at 380 nm and luminescence was measured over a range of 365 – 600 nm (at λ_{max} = 496 nm). I/I_0 in the range of 0.00 – 0.15 mM was plotted against the concentration of alkene.

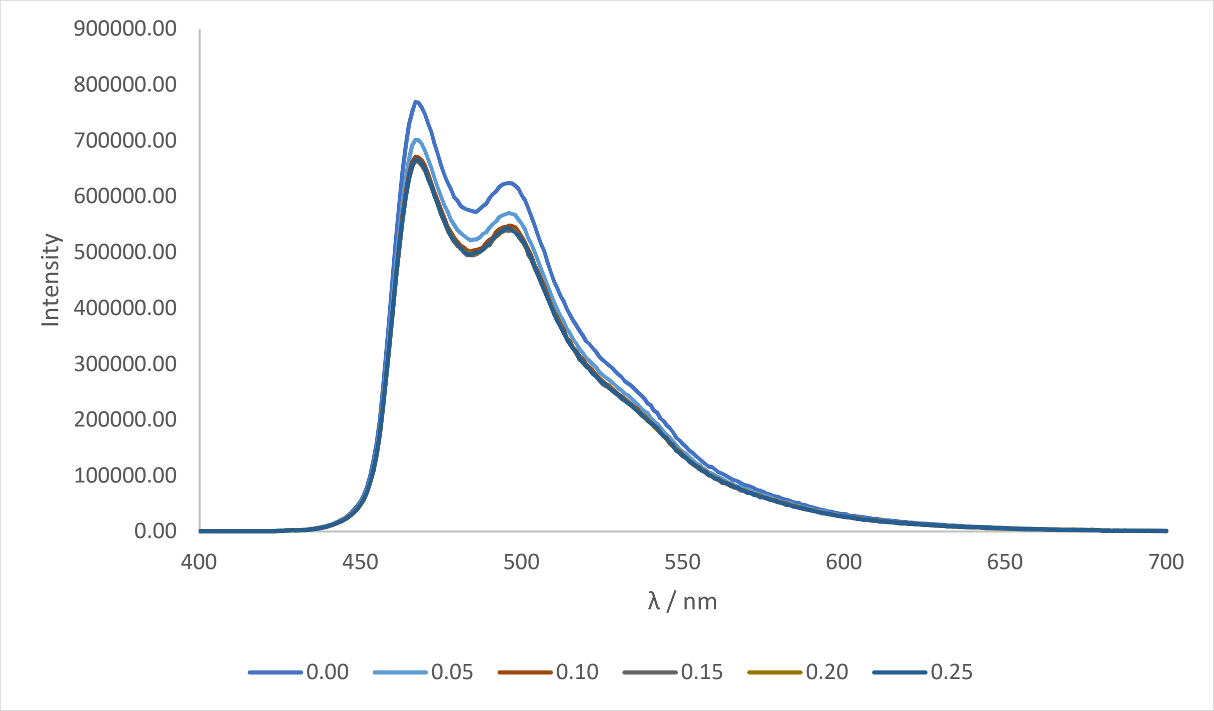

Supplementary Fig. 4 Fluorescence quenching of catalyst [Ir(dFCF$_3$ppy)$_2$(dtbbpy)][PF$_6$] in the presence of 3-methylene pentene at varying concentrations 3-methylene pentane in $(CH_2Cl)_2$ in mM.

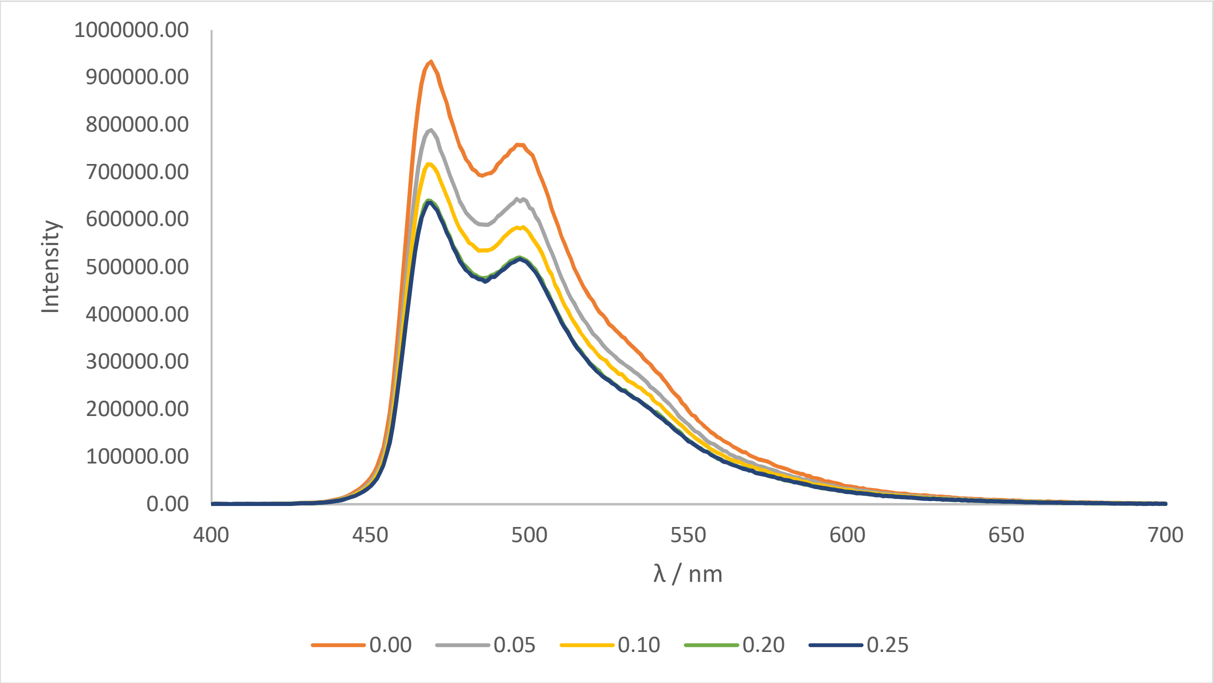

Supplementary Fig. 5 Fluorescence quenching of catalyst [Ir(dFCF$_3$ppy)$_2$(dtbbpy)][PF$_6$] in the presence **1** at varying concentrations in $(CH_2Cl)_2$ in mM.

Supplementary Table 8 Fluorescence quenching of catalyst [Ir(dFCF$_3$ppy)$_2$(dtbbpy)][PF$_6$].

	I_0/I	
**conc**	**1**	**3-methylene pentene**
0.00	1.00	1.00
0.05	1.19	1.09
0.10	1.30	1.14
0.15	1.43	1.16

The data indicates that the aldimine substrate is the most efficient quencher of the photocatalyst in the system.

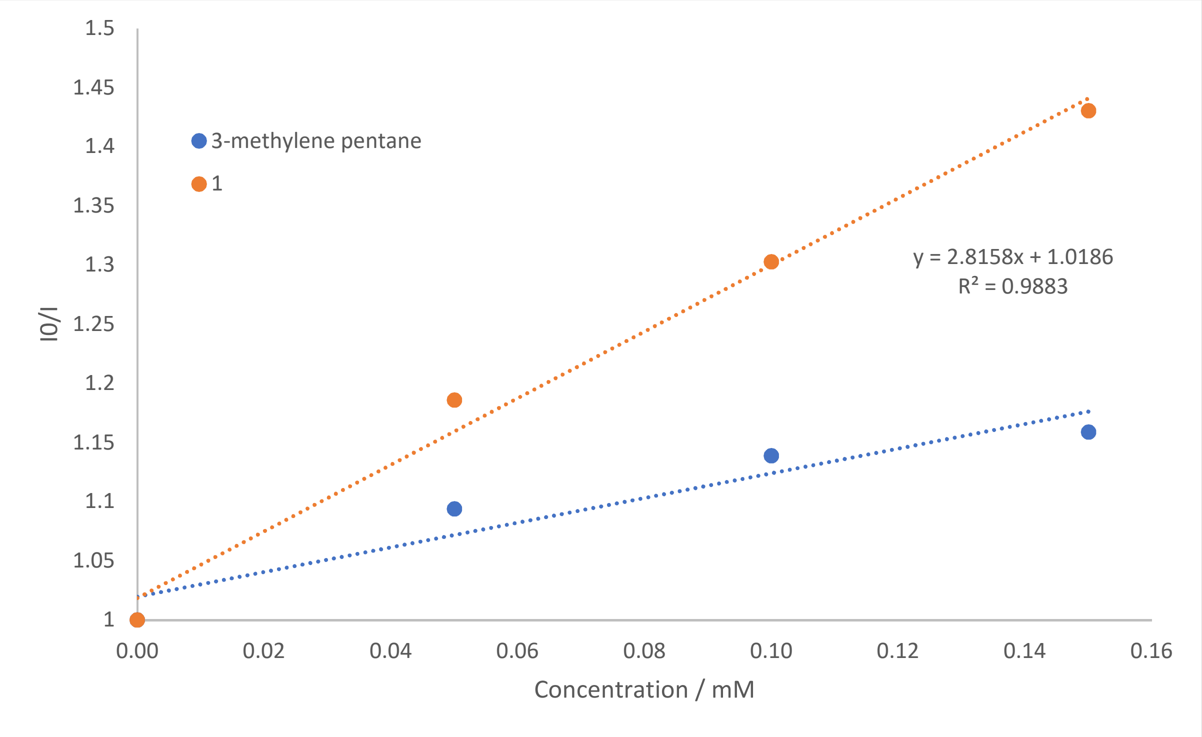

Supplementary Fig. 6 Stern-Volmer quenching of [Ir(dFCF$_3$ppy)$_2$(dtbbpy)][PF$_6$] using excitation at 380 nm.

Fluorescence quenching of 3-F-TX was investigated by measuring the emission spectrum of 3-F-TX in the presence of the reaction components and varying the concentration under an atmosphere of N_2. A solution of 3-F-TX (0.0125 mM) was treated with (0.10 – 0.30 mM) of 3-methylene pentane. The samples were irradiated at 355 nm and luminescence was measure over a range of 400 – 600 nm (at λ = 394 nm). I/I_0 in the range of 0.00 – 0.30 mM was plotted against the concentration of alkene. It was not possible to observe fluorescence quenching of 3-F-TX by **1** because the emission spectrum of **1**, under the irradiation conditions used for Stern-Volmer quenching of 3-F-TX, overlaps with that of 3-F-TX.

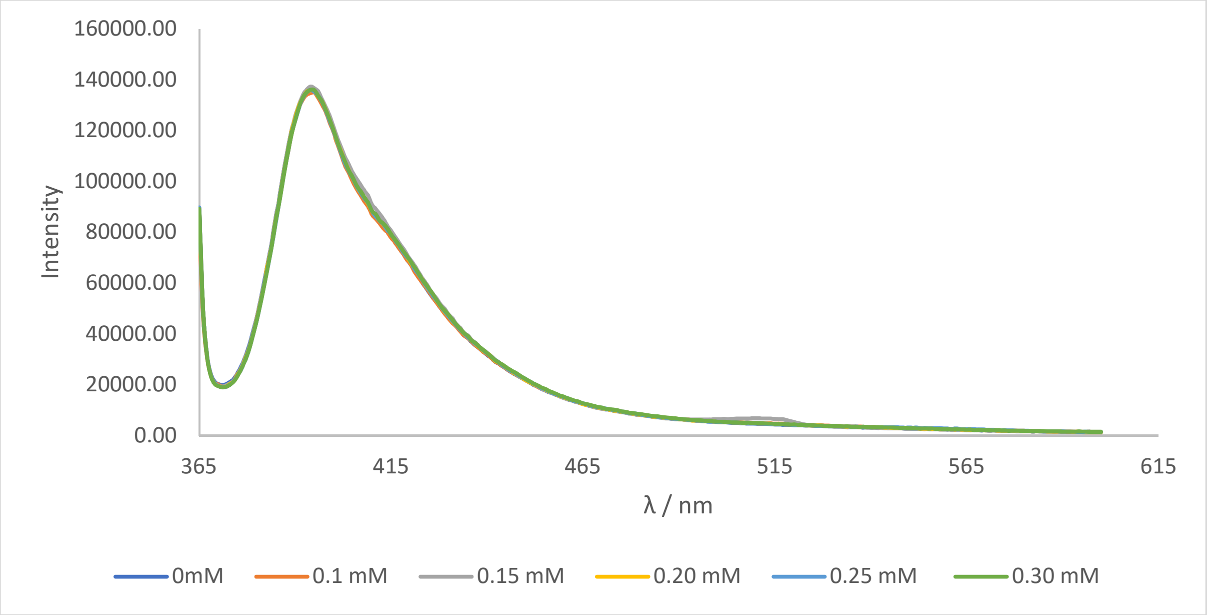

Supplementary Fig. 7 Fluorescence quenching of catalyst 3-F-TX in the presence of 3-methylene pentane at varying concentrations in $(CH_2Cl)_2$ in mM.

Supplementary Table 9 Fluorescence quenching of catalyst 3-F-TX.

	**I_0/I**
**Conc / mM**	3-methylene pentane
**0.00**	1.00
**0.10**	0.99
**0.15**	1.01
**0.20**	1.00
**0.25**	1.00
**0.30**	1.00

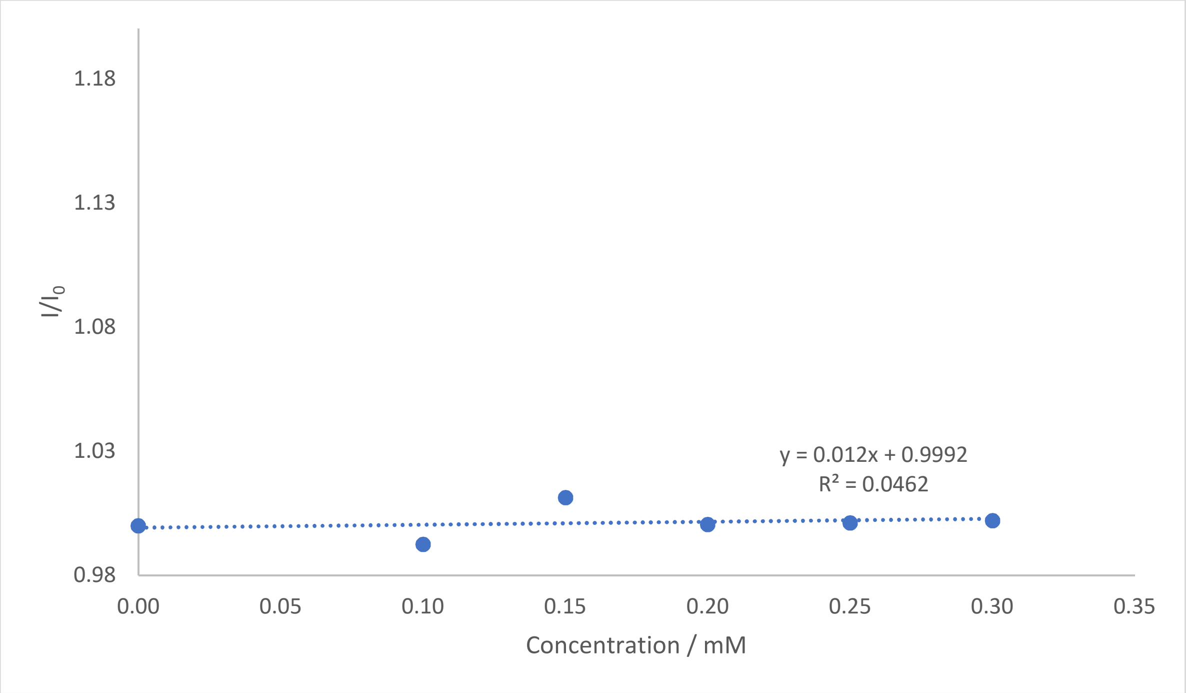

Supplementary Fig. 8 Stern-Volmer quenching of 3-F-TX in $(CH_2Cl)_2$ using excitation at 355 nm.

The data indicates that the alkene is not being quenched by the photocatalyst.

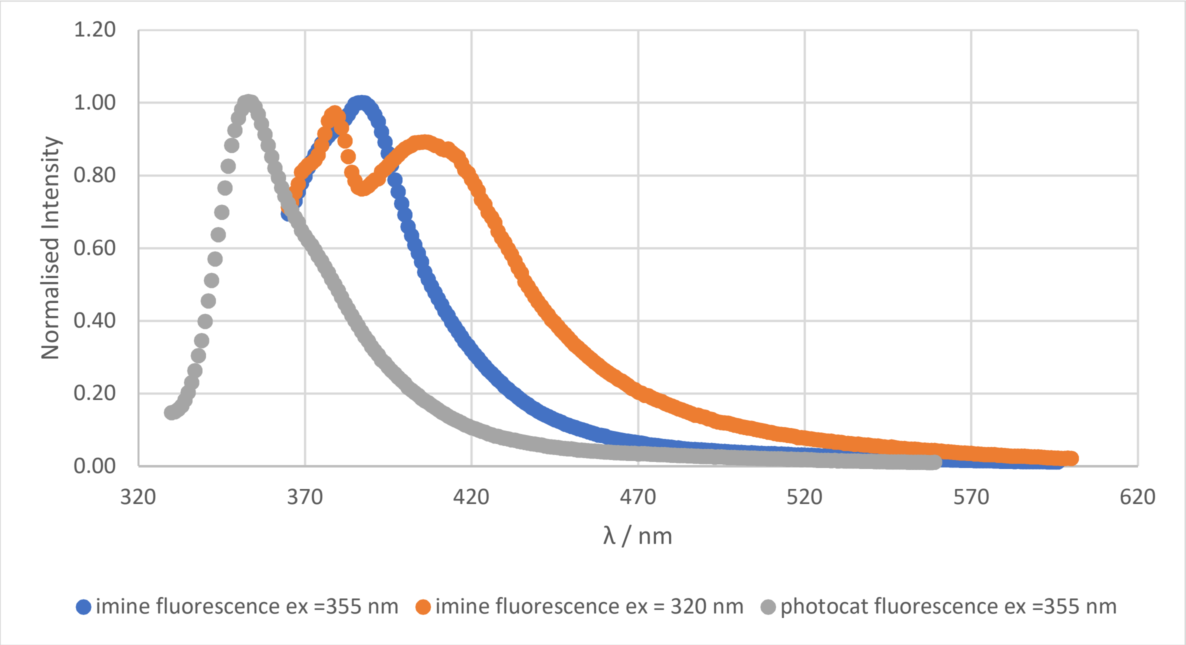

Supplementary Fig. 9 Normalised fluorescence spectra of **1** at multiple excitation wavelengths and 3-F-TX fluorescence using excitation at 355 nm.

Computational Studies and Data

Computational Methods

All calculations were carried out using the ORCA suite of programs (version 5.0.3).[4,5] The resolution of identity chain of spheres exchange (RIJCOSX) was used to speed up the SCF process,[6-9] employing the def2/J Coulomb-fitting auxiliary basis set. The integration grid "defgrid3" was selected for energies, implicit solvation was introduced by employing the SMD explicit solvent model using default parameters for 1,2-dichloroethane.[10] Geometries of all relevant stationary points were optimized

using the M06-2X[11] functional on Ahlrich's def2-TZVP triple-zeta basis set[12] and Grimme D3 atom-pairwise dispersion correction.[13,14] M06-2X was chosen due to its high performance across a range of benchmarking studies for main group chemistry.[15-17] All energies were corrected to 1M standard state at 298 K(addition of 1.89 kcalmol^{-1} to every species). Transition states contained a singular imaginary vibration, which were further verified by the connection of the transition states to reactants and products *via* the intrinsic reaction coordinates (IRC) as implemented by Morokuma and coworkers.[18]

Minimum energy crossing points (MECP), the point at which two potential energy surfaces cross, were calculated in ORCA[19] with the adapted frequency analysis "SurfCrossNumFreq" to verify the MECP is at a minimum. Energies and geometries of open-shell singlet intermediates were obtained via (1,1) broken-symmetry DFT computations, initialized from the corresponding triplet state using ORCA's "FlipSpin" function. The absence of spin contamination was verified by using Yamaguchi's spin projection method.[20] The spin densities of the open-shell singlet intermediates were inspected to ensure that they were chemically reasonable.

Born-Oppenheimer molecular dynamics were run using ORCA 5, forces acting on all atoms were calculated using DFT, at the SMD(1,2-dichloroethane) M06-2X/def2-SVP level of theory. Newtonian equations of motion were calculated with velocity Verlet integration algorithm. Initial velocities of each atom were assigned from a random velocity distribution where the total kinetic energy is consistent with the intended simulation temperature. In order to conserve the initial temperature of the canonical ensemble, the Nosé-Hoover thermostat[21] with a high-order Yoshida integrator[22] was employed, the temperature was set at 298 K and the coupling strength of the thermostat was set at 10 fs. with Each MD run was randomised using a stated seed. A step size of 0.5 fs and maximum simulation time of 1.25 ps were chosen as suitable for organic molecules.[23]

Natural bond orbitals (NBO) were obtained with the NBO programme (version 3.1)[24] through Gaussian16 (Revision C.01)[25] applying the SMD(1,2-DCE)-M06-2X/def2-TZVP level of theory. Various functionals and basis set sizes were tested to ensure concordant conclusions.

Molecule visualisations were created using CYLview software.[26]

Photochemistry of sulfonyl imines

Triplet energy

The photo physics of imines has been extensively computationally characterised in previous studies; [27,28] for our study we started by locating the triplet states of the imines. For each imine we found two triplet states. For triplet sulfamoyl imine, ^{3}A, we found a planar geometry around the C—N bond, ^{3}A1 and a twisted geometry around the C—N bond, ^{3}A2. The twisted state was found to be lower for all imines investigated bar the CN substituted sulfonyl imine. The adiabatic singlet-triplet energy gaps were calculated using the planar conformer in order to minimise structural reorganisation during triplet-triplet energy transfer as described in a previous report.[29,30]

$$E_T = G\big(T_1(planar)\big) - G(S_0)$$

$$E_T(twisted) = G\big(T_1(twisted)\big) - G(S_0)$$

Supplementary Table 10 Compiled triplet energies of sulfonyl aldimines in kcalmol^{-1}.

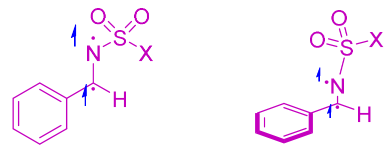

X	Cl	CF_3	CN	F	Me	Bn	Ph
E_T / kcalmol^{-1}	61.5	63.8	65.9	67.3	69.3	69.3	69.6
E_T(twisted) / kcalmol^{-1}	61.4	63.1	66.9	66.5	60.9	62.7	61.8

MECP

To give a first approximation of the lifetimes of the triplet aldimines in solution we looked at the minimum energy crossing points (MECP) relative to twisted triplet aldimine (*vide supra*) **Supplementary Table 11**, using the twisted triplet imines as the starting geometry.

Supplementary Table 11 Minimum energy crossing points (MECP) of selected aldimines in kcalmol^{-1}.

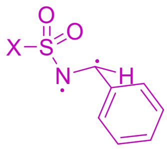

increasing MECP ⟹

X	Ph	Me	Bn	Cl	CF_3	F	CN
MECP / kcalmol^{-1}	5.9	7.3	8.8	9.9	11.5	11.9	13.2

The imidic *C*- and *N*- atoms of the triplet aldimines possess geometry consistent with atoms bearing sp^2 hybridisation, with trigonal planar geometry around the imidic *C* and bond angles close to 120°, as well as a bent geometry around *N* with a bond angle of 114.9° **Supplementary Fig. 10A**. Whereas these atoms in the MECP geometry have bond angles consistent with sp^3 hybridisation, with trigonal pyramidal geometry around the imidic C hence bond angles close to 109.5° and a bent geometry around *N* with a bond angle of 107.4° **Supplementary Fig. 10B**. Inspection of spin densities and charges show the singlet state species that is converged to is indistinguishable from shell species. Therefore, the MECP appears to represent the crossing between the lowest energy triplet state and the ground state. Generally, more electronegative groups on the sulfonyl group increase the energy of the MECP.

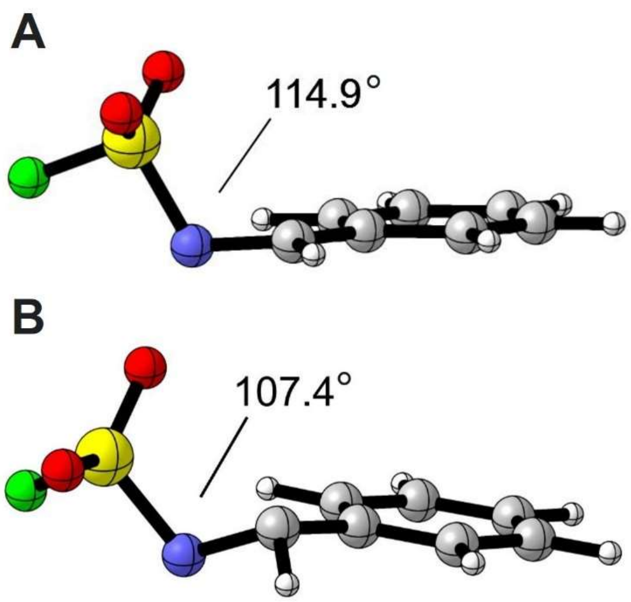

Supplementary Fig. 10 Geometries of selected aldimine species. A) Geometry of twisted triplet aldimine 3A2 with S-N-C bond angle annotated. B) MECP geomery of sulfamoyl fluoride aldimine MECP1. S-N-C bond angle annotated.

Isomerisation

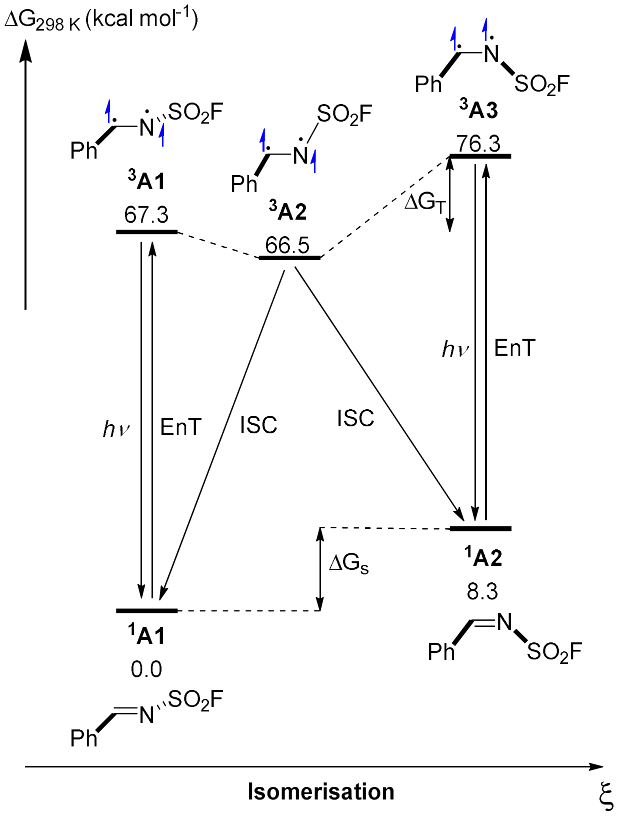

Supplementary Fig. 11 Energy profile for isomerisation.

During our experimental investigation the *Z*-aldimine was never observed, in order to establish why this was the case photochemical isomerisation was studied computationally **Supplementary Fig. 11**. The position of equilibrium between *E*- and *Z*-isomers for both ground state and excited state

aldimines were found to lie strongly towards the *E*- aldimine (ΔG_s = 8.3 kcal mol^{-1} and ΔG_T = 9.0 kcal mol^{-1} respectively). The adiabatic singlet-triplet energy gap for both of the aldimine geometries are very similar (67.3 vs 68.0 kcal mol^{-1}), given this, we may assume that the difference in the rates of energy transfer is negligible. Therefore, if we assume that rates of intersystem crossing are similar for both geometries of the triplet imine and this outcompetes phosphorescence, the distribution of geometries is only determined by the difference in Gibbs free energy of the two species in the ground state (ΔG_s).[31] This gives a value of the equilibrium constant for the reaction below to be K = 8.14×10^{-7}, or one in every 1.2 million molecules, consistent with the observation that the *Z*-imine is not detected under our conditions.

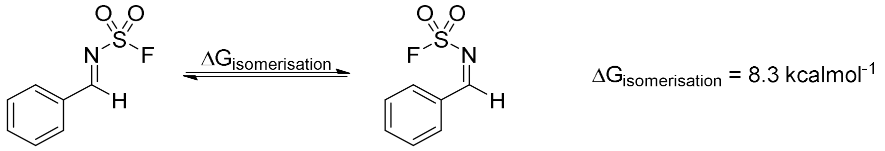

$$K = e^{\left(-\frac{\Delta G}{RT}\right)}$$

Fragmentation of triplet sulfonyl aldimines

To probe the fragmentation of the triplet sulfonyl aldimines in more detail various electronic parameters were examined for the different SO_2X units. Properties evaluated included the Hirshfield charge, Mulliken spin, dipole moment, bond dissociation energies (BDE), conceptual DFT properties,[32] NBO occupancies and energies as well as select second order perturbations in the singlet and triplet imine as well as the fragments SO_2X and X. The correlations were evaluated based on the coefficient of determination (R^2), mean absolute error (kcalmol^{-1}) and the Leave-one-out cross-validation metric (Q^2) to provide an initial insight into the variable's predictability. Out of the 190 variables evaluated the best corelation was observed with the N-S σ* orbital energy in the triplet imine **Supplementary Fig. 12**.

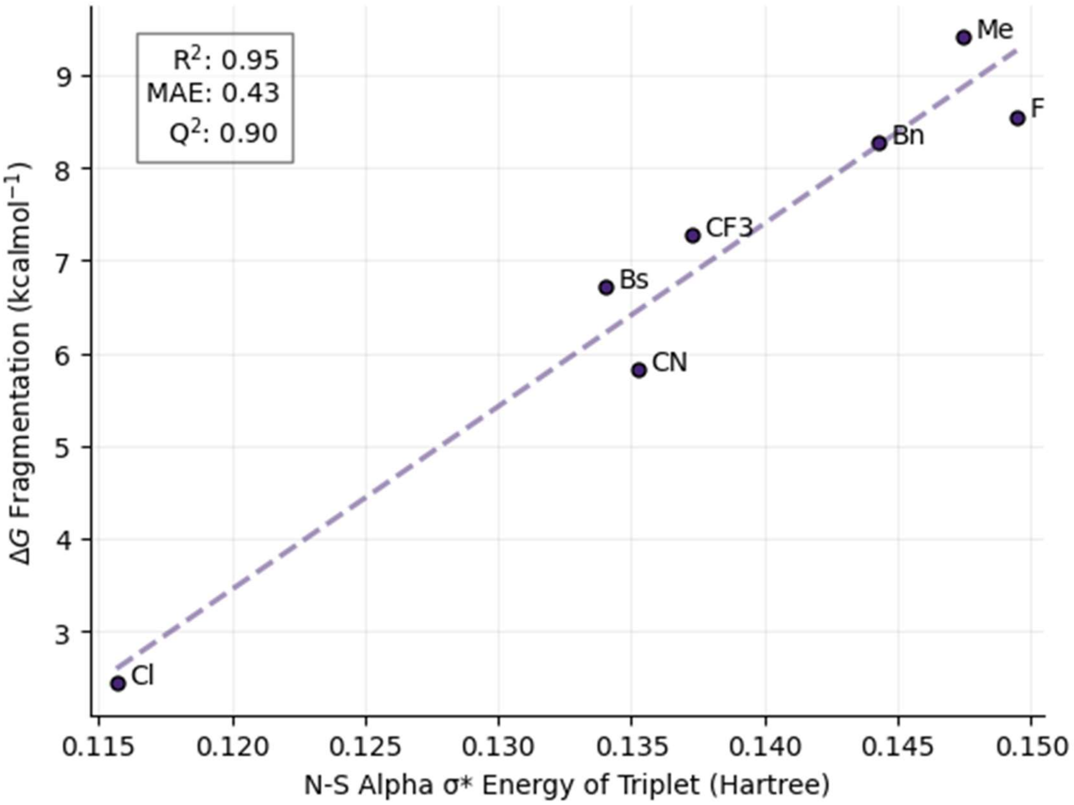

Supplementary Fig. 12 Best correlating feature with ΔG of activation for fragmentations of imines.

Addition of triplet sulfonyl aldimines into alkenes

Results and Discussion

Supplementary Table 12 Calculated Gibbs energy barrier of addition into isobutene in kcalmol^{-1}.

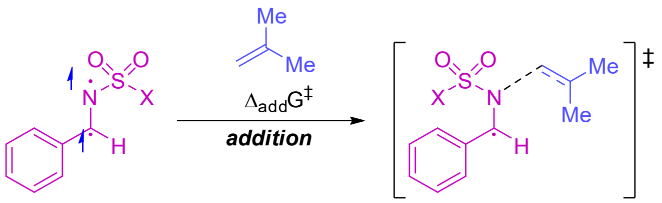

X	Me	Ph	CF_3	F	Bn	CN	Cl
$\Delta_{add}G^{\ddagger}$ / kcalmol^{-1}	14.0	12.1	-	-	10.7	-	-

- barrierless

We next looked at the corresponding activation energies for addition into isobutene for the triplet imines. While Ms-aldimine (X = CH_3) and $BnSO_2$-aldimine had a relatively large barrier to fragmentation, they had an even higher barrier to fragmentation so would be unlikely to succeed in the intermolecular [2+2] photocyclization. However, when we tried to find the barriers to addition for the imines with strong electron-withdrawing groups (-SO_2CN, -SO_2Cl -SO_2F, -SO_2CF_3) we were unable to find a transition state and instead found a series of relentlessly downhill potential energy surfaces, implying that there is either no transition state or an extremely low-lying transition state for these processes. For -SO_2Cl and -SO_2CN these were deemed unsuitable due to their low calculated fragmentation barriers and from this investigation, we concluded that the most promising candidates were the -SO_2F, and -Tf imines.

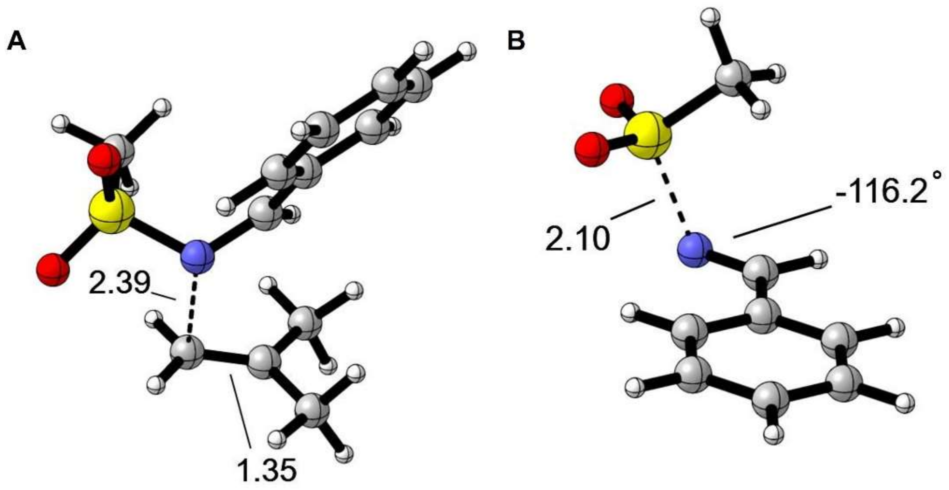

Supplementary Fig. 13 Distances in Angstroms (Å) **A** Transition state for addition of Ms-imine into isobutene. **B** Transition state for fragmentation of Ms-aldimine.

Potential energy surfaces

We examined the potential energy surface for the addition of ^{3}A into isobutene scanning from a C—N distance of 1.5 to 3.6 Å **Supplementary Fig. 14**. To ensure that our results were not specific to the level of theory used we examined a number of different variables including: the effect of basis set, solvent model, % of Hartree-Fock exchange and a range of alternative functionals. In particular, this approach helped to ensure that self-interaction error (SIE), arising from some density functionals incorrectly including an electron's interaction with its own density, and basis set superposition error (BSSE), resulting from basis functions being borrowed between interacting fragments, were minimised. Solutions were checked for the possibility of charge-transfer type mechanisms occurring and ensure the validity of the triplet solutions and given that photoinduced electron transfer between the triplet imine and isobutene is endergonic ($\Delta_{PET}G$ = 8.1 kcal mol^{-1}), an outer-sphere single electron transfer mechanism can be excluded.[33,34] We found that in almost all cases there was no maximum in the surface, which is indicative of a barrierless process.

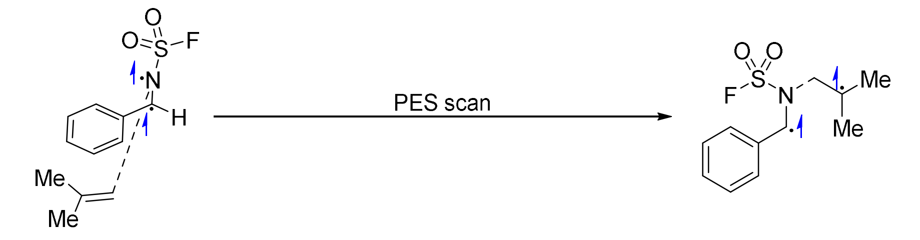

Supplementary Fig. 14 Diagramatic representation of PES scan. C-N distance indicated by dashed line.

Considering the size of the basis set, all basis sets with M06-2X produced potential energy surfaces that are entirely downhill over the range examined **Supplementary Fig. 15**. The smaller def2-SVP basis set results in the steepest potential energy surface, which is likely the result of basis set incompleteness error (BSIE). As the two fragments are moved further apart each component cannot adversely increase the size of its basis set and stabilise the adduct, therefore the energy increases. When running these geometries with the larger def2-QZVP basis set the relative energies decrease by about ~4 kcal mol^{-1} (median ΔE = 4.1 kcal mol^{-1}) corroborating that there is BSIE with def2-SVP basis set.

Increasing the size of the basis set to the triple zeta def2-TZVP leads to a less steep potential energy surface which has a discontinuity at 2.3 Å, this is as a result of a sudden flattening of the imine resulting in a lower energy conformation. Taking these geometries and evaluating them with a larger quadruple zeta basis set, def2-QZVP, the energies converge (largest ΔE = 0.14 kcal mol^{-1}) indicating that the def2-TZVP basis set is sufficiently large to model the system.

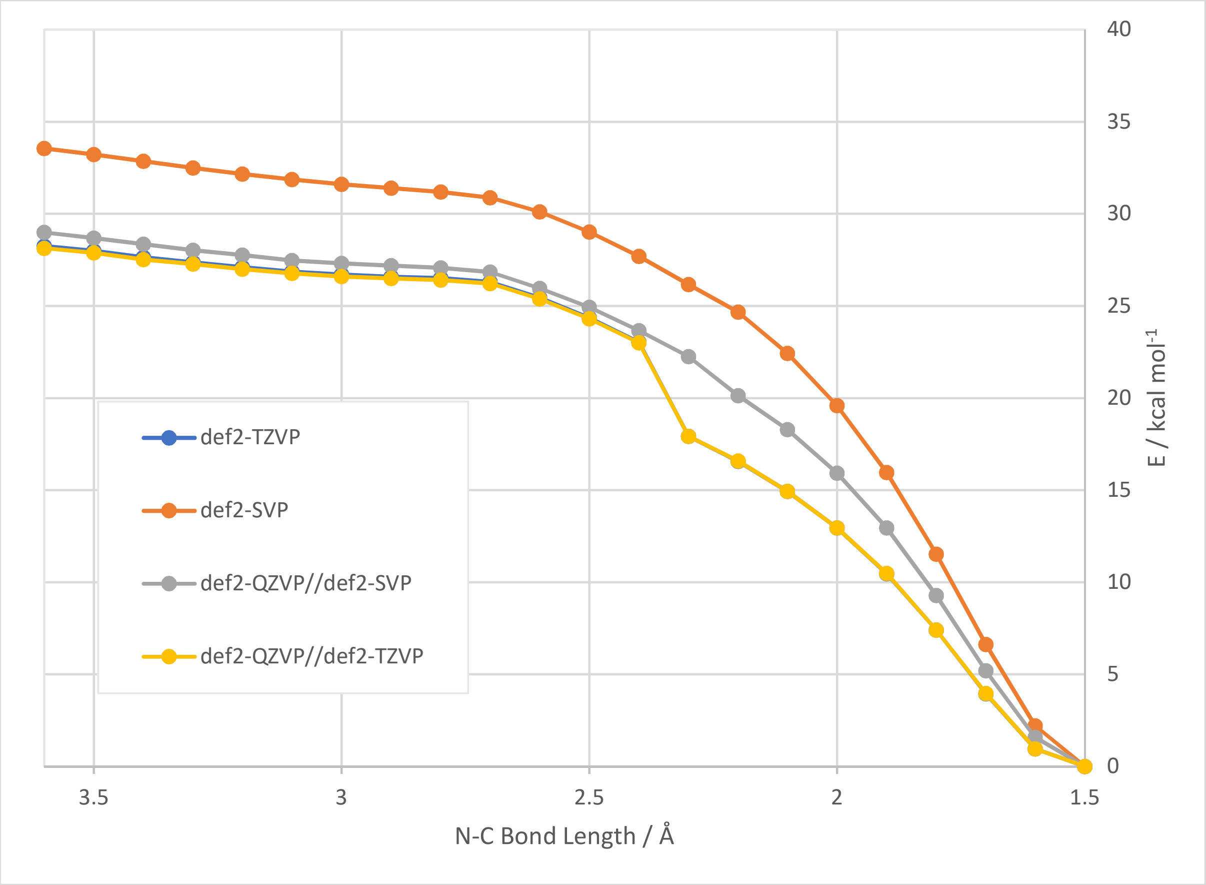

Supplementary Fig. 15 Potential energy surface scan varying the size of the basis set.

Next, the effect of the solvent model was investigated. In the solution state both CPCM and SMD solvent models do not give a point corresponding to a transition state in the potential energy surface, and have continuously downhill PES's **Supplementary Fig. 16**. At medium distances, the CPCM solvent model gives a lower energy surface than SMD but the overall shapes of the curves are very similar, showing that the choice of solvent model has little effect on the conclusions drawn.

However, in the gas phase a smooth potential energy surface results yielding a shallow maximum at 2.5 Å. Taking this geometry, it is possible to find a transition state with $\Delta^{‡}G$ = 5.4 kcal mol^{-1} and $\Delta^{‡}G$ = 9.1 kcal mol^{-1} with a larger basis set (def2-TZVP). Even though in the gas phase there is a transition state present it is still very low (below 10 kcal mol^{-1}). Given the different conclusions resulting from the absence of a solvent model, it was determined that it was important to model the solvent in order to obtain a valid conclusion for our system.

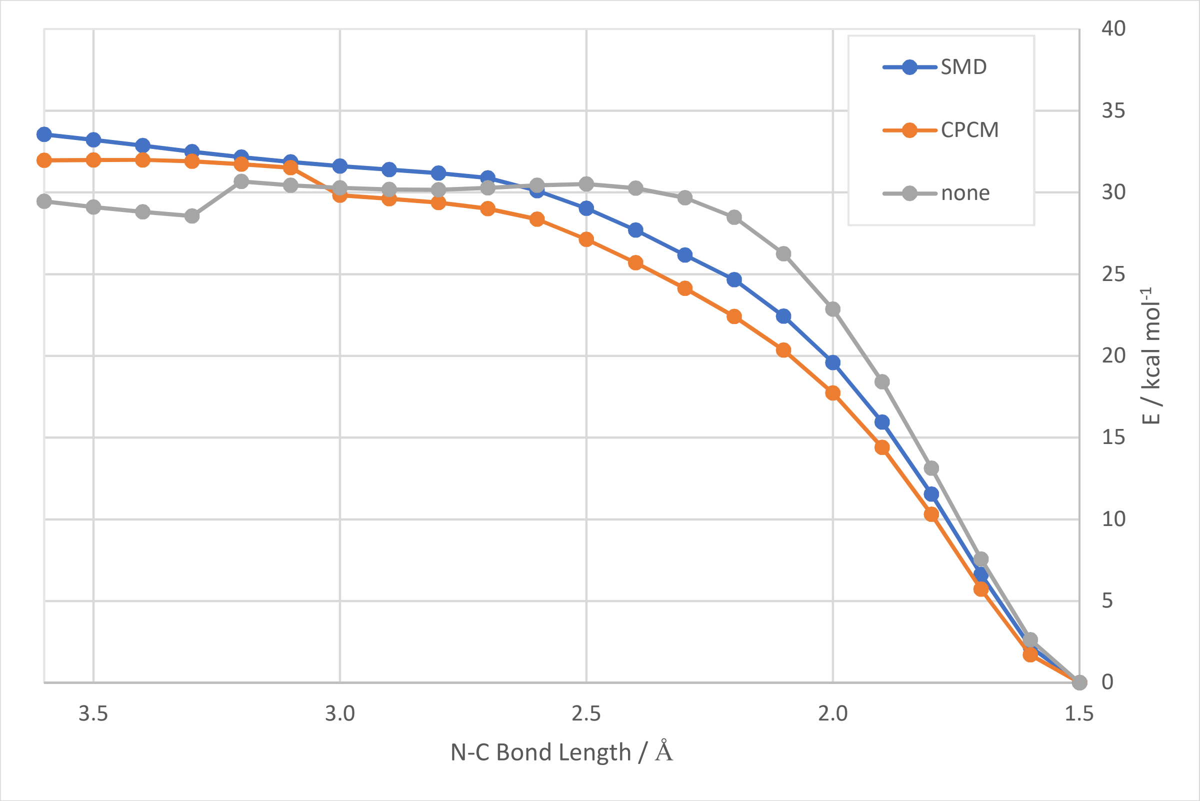

Supplementary Fig. 16 Potential energy surface scan varying the solvent model. The discontinuity in potential energy surface for the CPCM solvent model between 3.1 Å and 3.2 Å is as a result of the aldimine adding into the alkene in a different orientation.

Increasing the amount of exact exchange included in the density functional used leads to an increase in the energy of the system particularly at larger C—N distances **Supplementary Fig. 17**. This observation can be interpreted as increasing the amount of exact exchange in the density functional allows a better description of dynamic correlation in the system, hence leading to a reduction in self-interaction error and a reduction in the spurious lowering of potential energy. Increasing exchange from 54% to 100% shows no significant change in the potential energy surface from 1.5 Å to 2.3 Å and beyond 2.6 Å, implying that 54% exchange is sufficient to account for the dynamic correlation in this system. The difference in the gradient of the potential energy surface of M06-HF-D2 at distances between 2.3 Å and 2.6 Å may be a result of the D2 dispersion correction underestimating dispersion forces at this intermediate distance.

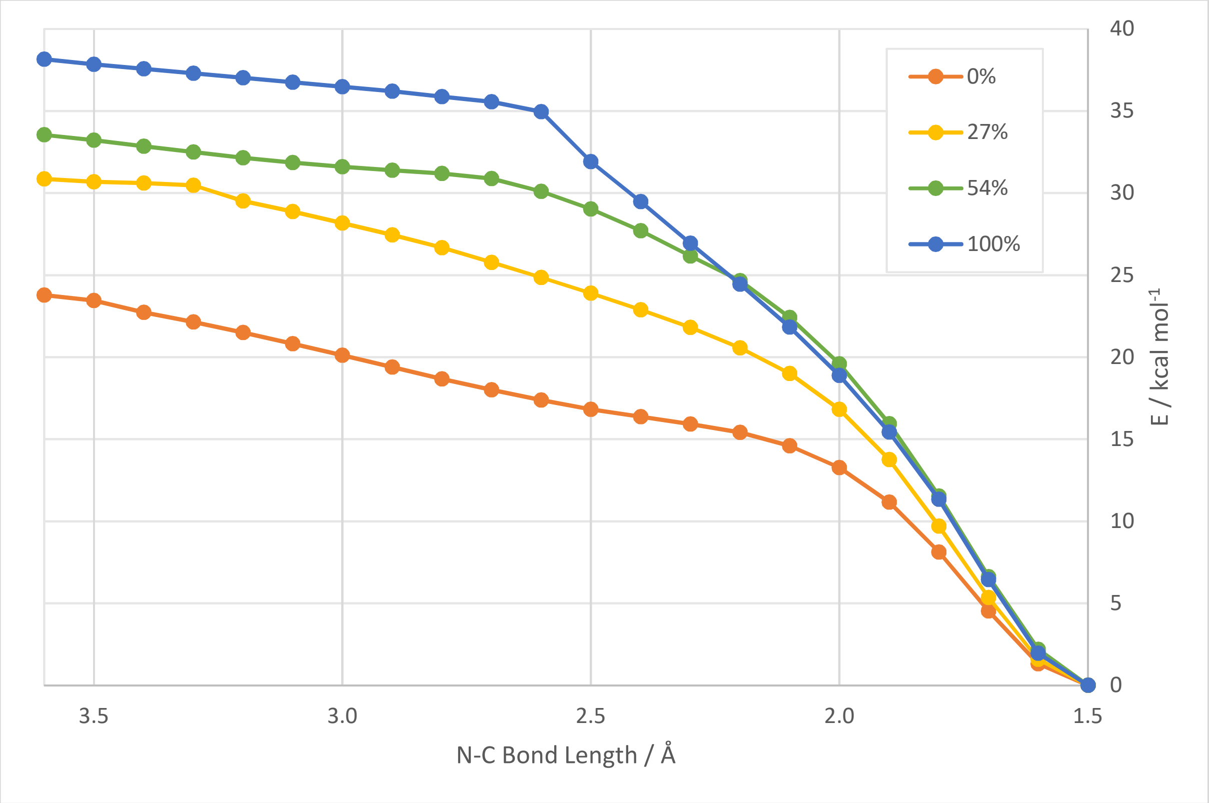

Supplementary Fig. 17 Potential energy surface scan varying % of Hartree-Fock exchange in functional. 0% is M06L-D3/def2-SVP, 27% is M06-D3/def2-SVP, 54% is M06-2X-D3/def2-SVP and 100% M06-HF-D2/def2-SVP (this functional uses the D2 dispersion correction as the D3 M06-HF-D3 counterpart is not implemented in ORCA).

Across the range of functionals tested they all produce potential energy surfaces of similar shape without any maxima in the range investigated **Supplementary Fig. 18**. Range-separated functional ωB97X-D3 and hybrid meta-GGA functional M06-2X, often two of the highest performing hybrid functionals in benchmarking studies, produced very similar PES's. Hybrid functionals M06 and in particular B3LYP produced much shallower surfaces possibly due these functionals underestimating long range interactions between the imine and alkene. M06L, a meta-GGA functional not including any Hartree-Fock exchange, performs very similarly to B3LYP at distances <2.1Å however at longer distances it produces the lowest energy potential energy surface.

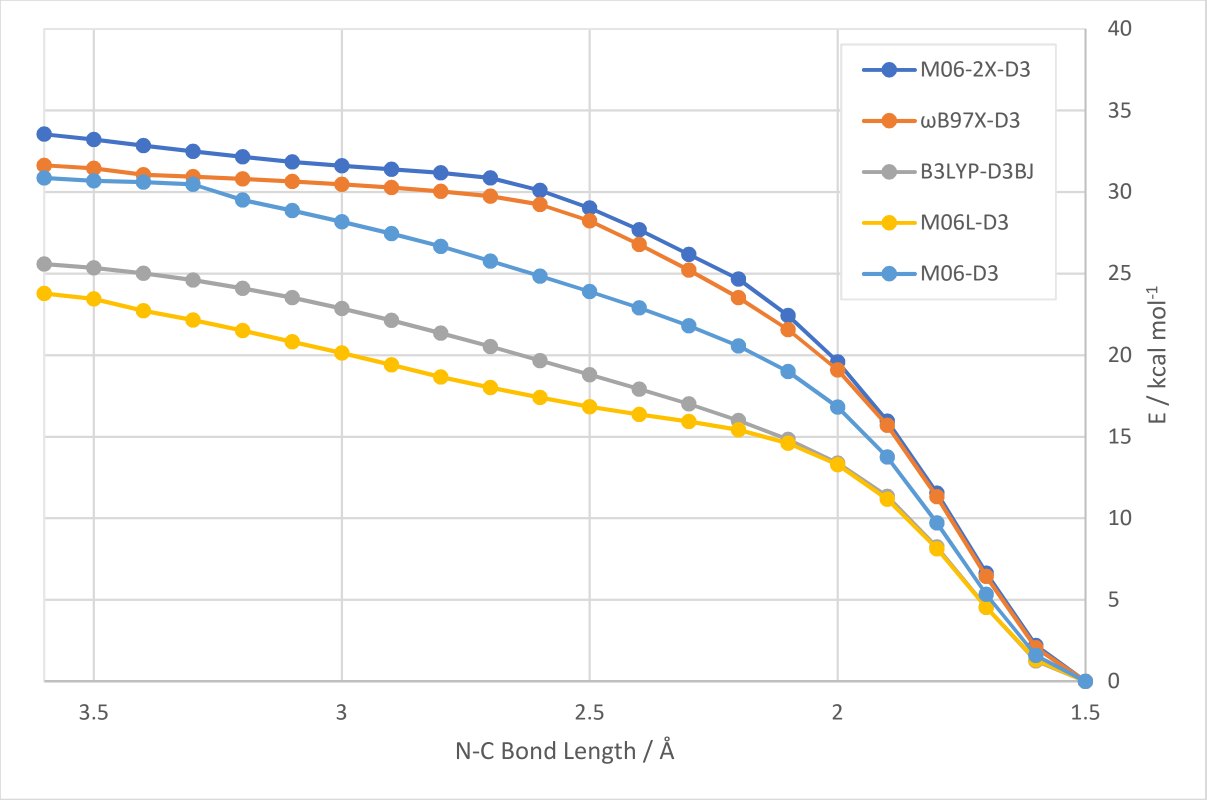

Supplementary Fig. 18 Potential energy surface scans varying the functionals.

Overall, the potential energy surfaces generated result in downhill potential energy surfaces, which indicate that this process is likely either barrierless or has an extremely small barrier. In all bar one case, we see a maximum in the potential energy surface, which occured when examining in the gas phase.

Alternative Transition States

For completeness, addition of the imine into the alkene with other orientations was considered **Supplementary Fig. 19**. For the -SO_2F aldimine a transition state was located with the gem-dimethyl portion of the alkene is eclipsing the sulfonyl group in a gauche manner (θ(S-N-C(alkene)-C(alkene)) = -62.1°) with a barrier of 3.2 kcal mol^{-1} **Supplementary Fig. 20**. By inspecting the Mulliken atomic charges and spin densities we noted that this transition state is consistent with the formation of an exciplex with significant charge transfer. Spin density is almost equally distributed between the imine and isobutene (1.09 and 0.91 respectively) accompanied by a charge transfer of +0.8 and -0.8 for the alkene and imine respectively **Supplementary Fig. 19**. Although these transition states would produce feasible processes, as discussed, PET is endergonic, which would preclude the initial electron transfer. Moreover, from the investigation of the level on theory on the PES's for addition discussed previously, some of the scans gave the gauche conformation through the triplet biradical without any barrier, suggesting that the dominant mechanism is addition the triplet imine into alkene without full charge transfer.

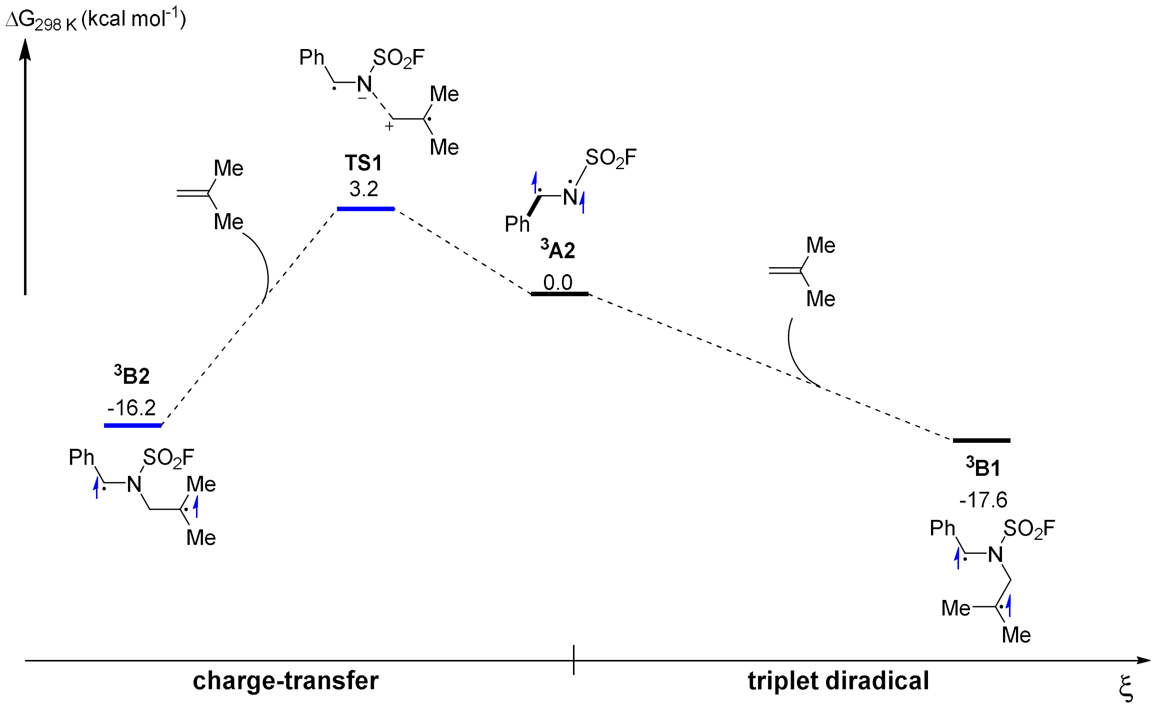

Supplementary Fig. 19 Comparison of reactivity profiles for charge transfer addition mechanism vs. triplet imine addition.

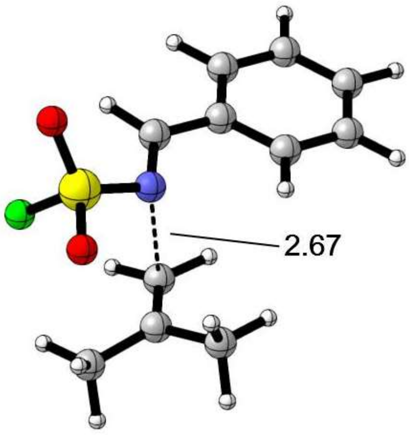

Supplementary Fig. 20 Structure of transition state (TS1) for addition into isobutene for the gauche conformation. Labelled distance in angstroms (Å).

Regioselectivity of Addition

Given the excellent regioselectivity observed experimentally for the product resulting from initial *N*-addition, the barrier to *C*- addition was investigated **Supplementary Fig. 21**. It was found that unlike addition at *N*-, there is a barrier for *C*-addition of 7.4 kcal mol^{-1}, which would preclude the formation the resulting regioisomer as it would not be kinetically competitive with addition at *N*. This computational data strongly supports the experimental observation, showing that the nucleophilic alkene reacts more rapidly at the electrophilic *N* than at the nucleophilic *C*, and that any process with a barrier greater than 7.4 kcal mol^{-1} would likewise be unable to compete, including fragmentation.

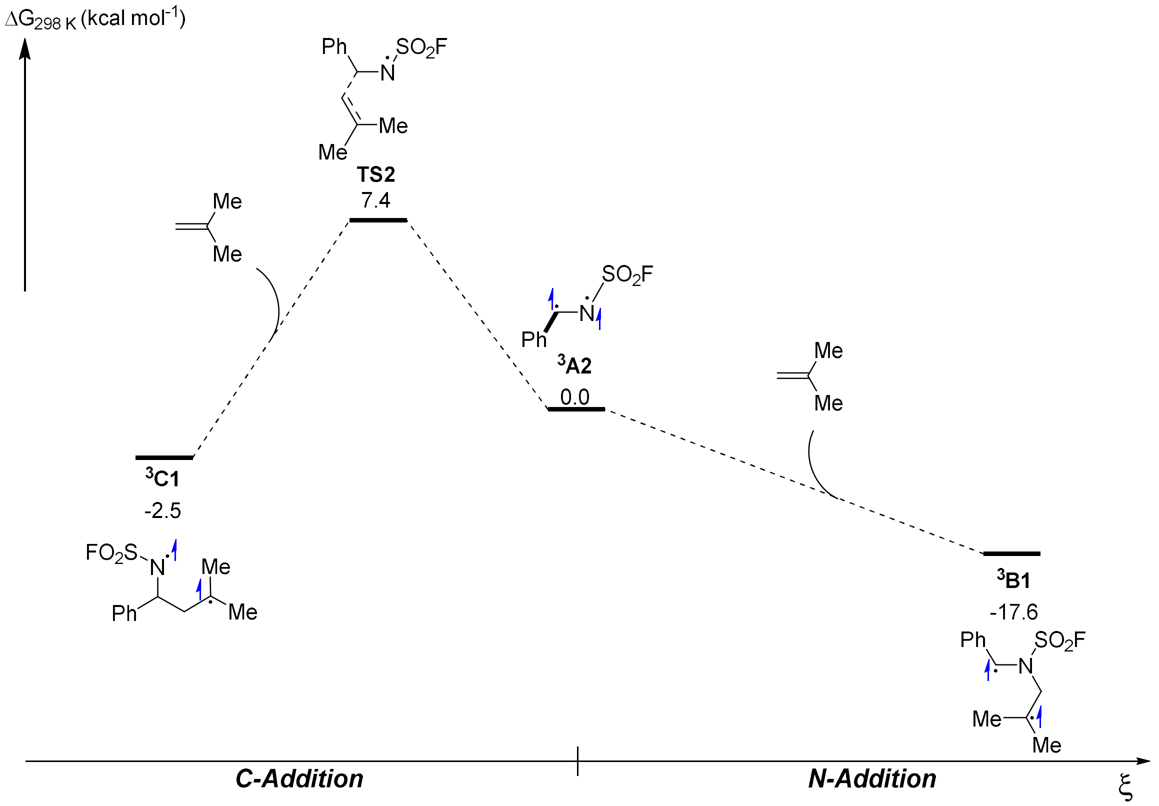

Supplementary Fig. 21 Addition of reaction profile for *N*-addition vs. *C*-addition.

In order to determine other possible reactive positions on the imine the spin densities were inspected and it was found there was significant spin density on the *ortho*- positions (0.22 and 0.24) of the arene ring. It was hypothesised that addition into this position may be feasible. An encounter complex, ^{3}D1, was located and following this endergonic encounter complex the transition state is a further 1.6 kcal mol^{-1} higher in energy for a total barrier of 4.6 kcal mol^{-1} to *o*- addition **Supplementary Fig. 22**. For our model system this precludes this regioisomer from forming relative to addition at *N*. It is worth noting that the transition state for *ortho*-addition (TS3) is actually lower in energy than transition state for addition at the imidic-*C* (TS2) ($\Delta\Delta G^{\ddagger}$ = 2.8 kcal mol^{-1}), despite the imidic-*C* possessing Mulliken spin density (0.67) higher than both *o*- carbons combined. This observation suggests that radical philicity may be a more important factor in determining reaction rates than spin density alone.

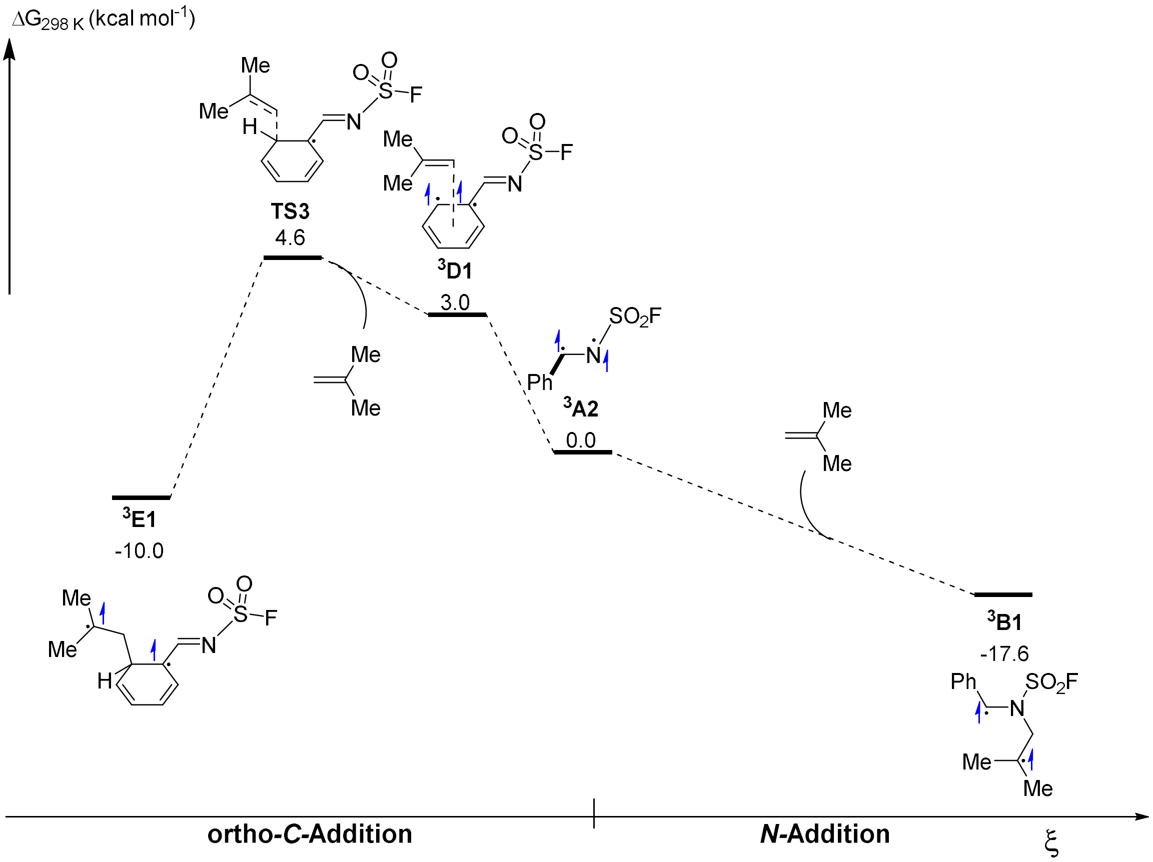

Supplementary Fig. 22 Addition of reaction profile for *N*-addition vs. *ortho C*-addition.

Intersystem crossing

Following addition of the triplet imine into isobutene we found two conformers for the triplet biradical intermediate a lower energy conformer with the isopropyl radical anti-periplanar to the -SO_2F group and a higher energy gauche conformation **Supplementary Fig. 23**. The resulting biradical from addition into isobutene has a very low-lying minimum energy crossing point (MECP) implying that intersystem crossing is very rapid. The following radical-radical recombination step, which is expected to barrierless, is also highly exergonic meaning that the addition of alkene is irreversible resulting in product formation. Two conformations of the singlet biradical were found with the same order of energy as the MECP and triplet states. Although DFT methods may result in static correlation effects, the broken symmetry solution and MECP provide an initial approximation to indicate a facile route to the product. Within the BS-DFT formulation the spin densities of the spin densities of the relevant atoms were checked to ensure a biradical species was formed and not a zwitterionic species.

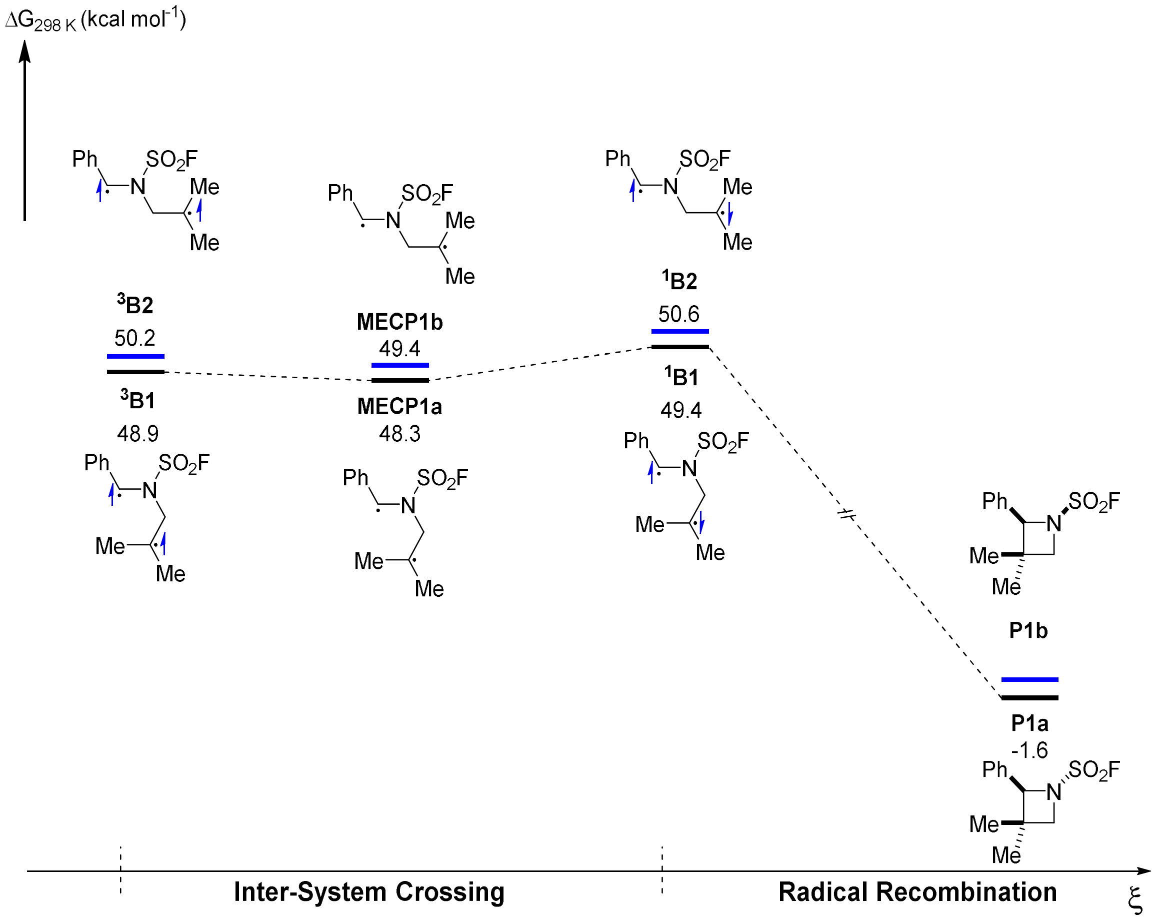

Supplementary Fig. 23 Reaction profile following the fate of the 1,4-biradical resulting from addition into isobutene.

Alternative Alkene

To rationalise why exocyclic 1,1-dialkyl alkenes required an increased loading relative to their non-exocyclic counterparts, we decided to investigate the reaction profile computationally and determine which step in the mechanism may explain this difference. Our model alkene for this study was methylene cyclobutene, as this class of alkene was shown to work experimentally and is computationally inexpensive.

Addition of Imine

Two conformations of the triplet biradical from the addition of the imine into methylene cyclobutene were investigated: a higher-energy extended conformation and a lower-energy contracted conformation **Supplementary Fig. 24**.

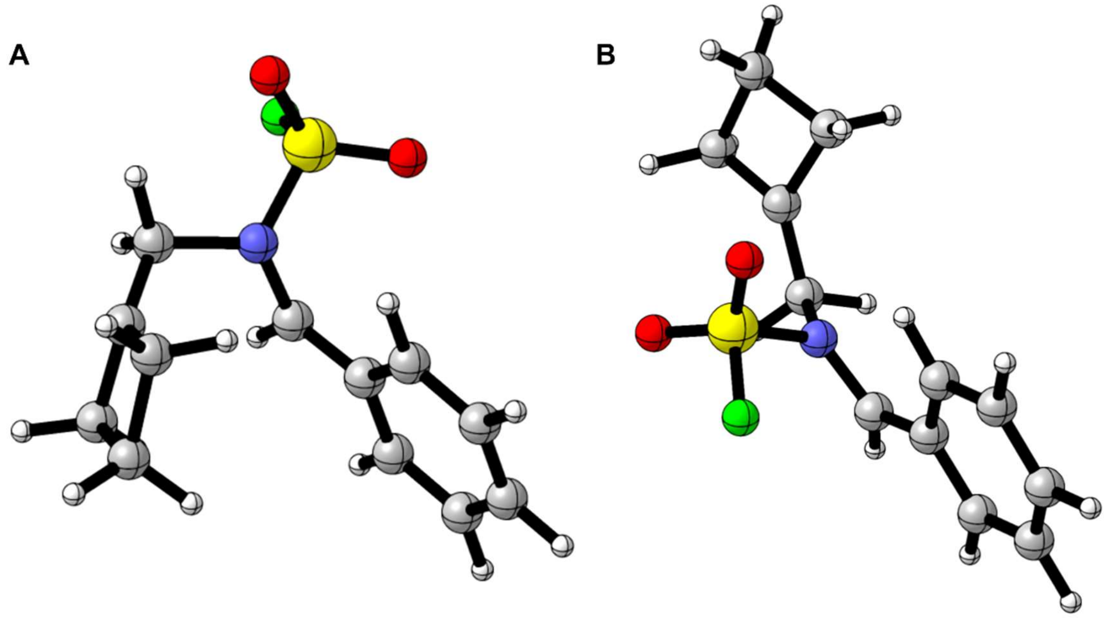

Supplementary Fig. 24A Lower energy conformation triplet biradical ^{3}E1 resulting from addition of imine into methylene cyclobutene. B Higher energy extended conformation ^{3}E2.

PES scans were calculated using SMD(DCE)-M06-2X-D3/def2-SVP, resulting in both conformations from the addition of the triplet imine into the alkene **Supplementary Fig. 25**. For the surface leading to the extended product, the energy surface was entirely downhill with no maxima from 3.6 Å onward. For the surface leading to the contracted conformation, the addition of the imine into cyclobutene resulted in a downhill surface from 3.0 Å. Beyond this distance, there is a decrease in energy due to a change in the orientation of the alkene as the imine begins to add to the more substituted carbon, resulting in an artificial maximum. Indeed, it was difficult to obtain a potential energy surface where the imine did not add to the more substituted carbon when starting from the separated imine and alkene. The 'extended' PES appears lower in energy compared to the 'contracted' surface because the extended product is higher in energy, effectively transposing the entire surface downward. During the scans, we sometimes observed surfaces where full charge transfer occurred. However, this possibility could be ruled out due to the endergonicity of PET for this system ($\Delta_{PET}G$ = 8.6 kcal mol^{-1}).

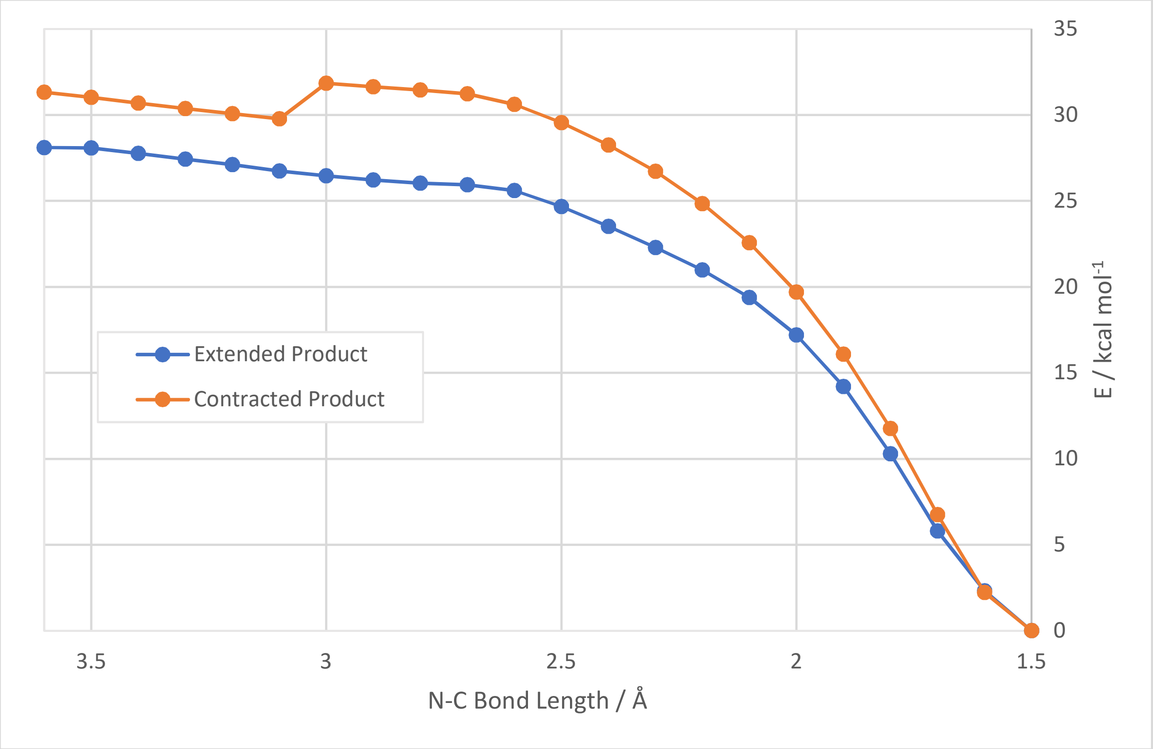

Supplementary Fig. 25 PES surface for the addition of methylene cyclobutane.

Therefore, we ruled that addition of the imine into methylene cyclobutene like isobutene is also barrierless, therefore another step in the mechanism is likely responsible for the difference in reactivity observed.

Intersystem Crossing

Following on from the barrierless formation of the triplet biradical resulting from addition into methylene cyclobutene, intersystem crossing was investigated to see if intersystem crossing was substantially different for the exocyclic alkene to the previous system **Supplementary Fig. 26**. To give a first approximation of the rate of intersystem crossing the MECP for both conformations was investigated. However, in this case we obtained similar results to the previous system with an MECP very close in energy to the initial triplet species and likely very rapid. We were able to locate the corresponding open-shell singlet biradical after MECP for the extended conformer however; it was not possible to locate a stable open-shelled singlet biradical species for the contracted species, which was unstable relative to the closed shell product.

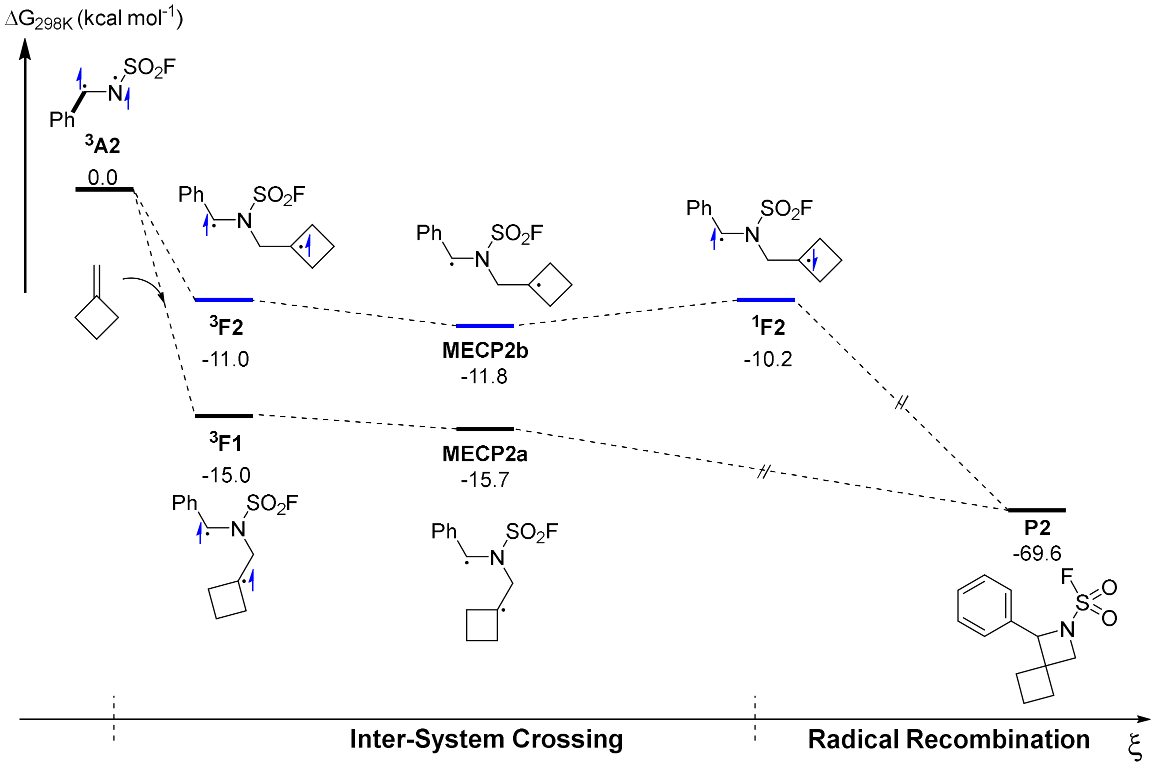

Supplementary Fig. 26 Reaction profile following the fate of the 1,4-biradical into methylene isobutene.

It is worth noting that the relative energy of the MECP to the triplet state does not necessarily reflect the rate of inter-system crossing and the dynamics of the system need to be taken into account.

Ultimately, it is not possible to conclusively state why the seemingly small structural difference of an exocyclic alkene *vs* acyclic alkene results in a large difference in reactivity. It is unlikely to be as a result of the addition step as these are very similar for both alkenes and are well described by DFT and is more likely a result of difference in intersystem crossing which even though this has an MECP that is close in energy to the parent triplet, is not well described by DFT.

Calculated reduction potentials

To further rule out the possibility of a photoredox catalysed reaction manifold operating in our system we looked at the reduction at series of aldimines varying the electronics of the arene ring **Supplementary Table 13**. This data along with calculated excited state oxidation potentials allows the determination of the feasibility of the generation of a radical anion.

A recent benchmarking study by Neese and Pantazis found that M06-2X functional in conjunction with the SMD solvent model performed comparatively with efficient local pair natural orbital (LPNO) approaches providing the smallest root-mean-square-error (RMSE) of 0.12 eV for the calculation of aqueous redox potentials of 19 small molecules.[35] Following this protocol, redox potentials were calculated as described previously (SMD(1,2-dichloroethane)-M06-2X-D3/def2-TZVP). Within this approach the reduction potential is calculated using the following equations.[36] The values of Faraday's constant and the Saturated Calomel Electrode (SCE) were 23.061 kcal/V and 4.429 V respectively.[37]

$$\Delta G^{\circ}_{red} = G_{A\cdot,\ sol} - G_{A+,\ solv}$$

$$E^{\circ}_{1/2} = -\frac{\Delta G^{\circ}_{red}}{nF}$$

Supplementary Table 13 Computed reduction potentials of sulfamoyl fluoride imines vs. SCE in DCE. Experimental value is in MeCN.

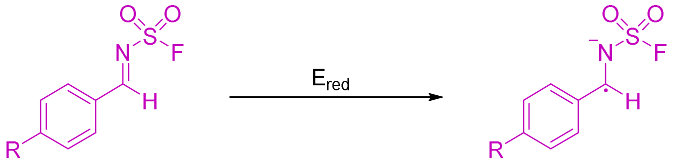

R	σ_p	Calculated E_{red} / V	Experimental E_{red} / V
H	+0.00	-1.13	-
OMe	-0.27	-1.28	-1.12
CN	+0.66	-0.77	-

The calculated reduction potential for the OMe substituted imine (E_{red} = -1.28 V) was benchmarked against the experimental reduction potential in MeCN (E_{red} = -1.12 V) and the two results show good agreement. In general, the calculated reduction potentials of sulfamoyl fluoride imines are very low, and are much lower than the experimental values for tosyl aldimines (for *N*-(4-methoxybenzylidene)-4-methylbenzenesulfonamide E_{red} = -1.44 V. [29]. From the limited dataset above adding a more electron-withdrawing group onto the imine and increasing the Hammett parameter significantly reduces the reduction potential of the aldimine. This puts the aldimine reduction potential in range of the more reducing ITX photocatalyst (*vide infra*) resulting in lower yield as a result reduction being competitive with sensitisation.

Next, we wanted to look at how the oxidising power of the same aldimines as above in the triplet state **Supplementary Table 14**. Adding electron withdrawing groups stabilises the resulting radical anion, making the aldimine more oxidising and *vice versa* for -OMe. We hypothesized that in some cases reductive quenching of the triplet aldimine with a ground state aldimine may possibly become competitive with [2+2]-photocyclisation. For these calculations the planar geometry of the imines was implemented as this allowed for the lowest structural reorganisation and therefore fastest rate of electron-transfer.

Supplementary Table 14 Computed reduction potentials of triplet sulfamoyl fluoride aldimines.

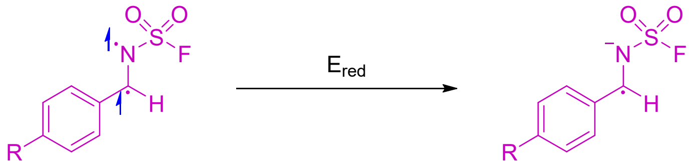

R	E_{red}
OMe	+1.38
H	+1.74
CN	+2.04

More electron-withdrawing substituents on the arene lead to more positive reduction potentials, reflecting the increased stability of the resulting radical anion.

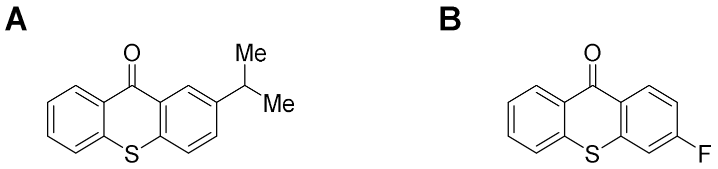

ITX | 3-F-TX

E_T(ITX) = 64.2 kcalmol^{-1}
E_{red}(ITX$^{\bullet+}$/ITX) = 1.76 V
E_{red}(ITX$^{\bullet+}$/^{3}ITX) = -1.16 V

E_T(3-FTX) = 68.3 kcalmol^{-1}
E_{red}(3-FTX$^{\bullet+}$/3-FTX) = 1.96 V
E_{red}(3-FTX$^{\bullet+}$/^{3}3-FTX) = -0.99 V

Supplementary Fig. 27 Photochemical and electrochemical properties of A ITX and B 3-FTX.

In order to gain further understanding of the photocatalysts employed in this study, the photochemical and electrochemical properties were investigated. First the triplet energies were computed using SMD(DCE)/M06-2X-D3/def2-TZVP (calculated values are E_T(ITX) = 64.2 kcal mol^{-1} and E_T(3-FTX) = 68.3 kcal mol^{-1}) which are consistent with the experimental values (E_T(ITX) = 63.6 kcal mol^{-1} and E_T(3-FTX) = 67.4 kcal mol^{-1}), previously determined by phosphorescence emission spectroscopy **Supplementary Fig. 27**.[2] Comparing the triplet energies (E_T) calculated in this study and triplet energy calculated for sulfamoyl fluoride aldimine A (E_T = 67.3 kcal mol^{-1}), energy transfer is predicted to be slightly endergonic for ITX and slightly exergonic for 3-FTX which may account for the improved performance of 3-FTX over ITX in our system for the simple phenyl aldimine.

The less negative reduction potential of 3-FTX compared to ITX should also be taken into account particularly with respect to the improved performance of 3-FTX for aldimines bearing an electron-withdrawing groups on the aromatic group, which we would expect to reduce the triplet energy of the aldimine. In the case of ITX, single electron reduction and back electron transfer of electron-deficient aldimines becomes competitive with energy transfer, which reduces the efficiency of energy transfer resulting in a diminished yield of the aza-Paternò Büchi reaction.

Supplementary Table 15 Tabulated thermodynamic quantities for photocatalyst derived species in hartrees. E – electronic energy, ZPE – vibrational zero-point energy, H – enthalpy, S – Entropy, G- gibbs free energy. T is the temperature at 298.15 K.

Species	E(el)	ZPE	H	T.S	G(1atm)	G(1M)
**ITX**	-1091.556829	0.257363	-1091.283401	-0.057009	-1091.340410	-1091.337398
**^{3}ITX**	-1091.449412	0.253632	-1091.179288	-0.058751	-1091.238039	-1091.235027
**ITX$^{\cdot+}$**	-1091.329347	0.257094	-1091.056007	-0.058000	-1091.114008	-1091.110996
**3-FTX**	-1072.880986	0.164437	-1072.703945	-0.050217	-1072.754162	-1072.751150
**^{3}FTX**	-1072.767462	0.160923	-1072.593610	-0.051741	-1072.645351	-1072.642339
**3-FTX$^{\cdot+}$**	-1072.645148	0.164261	-1072.468229	-0.050827	-1072.519056	-1072.516044

Born-Oppenheimer Molecular Dynamics

To further investigate the addition of the triplet sulfamoyl fluoride to the alkene and to show that there was no barrier to addition we performed molecular dynamics simulations using ORCA.

The starting geometry for the simulation was chosen from a position along the M06-2X-D3/def2-SVP potential energy surface (3.2 Å C—N bond distance). This distance was chosen as it is greater than twice the equilibrium bond length of the C—N bond and is much greater than the C—N bond distance in the solventless transition state with M06-2X-D3/def2-SVP level of theory (2.51 Å).

In our study, we performed eight simulation runs, and the imine added to the alkene in five out of these eight runs as can be seen from a plot of C — N distance against time **Supplementary Fig. 28**. When addition occurred, it took place within the first 400 fs of the simulation in runs 2, 3, 7, and 8. However, in run 4, additions only occurred after 1000 fs. When the imine and the alkene did not combine (runs 1,5 and 6), the distance between the two species may initially decrease however the distance between the species then increases beyond their initial separated distance, this is a consequence of the changed random initial velocities.

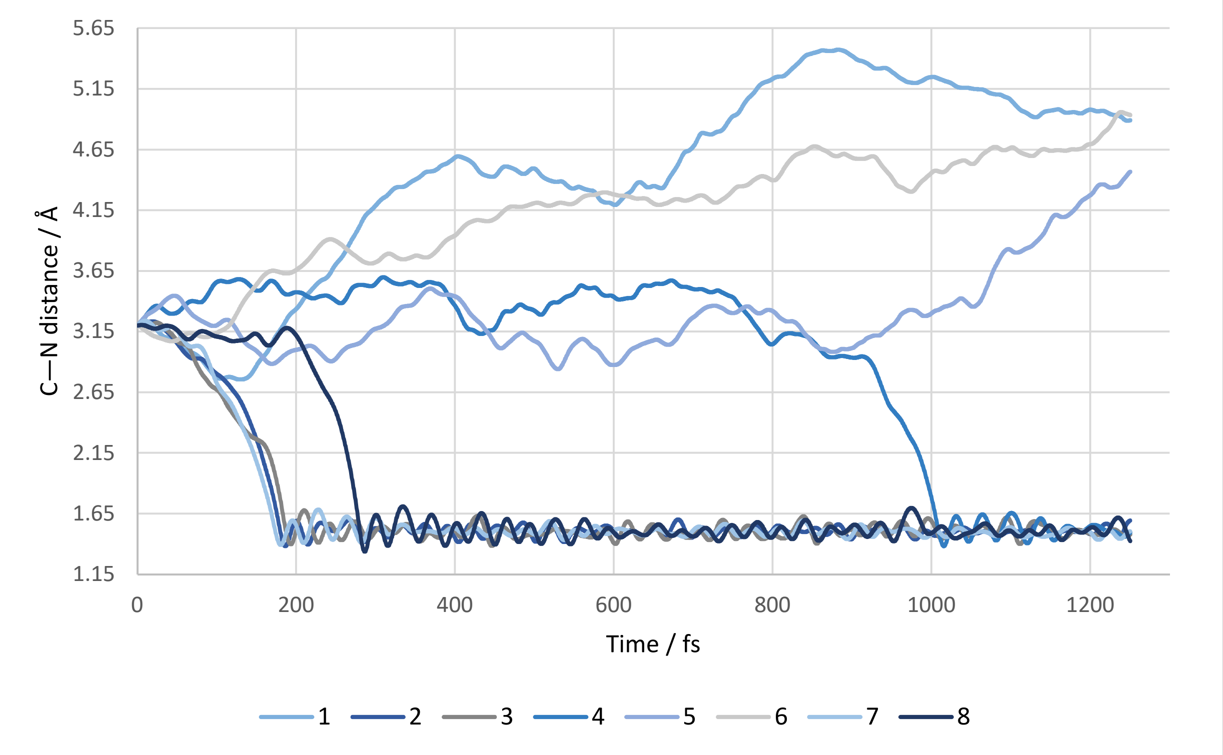

Supplementary Fig. 28 Plot of C—N bond distance over time for molecular dynamics simulations. Numbers indicate the random seed.

Upon addition of the imine into the alkene into the imine a corresponding increase in the equilibrium bond length of the C – C double bond of alkene is observed **Supplementary Fig. 29**.

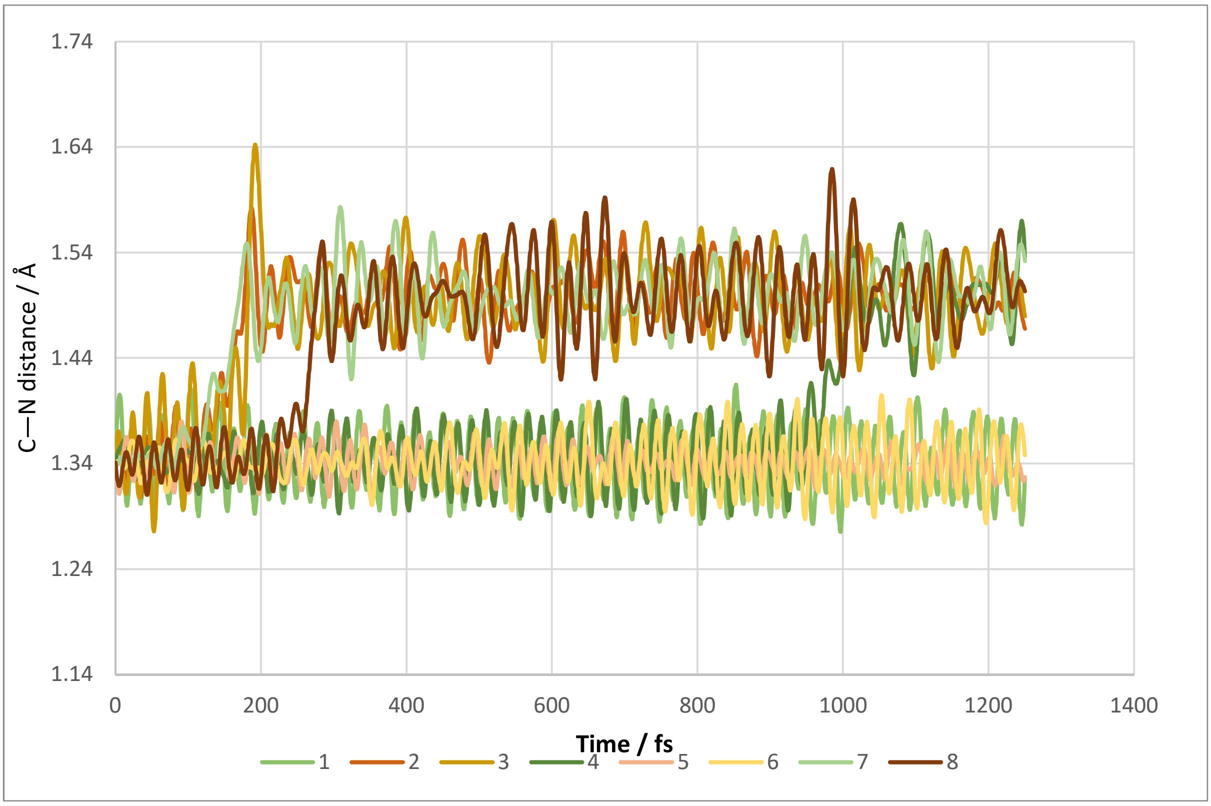

Supplementary Fig. 29 Plot of C—C bond distance over time for molecular dynamics simulations. Numbers indicate the random seed.

Addition of the imine and olefin coincides with a sharp decrease of the potential energy of the system, indicating the formation of a bond **Supplementary Fig. 30**. Once the two species combined, the adduct remained intact for the duration of the simulation, implying that addition of the imine into the alkene is irreversible, indicating there is no recrossing effect.

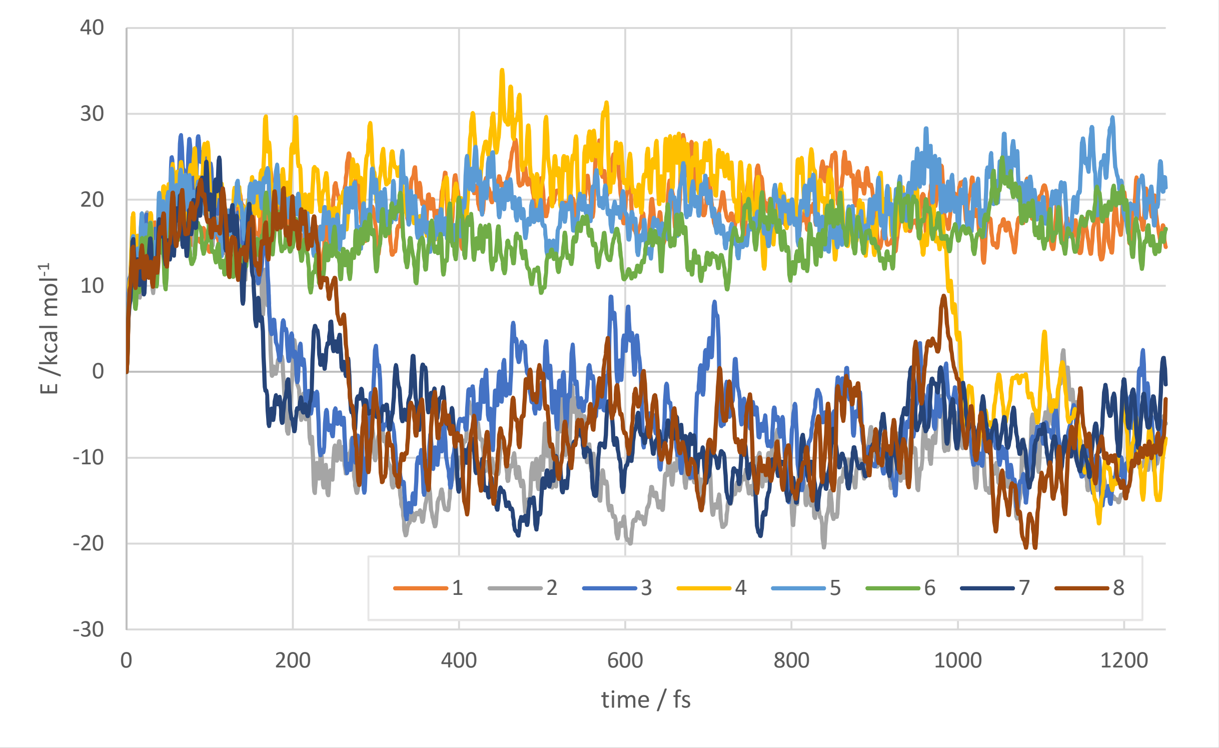

Supplementary Fig. 30 Potential energy surfaces for MD runs.

Addition of the alkene into the imine on the femtosecond timescale over multiple runs is strong evidence that there is no barrier to addition up to a distance of 3.2 Å and that this addition is incredibly fast. Hence the only barrier for the addition is likely the diffusion of the two species and the formation of an encounter complex. In this study, only one conformation of the imine and alkene was investigated.

Table of thermodynamic properties

Tabulated thermodynamic quantities in hartrees. E – electronic energy, ZPE – vibrational zero-point energy, H – enthalpy, S – Entropy, G- gibbs free energy. T is the temperature at 298.15 K. Label in brackets refers to compound label in main text.

Supplementary Table 16 Thermodynamic quantities for photochemistry of sulfonyl aldimine.

Label	E(el)	ZPE	H	T.S	G(1 atm)	G(1M)
$^1PhSO_2Cl$	-1333.924254	0.125276	-1333.787545	-0.048086	-1333.835630	-1333.832618
$^3PhSO_2Cl$	-1333.813362	0.121079	-1333.680086	-0.050624	-1333.730710	-1333.727698
$^3PhSO_2Cl$-twist	-1333.813898	0.121202	-1333.680772	-0.050103	-1333.730875	-1333.727863
$^1PhSO_2CF_3$	-1211.382208	0.139136	-1211.229065	-0.053520	-1211.282584	-1211.279572
$^3PhSO_2CF_3$	-1211.274951	0.135057	-1211.125298	-0.055594	-1211.180892	-1211.177880
$^3PhSO_2CF_3$-twist	-1211.276901	0.135354	-1211.127274	-0.054728	-1211.182002	-1211.178990
$^1PhSO_2CN$	-966.534266	0.132789	-966.389306	-0.049383	-966.438689	-966.435677
$^3PhSO_2CN$	-966.423184	0.128569	-966.281688	-0.051943	-966.333631	-966.330619
$^3PhSO_2CN$-twist	-966.422074	0.128696	-966.280695	-0.051448	-966.332143	-966.329131
$^1PhSO_2F$	-973.585840	0.126728	-973.448008	-0.047859	-973.495868	-973.492856

$^{3}PhSO_2F$	-973.473695	0.122684	-973.339307	-0.049384	-973.388691	-973.385679
$^{3}PhSO_2F$-twist	-973.476056	0.123148	-973.341584	-0.048392	-973.389976	-973.386964
$^{1}PhSO_2Me$	-913.633782	0.162094	-913.459875	-0.048293	-913.508168	-913.505156
$^{3}PhSO_2Me$	-913.517718	0.157901	-913.347479	-0.050244	-913.397723	-913.394711
$^{3}PhSO_2Me$-twist	-913.531698	0.158265	-913.361262	-0.049940	-913.411202	-913.408190
$^{1}PhSO_2Bn$	-1144.683442	0.244348	-1144.422892	-0.058115	-1144.481007	-1144.477995
$^{3}PhSO_2Bn$	-1144.566796	0.239900	-1144.309982	-0.060535	-1144.370518	-1144.367506
$^{3}PhSO_2Bn$-twist	-1144.562884	0.238697	-1144.321433	-0.059627	-1144.381061	-1144.378049
$^{1}PhSO_2Ph$	-1105.365573	0.215605	-1105.134975	-0.055540	-1105.190516	-1105.187504
$^{3}PhSO_2Ph$	-1105.249258	0.211462	-1105.022240	-0.057418	-1105.079657	-1105.076645
$^{3}PhSO_2Ph$-twist	-1105.263722	0.212506	-1105.036248	-0.055728	-1105.091975	-1105.088963

Supplementary Table 17 Thermodynamic quantities for imine MECPs.

Label	E(el)	ZPE	H	T.S	G(1 atm)	G(1M)
MECP-$PhSO_2F$	-973.456493	0.12147	-973.324797	-0.04618	-973.370977	-973.367965
MECP-$PhSO_2Me$	-913.519408	0.15626	-913.352103	-0.0474	-913.399507	-913.396495
MECP-$PhSO_2Bn$	-1144.56331	0.23895	-1144.30807	-0.05899	-1144.36706	-1144.36405
MECP-$PhSO_2Ph$	-1105.25244	0.21048	-1105.02712	-0.05551	-1105.08263	-1105.07962
MECP-$PhSO_2Cl$	-1333.79669	0.11954	-1333.66547	-0.04959	-1333.71506	-1333.71205
MECP-$PhSO_2CF_3$	-1211.25716	0.13378	-1211.10931	-0.05441	-1211.16372	-1211.16071
MECP-$PhSO_2CN$	-966.401085	0.12723	-966.261472	-0.05088	-966.312352	-966.30934

Supplementary Table 18 Thermodynamic quantities for imine photoisomerisation.

Label	E(el)	ZPE	H	T.S	G(1 atm)	G(1M)
^{1}A1 (^{1}A)	-973.585840	0.126728	-973.44801	-0.047859	-973.495868	-973.492856
^{1}A2	-973.574180	0.127139	-973.43619	-0.046457	-973.482650	-973.479638
^{3}A1 (^{3}A)	-973.473695	0.122684	-973.33931	-0.049384	-973.388691	-973.385679
^{3}A2 (^{3}A’)	-973.476056	0.123148	-973.34158	-0.048392	-973.389976	-973.386964
^{3}A3	-973.459909	0.122761	-973.32557	-0.048801	-973.374370	-973.371358

Supplementary Table 19 Thermodynamic quantities for imine N-S fragmentation.

Label	E(el)	ZPE	H	T.S	G(1 atm)	G(1M)
TS-frag-$PhSO_2F$ (TS1)	-973.460615	0.121028	-973.328856	-0.047491	-973.376347	-973.373335
TS-frag-$PhSO_2Me$	-913.514728	0.156556	-913.345887	-0.050312	-913.396199	-913.393187
TS-frag-$PhSO_2Bn$	-1144.562884	0.238697	-1144.307400	-0.060476	-1144.367875	-1144.364863
TS-frag-$PhSO_2Ph$	-1105.250253	0.210357	-1105.024628	-0.056641	-1105.081269	-1105.078257
TS-frag-$PhSO_2Cl$	-1333.807900	0.119706	-1333.676168	-0.050807	-1333.726975	-1333.723963

TS-frag-$PhSO_2CF_3$	-1211.170407	0.133300	-1211.116393	-0.054014	-1211.170407	-1211.167395
TS-frag-$PhSO_2CN$	-966.410588	0.126964	-966.270777	-0.052086	-966.322863	-966.319851
Imidyl radical (^{2}D)	-325.030368	0.109467	-324.913551	-0.038510	-324.952061	-324.949049
$^{\cdot}SO_2F$	-648.434352	0.010743	-648.418964	-0.031947	-648.450911	-648.447899

Supplementary Table 20 Transition states for addition of sulfonyl aldimines into isobutene.

Label	E(el)	ZPE	H	T.S	G(1 atm)	G(1M)
TS-addition-$PhSO_2Me$	-1070.734816	0.268628	-1070.448326	-0.061590	-1070.509916	-1070.506904
TS-addition-$PhSO_2Bn$	-1301.782152	0.350076	-1301.409612	-0.071422	-1301.481034	-1301.478022
TS-addition-$PhSO_2Ph$	-1262.467972	0.321414	-1262.125362	-0.068431	-1262.193793	-1262.190781

Supplementary Table 21 Thermodynamic quantities for intermediates in the aza Paterno Buchi reaction of SO_2F imine.

Label	E(el)	ZPE	H	T.S	G(1 atm)	G(1M)
^{3}B1 (^{3}B)	-1130.727733	0.235364	-1130.474940	-0.061088	-1130.536028	-1130.533016
^{3}B2	-1130.725981	0.235455	-1130.473235	-0.060592	-1130.533827	-1130.530815
^{1}B1(^{1}B)	-1130.728024	0.235653	-1130.475059	-0.060077	-1130.535136	-1130.532124
^{1}B2	-1130.726106	0.235385	-1130.473377	-0.059925	-1130.533302	-1130.530290
P1a (^{1}C)	-1130.817044	0.241192	-1130.560103	-0.056261	-1130.616364	-1130.613352
P1b	-1130.798711	0.241722	-1130.541475	-0.055574	-1130.597049	-1130.594037
^{3}C1	-1130.704660	0.235906	-1130.451567	-0.060485	-1130.512052	-1130.509040
^{3}D1	-1130.692058	0.232426	-1130.441681	-0.061867	-1130.503548	-1130.500536
^{3}E1	-1130.715876	0.235294	-1130.463363	-0.060663	-1130.524026	-1130.521014
isobutene	-157.201650	0.107929	-157.087467	-0.033559	-157.121027	-157.118015
isobutene$^{\cdot+}$	-195.027508	0.112420	-194.908402	-0.035634	-194.944036	-194.941024
TS1	-1130.690912	0.231750	-1130.441559	-0.061402	-1130.502961	-1130.499949
TS2	-1130.687536	0.234473	-1130.436103	-0.060120	-1130.496223	-1130.493211
TS3	-1130.688784	0.231739	-1130.439530	-0.061607	-1130.501137	-1130.498125
MECP1a (MECP)	-1130.727461	0.233715	-1130.476724	-0.060206	-1130.536930	-1130.533918
MECP1b	-1130.725834	0.233543	-1130.475294	-0.059947	-1130.535241	-1130.532229

Supplementary Table 22 Thermodynamic qunatities for intermediates in the aza Paterno Buchi reaction of SO_2F imine and exocyclic alkene.

Label	E(el)	ZPE	H	T.S	G(1 atm)	G(1M)
^{3}F1	-1168.798467	0.244808	-1168.537922	-0.057674	-1168.595595	-1168.592583
^{3}F2	-1168.788037	0.242663	-1168.528161	-0.061004	-1168.589165	-1168.586153
^{1}F2	-1168.787725	0.242641	-1168.527843	-0.059978	-1168.587821	-1168.584809
methylene cyclobutene	-195.272217	0.115259	-195.150966	-0.033707	-195.184673	-195.181661

methylene cyclobutene$^{\cdot+}$	-195.027508	0.112420	-194.908402	-0.035634	-194.944036	-194.941024
P2	-1168.889508	0.248320	-1168.625408	-0.057109	-1168.682517	-1168.679505
MECP2a	-1168.798301	0.243129	-1168.539808	-0.056875	-1168.596683	-1168.593671
MECP2b	-1168.787840	0.240757	-1168.530150	-0.060331	-1168.590480	-1168.587469

Supplementary Table 23 Thermodynamic quantities of imine radical anions and SO_2F imines with varying substituents on phenyl ring.

Label	E(el)	ZPE	H	T.S	G(1 atm)	G(1M)
4-OMe-PhSO$_2$F$^{\cdot-}$	-1088.229276	0.156824	-1088.058761	-0.053110	-1088.111871	-1088.108859
4-CN-PhSO$_2$F$^{\cdot-}$	-1065.835332	0.125555	-1065.696962	-0.050990	-1065.747952	-1065.744940
PhSO$_2$F$^{\cdot-}$	-973.704378	0.124031	-973.569221	-0.047752	-973.616973	-973.613961
^{1}4-OMe-PhSO$_2$F	-1088.117713	0.159985	-1087.944246	-0.052179	-1087.996424	-1087.993412
^{3}4-OMe-PhSO$_2$F	-1088.014992	0.156965	-1087.843986	-0.054427	-1087.898413	-1087.895401
^{3}4-OMe-PhSO$_2$F-twist	-1088.004738	0.155990	-1087.834872	-0.053799	-1087.888671	-1087.885659
^{1}4-CN-PhSO$_2$F	-1065.835332	0.125555	-1065.696962	-0.050990	-1065.747952	-1065.744940
^{3}4-CN-PhSO$_2$F	-1065.835332	0.122082	-1065.594931	-0.052377	-1065.647308	-1065.644296
^{3}4-CN-PhSO$_2$F-twist	-1065.726737	0.121879	-1065.591516	-0.053040	-1065.644557	-1065.641545

Energy-transfer mediated intermolecular aza-Paternò-Büchi reaction (GP1)

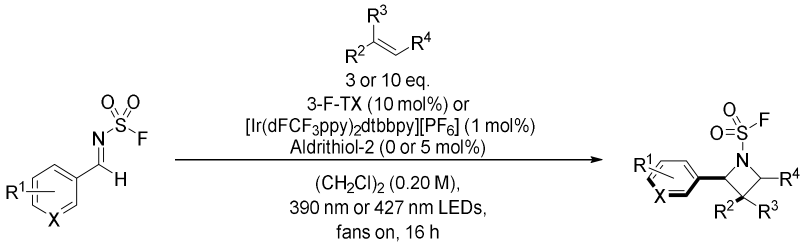

Fluorosulfamoyl imine (0.20 mmol, 1.0 eq.), 3-F-TX (4.6 mg, 0.020 mmol, 10 mol%), ITX (5.1 mg, 0.020 mmol, 10 mol%) or [Ir(dFCF$_3$ppy)$_2$(dtbbpy)][PF$_6$] (2.2 mg, 0.002 mmol, 0.01 eq.), Aldrithiol-2 (0 or 2.2 mg, 0.00 or 0.01 mmol, 0 or 5 mol%) and (if solid) alkene (0.60 –2.0 mmol, 3.0 – 10 eq.) were added to an oven-dried tapered microwave vial equipped with a stirrer bar and a septum. The vial was evacuated and backfilled with nitrogen 3 times, followed by addition of anhydrous $(CH_2Cl)_2$ (1.0 – 3.0 ml, 0.067 – 0.20 M) and (if liquid) alkene (0.60 –2.0 mmol, 3.0 – 10 eq.). The reaction was then stirred under irradiation with 390 nm LED (3-F-TX and ITX) or 427 nm LED [Ir(dFCF$_3$ppy)$_2$(dtbbpy)][PF$_6$] () at ambient temperature for 16 h, with the fans on. The reaction mixture was diluted with CH_2Cl_2, and the solvent removed under reduced pressure, followed by purification by flash column chromatography.

Note: For particular azetidines obtained using General Procedure 1, flash column chromatography afforded the product as an inseparable mixture of the photocatalyst or photocatalyst-derived impurity. These by-products can be removed via straightforward process detailed below:

Workup 1: Following chromatography, the impure azetidine was redissolved in CH_2Cl_2 (1 ml) *m*-CPBA (1 eq.) was added. Reaction mixture was stirred at rt for 16 h. Following completion, the reaction mixture was quenched with aq. sat. $NaHSO_3$ and layers partitioned. The aqueous layer was extracted with CH_2Cl_2 three times, combined organic layers were washed sequentially with aq. sat. $NaHCO_3$,

brine, dried over anhydrous $MgSO_4$ subsequently filtered and concentrated under reduced pressure. The residue was then purified by flash column chromatography

Azetidine characterisation

3,3-Diethyl-2-(4-methoxyphenyl)azetidine-1-sulfonyl fluoride (3a)

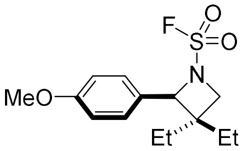

Prepared according to **GP1** using (4-methoxybenzylidene)sulfamoyl fluoride (**1**) (43.4 mg, 0.20 mmol, 1.0 eq.), ITX (5.1 mg, 0.020 mmol, 0.10 eq.) and 2-ethyl-1-butene (241 µl, 2.0 mmol, 10 eq.) in $(CH_2Cl)_2$ (1.0 ml, 0.20 M). Purification by flash column chromatography (2% Et_2O in pentane) afforded 3,3-diethyl-2-(4-methoxyphenyl)azetidine-1-sulfonyl fluoride (**3a**) as a yellow oil (55.4 mg, 91%).

R_f (10% diethyl ether in pentane) = 0.33.

^{1}H NMR (600 MHz, $CDCl_3$) δ 7.29 – 7.26 (m, 2H), 6.93 – 6.87 (m, 2H), 5.17 (d, *J* = 2.0 Hz, 1H), 3.84 (dd, *J* = 7.9, 1.1 Hz, 1H), 3.81 (s, 3H), 3.72 (dd, *J* = 8.0, 1.7 Hz, 1H), 1.80 (dq, *J* = 14.9, 7.5 Hz, 1H), 1.75 (dq, *J* = 14.6, 7.5 Hz, 1H), 1.39 (dq, *J* = 14.9, 7.4 Hz, 1H), 1.16 (dq, *J* = 14.7, 7.4 Hz, 1H), 0.94 (t, *J* = 7.5 Hz, 3H), 0.58 (t, *J* = 7.4 Hz, 3H).

^{13}C NMR (151 MHz, $CDCl_3$) δ 159.7, 128.2, 127.4, 113.9, 75.3, 60.0, 55.4, 43.7, 29.2, 24.3, 8.1, 7.3.

^{19}F NMR (565 MHz, $CDCl_3$) δ 31.29 (s).

HRMS (ESI) *m/z* $C_{14}H_{20}FNO_3SNa^+$ requires 324.1040 ($[M+Na]^+$), found 324.1046.

IR (thin film, ν_{max} /cm^{-1}) 2971, 2940, 1749, 1615, 1516, 1423, 1305, 1254, 1215, 1178, 1035, 837, 767, 625.

3,3-Diethyl-2-(2-methoxyphenyl)azetidine-1-sulfonyl fluoride (3b)

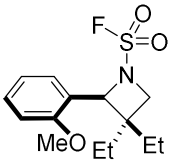

Prepared according to **GP1** using (2-methoxybenzylidene)sulfamoyl fluoride (**I-2**) (43.4 mg, 0.20 mmol, 1.0 eq.), ITX (5.1 mg, 0.020 mmol, 0.10 eq.) and 2-ethyl-1-butene (241 µl, 2.0 mmol, 10 eq.) in $(CH_2Cl)_2$ (1.0 ml, 0.20 M). Purification by flash column chromatography (0 – 20 CH_2Cl_2 in pentane) afforded 3,3-diethyl-2-(2-methoxyphenyl)azetidine-1-sulfonyl fluoride (**3b**) as a yellow oil (48.9 mg, 81%).

R_f (15% CH_2Cl_2 in pentane) = 0.20.

^{1}H NMR (600 MHz, $CDCl_3$) δ 7.56 (dd, *J* = 7.7, 1.7 Hz, 1H), 7.29 (td, *J* = 7.8, 1.7 Hz, 1H), 7.03 (t, *J* = 7.5 Hz, 1H), 6.84 (d, *J* = 8.2 Hz, 1H), 5.60 (d, *J* = 2.1 Hz, 1H), 3.93 (dd, *J* = 8.0, 2.0 Hz, 1H), 3.80 (s, 3H), 3.68 (dd, *J* = 8.0, 1.9 Hz, 1H), 1.85 (dq, *J* = 14.8, 7.4 Hz, 1H), 1.72 (dq, *J* = 14.7, 7.5 Hz, 1H), 1.38 (dq, *J* = 14.9, 7.7 Hz, 1H), 1.21 (dq, *J* = 14.6, 7.4 Hz, 1H), 1.02 (t, *J* = 7.5 Hz, 3H), 0.55 (t, *J* = 7.4 Hz, 3H).

^{13}C NMR (151 MHz, $CDCl_3$) δ 156.4, 129.2, 127.9, 124.1, 109.8, 70.1, 59.0, 55.1, 43.7, 28.6, 25.5, 8.3, 7.3.

^{19}F NMR (377 MHz, $CDCl_3$) δ 31.20 (s).

HRMS (ESI) *m/z* $C_{14}H_{20}FNO_3SNa^+$ requires 324.1040 ([M+Na]$^+$), found 324.1031.

IR (thin film, v_{max} /cm^{-1}) 2971, 1494, 1464, 1422, 1298, 1248, 1215, 1090, 1051, 1031, 755.

3,3-Diethyl-2-(2-fluoro-6-methoxyphenyl)azetidine-1-sulfonyl fluoride (3c)

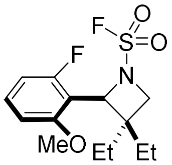

Prepared according to **GP1** using (2-fluoro-6-methoxybenzylidene)sulfamoyl fluoride (**I-3**) (47.0 mg, 0.20 mmol, 1.0 eq.), ITX (5.1 mg, 0.020 mmol, 0.10 eq.) and 2-ethyl-1-butene (241 µl, 2.0 mmol, 10 eq.) in $(CH_2Cl)_2$ (1.0 ml, 0.20 M). Purification by flash column chromatography (0 – 20% CH_2Cl_2 in pentane) afforded 3,3-diethyl-2-(2-fluoro-6-methoxyphenyl)azetidine-1-sulfonyl fluoride (**3c**) as a colourless oil (26.8 mg, 42%).

R_f (15% CH_2Cl_2 in pentane) = 0.19.

^{1}H NMR (600 MHz, $CDCl_3$) δ 7.25 (td, *J* = 8.4, 6.2 Hz, 1H), 6.73 (dd, *J* = 10.8, 8.4 Hz, 1H), 6.68 (d, *J* = 7.9 Hz, 1H), 5.59 (s, 1H), 3.96 – 3.88 (m, 1H), 3.88 – 3.77 (m, 4H), 1.85 (dq, *J* = 14.7, 7.4 Hz, 1H), 1.76 (dq, *J* = 14.6, 7.4 Hz, 1H), 1.63 (dq, *J* = 14.8, 7.5 Hz, 1H), 1.39 (dq, *J* = 14.6, 7.4 Hz, 1H), 0.93 (t, *J* = 7.4 Hz, 4H), 0.60 (t, *J* = 7.5 Hz, 3H).

^{13}C NMR (151 MHz, $CDCl_3$) δ 162.1 (d, $^1J_{C-F}$ = 250.7 Hz), 158.2, 130.2 (d, $^2J_{C-F}$ = 11.4 Hz), 111.8 (d, $^2J_{C-F}$ = 11.9 Hz), 109.3, 106.3, 67.5, 60.1, 56.2, 44.3, 29.1, 24.2, 8.0, 7.5.

^{19}F NMR (565 MHz, $CDCl_3$) δ 34.08(s), -112.60(s).

HRMS (ESI) *m/z* $C_{14}H_{19}F_2NO_3SNa^+$ requires 342.0946 ([M+Na]$^+$), found 343.09301.

IR (thin film, v_{max} /cm^{-1}) 2973, 1616, 1587, 1475, 1420, 1275, 1241, 1217, 1081, 751.

3,3-Diethyl-2-phenylazetidine-1-sulfonyl fluoride (3d)

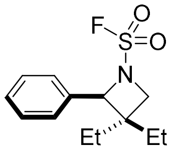

Prepared according to **GP1** using benzylidenesulfamoyl fluoride (**I-4**) (37.4 mg, 0.20 mmol, 1.0 eq.), 3-F-TX (4.6 mg, 0.020 mmol, 0.10 eq.) and 2-ethyl-1-butene (241 µl, 2.0 mmol, 10 eq.) in $(CH_2Cl)_2$ (1.0 ml, 0.20 M). Purification by flash column chromatography (0 – 20% CH_2Cl_2 in pentane) afforded 3,3-diethyl-2-phenylazetidine-1-sulfonyl fluoride (**3d**) as a colourless oil (40.9 mg, 75%).

R_f (20% CH_2Cl_2 in pentane) = 0.17.

^{1}H NMR (600 MHz, $CDCl_3$) δ 7.40 – 7.34 (m, 4H), 7.33 – 7.30 (m, 1H), 5.24 (d, *J* = 2.1 Hz, 1H), 3.88 (ddd, *J* = 7.9, 2.1, 1.0 Hz, 1H), 3.75 (dd, *J* = 7.9, 1.8 Hz, 1H), 1.84 (dq, *J* = 14.8, 7.5 Hz, 1H), 1.77 (dq, *J* = 14.9, 7.5 Hz, 1H), 1.36 (dq, *J* = 14.9, 7.5 Hz, 1H), 1.15 (dq, *J* = 14.6, 7.4 Hz, 1H), 0.97 (t, *J* = 7.5 Hz, 3H), 0.58 (t, *J* = 7.5 Hz, 3H).

^{13}C NMR (101 MHz, $CDCl_3$) δ 135.2, 128.5, 128.3, 126.9, 75.4, 60.0, 43.7, 29.3, 24.5, 8.2, 7.3.

^{19}F NMR (565 MHz, $CDCl_3$) δ 31.51(s).

HRMS (ESI) *m/z* $C_{13}H_{22}FN_2O_2S^+$ requires 289.1381 ($[M+NH_4]^+$), found 289.1381.

IR (thin film, v_{max} /cm^{-1}) 2971, 1456, 1425, 1217,1095, 761.

3,3-Diethyl-2-(p-tolyl)azetidine-1-sulfonyl fluoride (3e)

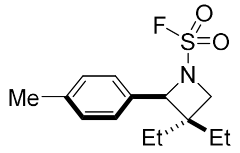

Prepared according to **GP1** using (4-methylbenzylidene)sulfamoyl fluoride (**I-5**) (40.2 mg, 0.20 mmol, 1.0 eq.), ITX (5.1 mg, 0.020 mmol, 0.10 eq.) and 2-ethyl-1-butene (241 µl, 2.0 mmol, 10 eq.) in $(CH_2Cl)_2$ (1.0 ml, 0.20 M). Purification by flash column chromatography (0 – 20% CH_2Cl_2 in pentane) afforded 3,3-diethyl-2-(p-tolyl)azetidine-1-sulfonyl fluoride (**3e**) as a colourless oil (40.9 mg, 86%).

R_f (20% CH_2Cl_2 in pentane) = 0.33.

^{1}H NMR (600 MHz, $CDCl_3$) δ 7.27 – 7.23 (m, 2H), 7.20 – 7.16 (m, 2H), 5.20 (d, *J* = 2.0 Hz, 1H), 3.86 (dd, *J* = 7.9, 1.1 Hz, 1H), 3.74 (dd, *J* = 8.0, 1.8 Hz, 1H), 2.36 (s, 3H), 1.82 (dq, *J* = 13.3, 6.7 Hz, 1H), 1.76 (dq, *J* = 14.3, 7.3 Hz, 1H), 1.38 (dq, *J* = 14.4, 7.3 Hz, 1H), 1.15 (dq, *J* = 14.6, 7.4 Hz, 1H), 0.96 (t, *J* = 7.5 Hz, 3H), 0.59 (t, *J* = 7.5 Hz, 3H).

^{13}C NMR (151 MHz, $CDCl_3$) δ 138.1, 132.3, 129.2, 126.8, 75.5, 60.0, 43.7, 29.3, 24.4, 21.3, 8.1, 7.3.

^{19}F NMR (565 MHz, $CDCl_3$) δ 31.41(s).

No relevant ions were detected by ESI, APCI and EI.

IR (thin film, v_{max} /cm^{-1}) 2971, 2939, 1763, 1517, 1424, 1216, 1096, 1055, 820, 747, 727, 626.

3,3-Diethyl-2-(4-(trimethylsilyl)phenyl)azetidine-1-sulfonyl fluoride (3f)

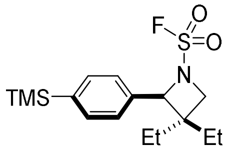

Prepared according to **GP1** using (4-(trimethylsilyl)benzylidene)sulfamoyl fluoride (**I-6**) (51.9 mg, 0.20 mmol, 1.0 eq.), 3-F-TX (4.6 mg, 0.020 mmol, 0.10 eq.) and 2-ethyl-1-butene (241 µl, 2.0 mmol, 10 eq.) in $(CH_2Cl)_2$ (1.0 ml, 0.20 M). Purification by flash column chromatography (0 – 20% CH_2Cl_2 in pentane) afforded 3,3-diethyl-2-(4-(trimethylsilyl)phenyl)azetidine-1-sulfonyl fluoride (**3f**) as a white solid (48.1 mg, 70%).

R_f (10% CH_2Cl_2 in pentane) = 0.10.

^{1}H NMR (600 MHz, $CDCl_3$) ^{1}H NMR (500 MHz, $CDCl_3$) δ 7.55 – 7.49 (m, 2H), 7.34 – 7.29 (m, 2H), 5.22 (d, *J* = 2.0 Hz, 1H), 3.87 (d, *J* = 7.8 Hz, 1H), 3.76 (dd, *J* = 7.9, 1.8 Hz, 1H), 1.83 (dq, *J* = 14.7, 7.4 Hz, 1H), 1.77 (dq, *J* = 14.5, 7.4 Hz, 1H), 1.37 (dq, *J* = 14.9, 7.5 Hz, 1H), 1.13 (dq, *J* = 14.7, 7.6 Hz, 1H), 0.97 (t, *J* = 7.5 Hz, 3H), 0.59 (t, *J* = 7.4 Hz, 3H), 0.26 (s, 9H).

^{13}C NMR (151 MHz, $CDCl_3$) δ 140.6, 135.6, 133.5, 126.1, 75.5, 60.0, 43.7, 29.3, 24.5, 8.2, 7.3, -1.0.

^{19}F NMR (565 MHz, $CDCl_3$) δ 31.41(s).

HRMS (ESI) *m/z* $C_{16}H_{26}FNO_2SSiNa^+$ requires 366.1330 ($[M+Na]^+$), found 366.1329.

IR (thin film, v_{max} /cm^{-1}) 2968, 1427, 1250, 1216, 1110, 1093, 854, 842, 760.

m.p. 60 – 62 °C.

3,3-Diethyl-2-(4-fluorophenyl)azetidine-1-sulfonyl fluoride (3g)

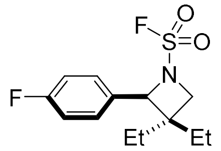

Prepared according to **GP1** using (4-fluorobenzylidene)sulfamoyl fluoride (**I-7**) (41.0 mg, 0.20 mmol, 1.0 eq.), 3-F-TX (4.6 mg, 0.020 mmol, 0.10 eq.) and 2-ethyl-1-butene (241 µl, 2.0 mmol, 10 eq.) in $(CH_2Cl)_2$ (1.0 ml, 0.20 M). Purification by flash column chromatography (0 – 20% CH_2Cl_2 in pentane) afforded 3,3-diethyl-2-(4-fluorophenyl)azetidine-1-sulfonyl (**3g**) as a colourless oil (45.3 mg, 78%).

R_f (20% CH_2Cl_2 in pentane) = 0.25.

^{1}H NMR (600 MHz, $CDCl_3$) δ 7.37 – 7.30 (m, 2H), 7.11 – 7.04 (m, 2H), 5.20 (d, J = 2.2 Hz, 1H), 3.87 (dd, J = 7.9, 1.1 Hz, 1H), 3.74 (dd, J = 8.0, 1.7 Hz, 1H), 1.82 (dq, J = 14.9, 7.5 Hz, 1H), 1.76 (dq, J = 14.6, 7.5 Hz, 1H), 1.36 (dq, J = 14.9, 7.5 Hz, 1H), 1.14 (dq, J = 14.6, 7.4 Hz, 1H), 0.96 (t, J = 7.5 Hz, 3H), 0.59 (t, J = 7.4 Hz, 3H).

^{13}C NMR (151 MHz, $CDCl_3$) δ 162.7 (d, $^1J_{C-F}$ = 247.0 Hz), 131.1 (d, $^4J_{C-F}$ = 3.1 Hz), 128.6 (d, $^3J_{C-F}$ = 8.2 Hz), 115.5 (d, $^2J_{C-F}$ = 21.8 Hz), 74.8, 60.0, 43.7, 29.2, 24.5, 8.1, 7.3.

^{19}F(^{1}H) NMR (377 MHz, $CDCl_3$) δ 31.27(s), -113.81(s).

No relevant ions were detected by ESI, APCI and EI.

IR (thin film, v_{max} /cm^{-1}) 2972, 1493, 1461, 1422, 1248, 1215, 1090, 1052, 1031, 755.

2-(4-Chlorophenyl)-3,3-diethylazetidine-1-sulfonyl fluoride (3h)

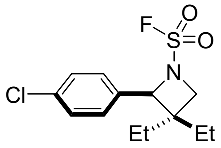

Prepared according to **GP1** using (4-chlorobenzylidene)sulfamoyl fluoride (**I-8**) (44.3 mg, 0.20 mmol, 1.0 eq.), 3-F-TX (4.6 mg, 0.020 mmol, 0.10 eq.) and 2-ethyl-1-butene (241 µl, 2.0 mmol, 10 eq.) in $(CH_2Cl)_2$ (1.0 ml, 0.20 M). Purification by flash column chromatography (0 – 20% CH_2Cl_2 in pentane) afforded 2-(4-chlorophenyl)-3,3-diethylazetidine-1-sulfonyl fluoride (**3h**) as a colourless oil (45.4 mg, 74%).

R_f (20% CH_2Cl_2 in pentane) = 0.32.

^{1}H NMR (600 MHz, $CDCl_3$) δ 7.36 (td, J = 13.8, 7.5 Hz, 2H), 7.29 (td, J = 4.0, 2.0 Hz, 2H), 5.19 (s, 1H), 3.87 (ddd, J = 7.9, 2.0, 1.0 Hz, 1H), 3.74 (dd, J = 8.0, 1.7 Hz, 1H), 1.82 (dq, J = 14.3, 7.4 Hz, 1H), 1.76 (dq, J = 14.4, 7.1 Hz, 1H), 1.35 (dq, J = 14.9, 7.5 Hz, 1H), 1.13 (dq, J = 14.7, 7.4 Hz, 1H), 0.96 (t, J = 7.5 Hz, 3H), 0.59 (t, J = 7.4 Hz, 3H).

^{13}C NMR (151 MHz, $CDCl_3$) δ 134.3, 133.8, 128.8, 128.2, 74.7, 60.0, 43.7, 29.2, 24.5, 8.2, 7.3.

^{19}F NMR (565 MHz, $CDCl_3$) δ 31.42(s).

No relevant ions were detected by ESI, APCI and EI.

IR (thin film, v_{max} /cm^{-1}) 2972, 2941, 1764, 1458, 1426, 1217, 1095, 753, 610.

2-(4-Bromophenyl)-3,3-diethylazetidine-1-sulfonyl fluoride (3i)

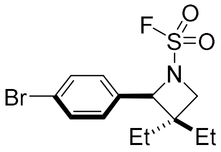

Prepared according to **GP1** using (4-bromobenzylidene)sulfamoyl fluoride (**I-9**) (53.2 mg, 0.20 mmol, 1.0 eq.), 3-F-TX (4.6 mg, 0.020 mmol, 0.10 eq.) and 2-ethyl-1-butene (241 µl, 2.0 mmol, 10 eq.) in $(CH_2Cl)_2$ (1.0 ml, 0.20 M). Purification by flash column chromatography (0 – 20% CH_2Cl_2 in pentane) afforded 2-(4-bromophenyl)-3,3-diethylazetidine-1-sulfonyl fluoride (**3i**) as a colourless oil (50.7 mg, 72%).

R_f (20% CH_2Cl_2 in pentane) = 0.29.

^{1}H NMR (600 MHz, CDCl$_3$) δ 7.54 – 7.48 (m, 2H), 7.25 – 7.21 (m, 2H), 5.17 (d, J = 2.4 Hz, 1H), 3.87 (dd, J = 8.0, 1.0 Hz, 1H), 3.74 (dd, J = 8.0, 1.7 Hz, 1H), 1.82 (dq, J = 12.8, 6.4 Hz, 1H), 1.76 (dq, J = 14.4, 7.3 Hz, 1H), 1.34 (dq, J = 14.9, 7.5 Hz, 1H), 1.13 (dq, J = 14.6, 7.4 Hz, 1H), 0.96 (t, J = 7.5 Hz, 3H), 0.60 (t, J = 7.4 Hz, 3H).

^{13}C NMR (151 MHz, CDCl$_3$) δ 134.4, 131.7, 128.6, 122.4, 74.7, 60.0, 43.7, 29.2, 24.5, 8.2, 7.3.

^{19}F NMR (565 MHz, CDCl$_3$) δ 31.44(s).

No relevant ions were detected by ESI, APCI and EI.

IR (thin film, v_{max} /cm^{-1}) 2972, 2940, 1765, 1490, 1425, 1216, 1074, 1011, 837, 751, 623.

2-([1,1'-Biphenyl]-4-yl)-3,3-diethylazetidine-1-sulfonyl fluoride (3j)

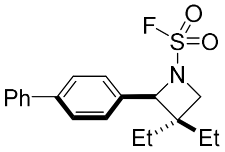

Prepared according to **GP1** using ([1,1'-biphenyl]-4-ylmethylene)sulfamoyl fluoride (**I-10**) (52.7 mg, 0.20 mmol, 1.0 eq.), ITX (5.1 mg, 0.020 mmol, 0.10 eq.) and 2-ethyl-1-butene (241 µl, 2.0 mmol, 10 eq.) in $(CH_2Cl)_2$ (1.0 ml, 0.20 M). Purification by flash column chromatography (0 – 20% CH_2Cl_2 in pentane) afforded 2-([1,1'-biphenyl]-4-yl)-3,3-diethylazetidine-1-sulfonyl fluoride (**3j**) as a white solid (48.5 mg, 70%).

R_f (20% CH_2Cl_2 in pentane) = 0.15.

^{1}H NMR (600 MHz, CDCl$_3$) δ 7.63 – 7.59 (m, 4H), 7.47 – 7.42 (m, 4H), 7.38 – 7.34 (m, 1H), 5.29 (d, *J* = 2.3 Hz, 1H), 3.90 (d, *J* = 8.0 Hz, 1H), 3.79 (dd, *J* = 8.0, 1.6 Hz, 1H), 1.86 (dq, *J* = 15.0, 7.6 Hz, 1H), 1.80 (dq, *J* = 14.8, 7.5 Hz, 1H), 1.43 (dq, *J* = 13.7, 6.9 Hz, 1H), 1.21 (dq, *J* = 14.6, 7.3 Hz, 1H), 1.00 (t, *J* = 7.5 Hz, 3H), 0.62 (t, *J* = 7.4 Hz, 3H).

^{13}C NMR (151 MHz, CDCl$_3$) δ 141.1, 140.5, 134.1, 128.8, 127.5, 127.2, 127.1, 75.2, 59.9, 43.7, 29.2, 24.4, 8.1, 7.2.

Note: 1 Carbon environment not observed due to signal overlap.

^{19}F NMR (565 MHz, CDCl$_3$) δ 31.49(s).

No relevant ions were detected by ESI, APCI and EI.

IR (thin film, ν_{max} /cm^{-1}) 2974, 2942, 1765, 1489, 1423, 1214, 1033, 763, 678.

m.p. 95 – 98 °C.

3,3-Diethyl-2-(o-tolyl)azetidine-1-sulfonyl fluoride (3k)

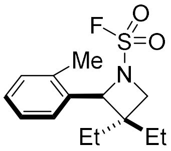

Prepared according to **GP1** using (2-methylbenzylidene)sulfamoyl fluoride (**I-11**) (40.2 mg, 0.20 mmol, 1.0 eq.), ITX (5.1 mg, 0.020 mmol, 0.10 eq.) and 2-ethyl-1-butene (73 µl, 0.60 mmol, 3.0 eq.) in $(CH_2Cl)_2$ (1.0 ml, 0.20 M). Purification by flash column chromatography (0 – 20% CH_2Cl_2 in pentane) afforded 3,3-diethyl-2-(o-tolyl)azetidine-1-sulfonyl fluoride (**3k**) as a colourless oil (40.9 mg, 49%).

R_f (10% CH_2Cl_2 in pentane) = 0.13.

^{1}H NMR (400 MHz, $CDCl_3$) δ 7.64 (d, *J* = 7.7 Hz, 1H), 7.29 (dd, *J* = 7.4, 1.6 Hz, 1H), 7.22 (td, *J* = 7.4, 1.5 Hz, 1H), 7.13 (dd, *J* = 7.3, 1.5 Hz, 1H), 5.44 (d, *J* = 2.1 Hz, 1H), 4.00 (dd, *J* = 7.9, 2.1 Hz, 1H), 3.64 (dd, *J* = 8.0, 1.9 Hz, 1H), 2.29 (s, 3H), 1.90 (dq, *J* = 14.8, 7.5 Hz, 1H), 1.73 (dq, *J* = 14.7, 7.5 Hz, 1H), 1.46 (dq, *J* = 15.0, 7.5 Hz, 1H), 1.38 (dq, *J* = 14.6, 7.4 Hz, 1H), 1.03 (t, *J* = 7.4 Hz, 3H), 0.43 (t, *J* = 7.4 Hz, 3H).

^{13}C NMR (101 MHz, $CDCl_3$) δ 134.8, 133.7, 130.4, 128.2, 127.3, 126.2, 71.6, 58.6, 44.1, 28.8, 26.0, 19.7, 8.5, 7.3.

^{19}F NMR (377 MHz, $CDCl_3$) δ 31.91(s).

HRMS (ESI) *m/z* $C_{14}H_{20}FNO_2SNa^+$ requires 308.1091 ($[M+Na]^+$), found 308.1085.

IR (thin film, ν_{max} /cm^{-1}) 2972, 2883, 1424, 1216, 1093, 1055, 1032, 918, 759, 621.

2-(4-(1,3-Dioxoisoindolin-2-yl)phenyl)-3,3-diethylazetidine-1-sulfonyl fluoride (3l)

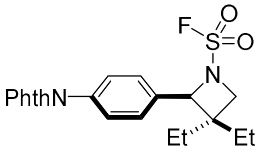

Prepared according to **GP1** using (4-(1,3-dioxoisoindolin-2-yl)benzylidene)sulfamoyl fluoride (**I-12**) (66.5 mg, 0.20 mmol, 1.0 eq.), 3-F-TX (4.6 mg, 0.020 mmol, 0.10 eq.) and 2-ethyl-1-butene (241 µl, 2.0 mmol, 10 eq.) in $(CH_2Cl)_2$ (3.0 ml, 0.067 M). Purification by flash column chromatography (20% EtOAc in pentane) afforded 2-(4-(1,3-dioxoisoindolin-2-yl)phenyl)-3,3-diethylazetidine-1-sulfonyl fluoride (**3l**) as a yellow solid (67.4 mg, 81%).

R_f (20% ethyl acetate in pentane) = 0.16.

^{1}H NMR (600 MHz, $CDCl_3$) δ 7.95 (dd, *J* = 5.4, 3.1 Hz, 2H), 7.79 (dd, *J* = 5.4, 3.0 Hz, 2H), 7.53 – 7.46 (m, 4H), 5.28 (d, *J* = 1.6 Hz, 1H), 3.89 (d, *J* = 8.0 Hz, 1H), 3.78 (dd, *J* = 7.9, 1.3 Hz, 1H), 1.85 (dq, *J* = 14.7, 7.4 Hz, 1H), 1.79 (dq, *J* = 14.6, 7.4 Hz, 1H), 1.39 (dq, *J* = 14.8, 7.5 Hz, 1H), 1.17 (dq, *J* = 14.6, 7.4 Hz, 1H), 0.99 (t, *J* = 7.4 Hz, 3H), 0.63 (t, *J* = 7.4 Hz, 3H).

^{13}C NMR (151 MHz, $CDCl_3$) δ 167.2, 135.0, 134.6, 131.8, 127.4, 126.3, 123.9, 74.9, 60.0, 43.8, 29.2, 24.7, 8.2, 7.3.

Note: 1 Carbon environment not observed due to signal overlap.

^{19}F NMR (565 MHz, $CDCl_3$) δ 31.69(s).

HRMS (ESI) *m/z* $C_{21}H_{22}FN_2O_4S^+$ requires 417.1279 ($[M+H]^+$), found 417.1288.

IR (thin film, v_{max} /cm^{-1}) 2970, 1722, 1517, 1424, 1383, 1215, 1084, 720.

m.p. 140 °C (decomp.).

4-(3,3-Diethyl-1-(fluorosulfonyl)azetidin-2-yl)phenyl pivalate (3m)

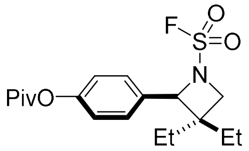

Prepared according to **GP1** using 4-(((fluorosulfonyl)imino)methyl)phenyl pivalate (**I-13**) (57.5 mg, 0.20 mmol, 1.0 eq.), 3-F-TX (4.6 mg, 0.020 mmol, 0.10 eq.) and 2-ethyl-1-butene (241 µl, 2.0 mmol, 10 eq.) in $(CH_2Cl)_2$ (1.0 ml, 0.20 M). Purification by flash column chromatography (0 – 4% Et_2O in pentane) afforded 4-(3,3-diethyl-1-(fluorosulfonyl)azetidin-2-yl)phenyl pivalate (**3m**) as a colourless crystalline solid (43.9 mg, 59%).

R_f (5% Et_2O in pentane) = 0.24.

^{1}H NMR (600 MHz, $CDCl_3$) δ 7.37 – 7.34 (m, 2H), 7.11 – 7.08 (m, 2H), 5.22 (d, *J* = 1.8 Hz, 1H), 3.86 (d, *J* = 7.9 Hz, 1H), 3.74 (d, *J* = 8.0 Hz, 1H), 1.82 (dq, *J* = 14.5, 7.3 Hz, 1H), 1.76 (dq, *J* = 14.6, 7.3 Hz, 1H), 1.39 – 1.32 (m, 10H), 1.14 (dq, *J* = 14.6, 7.4 Hz, 1H), 0.95 (t, *J* = 7.4 Hz, 3H), 0.58 (t, *J* = 7.4 Hz, 3H).

^{13}C NMR (151 MHz, $CDCl_3$) δ 177.0, 151.1, 132.5, 127.8, 121.6, 74.9, 60.0, 43.7, 39.2, 29.2, 27.2, 24.5, 8.1, 7.2.

^{19}F NMR (565 MHz, $CDCl_3$) δ 31.49(s).

HRMS (ESI) *m/z* $C_{18}H_{26}FNO_4SNa^+$ requires 394.1459 ($[M+Na]^+$), found 394.1463.

IR (thin film, v_{max} /cm^{-1}) 2973, 1755, 1509, 1425, 1215, 1168, 1120, 899, 748.

m.p. 82 – 86 °C.

Methyl 3-(3,3-diethyl-1-(fluorosulfonyl)azetidin-2-yl)benzoate (3n)

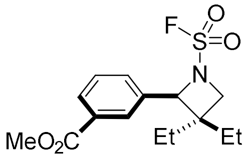

Prepared according to **GP1** using methyl 3-(((fluorosulfonyl)imino)methyl)benzoate (**I-14**) (49.0 mg, 0.20 mmol, 1.0 eq.), 3-F-TX (4.6 mg, 0.020 mmol, 0.10 eq.) and 2-ethyl-1-butene (241 µl, 2.0 mmol, 10 eq.) in $(CH_2Cl)_2$ (1.0 ml, 0.20 M). Purification by flash column chromatography (0 – 10% Et_2O in pentane) afforded methyl 3-(3,3-diethyl-1-(fluorosulfonyl)azetidin-2-yl)benzoate (**3n**) as a colourless oil (41.0 mg, 62%)

R_f (10% Et_2O in pentane) = 0.30.

^{1}H NMR (400 MHz, $CDCl_3$) δ 8.01 (s, 1H), 7.99 (d, *J* = 1.6 Hz, 1H), 7.57 (dd, *J* = 7.8, 1.7 Hz, 1H), 7.46 (t, *J* = 7.9 Hz, 1H), 5.26 (s, 1H), 3.93 (s, 1H), 3.89 (d, *J* = 8.2 Hz, 1H), 3.77 (d, *J* = 7.9 Hz, 1H), 1.89 – 1.72 (m,

2H), 1.35 (dq, J = 14.9, 7.8 Hz, 1H), 1.10 (dq, J = 14.6, 7.4 Hz, 1H), 0.97 (t, J = 7.5 Hz, 1H), 0.56 (t, J = 7.4 Hz, 1H).

^{13}C NMR (101 MHz, CDCl$_3$) δ 166.8, 135.8, 131.3, 130.6, 129.6, 128.7, 127.9, 74.8, 59.9, 52.4, 43.7, 29.2, 24.6, 8.2, 7.3.

^{19}F NMR (377 MHz, CDCl$_3$) δ 31.20(s).

HRMS (ESI) *m/z* $C_{15}H_{21}FNO_4S^+$ requires 330.1170 ([M+H]$^+$), found 330.1158.

IR (thin film, ν_{max} /cm^{-1}) 2971, 1726, 1424, 1294, 1214, 759, 620.

3,3-Diethyl-2-(4-(trifluoromethyl)phenyl)azetidine-1-sulfonyl fluoride (3o)

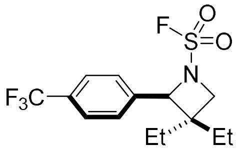

Prepared according to **GP1** using (4-(trifluoromethyl)benzylidene)sulfamoyl fluoride (**I-15**) (51.0 mg, 0.20 mmol, 1.0 eq.), 3-F-TX (4.6 mg, 0.020 mmol, 0.10 eq.) and 2-ethyl-1-butene (241 µl, 2.0 mmol, 10 eq.) in $(CH_2Cl)_2$ (1.0 ml, 0.20 M). Purification by flash column chromatography (0 – 20% CH_2Cl_2 in pentane) afforded 3,3-diethyl-2-(4-(trifluoromethyl)phenyl)azetidine-1-sulfonyl fluoride (**3o**) as a white solid (40.8 mg, 60%).

R$_f$ (20% CH$_2$Cl$_2$ in pentane) = 0.27.

^{1}H NMR (600 MHz, CDCl$_3$) δ 7.68 – 7.63 (m, 2H), 7.51 – 7.46 (m, 2H), 5.27 (s, 1H), 3.90 (d, J = 8.0 Hz, 1H), 3.78 (dd, J = 8.0, 1.7 Hz, 1H), 1.85 (dq, J = 14.7, 7.4 Hz, 1H), 1.79 (dq, J = 14.6, 7.5 Hz, 1H), 1.33 (dq, J = 14.8, 7.5 Hz, 1H), 1.11 (dq, J = 13.6, 6.8 Hz, 1H), 0.99 (t, J = 7.5 Hz, 3H), 0.60 (t, J = 7.4 Hz, 3H).

^{13}C NMR (151 MHz, CDCl$_3$) δ 139.3, 130.6 (q, $^2J_{C-F}$ = 32.5 Hz), 127.2, 125.6 (q, $^3J_{C-F}$ = 3.8 Hz), 124.8 (q, $^1J_{C-F}$ = 272.0 Hz), 74.6, 60.0, 43.9, 29.2, 24.7, 8.2, 7.3.

^{19}F(^{1}H) NMR (377 MHz, CDCl$_3$) δ 31.31(s), -62.60(s).

No relevant ions were detected by ESI, APCI and EI.

IR (thin film, ν_{max} /cm^{-1}) 2974, 2943, 1764, 1427, 1329, 1218, 1169, 1131, 767, 623.

m.p. 62 – 68 °C.

2-(2,2-Difluorobenzo[d][1,3]dioxol-5-yl)-3,3-diethylazetidine-1-sulfonyl fluoride (3p)

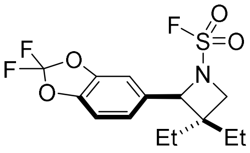

Prepared according to **GP1** using ((2,2-difluorobenzo[d][1,3]dioxol-5-yl)methylene)sulfamoyl fluoride (**I-16**) (53.4 mg, 0.20 mmol, 1.0 eq.), 3-F-TX (4.6 mg, 0.020 mmol, 0.10 eq.) and 2-ethyl-1-butene (241 µl, 2.00 mmol, 10 eq.) in $(CH_2Cl)_2$ (1.0 ml, 0.20 M). Purification by flash column chromatography (0 – 20% CH_2Cl_2 in pentane) afforded azetidine 2-(2,2-Difluorobenzo[d][1,3]dioxol-5-yl)-3,3-diethylazetidine-1-sulfonyl fluoride (**3p**) as a yellow oil (38.3 mg, 54 %).

R$_f$ (10% CH$_2$Cl$_2$ in pentane) = 0.14.

^{1}H NMR (600 MHz, CDCl$_3$) δ 7.13 (s, 1H), 7.07 – 7.04 (m, 2H), 5.19 (s, 1H), 3.87 (d, *J* = 8.0 Hz, 1H), 3.74 (d, *J* = 7.9 Hz, 1H), 1.78 (m, 2H), 1.37 (dq, *J* = 14.8, 7.5 Hz, 1H), 1.14 (dq, *J* = 15.0, 7.3 Hz, 1H), 0.96 (t, *J* = 7.5 Hz, 3H), 0.61 (t, *J* = 7.4 Hz, 3H).

^{13}C NMR (151 MHz, CDCl$_3$) δ 144.1, 143.7, 131.8 (t, $^{1}J_{C-F}$ = 255.6 Hz), 131.7, 122.3, 109.5, 108.4, 74.7, 59.9, 43.9, 29.2, 24.5, 8.1, 7.3.

^{19}F NMR (565 MHz, CDCl$_3$) δ 31.34(s), -49.71(s).

HRMS (ESI) *m/z* $C_{14}H_{16}F_3NO_4SNa^+$ requires 374.0644 ([M+Na]$^+$), found 374.0645.

IR (thin film, *ν$_{max}$* /cm^{-1}) 2974, 1503, 1427, 1256, 1216, 1162, 1037, 753, 623.

2-(4-(2-Bromoethoxy)phenyl)-3,3-diethylazetidine-1-sulfonyl fluoride (3q)

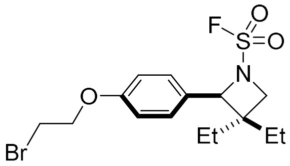

Prepared according to **GP1** (4-(2-bromoethoxy)benzylidene)sulfamoyl fluoride (**I-17**) (62.0 mg, 0.20 mmol, 1.0 eq.), 3-F-TX (4.6 mg, 0.020 mmol, 0.10 eq.) and 2-ethyl-1-butene (241 µl, 2.0 mmol, 10 eq.) in $(CH_2Cl)_2$ (1.0 ml, 0.20 M). Purification by flash column chromatography (0 – 30% CH_2Cl_2 in pentane) afforded 2-(4-(2-bromoethoxy)phenyl)-3,3-diethylazetidine-1-sulfonyl fluoride (**3q**) as a colourless oil (60.7 mg, 77%).

R*$_f$* (20% CH$_2$Cl$_2$ in pentane) = 0.12.

^{1}H NMR (600 MHz, CDCl$_3$) δ 7.30 – 7.27 (m, 2H), 6.95 – 6.89 (m, 2H), 5.17 (s, 1H), 4.29 (t, *J* = 6.2 Hz, 2H), 3.85 (d, *J* = 7.9 Hz, 1H), 3.72 (d, *J* = 8.7 Hz, 1H), 3.64 (t, *J* = 6.2 Hz, 2H), 1.80 (dq, *J* = 14.8, 7.4 Hz, 1H), 1.75 (dq, *J* = 14.6, 7.4 Hz, 1H), 1.38 (dq, *J* = 14.8, 7.5 Hz, 1H), 1.15 (dq, *J* = 15.0, 7.4 Hz, 1H), 0.94 (t, *J* = 7.4 Hz, 3H), 0.58 (t, *J* = 7.4 Hz, 3H).

^{19}F NMR (377 MHz, CDCl$_3$) δ 31.25(s).

^{13}C NMR (151 MHz, CDCl$_3$) δ 158.2, 128.3, 128.2, 114.7, 75.2, 68.0, 60.0, 43.7, 29.2, 29.2, 24.4, 8.1, 7.3.

HRMS (ESI) *m/z* $C_{15}H_{22}{}^{79}BrFNO_3S^+$ and $C_{15}H_{22}{}^{81}BrFNO_3S^+$requires 394.0482 and 396.0462 respectively ([M+H]$^+$), found 394.0487 and 396.0435.

IR (thin film, *ν$_{max}$* /cm^{-1}) 2973, 2941, 1765, 1462, 1445, 1250, 1214, 1033, 751, 684.

3,3-Diethyl-2-(2-methoxy-5-(4,4,5,5-tetraethyl-1,3,2-dioxaborolan-2-yl)phenyl)azetidine-1-sulfonyl fluoride (3r)

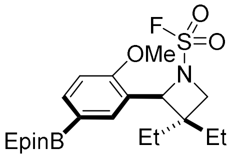

Prepared according to **GP1** using (2-methoxy-5-(4,4,5,5-tetraethyl-1,3,2-dioxaborolan-2-yl)benzylidene)sulfamoyl fluoride (**I-18**) (79.9 mg, 0.20 mmol, 1.0 eq.), 3-F-TX (4.6 mg, 0.020 mmol, 0.10 eq.) and 2-ethyl-1-butene (241 µl, 2.0 mmol, 10 eq.) in $(CH_2Cl)_2$ (1.0 ml, 0.20 M). Purification by flash column chromatography (0 – 10 Et_2O in pentane) afforded 3,3-diethyl-2-(2-methoxy-5-(4,4,5,5-

tetraethyl-1,3,2-dioxaborolan-2-yl)phenyl)azetidine-1-sulfonyl fluoride (**3r**) as a white solid (40.4 mg, 42%).

R$_f$ (10% Et$_2$O in pentane) = 0.26.

^{1}H NMR (400 MHz, CDCl$_3$) δ 7.94 (d, *J* = 1.6 Hz, 1H), 7.77 (dd, *J* = 8.0, 1.6 Hz, 1H), 6.82 (d, *J* = 8.2 Hz, 1H), 5.57 (d, *J* = 1.8 Hz, 1H), 3.92 (dd, *J* = 8.0, 1.8 Hz, 1H), 3.81 (s, 3H), 3.72 (dd, *J* = 8.0, 1.7 Hz, 1H), 1.90 – 1.64 (m, 10H), 1.38 (dq, *J* = 14.9, 7.5 Hz, 1H), 1.17 (dq, *J* = 14.6, 7.3 Hz, 1H), 1.04 – 0.91 (m, 15H), 0.54 (t, *J* = 7.4 Hz, 3H).

^{13}C NMR (151 MHz, CDCl$_3$) δ 158.8, 136.6, 134.3, 123.4, 109.2, 88.8, 70.2, 59.1, 55.1, 43.6, 28.7, 26.7, 26.5, 25.4, 9.1, 8.9, 8.3, 7.4.

^{19}F NMR (565 MHz, CDCl$_3$) δ 32.17(s).

HRMS (ESI) *m/z* $C_{24}H_{40}BFNO_5^+$ requires 484.2699 ([M+H]$^+$), found 484.2712.

IR (thin film, v_{max} /cm^{-1}) 2976, 1607, 1458, 1423, 1367, 1279, 1254, 1213, 1030, 924, 745.

m.p. 87 – 90 °C.

3,3-Diethyl-2-(3-hydroxy-4-methoxyphenyl)azetidine-1-sulfonyl fluoride (3s’)

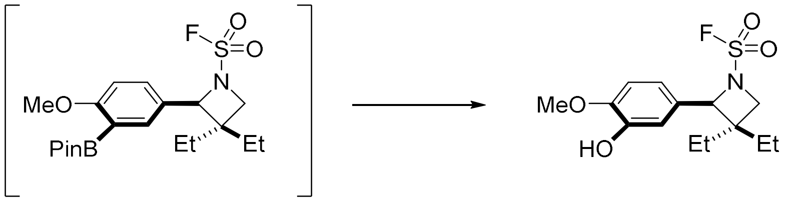

Prepared according to a modification of **GP1** using (4-methoxy-3-(4,4,5,5-tetramethyl-1,3,2-dioxaborolan-2-yl)benzylidene)sulfamoyl fluoride (**I-19**) (68.6 mg, 0.20 mmol, 1.0 eq.), 3-F-TX (4.6 mg, 0.020 mmol, 0.10 eq.) and 2-ethyl-1-butene (241 µl, 2.0 mmol, 10 eq.) in $(CH_2Cl)_2$ (1.0 ml, 0.20 M). Following the photochemical reaction, the reaction mixture was transferred to a round bottom flask and concentrated under reduced pressure. The crude reaction mixture was dissolved in a 1:1 mixture of THF/H_2O (3.2 ml, 0.063 M) followed by addition of $NaBO_3$ $4H_2O$ (462 mg, 3.0 mmol, 15 eq.). The reaction mixture was stirred under air at rt for 1 h after which the reaction mixture was transferred to a separatory funnel. Brine was added and the layers partitioned. The aqueous layer was extracted three times with Et_2O, the combined organic fractions were dried over anhydrous $MgSO_4$ and concentrated under reduced pressure. Purification by flash column chromatography (0 – 30% Et_2O in pentane) afforded 3,3-diethyl-2-(3-hydroxy-4-methoxyphenyl)azetidine-1-sulfonyl fluoride (**3s’**) as a colourless oil (39.5 mg, 62%).

R$_f$ (30% Et$_2$O in pentane) = 0.19.

^{1}H NMR (500 MHz, CDCl$_3$) δ 6.91 (d, *J* = 2.0 Hz, 1H), 6.88 – 6.83 (m, 2H), 5.66 (s, 1H), 5.12 (d, *J* = 2.0 Hz, 1H), 3.88 (s, 3H), 3.83 (dd, *J* = 7.9, 1.1 Hz, 1H), 3.71 (dd, *J* = 7.8, 1.8 Hz, 1H), 1.75 (m, 2H), 1.39 (m, 1H), 1.18 (dq, *J* = 14.6, 7.6 Hz, 1H), 0.94 (t, *J* = 7.4 Hz, 3H), 0.59 (t, *J* = 7.4 Hz, 3H).

^{13}C NMR (126 MHz, CDCl$_3$) δ 146.6, 145.6, 128.5, 118.7, 113.2, 110.5, 75.2, 60.0, 56.1, 43.7, 29.2, 24.2, 8.1, 7.3.

^{19}F NMR (471 MHz, CDCl$_3$) δ 31.47(s).

HRMS (ESI) *m/z* $C_{14}H_{20}FNO_4SNa^+$ requires 340.08989 ([M+Na]$^+$), found 340.0999.

IR (thin film, v_{max} /cm^{-1}) 3503, 2971, 1594, 1516, 1458, 1421, 1275, 1213, 1131, 1029, 762, 627.

3,3-Diethyl-2-(6-methoxypyridin-3-yl)azetidine-1-sulfonyl fluoride (3w)

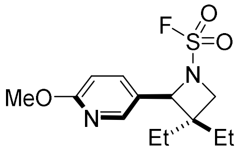

Prepared according to **GP1** using ((6-methoxypyridin-3-yl)methylene)sulfamoyl fluoride (**I-20**) (43.6 mg, 0.20 mmol, 1.0 eq.), 3-F-TX (4.6 mg, 0.020 mmol, 0.10 eq.) and 2-ethyl-1-butene (241 µl, 2.0 mmol, 10 eq.) in $(CH_2Cl)_2$ (1.0 ml, 0.20 M). Purification by flash column chromatography (80 – 90 CH_2Cl_2 in pentane) afforded 3,3-diethyl-2-(6-methoxypyridin-3-yl)azetidine-1-sulfonyl fluoride (**3w**) as a colourless oil (33.2 mg, 55%).

R$_f$ (10% EtOAc in petrol) = 0.29.

^{1}H NMR (600 MHz, CDCl$_3$) δ 8.12 (dt, J = 2.5, 0.7 Hz, 1H), 7.61 (dd, J = 8.6, 2.5 Hz, 1H), 6.76 (d, J = 8.6 Hz, 1H), 5.16 (d, J = 2.0 Hz, 1H), 3.93 (s, 3H), 3.85 (d, J = 7.8 Hz, 1H), 3.74 (dd, J = 8.0, 1.6 Hz, 1H), 1.80 (dq, *J* = 14.8, 7.5 Hz, 1H), 1.76 (dq, *J* = 14.5, 7.4 Hz, 1H), 1.41 (dq, J = 14.8, 7.5 Hz, 1H), 1.17 (dq, J = 14.6, 7.4 Hz, 1H), 0.92 (t, J = 7.5 Hz, 3H), 0.62 (t, J = 7.4 Hz, 3H).

^{19}F NMR (565 MHz, CDCl$_3$) δ 31.09 (s).

^{13}C NMR (151 MHz, CDCl$_3$) δ 164.4, 145.6, 137.4, 123.8, 110.8, 73.3, 60.1, 53.7, 43.8, 29.2, 24.4, 8.0, 7.3.

HRMS (ESI) *m/z* $C_{13}H_{20}FN_2O_3S^+$ requires 303.1173 ([M+H]$^+$), found 303.1173.

IR (thin film, v_{max} /cm^{-1}) 2972, 1611, 1498, 1425, 1290, 1216, 1028, 834, 747.

(2*R**,3*S**)-3-(2-Hydroxyethyl)-2-(4-methoxyphenyl)-3-methylazetidine-1-sulfonyl fluoride (4a) and (2*R**,3*R**)-3-(2-Hydroxyethyl)-2-(4-methoxyphenyl)-3-methylazetidine-1-sulfonyl fluoride (4a')

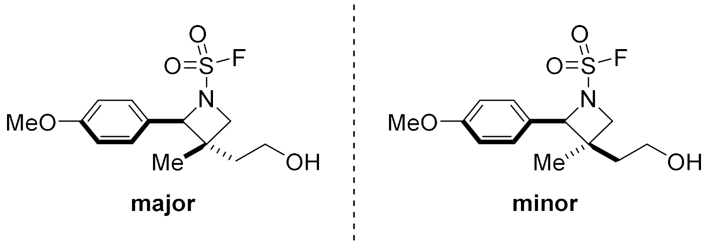

Prepared according to **GP1** using (4-methoxybenzylidene)sulfamoyl fluoride (**1)** (43.4 mg, 0.20 mmol, 1.0 eq.), ITX (5.1 mg, 0.020 mmol, 0.10 eq.) and 3-methylbut-3-en-1-ol (203 µl, 2.0 mmol, 10 eq.). ^{19}F NMR analysis of the crude reaction mixture revealed a diastereomer ratio of 1.2:1. Purification by flash column chromatography (0 – 50% ethyl acetate in pentane) afforded (2*R**,3*S**)-3-(2-Hydroxyethyl)-2-(4-methoxyphenyl)-3-methylazetidine-1-sulfonyl fluoride (**4a**) and (2*R**,3*R**)-3-(2-Hydroxyethyl)-2-(4-methoxyphenyl)-3-methylazetidine-1-sulfonyl fluoride (**4a'**) as an inseparable mixture of diastereomers as a colourless oil (52.7 mg, 87%).

major

R$_f$ (50% ethyl acetate in pentane) = 0.46.

^{1}H NMR (600 MHz, CDCl$_3$) δ 7.30 – 7.25 (m, 2H), 6.93 – 6.88 (m, 2H), 5.27 (s, 1H), 4.14 (d, *J* = 7.8 Hz, 1H), 3.80 (s, 3H), 3.83 – 3.77 (m, 1H), 3.78 – 3.70 (m, 1H), 3.68 (d, *J* = 8.0, 1 H), 2.00 (ddd, *J* = 13.9, 7.8, 6.1 Hz, 1H), 1.89 (dt, *J* = 14.1, 5.6 Hz, 1H), 0.90 (s, 3H).

^{13}C NMR (151 MHz, CDCl$_3$) δ 159.7, 127.8, 127.3, 114.0, 75.8, 62.2, 59.0, 55.4, 42.3, 39.1, 20.0.

^{19}F NMR (377 MHz, CDCl$_3$) 29.98 (s).

HRMS (ESI) *m/z* $C_{13}H_{18}FNO_4SNa$ requires 326.0833 ([M+Na]$^+$), found 326.0838.

IR (thin film, ν_{max} /cm^{-1}) 3380, 2920, 1515, 1419, 1252, 1211, 1031, 840, 756.

minor

R$_f$ (50% ethyl acetate in pentane) = 0.46.

^{1}H NMR (600 MHz, CDCl$_3$) δ 7.30 – 7.25 (m, 2H), 6.93 – 6.88 (m, 2H), 5.13 (s, 1H), 3.98 (dd, *J* = 8.0, 1.5 Hz, 1H), 3.93 (d, *J* = 8.4 Hz, 1H), 3.80 (s, 3H), 3.54 – 3.45 (m, 2H), 1.71 (app. dt, *J* = 14.6, 7.5 Hz, 1H), 1.41 (s, 3H), 1.17 – 1.10 (m, 1H).

^{13}C NMR (151 MHz, CDCl$_3$) δ 159.9, 127.7, 126.9, 114.1, 77.6, 61.7, 58.9, 55.4, 39.0, 37.4, 24.6.

^{19}F NMR (377 MHz, CDCl$_3$) 30.33 (s).

HRMS (ESI) *m/z* $C_{13}H_{18}FNO_4SNa$ requires 326.0833 ([M+Na]$^+$), found 326.0838.

IR (thin film, ν_{max} /cm^{-1}) 3380, 2920, 1515, 1419, 1252, 1211, 1031, 840, 756.

2-((2*R**,3*S**)-1-(Fluorosulfonyl)-2-(4-methoxyphenyl)-3-methylazetidin-3-yl)ethyl 4-methylbenzenesulfonate (4b) and 2-((2*R**,3*R**)-1-(Fluorosulfonyl)-2-(4-methoxyphenyl)-3-methylazetidin-3-yl)ethyl 4-methylbenzenesulfonate (4b')

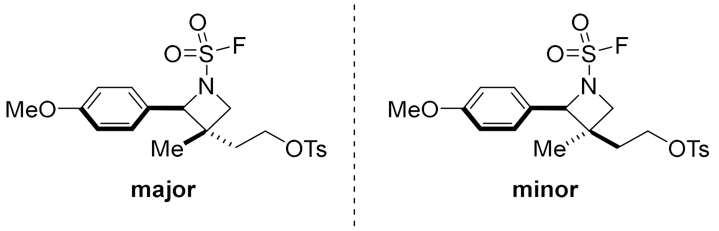

Prepared according to **GP1** using (4-methoxybenzylidene)sulfamoyl fluoride (**1**) (43.4 mg, 0.20 mmol, 1.0 eq.), 3-F-TX (4.6 mg, 0.020 mmol, 0.10 eq.) and 3-methylbut-3-en-1-yl 4-methylbenzenesulfonate (**O-29**) (481 mg, 2.0 mmol, 10 eq.) in $(CH_2Cl)_2$ (1.0 ml, 0.20 M). ^{19}F NMR analysis of the crude reaction mixture revealed a diastereomer ratio of 1.1:1. Purification by flash column chromatography (0 – 40% Et_2O in pentane) afforded a 3:1 mixture of both 2-((2*R**,3*S**)-1-(fluorosulfonyl)-2-(4-methoxyphenyl)-3-methylazetidin-3-yl)ethyl 4-methylbenzenesulfonate (**4b**) and 2-((2*R**,3*R**)-1-(fluorosulfonyl)-2-(4-methoxyphenyl)-3-methylazetidin-3-yl)ethyl 4-methylbenzenesulfonate (**4b'**) as a yellow oil (52.6 mg, 57%) and 2-((2*R**,3*R**)-1-(fluorosulfonyl)-2-(4-methoxyphenyl)-3-methylazetidin-3-yl)ethyl 4-methylbenzenesulfonate (**4b'**) as a yellow oil (28.5 mg, 31%).

major

R$_f$ (40% Et_2O in pentane) = 0.16.

^{1}H NMR (700 MHz, CDCl$_3$) δ 7.78 – 7.75 (m, 2H), 7.36 (d, *J* = 8.1 Hz, 2H), 7.22 (d, *J* = 8.5 Hz, 2H), 6.91 – 6.88 (m, 2H), 5.10 (s, 1H), 4.11 – 4.08 (m, 2H), 3.99 (d, *J* = 8.1 Hz, 1H), 3.80 (s, 3H), 3.63 (d, *J* = 8.1 Hz, 1H), 2.45 (s, 3H), 2.13 (dt, *J* = 14.4, 7.1 Hz, 1H), 1.95 (dt, *J* = 14.6, 5.2 Hz, 1H), 0.81 (s, 3H).

^{13}C NMR (176 MHz, CDCl$_3$) δ 159.9, 145.5, 132.6, 130.2, 128.0, 127.9, 126.4, 114.1, 75.7, 66.3, 61.7, 55.4, 38.9, 38.7, 21.8, 19.2.

^{19}F NMR (377 MHz, CDCl3) δ 30.25(s).

HRMS (ESI) *m/z*; $C_{20}H_{25}FNO_6S_2$ requires 458.1102 ([M+H]$^+$), found 458.1097.

IR (thin film, ν_{max} /cm^{-1}) 1516, 1423, 1364, 1254, 1178, 1097, 1036, 974, 919, 818, 762, 740, 666.

minor

R$_f$ (40% Et$_2$O in pentane) = 0.22.

^{1}H NMR (700 MHz, CDCl$_3$) δ 7.72 – 7.68 (m, 2H), 7.34 (d, *J* = 8.0 Hz, 2H), 7.19 – 7.14 (m, 2H), 6.89 – 6.84 (m, 2H), 5.10 (s, 1H), 3.90 (d, *J* = 8.3 Hz, 1H), 3.88 – 3.84 (m, 2H), 3.82 – 3.78 (m, 4H), 2.46 (s, 3H), 1.81 (ddd, *J* = 15.0, 8.5, 6.6 Hz, 1H), 1.35 (s, 3H), 1.22 (dt, *J* = 14.9, 5.4 Hz, 1H).

^{13}C NMR (176 MHz, CDCl$_3$) δ 160.0, 145.3, 132.7, 130.1, 127.9, 127.5, 126.3, 114.3, 77.2, 66.4, 61.2, 55.4, 38.7, 34.1, 24.2, 21.8.

^{19}F NMR (377 MHz, CDCl$_3$) δ 30.55(s).

HRMS (ESI) *m/z*; $C_{20}H_{25}FNO_6S_2$ requires 458.1102 ([M+H]$^+$), found 458.1097.

IR (thin film, ν_{max} /cm^{-1}) 1516, 1423, 1362, 1254, 1215, 1178, 1035, 969, 817, 743.

(2*R**,3*R**)-2-(4-Methoxyphenyl)-3-methyl-3-(2-((1-phenyl-1H-tetrazol-5-yl)sulfonyl)ethyl)azetidine-1-sulfonyl fluoride (4c) and (2*R**,3*S**)-2-(4-methoxyphenyl)-3-methyl-3-(2-((1-phenyl-1H-tetrazol-5-yl)sulfonyl)ethyl)azetidine-1-sulfonyl fluoride (4c')

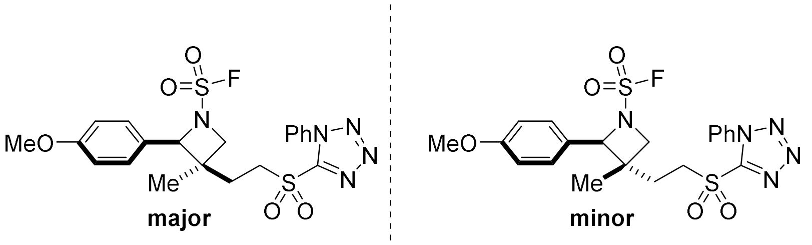

Prepared according to **GP1** using (4-methoxybenzylidene)sulfamoyl fluoride (**1**) (43.4 mg, 0.20 mmol, 1.0 eq.), 3-F-TX (4.6 mg, 0.020 mmol, 0.10 eq.) and 5-((3-methylbut-3-en-1-yl)sulfonyl)-1-phenyl-1H-tetrazole (**O-31**) (167 mg, 0.60 mmol, 3.0 eq.) in $(CH_2Cl)_2$ (1.0 ml, 0.20 M). ^{19}F NMR analysis of the crude reaction mixture revealed a diastereomer ratio of 1.1:1. Purification by flash column chromatography (0 – 20% EtOAc in pentane) afforded (2*R**,3*R**)-2-(4-methoxyphenyl)-3-methyl-3-(2-((1-phenyl-1H-tetrazol-5-yl)sulfonyl)ethyl)azetidine-1-sulfonyl fluoride (**4c**) as a colourless oil (32.6 mg, 32%) and (2*R**,3*S**)-2-(4-methoxyphenyl)-3-methyl-3-(2-((1-phenyl-1H-tetrazol-5-yl)sulfonyl)ethyl)azetidine-1-sulfonyl fluoride (**4c'**) as a white solid (39.6 mg, 30%).

Note: 107 mg (0.38 mmol) of 5-((3-methylbut-3-en-1-yl)sulfonyl)-1-phenyl-1H-tetrazole was recovered after the reaction.

major

R_f (20% EtOAc in pentane) = 0.10.

^{1}H NMR (400 MHz, CDCl$_3$) δ 7.65 – 7.55 (m, 5H), 7.29 – 7.25 (m, 2H), 6.94 – 6.90 (m, 2H), 5.16 (s, 1H), 3.98 (dd, *J* = 8.2, 1.8 Hz, 1H), 3.84 (d, *J* = 8.2 Hz, 1H), 3.80 (s, 3H), 3.42 (ddd, *J* = 14.4, 11.9, 4.2 Hz, 1H), 3.04 (ddd, *J* = 14.4, 11.9, 5.0 Hz, 1H), 2.06 (ddd, *J* = 14.0, 11.9, 4.2 Hz, 1H), 1.93 (ddd, *J* = 14.0, 11.9, 5.0 Hz, 1H), 1.48 (s, 3H).

^{13}C NMR (101 MHz, CDCl$_3$) δ 160.4, 153.2, 132.9, 131.7, 129.9, 127.6, 125.7, 125.1, 114.7, 76.0, 60.7, 55.5, 51.6, 39.4, 27.9, 24.1.

^{19}F NMR (377 MHz, CDCl$_3$) δ 30.55(s).

HRMS (ESI) *m/z*; $C_{20}H_{23}FN_5O_5S_2$ requires 496.1119 ([M+H]$^+$), found 496.1116.

IR (thin film, v_{max} /cm^{-1}) 1420, 1311, 1243,1212, 1159, 1082, 752, 689.

minor

R_f (20% EtOAc in pentane) = 0.17.

^{1}H NMR (400 MHz, CDCl$_3$) δ 7.73 – 7.57 (m, 5H), 7.31 – 7.27 (m, 2H), 6.97 – 6.92 (m, 2H), 5.25 (s, 1H), 4.06 (dd, *J* = 8.0, 1.9 Hz, 1H), 3.82 (s, 3H), 3.81 – 3.70 (m, 1H), 3.72 (d, *J* = 7.9 Hz, 1H), 3.66 (ddd, *J* = 14.5, 10.9, 5.7 Hz, 1H), 2.45 – 2.30 (m, 2H), 0.98 (s, 3H).

^{13}C NMR (126 MHz, CDCl$_3$) δ 160.2, 153.2, 133.0, 131.8, 130.0, 127.9, 126.2, 125.0, 114.4, 75.2, 60.7, 55.5, 51.7, 39.3, 32.8, 19.7.

^{19}F NMR (377 MHz, CDCl$_3$) δ 31.09(s).

HRMS (ESI) *m/z*; $C_{20}H_{23}FN_5O_5S_2$ requires 496.1119 ([M+H]$^+$), found 496.1118.

IR (thin film, v_{max} /cm^{-1}) 1516; 1421, 1343, 1253, 1189, 1016, 689, 650.

m.p. 126 – 130 °C.

(2*R**,3*S**)-2-(4-Methoxyphenyl)-3-methyl-3-(3-oxobutyl)azetidine-1-sulfonyl fluoride (4d) and (2*R**,3*R**)-2-(4-methoxyphenyl)-3-methyl-3-(3-oxobutyl)azetidine-1-sulfonyl fluoride (4d')

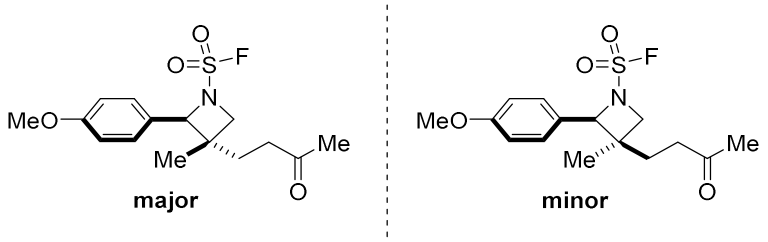

Prepared according to **GP1** using (4-methoxybenzylidene)sulfamoyl fluoride (**1**) (43.4 mg, 0.20 mmol, 1.0 eq.), 3-F-TX (4.6 mg, 0.020 mmol, 0.10 eq.) and 5-methylhex-5-en-2-one (258 µl, 2.0 mmol, 10 eq.) in $(CH_2Cl)_2$ (1.0 ml, 0.20 M). ^{19}F NMR analysis of the crude reaction mixture revealed a diastereomer ratio of 1.1:1. Purification by flash column chromatography (0 – 40% Et_2O in pentane) afforded (2*R**,3*S**)-2-(4-methoxyphenyl)-3-methyl-3-(3-oxobutyl)azetidine-1-sulfonyl fluoride (**4d**) as a colourless oil (25.4 mg, 39%) and a 10:1 mixture of ((2*R**,3*R**)-2-(4-methoxyphenyl)-3-methyl-3-(3-oxobutyl)azetidine-1-sulfonyl fluoride (**4d'**) (2*R**,3*S**)-2-(4-methoxyphenyl)-3-methyl-3-(3-oxobutyl)azetidine-1-sulfonyl fluoride (**4d**) as a colourless oil (30.1 mg, 46%).

major

R$_f$ (50% Et_2O in pentane) = 0.41.

^{1}H NMR (700 MHz, $CDCl_3$) δ 7.25 – 7.22 (m, 2H), 6.94 – 6.89 (m, 2H), 5.12 (s, 1H), 3.97 (dd, *J* = 7.7, 1.6 Hz, 1H), 3.81 (s, 3H), 3.63 (d, *J* = 7.8 Hz, 1H), 2.49 (ddd, *J* = 17.7, 9.9, 5.6 Hz, 1H), 2.41 (ddd, *J* = 17.6, 9.9, 5.7 Hz, 1H), 2.19 (s, 3H), 2.00 (ddd, *J* = 14.3, 10.0, 5.7 Hz, 1H), 1.94 (ddd, *J* = 14.3, 9.9, 5.7 Hz, 1H), 0.84 (s, 3H).

^{13}C NMR (176 MHz, $CDCl_3$) δ 207.2, 159.8, 127.8, 127.0, 114.1, 75.5, 61.2, 55.4, 39.7, 38.2, 33.9, 30.2, 20.0.

^{19}F NMR (377 MHz, $CDCl_3$) δ 30.40 (s).

HRMS (ESI) *m/z*; $C_{15}H_{20}FNO_4SNa$ requires 352.0989 ($[M+Na]^+$), found 352.0996.

IR (thin film, ν_{max} /cm^{-1}) 2968, 1716, 1615, 1515, 1420, 1305, 1252, 1211, 1177, 1085, 1033, 912, 817, 741, 641.

minor

R$_f$ (50% Et_2O in pentane) = 0.29.

^{1}H NMR (700 MHz, $CDCl_3$) δ 7.26 (d, *J* = 2.7 Hz, 2H), 6.93 – 6.90 (m, 2H), 5.10 (d, *J* = 1.9 Hz, 1H), 3.88 (dd, *J* = 8.0, 1.7 Hz, 1H), 3.81 (s, 3H), 3.73 (d, *J* = 7.9 Hz, 1H), 2.09 (ddd, *J* = 17.2, 10.5, 5.2 Hz, 1H), 1.96 (s, 3H), 1.95 – 1.90 (m, 1H), 1.67 (ddd, *J* = 14.6, 10.5, 5.2 Hz, 1H), 1.46 (ddd, *J* = 15.0, 10.4, 5.1 Hz, 1H), 1.33 (s, 3H).

^{13}C NMR (**176 MHz, $CDCl_3$)** δ 207.4, 160.0, 127.8, 126.6, 114.2, 76.6, 61.1, 55.4, 39.6, 37.9, 30.0, 28.8, 24.5

^{19}F NMR (377 MHz, CDCl3) δ 30.17 (s).

HRMS (ESI) *m/z*; $C_{15}H_{21}FNO_4S$ requires 330.1170 ($[M+H]^+$), found 330.1173.

IR (thin film, ν_{max} /cm^{-1}) 2964, 1717, 1615, 1516, 1461, 1422, 1358, 1305, 1254, 1213, 1177, 1085, 1034, 845, 816, 776, 742, 639.

2-((2*R**,3*S**)-1-(Fluorosulfonyl)-2-(4-methoxyphenyl)-3-methylazetidin-3-yl)ethyl but-3-enoate (4e) and 2-((2*R**,3*R**)-1-(Fluorosulfonyl)-2-(4-methoxyphenyl)-3-methylazetidin-3-yl)ethyl but-3-enoate (4e')

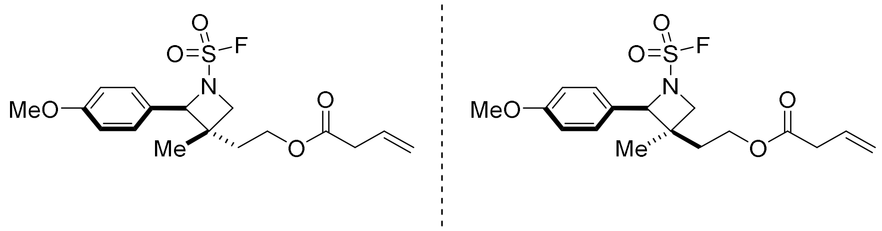

Prepared according to **GP1** using (4-methoxybenzylidene)sulfamoyl fluoride (**1**) (43.4 mg, 0.20 mmol, 1.0 eq.), 3-F-TX (4.6 mg, 0.020 mmol, 0.10 eq.) and 3-methylbut-3-en-1-yl but-3-enoate (**O-32**) (92.5 mg, 0.60 mmol, 3.0 eq.) in $(CH_2Cl)_2$ (1.0 ml, 0.20 M). ^{19}F NMR analysis of the crude reaction mixture revealed a diastereomer ratio of 1:1. Purification by flash column chromatography (0 – 20% EtOAc in pentane) afforded a mixture of 2-((2*R**,3*S**)-1-(fluorosulfonyl)-2-(4-methoxyphenyl)-3-

methylazetidin-3-yl)ethyl (**4e**) and but-3-enoate2-((2*R**,3*R**)-1-(fluorosulfonyl)-2-(4-methoxyphenyl)-3-methylazetidin-3-yl)ethyl but-3-enoate (**4e'**) as a colourless oil (45.9 mg, 62%).

mixture

R$_f$ (20% Et$_2$O in pentane) = 0.16.

^{1}H NMR (400 MHz, CDCl$_3$) δ 7.27 – 7.21 (m, 4H), 6.94 – 6.89 (m, 4H), 5.95 – 5.77 (m, 2H), 5.22 – 5.10 (m, 6H), 4.23 (ddd, *J* = 11.6, 6.6, 5.0 Hz, 1H), 4.13 (ddd, *J* = 11.5, 8.6, 5.9 Hz, 1H), 4.07 (dd, *J* = 7.9, 1.9 Hz, 1H), 3.96 – 3.89 (m, 4H), 3.81 (s, 3H), 3.80 (s, 3H), 3.66 (dd, *J* = 7.8, 1.3 Hz, 1H), 3.10 (dt, *J* = 7.0, 1.4 Hz, 2H), 3.02 (dt, *J* = 7.0, 1.4 Hz, 2H), 2.14 (ddd, *J* = 14.9, 8.6, 6.6 Hz, 1H), 1.95 (dt, *J* = 14.3, 5.5 Hz, 1H), 1.83 – 1.74 (m, 1H), 1.41 (s, 3H), 1.20 (dt, *J* = 14.5, 5.8 Hz, 1H), 0.90 (s, 3H).

^{13}C NMR (101 MHz, CDCl$_3$) δ 171.3, 171.3, 159.9, 159.8, 129.9, 129.8, 127.7, 127.6, 126.7, 126.5, 119.2, 119.0, 114.2, 114.1, 77.4, 75.7, 61.8, 61.2, 60.8, 55.4, 39.2, 39.1, 39.0, 38.9, 38.7, 33.7, 24.4, 19.7.

^{19}F NMR (377 MHz, CDCl$_3$) δ 30.44(s), 30.08(s).

HRMS (ESI) *m/z*; $C_{17}H_{22}FNO_5SNa$ requires 394.1095 ([M+Na]$^+$), found 394.1091.

IR (thin film, ν_{max} /cm^{-1}) 1738, 1615, 1516, 1253, 1214, 1088, 1034, 993, 842, 740, 640.

(1*R**,4*r**,6*R**)-6-Cyano-1-(4-methoxyphenyl)-2-azaspiro[3.3]heptane-2-sulfonyl fluoride (4f) and (1*R**,4*s**,6*S**)-6-Cyano-1-(4-methoxyphenyl)-2-azaspiro[3.3]heptane-2-sulfonyl fluoride (4f')

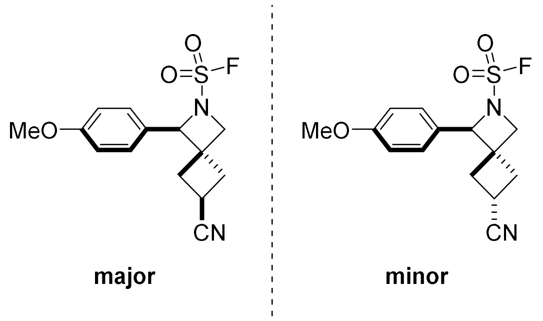

Prepared according to **GP1** using (4-methoxybenzylidene)sulfamoyl fluoride (**1**) (43.4 mg, 0.20 mmol, 1.0 eq.), 3-F-TX (4.6 mg, 0.020 mmol, 0.10 eq.) and 3-methylenecyclobutane-1-carbonitrile (207 µl, 2.0 mmol, 10 eq.) in $(CH_2Cl)_2$ (1.0 ml, 0.20 M). ^{19}F NMR analysis of the crude reaction mixture revealed a diastereomer ratio of 1.1:1. Purification by flash column chromatography (0 – 50% Et$_2$O in pentane) afforded (1*R**,4*r**,6*R**)-6-Cyano-1-(4-methoxyphenyl)-2-azaspiro[3.3]heptane-2-sulfonyl fluoride (**4f**) as a yellow oil (22.5 mg, 37 %) and (1*R**,4*s**,6*S**)-6-Cyano-1-(4-methoxyphenyl)-2-azaspiro[3.3]heptane-2-sulfonyl fluoride (**4f'**) as a yellow oil (19.5 mg, 32%).

major

R$_f$ (40% Et$_2$O in pentane) = 0.26.

^{1}H NMR (600 MHz, CDCl$_3$) δ 7.28 – 7.24 (m, 2H), 6.99 – 6.95 (m, 2H), 5.23 (s, 1H), 4.26 (d, *J* = 8.8 Hz, 1H), 4.09 (d, *J* = 8.8 Hz, 1H), 3.83 (s, 3H), 2.68 – 2.59 (m, 3H), 2.33 – 2.28 (m, 1H), 2.27 – 2.21 (m, 1H).

^{13}C NMR (101 MHz, CDCl$_3$) 160.6, 127.9, 126.3, 121.2, 114.7, 76.1, 61.1, 55.5, 42.2, 36.2, 33.0, 17.2.

^{19}F NMR (377 MHz, CDCl$_3$) δ 30.53 (s).

HRMS (ESI) *m/z* $C_{14}H_{16}FN_2O_3S^+$ requires 311.0860 ([M+H]$^+$), found 311.0870.

IR (thin film, ν_{max} /cm^{-1}) 2927, 2241, 1615, 1517, 1307, 1255, 1213, 1178, 1034, 833, 768, 637.

minor

R$_f$ (40% Et$_2$O in pentane) = 0.15.

^{1}H NMR (600 MHz, CDCl$_3$) δ 7.28 – 7.23 (m, 2H), 7.01 – 6.95 (m, 2H), 5.18 (s, 1H), 4.25 (d, *J* = 8.4 Hz, 1H), 4.11 (d, *J* = 8.4 Hz, 1H), 3.84 (s, 3H), 2.99 (p, *J* = 8.8 Hz, 1H), 2.65 (ddd, *J* = 12.7, 8.7, 4.0 Hz, 1H), 2.55 (dd, *J* = 12.7, 8.9 Hz, 1H), 2.31 (ddd, *J* = 12.7, 8.6, 4.1 Hz, 1H), 2.12 (dd, *J* = 12.7, 9.0 Hz, 1H).

^{13}C NMR (151 MHz, CDCl$_3$) δ 160.6, 128.2, 125.9, 120.7, 114.8, 75.7, 61.9, 55.5, 42.0, 35.9, 33.6, 16.9.

^{19}F NMR (377 MHz, CDCl$_3$) δ 30.86(s).

HRMS (ESI) *m/z* $C_{14}H_{16}FN_2O_3S$ requires 311.0860 ([M+H]$^+$), found 311.0864.

IR (thin film, ν_{max} /cm^{-1}) 2942, 2847, 2240, 1614, 1517, 1422, 1308, 1255, 1212, 1178, 1032, 839, 755, 661.

(1*R**,4*r**,6*R**)-6-Cyano-1-(4-bromophenyl)-2-azaspiro[3.3]heptane-2-sulfonyl fluoride (4g) and (1*R**,4*s**,6*S**)-6-Cyano-1-(4-bromophenyl)-2-azaspiro[3.3]heptane-2-sulfonyl fluoride (4g')

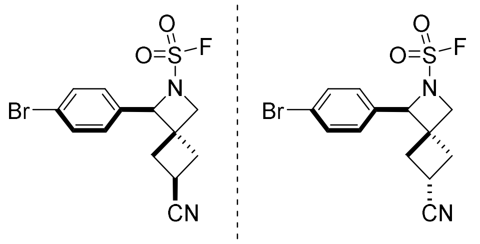

Prepared according to **GP1** using (4-bromobenzylidene)sulfamoyl fluoride (**I-9**) (53.2 mg, 0.20 mmol, 1.0 eq.), 3-F-TX (4.6 mg, 0.020 mmol, 0.10 eq.) and 3-methylenecyclobutane-1-carbonitrile (207 µl, 2.0 mmol, 10 eq.) in $(CH_2Cl)_2$ (1.0 ml, 0.20 M). ^{19}F NMR analysis of the crude reaction mixture revealed a diastereomer ratio of 1.1:1. Purification by flash column chromatography (40 – 50% Et_2O in pentane) afforded (1*R**,4*r**,6*R**)-6-cyano-1-(4-bromophenyl)-2-azaspiro[3.3]heptane-2-sulfonyl fluoride (**4g**) as a colourless oil (27.0 mg, 27 %) and and (1*R**,4*s**,6*S**)-6-cyano-1-(4-bromophenyl)-2-azaspiro[3.3]heptane-2-sulfonyl fluoride (**4g'**) as a colourless oil (16.5 mg, 23%).

major

R$_f$ (40% Et$_2$O in pentane) = 0.20.

^{1}H NMR (600 MHz, CDCl$_3$) δ 7.62 – 7.58 (m, 2H), 7.24 – 7.21 (m, 2H), 5.25 (s, 1H), 4.31 (dd, *J* = 8.8, 1.8 Hz, 1H), 4.13 (d, *J* = 8.7 Hz, 1H), 2.71 – 2.63 (m, 3H), 2.32 (ddd, *J* = 13.3, 4.7, 2.4 Hz, 1H), 2.18 (ddd, *J* = 12.9, 4.9, 4.1 Hz, 1H).

^{13}C NMR (151 MHz, CDCl$_3$) δ 133.3, 132.7, 128.0, 123.8, 121.0, 75.5, 61.4, 42.0, 36.2, 33.0, 17.3.

^{19}F NMR (377 MHz, CDCl$_3$) δ 30.89 (s).

HRMS (ESI) *m/z* $C_{13}H_{11}{}^{79}BrFN_2O_2S$ requires 356.9714 ([M-H]$^-$), found 356.9711.

minor

R$_f$ (40% Et$_2$O in pentane) = 0.12.

^{1}H NMR (600 MHz, CDCl$_3$) δ 7.63 – 7.60 (m, 2H), 7.25 – 7.22 (m, 2H), 5.21 (s, 1H), 4.28 (dd, *J* = 8.3, 2.0 Hz, 1H), 4.13 (d, *J* = 8.4 Hz, 1H), 3.01 (p, *J* = 8.7 Hz, 1H), 2.69 (ddd, *J* = 12.8, 8.7, 4.1 Hz, 1H), 2.59 (dd, *J* = 12.8, 8.7 Hz, 1H), 2.31 (ddd, *J* = 13.1, 8.7, 3.8 Hz, 1H), 2.07 (dd, *J* = 12.8, 8.8 Hz, 1H).

^{13}C NMR (151 MHz, CDCl$_3$) δ 132.9, 132.7, 128.3, 123.8, 120.5, 75.1, 62.1, 41.6, 36.0, 33.6, 16.9.

^{19}F NMR (377 MHz, CDCl$_3$) δ 31.22(s).

No relevant ions were detected by ESI, APCI and EI.

(2*R**,*3r**,3a'*R**,6a'*S**)-2-(4-Methoxyphenyl)tetrahydro-1'H,3'H-dispiro[azetidine-3,2'-pentalene-5',2''-[1,3]dioxolane]-1-sulfonyl fluoride (4h) and (2*S**,*3r**,3a'*R**,6a'*S**)-2-(4-Methoxyphenyl)tetrahydro-1'H,3'H-dispiro[azetidine-3,2'-pentalene-5',2''-[1,3]dioxolane]-1-sulfonyl fluoride (4h')

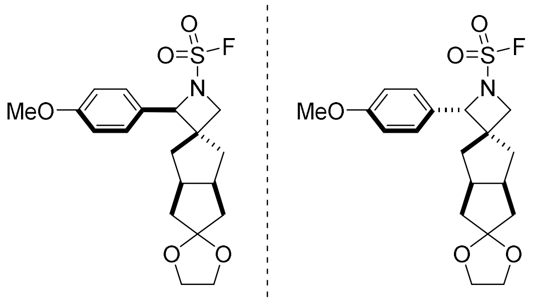

Prepared according to **GP1** using (4-methoxybenzylidene)sulfamoyl fluoride (**1**) (43.4 mg, 0.20 mmol, 1.0 eq.), 3-F-TX (4.6 mg, 0.020 mmol, 0.10 eq.) and 5-methylenehexahydro-1H-spiro[pentalene-2,2'-[1,3]dioxolane] (**O-34**) (361 mg, 2.0 mmol, 10 eq.) in $(CH_2Cl)_2$ (1.0 ml, 0.20 M). ^{19}F NMR analysis of the crude reaction mixture revealed a diastereomer ratio of 1.1:1. Purification by flash column chromatography (10 – 30% Et$_2$O in pentane) afforded an inseparable mixture of (2*R**,*3r**,3a'*R**,6a'*S**)-2-(4-methoxyphenyl)tetrahydro-1'H,3'H-dispiro[azetidine-3,2'-pentalene-5',2''-[1,3]dioxolane]-1-sulfonyl fluoride (**4h**) and (2*S**,*3r**,3a'*R**,6a'*S**)-2-(4-methoxyphenyl)tetrahydro-1'H,3'H-dispiro[azetidine-3,2'-pentalene-5',2''-[1,3]dioxolane]-1-sulfonyl fluoride (**4h'**) as a white solid (70.1 mg, 88%).

mixture

R$_f$ (30% Et$_2$O in pentane) = 0.09.

^{1}H NMR (600 MHz, CDCl$_3$) δ 7.33 – 7.27 (m, 2H), 7.29 – 7.26 (m, 2H), 6.97 – 6.89 (m, 4H), 5.33 (s, 1H), 5.14 (d, *J* = 1.7 Hz, 1H), 4.19 (dd, *J* = 8.1, 1.7 Hz, 1H), 4.00 (d, *J* = 8.0 Hz, 1H), 3.94 (d, *J* = 7.8 Hz, 1H), 3.91 – 3.87 (m, 1H), 3.87 – 3.80 (m, 10H), 2.57 – 2.43 (m, 3H), 2.22 (dtd, *J* = 12.8, 7.9, 1.9 Hz, 2H), 2.00 – 1.77 (m, 8H), 1.70 (dd, *J* = 12.8, 8.9 Hz, 1H), 1.58 (ddd, *J* = 13.5, 4.8, 1.6 Hz, 1H), 1.54 (ddd, *J* = 13.4, 5.1, 1.5 Hz, 1H), 1.42 (ddt, *J* = 12.4, 4.9, 2.8 Hz, 3H), 1.01 – 0.94 (m, 1H).

^{13}C NMR (151 MHz, CDCl$_3$) δ 159.9, 159.8, 128.0, 127.8, 127.7, 127.6, 119.1, 118.8, 114.1, 114.1, 77.1, 73.3, 64.6, 64.6, 64.1, 64.1, 63.4, 58.8, 55.4, 49.9, 49.7, 45.3, 44.1, 41.5, 41.1, 41.1, 41.0, 40.2, 39.5, 39.0, 38.7, 38.0, 37.7.

^{19}F NMR (565 MHz, CDCl$_3$) δ 30.17 (s), 30.07 (s).

HRMS (ESI) *m/z* $C_{19}H_{25}FNO_5S^+$ requires 398.1432 ($[M+H]^+$), found 398.1432.

IR (thin film, v_{max} /cm^{-1}) 1614, 1516, 1318, 1305, 1252, 1211, 1176, 1120, 1032, 836, 741.

1-(4-Methoxyphenyl)-2-azaspiro[3.5]nonane-2-sulfonyl fluoride (4i)

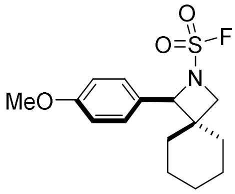

Prepared according to **GP1** using (4-methoxybenzylidene)sulfamoyl fluoride (**1**) (43.4 mg, 0.20 mmol, 1.0 eq.), ITX (5.1 mg, 0.020 mmol, 0.10 eq.) and methylene cyclohexene (240 µl, 2.0 mmol, 10 eq.) in $(CH_2Cl)_2$ (1.0 ml, 0.20 M). Purification by flash column chromatography (0 – 10% Et_2O in pentane) afforded 1-(4-methoxyphenyl)-2-azaspiro[3.5]nonane-2-sulfonyl fluoride (**4i**) as a yellow oil (50.0 mg, 80%).

R_f (10% Et_2O in pentane) = 0.33.

^{1}H NMR (600 MHz, $CDCl_3$) δ 7.28 – 7.22 (m, 2H), 6.94 – 6.87 (m, 2H), 5.05 (s, 1H), 3.92 (d, J = 8.0 Hz, 1H), 3.81 (s, 3H), 3.79 (d, J = 7.9 Hz, 1H), 1.94 – 1.88 (m, 1H), 1.70 – 1.60 (m, 2H), 1.58 – 1.45 (m, 2H,), 1.41 – 1.33 (m, 1H), 1.34 – 1.25 (m, 1H), 1.19 – 1.06 (m, 2H), 0.93 – 0.85 (m, 1H).

^{13}C NMR (101 MHz, $CDCl_3$) δ 159.7, 127.8, 127.0, 113.9, 76.6, 60.7, 55.4, 41.2, 37.5, 31.7, 25.2, 23.0, 22.0.

^{19}F NMR (377 MHz, $CDCl_3$) δ 30.18 (s).

HRMS (ESI) *m/z* $C_{15}H_{21}FNO_3S$ requires 314.1221 ([M+H]$^+$), found 321.1226.

IR (thin film, v_{max} /cm^{-1}) 2933, 1615, 1515, 1450, 1420, 1212, 1176, 1033, 734.

1-(4-Methoxyphenyl)-7-(2,2,2-trifluoroacetyl)-2,7-diazaspiro[3.5]nonane-2-sulfonyl fluoride (4j)

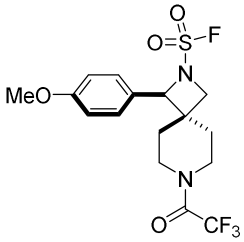

Prepared according to **GP1** using (4-methoxybenzylidene)sulfamoyl fluoride (**1**) (43.4 mg, 0.20 mmol, 1.0 eq.), 3-F-TX (4.6 mg, 0.020 mmol, 0.10 eq.) and 2,2,2-trifluoro-1-(4-methylenepiperidin-1-yl)ethan-1-one (**O-35**) (386 mg, 2.0 mmol, 10 eq.) in $(CH_2Cl)_2$ (1.0 ml, 0.20 M). Purification by flash column chromatography (0 – 40% EtOAc in pet ether) and **Work Up A** afforded 1-(4-methoxyphenyl)-7-(2,2,2-trifluoroacetyl)-2,7-diazaspiro[3.5]nonane-2-sulfonyl fluoride (**4j**) as a colourless oil (58.4 mg, 71%).

1.1:1 mixture of rotamers

R_f (30% Et_2O in pentane) = 0.15.

^{1}H NMR (600 MHz, $CDCl_3$) δ 7.29 – 7.24 (m, 4H), 6.95 – 6.92 (m, 4H), 5.17 (d, *J* = 1.6 Hz, 1H), 5.16 (s, 1H), 4.08 (dt, *J* = 13.7, 4.7 Hz, 1H), 4.03 (d, *J* = 8.1 Hz, 1H), 4.02 (d, *J* = 8.1 Hz, 1H), 3.90 (d, *J* = 8.2 Hz, 1H), 3.88 (d, *J* = 8.1 Hz, 1H), 3.82 (s, 3H), 3.81 (s, 3H), 3.80 – 3.78 (m, 1H,), 3.75 (dt, *J* = 13.8, 4.9 Hz, 1H), 3.40 (dt, *J* = 14.4, 4.9 Hz, 1H), 3.30 (ddd, *J* = 13.9, 10.2, 3.2 Hz, 1H), 3.20 (ddd, *J* = 13.6, 10.0, 3.4

Hz, 1H), 3.15 (ddd, J = 13.9, 10.0, 3.2 Hz, 1H), 2.99 (ddd, J = 13.8, 10.3, 3.4 Hz, 1H), 2.07 – 1.99 (m, 2H), 1.95 – 1.88 (m, 2H), 1.64 (ddd, J = 5.2, 3.3, 1.5 Hz, 1H), 1.62 (ddd, J = 5.1, 3.2, 1.5 Hz, 1H), 1.35 – 1.24 (m, 2H).

^{13}C NMR (151 MHz, $CDCl_3$) δ 160.3, 160.3, 155.6 (q, $^{2}J_{C-F}$ = 35.8 Hz), 155.5 (q, $^{2}J_{C-F}$ = 35.8 Hz), 127.9, 127.9, 125.7, 125.6, 116.5 (q, $^{1}J_{C-F}$ = 288.0 Hz), 116.4 (q, $^{1}J_{C-F}$ = 288.0 Hz), 114.5, 75.6, 75.6, 59.7, 59.6, 55.5, 42.5 (q, $^{3}J_{C-F}$ = 3.6 Hz), 42.1 (q, $^{3}J_{C-F}$ = 3.6 Hz), 40.4, 39.9, 39.6, 39.5, 36.7, 35.8, 31.7, 30.7.

Note: 2 Carbon environments not observed due to signal overlap.

^{19}F NMR (377 MHz, $CDCl_3$) δ 31.32(s), 31.20(s), -68.96(s), -69.00(s).

HRMS (ESI) *m/z*; $C_{16}H_{18}F_4N_2O_4SNa$ requires 433.0816 ($[M+Na]^+$), found 433.0818.

IR (thin film, ν_{max} /cm^{-1}) 2932, 1694, 1614, 1516, 1467, 1304, 1255, 1214, 1129, 1034, 837, 821, 774, 738.

1-(4-Methoxyphenyl)-7-thia-2-azaspiro[3.5]nonane-2-sulfonyl fluoride 7,7-dioxide (4k)

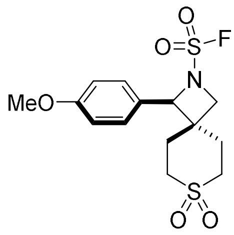

Prepared according to **GP1** using (4-methoxybenzylidene)sulfamoyl fluoride (**1**) (43.4 mg, 0.20 mmol, 1.0 eq.), 3-F-TX (4.6 mg, 0.020 mmol, 0.10 eq.) and 4-methylenetetrahydro-2H-thiopyran 1,1-dioxide (**O-37**) (292 mg, 2.0 mmol, 10 eq.) in $(CH_2Cl)_2$ (1.0 ml, 0.20 M). Purification by flash column chromatography (0 – 40% EtOAc in pet ether) afforded 1-(4-methoxyphenyl)-7-thia-2-azaspiro[3.5]nonane-2-sulfonyl fluoride 7,7-dioxide (**4k**) as a white solid (44.6 mg, 61%).

Note: 241 mg (1.7 mmol) of 4-methylenetetrahydro-2H-thiopyran 1,1-dioxide was recovered after the reaction.

R_f (40% EtOAc in pet ether) = 0.34.

^{1}H NMR (700 MHz, DMSO-d6) δ 7.41 – 7.35 (m, 2H), 7.03 – 6.98 (m, 2H), 5.46 (s, 1H), 4.18 (d, J = 8.5 Hz, 1H), 4.09 (d, J = 8.5 Hz, 1H), 3.77 (s, 3H), 3.24 (ddd, J = 15.0, 11.5, 3.5 Hz, 1H), 3.13 – 3.04 (m, 2H), 2.65 (m, 1H), 2.40 (ddd, J = 14.3, 5.9, 3.1 Hz, 1H), 2.22 (ddd, J = 14.8, 11.6, 3.5 Hz, 1H), 1.93 (ddd, J = 14.4, 6.0, 3.0 Hz, 1H), 1.33 (m, 1H).

^{13}C NMR (176 MHz, DMSO-d6) δ 159.5, 127.9, 126.3, 114.0, 73.6, 58.8, 55.2, 47.1, 46.3, 38.1, 33.7, 29.2.

^{19}F NMR (377 MHz, DMSO-d6) δ 32.89(s).

HRMS (ESI) *m/z*; $C_{14}H_{18}FNO_5S_2Na$ requires 386.0503 ($[M+Na]^+$), found 386.0503.

IR (neat, ν_{max} /cm^{-1}) 2963, 1513, 1295, 1263, 1108, 1024, 812, 773, 753, 608.

m.p. 186 °C.

(1R*,5S*,7S*)-7-(4-Methoxyphenyl)-6-azabicyclo[3.2.0]heptane-6-sulfonyl fluoride (4l) and (1R*,5S*,7R*)-7-(4-Methoxyphenyl)-6-azabicyclo[3.2.0]heptane-6-sulfonyl fluoride (4l')

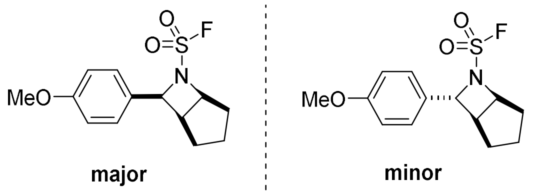

Prepared according to **GP1** using (4-methoxybenzylidene)sulfamoyl fluoride (**1**) (43.4 mg, 0.20 mmol, 1.0 eq.), ITX (5.1 mg, 0.020 mmol, 0.10 eq.) and cyclopentene (177 µl, 2.0 mmol, 10 eq.) in $(CH_2Cl)_2$ (1.0 ml, 0.20 M). ^{19}F NMR analysis of the crude reaction mixture revealed a diastereomer ratio of 5.5:1. Purification by flash column chromatography (0 – 6% Et_2O in pentane) afforded (1R*,5S*,7S*)-7-(4-methoxyphenyl)-6-azabicyclo[3.2.0]heptane-6-sulfonyl fluoride (**4l**) as a colourless oil (24.5 mg, 43%) and a 3:1 mixture of (1R*,5S*,7R*)-7-(4-methoxyphenyl)-6-azabicyclo[3.2.0]heptane-6-sulfonyl fluoride (**4l'**) and (1R*,5S*,7S*)-7-(4-methoxyphenyl)-6-azabicyclo[3.2.0]heptane-6-sulfonyl fluoride (**4l**) as a colourless oil (13.3 mg, 23%).

major

R_f (5% Et_2O in pentane) = 0.20;

^{1}H NMR (600 MHz, $CDCl_3$) δ 7.22 – 7.16 (m, 2H), 6.94 – 6.89 (m, 2H), 5.56 (dd, J = 8.7, 2.2 Hz, 1H), 4.94 – 4.91 (m, 1H), 3.81 (s, 3H), 3.21 (td, J = 9.1, 6.4 Hz, 1H), 2.16 (dd, J = 14.1, 6.6 Hz, 1H), 1.84 – 1.69 (m, 2H), 1.48 (dddd, J = 14.1, 12.0, 7.1, 4.4 Hz, 1H), 1.41 – 1.34 (m, 2H);

^{13}C NMR (151 MHz, $CDCl_3$) δ 159.3, 127.1, 127.0, 114.1, 70.0, 67.2, 55.4, 40.1, 32.8, 25.9, 24.8;

^{19}F NMR (565 MHz, $CDCl_3$) δ 31.52(s);

HRMS (ESI) m/z $C_{13}H_{16}FNO_3SNa^+$ requires 308.0727 ($[M+Na]^+$), found 308.0741;

IR (thin film, ν_{max} /cm^{-1}) 2961, 2841, 1615, 1515, 1419, 1304, 1253, 1206, 1176, 1095, 1050, 1032, 1000, 912, 843, 765, 748, 695.

minor

R_f (5% Et_2O in pentane) = 0.18;

^{1}H NMR (600 MHz, $CDCl_3$) δ 7.38 – 7.35 (m, 2H), 6.96 – 6.94 (m, 2H), 4.99 (q, J = 4.9 Hz, 1H,), 4.81 (t, J = 3.8 Hz, 1H), 3.84 (s, 3H), 2.97 (td, J = 6.6, 4.3 Hz, 1H), 2.50 (dd, J = 14.8, 5.9 Hz, 1H), 2.20 – 2.10 (m, 1H), 2.09 (dq, J = 13.7, 7.3 Hz, 1H), 1.96 (dd, J = 13.6, 6.2 Hz, 1H), 1.66 – 1.59 (m, 2H).

^{13}C NMR (151 MHz, $CDCl_3$) δ 160.1, 128.1, 127.0, 114.4, 71.9, 69.9 (d, J = 2.4 Hz), 55.5, 45.2, 31.4, 30.5, 23.9.

Note: Coupling between Carbon and Fluorine observed.

^{19}F NMR (565 MHz, $CDCl_3$) δ 50.56(s);

HRMS (ESI) m/z $C_{13}H_{17}FNO_3S^+$ requires 286.0908 ($[M+H]^+$), found 286.0911;

IR (thin film, ν_{max} /cm^{-1}) 2918, 2850, 1614, 1516, 1415, 1307, 1252, 1210, 1179, 1060, 1034, 909, 845, 774, 742, 643, 628.

(1R*,6S*,8S*)-8-(4-Methoxyphenyl)-7-azabicyclo[4.2.0]octane-7-sulfonyl fluoride (4m)

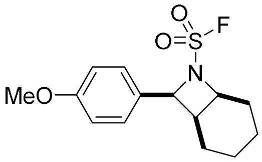

Prepared according to **GP1** using (4-methoxybenzylidene)sulfamoyl fluoride (**1**) (43.4 mg, 0.20 mmol, 1.0 eq.), ITX (5.1 mg, 0.020 mmol, 0.10 eq.) and cyclohexene (203 µl, 2.0 mmol, 10 eq.) in $(CH_2Cl)_2$ (1.0 ml, 0.20 M). Purification by flash column chromatography (0 – 10% Et_2O in pentane) afforded (1R*,6S*,8S*)-8-(4-methoxyphenyl)-7-azabicyclo[4.2.0]octane-7-sulfonyl fluoride (**4m**) as a colourless oil (35.5 mg, 59%).

R_f (10% Et_2O in pentane) = 0.38.

^{1}H NMR (600 MHz, $CDCl_3$) δ 7.27 – 7.22 (m, 2H), 6.96 – 6.87 (m, 2H), 5.46 (d, J = 7.7 Hz, 1H), 4.57 – 4.49 (m, 1H), 3.81 (s, 3H), 2.76 (p, J = 8.4 Hz, 1H), 2.25 – 2.15 (m, 1H), 1.65 – 1.47 (m, 4H), 1.41 – 1.22 (m, 2H), 1.13 – 1.01 (m, 1H).

^{19}F NMR (377 MHz, $CDCl_3$) δ 28.23(s).

^{13}C NMR (101 MHz, $CDCl_3$) δ 159.4, 127.8, 127.2, 113.9, 68.5, 61.3, 55.4, 34.0, 26.5, 21.6, 21.3, 20.1.

HRMS (ESI) m/z $C_{14}H_{18}FNO_3SNa^+$ requires 322.0884 ($[M+Na]^+$), found 322.0889.

IR (thin film, v_{max} /cm^{-1}) 2939, 1615, 1515, 1418, 1304, 1252, 1207, 1176, 1049, 1036, 835, 742, 669.

(2S*,3S*,4S*)-2,3-diethyl-4-(4-methoxyphenyl)azetidine-1-sulfonyl fluoride (Z-4n)

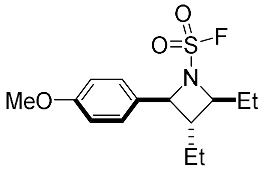

major

Prepared according to **GP1** using (4-methoxybenzylidene)sulfamoyl fluoride (**1**) (43.4 mg, 0.20 mmol, 1.0 eq.), 3-F-TX (4.6 mg, 0.020 mmol, 10 mol%) and Z-hex-3-ene (0.25 ml, 2.0 mmol, 10 eq.) in $(CH_2Cl)_2$ (1.0 ml, 0.20 M). ^{19}F NMR analysis of the crude reaction mixture revealed a diastereomer ratio of 10:1.4:1:0. Purification by flash column chromatography (0 – 2% Et_2O in pentane) afforded 7.6:1 mixture of 2S*,3S*,4S*)-2,3-diethyl-4-(4-methoxyphenyl)azetidine-1-sulfonyl fluoride (**4n**) and (2S*,3R*,4S*)-2,3-diethyl-4-(4-methoxyphenyl)azetidine-1-sulfonyl fluoride (**4n'**) (38.3 mg, 64%) as a colourless oil and (2R*,3R*,4S*)-2,3-diethyl-4-(4-methoxyphenyl)azetidine-1-sulfonyl fluoride as a colourless oil (**4n''**) 6.5 mg, 11%).

Characterisation is given for the major diastereomer only.

major

R_f(4% Et_2O in pentane) = 0.33.

^{1}H NMR (400 MHz, $CDCl_3$) δ 7.37 – 7.32 (m, 2H), 6.93 – 6.89 (m, 2H), 4.77 (dd, J = 7.0, 1.6 Hz, 1H), 3.97 (tdd, J = 7.1, 4.8, 1.5 Hz, 1H), 3.81 (s, 3H), 2.21 (p, J = 7.2 Hz, 1H), 2.05 – 1.93 (m, 1H), 1.93 – 1.81 (m, 1H), 1.78 – 1.65 (m, 2H), 1.02 (t, J = 7.5 Hz, 3H), 0.90 (t, J = 7.4 Hz, 3H).

^{13}C NMR (101 MHz, $CDCl_3$) δ 159.9, 131.1, 128.0, 114.3, 70.7, 69.7, 55.4, 47.7, 28.1, 26.7, 11.2, 9.0.

^{19}F NMR (377 MHz, $CDCl_3$) δ 31.40(s).

HRMS (ESI) *m/z* $C_{14}H_{21}FNO_3S^+$ requires 302.1221 ($[M+H]^+$), found 302.1231.

IR (thin film, v_{max} /cm^{-1}) 2695, 2935, 1615, 1517, 1463, 1421, 1254, 1209, 1037, 840, 740.

(2*S**,3*S**,4*S**)-2,3-diethyl-4-(4-methoxyphenyl)azetidine-1-sulfonyl fluoride (*E*-4n)

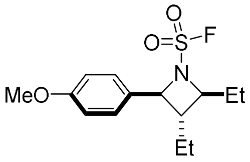

major

Prepared according to **GP1** using (4-methoxybenzylidene)sulfamoyl fluoride (**1**) (43.4 mg, 0.20 mmol, 1.0 eq.), 3-F-TX (4.6 mg, 0.020 mmol, 10 mol%) and E-hex-3-ene (0.25 ml, 2.0 mmol, 10 eq.) in $(CH_2Cl)_2$ (1.0 ml, 0.20 M). ^{19}F NMR analysis of the crude reaction mixture revealed a diastereomer ratio of 7.4:1:2.5:0. Purification by flash column chromatography (0 – 2% Et_2O in pentane) afforded afforded (2*S**,3*S**,4*S**)-2,3-diethyl-4-(4-methoxyphenyl)azetidine-1-sulfonyl fluoride (**4n**) (16.1 mg, 27%) and a 3:2:1 mixture of (2*R**,3*R**,4*S**)-2,3-diethyl-4-(4-methoxyphenyl)azetidine-1-sulfonyl fluoride (**4n''**), (2*S**,3*R**,4*S**)-2,3-diethyl-4-(4-methoxyphenyl)azetidine-1-sulfonyl fluoride and (2*S**,3*S**,4*S**)-2,3-diethyl-4-(4-methoxyphenyl)azetidine-1-sulfonyl fluoride (**4n'**) (13.2 mg, 22%).

Characterisation is given for the major diastereomer only.

Characterisation as before (**4n**).

(1*R**,6*S**,8*R**)-8-(4-Methoxyphenyl)-1-methyl-7-azabicyclo[4.2.0]octane-7-sulfonyl fluoride (4o) and (1*S**,6*R**,8*R**)-8-(4-Methoxyphenyl)-1-methyl-7-azabicyclo[4.2.0]octane-7-sulfonyl fluoride (4o')

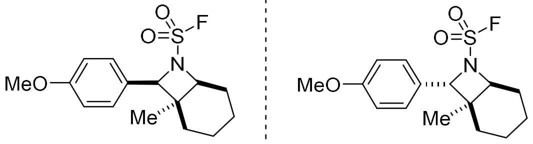

major | minor

Prepared according to **GP1** using (4-methoxybenzylidene)sulfamoyl fluoride (**1**) (43.4 mg, 0.20 mmol, 1.0 eq.), ITX (5.1 mg, 0.020 mmol, 0.10 eq.) and 1-methyl-1-cyclohexene (237 μl, 2.0 mmol, 10 eq.) in $(CH_2Cl)_2$ (1.0 ml, 0.20 M). ^{19}F NMR analysis of the crude reaction mixture revealed a diastereomer ratio of 1.2:1. Purification by flash column chromatography (2.5 – 5% Et_2O in pentane) afforded (1*R**,6*S**,8*R**)-8-(4-methoxyphenyl)-1-methyl-7-azabicyclo[4.2.0]octane-7-sulfonyl fluoride (**4o**) as a colourless oil (13.6 mg, 22%) and (1*S**,6*R**,8*R**)-8-(4-methoxyphenyl)-1-methyl-7-azabicyclo[4.2.0]octane-7-sulfonyl fluoride (**4o'**) as a colourless oil (7.5 mg, 12%).

major

R_f (10% Et_2O in pentane) = 0.40.

^{1}H NMR (600 MHz, $CDCl_3$) δ 7.25 – 7.19 (m, 2H), 6.94 – 6.88 (m, 2H), 4.98 (s, 1H), 4.13 (m, 1H), 3.81 (s, 3H), 2.22 – 2.15 (m, 1H), 1.62 – 1.47 (m, 5H), 1.32 (s, 3H), 1.14 – 1.04 (m, 1H), 0.90 (dt, J = 14.1, 4.3 Hz, 1H).

^{13}C NMR (101 MHz, CDCl$_3$) δ 159.5, 127.6, 127.0, 113.9, 75.5, 68.3, 55.4, 40.1, 29.2, 25.6, 25.0, 20.5, 20.3.

^{19}F NMR (377 MHz, CDCl$_3$) δ 27.63 (s).

HRMS (ESI) *m/z* $C_{15}H_{21}FNO_3S^+$ requires 314.1221 ([M+H]$^+$), found 314.1214.

IR (thin film, ν_{max} /cm^{-1}) 2939, 1615, 1515, 1458, 1304, 1252, 1208, 1177, 1076, 1034, 994, 831, 780, 743.

minor

^{1}H NMR (600 MHz, CDCl$_3$) δ 7.24 – 7.20 (m, 2H), 6.93 – 6.89 (m, 2H), 5.17 (d, *J* = 2.9 Hz, 1H), 4.19 (q, *J* = 5.7 Hz, 1H), 3.81 (s, 3H), 2.25 – 2.16 (m, 1H), 1.97 (ddt, *J* = 14.7, 7.8, 5.4 Hz, 1H), 1.85 – 1.75 (m, 3H), 1.58 – 1.51 (m, 2H), 1.46 – 1.38 (m, 1H), 0.79 (s, 3H).

^{13}C NMR (151 MHz, CDCl$_3$) δ 159.7, 128.1, 127.2, 114.0, 75.6, 70.2 (d, *J* = 2.4 Hz), 55.4, 39.5, 32.9, 24.2, 22.5, 19.0, 17.9.

Note: Coupling between Carbon and Fluorine observed.

^{19}F NMR (377 MHz, CDCl$_3$) δ 52.39 (s).

HRMS (ESI) *m/z* $C_{15}H_{21}FNO_3S^+$ requires 314.1221 ([M+H]$^+$), found 314.1223.

IR (thin film, ν_{max} /cm^{-1}) 2959, 2873, 1615, 1516, 1416, 1254, 1206, 1036, 912, 836, 737, 616.

4-((2*R**,3*S**)-1-(Fluorosulfonyl)-2-(4-methoxyphenyl)azetidin-3-yl)phenyl acetate (4p) and 4-((2*R**,3*R**)-1-(Fluorosulfonyl)-2-(4-methoxyphenyl)azetidin-3-yl)phenyl acetate (4p')

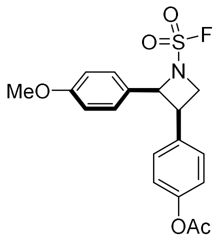

major

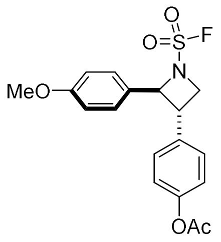

minor

Prepared according to **GP1** using (4-methoxybenzylidene)sulfamoyl fluoride (**1**) (43.4 mg, 0.20 mmol, 1.0 eq.), ITX (5.1 mg, 0.020 mmol, 0.10 eq.) and 4-acetoxy-styrene (295 µl, 2.0 mmol, 10 eq.) in $(CH_2Cl)_2$ (1.0 ml, 0.20 M). ^{19}F NMR analysis of the crude reaction mixture revealed a regioisomer ratio of 17:1, a diastereomer ratio of 2.1:1 and a combined yield of 47%. Purification by flash column chromatography (10 – 18% EtOAc in pentane) afforded 4-((2*R**,3*S**)-1-(fluorosulfonyl)-2-(4-methoxyphenyl)azetidin-3-yl)phenyl acetate (**4p**) with approximately 9% minor regioisomer (30.1 mg, 40%) as a colourless oil and 4-((2*R**,3*R**)-1-(fluorosulfonyl)-2-(4-methoxyphenyl)azetidin-3-yl)phenyl acetate (**4p'**) (9.2 mg, 12%) as a colourless oil.

major

R$_f$ (30% Et$_2$O in pentane) = 0.17.

^{1}H NMR (600 MHz, CDCl$_3$) δ 7.09 – 7.06 (m, 2H), 7.03 – 7.00 (m, 2H), 6.94 – 6.90 (m, 2H), 6.72 – 6.69 (m, 2H), 5.89 (app. dt, *J* = 8.9, 1.1 Hz, 1H), 4.69 (td, *J* = 9.1, 1.8 Hz, 1H), 4.33 (ddd, *J* = 9.0, 4.9, 1.1 Hz, 1H), 4.23 (td, *J* = 9.2, 4.8 Hz, 1H), 3.72 (s, 3H), 2.26 (s, 3H).

^{13}C NMR (151 MHz, CDCl$_3$) δ 169.3, 159.4, 150.0, 133.4, 129.5, 127.9, 126.6, 121.6, 113.7, 71.3, 55.3, 55.1, 40.4, 21.2.

^{19}F NMR (377 MHz, CDCl$_3$) δ 30.27 (s).

HRMS (ESI) *m/z*; $C_{18}H_{19}FNO_5S$ requires 380.0963 ([M+H]$^+$), found 380.0959.

IR (thin film, v_{max} /cm^{-1}) 2962, 2933, 1766, 1613, 1513, 1423, 1370, 1306, 1254, 1212, 1098, 913, 851, 807, 755, 676.

minor

R$_f$ (30% Et$_2$O in pentane) = 0.30.

^{1}H NMR (600 MHz, CDCl$_3$) δ 7.39 – 7.34 (m, 2H), 7.29 – 7.26 (m, 2H), 7.12 – 7.09 (m, 2H), 6.94 – 6.91 (m, 2H), 5.29 (d, *J* = 7.8 Hz, 1H), 4.39 (t, *J* = 8.4 Hz, 1H), 4.31 (td, *J* = 8.2, 1.7 Hz, 1H), 3.85 – 3.80 (m, 4H), 2.31 (s, 3H).

^{13}C NMR (151 MHz, CDCl$_3$) δ 169.5, 160.4, 150.5, 135.4, 129.6, 128.2, 128.1, 122.5, 114.5, 75.6, 55.6, 55.5, 44.3, 21.2.

^{19}F NMR (565 MHz, CDCl$_3$) δ 30.74 (s).

HRMS (ESI) *m/z*; $C_{18}H_{19}FNO_5S$ requires 380.0963 ([M+H]$^+$), found 380.0966.

IR (thin film, v_{max} /cm^{-1}) 1764, 1614, 1516, 1425, 1371, 1255, 1214, 1033, 913, 838, 765, 741, 640.

Tert-butyl (5-((2*S**,3*R**)-1-(fluorosulfonyl)-2-(4-methoxyphenyl)azetidin-3-yl)pyridin-2-yl)carbamate (4q') and Tert-butyl (Tert-butoxycarbonyl)(5-((2*R**,3*R**)-1-(fluorosulfonyl)-2-(4-methoxyphenyl)azetidin-3-yl)pyridin-2-yl)carbamate (4q'')

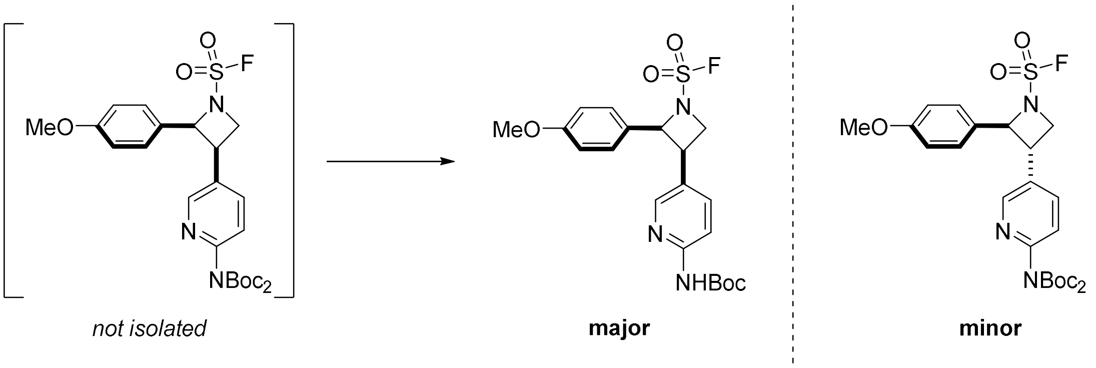

Prepared according to **GP1** using (4-methoxybenzylidene)sulfamoyl fluoride (**1**) (43.4 mg, 0.20 mmol, 1.0 eq.), 3-F-TX (4.6 mg, 0.020 mmol, 0.10 eq.) and tert-butyl (tert-butoxycarbonyl)(5-vinylpyridin-2-yl)carbamate (**O-39**) (640 mg, 2.00 mmol, 10 eq.) in $(CH_2Cl)_2$ (1.0 ml, 0.20 M). ^{19}F NMR analysis of the crude reaction mixture revealed a diastereomer ratio of 1.5:1 and a total yield of both diastereomers of 52%. Purification by flash column chromatography (0 – 20% EtOAc in pentane) afforded tert-butyl (tert-butoxycarbonyl)(5-((2*R**,3*R**)-1-(fluorosulfonyl)-2-(4-methoxyphenyl)azetidin-3-yl)pyridin-2-yl)carbamate (**4q''**) as a white solid (25.3 mg, 24%). Further purification by flash column chromatography (0 – 20% Et_2O/CH_2Cl_2) yielded tert-butyl (5-((2*S**,3*R**)-1-(fluorosulfonyl)-2-(4-methoxyphenyl)azetidin-3-yl)pyridin-2-yl)carbamate (**4q'**) (13.3 mg, 15%) as a white solid.

major (characterised as mono-Boc derivative)

^{1}H NMR (600 MHz, CDCl$_3$) δ 7.94 (s, 1H), 7.81 (s, 1H), 7.76 (s, 1H), 7.37 (d, *J* = 8.6 Hz, 1H), 7.04 – 6.99 (m, 2H), 6.74 – 6.69 (m, 2H), 5.88 (d, *J* = 9.1 Hz, 1H), 4.72 – 4.66 (m, 1H), 4.24 (dd, *J* = 8.9, 4.7 Hz, 1H), 4.15 (td, *J* = 9.2, 4.8 Hz, 1H), 3.71 (s, 3H), 1.51 (s, 9H).

^{13}C NMR (151 MHz, CDCl$_3$) δ 159.5, 152.4, 151.5, 147.6, 138.0, 127.8, 126.2, 126.1, 114.0, 111.8, 81.3, 71.1, 55.3, 55.0, 38.1, 28.4.

^{19}F NMR (565 MHz, CDCl$_3$) δ 30.39.

HRMS (ESI) *m/z*; $C_{20}H_{25}FN_3O_5S$ requires 438.1494 ([M+H]$^+$), found 438.1499.

IR (thin film, v_{max} /cm^{-1}) 2978, 2931, 1725, 1612, 1588, 1538, 1516, 1426, 1369, 1311, 1255, 1213, 1157, 1101, 1062, 1031, 912, 848, 809, 760, 644.

m.p. 142 °C (decomp).

minor

^{1}H NMR (600 MHz, CDCl$_3$) δ 8.34 (d, *J* = 2.5 Hz, 1H), 7.69 (dd, *J* = 8.3, 2.5 Hz, 1H), 7.37 – 7.31 (m, 3H), 6.93 (d, *J* = 8.4 Hz, 2H), 5.27 (d, *J* = 7.8 Hz, 1H), 4.43 (t, *J* = 8.4 Hz, 1H), 4.31 (t, *J* = 8.3 Hz, 1H), 3.85 (q, *J* = 8.5 Hz, 1H), 3.81 (s, 3H), 1.47 (s, 18H).

^{13}C NMR (151 MHz, CDCl$_3$) δ 160.6, 152.4, 151.5, 147.6, 136.4, 131.8, 129.1, 128.0, 121.8, 114.6, 83.7, 75.3, 55.5, 55.1, 42.1, 28.0.

^{19}F NMR (565 MHz, CDCl$_3$) δ 30.86 (s).

HRMS (ESI) *m/z*; $C_{25}H_{33}FN_3O_7S$ requires 538.2018 ([M+H]$^+$), found 538.2025.

IR (thin film, v_{max} /cm^{-1}) 2981, 1794, 1765, 1727, 1613, 1517, 1484, 1425, 1394, 1371, 1341, 1312, 1255, 1211, 1156, 1119, 1023, 913, 838, 734, 643.

(2*R**,3*R**)-2-(4-Methoxyphenyl)-3-methyl-3-phenylazetidine-1-sulfonyl fluoride (4r) and (2*R**,3*S**)-2-(4-Methoxyphenyl)-3-methyl-3-phenylazetidine-1-sulfonyl fluoride (4r’)

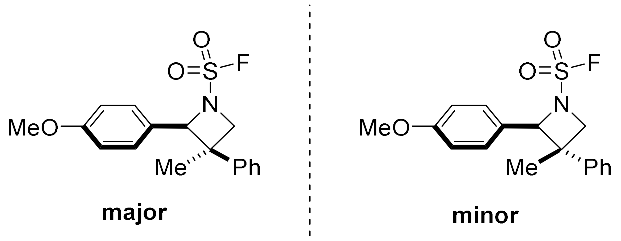

Prepared according to **GP1** using (4-methoxybenzylidene)sulfamoyl fluoride (**1)** (43.4 mg, 0.20 mmol, 1.0 eq.), 3-F-TX (4.6 mg, 0.020 mmol, 0.10 eq.) and 2-phenylpropene (260 µl, 2.00 mmol, 10 eq.) in $(CH_2Cl)_2$ (1.0 ml, 0.20 M). ^{19}F NMR analysis of the crude reaction mixture revealed a diastereomer ratio of 2.1:1. Purification by flash column chromatography (0 – 3% Et_2O in pentane) afforded azetidine (2*R**,3*R**)-2-(4-methoxyphenyl)-3-methyl-3-phenylazetidine-1-sulfonyl fluoride (**4r**) as a white gum (23.1 mg, 34%) and a 9:1 mixture of (2*R**,3*S**)-2-(4-methoxyphenyl)-3-methyl-3-phenylazetidine-1-sulfonyl fluoride (**4r’**) and 2*R**,3*R**)-2-(4-methoxyphenyl)-3-methyl-3-phenylazetidine-1-sulfonyl fluoride (**4r**) as a colourless oil (14.1 mg, 21%).

major

R$_f$ (10% Et_2O in pentane) = 0.39.

^{1}H NMR (600 MHz, $CDCl_3$) δ 7.16 – 7.12 (m, 2H), 7.12 – 7.08 (m, 1H), 6.99 – 6.94 (m, 2H), 6.94 – 6.91 (m, 2H), 6.68 – 6.63 (m, 2H), 5.38 (d, *J* = 2.0 Hz, 1H), 4.69 (dd, *J* = 8.6, 1.8 Hz, 1H), 4.23 (dd, *J* = 8.5, 2.1 Hz, 1H), 3.70 (s, 3H), 1.82 (s, 3H).

^{13}C NMR (151 MHz, $CDCl_3$) δ 159.7, 139.7, 128.5, 128.2, 127.5, 126.9, 113.6, 78.8, 60.9, 55.3, 45.4, 29.3.

Note: 1 Carbon environment not observed due to signal overlap.

^{19}F NMR (377 MHz, CDCl3) δ 31.66 (s).

HRMS (ESI) *m/z*; $C_{17}H_{19}FNO_3S$ requires 336.1064 ([M+H]$^+$), found 336.1069.

IR (thin film, v_{max} /cm^{-1}) 2918, 1614, 1586, 1516, 1424, 1306, 1254, 1215, 1178, 1034, 912, 814, 747, 702, 644.

minor

R$_f$ (10% Et_2O in pentane) = 0.50.

^{1}H NMR (500 MHz, $CDCl_3$) δ 7.44 – 7.37 (m, 4H), 7.33 – 7.29 (m, 1H), 7.28 – 7.23 (m, 2H), 6.99 – 6.96 (m, 2H), 5.59 (s, 1H), 4.58 (dd, *J* = 7.5, 1.9 Hz, 1H), 3.97 (d, *J* = 7.5 Hz, 1H), 3.84 (s, 3H), 1.31 (s, 3H).

^{13}C NMR (126 MHz, $CDCl_3$) δ 160.1, 145.2, 129.2, 128.5, 127.4, 126.6, 125.2, 114.2, 76.4, 61.4, 55.4, 44.1, 24.3.

^{19}F NMR (377 MHz, CDCl3) δ 30.28 (s).

HRMS (ESI) *m/z*; $C_{17}H_{18}FNO_3S$ requires 358.0884 ([M+Na]$^+$), found 358.0901.

IR (thin film, v_{max} /cm^{-1}) 1615, 1518, 1425, 1305, 1254, 1215, 1177, 1034, 821, 757, 737, 702, 658.

(2*R**,3*S**)-3-Hydroxy-2-(4-methoxyphenyl)-3-methylazetidine-1-sulfonyl fluoride (4s) and(2*R**,3*R**)-3-Hydroxy-2-(4-methoxyphenyl)-3-methylazetidine-1-sulfonyl fluoride (4s')

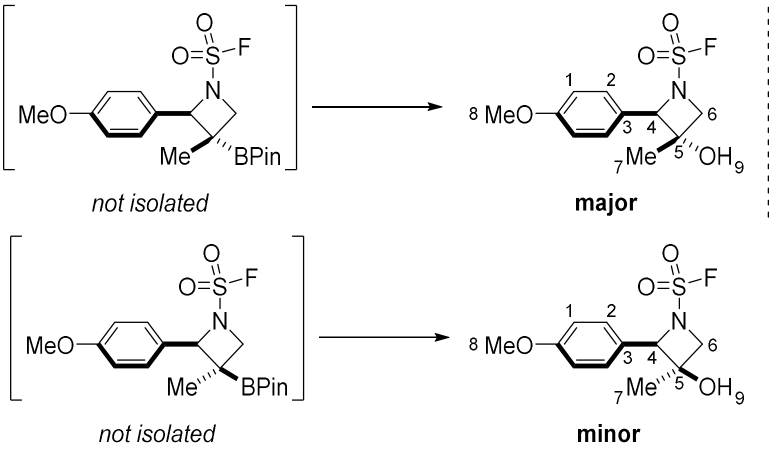

Prepared according to **GP1** using (4-methoxybenzylidene)sulfamoyl fluoride (**1**) (43.4 mg, 0.20 mmol, 1.0 eq.), 3-F-TX (4.6 mg, 0.020 mmol, 0.10 eq.) and isopropenylboronic acid pinacol ester (378 µl, 2.00 mmol, 10 eq.) in $(CH_2Cl)_2$ (1.0 ml, 0.20 M). ^{19}F NMR analysis of the crude reaction mixture revealed a diastereomer ratio of 3.3:1 and a total yield of both diastereomers of 44% for the boronic ester.

Following the photochemical reaction, the reaction mixture was dissolved in THF (9.0 ml) and cooled to 0 °C. 3M NaOH (6 ml) was added followed by dropwise addition of 30% (w/w) H_2O_2 solution (3.0 ml). Following addition reaction mixture was warmed to rt and stirred for 4 h. The reaction mixture was then cooled to 0 °C and quenched with aq. sat. $Na_2S_2O_3$ (caution: exotherm). The reaction mixture was then warmed to rt and extracted thrice with Et_2O, dried over anhydrous $MgSO_4$, filtered and concentrated under reduced pressure. Purification by flash column chromatography (0 – 20% EtOAc in pentane) afforded an inseparable mixture of (2*R**,3*S**)-3-hydroxy-2-(4-methoxyphenyl)-3-methylazetidine-1-sulfonyl fluoride (**4s**) and (2*R**,3*R**)-3-hydroxy-2-(4-methoxyphenyl)-3-methylazetidine-1-sulfonyl fluoride (**4s'**) as a colourless oil (17.1 mg, 31%).

major

^{1}H NMR (600 MHz, $CDCl_3$) δ 7.27 (d, *J* = 8.5 Hz, 2H), 6.94 – 6.91 (m, 2H), 5.30 (s, 1H), 4.16 (dd, *J* = 8.1, 2.0 Hz, 1H), 3.89 (dd, *J* = 8.0, 1.4 Hz, 1H), 3.81 (s, 3H), 2.35 (s, 1H), 1.12 (s, 3H).

^{13}C NMR (151 MHz, $CDCl_3$) δ 160.0, 127.6, 126.0, 114.2, 79.4, 72.4, 64.0, 55.4, 22.8.

^{19}F NMR (377 MHz, $CDCl_3$) δ 29.71 (s).

HRMS (ESI) *m/z*; $C_{11}H_{15}FNO_4S$ requires 276.0700 ($[M+H]^+$), found 276.0709.

IR (thin film, v_{max} /cm^{-1}) 3451, 2998, 1779, 1718, 1615, 1516, 1422, 1303, 1253, 1213, 1178, 1033, 831, 746, 622.

minor

^{1}H NMR (600 MHz, $CDCl_3$) δ 7.36 – 7.32 (m, 2H), 7.00 – 6.96 (m, 2H), 5.24 (d, *J* = 2.4 Hz, 1H), 4.20 (dd, *J* = 9.1, 2.0 Hz, 1H), 4.03 (dt, *J* = 9.2, 1.2 Hz, 1H), 3.82 (s, 3H), 1.75 (s, 1H), 1.58 (s, 3H).

^{13}C NMR (151 MHz, $CDCl_3$) δ 160.6, 128.6, 124.4, 114.7, 77.7, 71.0, 64.5, 55.5, 26.5.

^{19}F NMR (377 MHz, $CDCl_3$) δ 31.36 (s).

HRMS (ESI) *m/z*; $C_{11}H_{15}FNO_4S$ requires 276.0700 ($[M+H]^+$), found 276.0709.

IR (thin film, v_{max} /cm^{-1}) 3451, 2998, 1779, 1718, 1615, 1516, 1422, 1303, 1253, 1213, 1178, 1033, 831, 746, 622.

(2*R**,3*R**)-2-(4-Methoxyphenyl)-3-(2-oxopyrrolidin-1-yl)azetidine-1-sulfonyl fluoride (4t) and (2*S**,3*R**)-2-(4-Methoxyphenyl)-3-(2-oxopyrrolidin-1-yl)azetidine-1-sulfonyl fluoride (4t')

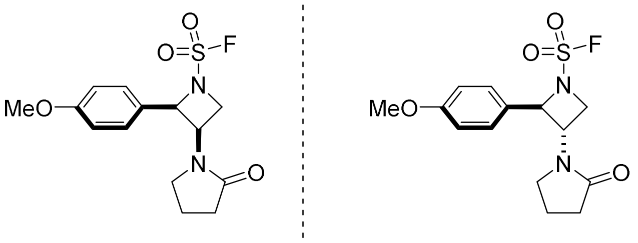

major **minor**

Prepared according to **GP1** using (4-methoxybenzylidene)sulfamoyl fluoride (**1**) (43.4 mg, 0.20 mmol, 1.0 eq.), 3-F-TX (4.6 mg, 0.020 mmol, 0.10 eq.) and 1-vinyl-2-pyrrolidinone (214 µl, 2.0 mmol, 10 eq.) in $(CH_2Cl)_2$ (1.0 ml, 0.20 M). ^{19}F NMR analysis of the crude reaction mixture revealed a diastereomer ratio of 3.0:1. Purification by flash column chromatography (50 – 70% EtOAc in pentane)

afforded(2R*,3R*)-2-(4-methoxyphenyl)-3-(2-oxopyrrolidin-1-yl)azetidine-1-sulfonyl fluoride (**4t**) as a pale-yellow oil (41.2 mg, 63%) and (2S*,3R*)-2-(4-methoxyphenyl)-3-(2-oxopyrrolidin-1-yl)azetidine-1-sulfonyl fluoride (**4t'**) as a colourless oil (3.7 mg, 5%).

major

^{1}H NMR (500 MHz, CDCl$_3$) δ 7.23 – 7.18 (m, 2H), 6.94 – 6.89 (m, 2H), 5.77 (d, J = 8.4 Hz, 1H), 5.29 (td, J = 8.8, 4.8 Hz, 1H), 4.61 (app td, J = 9.6, 1.9 Hz, 1H), 4.29 (ddd, J = 10.0, 5.0, 1.6 Hz, 1H), 3.80 (s, 3H), 3.29 (ddd, J = 9.2, 7.8, 6.8 Hz, 1H), 2.93 (td, J = 8.8, 4.5 Hz, 1H), 2.23 (ddd, J = 17.1, 9.2, 8.0 Hz, 1H), 2.06 (ddd, J = 17.0, 9.4, 5.3 Hz, 1H), 1.80 – 1.69 (m, 1H), 1.38 (ddtd, J = 12.7, 9.5, 8.2, 6.8 Hz, 1H).

^{13}C NMR (126 MHz, CDCl$_3$) δ 176.4, 159.7, 126.9, 125.5, 114.1, 72.2, 55.4, 53.0, 45.9, 44.8, 30.9, 18.5.

^{19}F NMR (377 MHz, CDCl$_3$) δ 30.30 (s).

HRMS (ESI) m/z $C_{14}H_{18}FN_2O_4S^+$ requires 329.0966 ([M+H]$^+$), found 329.0977.

IR (thin film, ν_{max} /cm^{-1}) 2959, 1691, 1614, 1515, 1464, 1422, 1294, 1253, 1211, 1177, 1112, 1031, 913, 843, 808, 755, 735, 683, 619.

minor

^{1}H NMR (400 MHz, CDCl$_3$) δ 7.40 – 7.32 (m, 2H), 6.95 – 6.89 (m, 2H), 5.43 (dd, J = 7.4, 2.1 Hz, 1H), 4.87 (q, J = 7.8 Hz, 1H), 4.34 (td, J = 8.1, 2.1 Hz, 1H), 4.22 (tt, J = 8.3, 1.1 Hz, 1H), 3.81 (s, 3H), 3.59 – 3.53 (m, 2H), 2.47 – 2.38 (m, 2H), 2.19 – 2.07 (m, 2H).

^{13}C NMR (101 MHz, CDCl$_3$) δ 175.6, 160.6, 128.4, 128.0, 114.5, 71.4, 55.5, 52.6, 49.9, 44.7, 31.2, 18.3.

^{19}F NMR (377 MHz, CDCl$_3$) δ 30.64.

HRMS (ESI) m/z $C_{14}H_{18}FN_2O_4S^+$ requires 329.0966 ([M+H]$^+$), found 329.0979.

IR (thin film, ν_{max} /cm^{-1}) 2921, 1667, 1613, 1515, 1493, 1465, 1422, 1398, 1294, 1250, 1207, 1178, 1071, 1032, 968, 913, 837, 737, 654.

(2R*,3R*)-3-(1H-Imid-1-yl)-2-(4-methoxyphenyl)azetidine-1-sulfonyl fluoride (4u) and (2R*,3S*)-3-(1H-Imidazol-1-yl)-2-(4-methoxyphenyl)azetidine-1-sulfonyl fluoride (4u')

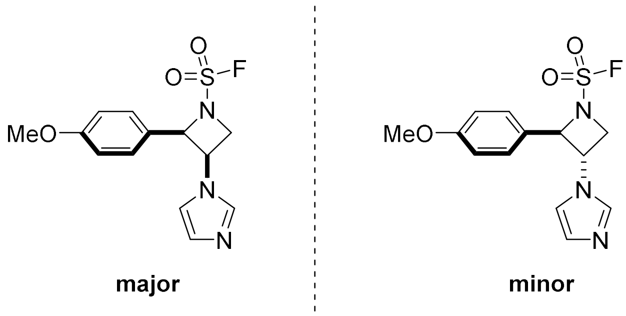

Prepared according to **GP1** using (4-methoxybenzylidene)sulfamoyl fluoride (**1**) (43.4 mg, 0.20 mmol, 1.0 eq.), [Ir(dFCF$_3$ppy)$_2$(dtbbpy)][PF$_6$] (2.2 mg, 0.002 mmol, 0.01 eq.), Aldrithiol-2 (2.2 mg, 0.010 mmol, 0.05 eq.) and N-vinyl imidazole (56 µl, 0.6 mmol, 3.0 eq.) in $(CH_2Cl)_2$ (1.0 ml, 0.20 M). ^{19}F NMR analysis of the crude reaction mixture revealed a diastereomer ratio of 2.9:1. Purification by flash column chromatography (0 – 3% MeOH in EtOAc) afforded (2R*,3R*)-3-(1H-imidazol-1-yl)-2-(4-methoxyphenyl)azetidine-1-sulfonyl fluoride (**4u**) as a white solid (30.0 mg, 48%) and (2R*,3S*)-3-(1H-imidazol-1-yl)-2-(4-methoxyphenyl)azetidine-1-sulfonyl fluoride (**4u'**) (10.6 mg, 17%) as a colourless oil.

Note: Excess N-vinyl imidazole can be distilled off using a rotary evaporator or under high vacuum.

major

R_f(2% MeOH/EtOAc) = 0.10.

^{1}H NMR (600 MHz, CDCl$_3$) 7.41 (s, 1H), 7.08 – 7.05 (m, 2H), 6.86 (s, 1H), 6.78 – 6.74 (m, 3H), 5.85 (d, *J* = 7.8 Hz, 1H), 5.27 (td, *J* = 8.6, 4.4 Hz, 1H), 4.84 (ddd, *J* = 10.2, 8.4, 1.8 Hz, 1H), 4.42 (dd, *J* = 10.3, 4.1 Hz, 1H), 3.73 (s, 3H).

^{13}C NMR (151 MHz, CDCl$_3$) δ 160.3, 136.6, 130.1, 127.2, 123.4, 117.9, 114.3, 72.1, 56.1, 55.3, 51.5.

^{19}F NMR (565 MHz, CDCl$_3$) δ 31.68.

HRMS (ESI) *m/z* $C_{13}H_{15}FN_3O_3S^+$ requires 312.0813 ([M+H]$^+$), found 312.0802.

IR (thin film, v_{max} /cm^{-1}) 1614, 1516, 1426, 1306, 1255, 1214, 1179, 1109, 1032, 845, 810, 764.

m.p. 230 °C (decomp.).

minor

R_f(2% MeOH/EtOAc) = 0.20.

^{1}H NMR (600 MHz, CDCl$_3$) δ 7.54 (s, 1H), 7.34 – 7.29 (m, 2H), 7.21 (s, 1H), 7.15 (d, *J* = 1.5 Hz, 1H), 6.98 – 6.92 (m, 2H), 5.41 (d, *J* = 6.7 Hz, 1H), 4.83 (q, *J* = 7.5 Hz, 1H), 4.56 (t, *J* = 8.5 Hz, 1H), 4.45 (t, *J* = 8.2 Hz, 1H), 3.82 (s, 3H).

^{13}C NMR (151 MHz, CDCl$_3$) δ 161.0, 136.7, 131.7, 127.9, 127.1, 116.5, 114.8, 76.3, 56.2, 55.5, 54.2.

^{19}F NMR (377 MHz, CDCl$_3$) δ 31.42(s).

HRMS (ESI) *m/z* $C_{13}H_{15}FN_3O_3S^+$ requires 312.0813 ([M+H]$^+$), found 312.0810.

IR (thin film, v_{max} /cm^{-1}) 2968, 1668, 1541, 1519, 1425, 1214, 1030, 760, 668.

m.p. 200 °C (decomp.).

(2*R**,3*R**)-3-(3,5-Dimethyl-1H-pyrazol-1-yl)-2-(4-methoxyphenyl)azetidine-1-sulfonyl fluoride (4u) and (2*R**,3*S**)-3-(3,5-Dimethyl-1H-pyrazol-1-yl)-2-(4-methoxyphenyl)azetidine-1-sulfonyl fluoride (4u')

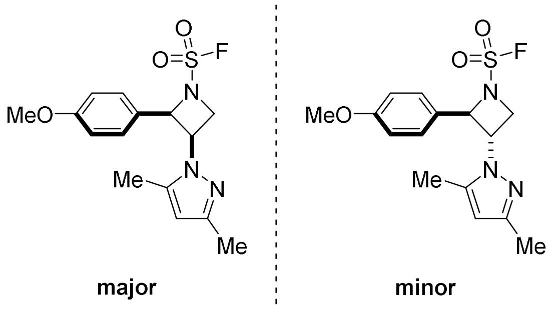

Prepared according to **GP1** using (4-methoxybenzylidene)sulfamoyl fluoride (**1**) (43.4 mg, 0.20 mmol, 1.0 eq.), 3-F-TX (4.6 mg, 0.020 mmol, 0.10 eq.) and 3,5-dimethyl-1-vinyl-1H-pyrazole (**O-40**) (76 µl, 0.60 mmol, 3.0 eq.) in $(CH_2Cl)_2$ (1.0 ml, 0.20 M). ^{19}F NMR analysis of the crude reaction mixture revealed a diastereomer ratio of 1.3:1. Purification by flash column chromatography (0 – 10% EtOAc in pentane) afforded (2*R**,3*R**)-3-(3,5-dimethyl-1H-pyrazol-1-yl)-2-(4-methoxyphenyl)azetidine-1-

sulfonyl fluoride (**4u**) as a white solid (30.4 mg, 45%) and (2*R**,3*S**)-3-(3,5-dimethyl-1H-pyrazol-1-yl)-2-(4-methoxyphenyl)azetidine-1-sulfonyl fluoride (**4u'**) (22.7 mg, 33%) as a colourless oil.

major

R_f (10% EtOAc in pentane) = 0.19.

^{1}H NMR (600 MHz, CDCl$_3$) δ 7.13 – 7.10 (m, 2H), 6.72 – 6.66 (m, 2H), 5.65 – 5.60 (m, 1H), 5.59 (s, 1H), 5.34 (dddd, *J* = 9.1, 4.1, 1.7, 0.9 Hz, 1H), 5.15 (ddd, *J* = 8.7, 7.5, 4.0 Hz, 1H), 4.67 (td, *J* = 8.9, 2.0 Hz, 1H), 3.74 (s, 3H), 2.15 (s, 3H), 1.85 (d, *J* = 0.8 Hz, 3H).

^{13}C NMR (151 MHz, CDCl$_3$) δ 160.3, 147.9, 139.5, 129.0, 124.6, 113.5, 106.8, 72.5, 55.3, 53.8, 51.8, 13.6, 10.8.

^{19}F NMR (565 MHz, CDCl$_3$) δ 31.81 (s).

HRMS (ESI) *m/z*; $C_{15}H_{19}FN_3O_3S$ requires 340.1126 ([M+H]$^+$), found 340.1125.

IR (thin film, ν_{max} /cm^{-1}) 2961, 1614, 1562, 1518, 1465, 1425, 1301, 1256, 1214, 1178, 1102, 1033, 839, 791, 771, 753.

m.p. 148 – 150 °C.

minor

R_f (10% EtOAc in pentane) = 0.26.

^{1}H NMR (600 MHz, CDCl$_3$) 7.36 – 7.32 (m, 2H), 6.94 – 6.90 (m, 2H), 5.80 (s, 1H), 5.70 (dd, *J* = 6.8, 2.2 Hz, 1H), 4.91 (td, *J* = 7.6, 2.1 Hz, 1H), 4.73 (td, *J* = 7.8, 6.7 Hz, 1H), 4.33 (t, *J* = 7.8 Hz, 1H), 3.81 (s, 3H), 2.26 (s, 3H), 1.99 (s, 3H).

^{13}C NMR (151 MHz, CDCl$_3$) δ 160.5, 149.5, 139.7, 128.4, 128.0, 114.6, 106.5, 75.0, 55.5, 55.3, 54.6, 13.8, 10.8.

^{19}F NMR (565 MHz, CDCl$_3$) δ 32.19 (s).

HRMS (ESI) *m/z*; $C_{15}H_{19}FN_3O_3S$ requires 340.1126 ([M+H]$^+$), found 340.1128.

IR (thin film, ν_{max} /cm^{-1}) 2962, 1615, 1562, 1517, 1461, 1423, 1308, 1255, 1213, 1178, 1105, 1033, 842, 793, 679.

Methyl (2*R**,3*S**)-3-acetamide-1-(fluorosulfonyl)-2-(4-methoxyphenyl)azetidine-3-carboxylate (4w) and Methyl (2*S**,3*S**)-3-acetamide-1-(fluorosulfonyl)-2-(4-methoxyphenyl)azetidine-3-carboxylate (4w')

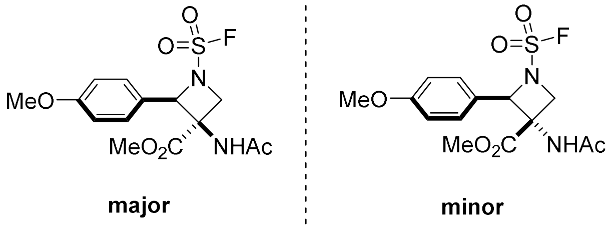

Prepared according to **GP1** using (4-methoxybenzylidene)sulfamoyl fluoride (**1**) (43.4 mg, 0.20 mmol, 1.0 eq.), ITX (5.1 mg, 0.020 mmol, 0.10 eq.) and methyl 2-acetamidoacrylate (286.3 mg 2.0 mmol, 10 eq.) in $(CH_2Cl)_2$ (1.0 ml, 0.20 M). ^{19}F NMR analysis of the crude reaction mixture revealed a diastereomer ratio of 4:1. Purification by flash column chromatography (50% - 67% EtOAc in petrol

ether) afforded methyl (2*R**,3*S**)-3-acetamide-1-(fluorosulfonyl)-2-(4-methoxyphenyl)azetidine-3-carboxylate (**4w**) and methyl (2*S**,3*S**)-3-acetamide-1-(fluorosulfonyl)-2-(4-methoxyphenyl)azetidine-3-carboxylate (**4w'**) as an inseparable mixture of diastereomers in the form of an off-white solid (32.4 mg, 45%).

major

^{1}H NMR (500 MHz, CDCl$_3$) δ 7.38 – 7.31 (m, 2H), 7.05 – 6.98 (m, 2H), 5.61 (s, 1H), 5.44 (s, 1H), 5.03 (dd, *J* = 9.2, 1.9 Hz, 1H), 4.09 (dd, *J* = 9.2, 1.5 Hz, 1H), 3.88 (s, 3H), 3.85 (s, 3H), 1.86 (s, 3H).

^{13}C NMR (126 MHz, CDCl$_3$) δ 170.6, 169.5, 161.2, 128.8, 122.5, 115.0, 71.4, 58.9, 56.8, 55.6, 53.9, 22.8.

^{19}F NMR (377 MHz, CDCl$_3$) δ 32.96 (d, *J* = 2.4 Hz).

HRMS (ESI) *m/z* $C_{14}H_{17}FN_2O_6SNa^+$ requires 383.0684 ([M+H]$^+$), found 383.0681.

IR (thin film, v_{max} /cm^{-1}) 3278, 2959, 1748, 1659, 1614, 1517, 1428, 1254, 1213, 1178, 1032, 912, 838, 767, 734.

minor

^{1}H NMR (500 MHz, CDCl$_3$) δ 7.25 (d, *J* = 5.7 Hz, 2H), 6.89 – 6.86 (m, 2H), 6.63 (s, 1H), 5.89 – 5.85 (m, 1H), 4.71 (dd, *J* = 8.3, 2.1 Hz, 1H), 4.67 (d, *J* = 8.3 Hz, 1H), 3.79 (s, 3H), 3.42 (s, 3H), 2.11 (s, 3H).

^{13}C NMR (126 MHz, CDCl$_3$) δ 170.9, 168.2, 160.5, 127.8, 125.0, 114.1, 73.0, 60.8, 57.1, 55.4, 53.1, 23.4.

^{19}F NMR (377 MHz, CDCl$_3$) δ 34.04 (d, *J* = 2.9 Hz).

HRMS (ESI) *m/z* $C_{14}H_{17}FN_2O_6SNa^+$ requires 383.0684 ([M+H]$^+$), found 383.0681.

IR (thin film, v_{max} /cm^{-1}) 3278, 2959, 1748, 1659, 1614, 1517, 1428, 1254, 1213, 1178, 1032, 912, 838, 767, 734.

Tert-butyl (tert-butoxycarbonyl)((2*R**,3*R**)-1-(fluorosulfonyl)-2-(4-methoxyphenyl)azetidin-3-yl)carbamate (4x) and Tert-butyl (tert-butoxycarbonyl)((2*R**,3*S**)-1-(fluorosulfonyl)-2-(4-methoxyphenyl)azetidin-3-yl)carbamate (4x')

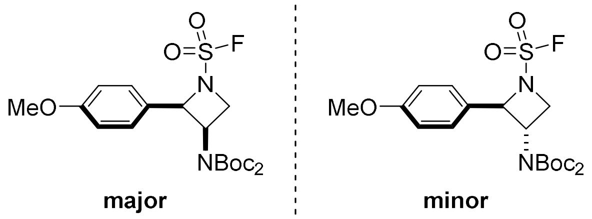

Prepared according to **GP1** using (4-methoxybenzylidene)sulfamoyl fluoride (**1**) (43.4 mg, 0.20 mmol, 1.0 eq.), 3-F-TX (4.6 mg, 0.020 mmol, 0.10 eq.), tert-butyl (tert-butoxycarbonyl)(vinyl)carbamate (**O-42**) (146 mg, 0.60 mmol, 3.0 eq.) and Aldrithiol-2 (2.2 mg, 0.010 mmol, 0.050 eq.) in $(CH_2Cl)_2$ (1.0 ml, 0.20 M). ^{19}F NMR analysis of the crude reaction mixture revealed a diastereomer ratio of 1.6:1. Purification by flash column chromatography (4 – 6% EtOAc in pentane) afforded a mixture of tert-butyl (tert-butoxycarbonyl)((2*R**,3*R**)-1-(fluorosulfonyl)-2-(4-methoxyphenyl)azetidin-3-yl)carbamate (**4x**) and tert-butyl (tert-butoxycarbonyl)((2*R**,3*S**)-1-(fluorosulfonyl)-2-(4-methoxyphenyl)azetidin-3-yl)carbamate (**4x'**) as a colourless oil (53.2 mg, 58%).

Note: compound degrades rapidly (2 – 3 days) even when stored at 0 °C.

major

^{1}H NMR (400 MHz, Acetone-d6) δ 7.40 – 7.35 (m, 2H), 6.96 – 6.92 (m, 2H), 5.76 (d, *J* = 2.2 Hz, 1H), 5.25 (ddd, *J* = 9.5, 8.4, 6.2 Hz, 1H), 4.87 (ddd, *J* = 8.9, 6.2, 2.9 Hz, 1H), 4.66 (td, *J* = 9.5, 2.3 Hz, 1H), 3.80 (s, 3H), 1.35 (s, 18H).

^{13}C NMR (151 MHz, Acetone-d6) δ 160.9, 153.0, 130.1, 126.6, 114.3, 83.5, 73.2, 55.9, 55.6, 51.4, 28.0.

^{19}F NMR (377 MHz, Acetone-d6) δ 32.82 (s).

HRMS (ESI) *m/z* $C_{20}H_{29}FN_2O_7SNa^+$ requires 483.1572 ([M+H]$^+$), found 483.1577.

IR (thin film, ν_{max} /cm^{-1}) 2983, 1818, 1740, 1704, 1614, 1517, 1480, 1461, 1425, 1396, 1371, 1354, 1307, 1276, 1255, 1213, 1179, 1126, 1035, 912, 843, 808, 777, 758, 736.

minor

^{1}H NMR (400 MHz, Acetone-d6) δ 7.50 – 7.45 (m, 2H), 7.00 – 6.96 (m, 2H), 5.78 (d, *J* = 2.4 Hz, 1H), 5.02 (q, *J* = 7.6 Hz, 1H), 4.65 – 4.60 (m, 1H), 4.37 – 4.32 (m, 1H), 3.82 (s, 3H), 1.49 (s, 18H).

^{13}C NMR (151 MHz, Acetone-d6) δ 161.4, 153.2, 130.2, 129.6, 114.8, 84.3, 73.8, 55.9, 55.1, 54.2, 28.0.

^{19}F NMR (377 MHz, Acetone-d6) δ 33.64 (s).

HRMS (ESI) *m/z* $C_{20}H_{29}FN_2O_7SNa^+$ requires 483.1572 ([M+H]$^+$), found 483.1577.

IR (thin film, ν_{max} /cm^{-1}) 2983, 1818, 1740, 1704, 1614, 1517, 1480, 1461, 1425, 1396, 1371, 1354, 1307, 1276, 1255, 1213, 1179, 1126, 1035, 912, 843, 808, 777, 758, 736.

(2*R**,3*S**)-3-(1,3-Dioxoisoindolin-2-yl)-2-(4-methoxyphenyl)azetidine-1-sulfonyl fluoride (4y) and (2*R**,3*R**)-3-(1,3-Dioxoisoindolin-2-yl)-2-(4-methoxyphenyl)azetidine-1-sulfonyl fluoride (4y')

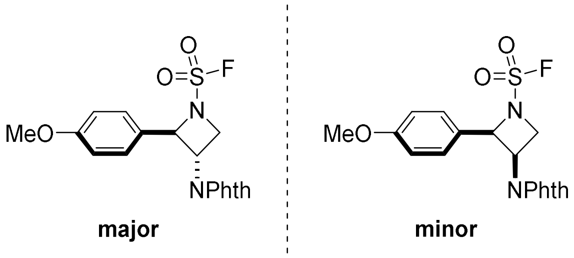

Prepared according to **GP1** using (4-methoxybenzylidene)sulfamoyl fluoride (**1**) (43.4 mg, 0.20 mmol, 1.0 eq.), 3-F-TX (4.6 mg, 0.020 mmol, 0.10 eq.), N-vinyl phthalimide (52 mg, 0.60 mmol, 3.0 eq.) in $(CH_2Cl)_2$ (1.0 ml, 0.20 M). ^{19}F NMR analysis of the crude reaction mixture revealed a diastereomer ratio of 2.2:1. Purification by flash column chromatography (50 – 75% CH_2Cl_2 in pentane) afforded (2*R**,3*S**)-3-(1,3-dioxoisoindolin-2-yl)-2-(4-methoxyphenyl)azetidine-1-sulfonyl fluoride (**4y**) as a white solid (20.6 mg, 26%) and a 1.4:1 mixture of (2*R**,3*R**)-3-(1,3-dioxoisoindolin-2-yl)-2-(4-methoxyphenyl)azetidine-1-sulfonyl fluoride (**4y'**) and (2*R**,3*S**)-3-(1,3-dioxoisoindolin-2-yl)-2-(4-methoxyphenyl)azetidine-1-sulfonyl fluoride (**4y**) (23.1 mg, 30%) as a yellow solid.

major

R_f (60% CH_2Cl_2 in pentane) = 0.25.

^{1}H NMR (400 MHz, CDCl3) δ 7.93 – 7.84 (m, 2H), 7.84 – 7.74 (m, 2H), 7.44 – 7.36 (m, 2H), 6.96 – 6.88 (m, 2H), 6.10 (dd, *J* = 7.4, 2.1 Hz, 1H), 5.04 (td, *J* = 8.3, 7.4 Hz, 1H), 4.89 (td, *J* = 7.8, 2.4 Hz, 1H), 4.28 (ddd, *J* = 8.3, 6.5, 2.9 Hz, 1H), 3.81 (s, 3H).

^{13}C NMR (151 MHz, $CDCl_3$) δ 167.6, 160.7, 134.9, 131.5, 128.6, 128.1, 124.0, 114.6, 71.3, 55.5, 52.9, 47.1.

^{19}F NMR (565 MHz, $CDCl_3$) δ 32.64(s).

HRMS (ESI) *m/z*; $C_{18}H_{16}FN_2O_5S$ requires 391.0759 ($[M+H]^+$), found 391.0768.

IR (thin film, v_{max} /cm^{-1}) 2962, 1779, 1729, 1614, 1517, 1469, 1420, 1387, 1256, 1211, 1105, 1031, 844, 814, 774, 720.

m.p. 116 – 120 °C.

minor

R_f (60% CH_2Cl_2 in pentane) = 0.19.

^{1}H NMR (600 MHz, $CDCl_3$) δ 7.69 (td, *J* = 5.1, 2.0 Hz, 2H), 7.65 (dt, *J* = 5.3, 3.5 Hz, 2H), 7.25 – 7.22 (m, 2H), 6.76 – 6.70 (m, 2H), 5.85 (d, *J* = 8.1 Hz, 1H), 5.44 – 5.35 (m, 2H), 4.71 – 4.64 (m, 1H), 3.66 (s, 3H).

^{13}C NMR (151 MHz, $CDCl_3$) δ 167.8, 159.8, 134.5, 131.0, 127.9, 124.7, 123.5, 114.0, 72.4, 55.3, 51.9, 45.5.

^{19}F NMR (565 MHz, $CDCl_3$) δ 32.06(s).

HRMS (ESI) *m/z*; $C_{18}H_{16}FN_2O_5S$ requires 391.0759 ($[M+H]^+$), found 391.0760.

IR (thin film, v_{max} /cm^{-1}) 2962, 2920, 1779, 1719, 1615, 1516, 1468, 1419, 1383, 1256, 1211, 1104, 843, 774, 720.

(2*R**,3*S**)-1-(Fluorosulfonyl)-2-(4-methoxyphenyl)-3-methylazetidin-3-yl acetate (4z) and (2*R**,3*R**)-1-(Fluorosulfonyl)-2-(4-methoxyphenyl)-3-methylazetidin-3-yl acetate (4z')

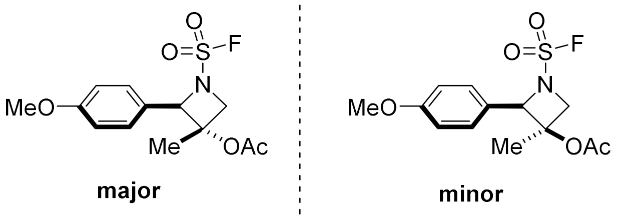

Prepared according to **GP1** using (4-methoxybenzylidene)sulfamoyl fluoride (**1**) (43.4 mg, 0.20 mmol, 1.0 eq.), 3-F-TX (4.6 mg, 0.020 mmol, 0.10 eq.) and isopropenyl acetate (220 µl, 2.0 mmol, 10 eq.) in $(CH_2Cl)_2$ (1.0 ml, 0.20 M). ^{19}F NMR analysis of the crude reaction mixture revealed a diastereomer ratio of 4.4:1. Purification by flash column chromatography (0 – 10% Et_2O in pentane) afforded (2*R**,3*S**)-1-(fluorosulfonyl)-2-(4-methoxyphenyl)-3-methylazetidin-3-yl acetate (**4z**) as a colourless oil (28.8 mg, 45%) and (2*R**,3*R**)-1-(fluorosulfonyl)-2-(4-methoxyphenyl)-3-methylazetidin-3-yl acetate (**4z'**) as a colourless oil (22.2 mg, 35%).

major

R_f(10% Et_2O in pentane) = 0.20.

^{1}H NMR (500 MHz, CDCl$_3$) δ 7.38 – 7.30 (m, 2H), 6.96 – 6.92 (m, 2H), 5.43 (s, 1H), 4.34 (dd, *J* = 9.3, 2.0 Hz, 1H), 4.12 (d, *J* = 9.3 Hz, 1H), 3.82 (s, 3H), 2.12 (s, 3H), 1.24 (s, 3H).

^{13}C NMR (126 MHz, CDCl$_3$) δ 169.7, 160.2, 128.4, 125.2, 114.2, 77.2, 76.6, 62.4, 55.4, 21.4, 19.1.

^{19}F NMR (470 MHz, CDCl$_3$) δ 30.60(s).

HRMS (ESI) *m/z* $C_{13}H_{17}FNO_5S^+$ requires 318.0806 ([M+H]$^+$), found 318.0817.

IR (thin film, v_{max} /cm^{-1}) 2940, 1747, 1616, 1517, 1427, 1372, 1306, 1252, 1213, 1177, 1148, 1123, 1081, 1031, 968, 912, 838, 817, 775, 740, 685, 607.

minor

R$_f$(10% Et$_2$O in pentane) = 0.07.

^{1}H NMR (500 MHz, CDCl$_3$) δ 7.35 – 7.29 (m, 2H), 6.95 – 6.91 (m, 2H), 5.22 (d, *J* = 2.1 Hz, 1H), 4.39 (dd, *J* = 10.2, 2.3 Hz, 1H), 4.29 (dd, *J* = 10.3, 2.1 Hz, 1H), 3.83 (s, 3H), 1.84 (s, 3H), 1.81 (s, 3H).

^{13}C NMR (126 MHz, CDCl$_3$) δ 169.6, 160.3, 129.2, 125.0, 113.9, 77.6, 76.5, 62.4, 55.4, 23.5, 21.3.

^{19}F NMR (470 MHz, CDCl$_3$) δ 33.41(s).

HRMS (ESI) *m/z* $C_{13}H_{17}FNO_5S^+$ requires 318.0806 ([M+H]$^+$), found 318.0821.

IR (thin film, v_{max} /cm^{-1}) 2939, 1744, 1615, 1516, 1423, 1371, 1308, 1252, 1211, 1178, 1078, 1031, 914, 833, 778, 742, 670, 605.

(2*S**,2a*S**,7a*S**)-2-(4-Methoxyphenyl)-2a,7a-dihydrobenzofuro[2,3-*b*]azete-1(2*H*)-sulfonyl fluoride (4aa)

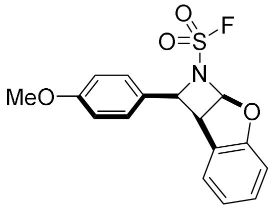

Prepared according to **GP1** using (4-methoxybenzylidene)sulfamoyl fluoride (**1**) (43.4 mg, 0.20 mmol, 1.0 eq.), 3-F-TX (4.6 mg, 0.020 mmol, 0.10 eq.), 2,3-benzofuran (220 µl, 2.0 mmol, 10 eq.) $(CH_2Cl)_2$ (1.0 ml, 0.20 M).^{19}F NMR analysis of the crude reaction mixture revealed a diastereomer ratio of 4.4:1. Purification by flash column chromatography (10 – 20% Et$_2$O in pentane) afforded (2*S**,2a*S**,7a*S**)-2-(4-methoxyphenyl)-2a,7a-dihydrobenzofuro[2,3-*b*]azete-1(2*H*)-sulfonyl fluoride (**4aa**) as a white crystalline solid (40.4 mg, 60%).

^{1}H NMR (400 MHz, CDCl3) δ 7.15 (t, *J* = 7.8 Hz, 1H), 6.96 (d, *J* = 8.2 Hz, 1H), 6.84 (d, *J* = 8.3 Hz, 2H), 6.72 – 6.62 (m, 3H), 6.44 – 6.35 (m, 2H), 5.60 (d, *J* = 7.4 Hz, 1H), 4.45 (t, *J* = 6.6 Hz, 1H), 3.67 (s, 3H).

^{13}C NMR (101 MHz, CDCl3) δ 160.0, 159.9, 130.0, 128.0, 127.0, 125.7, 122.6, 122.4, 113.7, 111.4, 94.0, 69.3, 55.2, 48.1.

^{19}F NMR (377 MHz, CDCl3) δ 43.26(s).

HRMS (ESI) *m/z* $C_{16}H_{14}FNO_4SNa^+$ requires 358.0520 ([M+Na]$^+$), found 358.0530,

IR (thin film, v_{max} /cm^{-1}) 2936, 1615, 1516, 1478, 1463, 1428, 1306, 1253, 1209, 1177, 1155, 1090, 1021, 948, 911, 856, 835, 796, 757, 734, 666.

m.p. 118–120 °C.

(1*R**,6*S**,8*R**)-8-(4-Methoxyphenyl)-2-oxa-7-azabicyclo[4.2.0]octane-7-sulfonyl fluoride (4ab) and (1*R**,6*S**,8*S**)-8-(4-Methoxyphenyl)-2-oxa-7-azabicyclo[4.2.0]octane-7-sulfonyl fluoride (4ab')

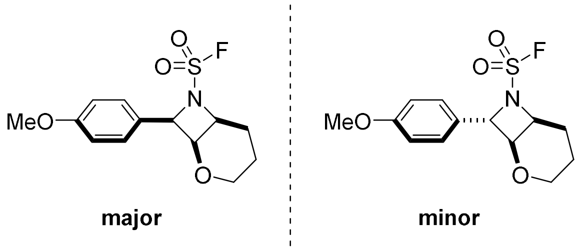

Prepared according to **GP1** using (4-methoxybenzylidene)sulfamoyl fluoride (**1**) (43.4 mg, 0.20 mmol, 1.0 eq.), ITX (5.1 mg, 0.020 mmol, 0.10 eq.) and 3,4-dihydro-2H-pyran (183 µl, 2.0 mmol, 10 eq.) in $(CH_2Cl)_2$ (1.0 ml, 0.20 M). ^{19}F NMR analysis of the crude reaction mixture revealed a diastereomer ratio of 1.5:1. Purification by flash column chromatography (0 – 20% Et_2O in pentane) afforded(1*R**,6*S**,8*R**)-8-(4-methoxyphenyl)-2-oxa-7-azabicyclo[4.2.0]octane-7-sulfonyl fluoride (**4ab**) as a colourless oil (15.8 mg, 26%) and (1*R**,6*S**,8*S**)-8-(4-methoxyphenyl)-2-oxa-7-azabicyclo[4.2.0]octane-7-sulfonyl fluoride (**4ab'**) as a colourless oil (11.6 mg, 19%).

major

R_f (20% Et_2O in pentane) = 0.06.

^1H NMR (600 MHz, $CDCl_3$) δ 7.41 – 7.35 (m, 2H), 6.95 – 6.87 (m, 2H), 5.26 – 5.22 (m, 1H), 4.44 (t, *J* = 4.9 Hz, 1H), 4.40 (t, *J* = 5.0 Hz, 1H), 3.90 (ddq, *J* = 11.6, 4.3, 2.0 Hz, 1H), 3.80 (s, 3H), 3.31 (td, *J* = 11.6, 1.9 Hz, 1H), 2.33 (ddt, *J* = 15.3, 4.5, 2.3 Hz, 1H), 2.14 – 2.02 (m, 1H), 1.77 (ddt, *J* = 15.4, 12.5, 5.3 Hz, 1H), 1.58 (ddt, *J* = 13.9, 5.5, 2.5 Hz, 1H).

^{13}C NMR (151 MHz, $CDCl_3$) δ 160.1, 129.6, 124.5, 113.8, 70.2, 69.5, 64.3, 60.4, 55.4, 23.9, 20.6.

^{19}F NMR (565 MHz, $CDCl_3$) δ 28.96.

HRMS (ESI) *m/z* $C_{13}H_{17}FNO_4S^+$ requires 302.0857 ($[M+H]^+$), found 302.0864.

IR (thin film, ν_{max} /cm^{-1}) 2954, 1614, 1515, 1420, 1304, 1252, 1210, 1177, 1140, 1109, 1073, 1060, 1038, 998, 913, 835, 800, 752, 686.

minor

R_f (20% Et_2O in pentane) = 0.15.

^1H NMR (600 MHz, $CDCl_3$) δ 7.37 – 7.32 (m, 2H), 6.97 – 6.93 (m, 2H), 5.31 (t, *J* = 3.6 Hz, 1H), 4.69 – 4.60 (m, 1H), 4.36 (dd, *J* = 6.4, 3.9 Hz, 1H), 4.00 (dt, *J* = 11.5, 5.8 Hz, 1H), 3.84 (s, 3H), 3.65 (ddd, *J* = 11.8, 7.3, 4.9 Hz, 1H), 2.36 (ddt, *J* = 14.3, 9.9, 5.3 Hz, 1H), 2.18 (dddd, *J* = 14.2, 7.8, 6.3, 4.7 Hz, 1H), 2.03 (dtt, *J* = 14.9, 7.6, 5.1 Hz, 1H), 1.61 (dddd, *J* = 14.0, 9.0, 6.3, 4.6 Hz, 1H).

^{13}C NMR (101 MHz, CDCl3) δ 160.3, 128.6, 127.5, 114.4, 74.1, 72.7, 63.3, 62.1, 55.5, 22.2, 19.5.

^{19}F NMR (377 MHz, $CDCl_3$) δ 49.73(s).

HRMS (ESI) *m/z* $C_{13}H_{17}FNO_4S^+$ requires 302.0857 ($[M+H]^+$), found 302.0855.

IR (thin film, ν_{max} /cm^{-1}) 2957, 1614, 1516, 1461, 1416, 1252, 1206, 1178, 1154, 1079, 1033, 912, 837, 771, 746, 615.

Telescoped energy-transfer mediated intermolecular aza-Paternò-Büchi reaction from aldehyde

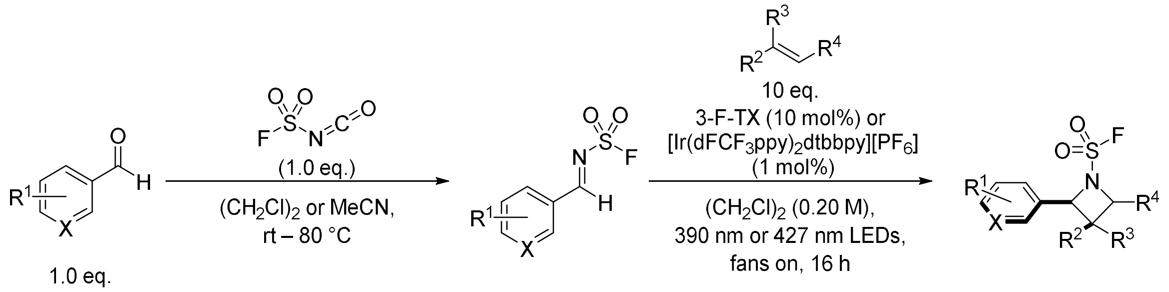

Azetidine characterisation

3,3-Diethyl-2-(4-methoxyphenyl)azetidine-1-sulfonyl fluoride (3a)

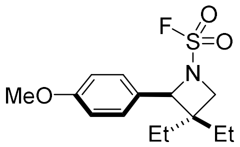

Fluorosulfonyl isocyanate (FSI) (16 µl, 0.20 mmol, 1.0 eq.) was added dropwise to a stirred solution of 4-anisaldehyde (0.20 mmol, 1.0 eq.) in dry $(CH_2Cl)_2$ (0.50 ml, 0.4 M) under nitrogen at 0 °C in a tapered microwave vial. (Caution: CO_2 evolution). The reaction mixture was then warmed to rt and stirred for 16 h. A solution of $[Ir(dFCF_3ppy)_2dtbbpy][PF_6]$ in $(CH_2Cl)_2$ (0.002 M, 1.0 ml, 1 mol%) was then added followed by the addition of 2-ethyl-1-butene (241 µl, 2.0 mmol, 10 eq). The reaction was then stirred under blue light irradiation (427 nm) at ambient temperature for 16 h. The reaction mixture was diluted with CH_2Cl_2, and the solvent removed under reduced pressure. Purification by flash column chromatography (0 – 2 Et_2O in pentane) afforded (*R**)-3,3-diethyl-2-(4-methoxyphenyl)azetidine-1-sulfonyl fluoride (**3a**) as a colourless oil (39.2 mg, 65%).

Characterisation as before (**3a**).

3,3-Diethyl-2-(5-hydroxy-6-methoxypyridin-3-yl)azetidine-1-sulfonyl fluoride (3t)

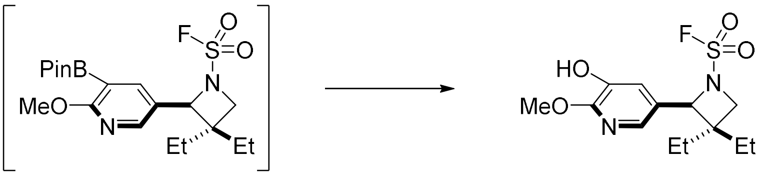

FSI (16 µl, 0.20 mmol, 1.0 eq.) was added dropwise to a stirred solution of 6-Methoxy-5-(4,4,5,5-tetramethyl-1,3,2-dioxaborolan-2-yl)nicotinaldehyde (**A-27**) (0.2 mmol, 1.0 eq.) in dry $(CH_2Cl)_2$ (1.0 ml, 0.2 M) under nitrogen at 0 °C in a tapered microwave vial. (Caution: CO_2 evolution). The reaction mixture was then heated to 55 °C and stirred for 16 h. After cooling to rt, the solvent was removed by a constant flow of N_2, before a solution of 3-F-TX in $(CH_2Cl)_2$ (0.020 M, 1.0 ml, 10 mol%) was added, followed by 2-ethyl-1-butene (241 µl, 2.0 mmol, 10 eq.). The reaction was then stirred under purple light irradiation (390 nm) at ambient temperature for 16 h. Following the photochemical reaction, the reaction mixture was transferred to a round bottom flask and concentrated under reduced pressure. The crude reaction mixture was dissolved in a 1:1 mixture of THF/H_2O (3.2 ml, 0.063 M) followed by addition of $NaBO_3$ $4H_2O$ (462 mg, 3.0 mmol, 15 eq.). The reaction mixture was stirred under air at rt for 1 h after which the reaction mixture was transferred to a separatory funnel. Brine was added and the layers partitioned. The aqueous layer was extracted three times with Et_2O, the combined organic

fractions were dried over anhydrous $MgSO_4$ and concentrated under reduced pressure. Purification by flash column chromatography (0 – 20 % Et_2O in pentane) afforded 3,3-diethyl-2-(5-hydroxy-6-methoxypyridin-3-yl)azetidine-1-sulfonyl fluoride (**3t**) as a colourless oil (22.4 mg, 35%).

R_f (20% EtOAc in pentane) = 0.24.

^{1}H NMR (400 MHz, $CDCl_3$) δ 7.67 (d, *J* = 2.1 Hz, 1H), 7.17 (d, *J* = 2.1 Hz, 1H), 5.52 (s, 1H), 5.13 (d, *J* = 2.1 Hz, 1H), 4.03 (s, 3H), 3.84 (d, *J* = 7.7 Hz, 1H), 3.74 (dd, *J* = 8.0, 1.7 Hz, 1H), 1.83 – 1.72 (m, 2H), 1.42 (dq, *J* = 14.9, 7.3 Hz, 1H), 1.27 – 1.14 (m, 1H), 0.93 (t, *J* = 7.4 Hz, 3H), 0.63 (t, *J* = 7.4 Hz, 3H).

^{13}C NMR (101 MHz, $CDCl_3$) δ 153.0, 140.4, 135.7, 125.5, 119.6, 73.1, 60.2, 54.0, 43.7, 29.3, 24.4, 8.0, 7.4.

^{19}F NMR (377 MHz, $CDCl_3$) δ 31.33(s).

HRMS (ESI) *m/z* $C_{13}H_{18}FN_2O_4S^-$ requires 317.0977 ([M-H]$^-$), found 317.0991.

IR (thin film, ν_{max} /cm^{-1}) 3486, 2970, 1491, 1419, 1254, 1214, 1164, 1039, 1020, 913, 760, 622.

2-(2,4-Dimethoxypyrimidin-5-yl)-3,3-diethylazetidine-1-sulfonyl fluoride (3u)

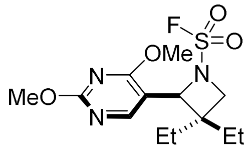

FSI (16 µl, 0.20 mmol, 1.0 eq.) was added dropwise to a stirred solution of 2,4-dimethoxypyrimidine-5-carbaldehyde (**A-28**) (0.2 mmol, 1.0 eq.) in dry $(CH_2Cl)_2$ (1.0 ml, 0.2 M) under nitrogen at 0 °C in a tapered microwave vial. (Caution: CO_2 evolution). The reaction mixture was then heated to 55 °C and stirred for 16 h. The reaction was then cooled to rt and the solvent removed by a constant flow of N_2.A solution of 3-F-TX in $(CH_2Cl)_2$ (0.020 M, 1.0 ml, 10 mol%) was then added followed by the addition of 2-ethyl-1-butene (241 µl, 2.0 mmol, 10 eq). The reaction was then stirred under purple light irradiation (390 nm) at ambient temperature for 16 h. The reaction mixture was diluted with CH_2Cl_2, and the solvent removed under reduced pressure. Purification by flash column chromatography (0 – 20 % Et_2O in pentane) afforded 2-(2,4-dimethoxypyrimidin-5-yl)-3,3-diethylazetidine-1-sulfonyl fluoride (**3u**) as a white solid (43.0 mg, 64%).

R_f (20% Et_2O in pentane) = 0.20.

^{1}H NMR (600 MHz, $CDCl_3$) δ 8.40 (s, 1H), 5.31 (s, 1H), 4.00 (s, 3H), 3.98 (s, 3H), 3.89 (d, *J* = 8.0 Hz, 1H), 3.68 (d, *J* = 8.1 Hz, 1H), 1.81 (dq, *J* = 14.8, 7.4 Hz, 1H), 1.69 (dq, *J* = 14.6, 7.4 Hz, 1H), 1.49 – 1.40 (m, 1H), 1.27 – 1.18 (m, 1H), 0.95 (t, *J* = 7.5 Hz, 3H), 0.62 (t, *J* = 7.4 Hz, 3H).

^{13}C NMR (151 MHz, $CDCl_3$) δ 168.2, 165.4, 157.1, 109.6, 68.0, 59.4, 55.1, 54.1, 43.9, 28.7, 25.3, 8.1, 7.5.

^{19}F NMR (565 MHz, $CDCl_3$) δ 30.86(s).

HRMS (ESI) *m/z* $C_{13}H_{20}FN_3O_4SNa^+$ requires 356.1051 ([M+Na]$^+$), found 356.1047.

IR (thin film, ν_{max} /cm^{-1}) 2971, 1606, 1573, 1474, 1425, 1404, 1215, 1079, 1052, 1018, 753.

m.p. 79 – 81 °C.

3,3-Diethyl-2-(2-methoxy-1-methyl-4-oxo-1,4-dihydropyrimidin-5-yl)azetidine-1-sulfonyl fluoride (3v)

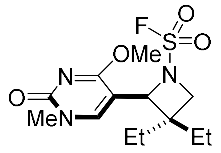

FSI (16 µl, 0.20 mmol, 1.0 eq.) was added dropwise to a stirred solution of 2,4-dimethoxypyrimidine-5-carbaldehyde (**A-28**) (0.20 mmol, 1.0 eq.) in dry MeCN (0.20 ml, 1.0 M) under nitrogen at 0 °C in a tapered microwave vial. (Caution: CO_2 evolution). The reaction mixture was then heated to 84 °C and stirred for 16 h. The reaction was then cooled to rt and the solvent removed by a constant flow of N_2. A solution of 3-F-TX in $(CH_2Cl)_2$ (0.020 M, 1.0 ml, 10 mol%) was then added followed by the addition of 2-ethyl-1-butene (241 µl, 2.0 mmol, 10 eq.). The reaction was then stirred under purple light irradiation (390 nm) at ambient temperature for 16 h. The reaction mixture was diluted with CH_2Cl_2, and the solvent removed under reduced pressure. Purification by flash column chromatography (50 – 70 % EtOAc in pentane) afforded 3,3-diethyl-2-(2-methoxy-1-methyl-4-oxo-1,4-dihydropyrimidin-5-yl)azetidine-1-sulfonyl fluoride (**3v**) as a colourless oil (40.0 mg, 60%).

Note: Imine intermediate undergoes a rearrangement before photo-addition from pyrimidine to pyrimidone.

R_f (60% EtOAc in pentane) = 0.15.

^{1}H NMR (600 MHz, CDCl$_3$) δ 7.58 (s, 1H), 5.14 (s, 1H), 3.94 (s, 3H), 3.84 (d, *J* = 8.1 Hz, 1H), 3.59 (d, *J* = 8.1 Hz, 1H), 3.52 (s, 3H), 1.77 (dq, *J* = 14.8, 7.4 Hz, 1H), 1.64 (dq, *J* = 14.8, 7.5 Hz, 1H), 1.43 (dq, *J* = 14.9, 7.5 Hz, 1H), 1.28 (dq, *J* = 14.5, 7.3 Hz, 1H), 0.94 (t, *J* = 7.5 Hz, 3H), 0.67 (t, *J* = 7.4 Hz, 3H).

^{13}C NMR (151 MHz, CDCl$_3$) δ 168.6, 156.6, 146.2, 103.2, 67.4, 59.1, 54.7, 43.7, 38.5, 28.4, 25.4, 8.1, 7.5.

^{19}F NMR (565 MHz, CDCl$_3$) δ 30.27(s).

HRMS (ESI) *m/z* $C_{13}H_{21}FN_3O_4S^+$ requires 334.1231 ([M+H]$^+$), found 334.1228.

IR (thin film, v_{max} /cm^{-1}) 2969, 2240, 1665, 1540, 1422, 1348, 1213, 1004, 913, 735.

m.p. 112 – 114 °C.

2-(benzo[d]oxazol-5-yl)-3,3-diethylazetidine-1-sulfonyl fluoride (3x)

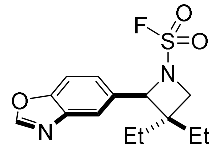

FSI (16 µl, 0.20 mmol, 1.0 eq.) was added dropwise to a stirred solution of benzo[d]oxazole-5-carbaldehyde (0.2 mmol, 1.0 eq.) in dry $(CH_2Cl)_2$ (0.5 ml, 0.4 M) under nitrogen at rt in a tapered microwave vial. (Caution: CO_2 evolution). The reaction mixture was then heated to 55 °C and stirred for 16 h. The reaction was then cooled to rt and a solution of 3-F-TX in $(CH_2Cl)_2$ (0.040 M, 0.5 ml, 10 mol%) was then added followed by the addition of 2-ethyl-1-butene (241 µl, 2.0 mmol, 10 eq). The reaction was then stirred under purple light irradiation (390 nm) at ambient temperature for 16 h. The reaction mixture was diluted with CH_2Cl_2, and the solvent removed under reduced pressure.

Purification by flash column chromatography (0 – 6 % EtOAc in pentane) afforded (-2-(benzo[d]oxazol-5-yl)-3,3-diethylazetidine-1-sulfonyl fluoride (**3x**) as a colourless oil (33.2 mg, 53%).

R_f (10% EtOAc in pentane) = 0.20.

^{1}H NMR (400 MHz, CDCl$_3$) δ 8.12 (s, 1H), 7.81 (s, 1H), 7.60 (d, *J* = 8.47 Hz, 1H), 7.41 (d, *J* = 8.35 Hz, 1H), 5.35 (d, *J* = 2.11 Hz, 1H), 3.91 (d, *J* = 8.00 Hz, 1H), 3.79 (dd, *J* = 8.04, 1.81 Hz, 1H), 1.93 – 1.72 (m, 2H), 1.38 (dq, *J* = 14.87, 7.46 Hz, 1H), 1.12 (dq, *J* = 14.65, 7.41 Hz, 1H), 0.99 (t, *J* = 7.44 Hz, 3H), 0.56 (t, *J* = 7.43 Hz, 3H)

^{13}C NMR (151 MHz, CDCl$_3$) δ 153.4, 140.6, 132.3, 124.6, 119.2, 111.0, 75.2, 60.0, 43.8, 29.3, 24.5, 8.2, 7.3.

^{19}F NMR (377 MHz, CDCl$_3$) δ 31.56.

HRMS (ESI) *m/z* $C_{14}H_{17}FN_2O_3S^+$ requires 313.1017 ([M+H]$^+$), found 313.1016.

IR (thin film, v_{max} /cm^{-1}) 1719, 1596, 1537, 1424, 1214, 1051, 911, 734.

One-gram scale reaction and derivatisation of azetidine sulfamoyl fluorides

One-gram scale reaction

3,3-Diethyl-2-(4-methoxyphenyl)azetidine-1-sulfonyl fluoride (3a)

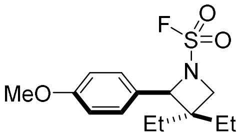

Method A: A Schlenk flask was charged with (4-methoxybenzylidene)sulfamoyl fluoride (**1**) (1.09 g, 5.0 mmol, 1.0 eq.) and [Ir(dFCF$_3$ppy)$_2$(dtbbpy)][PF$_6$] (56.1 mg, 0.01 eq, 0.05 mmol). The flask was capped with a septum and evacuated and backfilled with N_2 thrice. $(CH_2Cl)_2$ (25 ml, 0.20 M) and 2-ethyl-1-butene (1.9 ml, 15 mmol, 3.0 eq.) were added sequentially *via* syringe and the reaction was stirred under irradiation with 427 nm light for 16 h. The reaction was concentrated under reduced pressure and purified by flash column chromatography (0 – 3% Et_2O in pentane) to give 3,3-diethyl-2-(4-methoxyphenyl)azetidine-1-sulfonyl fluoride (**3a**) as a colourless oil (1.16 g, 77%).

Method B: A Schlenk flask was charged with (4-methoxybenzylidene)sulfamoyl fluoride (**1**) (1.09 g, 5.0 mmol, 1.0 eq.) and 3-F-TX (115 mg, 0.10 eq, 0.5 mmol). The flask was capped with a septum and then evacuated and backfilled with N_2 thrice. $(CH_2Cl)_2$ (25 ml, 0.20 M) and 2-ethyl-1-butene (1.9 ml, 15 mmol, 3.0 eq.) were added sequentially *via* syringe and the reaction was stirred under irradiation with 390 nm light for 16 h. The reaction was then concentrated under reduced pressure and the crude residue was redissolved in CH_2Cl_2 (25 ml), before cooling to 0 °C and *m*-CPBA (1.0 eq.) was added in one portion. The reaction mixture was warmed to rt and stirred for 1 h. Following completion, the reaction mixture was quenched with aq. sat. $NaHSO_3$ and the layers partitioned. The aqueous layer was extracted with CH_2Cl_2 three times, combined organic layers were washed sequentially with aq. sat. $NaHCO_3$, brine, dried over anhydrous $MgSO_4$ subsequently filtered and concentrated under reduced pressure. Purification by flash column chromatography (0 – 5% Et_2O in pentane) afforded 3,3-diethyl-2-(4-methoxyphenyl)azetidine-1-sulfonyl fluoride (**3a**) as a colourless oil (1.17 g, 77%).

Characterisation as before (**3a**).

Cleavage of -SO_2F moiety

3,3-Diethyl-2-(4-methoxyphenyl)azetidine (5)

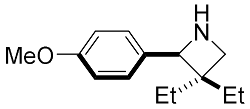

Sodium bis(2-methoxyethoxy)aluminium hydride (0.14 ml, 0.5 mmol, 60 wt% in toluene, 5.0 eq.) was added to a stirred solution of (*R**)-3,3-diethyl-2-(4-methoxyphenyl)azetidine-1-sulfonyl fluoride (**3a**) (30.1 mg, 0.10 mmol, 1.0 eq.) in toluene (0.25 ml, 0.40 M). The reaction was stirred at rt for 6 h, then quenched by sequential addition of 1N NaOH and brine. The product was extracted with Et_2O three times and the combined organic layers were dried over anhydrous $MgSO_4$, filtered and concentrated under reduced pressure to afford 3,3-diethyl-2-(4-methoxyphenyl)azetidine (**5**) as a colourless oil (20.8 mg, 95%).

^{1}H NMR (600 MHz, $CDCl_3$) δ 7.34 – 7.28 (m, 2H), 6.89 – 6.83 (m, 2H), 4.67 (s, 1H), 3.80 (s, 3H), 3.37 (dd, *J* = 7.2, 0.9 Hz, 1H), 3.11 (d, *J* = 7.1 Hz, 1H), 2.30 – 2.12 (m, 1H), 1.74 (dq, *J* = 14.9, 7.5 Hz, 1H), 1.65 (dq, *J* = 14.9, 7.5 Hz, 1H), 1.44 (dq, *J* = 14.9, 7.5 Hz, 1H), 1.15 (dq, *J* = 14.6, 7.3 Hz, 1H), 0.88 (t, *J* = 7.5 Hz, 3H), 0.54 (t, *J* = 7.5 Hz, 3H).

^{13}C NMR (151 MHz, $CDCl_3$) δ 158.6, 134.0, 128.3, 113.4, 68.8, 55.4, 53.7, 47.3, 29.9, 24.4, 8.5, 7.5;

HRMS (ESI) *m/z*; $C_{14}H_{22}NO$ requires 220.1696 ($[M+H]^+$), found 220.1690.

IR (thin film, ν_{max} /cm^{-1}) 2964, 2937, 2863, 1614, 1515, 1459, 1423, 1301, 1251, 1213, 1180, 1037, 835, 743, 623.

Telescoped amide coupling

Benzyl ((*S**)-1-((*R**)-3,3-diethyl-2-(4-methoxyphenyl)azetidin-1-yl)-1-oxo-3-phenylpropan-2-yl)carbamate (6) and Benzyl ((*S**)-1-((*S**)-3,3-diethyl-2-(4-methoxyphenyl)azetidin-1-yl)-1-oxo-3-phenylpropan-2-yl)carbamate (6')

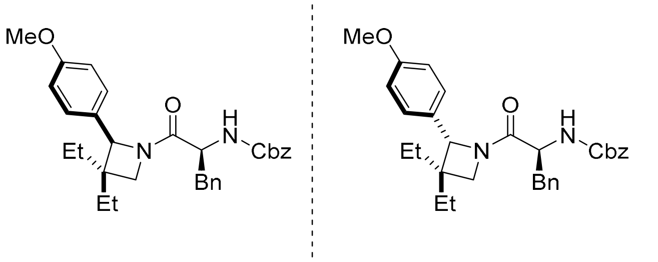

Sodium bis(2-methoxyethoxy)aluminium hydride (0.14 ml, 0.50 mmol, 60 wt% in toluene, 5.0 eq.) was added to a stirred solution of 3,3-diethyl-2-(4-methoxyphenyl)azetidine-1-sulfonyl fluoride (**3a**) (30.1 mg, 0.10 mmol, 1.0 eq.) in toluene (0.25 ml, 0.40 M). The reaction was stirred at rt for 6 h. The reaction was then quenched by sequential addition of 1N NaOH and brine. The reaction mixture was extracted with Et_2O three times and the combined organic layers were dried over anhydrous $MgSO_4$, filtered and concentrated under reduced pressure. The crude residue dissolved in DMF (0.25 ml) and added as a solution to a separate solution of HATU (84 mg, 0.22 mmol, 2.2 eq.), DIPEA (58 µl, 0.33 mmol, 3.3 eq.) and Z-Phe-OH (33 mg , 0.11 mmol, 1.1 eq.) in DMF. The reaction was stirred at rt for 16 h, then diluted with water and extracted with EtOAc three times. The combined organic layers were dried over anhydrous $MgSO_4$, filtered and concentrated under reduced pressure. Purification by flash column chromatography (0 – 30% EtOAc in pentane) afforded an inseparable mixture of diastereomers benzyl ((*S**)-1-((*R**)-3,3-diethyl-2-(4-methoxyphenyl)azetidin-1-yl)-1-oxo-3-phenylpropan-2-yl)carbamate (**6**)

and benzyl ((*S**)-1-((*R**)-3,3-diethyl-2-(4-methoxyphenyl)azetidin-1-yl)-1-oxo-3-phenylpropan-2-yl)carbamate (**6'**) as a colourless oil (43.4 mg, 87%).

Mixture of 2 diastereomers each having 2 rotamers leading to complex spectrum. VT NMR was unable to reach the high temperature regime and simplify the spectrum.

R_f (20% EtOAc in pentane) = 0.16.

^{1}H NMR (600 MHz, $CDCl_3$) δ 7.42 – 7.22 (m, 32H), 7.20 (dd, *J* = 13.1, 7.6 Hz, 2H), 7.11 (dt, *J* = 14.7, 7.2 Hz, 3H), 7.00 (d, *J* = 8.3 Hz, 2H), 6.90 (d, *J* = 8.2 Hz, 3H), 6.81 (t, *J* = 8.3 Hz, 4H), 6.76 (d, *J* = 8.2 Hz, 1H), 6.67 (d, *J* = 8.2 Hz, 1H), 6.61 (d, *J* = 7.2 Hz, 2H), 5.59 (t, *J* = 9.7 Hz, 2H), 5.56 (d, *J* = 7.0 Hz, 1H), 5.31 (s, 1H), 5.24 (d, *J* = 8.6 Hz, 1H), 5.15 (s, 1H), 5.03 (d, *J* = 12.5 Hz, 1H), 5.00 (s, 3H), 4.93 (s, 1H), 4.75 (s, 1H), 4.63 (td, *J* = 9.2, 5.5 Hz, 1H), 4.48 (td, *J* = 9.5, 5.4 Hz, 1H), 4.17 (td, *J* = 9.1, 4.9 Hz, 1H), 4.06 (d, *J* = 10.2 Hz, 2H), 3.85 (s, 1H), 3.83 (s, 3H), 3.78 (s, 4H), 3.76 (s, 3H), 3.69 (d, *J* = 8.0 Hz, 1H), 3.63 (d, *J* = 9.8 Hz, 1H), 3.55 (d, *J* = 9.8 Hz, 1H), 3.40 (d, *J* = 9.8 Hz, 1H), 3.29 (d, *J* = 8.1 Hz, 1H), 3.10 (dd, *J* = 13.2, 5.5 Hz, 1H), 3.00 (tt, *J* = 12.4, 8.0 Hz, 3H), 2.83 (dd, *J* = 22.4, 10.2 Hz, 2H), 2.77 – 2.71 (m, 2H), 2.58 (dd, *J* = 13.8, 9.5 Hz, 1H), 1.81 (s, 1H), 1.75 (h, *J* = 7.0 Hz, 2H), 1.66 (q, *J* = 7.4 Hz, 1H), 1.42 (dq, *J* = 14.4, 7.3 Hz, 1H), 1.26 (s, 4H), 1.21 – 1.08 (m, 2H), 0.97 (dq, *J* = 13.3, 6.9 Hz, 1H), 0.91 (t, *J* = 7.3 Hz, 3H), 0.87 (t, *J* = 7.6 Hz, 2H), 0.86 – 0.79 (m, 1H), 0.72 (t, *J* = 7.4 Hz, 3H), 0.62 (t, *J* = 7.3 Hz, 4H), 0.55 (t, *J* = 7.4 Hz, 3H), 0.46 (t, *J* = 7.5 Hz, 6H), 0.43 (d, *J* = 7.4 Hz, 2H).

^{13}C NMR (151 MHz, $CDCl_3$) δ 173.7, 171.8, 171.6, 170.8, 159.5, 159.3, 158.8, 155.9, 155.8, 154.9, 137.0, 136.8, 136.6, 136.6, 136.6, 136.5, 136.5, 136.5, 136.4, 136.3, 130.0, 129.7, 129.5, 129.2, 128.8, 128.8, 128.8, 128.7, 128.6, 128.6, 128.5, 128.4, 128.2, 128.1, 128.0, 127.9, 127.8, 127.7, 127.4, 127.2, 127.1, 127.1, 126.7, 114.2, 114.0, 113.7, 113.6, 73.6, 72.3, 70.7, 70.6, 67.0, 66.9, 66.8, 66.5, 59.2, 58.3, 56.7, 56.4, 55.5, 55.3, 55.3, 54.2, 53.7, 52.6, 52.5, 51.8, 51.7, 43.1, 42.7, 42.5, 42.3, 41.2, 40.3, 39.6, 39.5, 38.1, 29.8, 29.3, 28.9, 28.8, 24.5, 24.3, 24.0, 24.0, 8.1, 8.1, 8.0, 8.0, 7.5, 7.4, 7.2.

HRMS (ESI) m/*z;* $C_{31}H_{36}N_2O_4$ requires 501.2748 ($[M+H]^+$), found 501.2752.

IR (thin film, v_{max} /cm^{-1}) 3271, 2964, 2876, 1719, 1648, 1514, 1456, 1251, 1177, 1033, 838, 741, 701.

HPLC(retention time) 11.9 and 12.0 minutes.

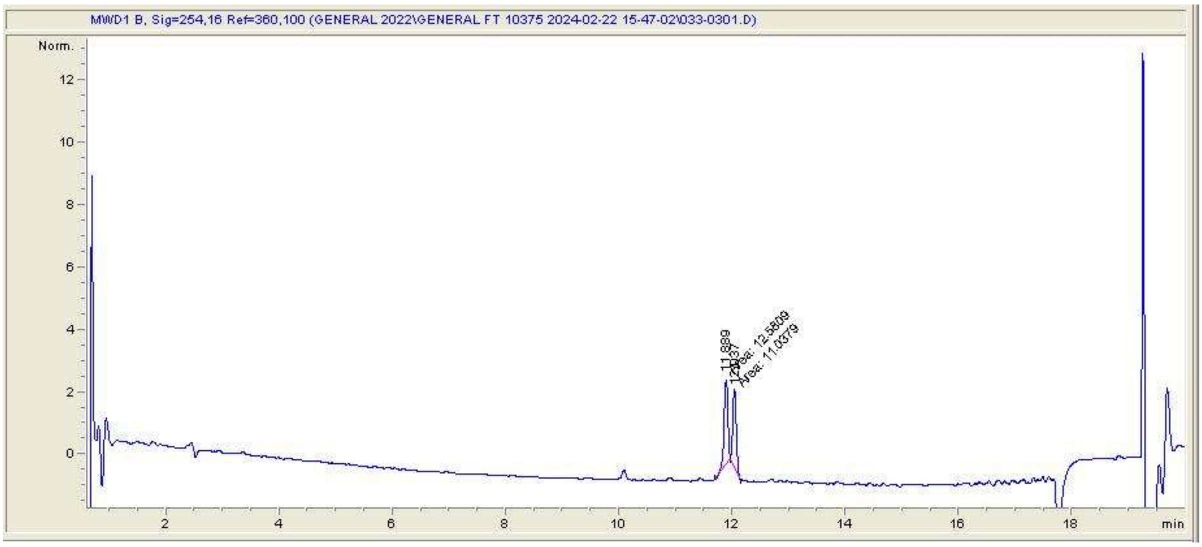

Calcium Bistriflimide-mediated SuFEx

3-(4-((3,3-Diethyl-2-(4-methoxyphenyl)azetidin-1-yl)sulfonyl)piperazin-1-yl)benzo[d]isothiazole (7)

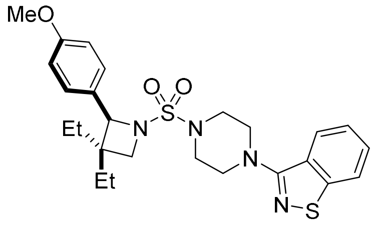

A solution of 3,3-diethyl-2-(4-methoxyphenyl)azetidine-1-sulfonyl fluoride (**3a**) (0.50 ml, 0.20 M, 0.10 mmol, 1.0 eq.) in *t*-amylOH was added to a vial containing 3-(piperazin-1-yl)benzo[d]isothiazole (43.9 mg, 0.20 mmol, 2.0 eq.) and $Ca(NTf_2)_2$ (60.0 mg, 0.10 mmol, 1.0 eq.) under N_2. The reaction was then heated at 90 °C for 20 h and then cooled to rt, diluted with EtOAc and partitioned over brine. The combined organic fractions were dried over anhydrous $MgSO_4$, filtered and concentrated under reduced pressure. Purification by flash column chromatography (0 – 5% Et_2O in pentane) afforded 3-(4-((3,3-diethyl-2-(4-methoxyphenyl)azetidin-1-yl)sulfonyl)piperazin-1-yl)benzo[d]isothiazole (**7**) as a colourless oil (34.3 mg, 68%).

R_f (10% EtOAc in pentane) = 0.26.

^{1}H NMR (400 MHz, $CDCl_3$) δ 7.81 (d, *J* = 8.1 Hz, 1H), 7.78 (d, *J* = 8.2 Hz, 1H), 7.47 (t, *J* = 7.5 Hz, 1H), 7.37 (d, *J* = 7.7 Hz, 1H), 7.32 (dd, *J* = 7.7, 5.7 Hz, 2H), 6.87 (d, *J* = 8.5 Hz, 2H), 4.94 (s, 1H), 3.77 (s, 3H), 3.75 (d, *J* = 7.7 Hz, 1H), 3.43 (dd, *J* = 6.2, 3.7 Hz, 5H), 3.40 – 3.26 (m, 4H), 1.75 (dq, *J* 14.5, 7.3 Hz, 1H), 1.71 (dq, *J* = 14.5, 7.3 Hz, 1H), 1.46 (dq, *J* = 14.6, 7.3 Hz, 1H), 1.17 (dq, *J* = 14.5, 7.3 Hz, 1H), 0.87 (t, *J* = 7.4 Hz, 3H), 0.55 (t, *J* = 7.4 Hz, 3H).

^{13}C NMR (101 MHz, $CDCl_3$) δ 163.2, 159.4, 152.9, 129.8, 128.8, 127.9, 127.8, 124.2, 123.7, 120.8, 113.7, 73.2, 58.7, 55.4, 49.7, 45.7, 42.5, 29.3, 24.5, 8.0, 7.5.

HRMS (ESI) *m/z*; $C_{25}H_{32}N_4O_3S_2Na$ requires 523.1808 ($[M+Na]^+$), found 523.1834.

IR (thin film, v_{max} /cm^{-1}) 2963, 2878, 1734, 1613, 1513, 1494, 1423, 1250, 1158, 1070, 946, 911, 838, 763, 741, 648.

RuO_4 oxidative cleavage of aromatic group

Methyl 3,3-diethyl-1-(fluorosulfonyl)azetidine-2-carboxylate (8)

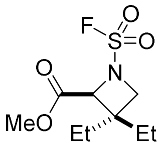

3,3-diethyl-2-(4-methoxyphenyl)azetidine-1-sulfonyl fluoride (**3a**) (60.3 mg, 0.2 mmol, 1.0 eq.) was dissolved in a mixture of MeCN and EtOAc (1:1, 2.0 ml total, 0.10 M). This mixture was added to a suspension of $NaIO_4$ (830 mg, 3.80 mmol, 19.4 eq.) in H_2O (3.0 ml) and cooled to 10 °C to which the solution of sulfamoyl fluoride was added to. $RuCl_3 \cdot xH_2O$ was then added in one portion and the solution turned dark brown. After 1 h, the reaction was slowly warmed to rt and further stirred for 2h, at which point the solution turned pale yellow. The resulting thick suspension was decanted and the white residue washed with copious EtOAc. Et_2O (5 ml) was added to the combined organic fractions and, this solution was stirred for a further 30 minutes turning the reaction mixture from pale-yellow solution to dark brown. The suspension was then filtered through Celite and washed with EtOAc. The

filtrate was washed with 20% NaCl solution, dried over anhydrous $MgSO_4$, filtered and concentrated under reduced pressure to give the crude acid.

$TMSCH_2N_2$ (0.13 ml, 0.26 mmol, 2.0 M in hexane, 1.3 eq.) was added to a solution of acid in a 4:1 mixture of toluene and MeOH (2.0 ml total, 0.10 M). The reaction was stirred for 30 minutes at rt and subsequently quenched with 10% AcOH in water (v/v). The phases were then partitioned and the aqueous phase was washed with Et_2O thrice. The combined organic fractions were washed with aq. sat. $NaHCO_3$, dried over anhydrous $MgSO_4$ and concentrated under reduced pressure. Purification by flash column chromatography (0 – 5% Et_2O in pentane) afforded methyl 3,3-diethyl-1-(fluorosulfonyl)azetidine-2-carboxylate (**8**) as a colourless oil (23.6 mg, 47%).

R*f* (5% Et_2O in pentane) = 0.25.

^{1}H NMR (400 MHz, $CDCl_3$) δ 4.58 (d, *J* = 2.4 Hz, 1H), 3.80 (s, 3H), 3.78 (app. t, *J* = 2.0 Hz, 2H), 1.81 – 1.70 (m, 2H), 1.68 – 1.57 (m, 2H), 0.95 (t, *J* = 7.5 Hz, 3H), 0.80 (t, *J* = 7.4 Hz, 3H).

^{13}C NMR (101 MHz, $CDCl_3$) δ 167.7, 70.6, 60.0, 52.6, 43.2, 28.9, 23.8, 7.8, 7.4.

^{19}F NMR (377 MHz, $CDCl_3$) δ 36.13 (s).

HRMS (ESI) *m/z* $C_9H_{16}FNO_4SNa$ requires 276.0676 ($[M+Na]^+$), found 276.0686.

IR (thin film, v_{max} /cm^{-1}) 2974, 1762, 1740, 1460, 1429, 1294, 1221, 1116, 758, 625.

Telescoped Suzuki coupling

3,3-Diethyl-2-(4-methoxy-3-(quinolin-3-yl)phenyl)azetidine-1-sulfonyl fluoride (9)

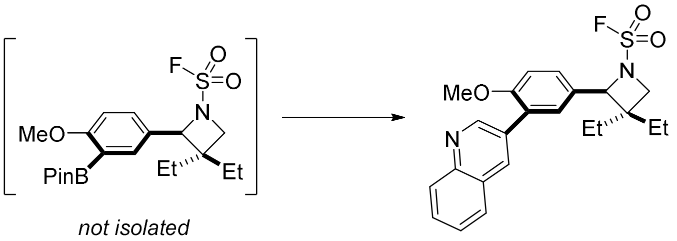

Prepared according to a modification of **GP1** using (4-Methoxy-3-(4,4,5,5-tetramethyl-1,3,2-dioxaborolan-2-yl)benzylidene)sulfamoyl fluoride (**I-19**) (68.6 mg, 0.20 mmol, 1.0 eq.), 3-F-TX (4.6 mg, 0.020 mmol, 0.10 eq.) and 2-ethyl-1-butene (241 µL, 2.0 mmol, 10 eq.) in $(CH_2Cl)_2$ (1.0 mL, 0.20 M). Following the photochemical reaction, the mixture was transferred to a round bottom flask and concentrated under reduced pressure. $Pd(PPh_3)_4$ (4.6 mg, 0.004 mmol, 2 mol%), 3-bromoquinoline (42 µL, 0.3 mmol, 1.5 eq.) and K_2CO_3 (415 mg, 3.0 mmol, 15 eq.) was added to the crude reaction mixture and the vial was capped with a septum. A 2:1 $MeCN/H_2O$ mixture was added *via* syringe (3.0 ml total, 0.067 M) and the flask was sparged with nitrogen for ten minutes, then heated at 80 °C for 16 h. The reaction was then cooled to rt, filtered through celite and the filtrate was washed with aq. sat. $NaHCO_3$. The aqueous phase was then washed with EtOAc three times and the combined organic layers were dried over anhydrous $MgSO_4$, filtered and then concentrated under reduced pressure. Purification by flash column chromatography (0 – 40% Et_2O in pentane) to give 3,3-diethyl-2-(4-methoxy-3-(quinolin-3-yl)phenyl)azetidine-1-sulfonyl fluoride (**9**) as a mixture with pinacol.

To remove pinacol the residue obtained was redissolved in a 1:1 mixture of $MeOH/H_2O$ (10 ml) and concentrated under reduced pressure. This process was repeated a further 4 times to remove all

pinacol *via* azeotropic evaporation. This afforded 3,3-diethyl-2-(4-methoxy-3-(quinolin-3-yl)phenyl)azetidine-1-sulfonyl fluoride (**9**) as a yellow solid (39.8 mg, 46%).

R*f* (50% Et_2O in pentane) = 0.34.

^{1}H NMR (600 MHz, $CDCl_3$) δ 9.11 (s, 1H), 8.25 (d, *J* = 2.3 Hz, 1H), 8.14 (d, *J* = 8.4 Hz, 1H), 7.87 (d, *J* = 8.1 Hz, 1H), 7.76 – 7.69 (m, 1H), 7.59 – 7.53 (m, 1H), 7.41 (dd, *J* = 8.5, 2.3 Hz, 1H), 7.38 (d, *J* = 2.3 Hz, 1H), 7.04 (d, *J* = 8.5 Hz, 1H), 3.87 (d, *J* = 8.0 Hz, 1H), 3.85 (s, 3H), 3.75 (d, *J* = 7.9 Hz, 1H), 1.82 (dq, *J* = 14.6, 7.3 Hz, 1H), 1.77 (dq, *J* = 14.6, 7.4 Hz, 1H), 1.46 (dq, *J* = 14.5, 7.3 Hz, 1H), 1.28 – 1.20 (m, 1H), 0.96 (t, *J* = 7.4 Hz, 3H), 0.63 (t, *J* = 7.4 Hz, 3H).

^{13}C NMR (151 MHz, $CDCl_3$) δ 156.8, 152.0, 147.1, 135.7, 131.4, 129.5, 129.4, 129.3, 128.2, 128.1, 127.9, 127.2, 126.8, 111.3, 75.1, 60.0, 55.8, 43.8, 29.2, 24.5, 8.2, 7.4.

^{19}F NMR (565 MHz, $CDCl_3$) δ 31.42 (s).

HRMS (ESI) *m/z* $C_{23}H_{26}FN_2O_3S$ requires 429.1643 ($[M+H]^+$), found 429.1647.

IR (thin film, ν_{max} /cm^{-1}) 2966, 1505, 1463, 1421, 1255, 1213, 1179, 1028, 742, 643, 622.

m.p. 120 – 122 °C.

Substrate Synthesis

Synthesis of sulfamoyl fluoride imines (GP2)

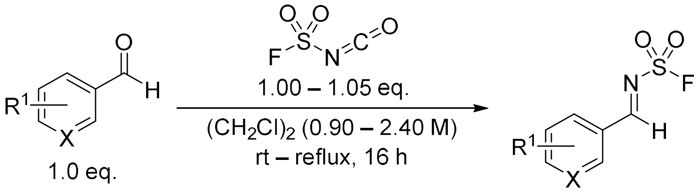

Fluorosulfonyl isocyanate (FSI) (1.00-1.80 eq.) was added dropwise to a stirred solution of aldehyde (1.00 eq.) in dry $(CH_2Cl)_2$ (0.9 – 2.4 M) under nitrogen at 0 °C or rt. (Caution: CO_2 evolution). The reaction was warmed to (rt – reflux) and stirred for a further 16 h. The solvent was removed under reduced pressure to give the crude product, which was either recrystalised or triturated to give the desired product.

Imine product characterisation

(4-Methoxybenzylidene)sulfamoyl fluoride (1)

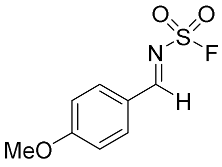

Prepared according to **GP2**, FSI (1.00 ml, 12.72 mmol, 1.05 eq.) was added dropwise to a stirred solution of anisaldeyde (1.50 ml, 12.12 mmol, 1.00 eq.) in dry $(CH_2Cl)_2$ (5.0 ml, 2.4 M) under N_2 at 0 °C. (Caution: CO_2 evolution). The reaction was warmed to rt and stirred for a further 16 h. The solvent was removed under reduced pressure to give (4-methoxybenzylidene)sulfamoyl fluoride (**1**), which was washed with hexane and isolated as an off-white solid (2.45 g, 93%).

^{1}H NMR (400 MHz, $CDCl_3$) δ 8.98 (s, 1H), 8.06 – 7.97 (m, 2H), 7.12 – 7.04 (m, 2H), 3.95 (s, 3H).

^{13}C NMR (101 MHz, $CDCl_3$) δ 176.0 (d, $^3J_{C-F}$ = 3.0 Hz), 167.2, 135.2, 124.0, 115.3, 56.0.

^{19}F NMR (377 MHz, CDCl$_3$) δ 44.48 (s).

HRMS (ESI) *m/z* $C_8H_9FNO_3S$ requires 218.0282 ([M+H]$^+$), found 218.0293.

IR (thin film, ν_{max} /cm^{-1}) 1590, 1555, 1514, 1399, 1320, 1272, 1198, 1164, 1021, 837, 791.

m.p. 75–77 °C.

UV/Vis λ_{max}(fluorobenzene)/nm 324 (ε/dm^3 mol^{-1}cm^{-1} 9 420).

(2-Methoxybenzylidene)sulfamoyl fluoride (I-2)

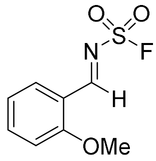

Prepared according to **GP2**, FSI (0.20 ml, 2.56 mmol, 1.05 eq.) was added dropwise to a stirred solution of o-anisaldeyde (302 µl, 2.44 mmol, 1.00 eq.) in dry $(CH_2Cl)_2$ (2.0 ml, 1.2 M) under N_2 at 0 °C. (Caution: CO_2 evolution). The reaction was warmed to rt and stirred for a further 16 h. The solvent was removed under reduced pressure to give (2-methoxybenzylidene)sulfamoyl fluoride (**I-2**), which was washed with hexane and isolated as a light-yellow solid (340 mg, 64%).

^{1}H NMR (400 MHz, CDCl$_3$) δ 9.58 (s, 1H), 8.12 (dd, *J* = 7.9, 1.8 Hz, 1H), 7.71 (ddd, *J* = 8.9, 7.3, 1.8 Hz, 1H), 7.12 – 7.03 (m, 1H), 7.02 (d, *J* = 8.5 Hz, 1H), 3.96 (s, 3H).

^{13}C NMR (101 MHz, CDCl$_3$) δ 173.6 (d, $^3J_{C-F}$ = 3.8 Hz), 163.0, 139.5, 129.9, 121.5, 119.9, 112.0, 56.1.

^{19}F NMR (377 MHz, CDCl$_3$) δ 43.82 (s).

HRMS (ESI) *m/z* $C_8H_9FNO_3S^+$ requires 218.0282 ([M+H]$^+$), found 218.0290.

IR (thin film, ν_{max} /cm^{-1}) 2924, 1606, 1590, 1564, 1488, 1405, 1264, 1201, 1165, 848, 798, 769, 636.

m.p. 50 – 52 °C.

(2-Fluoro-6-methoxybenzylidene)sulfamoyl fluoride (I-3)

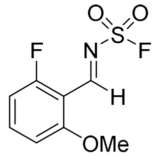

Prepared according to **GP2**, FSI (0.16 ml, 2.00 mmol, 1.05 eq.) was added dropwise to a stirred solution of 2-fluoro-6-methoxybenzaldehyde (293 mg, 1.90 mmol, 1.00 eq.) in dry $(CH_2Cl)_2$ (2.0 ml, 1.0 M) under N_2 at 0 °C. (Caution: CO_2 evolution). The reaction was warmed to rt and stirred for a further 16 h. The solvent was removed under reduced pressure to give (2-fluoro-6-methoxybenzylidene)sulfamoyl fluoride (**I-3**), which was washed with hexane and isolated as an orange solid (416 mg, 93%).

^{1}H NMR (400 MHz, CDCl$_3$) δ 9.95 (d, *J* = 1.5 Hz, 1H), 8.12 (td, *J* = 8.5, 6.2 Hz, 1H), 7.34 – 7.30 (m, 1H), 7.30 – 7.27 (m, 1H), 4.45 (s, 3H).

^{13}C NMR (101 MHz, CDCl$_3$) δ 170.9 (d, $^3J_{C-F}$ = 4.1 Hz), 164.0 (d, $^1J_{C-F}$ = 269.0 Hz), 163.3 (d, $^3J_{C-F}$ = 4.8 Hz), 139.5 (d, $^2J_{C-F}$ = 12.3 Hz), 109.6 (d, $^3J_{C-F}$ = 10.3 Hz), 109.2 (d, $^2J_{C-F}$ = 21.0 Hz), 107.6 (d, $^4J_{C-F}$ = 3.6 Hz), 56.9.

^{19}F NMR (377 MHz, CDCl$_3$) δ 43.16 (s), -102.13 – -102.33 (m).

HRMS (ESI) *m/z* $C_8H_8F_2NO_3S^+$ requires 236.0188 ($[M+H]^+$), found 236.0189.

IR (thin film, v_{max} /cm^{-1}) 2925, 1620, 1566, 1481, 1407, 1378, 1291, 1256, 1202, 1091, 834, 790.

m.p. 84 – 87 °C.

Benzylidenesulfamoyl fluoride (I-4)

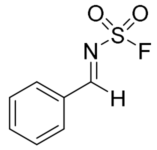

Prepared according to **GP2**, FSI (0.16 ml, 2.00 mmol, 1.05 eq.) was added dropwise to a stirred solution of benzaldehyde (202 µl, 1.90 mmol, 1.00 eq.) in dry $(CH_2Cl)_2$ (2.0 mL, 1.0 M) under nitrogen at 0 °C. (Caution: CO_2 evolution). The reaction was warmed to rt and stirred for a further 16 h. The solvent was removed under reduced pressure to give benzylidenesulfamoyl fluoride (**I-4**), which was washed with hexane and isolated as a white solid (257mg, 72%).

^{1}H NMR (400 MHz, CDCl$_3$) δ 9.10 (s, 1H), 8.06 – 7.99 (m, 2H), 7.82 – 7.73 (m, 1H), 7.66 – 7.52 (m, 2H).

^{13}C NMR (101 MHz, CDCl$_3$) δ 177.7 (d, $^3J_{C-F}$ = 3.9 Hz), 137.2, 132.5, 131.3, 129.8.

^{19}F NMR (377 MHz, CDCl$_3$) δ 43.65 (s).

IR (thin film, v_{max} /cm^{-1}) 2924, 1598, 1572, 1455, 1409, 1377, 1206, 870, 846, 768, 687, 641.

m.p.: 45–46 °C.

Data consistent with literature.[38]

(4-Methylbenzylidene)sulfamoyl fluoride (I-5)

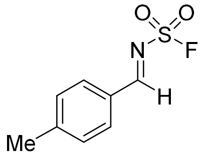

Prepared according to **GP2**, FSI (0.15 ml, 1.92 mmol, 1.05 eq.) was added dropwise to a stirred solution of 4-methylbenzaldehyde (220 mg, 1.83 mmol, 1.00 eq.) in dry $(CH_2Cl)_2$ (2.0 mL, 0.9 M) under nitrogen at 0 °C. (Caution: CO_2 evolution). The reaction was warmed to rt and stirred for a further 16 h. The solvent was removed under reduced pressure to give (4-methylbenzylidene)sulfamoyl fluoride (**I-5**), which was recrystalised from Et_2O, as a white solid (245 mg, 67%).

^{1}H NMR (400 MHz, CDCl$_3$) δ 9.05 (s, 1H), 7.92 (d, *J* = 8.3 Hz, 2H), 7.39 (d, *J* = 7.9 Hz, 2H), 2.50 (s, 3H).

^{13}C NMR (101 MHz, CDCl$_3$) δ 177.3 (d, $^3J_{C-F}$ = 3.7 Hz), 149.3, 132.6, 130.6, 128.8, 22.4.

^{19}F NMR (377 MHz, CDCl$_3$) δ 43.89 (s).

IR (neat, v_{max} /cm^{-1}) 3066, 1593, 1559, 1512, 1418, 1393, 1196, 1178, 874, 782, 762.

m.p. 77 – 79 °C.

Data consistent with literature.[39]

(4-(Trimethylsilyl)benzylidene)sulfamoyl fluoride (I-6)

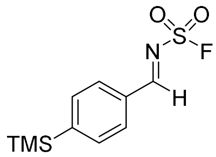

Prepared according to **GP2**, FSI (0.16 ml, 2.00 mmol, 1.05 eq.) was added dropwise to a stirred solution of 4-trimethylsilylbenzaldehyde (**A-21**) (342 µl, 1.90 mmol, 1.00 eq.) in dry $(CH_2Cl)_2$ (2.0 ml, 1.0 M) under N_2 at 0 °C. (Caution: CO_2 evolution). The reaction was warmed to rt and stirred for a further 16 h. The solvent was removed under reduced pressure to give (4-(trimethylsilyl)benzylidene)sulfamoyl fluoride (**I-6**), which was washed with pentane and isolated as a white solid (280 mg, 57%).

^{1}H NMR (400 MHz, $CDCl_3$) δ 9.09 (s, 1H), 8.00 – 7.93 (m, 2H), 7.78 – 7.69 (m, 2H), 0.33 (s, 9H).

^{13}C NMR (101 MHz, $CDCl_3$) δ 177.9 (d, $^3J_{C-F}$ = 3.8 Hz), 153.4, 134.5, 131.3, 131.1, -1.4**.**

^{19}F NMR (377 MHz, $CDCl_3$) δ 43.74 (s).

IR (thin film, ν_{max} /cm^{-1}) 2957, 1595, 1545, 1412, 1252, 106, 1186, 1105, 845, 794, 761, 716, 624;

m.p. 59 – 60 °C.

(4-Fluorobenzylidene)sulfamoyl fluoride (I-7)

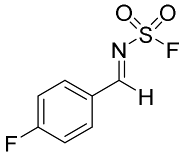

Prepared according to **GP2**, FSI (0.20 ml, 2.56 mmol, 1.05 eq.) was added dropwise to a stirred solution of 4-fluorobenzaldehyde (275 µl, 2.44 mmol, 1.00 eq.) in dry $(CH_2Cl)_2$ (2.0 mL, 1.2 M) under N_2 at 0 °C. (Caution: CO_2 evolution). The reaction was warmed to rt and stirred for a further 16 h. The solvent was removed under reduced pressure to give (4-fluorobenzylidene)sulfamoyl fluoride (**I-7**), which was recrystalised successively from CPME and MTBE as a white solid (160 mg, 32%).

^{1}H NMR (400 MHz, $CDCl_3$) δ 9.07 (s, 1H), 8.14 – 8.03 (m, 2H), 7.34 – 7.24 (m, 2H).

^{13}C NMR (101 MHz, $CDCl_3$) δ 176.0 (d, $^3J_{C-F}$ = 3.8 Hz), 168.3 (d, $^1J_{C-F}$ = 262.3 Hz), 135.2 (d, $^3J_{C-F}$ = 10.3 Hz), 127.8, 117.5 (d, $^2J_{C-F}$ = 22.5 Hz).

^{19}F NMR (377 MHz, $CDCl_3$) δ 43.80, -96.70.

IR (thin film, ν_{max} /cm^{-1}) 2925, 1604, 1578, 1513, 1420, 1402, 1250, 1202, 1158, 881, 858, 805, 777.

m.p. 66 – 68 °C.

(4-Chlorobenzylidene)sulfamoyl fluoride (I-8)

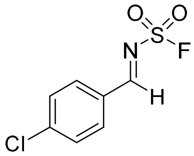

Prepared according to **GP2**, FSI (0.54 ml, 6.91 mmol, 1.80 eq.) was added dropwise to a stirred solution of 4-chlorobenzaldehyde (540 mg, 3.84 mmol, 1.00 eq.) in dry $(CH_2Cl)_2$ (2.0 mL, 1.9 M) under N_2 at 0 °C. (Caution: CO_2 evolution). The reaction was heated to 50 °C and stirred for a further 16 h. The

solvent was removed under reduced pressure to give (4-chlorobenzylidene)sulfamoyl fluoride (**I-8**), which was recrystalised from MTBE (313 mg, 37%).

^{1}H NMR (400 MHz, $CDCl_3$) δ 9.07 (s, 1H), 8.01 – 7.94 (m, 2H), 7.62 – 7.54 (m, 2H).

^{13}C NMR (101 MHz, $CDCl_3$) δ 176.2 (d, $^3J_{C\text{-}F}$ = 3.9 Hz), 144.1, 133.5, 130.3, 129.7.

^{19}F NMR (377 MHz, $CDCl_3$) δ 43.77.

IR (neat, ν_{max} /cm^{-1}) 3092, 1590, 1559, 1489, 1412, 1200, 1089, 841, 831, 697.

m.p. 80 – 82 °C.

(4-Bromobenzylidene)sulfamoyl fluoride (I-9)

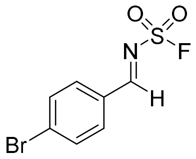

Prepared according to **GP2**, FSI (0.16 ml, 2.00 mmol, 1.05 eq.) was added dropwise to a stirred solution of 4-bromobenzaldehyde (352 mg, 1.90 mmol, 1.00 eq.) in dry $(CH_2Cl)_2$ (2.0 mL, 1.0 M) under N_2 at rt. (Caution: CO_2 evolution). The reaction was heated to reflux and stirred for a further 16 h. The solvent was removed under reduced pressure to approximately ~50% original volume and triturated with heptane to give (4-bromobenzylidene)sulfamoyl fluoride (**I-9**) as a white solid (232 mg, 46%).

^{1}H NMR (400 MHz, $CDCl_3$) δ 9.06 (s, 1H), 7.93 – 7.84 (m, 2H), 7.79 – 7.71 (m, 2H).

^{13}C NMR (101 MHz, $CDCl_3$) δ 176.4 (d, $^3J_{C\text{-}F}$ = 3.8 Hz), 133.4, 133.3, 133.1, 130.1.

^{19}F NMR (376 MHz, $CDCl_3$) δ 43.76 (s).

IR (neat, ν_{max} /cm^{-1}) 1586, 1556, 1485, 1220, 1069, 1011, 838, 674.

m.p. 98 – 100 °C.

([1,1'-Biphenyl]-4-ylmethylene)sulfamoyl fluoride (I-10)

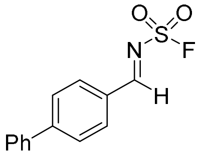

Prepared according to **GP2**, FSI (0.16 ml, 2.00 mmol, 1.05 eq.) was added dropwise to a stirred solution of 4-phenylbenzaldehyde (346 mg, 1.90 mmol, 1.00 eq.) in dry $(CH_2Cl)_2$ (2.0 ml, 1.0 M) under nitrogen at 0 °C. (Caution: CO_2 evolution). The reaction was warmed to rt and stirred for a further 16 h. The solvent was removed under reduced pressure to give ([1,1'-biphenyl]-4-ylmethylene)sulfamoyl fluoride (**I-10**), which was washed with hexane and isolated as a white solid (397mg, 79%).

^{1}H NMR (400 MHz, CDCl3) δ 9.13 (s, 1H), 8.13 – 8.06 (m, 2H), 7.85 – 7.78 (m, 2H), 7.72 – 7.62 (m, 2H), 7.56 – 7.42 (m, 3H).

^{13}C NMR (101 MHz, CDCl3) δ 177.0 (d, $^3J_{C\text{-}F}$ = 3.7 Hz), 150.0, 139.0, 133.1, 130.0, 129.4, 129.3, 128.3, 127.6.

^{19}F NMR (377 MHz, CDCl3) δ 43.98 (s).

IR (thin film, v_{max} /cm^{-1}) 2925, 1594, 1556, 1412, 1213, 883, 858, 766, 631.

m.p. 110–115 °C.

(2-Methylbenzylidene)sulfamoyl fluoride (I-11)

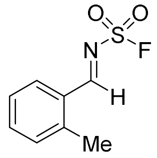

Prepared according to **GP2**, FSI (0.20 ml, 2.56 mmol, 1.05 eq.) was added dropwise to a stirred solution of 2-methylbenzaldehyde (228 µl, 1.90 mmol, 1.00 eq.) in dry $(CH_2Cl)_2$ (2.0 mL, 1.2 M) under nitrogen at 0 °C. (Caution: CO_2 evolution). The reaction was warmed to rt and stirred for a further 16 h. The ~80% solvent was removed under reduced pressure and the product was crashed out by adding heptane which was then washed with pentane to give (2-methylbenzylidene)sulfamoyl fluoride (**I-11**) as a white solid (96.1 mg, 25%).

^{1}H NMR (400 MHz, CDCl$_3$) δ 9.39 (s, 1H), 8.10 (dd, *J* = 7.9, 1.5 Hz, 1H), 7.62 (td, *J* = 7.6, 1.5 Hz, 1H), 7.42 – 7.34 (m, 2H), 2.67 (s, 3H).

^{13}C NMR (101 MHz, CDCl$_3$) δ 176.2 (d, $^3J_{C-F}$ = 3.8 Hz), 144.2, 136.9, 132.2, 132.0, 129.4, 127.2, 20.0.

^{19}F NMR (377 MHz, CDCl$_3$) δ 43.81(s).

IR (thin film, v_{max} /cm^{-1}) 1589, 1565, 1409, 1293, 1206, 851, 767.

m.p. 38 – 39 °C.

(4-(1,3-Dioxoisoindolin-2-yl)benzylidene)sulfamoyl fluoride (I-12)

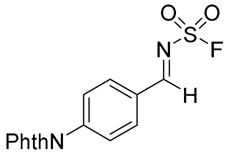

Prepared according to **GP2**, FSI (0.16 ml, 2.00 mmol, 1.05 eq.) was added dropwise to a stirred solution of 4-(1,3-dioxoisoindolin-2-yl)benzaldehyde (**A-22**) (477 mg, 1.9 mmol, 1.00 eq.) in dry $(CH_2Cl)_2$ (2.0 mL, 1.0 M) under N_2 at 0 °C. (Caution: CO_2 evolution). The reaction was heated to reflux and stirred for a further 72 h. The reaction mixture was filtered to and residue washed with copious CH_2Cl_2. Filtrate was then concentrated under reduced pressure and the compound was recrystalised from CH_2Cl_2 to give (4-(1,3-dioxoisoindolin-2-yl)benzylidene)sulfamoyl fluoride (**I-12**) as a pale-yellow solid (133 mg, 21%).

^{1}H NMR (400 MHz, CDCl$_3$) δ 9.13 (s, 1H), 8.21 – 8.12 (m, 2H), 8.06 – 7.96 (m, 2H), 7.90 – 7.80 (m, 4H).

^{13}C NMR (101 MHz, CDCl$_3$) δ 176.4 (d, $^3J_{C-F}$ = 3.8 Hz), 166.5, 139.7, 135.2, 133.1, 131.5, 129.8, 126.6, 124.4.

^{19}F NMR (377 MHz, CDCl$_3$) δ 43.88 (s).

IR (neat, v_{max} /cm^{-1}) 1719, 1700, 1590, 1560, 1413, 1219, 1089, 843, 719, 697.

m.p. 226 °C (decomp.).

4-(((Fluorosulfonyl)imino)methyl)phenyl pivalate (I-13)

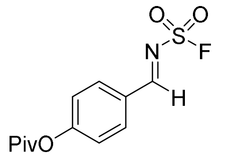

Prepared according to **GP2**, FSI (0.16 ml, 2.00 mmol, 1.05 eq.) was added dropwise to a stirred solution of 4-formyl pivalate (**A-23**) (392 mg, 1.90 mmol, 1.00 eq.) in dry $(CH_2Cl)_2$ (2.0 mL, 1.0 M) under N_2 at 0 °C. (Caution: CO_2 evolution). The reaction was heated to 35 °C and stirred for a further 16 h. The solvent was removed under reduced pressure to give 4-(((fluorosulfonyl)imino)methyl)phenyl pivalate (**I-13**), which was washed with hexane and isolated as an orange solid (302 mg, 55%).

^{1}H NMR (400 MHz, CDCl$_3$) δ 9.07 (s, 1H), 8.10 – 8.02 (m, 2H), 7.36 – 7.28 (m, 2H), 1.38 (s, 9H).

^{13}C NMR (101 MHz, CDCl$_3$) δ 176.4 (d, $^3J_{C\text{-}F}$ = 3.8 Hz), 176.2, 158.2, 134.0, 128.5, 123.1, 39.5, 27.1.

^{19}F NMR (377 MHz, CDCl$_3$) δ 43.86 (s).

HRMS (ESI) *m/z* $C_{12}H_{15}FNO_4S^+$ requires 288.0700 ([M+H]$^+$) found 288.0705.

IR (thin film, ν_{max} /cm^{-1}) 2926, 1748, 1600, 1572, 1478, 1410, 1235, 1121, 1165, 1112, 880, 844, 776, 630.

m.p. 96 – 98 °C.

Methyl 3-(((fluorosulfonyl)imino)methyl)benzoate (I-14)

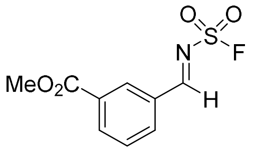

Prepared according to **GP2**, FSI (0.16 ml, 2.00 mmol, 1.05 eq.) was added dropwise to a stirred solution of Methyl 3-formylbenzoate (312 mg, 1.90 mmol, 1.00 eq.) in dry $(CH_2Cl)_2$ (2.0 mL, 1.0 M) under nitrogen at 0 °C. (Caution: CO_2 evolution). The reaction was heated to reflux and stirred for a further 16 h. The solvent was removed under reduced pressure to give the methyl 3-(((fluorosulfonyl)imino)methyl)benzoate (**I-14**), which was washed with a 15:1 pentane/Et_2O mixture and isolated as a white solid (302 mg, 55%).

^{1}H NMR (400 MHz, CDCl$_3$) δ 9.16 (s, 1H), 8.66 (t, *J* = 1.8 Hz, 1H), 8.41 (dt, *J* = 7.8, 1.5 Hz, 1H), 8.24 (dt, *J* = 7.8, 1.5 Hz, 1H), 7.70 (t, *J* = 7.8 Hz, 1H), 3.98 (s, 3H).

^{13}C NMR (101 MHz, CDCl$_3$) δ 176.7 (d, $^3J_{C\text{-}F}$ = 4.0 Hz), 165.4, 137.5, 135.5, 133.6, 132.0, 131.6, 130.0, 52.9.

^{19}F NMR (377 MHz, CDCl$_3$) δ 43.59 (s).

IR (thin film, ν_{max} /cm^{-1}) 2924, 1729, 1611, 1578, 1415, 1304, 1221, 1205, 1083, 852, 754, 646.

m.p. 97 – 100 °C.

(4-(Trifluoromethyl)benzylidene)sulfamoyl fluoride (I-15)

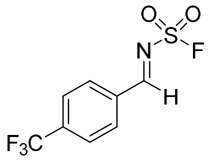

Prepared according to **GP2**, FSI (0.16 ml, 2.00 mmol, 1.00 eq.) was added dropwise to a stirred solution of 4-Trifluoromethylbenzaldehyde (254 µl, 1.90 mmol, 1.00 eq.) in dry $(CH_2Cl)_2$ (2.0 mL, 1.0 M) under N_2 at rt. (Caution: CO_2 evolution). The reaction was heated to reflux and stirred for a further 16 h. The solvent was removed under reduced pressure to approximately ~20% original volume and triturated with heptane to give the (4-(trifluoromethyl)benzylidene)sulfamoyl fluoride (**I-15**) as a white solid (337 mg, 70%).

^{1}H NMR (400 MHz, $CDCl_3$) δ 9.17 (s, 1H), 8.17 (dt, *J* = 7.9, 0.9 Hz, 2H), 7.86 (d, *J* = 8.2 Hz, 2H).

^{13}C NMR (101 MHz, $CDCl_3$) δ 176.2 (d, $^3J_{C\text{-}F}$ = 4.2 Hz), 137.7 (q, $^2J_{C\text{-}F}$ = 33.2 Hz), 134.1, 132.5, 128.7 (q, $^1J_{C\text{-}F}$ = 273.2 Hz), 126.7 (q, $^3J_{C\text{-}F}$ = 3.7 Hz).

^{19}F NMR (377 MHz, $CDCl_3$) δ 43.49 (s), -63.55(s).

IR (neat, ν_{max} /cm^{-1}) 1609, 1574, 1516, 1422, 1379, 1327, 1212, 1175, 1131, 1112, 1067, 1019, 877, 835, 785, 662.

m.p. 72 – 75 °C.

((2,2-Difluorobenzo[d][1,3]dioxol-5-yl)methylene)sulfamoyl fluoride (I-16)

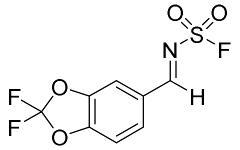

Prepared according to **GP2**, FSI (0.16 ml, 2.00 mmol, 1.00 eq.) was added dropwise to a stirred solution of 2,2-difluorobenzo[d][1,3]dioxole-5-carbaldehyde (354 mg, 1.90 mmol, 1.00 eq.) in dry $(CH_2Cl)_2$ (2.0 mL, 1.0 M) under N_2 at 0 °C. (Caution: CO_2 evolution). The reaction was heated to reflux and stirred for a further 16 h. The solvent was removed under reduced pressure to give ((2,2-difluorobenzo[d][1,3]dioxol-5-yl)methylene)sulfamoyl fluoride (**I-16**), which was washed with pentane and isolated as a white solid (195 mg, 38%).

^{1}H NMR (400 MHz, $CDCl_3$) δ 9.05 (s, 1H), 7.83 (s, 1H), 7.78 (d, *J* = 8.3 Hz, 1H), 7.29 (d, *J* = 8.2 Hz, 1H).

^{13}C NMR (101 MHz, $CDCl_3$) δ 175.8 (d, $^3J_{C\text{-}F}$ = 3.9 Hz), 150.1, 145.0, 132.5, 131.8 (t, $^1J_{C\text{-}F}$ = 260.0 Hz), 127.8, 110.6, 110.5.

^{19}F NMR (377 MHz, $CDCl_3$) δ 44.00 (s), -49.66 (s).

IR (thin film, ν_{max} /cm^{-1}) 2926, 1633, 1592, 1504, 1460, 1414, 1254, 1206, 1033, 902, 860, 788, 710.

m.p. 43 – 48 °C.

(4-(2-Bromoethoxy)benzylidene)sulfamoyl fluoride (I-17)

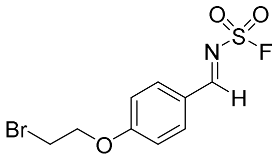

Prepared according to **GP2**, FSI (0.15 ml, 1.92 mmol, 1.05 eq.) was added dropwise to a stirred solution of 4-(2-bromoethoxy)benzaldehyde (435.3 mg, 1.90 mmol, 1.00 eq.) in dry $(CH_2Cl)_2$ (2.0 mL, 1.0 M) under N_2 at 0 °C. (Caution: CO_2 evolution). The reaction was warmed to rt and stirred for a further 16 h. The solvent was removed under reduced pressure to give the (4-(2-bromoethoxy)benzylidene)sulfamoyl fluoride (**I-17**), which was recrystalised from CH_2Cl_2/heptane and then washed with a 7:1 pentane/CH_2Cl_2 mixture to give the product as a yellow solid (182 mg, 31%).

^{1}H NMR (400 MHz, $CDCl_3$) δ 8.99 (s, 1H), 8.00 (d, *J* = 8.6 Hz, 2H), 7.07 (d, *J* = 8.5 Hz, 2H), 4.43 (t, *J* = 6.1 Hz, 2H), 3.69 (t, *J* = 6.1 Hz, 2H).

^{13}C NMR (101 MHz, $CDCl_3$) δ 175.9 (d, $^3J_{C\text{-}F}$ = 3.4 Hz), 165.4, 135.1, 124.6, 115.7, 68.3, 28.1.

^{19}F NMR (377 MHz, $CDCl_3$) δ 44.37 (s).

IR (thin film, v_{max} /cm^{-1}) 2973, 2941, 1514, 1423, 1250, 1214, 1033, 732;

m.p. 88 – 90 °C.

(2-Methoxy-5-(4,4,5,5-tetraethyl-1,3,2-dioxaborolan-2-yl)benzylidene)sulfamoyl fluoride (I-18)

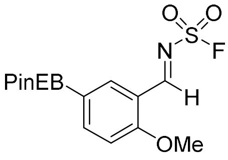

Prepared according to **GP2**, FSI (0.16 ml, 2.00 mmol, 1.05 eq.) was added dropwise to a stirred solution of 2-methoxy-5-(4,4,5,5-tetraethyl-1,3,2-dioxaborolan-2-yl)benzaldehyde (**A-24**) (605 mg, 1.90 mmol, 1.00 eq.) in dry $(CH_2Cl)_2$ (2.0 mL, 1.0 M) under N_2 at 0 °C. (Caution: CO_2 evolution). The reaction was heated to 50 °C and stirred for a further 16 h. The solvent was removed under reduced pressure to approximately ~20% and crashed out by addition of heptane to give (2-methoxy-5-(4,4,5,5-tetraethyl-1,3,2-dioxaborolan-2-yl)benzylidene)sulfamoyl fluoride (**I-18**), which was washed with pentane and isolated as a white solid (661 mg, 87%).

^{1}H NMR (400 MHz, $CDCl_3$) δ 9.58 (s, 1H), 8.56 (d, *J* = 1.7 Hz, 1H), 8.12 (dd, *J* = 8.4, 1.7 Hz, 1H), 7.00 (d, *J* = 8.5 Hz, 1H), 3.98 (s, 3H), 1.75 (ddt, *J* = 17.7, 14.4, 7.2 Hz, 8H), 0.97 (t, *J* = 7.5 Hz, 12H).

^{13}C NMR (101 MHz, $CDCl_3$) δ 173.6 (d, $^3J_{C\text{-}F}$ = 3.6 Hz), 164.9, 145.9, 137.2, 119.5, 111.2, 89.4, 56.2, 26.6, 9.0.

^{19}F NMR (377 MHz, $CDCl_3$) δ 43.93 (s).

IR (neat, v_{max} /cm^{-1}) 2982, 1612, 1584, 1401, 1362, 1314, 1192, 1140, 1024, 910, 850, 823, 803, 784, 673.

m.p. 75 – 79 °C.

(4-Methoxy-3-(4,4,5,5-tetramethyl-1,3,2-dioxaborolan-2-yl)benzylidene)sulfamoyl fluoride (I-19)

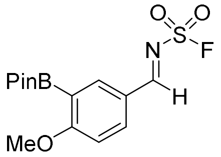

Prepared according to **GP2**, FSI (0.16 ml, 2.00 mmol, 1.00 eq.) was added dropwise to a stirred solution of 4-methoxy-3-(4,4,5,5-tetramethyl-1,3,2-dioxaborolan-2-yl)benzaldehyde (**A-25**) (497 mg, 1.90 mmol, 1.00 eq.) in dry $(CH_2Cl)_2$ (2.0 mL, 1.0 M) under N_2 at 0 °C. (Caution: CO_2 evolution). The reaction was heated to 50 °C and stirred for a further 16 h. The solvent was removed under reduced pressure to approximately ~20% original volume and the solid that crashed out was filtered off to give the (4-methoxy-3-(4,4,5,5-tetramethyl-1,3,2-dioxaborolan-2-yl)benzylidene)sulfamoyl fluoride (**I-19**), which was washed with a 15:1 pentane/Et_2O mixture and isolated as a white solid (462 mg, 71%).

^{1}H NMR (400 MHz, $CDCl_3$) δ 8.98 (s, 1H), 8.33 (d, *J* = 2.3 Hz, 1H), 8.10 (dd, *J* = 8.8, 2.4 Hz, 1H), 7.02 (d, *J* = 8.8 Hz, 1H), 3.97 (s, 3H), 1.36 (s, 12H).

^{13}C NMR (101 MHz, $CDCl_3$) δ 176.2, 171.4, 143.0, 137.6, 123.5, 111.5, 84.3, 56.5, 24.9.

^{19}F NMR (377 MHz, $CDCl_3$) δ 44.54 (s).

IR (thin film, v_{max} /cm^{-1}) 2984, 1588, 1560, 1492, 1412, 1274, 1198, 1146, 1068, 1021,968, 858.

m.p. 150 °C (decomp.).

((6-Methoxypyridin-3-yl)methylene)sulfamoyl fluoride (I-20)

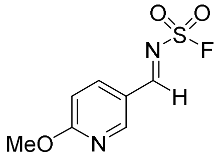

Prepared according to **GP2**, FSI (0.16 ml, 2.00 mmol, 1.00 eq.) was added dropwise to a stirred solution of 5-formyl-2-methoxypyridine (274 mg, 2.00 mmol, 1.00 eq.) in dry $(CH_2Cl)_2$ (2.0 mL, 1.0 M) under N_2 at rt. (Caution: CO_2 evolution). The reaction was heated to reflux and stirred for a further 16 h. The solvent was removed under reduced pressure to approximately ~50% original volume and triturated with heptane and washed with 9:1 Et_2O in pentane to give the ((6-methoxypyridin-3-yl)methylene)sulfamoyl fluoride (**I-20**) as a white solid (320 mg, 73%).

^{1}H NMR (400 MHz, $CDCl_3$) δ 9.04 (s, 1H), 8.68 (d, *J* = 2.4 Hz, 1H), 8.26 (dd, *J* = 8.8, 2.4 Hz, 1H), 6.95 – 6.85 (m, 1H), 4.08 (s, 3H).

^{13}C NMR (101 MHz, $CDCl_3$) δ 174.5 (d, $^3J_{C\text{-}F}$ = 3.6 Hz), 169.6, 156.3, 138.8, 121.6, 113.4, 55.0.

^{19}F NMR (377 MHz, $CDCl_3$) δ 44.19 (s).

HRMS (ESI) *m/z* $C_7H_8FN_2O_3S^+$ requires 219.0244 ($[M+H]^+$) found 219.0244.

IR (thin film, v_{max} /cm^{-1}) 2926, 1779, 1607, 1553, 1504, 1404, 1203, 848, 773.

m.p. 108 – 113 °C.

Aldehydes

4-(Trimethylsilyl)benzaldehyde (A-21)

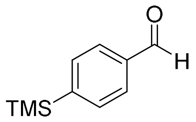

Prepared according to a modified procedure.[40] Under N_2, *n*-butyllithium (2.35 M, 1.87 ml, 4.4 mmol, 1.1 eq.) was added dropwise to a stirred solution of (4-bromophenyl)trimethylsilane (764 µl, 4.0 mmol, 1.0 eq.) in THF (25 ml, 0.16 M) at -78 °C *via* syringe. The reaction mixture was stirred for 15 minutes at -78 °C. DMF was added dropwise at -78 °C following addition, the dry ice/acetone bath was removed and the reaction was warmed to rt and stirred for a further 16 h. Reaction was quenched with aq. sat. NH_4Cl and extracted twice with Et_2O. The combined organic extracts were dried over anhydrous $MgSO_4$, filtered and concentrated under reduced pressure. Purification by flash column chromatography (0 – 3% Et_2O in pentane) afforded 4-(trimethylsilyl)benzaldehyde (**A-21**) as a colourless oil (558 mg, 78%).

R_f (2% Et_2O in pentane) = 0.08.

^{1}H NMR (400 MHz, $CDCl_3$) δ 10.02 (s, 1H), 7.88 – 7.80 (m, 2H), 7.72 – 7.65 (m, 2H), 0.31 (s, 9H).

^{13}C NMR (101 MHz, $CDCl_3$) δ 192.7, 149.2, 136.5, 133.9, 128.7, -1.3.

HRMS (ESI) *m/z* $C_{10}H_{15}OSi$ requires 179.0887 ($[M+H]^+$), found 179.0892.

Data consistent with literature.[41]

4-(1,3-Dioxoisoindolin-2-yl)benzaldehyde (A-22)

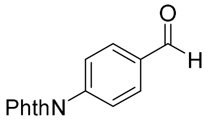

Prepared according to a modified procedure.[42] A pressure tube was charged with 4-bromobenzaldehyde (740 mg, 4.0 mmol, 1.0 eq.) and potassium phthalimide (741 mg, 4.0 mmol, 1.0 eq.). The vessel was then capped with a rubber septum and evacuated and backfilled with N_2 three times. Dry, degassed DMA (40 ml, 0.1 mmol) was added *via* syringe, the rubber septum was removed and CuI (762 mg, 4.0 mmol, 1.0 eq.) was quickly added followed and the vial was sealed with a screw cap. The reaction was heated to 170 °C for 24h and then reaction was then cooled to rt and crude reaction mixture was diluted with EtOAc, transferred to a separating funnel and washed three times with 10% LiCl solution, followed by one wash with brine and then dried over anhydrous $MgSO_4$. The reaction mixture was filtered and concentrated under reduced pressure. Purification by flash column chromatography (0 – 5% EtOAc in CH_2Cl_2) afforded 4-(1,3-dioxoisoindolin-2-yl)benzaldehyde (**A-22**) as a yellow solid (517 mg, 51%).

R_f (2% EtOAc in CH_2Cl_2) = 0.41.

^{1}H NMR (400 MHz, $CDCl_3$) δ 10.06 (s, 1H), 8.06 – 7.98 (m, 2H), 7.98 (dd, *J* = 5.5, 3.1 Hz, 2H), 7.83 (dd, *J* = 5.5, 3.0 Hz, 2H), 7.73 – 7.69 (m, 2H).

^{13}C NMR (101 MHz, $CDCl_3$) δ 191.4, 166.8, 137.2, 135.2, 134.9, 131.6, 130.5, 126.6, 124.2.

HRMS (ESI) *m/z* $C_{15}H_9NO_3$ requires 252.0655 ($[M+H]^+$), found 252.0658.

Data consistent with literature.[42]

4-Formylphenyl pivalate (A-23)

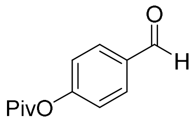

Prepared according to a modified procedure.[43] Triethylamine (1.04 ml, 7.5 mmol, 1.5 eq.) and pivaloyl chloride (923 µl, 7.5 mmol, 1.5 eq.) were added sequentially *via* syringe to a stirred solution of 4-hydroxybenzaldehyde (611 mg, 5.00 mmol, 1.00 eq.) in THF (5.0 ml, 1.0 M) at 0 °C. Subsequently the reaction was warmed gradually to rt and stirred for a further 16 h. The reaction mixture was quenched with aq. sat. NH_4Cl, diluted with Et_2O and the layers were partitioned. The aqueous layer was extracted three times and with Et_2O, the combined organic layers were washed with brine, dried over anhydrous $MgSO_4$ and concentrated under reduced pressure. Purification by flash column chromatography (0 – 10% EtOAc in pet. ether) afforded 4-formylphenyl pivalate (**A-23**) as a colourless oil (302 mg, 55%).

R_f (10% Et_2O in pentane) = 0.40.

^{1}H NMR (400 MHz, $CDCl_3$) δ 9.96 (s, 1H), 7.93 – 7.85 (m, 2H), 7.26 – 7.18 (m, 2H), 1.35 (s, 9H).

^{13}C NMR (101 MHz, $CDCl_3$) δ 191.0, 176.4, 156.0, 133.9, 131.2, 122.4, 39.3, 27.1.

HRMS (ESI) *m/z* $C_{12}H_{15}O_3$ requires 207.1016 ($[M+H]^+$), found 207.1024.

Data consistent with literature.[43]

2-Methoxy-5-(4,4,5,5-tetraethyl-1,3,2-dioxaborolan-2-yl)benzaldehyde (A-24)

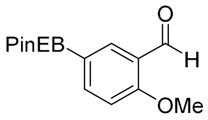

Prepared according to a modified procedure.[44] A round-bottom flask equipped with stirrer bar and reflux condenser was charged with 3-formyl-4-methoxyphenyl boronic acid (500 mg, 2.78 mmol, 1.00 eq.), ethyl pinacol (485 mg, 2.78 mmol, 1.00 eq.) and 3 Å molecular sieves (1.25 g). The reaction vessel was evacuated and backfilled with N_2 and anhydrous CH_2Cl_2 (25 ml, 0.10 M) was added *via* syringe. The reaction mixture was heated at 40 °C for 16 h. The reaction mixture was filtered through celite and the filtrate was concentrated under reduced pressure. The crude mixture was purified by flash column chromatography (0 – 5% EtOAc in pentane), to give 2-methoxy-5-(4,4,5,5-tetraethyl-1,3,2-dioxaborolan-2-yl)benzaldehyde (**A-24**) as a white solid (817 mg, 92%).

R_f (5% EtOAc in pentane) = 0.48.

^{1}H NMR (400 MHz, $CDCl_3$) δ10.45 (s, 1H), 8.28 (d, *J* = 1.7 Hz, 1H), 7.99 (dd, *J* = 8.4, 1.8 Hz, 1H), 6.97 (d, *J* = 8.4 Hz, 1H), 3.94 (s, 3H), 1.84 – 1.64 (m, 8H), 0.95 (t, *J* = 7.5 Hz, 12H).

^{13}C NMR (101 MHz, $CDCl_3$) δ 190.1, 164.0, 142.7, 136.2, 124.5, 111.0, 89.1, 55.8, 26.6, 9.0.

HRMS (ESI) *m/z* $C_{18}H_{28}BO_4$ requires 319.2075 ($[M+H]^+$), found 319.2089.

IR (thin film, v_{max} /cm^{-1}) 2980, 1683, 1605, 1457, 1365, 1350, 1273, 1183, 1132, 1022, 906, 839, 669.

m.p. 50 – 52 °C.

4-Methoxy-3-(4,4,5,5-tetramethyl-1,3,2-dioxaborolan-2-yl)benzaldehyde (A-25)

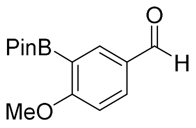

Prepared according to a modified procedure.[45] A round-bottom flask equipped with stirrer bar and reflux condenser was charged with 3-bromo-4-methoxybenzaldehyde (1.29 mg, 6.00 mmol, 1.0 eq.), B_2Pin_2 (2.03 g, 8.00 mmol, 1.30 eq.), KOAc (1.18 g, 12.0 mmol, 2.00 eq.) and [1,1'- $PdCl_2dppf$] 147mg, 0.18 mmol, 3.0 mol%). The reaction vessel was evacuated and backfilled three times with N_2 and then dioxane (20 ml, 0.30 M) was added *via* syringe. Reaction was heated at reflux for 16 h and then cooled to rt and filtered through a short pad of silica gel. The filtrate was concentrated under reduced pressure and purified by flash column chromatography (0 – 30% Et_2O in pentane) to give 4-methoxy-3-(4,4,5,5-tetramethyl-1,3,2-dioxaborolan-2-yl)benzaldehyde (**A-25**) as a white solid (1.38 g, 88%).

R_f (25% Et_2O in pentane) = 0.20.

^{1}H NMR (400 MHz, $CDCl_3$) δ 9.87 (s, 1H), 8.18 (d, *J* = 2.3 Hz, 1H), 7.93 (dd, *J* = 8.7, 2.3 Hz, 1H), 6.94 (d, *J* = 8.6 Hz, 1H), 3.89 (s, 3H), 1.34 (s, 12H).

^{13}C NMR (101 MHz, $CDCl_3$) δ 191.1, 168.9, 140.0, 134.2, 129.4, 110.6, 84.0, 56.1, 24.9.

HRMS (ESI) *m/z* $C_{14}H_{20}BO_4$ requires 263.1449 ($[M+H]^+$), found 263.1457.

Data consistent with literature.[45]

5-bromo-6-methoxynicotinaldehyde (A-26)

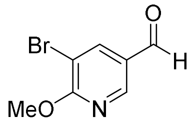

Under an atmosphere of air, a solution of Br_2 in AcOH (3.1 M, 10 ml, 31 mmol, 1.4 eq.) was added dropwise over a period of 30 mins *via* addition funnel to a stirred solution of NaOAc (3.5 g, 43 mmol, 1.9 eq.) and 6-methoxynicotinaldehyde (3.0 g, 22 mmol, 1.0 eq.) in AcOH (10 ml, 2.2 M). The mixture was then heated to 90 °C for 5 h, after which the reaction was cooled to rt, poured over ice water and neutralised to ~pH 7 with 1N aq. NaOH. The mixture was extracted with EtOAc three times and the combined organic layer was washed with brine and dried over anhydrous $MgSO_4$, filtered and concentrated under reduced pressure. Purification by flash column chromatography (0 – 50% CH_2Cl_2 in pentane) afforded 5-bromo-6-methoxynicotinaldehyde (**A-26**) as a white solid (1.77 g, 39%).

R_f (5% Et_2O in pentane) = 0.32.

^{1}H NMR (400 MHz, $CDCl_3$) δ 9.85 (s, 1H), 8.49 (d, *J* = 2.0 Hz, 1H), 8.22 (d, *J* = 2.1 Hz, 1H), 4.04 (s, 3H).

^{13}C NMR (101 MHz, $CDCl_3$) δ 188.3, 163.6, 150.6, 140.3, 127.9, 108.6, 55.7.

HRMS (ESI) *m/z* $C_7H_7{}^{79}BrNO_2$ and $C_7H_7{}^{81}BrNO_2$ requires 215.9655 and 217.9634 respectively ($[M+H]^+$), found 215.9653 and 217.9633.

Data consistent with literature.[46]

6-Methoxy-5-(4,4,5,5-tetramethyl-1,3,2-dioxaborolan-2-yl)nicotinaldehyde (A-27)

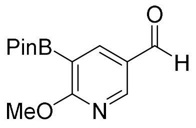

A 25 ml round-bottom flask equipped with reflux condenser and stirrer bar was sequentially charged with 5-bromo-6-methoxynicotinaldehyde (**A-26**) (648 mg, 3.0 mmol, 1.0 eq.), B_2Pin_2 (990 mg, 4.0 mmol, 1.3 eq.), KOAc (589 mg, 6.0 mmol, 2.0 eq.) and $PdCl_2dppf{\cdot}CH_2Cl_2$ (73.5 mg, 0.03 eq., 0.09 mmol). The apparatus was placed under an atmosphere of N_2 and dioxane (10 ml, 0.3 M) was added *via* syringe. The reaction was heated to reflux for 16 h. The reaction was cooled to rt, filtered through a pad of celite® and washed with copious EtOAc. Purification by flash column chromatography (0 – 30% EtOAc in pentane) afforded 6-methoxy-5-(4,4,5,5-tetramethyl-1,3,2-dioxaborolan-2-yl)nicotinaldehyde (**A-27**) as a white solid (544 mg, 69%).

R_f (20% EtOAc in pentane) = 0.15.

^{1}H NMR (400 MHz, $CDCl_3$) δ 9.96 (s, 1H), 8.70 (d, *J* = 2.5 Hz, 1H), 8.46 (d, *J* = 2.5 Hz, 1H), 4.06 (s, 3H), 1.36 (s, 12H).

^{13}C NMR (101 MHz, $CDCl_3$) δ 189.7, 170.6, 154.4, 146.5, 126.2, 84.4, 54.9, 24.9.

HRMS (ESI) *m/z* $C_{13}H_{19}BNO_4$ requires 264.1402 ($[M+H]^+$), found 264.1402.

IR (thin film, ν_{max} /cm^{-1}) 2981 ,1695, 1593, 1474, 1407, 1346, 1199, 1144, 1073, 854, 792, 676.

m.p. 104 – 108 °C.

2,4-Dimethoxypyrimidine-5-carbaldehyde (A-28)

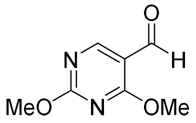

Prepared according to a modified procedure.[40] Under N_2, *n*-butyllithium (2.12 M, 2.10 ml, 4.4 mmol, 1.1 eq.) was added dropwise to a stirred solution of 5-bromo-2,4-dimethoxypyrimidine (876 mg, 4.0 mmol, 1.0 eq.) in Et_2O (13 ml, 0.30 M) at -78 °C. The reaction mixture was stirred for 15 minutes at 78 °C. DMF (0.93 ml, 12.0 mmol, 3.0 eq.) was added dropwise at -78 °C and stirred for 1h at that temperature. The dry ice/acetone bath was then removed and the reaction was warmed to rt and stirred for a further 1 h. The reaction was quenched with aq. sat. NH_4Cl and extracted thrice with Et_2O. The combined organic extracts were washed with brine, dried over anhydrous $MgSO_4$, filtered and concentrated under reduced pressure. Purification by flash column chromatography (0 – 3% Et_2O in pentane) afforded 2,4-dimethoxypyrimidine-5-carbaldehyde (**A-28**) as a white solid (373 mg, 55%).

R_f (20% Et_2O in pet ether) = 0.21.

^{1}H NMR (400 MHz, $CDCl_3$) δ 10.16 (s, 1H), 8.77 (s, 1H), 4.11 (s, 3H), 4.07 (s, 3H).

^{13}C NMR (101 MHz, $CDCl_3$) δ 186.6, 171.3, 167.7, 161.6, 112.4, 55.8, 54.7.

HRMS (ESI) *m/z* $C_7H_9N_2O_3$ requires 169.0608 ($[M+H]^+$), found 169.0613.

Data consistent with literature.[47]

Alkenes

3-Methylbut-3-en-1-yl 4-methylbenzenesulfonate (O-29)

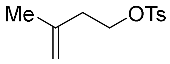

3-Methyl-3-buten-1-ol (1.52 ml, 15.0 mmol, 1.00 eq.) was added dropwise to a solution of DMAP (2.75 g, 22.5 mmol, 1.50 eq.) and TsCl (3.58 g, 18.8 mmol, 1.25 eq.) in CH_2Cl_2 (75 ml, 0.20 M) under N_2, at 0 °C. The reaction was warmed slowly to rt and stirred for a further 16 h. The reaction was cooled to 0 °C, quenched with water and stirred for a further 10 minutes. The phases were then partitioned and the aqueous layer was washed with CH_2Cl_2. The combined organic layers were washed with brine, dried over anhydrous $MgSO_4$ and concentrated under reduced pressure. Purification by flash column chromatography (4 – 5% EtOAc in pet. ether) afforded 3-methylbut-3-en-1-yl 4-methylbenzenesulfonate (**O-29**) as a colourless oil (3.28 g, 91%).

R_f (14% EtOAc in pet ether) = 0.54.

^{1}H NMR (400 MHz, $CDCl_3$) δ 7.82 – 7.73 (m, 2H), 7.38 – 7.30 (m, 2H), 4.78 (dq, *J* = 2.3, 1.1 Hz, 1H), 4.67 (dq, *J* = 2.2, 1.2 Hz, 1H,), 4.12 (t, *J* = 6.9 Hz, 2H), 2.44 (s, 3H), 2.34 (t, *J* = 6.6 Hz, 2H), 1.65 (t, *J* = 1.1 Hz, 3H).

^{13}C NMR (101 MHz, $CDCl_3$) δ 144.8, 140.2, 133.3, 129.9, 128.0, 113.2, 68.6, 36.9, 22.4, 21.7.

HRMS (ESI) *m/z* $C_{12}H_{16}O_3SNa$ requires 263.0712 ($[M+Na]^+$), found 263.0724.

Data consistent with literature.[48]

5-((3-methylbut-3-en-1-yl)thio)-1-phenyl-1H-tetrazole (O-30)

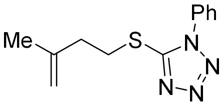

Prepared according to a modified procedure.[49] 3-Methylbut-3-en-1-yl 4-methylbenzenesulfonate (**O-29**) (1.49 g, 6.20 mmol, 1.0 eq.) was added to a solution of 1-phenyl-1H-tetrazole-5-thiol (1.22 g, 6.82 mmol, 1.1 eq.) and K_2CO_3 (4.28 g, 31.0 mmol, 5.0 eq.) in MeCN (30 ml, 0.21 M) under N_2. The reaction was heated at 50 °C for 16 h. The reaction was cooled to rt and partitioned between water and EtOAc. The aqueous layer was extracted with EtOAc thrice, the combined organic layers were washed with aq. sat. $NaHCO_3$, dried over anhydrous $MgSO_4$, filtered and concentrated under reduced pressure. Purification by flash column chromatography (4 – 5% EtOAc in pet. ether) afforded 5-((3-methylbut-3-en-1-yl)thio)-1-phenyl-1H-tetrazole (**O-30**) as a colourless oil (1.13 g, 74%).

R_f (10% Et_2O in pentane) = 0.31.

^{1}H NMR (400 MHz, $CDCl_3$) δ 7.60 – 7.46 (m, 5H), 4.81 (s, 1H), 4.75 (s, 1H), 3.50 (t, *J* = 7.4 Hz, 2H), 2.51 (t, *J* = 7.4 Hz, 2H), 1.75 (s, 3H).

^{13}C NMR (101 MHz, $CDCl_3$) δ 154.4, 142.5, 133.7, 130.1, 129.8, 123.9, 112.5, 36.9, 31.5, 22.1.

HRMS (ESI) *m/z* $C_{12}H_{15}N_4S$ requires 247.1012 ($[M+H]^+$), found 247.1009.

IR (thin film, ν_{max} /cm^{-1}) 3075, 1650, 1597, 1500, 1412, 1388, 1413, 1388, 1243, 1090, 1015, 896, 761, 694.

Data consistent with literature.[50]

5-((3-Methylbut-3-en-1-yl)sulfonyl)-1-phenyl-1H-tetrazole (O-31)

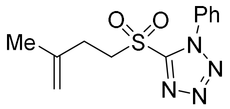

Prepared according to a modified procedure.[51] A solution of oxidant made from $Mo_7O_{24}(NH_4)_6{\cdot}4H_2O$ (742 mg, 0.60 mmol, 0.16 eq.) in H_2O_2 (30% in H_2O, 4 ml) was added to a solution of sulfide (**O-30**) (900 mg, 3.65 mmol, 1.0 eq.) at 0 °C in EtOH (40 ml 0.091 M). The reaction was slowly warmed to rt and stirred for 3 days. The reaction was diluted with EtOAc and the organic phase was sequentially washed with water, brine and then dried over $MgSO_4$, filtered and concentrated under reduced pressure. Purification by flash column chromatography (4 – 10% EtOAc in pet. ether) afforded 5-((3-methylbut-3-en-1-yl)sulfonyl)-1-phenyl-1H-tetrazole (**O-31**) as a colourless oil (563 mg, 55%).

R_f (10% EtOAc in pet ether) = 0.30.

^{1}H NMR (400 MHz, $CDCl_3$) δ 7.71 – 7.66 (m, 2H), 7.66 – 7.57 (m, 3H), 4.87 (s, 1H), 4.81 (s, 1H), 3.91 – 3.82 (m, 2H), 2.69 – 2.60 (m, 2H), 1.80 (s, 3H).

^{13}C NMR (101 MHz, $CDCl_3$) δ 153.5, 140.5, 133.1, 131.6, 129.9, 125.2, 113.1, 54.7, 29.9, 22.3.

HRMS (ESI) *m/z* $C_{12}H_{15}N_4O_2S$ requires 279.0910 ($[M+H]^+$), found 279.0908.

IR (thin film, v_{max} /cm^{-1}) 3080, 1652, 1596, 1498, 1157, 903, 764, 689, 624.

3-Methylbut-3-en-1-yl but-3-enoate (O-32)

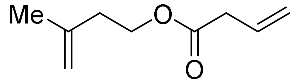

A solution of crotonyl chloride (1.85 ml, 19.5 mmol, 1.1 eq.) in CH_2Cl_2 (2.0 ml, 2.0 M) was added to a solution of 3-methyl-3-buten-1-ol (1.32 ml, 13.0 mmol, 1.0 eq.) and NEt_3 (3.60 ml, 26.0 mmol, 2.0 eq.) in CH_2Cl_2 under nitrogen at 0 °C. The reaction was slowly warmed to rt and then stirred at rt for 1 h and the reaction mixture was poured into cold water and the phases were partitioned. The aqueous phase was extracted with CH_2Cl_2 three times, dried over anhydrous $MgSO_4$, filtered and the solvent was removed under reduced pressure. Purification by flash column chromatography (0 – 40% CH_2Cl_2 in pet ether) afforded 3-methylbut-3-en-1-yl but-3-enoate (**O-32**) as a yellow oil (1.84 g, 92%).

R_f (5% CH_2Cl_2 in pentane) = 0.33.

^{1}H NMR (400 MHz, $CDCl_3$) δ 5.97 – 5.84 (m, 1H), 5.19 – 5.14 (m, 1H), 5.14 – 5.11 (m, 1H), 4.79 (s, 1H), 4.72 (s, 1H), 4.20 (td, *J* = 6.9, 0.8 Hz, 2H), 3.07 (dq, *J* = 7.0, 1.3 Hz, 2H), 2.33 (t, *J* = 6.9 Hz, 2H), 1.74 (s, 3H).

^{13}C NMR (101 MHz, $CDCl_3$) δ 171.6, 141.7, 130.4, 118.5, 112.4, 63.0, 39.2, 36.8, 22.6.

HRMS (ESI) *m/z* $C_9H_{15}O_2$ ($[M+H]^+$) requires 155.1067, found 155.1067.

IR (thin film, v_{max} /cm^{-1}) 2972, 1741, 1650, 1378, 1329, 1253, 1173, 1035, 994, 921, 894.

tetrahydro-1H-spiro[pentalene-2,2'-[1,3]dioxolan]-5(3H)-one (O-33)

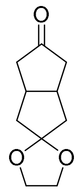

Ethylene glycol (0.89 ml, 15.8 mmol, 0.90 eq.) was added to a solution of tetrahydropentalene-2,5(1H,3H)-dione (2.42 g, 17.5 mmol, 1.0 eq.) and TsOH (329 mg, 1.75 mmol, 0.10 eq.) in benzene (50 ml, 0.35 M). The flask was fitted with a Dean-Stark apparatus and heated at reflux for 6 h. The reaction mixture was cooled to rt, diluted with toluene and washed sequentially with 1N NaOH and brine solutions. The combined organic layers were dried over anhydrous $MgSO_4$, filtered and concentrated under reduced pressure. Purification by flash column chromatography (0 – 30% EtOAc in pet ether) afforded tetrahydro-1H-spiro[pentalene-2,2'-[1,3]dioxolan]-5(3H)-one (**O-33**) as a pale-yellow oil (915 mg, 29%).

R_f (30% EtOAc in pet ether) = 0.17.

^{1}H NMR (400 MHz, $CDCl_3$) δ 3.83 (s, 4H), 2.85 – 2.70 (m, 2H), 2.49 – 2.35 (m, 2H), 2.21 – 2.05 (m, 4H), 1.69 – 1.63 (m, 2H).

^{13}C NMR (101 MHz, $CDCl_3$) δ 220.3, 118.4, 64.7, 64.3, 44.5, 42.6, 37.1.

HRMS (ESI) *m/z* $C_{10}H_{15}O_3$ requires 183.1016 ($[M+H]^+$), found 183.1019.

Data consistent with literature.[52]

5-Methylenehexahydro-1H-spiro[pentalene-2,2'-[1,3]dioxolane] (O-34)

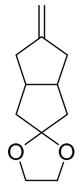

Prepared according to a modified procedure.[53] NaHMDS in THF (2.0 M, 2.64 ml, 5.27 mmol, 1.2 eq.) was added dropwise to a stirred solution of $MePPh_3Br$ (2.35 g, 6.59 mmol, 1.5 eq.) in THF (20 ml, 0.22 M) at 0 °C for 30 minutes. A solution of 5-methylenehexahydro-1H-spiro[pentalene-2,2'-[1,3]dioxolane] (**O-33**) (800 mg, 4.39 mmol, 1.0 eq.) in 5 ml THF was added dropwise at 0 °C. The reaction was stirred for a further 30 minutes at 0 °C after which, the reaction was slowly warmed to rt and stirred for a further 3 h. The reaction was diluted with Et_2O and washed sequentially with water, brine and then dried over anhydrous $MgSO_4$ and then concentrated under reduced pressure. Purification by flash column chromatography (6% Et_2O in pentane) afforded 5-methylenehexahydro-1H-spiro[pentalene-2,2'-[1,3]dioxolane] (**O-34**) as a colourless oil (683 mg, 86%).

R_f (10% Et_2O in pentane) = 0.40.

^{1}H NMR (400 MHz, $CDCl_3$) δ 4.81 (h, J = 1.8 Hz, 2H), 3.92 – 3.83 (m, 4H), 2.64 – 2.53 (m, 2H), 2.53 – 2.42 (m, 2H), 2.11 – 2.03 (m, 2H), 1.99 (ddt, J = 13.4, 6.5, 1.4 Hz, 2H), 1.56 (ddd, J = 13.5, 7.0, 1.5 Hz, 2H).

^{13}C NMR (101 MHz, $CDCl_3$) δ 152.4, 118.7, 106.3, 64.7, 64.0, 42.0, 40.0, 39.8.

HRMS (ESI) *m/z* $C_{11}H_{17}O_2$ requires 181.1223 ($[M+H]^+$), found 181.1232.

Data consistent with literature.[53]

2,2,2-Trifluoro-1-(4-methylenepiperidin-1-yl)ethan-1-one (O-35)

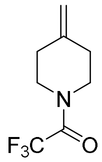

Prepared according to a modified procedure.[54] Trifluoroacetic acid (24 ml) was added to a stirred solution of tert-butyl 4-methylidenepiperidine-1-carboxylate (1.18 g, 6.0 mmol, 1.0 eq.) in CH_2Cl_2 (24 ml). The reaction mixture was stirred at rt for 1 h and then concentrated thoroughly under reduced pressure. The flask was evacuated and backfilled with N_2 three times and the crude residue was dissolved in CH_2Cl_2 (10 ml, 0.60 M) and cooled to 0 °C. The reaction mixture was charged sequentially with NEt_3 (2.50 ml, 18 mmol, 3.0 eq.) and TFAA (0.92 ml, 6.6 mmol, 1.1 eq.). The flask was then warmed to rt and the mixture stirred for a further 16 h. The reaction was quenched with water and the aqueous layer was extracted 3 times with CH_2Cl_2. The combined organic layers were washed with brine, dried over anhydrous $MgSO_4$ and concentrated under reduced pressure. Purification by flash column chromatography (0 – 10% Et_2O in pentane) gave 2,2,2-trifluoro-1-(4-methylenepiperidin-1-yl)ethan-1-one (**O-35**) as a white solid (847 mg, 73%).

R_f (10% Et_2O in pentane) = 0.22.

^{1}H NMR (400 MHz, $CDCl_3$) δ 4.83 (s, 2H), 3.69 – 3.62 (m, 2H), 3.62 – 3.53 (m, 2H), 2.35 – 2.25 (m, 4H).

^{13}C NMR (101 MHz, $CDCl_3$) δ 155.6 (q, $^2J_{C-F}$ = 35.2 Hz), 142.9, 116.7 (q, $^1J_{C-F}$ = 288.0 Hz), 111.0, 47.1 (q, $^3J_{C-F}$ = 3.5 Hz), 45.2, 34.9, 33.9.

^{19}F NMR (377 MHz, $CDCl_3$) δ -68.88 (s).

HRMS (ESI) *m/z* $C_8H_{11}F_3NO$ requires 194.0787 ($[M+H]^+$), found 194.0787.

IR (thin film, v_{max} /cm^{-1}) 3023, 2941, 1754, 1700, 1466, 1299, 1204, 1177, 1145, 988, 902.

tetrahydro-4H-thiopyran-4-one 1,1-dioxide (O-36)

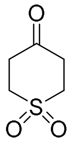

Prepared according to a modified procedure.[55] A mixture of Oxone (31.7 g, 100 mmol, 2.9 eq.) and $NaHCO_3$ (26.9 g, 32 mmol, 0.9 eq.) was added portion wise within 30 min to a solution of tetrahydrothiopyran-4-one (3.95 g, 34 mmol, 1.0 eq.) in MeCN (45 ml, 0.76 M) and aqueous Na_2EDTA solution (30 mL, 4×10^{-4} M). at 0 °C. The reaction was warmed to rt and was stirred for 3 h. The reaction mixture was diluted with CH_2Cl_2, dried over anhydrous $MgSO_4$, and filtered through Celite. The filtrate was concentrated under reduced pressure to afford tetrahydro-4H-thiopyran-4-one 1,1-dioxide (**O-36**) as a white solid (4.16 g, 83% yield).

^{1}H NMR (400 MHz, $CDCl_3$) δ 3.37 – 3.27 (m, 4H), 2.96 – 2.87 (m, 4H).

^{13}C NMR (101 MHz, $CDCl_3$) δ 202.2, 49.7, 38.3.

LRMS (ESI) *m/z* 149.0 ($[M+H]^+$).

Data consistent with literature.[55]

4-Methylenetetrahydro-2H-thiopyran 1,1-dioxide (O-37)

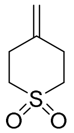

Methyltriphenylphosphonium bromide (5.89 g, 16.5 mmol, 1.1 eq.) was added to a stirred solution of potassium tert-butoxide (2.22 g, 18.0 mmol, 1.2 eq.) in THF (30 ml, 0.5 M) at 0°C under N_2. The reaction was stirred at 0 °C for 30 minutes. A solution of tetrahydro-4H-thiopyran-4-one 1,1-dioxide (**O-36**) (2.22 g, 15.0 mmol, 1.0 eq.) in DMSO (15 ml) was added dropwise. The reaction warmed to rt and stirred for 16 h. The reaction mixture was quenched with aq. sat. NH_4Cl and the phases were partitioned. The aqueous phase was extracted three times with EtOAc and the combined organic layers were washed with brine, dried over $MgSO_4$ and concentrated under reduced pressure. Purification by flash column chromatography (0 – 30% EtOAc in pet ether) afforded 4-methylenetetrahydro-2H-thiopyran 1,1-dioxide (**O-37**) as a white solid (730 mg, 33%).

R_f (40% EtOAc in pet ether) = 0.29.

^{1}H NMR (400 MHz, $CDCl_3$) δ 4.94 (s, 2H), 3.09 – 2.99 (m, 4H), 2.76 – 2.67 (m, 4H).

^{13}C NMR (101 MHz, $CDCl_3$) δ 140.6, 114.9, 52.4, 32.1.

HRMS (ESI) *m/z* $C_6H_{10}O_2SNa$ requires 169.0294 ($[M+Na]^+$), found 169.0293.

Data consistent with literature.[56]

tert-butyl (5-bromopyridin-2-yl)(tert-butoxycarbonyl)carbamate (O-38)

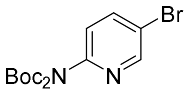

Boc_2O (9.61 g, 44 mmol, 2.2 eq.) was added portion wise to a stirred solution of 5-bromopyridin-2-amine (3.46 g, 20 mmol, 1.0 eq.), DMAP (244 mg, 2.0 mmol, 0.10 eq.) and NEt_3 (5.54 ml, 40 mmol, 2.0 eq.) in CH_2Cl_2 (70 ml, 0.30 M). The reaction was stirred at rt for 16 h after which, the reaction mixture was partitioned over water and the aqueous layer was extracted 3 times with CH_2Cl_2. The combined organic extracts were washed with brine, dried over $MgSO_4$, filtered and the solvent was removed under reduced pressure. Purification by flash column chromatography (5 – 8% EtOAc in pentane) afforded tert-butyl (5-bromopyridin-2-yl)(tert-butoxycarbonyl)carbamate (**O-38**) as a white solid (4.67 g, 63%).

R_f (5% EtOAc in pentane) = 0.23.

^{1}H NMR (400 MHz, $CDCl_3$) δ 8.51 (dd, *J* = 2.5, 0.7 Hz, 1H), 7.83 (dd, *J* = 8.5, 2.5 Hz, 1H), 7.17 (dd, *J* = 8.4, 0.7 Hz, 1H), 1.44 (s, 18H).

^{13}C NMR (101 MHz, $CDCl_3$) δ 151.1, 151.0, 149.8, 140.6, 123.0, 118.7, 83.6, 28.0.

HRMS (ESI) *m/z* $C_{15}H_{22}{}^{79}BrN_2O_4$ and $C_{15}H_{22}{}^{81}BrN_2O_4$ ($[M+H]^+$) requires 373.0757 and 375.0737, found 373.0765 and 375.0728.

Data consistent with literature.[57]

Tert-butyl (*tert*-butoxycarbonyl)(5-vinylpyridin-2-yl)carbamate (O-39)

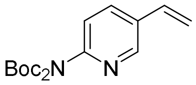

NEt_3 (1.04 ml, 7.5 mmol, 1.5 eq.) was added to a suspension of potassium vinyltrifluoroborate (1.00 g, 7.5 mmol, 1.5 eq.), *tert*-butyl (5-bromopyridin-2-yl)(*tert*-butoxycarbonyl)carbamate (**O-38**) (1.87 g, 5.0 mmol, 1.0 eq.) and $PdCl_2dppf{\cdot}CH_2Cl_2$ (204 mg, 0.25 mmol, 5 mol%) in EtOH (75 ml, 0.10 M). The reaction mixture was then sparged with N_2 for 10 minutes and then heated at reflux for 1.5 h. Subsequently the reaction was cooled to rt, diluted with EtOAc and partitioned over water. The organic layer was dried over $MgSO_4$, filtered and concentrated under reduced pressure. Purification by flash column chromatography (5 – 8% EtOAc in pentane) afforded *tert*-butyl (*tert*-butoxycarbonyl)(5-vinylpyridin-2-yl)carbamate (**O-39**) as a white solid (1.34 g, 84%).

R_f (5% EtOAc in pentane) = 0.17.

^{1}H NMR (400 MHz, $CDCl_3$) δ 8.46 (d, *J* = 2.4 Hz, 1H), 7.77 (dd, *J* = 8.3, 2.5 Hz, 1H), 7.19 (d, *J* = 8.3 Hz, 1H), 6.70 (dd, *J* = 17.7, 11.0 Hz, 1H), 5.81 (dd, *J* = 17.6, 0.6 Hz, 1H), 5.38 (dd, *J* = 11.0, 0.6 Hz, 1H), 1.44 (s, 18H).

^{13}C NMR (101 MHz, $CDCl_3$) δ 151.6, 151.4, 147.2, 134.7, 132.9, 131.9, 121.6, 116.6, 83.2, 28.0.

HRMS (ESI) *m/z* $C_{17}H_{25}N_2O_4$ requires 321.1809 ($[M+H]^+$), found 321.1810.

IR (thin film, ν_{max} /cm^{-1}) 2981, 2929, 1779, 1718, 1614, 1517, 1468, 1421, 1388, 1310, 1255, 1212, 1178, 1119, 1032, 848, 720, 652.

3,5-Dimethyl-1-vinyl-1H-pyrazole (O-40)

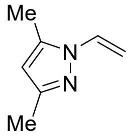

Prepared according to a modified procedure.[1] Under air, DBU (0.91 ml, 6.0 mmol, 2.0 eq.) was added to a solution of 3,5-dimethyl-1*H*-pyrazole (288 mg, 3.0 mmol, 1.0 eq.) and vinyl-thianthrenium BF_4 (1.68 g, 5.1 mmol, 1.7 eq.) in CH_2Cl_2 (30 ml, 0.10 M). The reaction was stirred for 3 h at rt and then the solvent was removed under reduced pressure. Purification by flash column chromatography (0 – 10% Et_2O in pentane) afforded 3,5-dimethyl-1-vinyl-1H-pyrazole (**O-40**) as a colourless oil (320 mg, 87%).

R_f (10% Et_2O in pentane) = 0.24.

^{1}H NMR (400 MHz, $CDCl_3$) δ 6.86 (dd, *J* = 15.3, 8.9 Hz, 1H), 5.85 (s, 1H), 5.58 (d, *J* = 15.4 Hz, 1H), 4.74 (d, *J* = 8.9 Hz, 1H), 2.25 (d, *J* = 0.8 Hz, 4H), 2.24 (s, 3H).

^{13}C NMR (101 MHz, $CDCl_3$) δ 150.0, 139.2, 129.3, 106.9, 99.4, 13.8, 11.0.

HRMS (ESI) *m/z* $C_7H_{11}N_2$ requires 123.0917 ($[M+H]^+$), found 123.0916.

Data consistent with literature.[58]

tert-butyl vinylcarbamate (O-41)

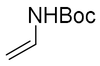

Boc_2O was added portionwise to a solution of N-vinyl formamide (0.71 ml, 10 mmol, 1.0 eq.), NEt_3 (1.94 ml, 14 mmol, 1.4 eq.) and DMAP (122 mg, 1.0 mmol, 0.10 eq.) in THF (10 ml ,1.0 M) under N_2 at 0 °C. The reaction was then warmed to rt and stirred for 2 h. LiOH (0.84 g, 35 mmol, 3.5 eq.) was then added and the reaction was then heated at 50 °C for 4 h, subsequently the reaction was cooled to rt and diluted with water and Et_2O. The phases were partitioned and the aqueous phase was washed with Et_2O. The combined organic phases were dried over anhydrous $MgSO_4$, filtered and concentrated

under reduced pressure. Purification by flash column chromatography (10% EtOAc in pentane) afforded *tert*-butyl vinylcarbamate (**O-41**) as a white solid (1.02 g, 71%).

^{1}H NMR (400 MHz, CDCl$_3$) δ 6.73 – 6.58 (m, 1H), 6.30 (s, 1H), 4.40 (d, *J* = 15.7 Hz, 1H), 4.21 (d, *J* = 8.8 Hz, 1H), 1.46 (s, 9H).

Data consistent with literature.[59]

Di-tert-butyl vinyliminodicarbonate (O-42)

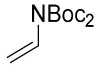

Boc$_2$O (1.93 g, 8.82 mmol, 3.0 eq.) and DMAP (35 mg, 0.29 mmol, 0.10 eq.) were added sequentially to a stirred solution of tert-butyl vinylcarbamate (**O-41**) (421 mg, 2.94 mmol, 1.0 eq.) in MeCN (7.0 ml, 0.40 M). The reaction mixture was stirred at rt for 2 h and subsequently concentrated under reduced pressure. Purification by flash column chromatography (0 – 5% Et$_2$O in pentane) afforded di-tert-butyl vinyliminodicarbonate (**O-42**) as a white solid (714 mg, 99%).

R*f* (5% Et$_2$O in pentane) = 0.17.

^{1}H NMR (400 MHz, CDCl$_3$) δ 6.68 – 6.57 (m, 1H), 4.82 (dd, *J* = 16.0, 0.9 Hz, 1H), 4.60 (dq, *J* = 9.1, 0.9 Hz, 2H), 1.51 (s, 18H).

^{13}C NMR (101 MHz, CDCl$_3$) δ 151.4, 130.3, 99.3, 83.3, 28.0.

HRMS (ESI) *m/z* $C_{12}H_{21}NO_4Na$ requires 266.1363 ([M+Na]$^+$), found 266.1374.

Data consistent with literature.[59]

Characterisation of N-Triflyl system

Procedure for aza-Paternò-Büchi reaction of 2a

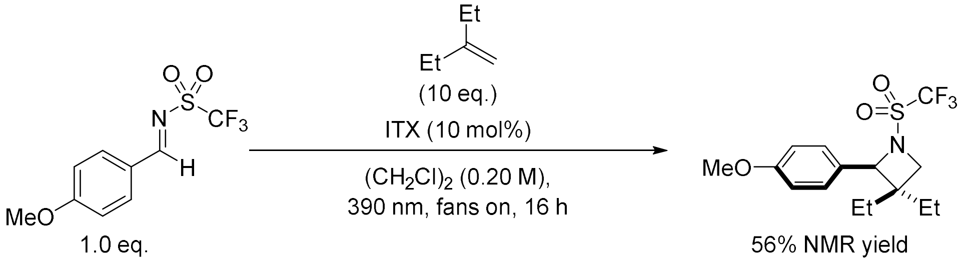

1,1,1-trifluoro-N-(4-methoxybenzylidene)methanesulfonamide (**I-43**) (53.4 mg,, 0.20 mmol, 1.0 eq.), ITX (5.1 mg, 0.020 mmol, 10 mol%) were added to an oven-dried tapered microwave vial equipped with a stirrer bar and a septum. The vial was evacuated and backfilled with nitrogen 3 times, followed by addition of anhydrous (CH$_2$Cl)$_2$ (1.0, 0.20 M) and 3-methylenepentane (240 µl, 2.0 mmol, 10 eq.). The reaction was then stirred under purple light irradiation (390 nm) at ambient temperature for 16 h. The reaction mixture was diluted with CH$_2$Cl$_2$, and the solvent removed under reduced pressure. ^{19}F NMR analysis of the crude reaction mixture a total yield of 56%.

In this system the photocatalyst and product are inseparable, in order to get an analytical sample, the reaction was run with the following procedure:

1,1,1-trifluoro-N-(4-methoxybenzylidene)methanesulfonamide (**I-43**) (53.4 mg, 0.20 mmol, 1.0 eq.) and [Ir(dFCF$_3$ppy)$_2$dtbbpy]PF$_6$ (2.2 mg, 0.002 mmol, 1 mol%) were added to an oven-dried tapered microwave vial equipped with a stirrer bar and a septum. The vial was evacuated and backfilled with

nitrogen 3 times, followed by addition of anhydrous $(CH_2Cl)_2$ (1.0 ml, 0.20 M) and 3-methylenepentane (240 µl, 2.0 mmol, 10 eq.). The reaction was then stirred under blue light irradiation (427 nm) at ambient temperature for 16 h. The reaction mixture was diluted with CH_2Cl_2, and the solvent removed under reduced pressure. Purification by flash column chromatography (0 – 2% Et_2O in pentane) afforded 3,3-diethyl-2-(4-methoxyphenyl)-1-((trifluoromethyl)sulfonyl)azetidine (**2a**) (47.1 mg, 66%).

3,3-diethyl-2-(4-methoxyphenyl)-1-((trifluoromethyl)sulfonyl)azetidine (2a)

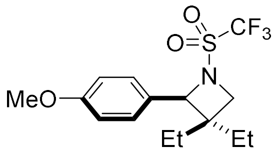

R_f(3% Et_2O in pentane) = 0.45.

^{1}H NMR (400 MHz, $CDCl_3$) δ 7.26 (d, *J* = 8.5 Hz, 2H), 6.94 – 6.86 (m, 2H), 5.25 (s, 1H), 3.93 (d, *J* = 7.9 Hz, 1H), 3.81 (s, 3H), 3.74 (d, *J* = 7.9 Hz, 1H), 1.87 – 1.71 (m, 2H), 1.33 (dq, *J* = 14.9, 7.3 Hz, 1H), 1.12 (dq, *J* = 14.6, 7.4 Hz, 1H), 0.93 (t, *J* = 7.4 Hz, 3H), 0.56 (t, *J* = 7.4 Hz, 3H).

^{13}C NMR (101 MHz, $CDCl_3$) δ 159.7, 128.4, 127.8, 120.0 (q, J = 322.3 Hz), 113.8, 75.5, 60.3, 55.4, 43.6, 29.2, 24.2, 8.0, 7.3

^{19}F NMR (377 MHz, $CDCl_3$) δ -74.66(s).

HRMS (ESI) *m/z* $C_{15}H_{20}F_3NO_3SK^+$ requires 390.0748 ($[M+K]^+$), found 390.0762.

IR (thin film, ν_{max} /cm^{-1}) 2971, 1614, 1515, 1462, 1305, 1254, 1227, 1206, 1035, 837, 630.

Preparation of imine I-43

1,1,1-trifluoro-N-(4-methoxybenzylidene)methanesulfonamide (I-43)

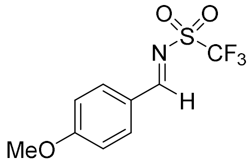

Prepared according to a modified procedure.[60] Under air, a pressure tube was charged sequentially with trifluoromethanesulfonamide (358 mg, 2.40 mmol, 1.25 eq.), 3Å molecular sieves (2.0 g), CH_2Cl_2 (7.5 ml, 0.3 M), p-anisialdehyde (0.24 ml, 1.90 mmol, 1.00 eq.) and pyrrolidine (18 µl, 0.19 mmol, 10 mol%). The tube was sealed and heated to 60 °C for 16 h. After the elapsed time, the reaction was cooled to room temperature, filtered through a short pad of Celite® and concentrated under reduced pressure to give 1,1,1-trifluoro-N-(4-methoxybenzylidene)methanesulfonamide (**I-43**), which was recrystalised from Et_2O, as a white solid (211 mg, 42%).

^{1}H NMR (400 MHz, $CDCl_3$) δ 9.03 (s, 1H), 8.03 (d, *J* = 8.5 Hz, 2H), 7.10 – 7.03 (m, 2H), 3.96 (s, 3H).

^{13}C NMR (101 MHz, $CDCl_3$) δ 178.4, 167.7, 135.8, 124.65, 119.3 (q, *J* = 321.5 Hz), 115.5, 56.2.

^{19}F NMR (377 MHz, $CDCl_3$) δ -76.97 (s).

IR (thin film, ν_{max} /cm^{-1}) 1602, 1585, 1546, 1512, 1430, 1356, 1266, 1205, 1168, 1119, 1020, 828, 758, 623.

Data consistent with literature.[61]

Crystallographic Data

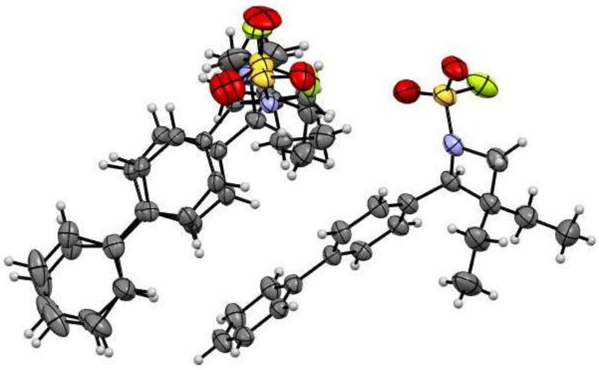

Supplementary Fig. 31 XRD structure of 3j (CCDC number: 2375437).

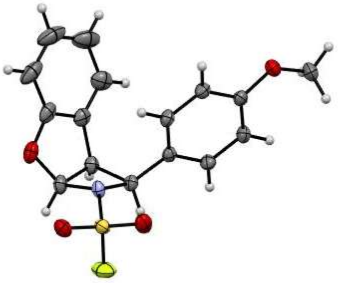

Supplementary Fig. 32 XRD structure of 4aa (CCDC number: 2375438).

Supplementary Table 24 Combined crystallographic data for structures **3j** and **4aa**.

	**3j**	**4aa**
Chemical Formula	C19 H22 F N O2 S	C16 H14 F N O4 S
Molecular Weight	347.45	335.36
Space Group System	triclinic	monoclinic
Space Group (H-M)	P -1	P 21/c
Space Group (Hall)	-P 1	-P 2ybc
a	9.7064(2)	12.3026(4)
b	10.3842(2)	5.4910(2)
c	18.0415(4)	22.6138(7)
α	80.6392(17)	90
β	87.9490(17)	92.289(3)
γ	89.1239(17)	90
V	1793.03(7)	1526.42(9)
Z	4	4
T(K)	150	150
Unique Reflections	16843	4405
Collected Reflections	38316	9656
Mu	1.778	2.176
Radiation Type	Cu K\a	Cu K\a
λ	1.54184	1.5418
Parameters	548	208
R_{all}	0.0657	0.0438
R_{gt}	0.0584	0.0349
wR	0.1629	0.0939
R_{int}	0.035	0.031
goof	0.9957	0.9963
CCDC deposition number	2375437	2375438

Unsuccessful Substrates

Imines

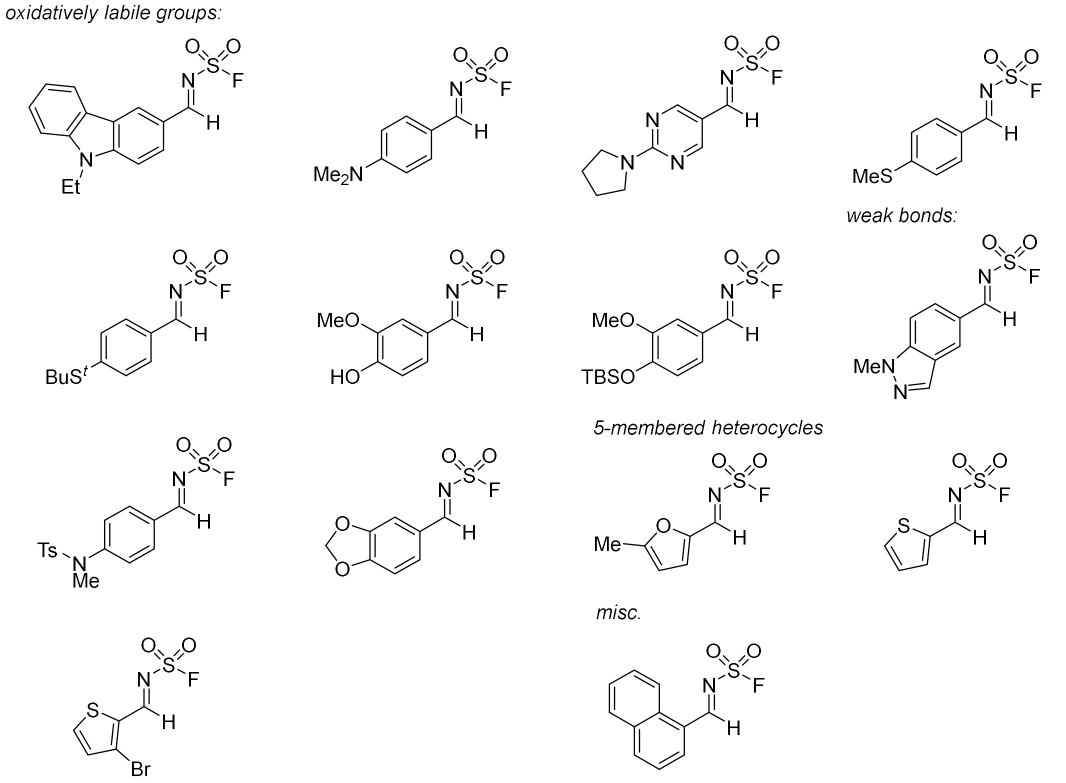

Supplementary Fig. 33 Unsuccessful imine substrates.

Alkenes

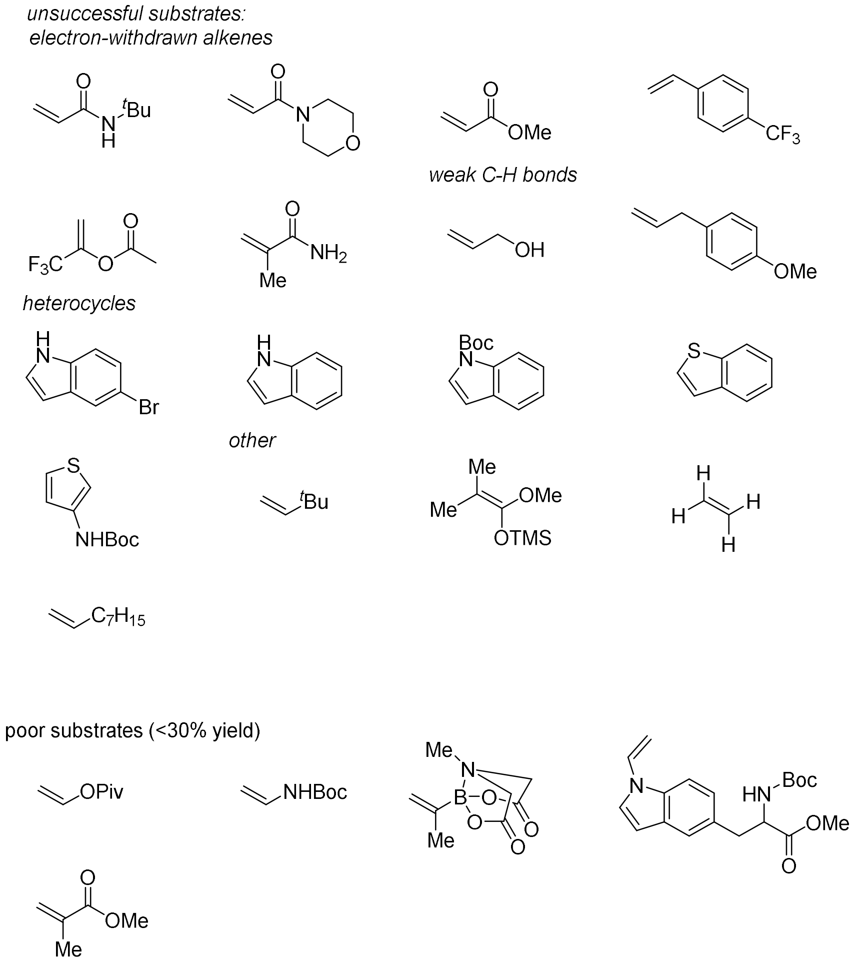

Supplementary Fig. 34 Unsuccessful alkene substrates.

Spectroscopic Data

Azetidines

3,3-diethyl-2-(4-methoxyphenyl)-1-((trifluoromethyl)sulfonyl)azetidine (2a)

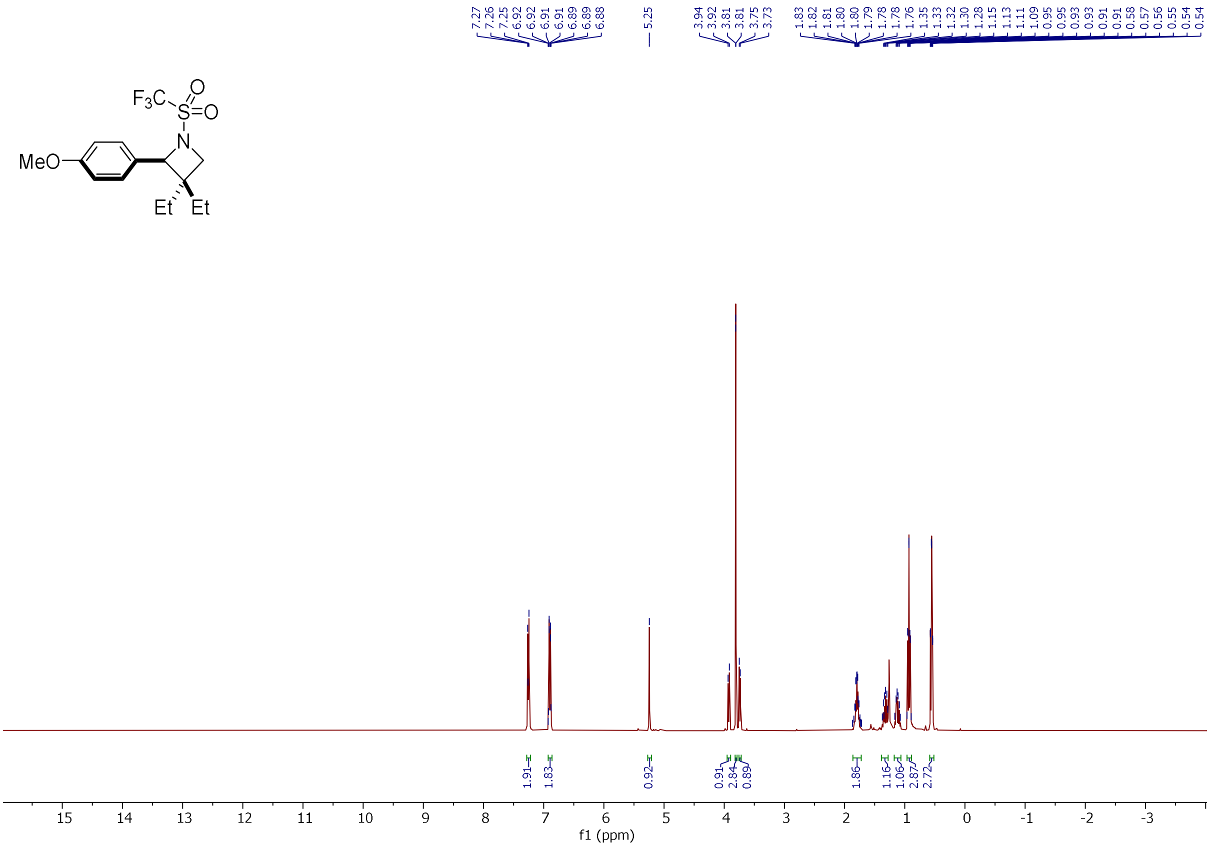

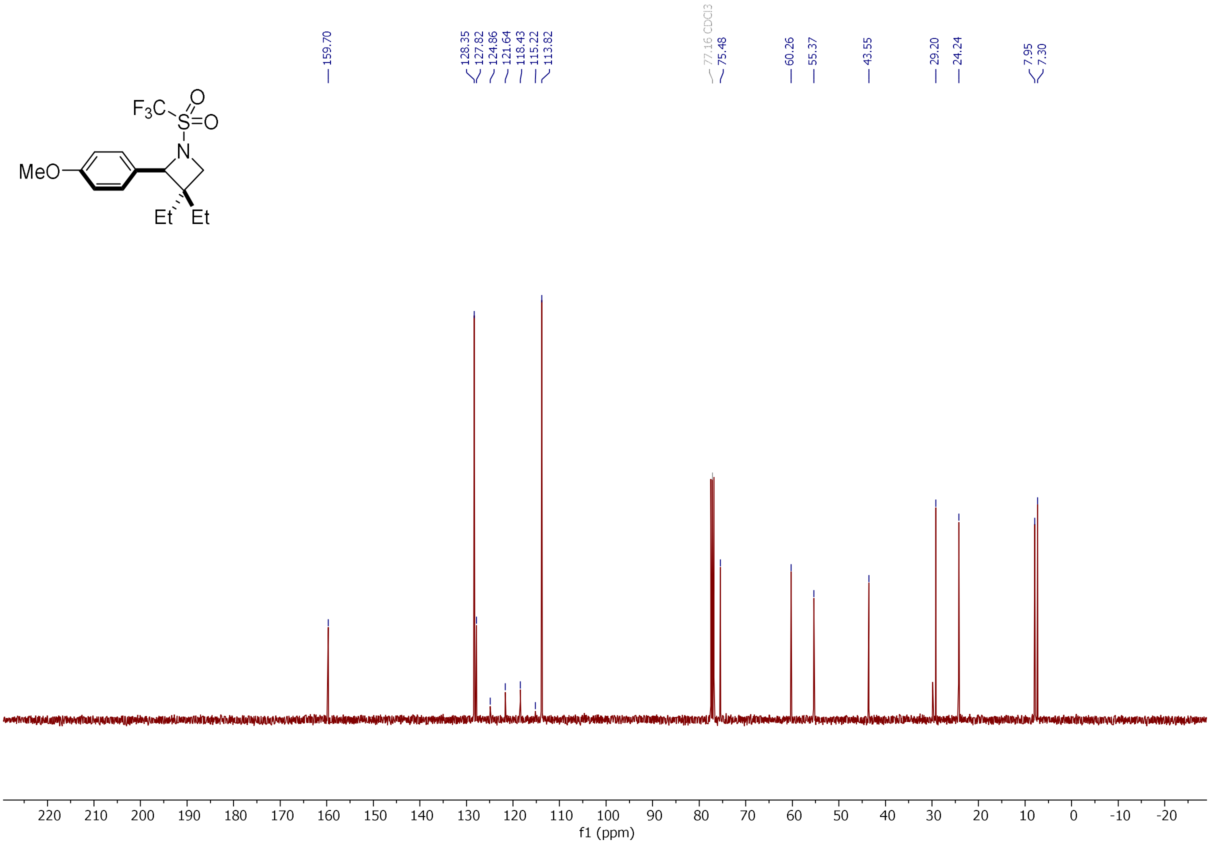

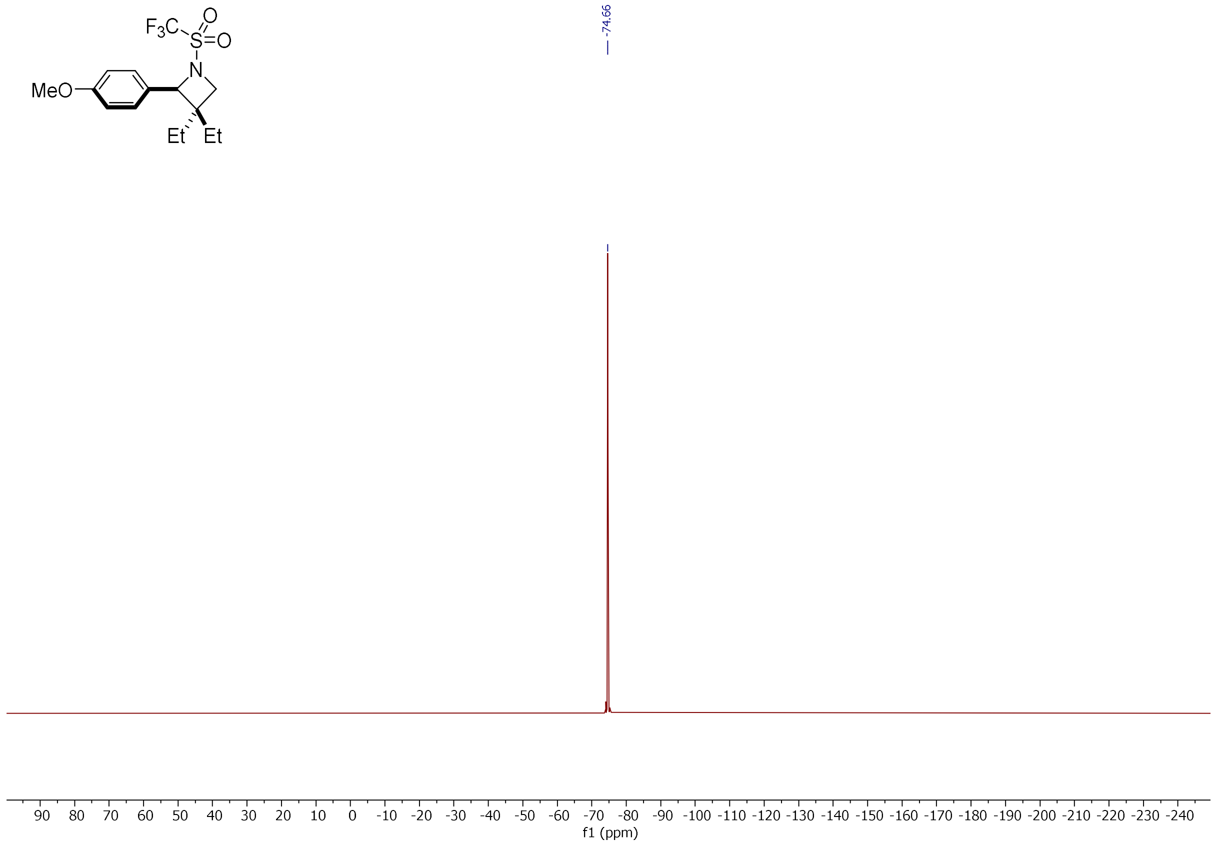

3,3-Diethyl-2-(4-methoxyphenyl)azetidine-1-sulfonyl fluoride (3a)

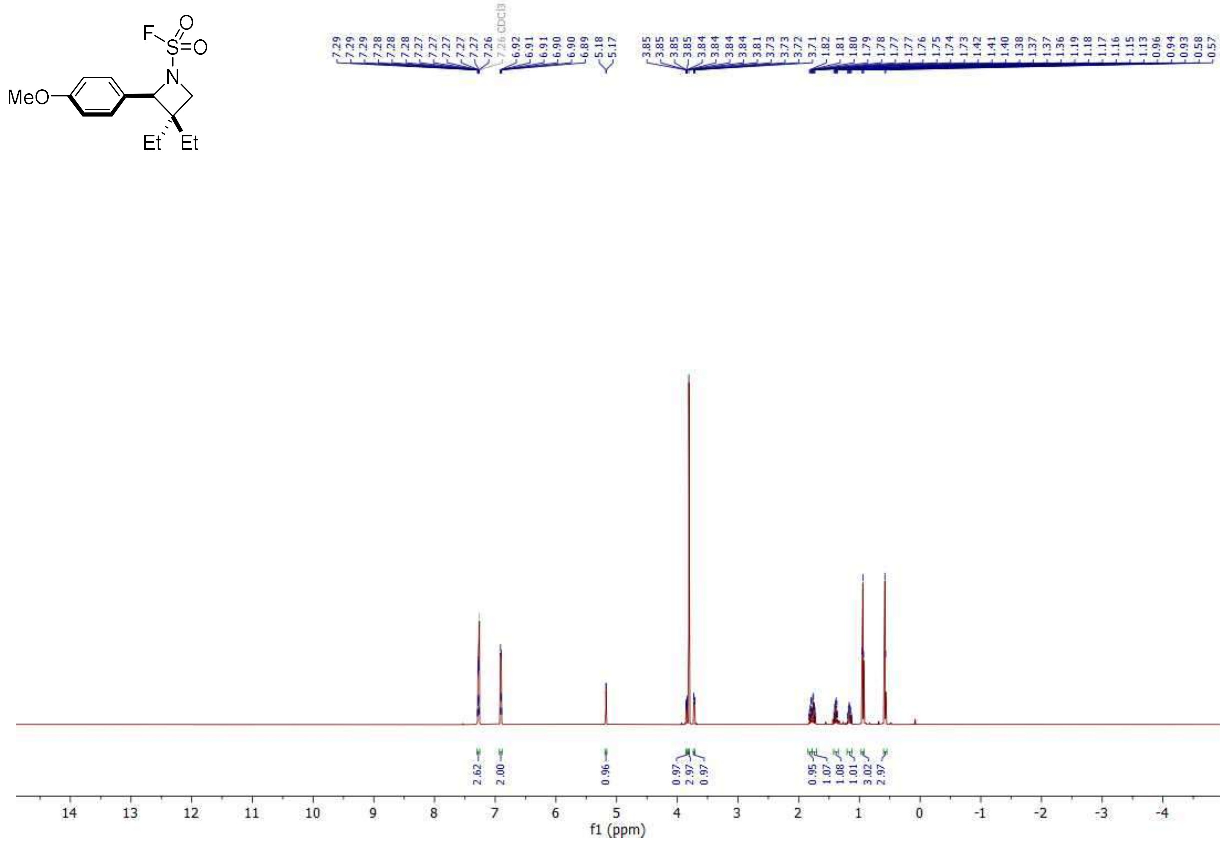

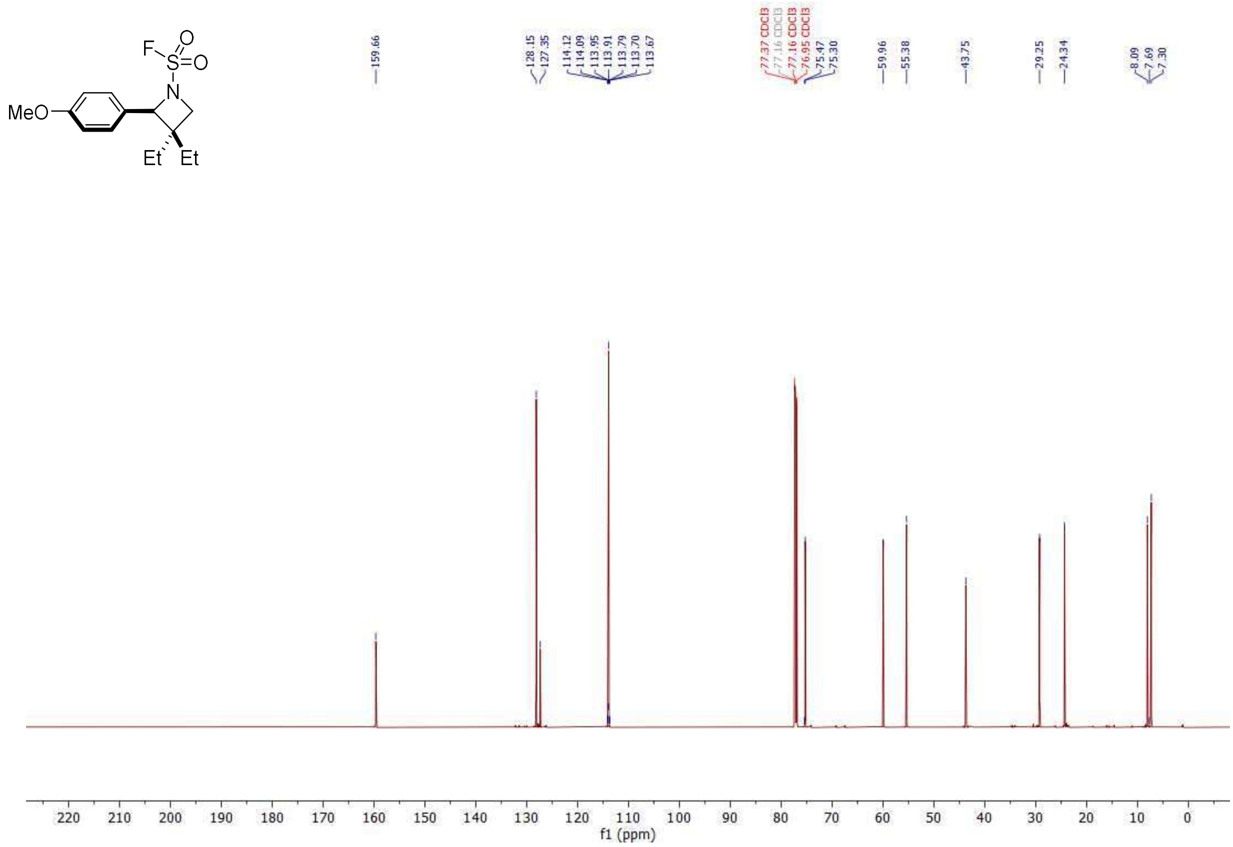
MeO
F
S
O
N
Et
Et
159.66
128.15
127.35
114.12
114.09
113.95
113.91
113.79
113.70
113.67
77.37 CDCl3
77.16 CDCl3
77.16 CDCl3
76.95 CDCl3
75.47
75.30
59.96
55.38
43.75
29.25
24.34
8.09
7.69
7.30
f1 (ppm)

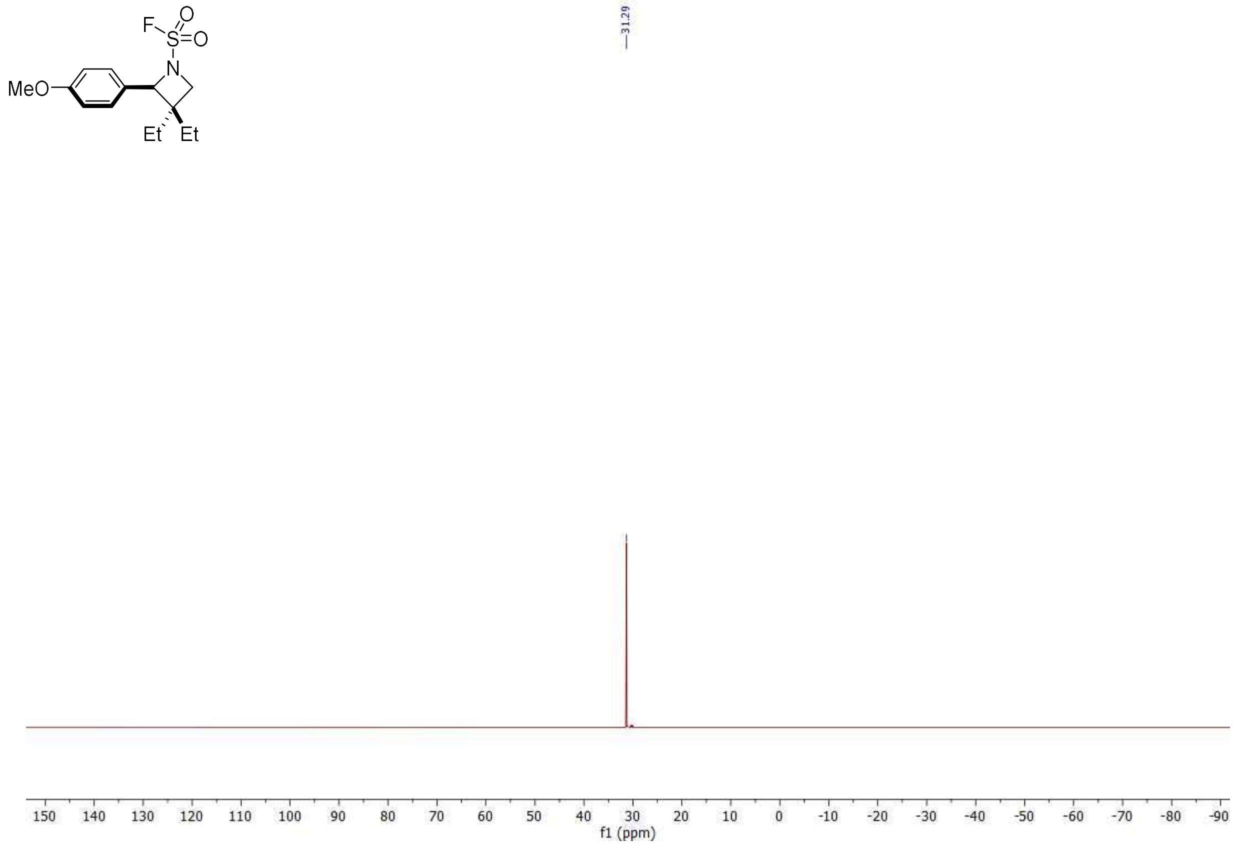
MeO
F
S
O
N
Et
Et
31.29
f1 (ppm)

3,3-Diethyl-2-(2-methoxyphenyl)azetidine-1-sulfonyl fluoride (3b)

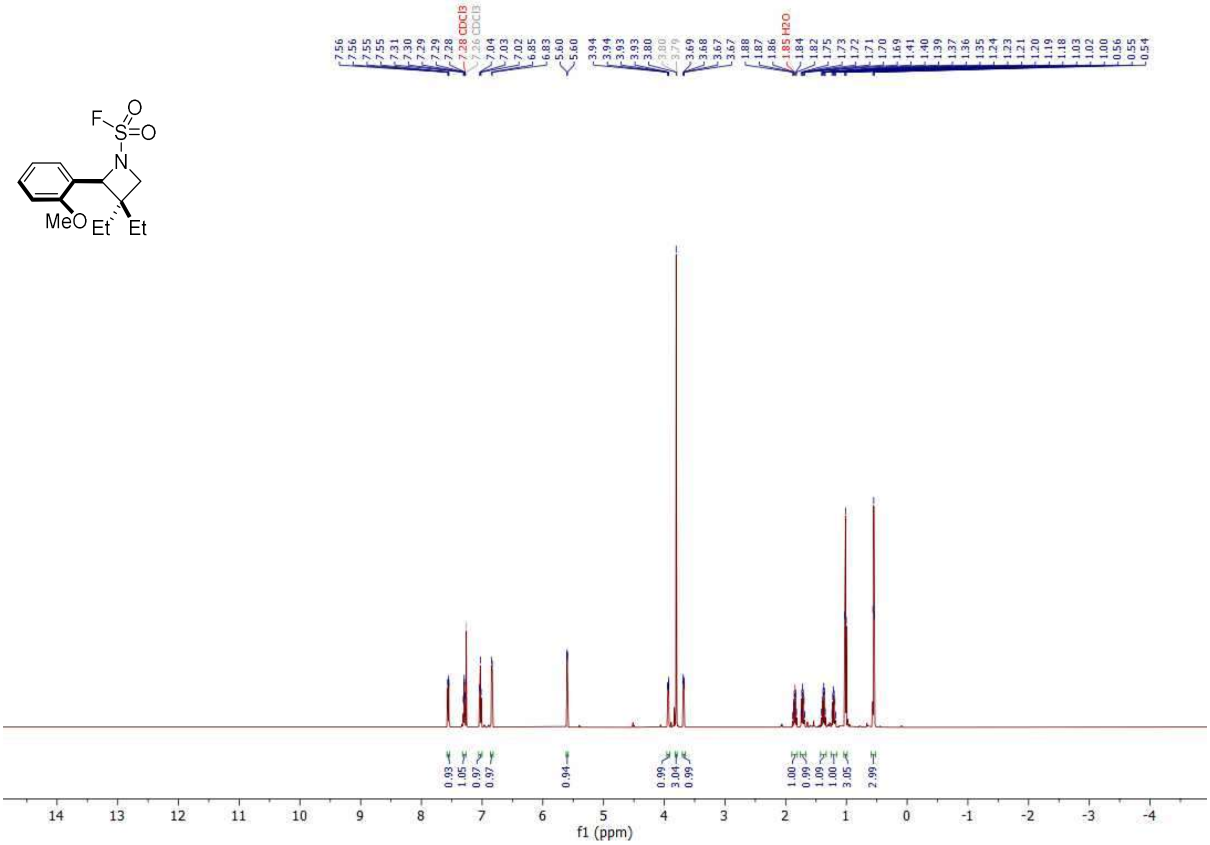

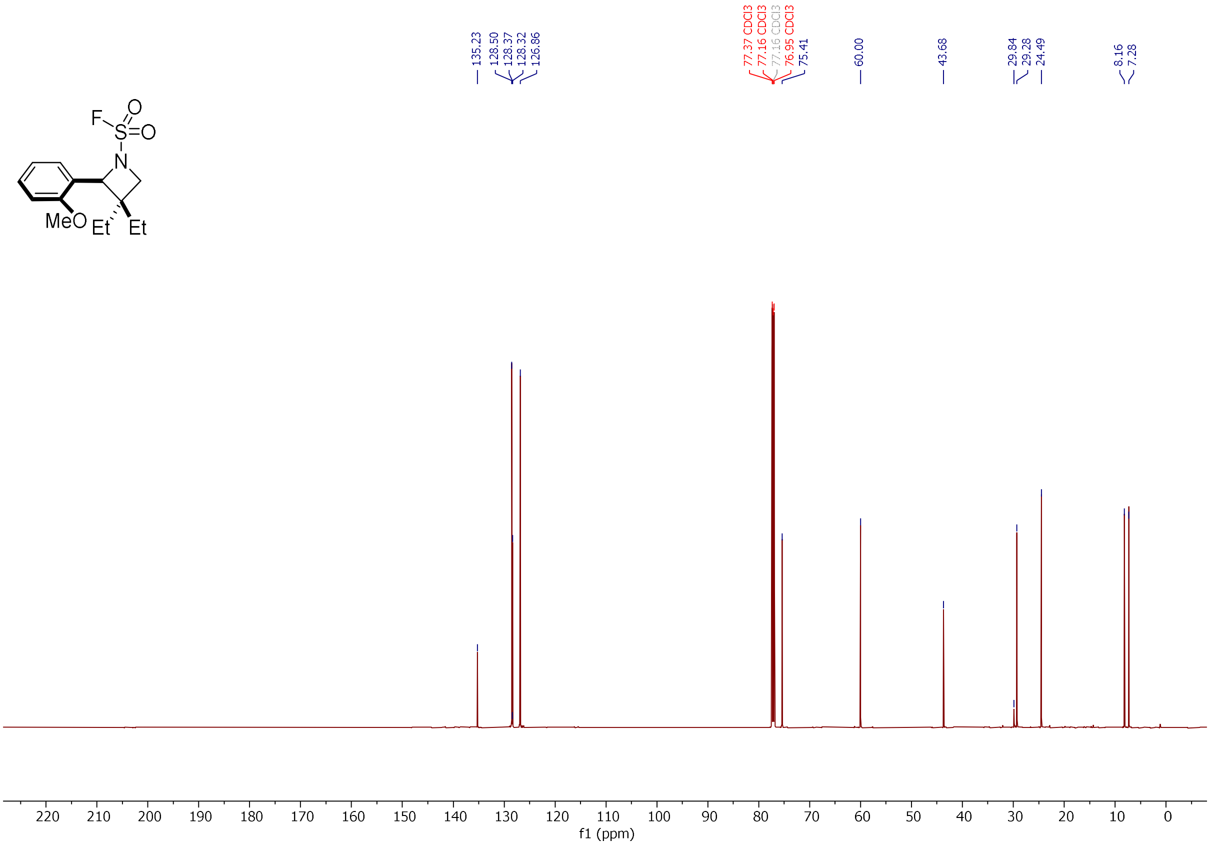

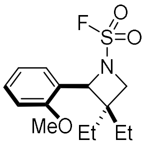
F
O
S
O
N
MeO
Et
Et

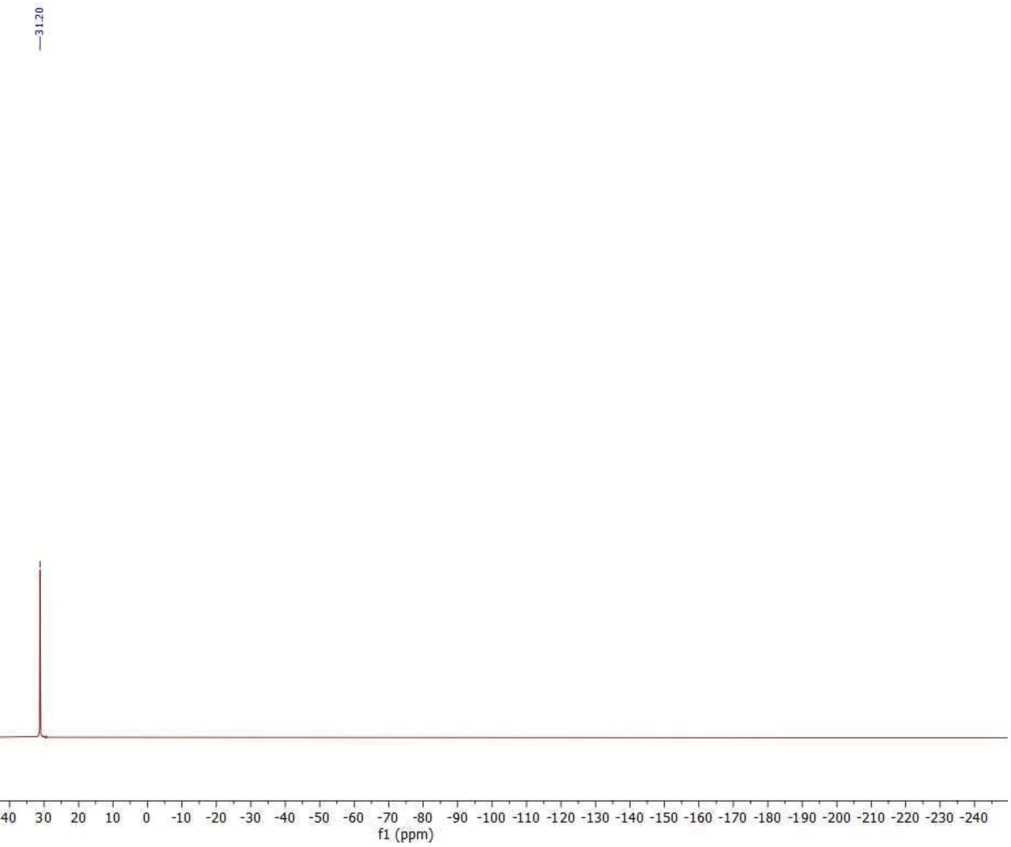
31.20
90 80 70 60 50 40 30 20 10 0 -10 -20 -30 -40 -50 -60 -70 -80 -90 -100 -110 -120 -130 -140 -150 -160 -170 -180 -190 -200 -210 -220 -230 -240
f1 (ppm)

3,3-Diethyl-2-(2-fluoro-6-methoxyphenyl)azetidine-1-sulfonyl fluoride (3c)

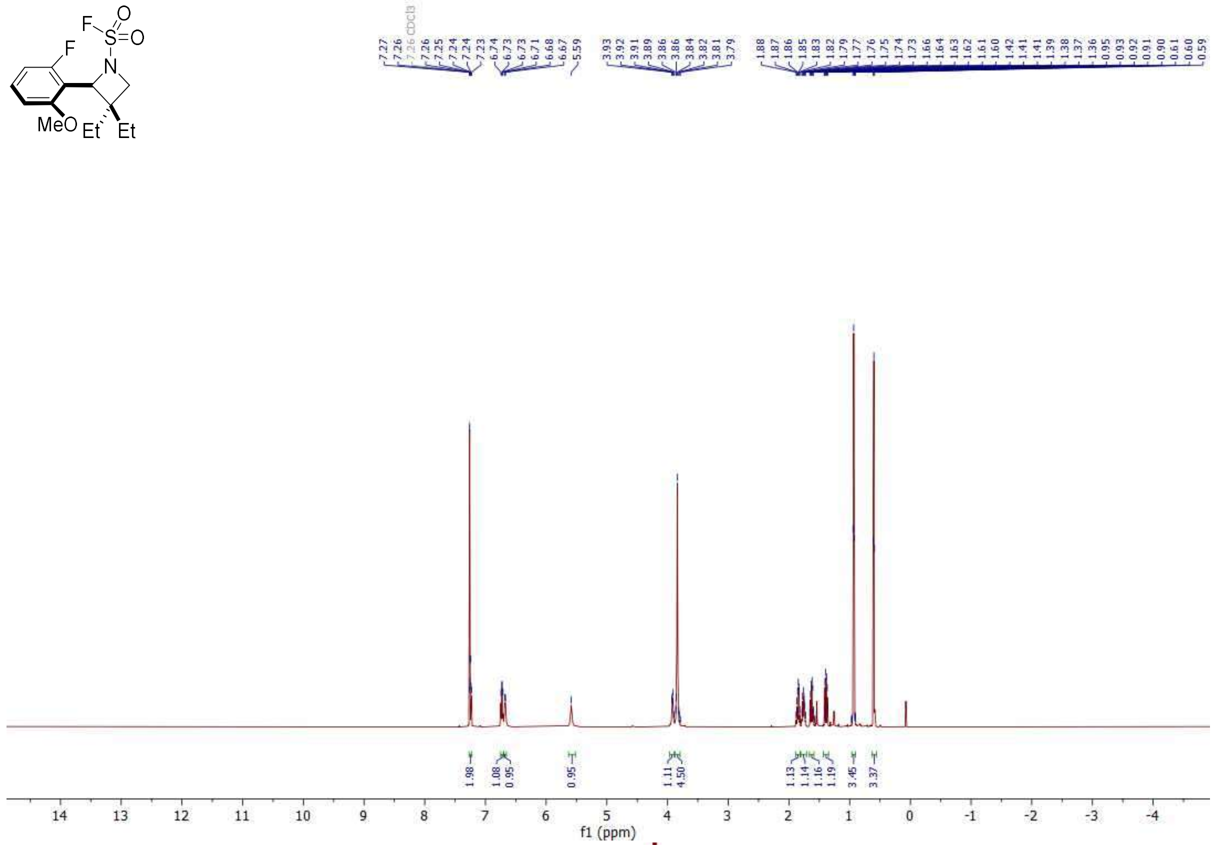

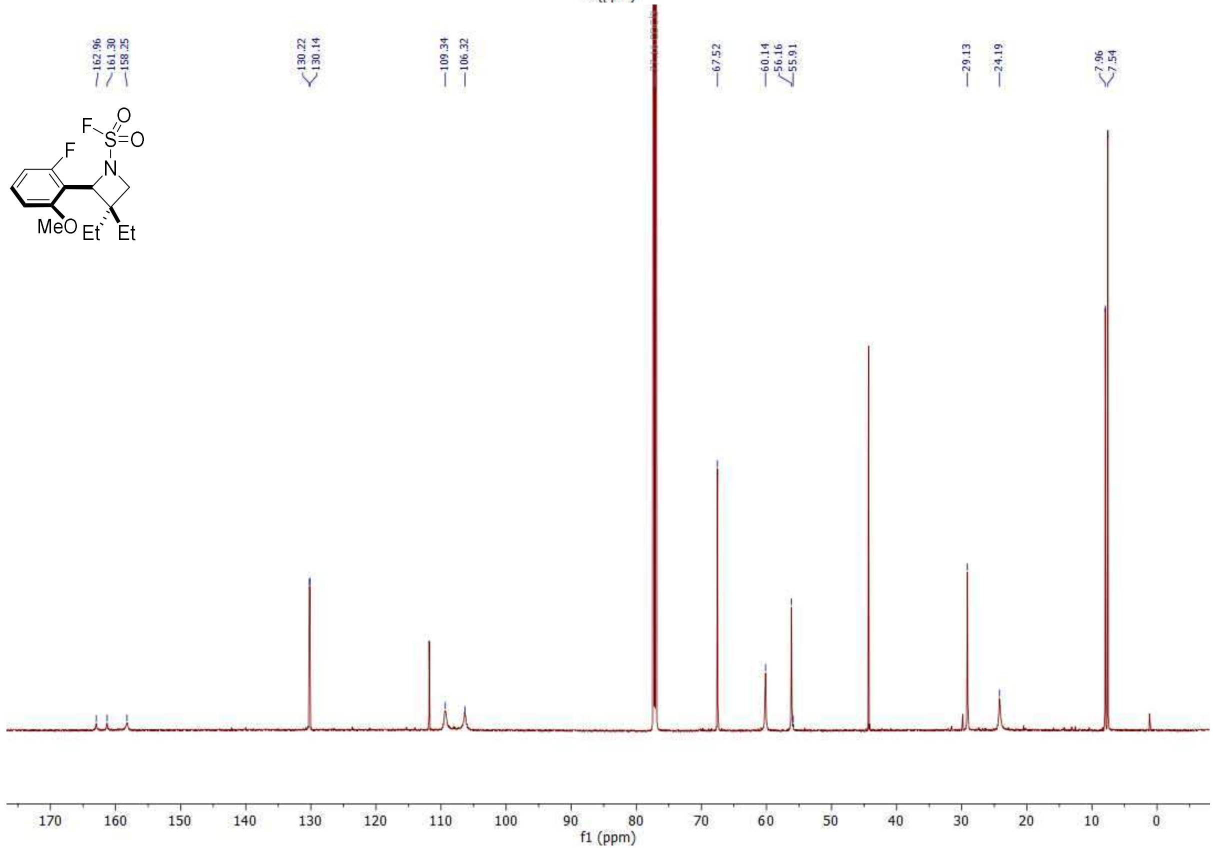

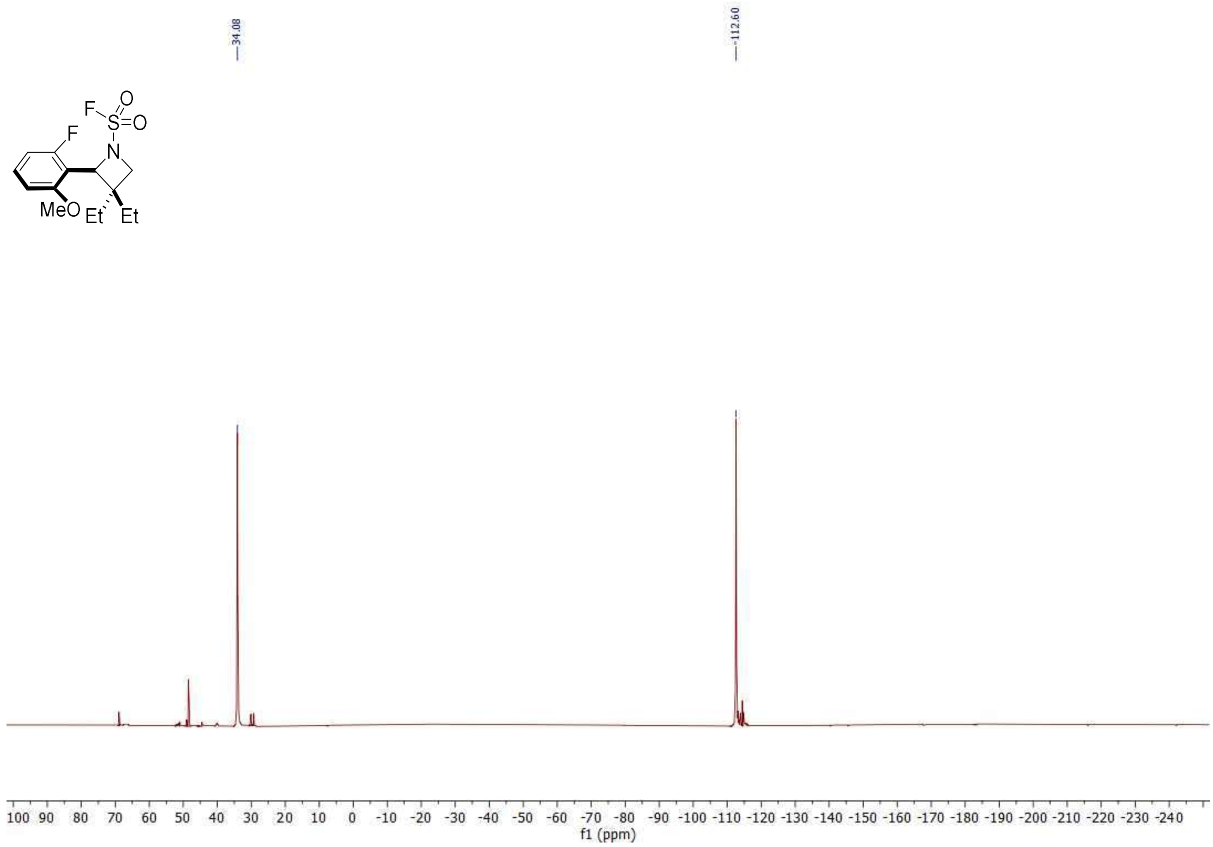
34.08
-112.60
F
O
S=O
F
N
MeO
Et
Et
100 90 80 70 60 50 40 30 20 10 0 -10 -20 -30 -40 -50 -60 -70 -80 -90 -100 -110 -120 -130 -140 -150 -160 -170 -180 -190 -200 -210 -220 -230 -240
f1 (ppm)

3,3-Diethyl-2-phenylazetidine-1-sulfonyl fluoride (3d)

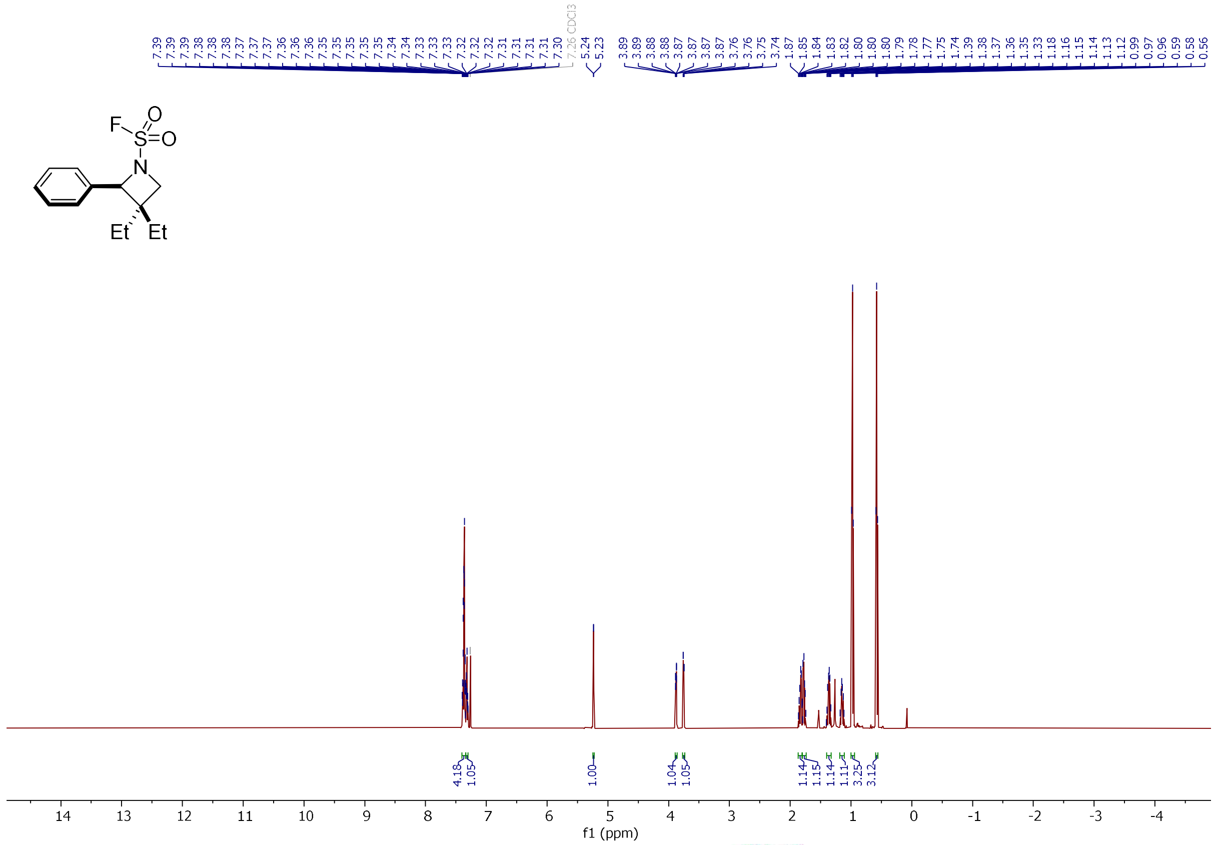

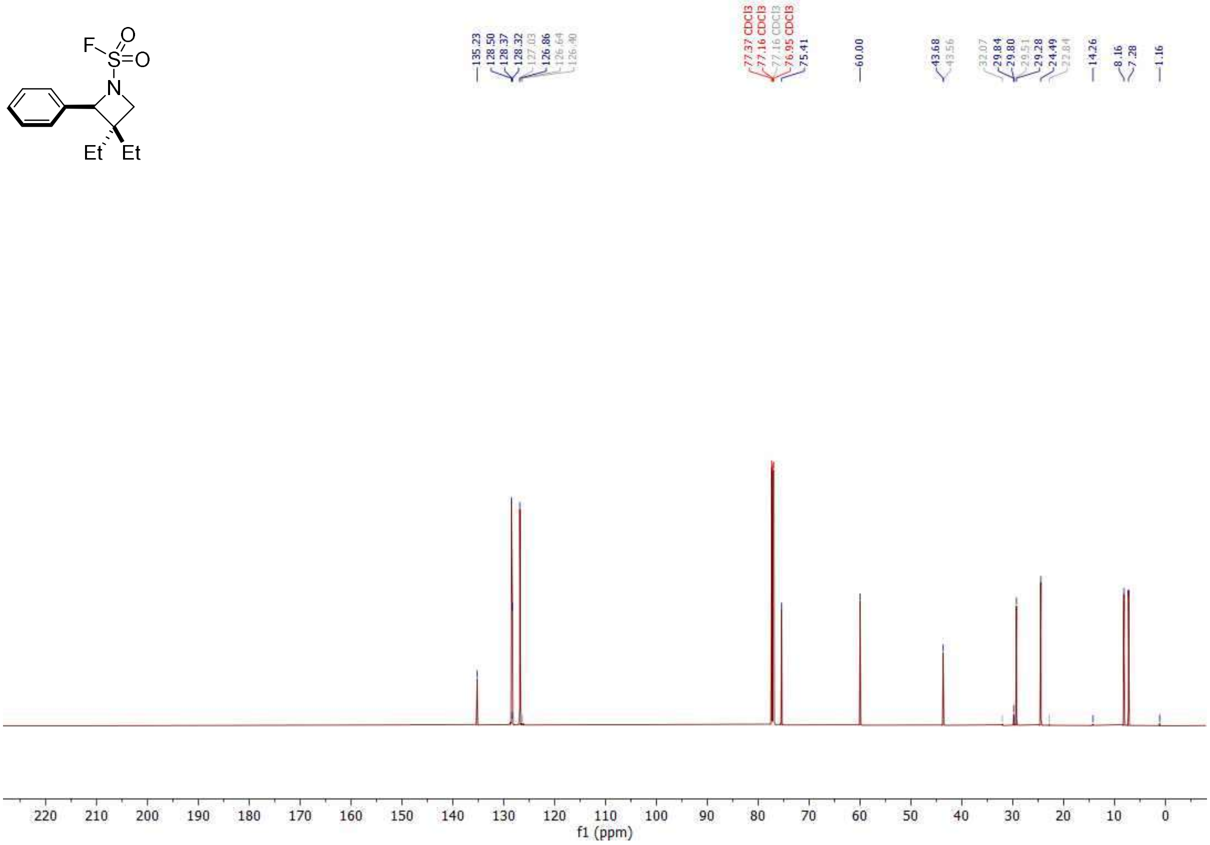

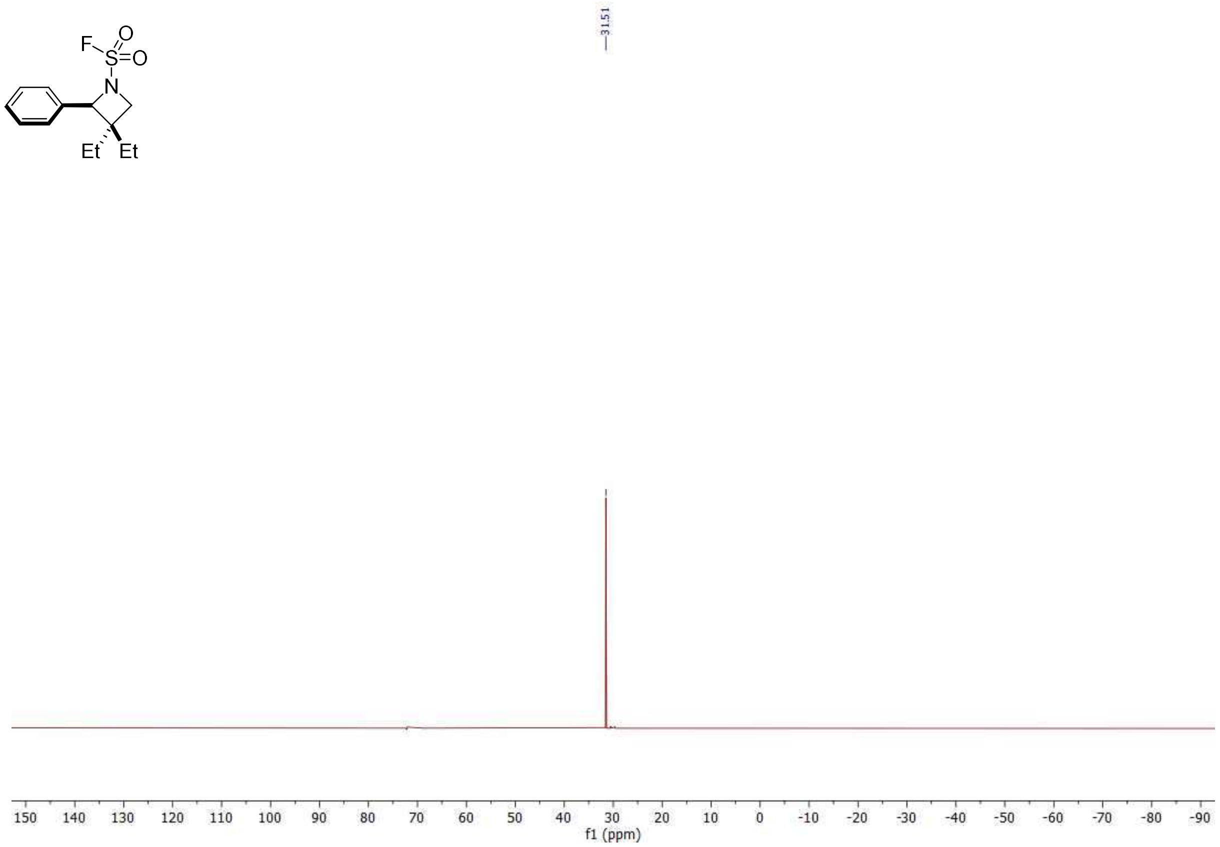

3,3-Diethyl-2-(p-tolyl)azetidine-1-sulfonyl fluoride (3e)

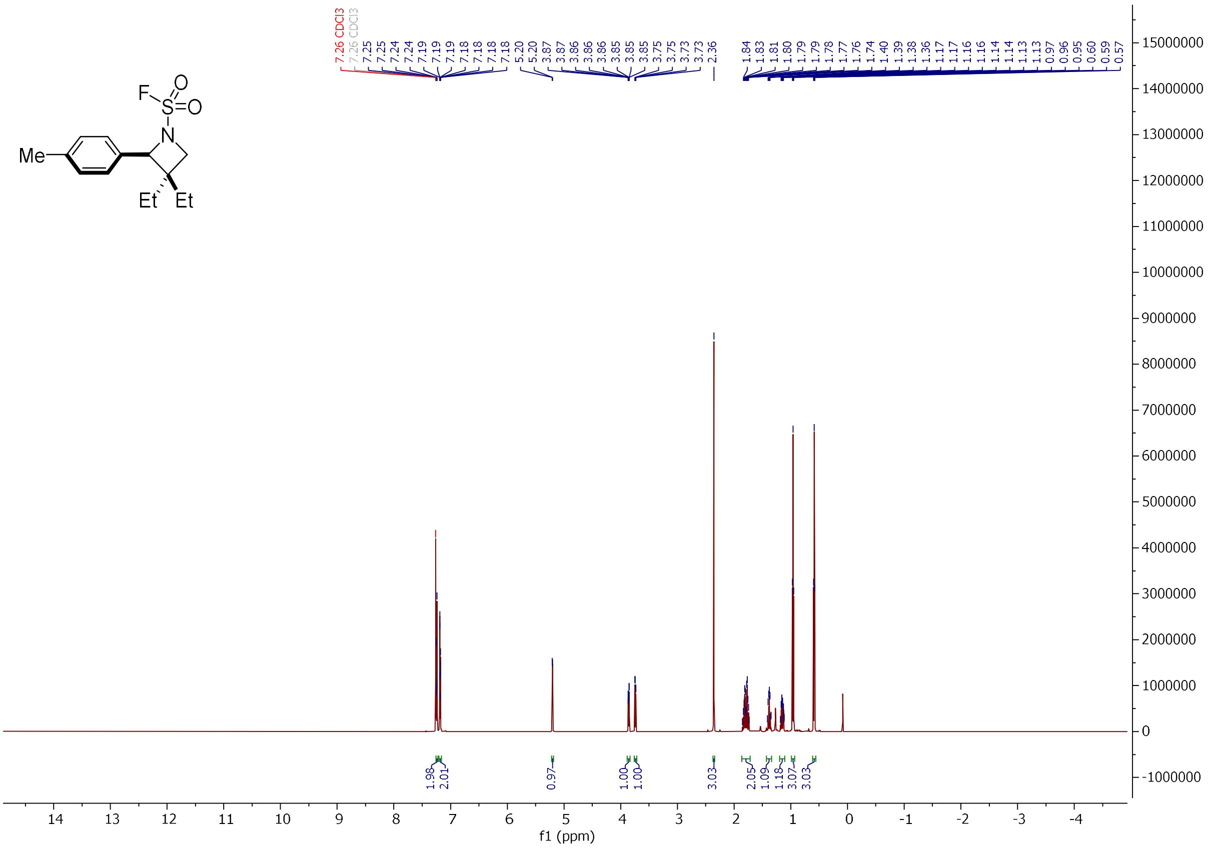

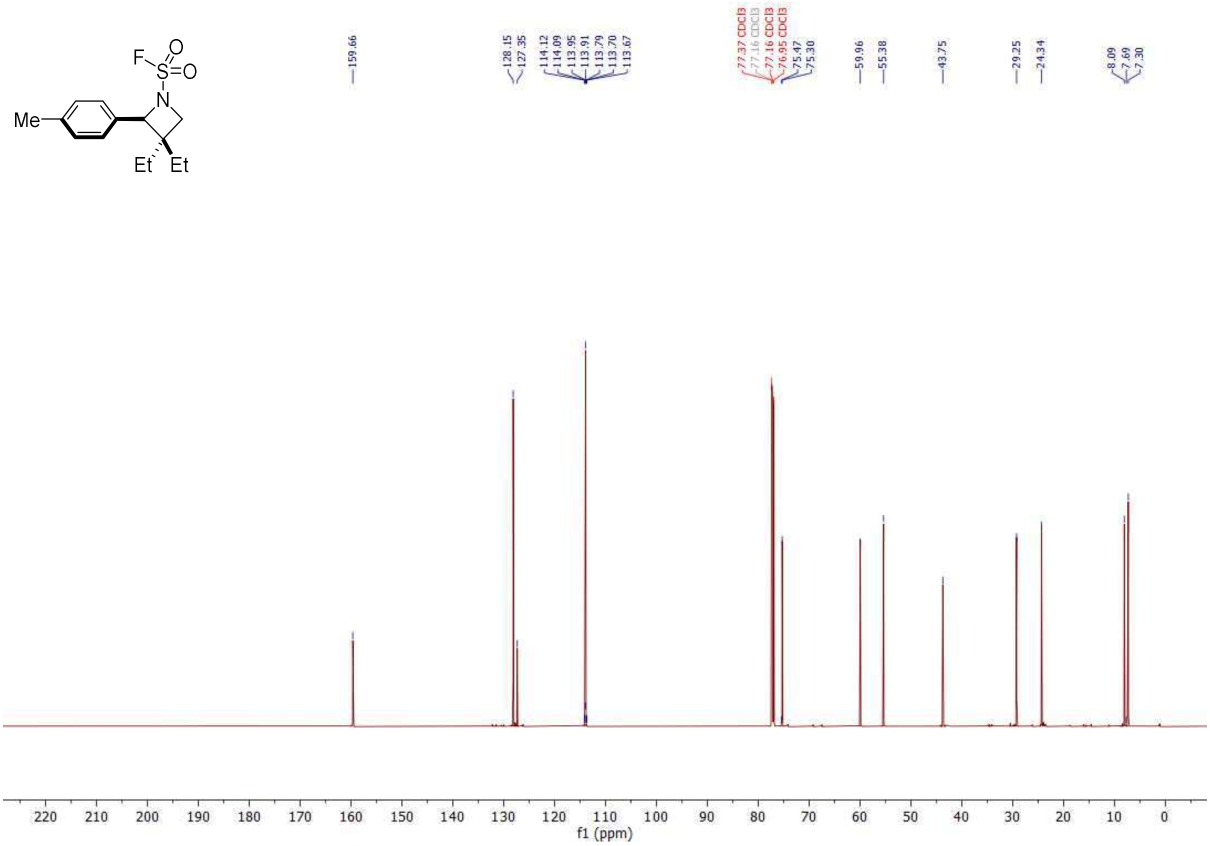

F
S=O
O
N
Me
Et
Et
159.66
128.15
127.35
114.12
114.09
113.95
113.91
113.79
113.70
113.67
77.37 CDCl3
77.16 CDCl3
77.16 CDCl3
76.95 CDCl3
75.47
75.30
59.96
55.38
43.75
29.25
24.34
8.09
7.69
7.30
220 210 200 190 180 170 160 150 140 130 120 110 100 90 80 70 60 50 40 30 20 10 0
f1 (ppm)

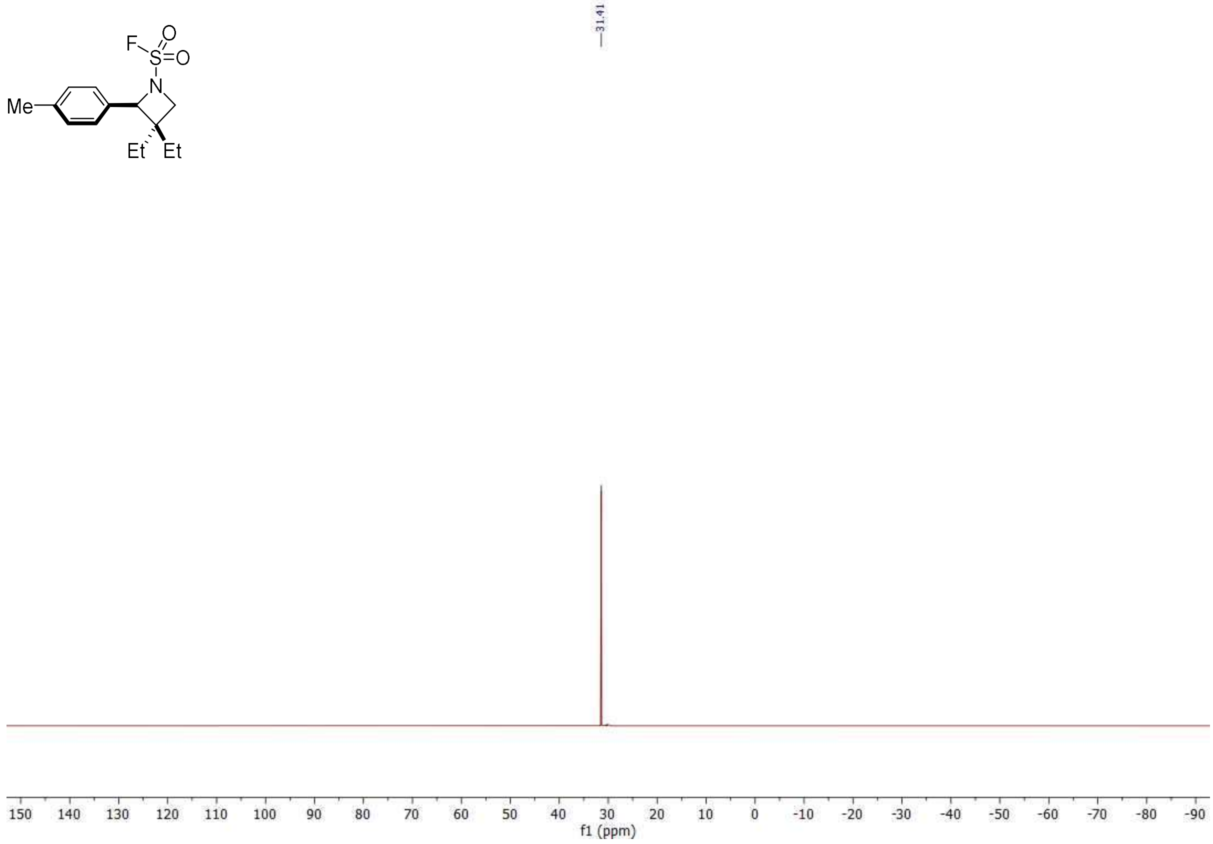

F
S=O
O
N
Me
Et
Et
31.41
150 140 130 120 110 100 90 80 70 60 50 40 30 20 10 0 -10 -20 -30 -40 -50 -60 -70 -80 -90
f1 (ppm)

3,3-Diethyl-2-(4-(trimethylsilyl)phenyl)azetidine-1-sulfonyl fluoride (3f)

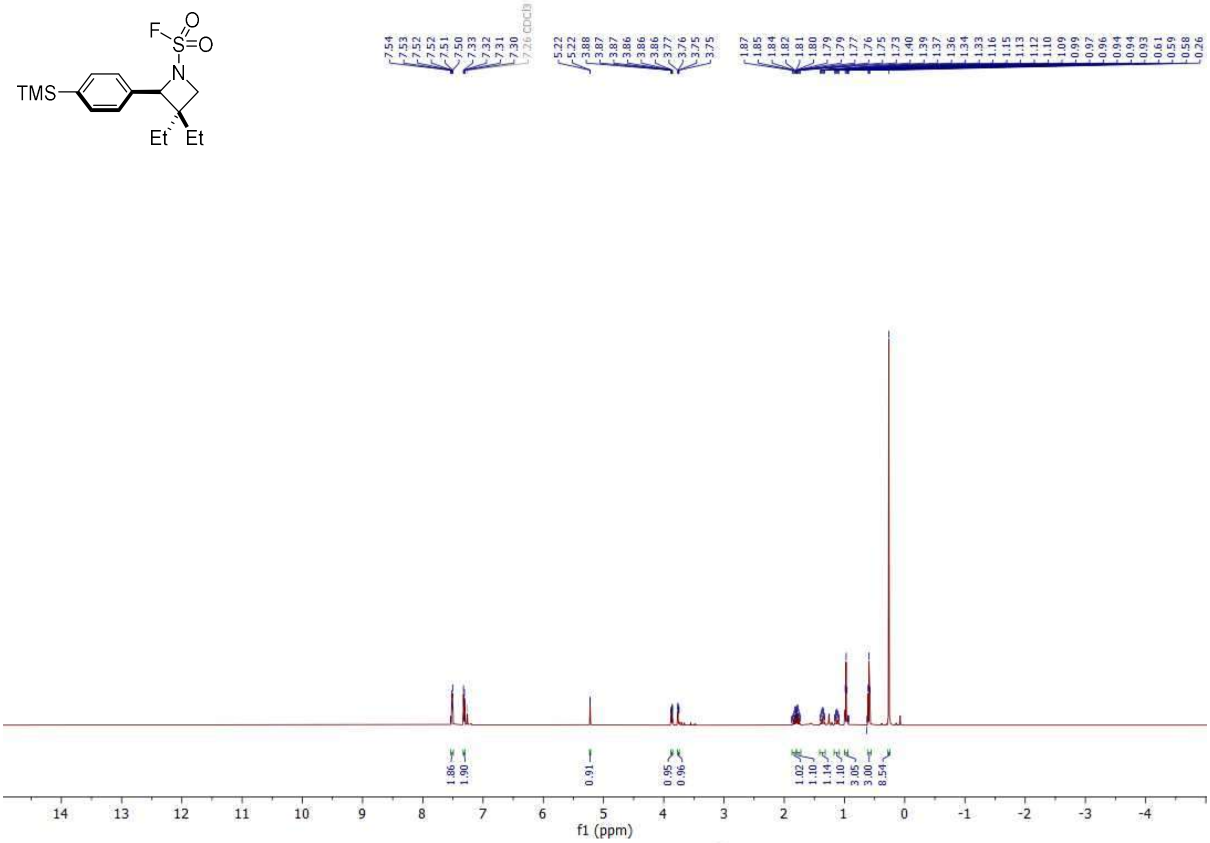

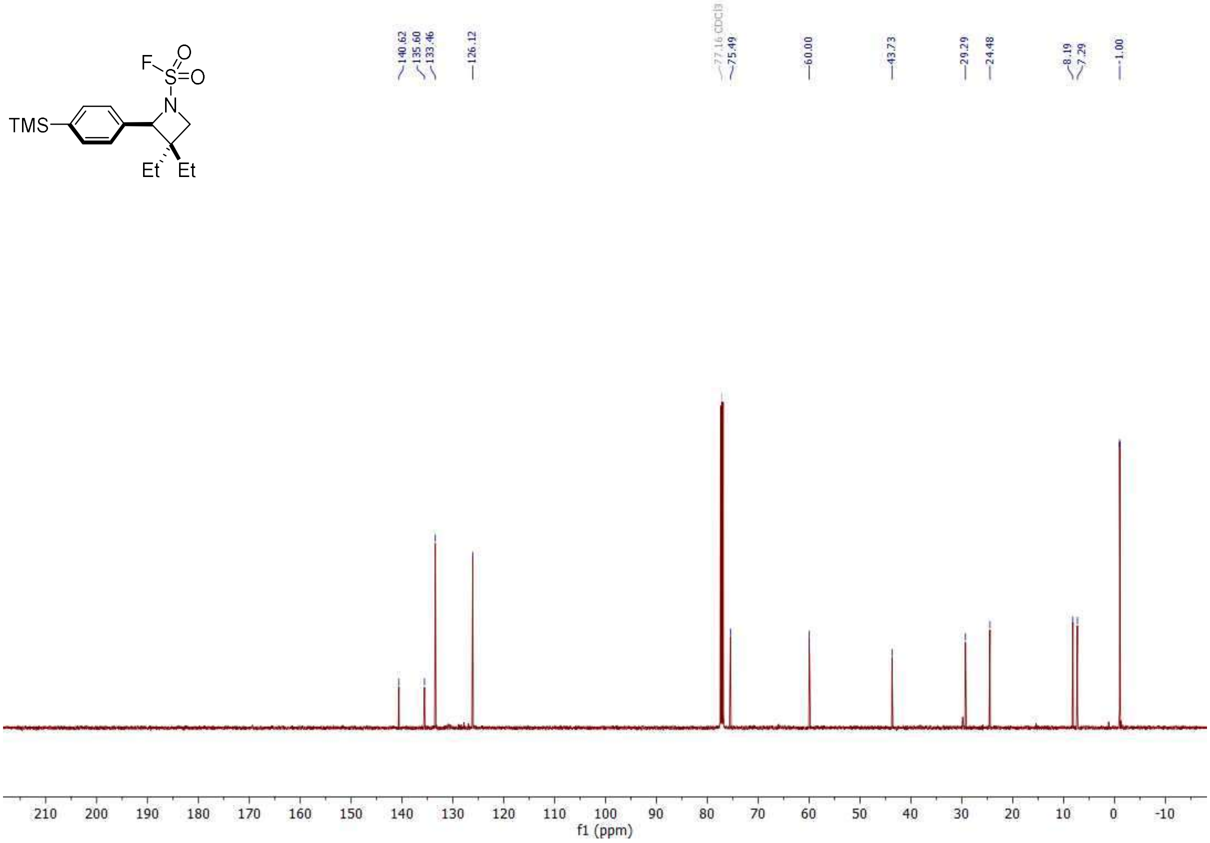

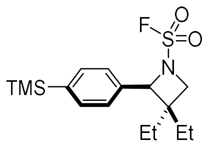

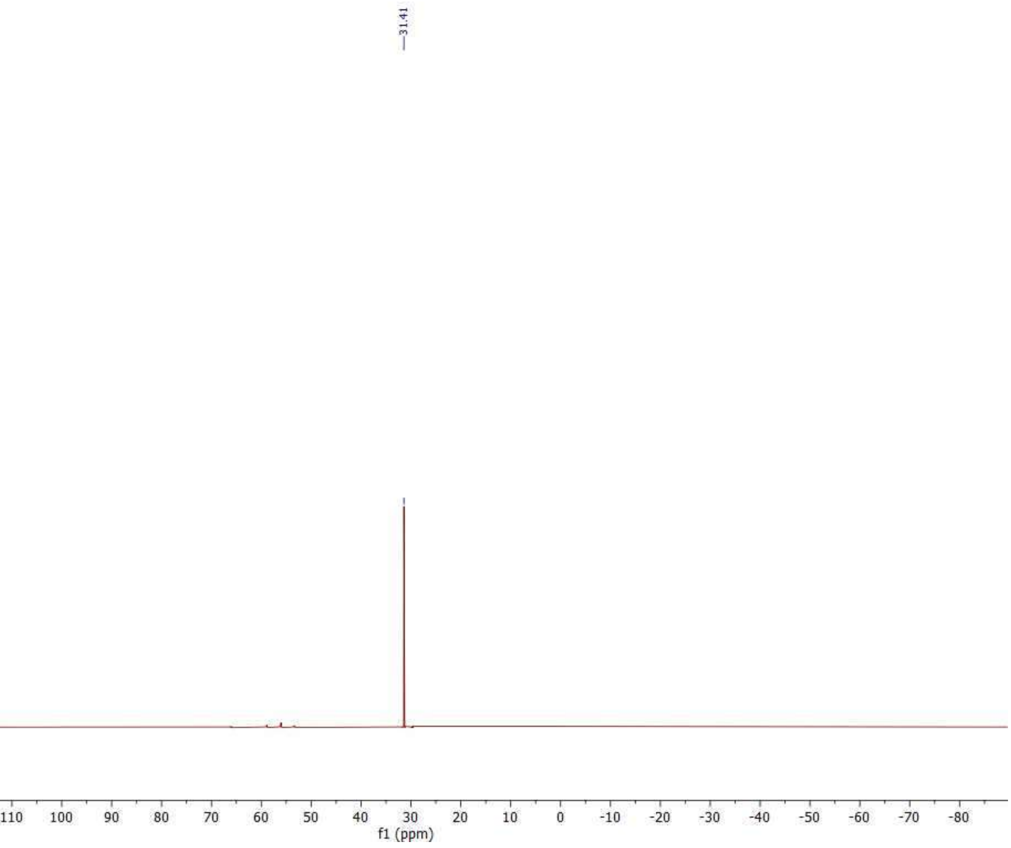

31.41
150
140
130
120
110
100
90
80
70
60
50
40
30
20
10
0
-10
-20
-30
-40
-50
-60
-70
-80
f1 (ppm)

3,3-Diethyl-2-(4-fluorophenyl)azetidine-1-sulfonyl fluoride (3g)

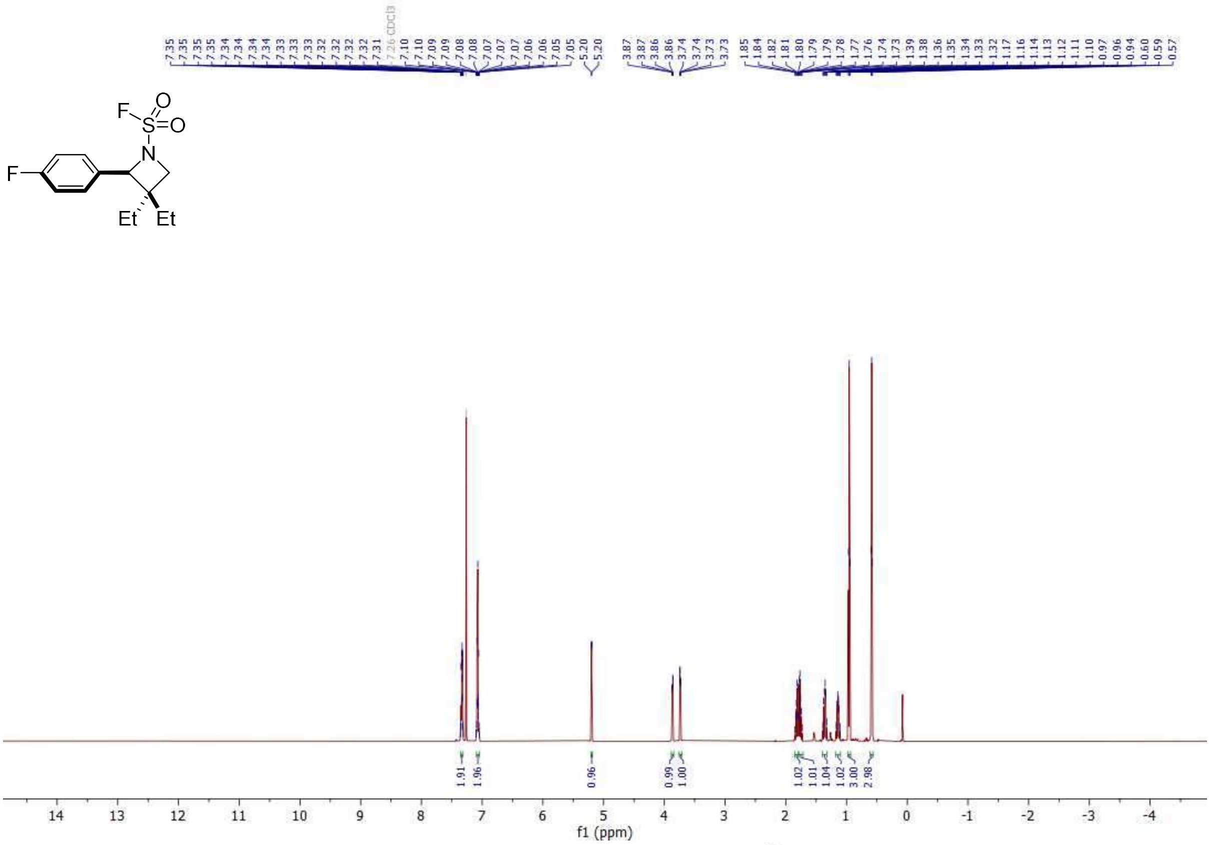

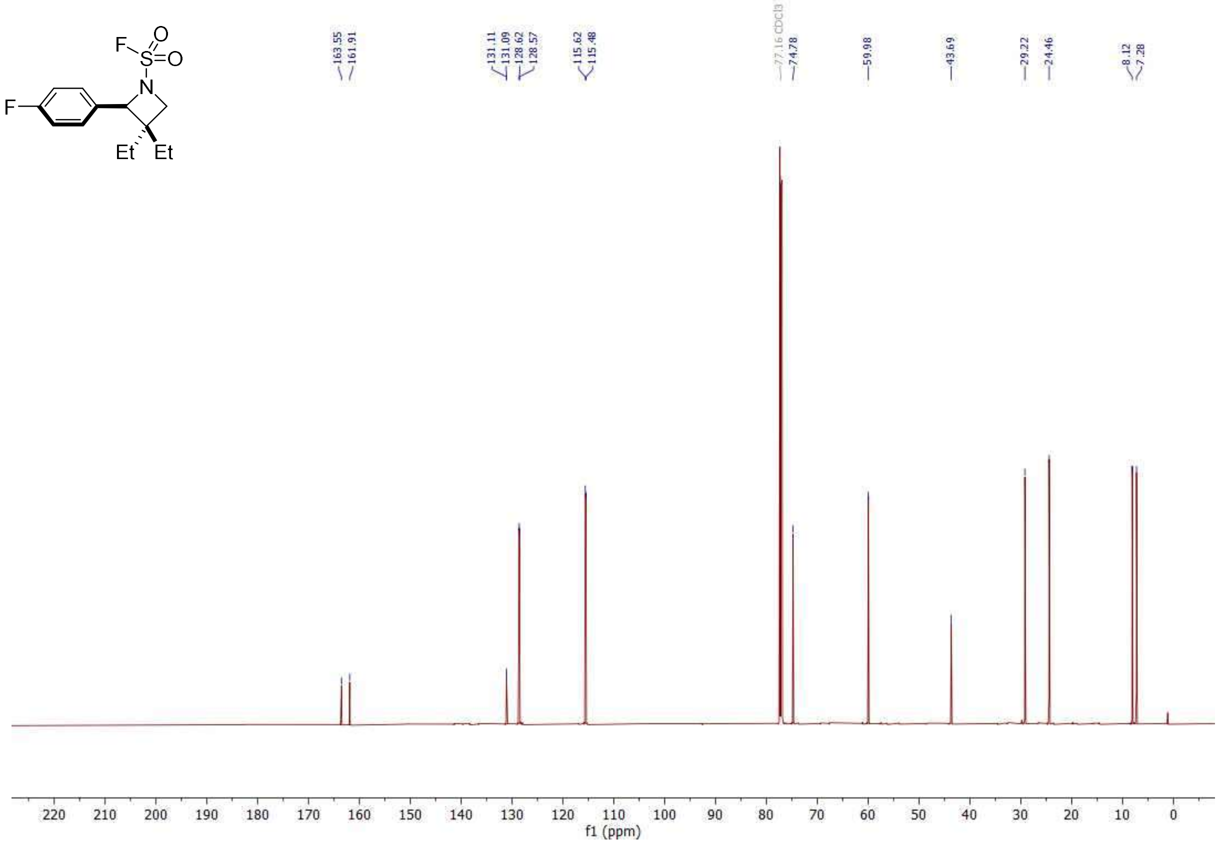

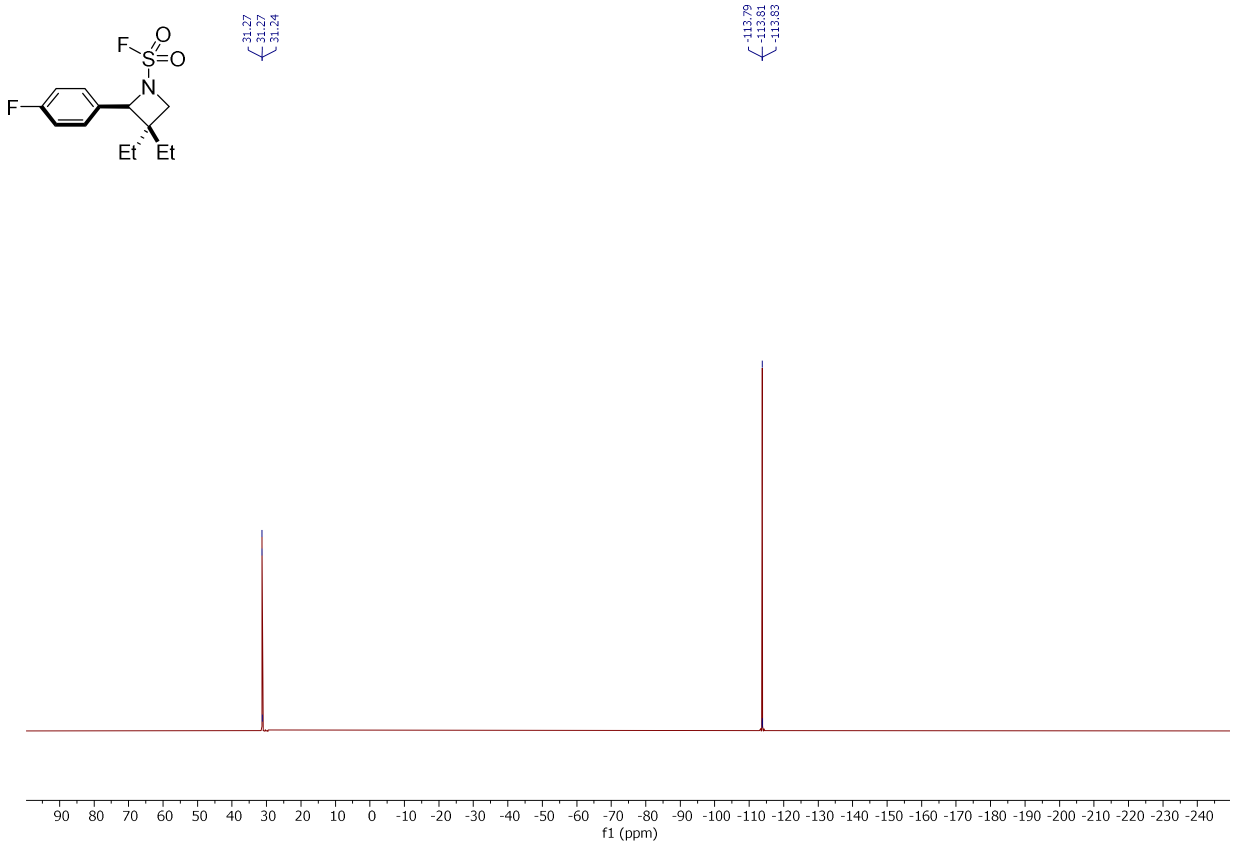

2-(4-Chlorophenyl)-3,3-diethylazetidine-1-sulfonyl fluoride (3h)

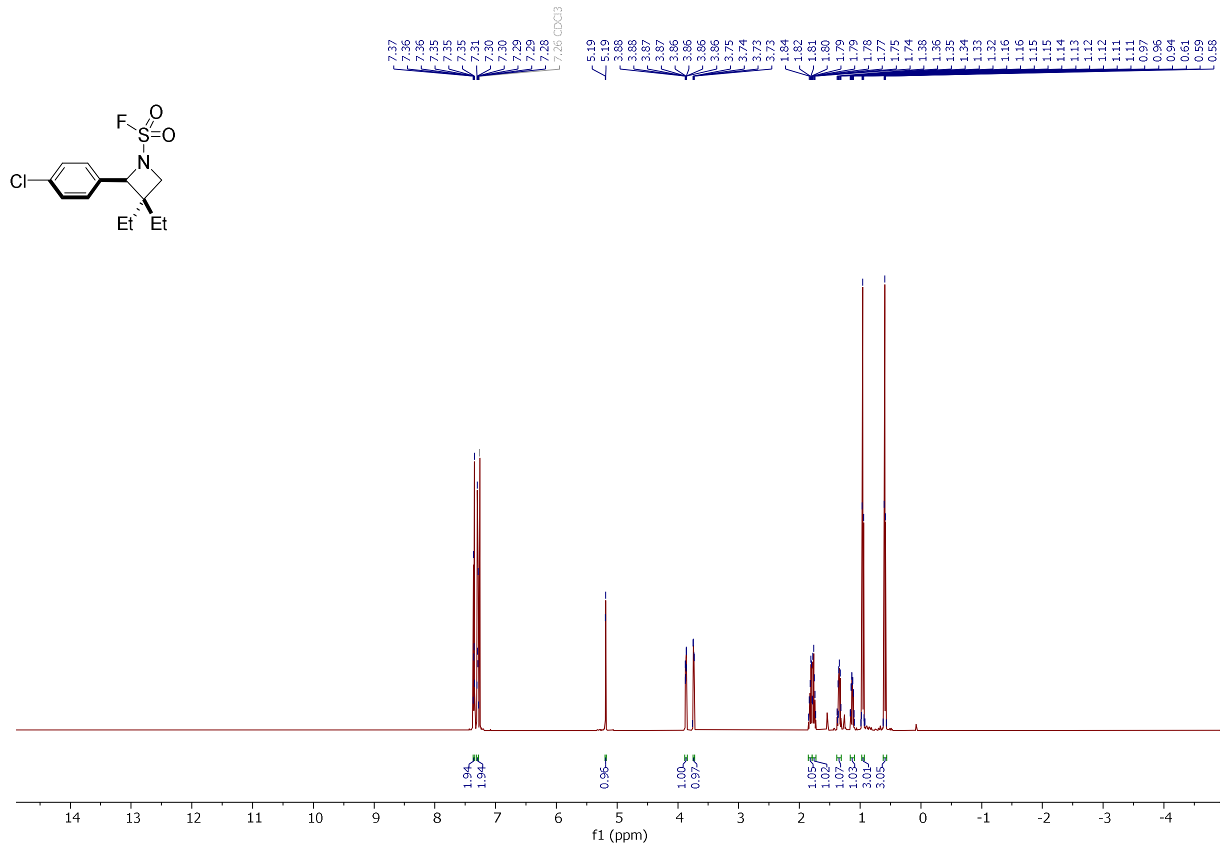

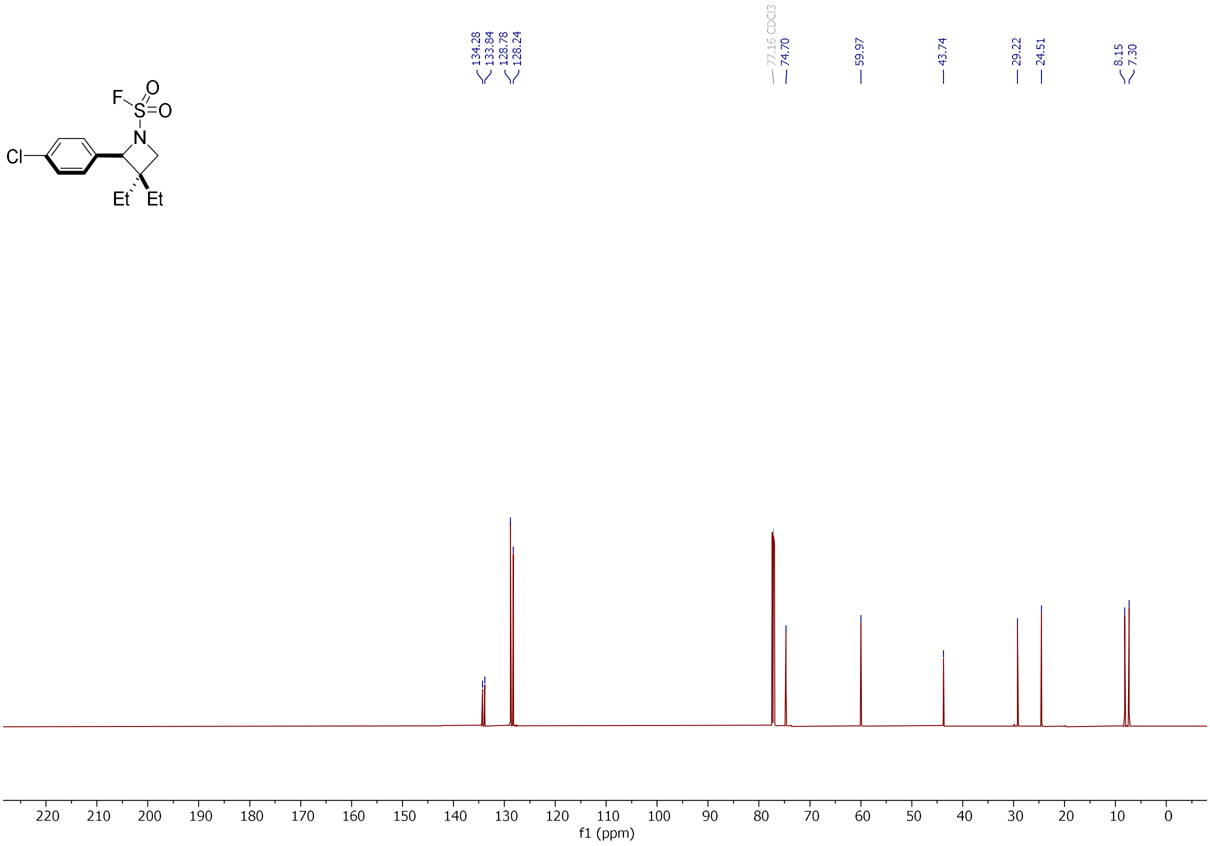
134.28
133.84
128.78
128.24
77.16 CDCl3
74.70
59.97
43.74
29.22
24.51
8.15
7.30
f1 (ppm)

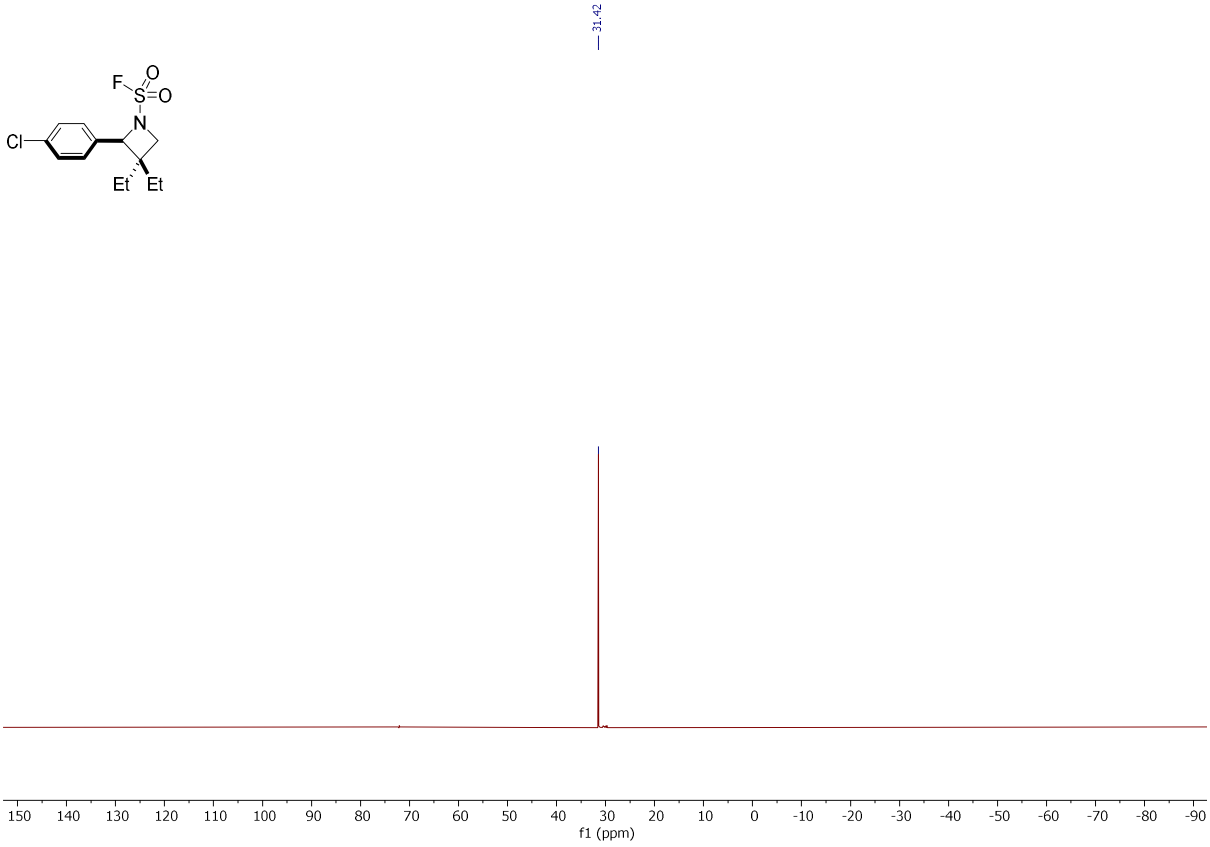
31.42
f1 (ppm)

2-(4-Bromophenyl)-3,3-diethylazetidine-1-sulfonyl fluoride (3l)

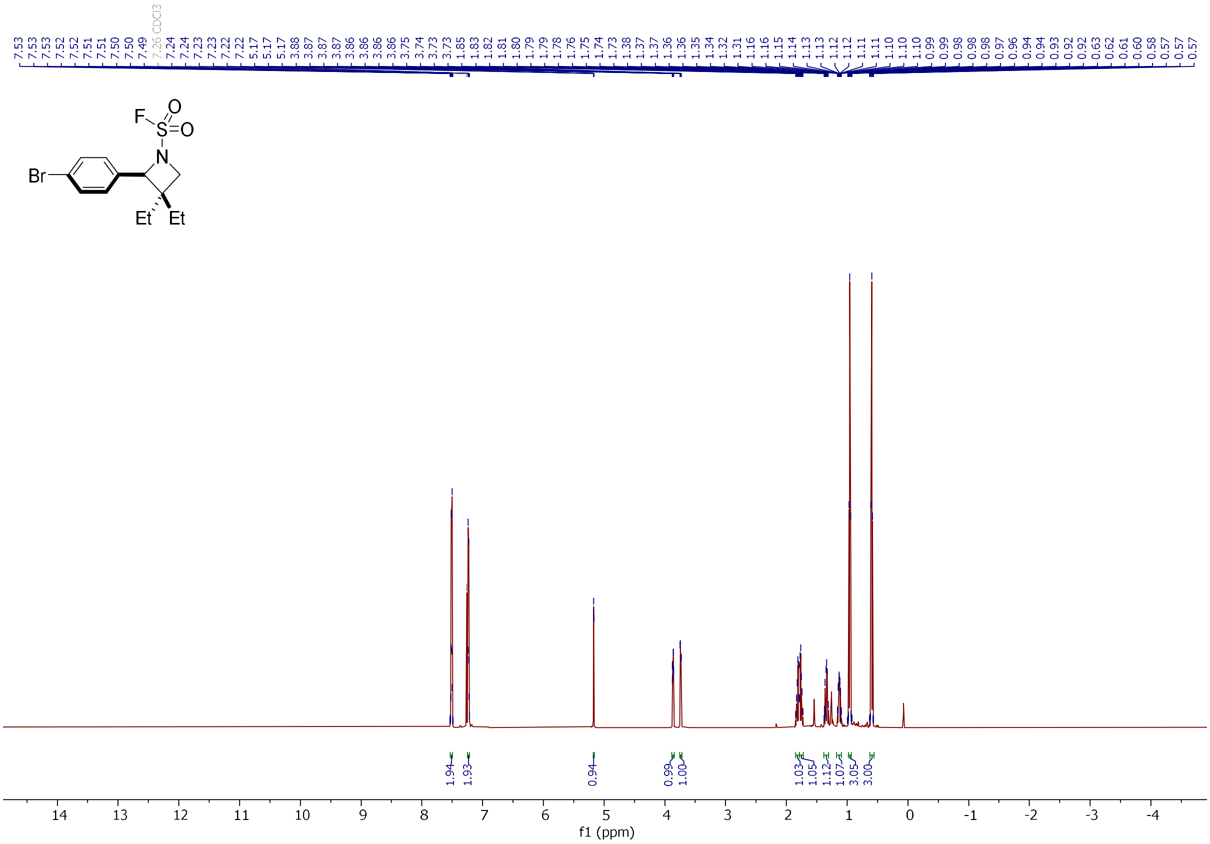

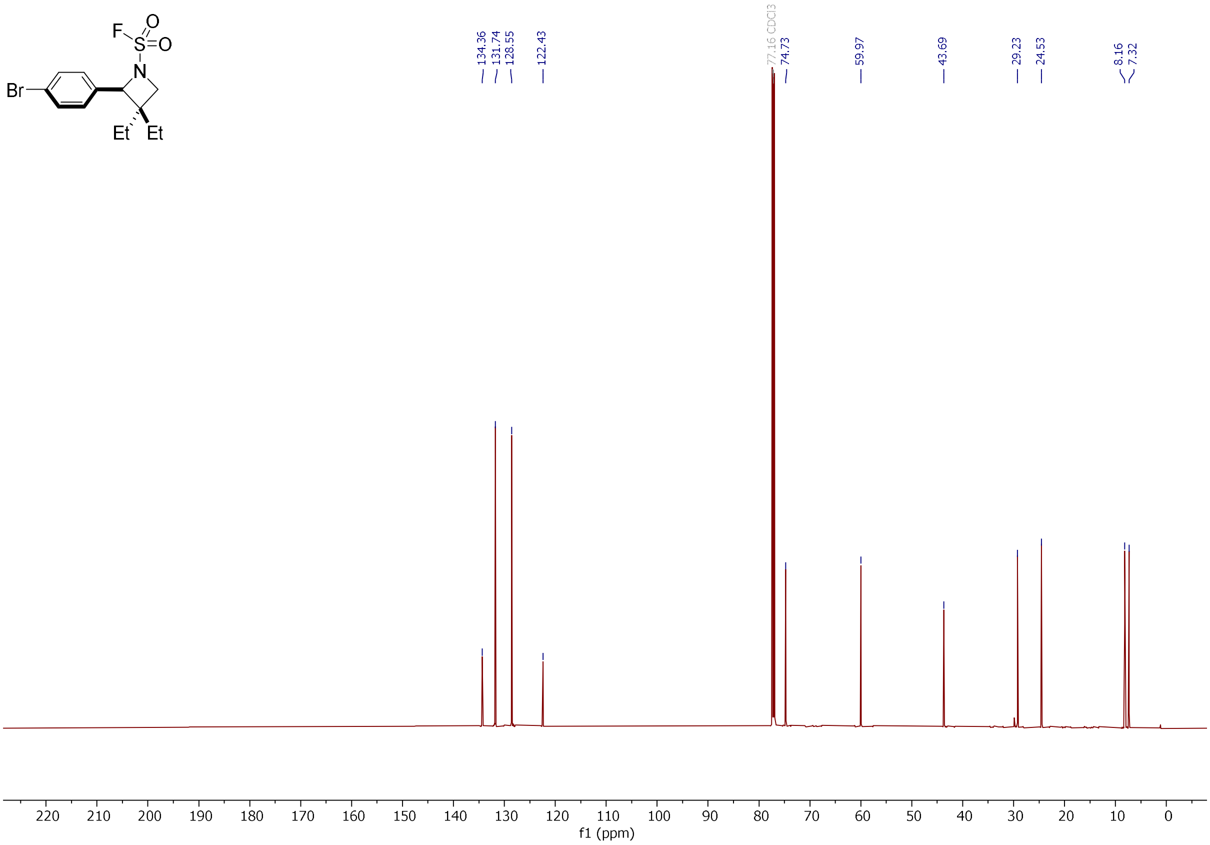

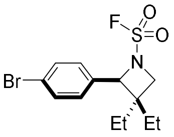
F
O
S
O
N
Br
Et
Et

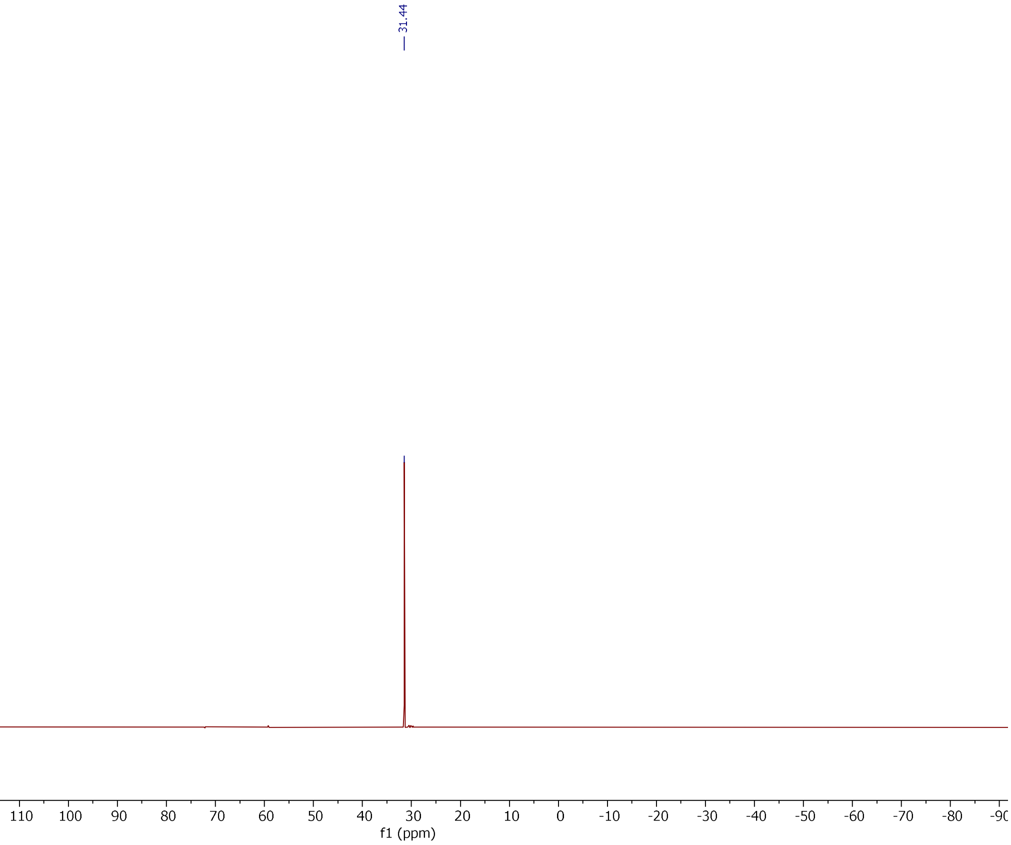
31.44
150
140
130
120
110
100
90
80
70
60
50
40
30
20
10
0
-10
-20
-30
-40
-50
-60
-70
-80
-90
f1 (ppm)

2-([1,1'-Biphenyl]-4-yl)-3,3-diethylazetidine-1-sulfonyl fluoride (3j)

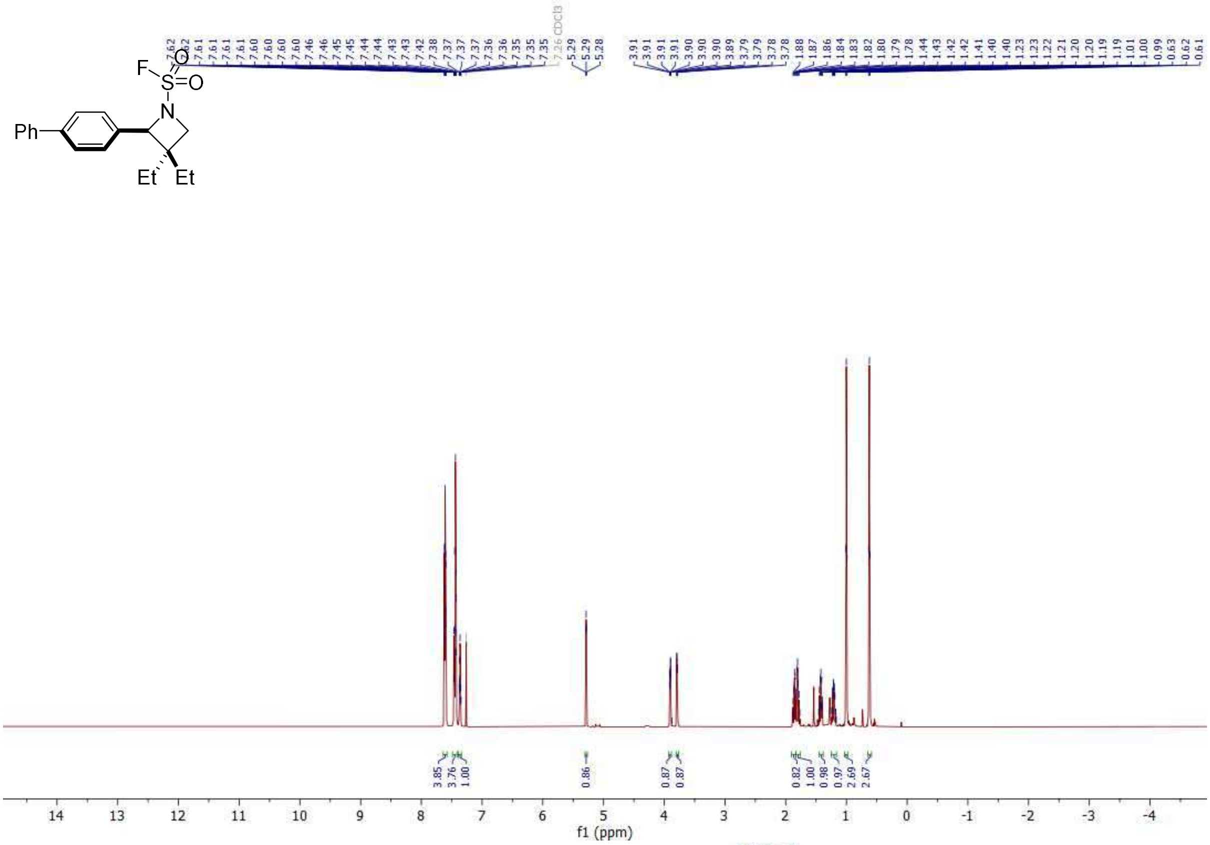

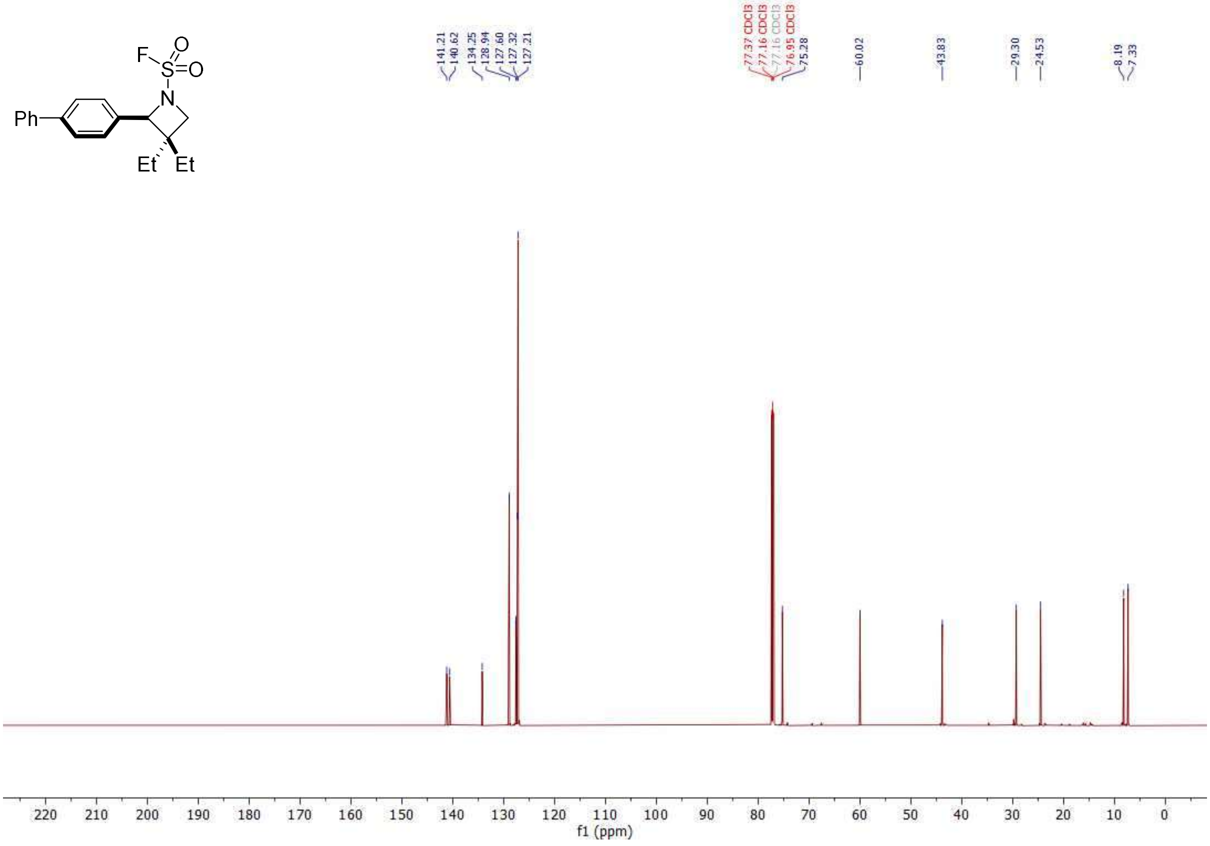

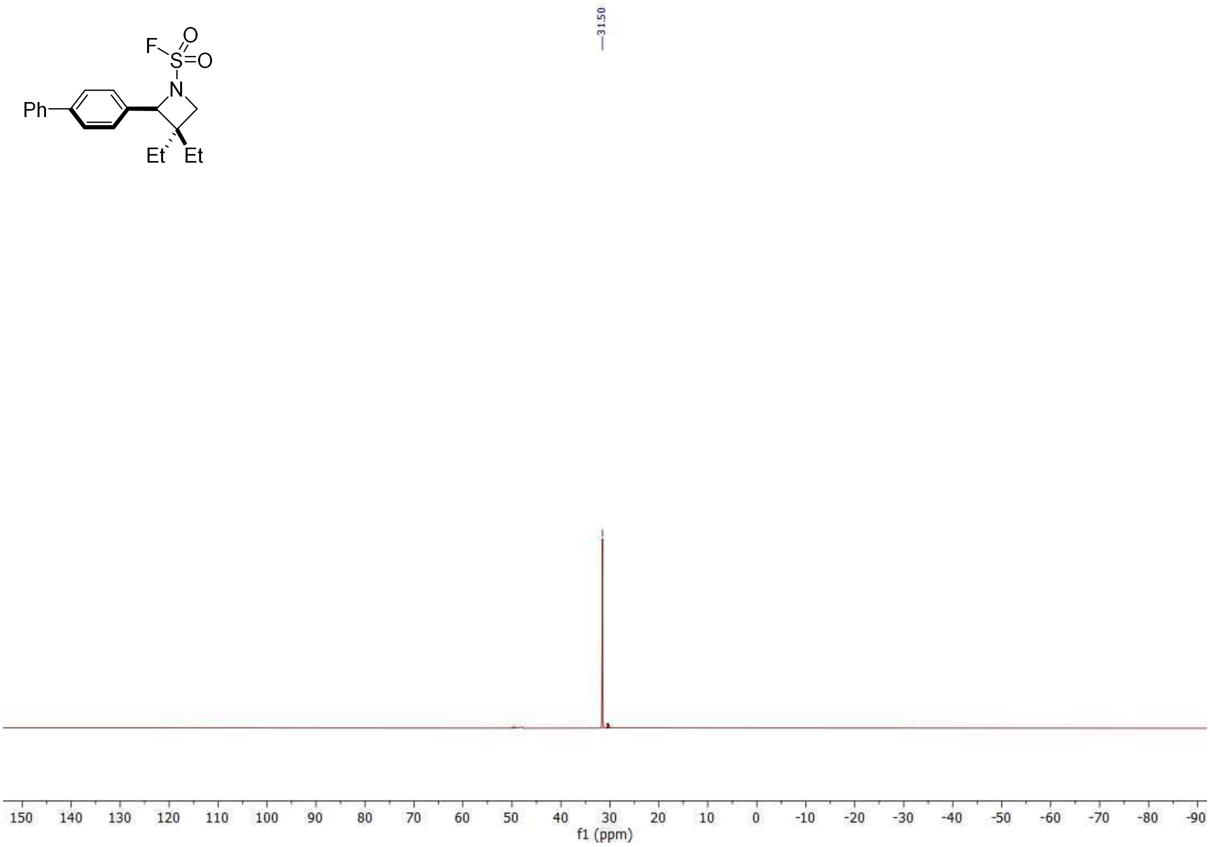
F
S
O
O
N
Ph
Et
Et
31.50
150 140 130 120 110 100 90 80 70 60 50 40 30 20 10 0 -10 -20 -30 -40 -50 -60 -70 -80 -90
f1 (ppm)

3,3-Diethyl-2-(o-tolyl)azetidine-1-sulfonyl fluoride (3k)

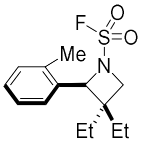

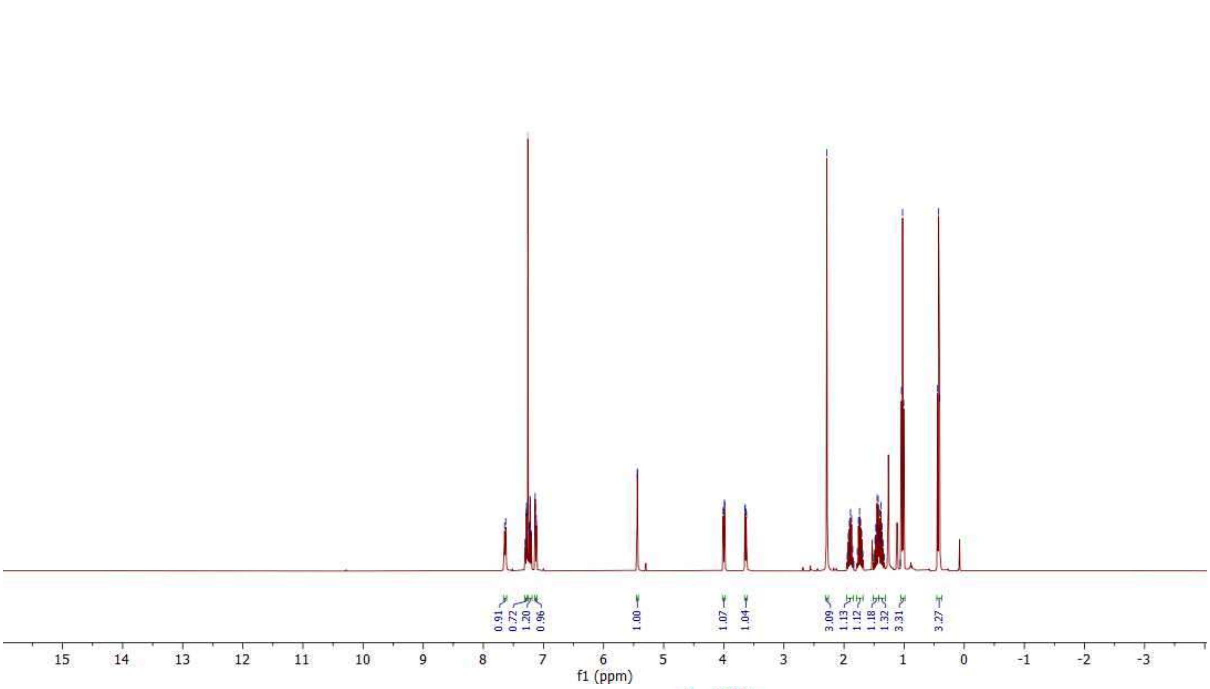

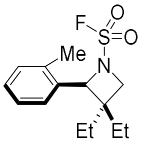

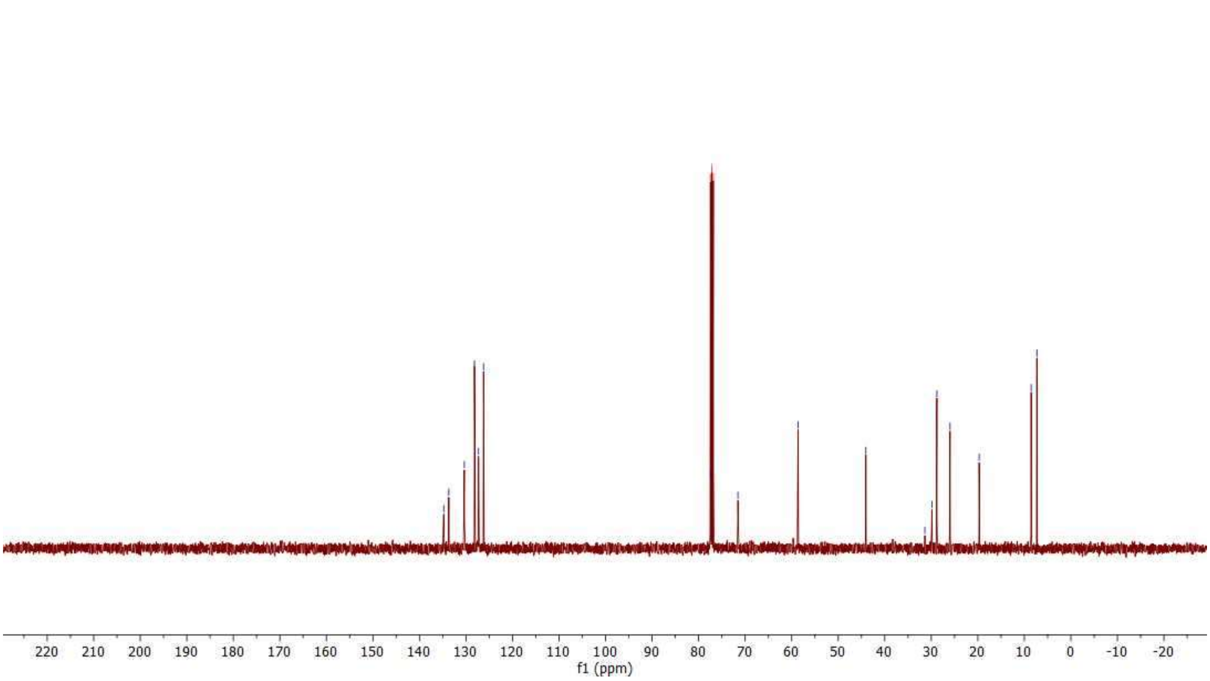

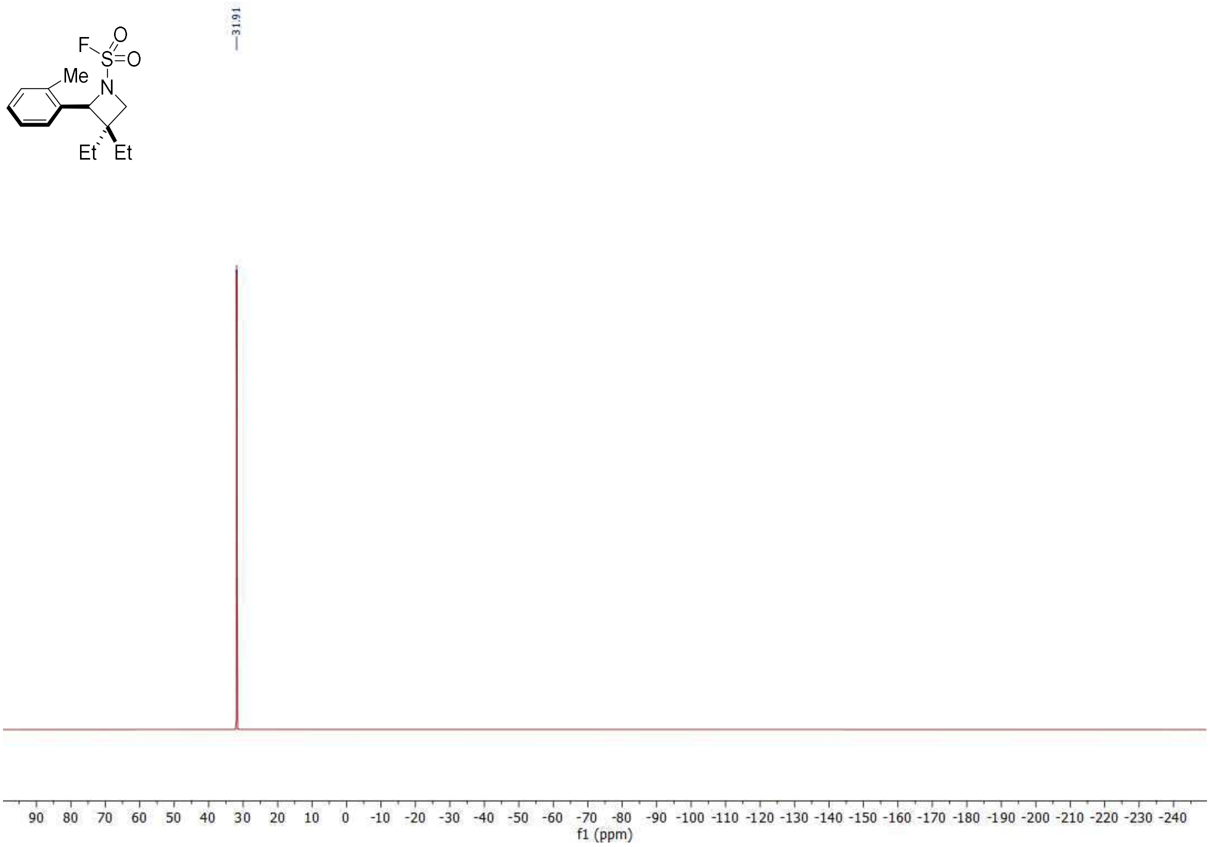

2-(4-(1,3-Dioxoisoindolin-2-yl)phenyl)-3,3-diethylazetidine-1-sulfonyl fluoride (3l)

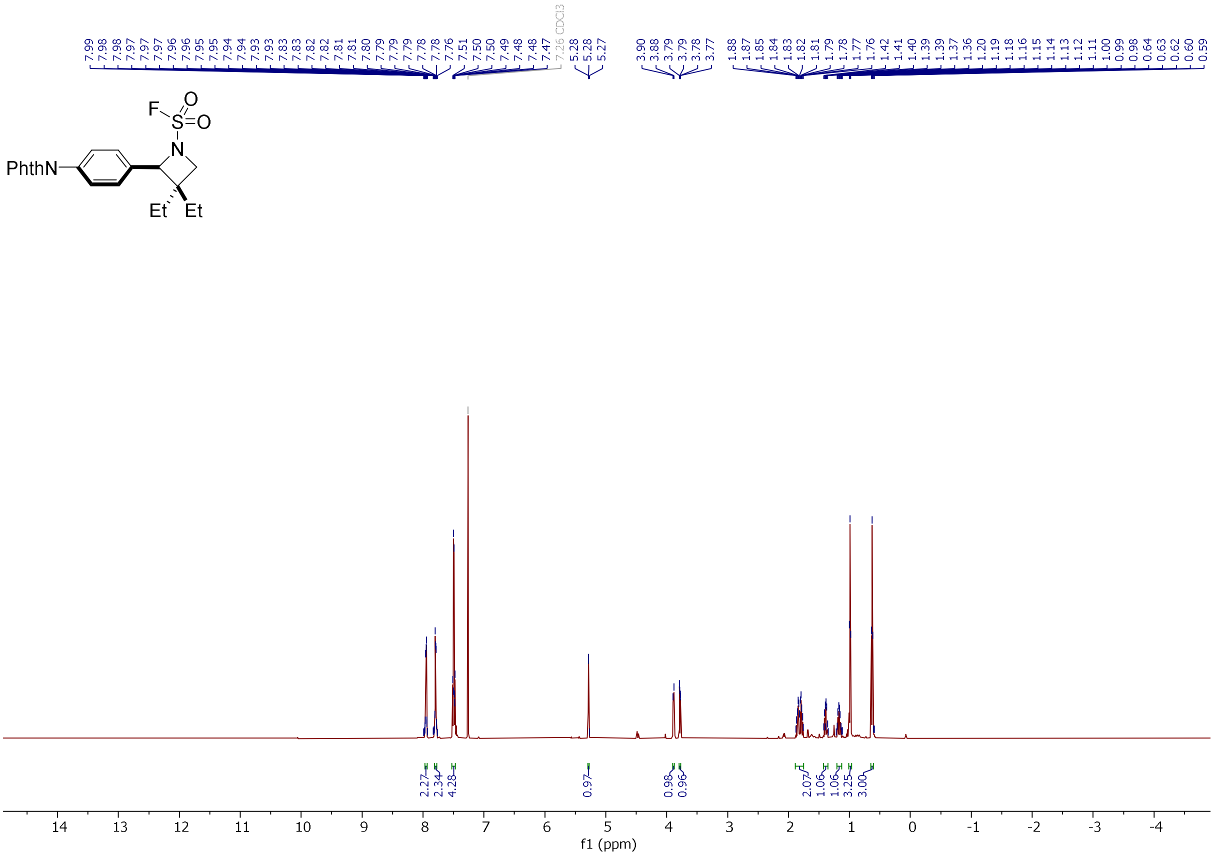

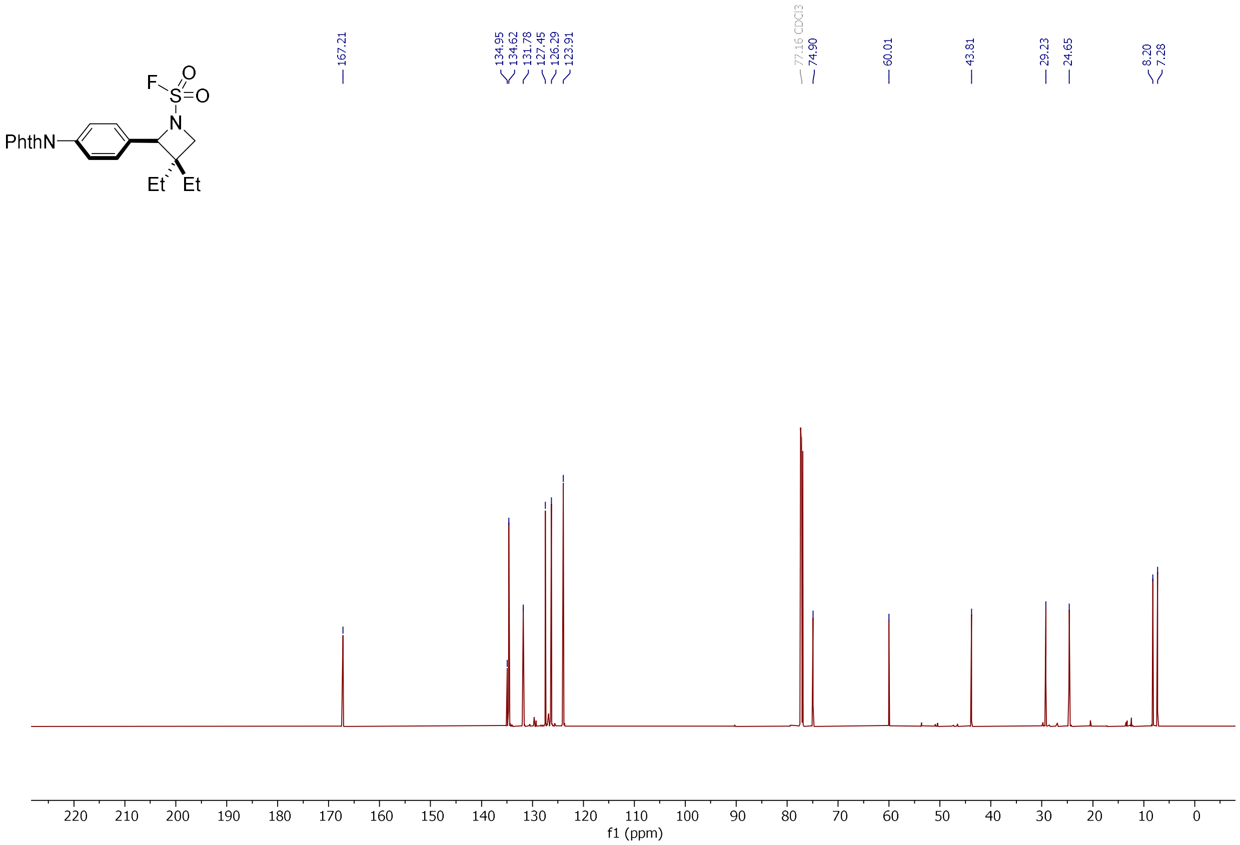
PhthN
F
S
O
O
N
Et
Et
167.21
134.95
134.62
131.78
127.45
126.29
123.91
77.16 CDCl3
74.90
60.01
43.81
29.23
24.65
8.20
7.28
f1 (ppm)

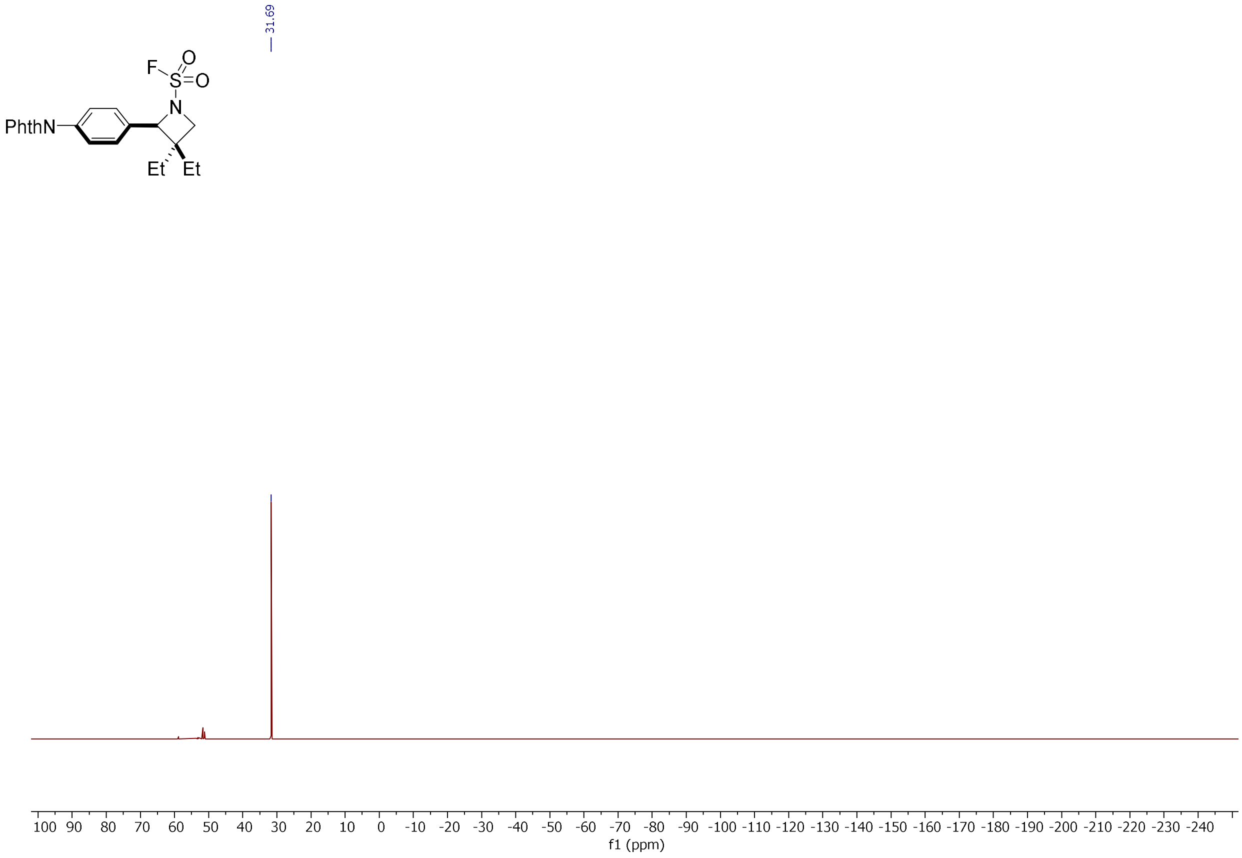
PhthN
F
S
O
O
N
Et
Et
31.69
f1 (ppm)

4-(3,3-Diethyl-1-(fluorosulfonyl)azetidin-2-yl)phenyl pivalate (3m)

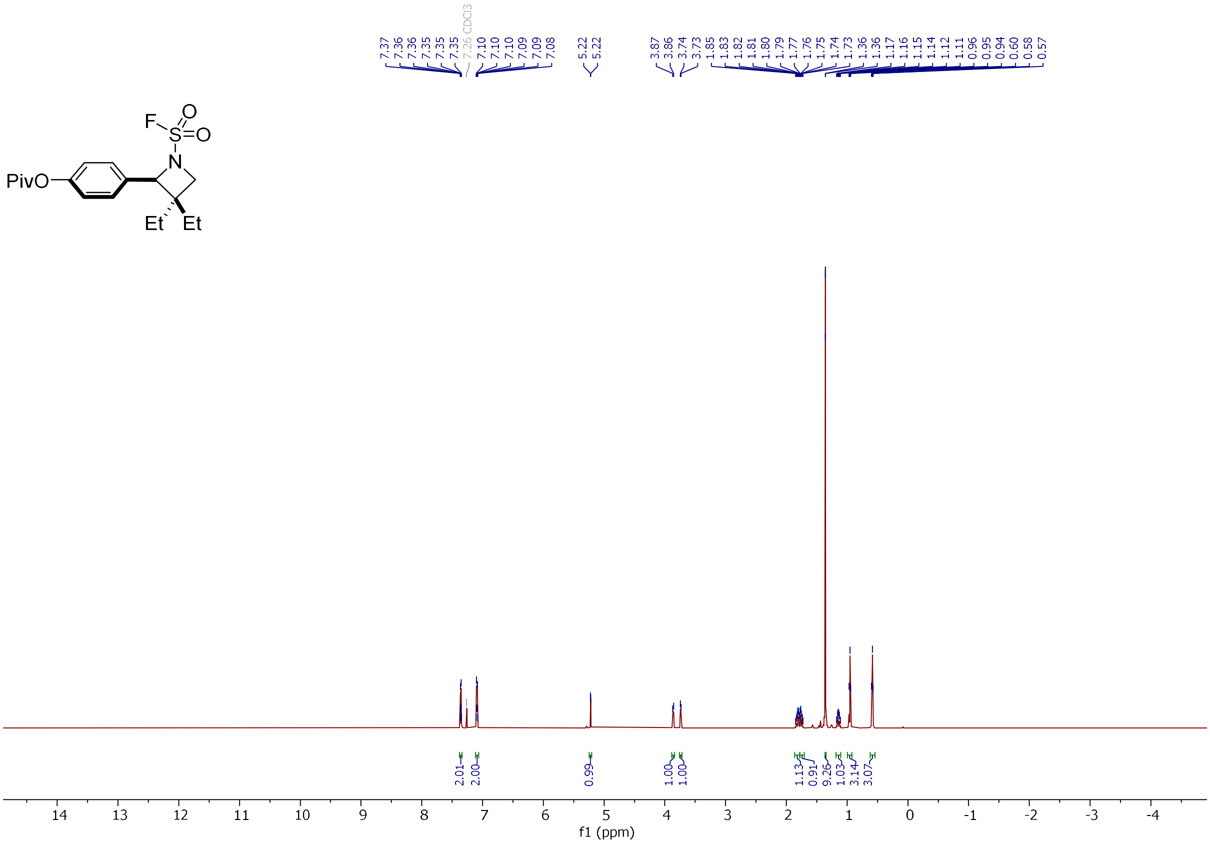

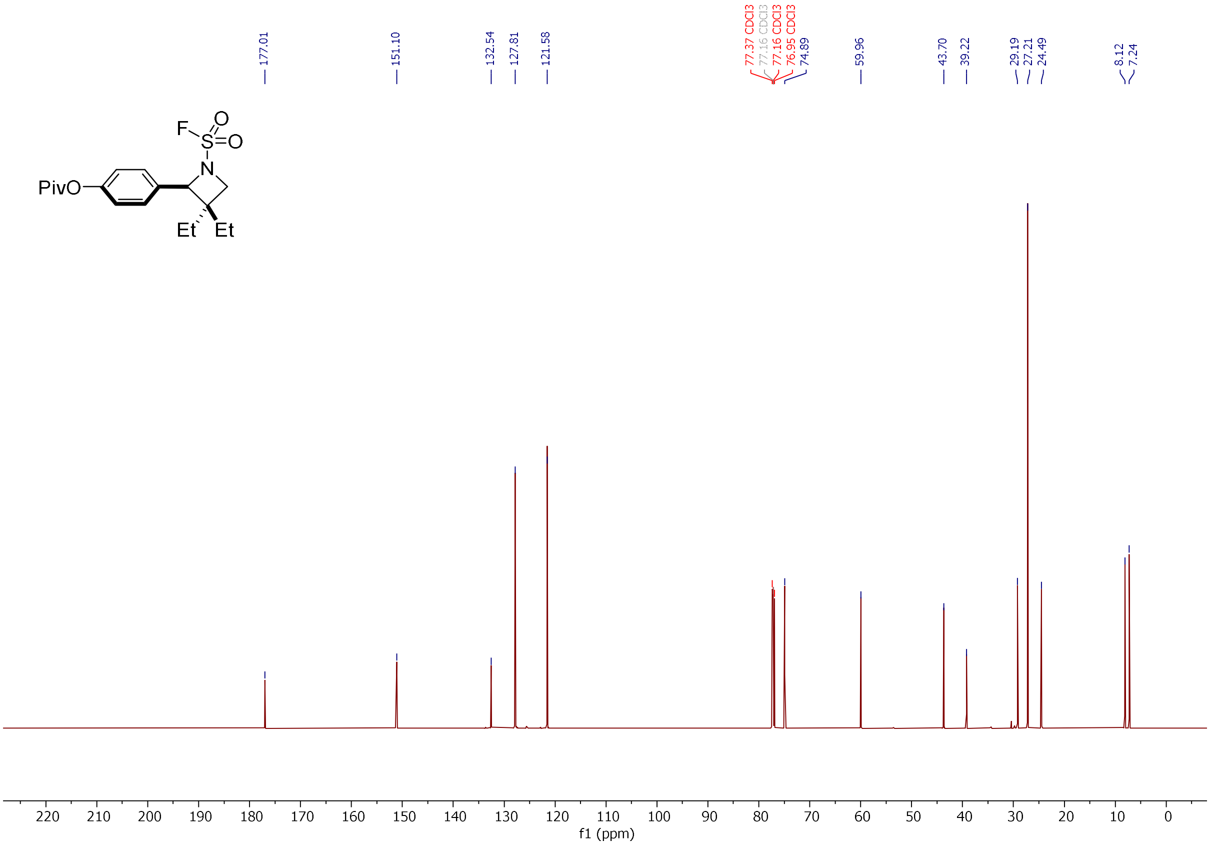

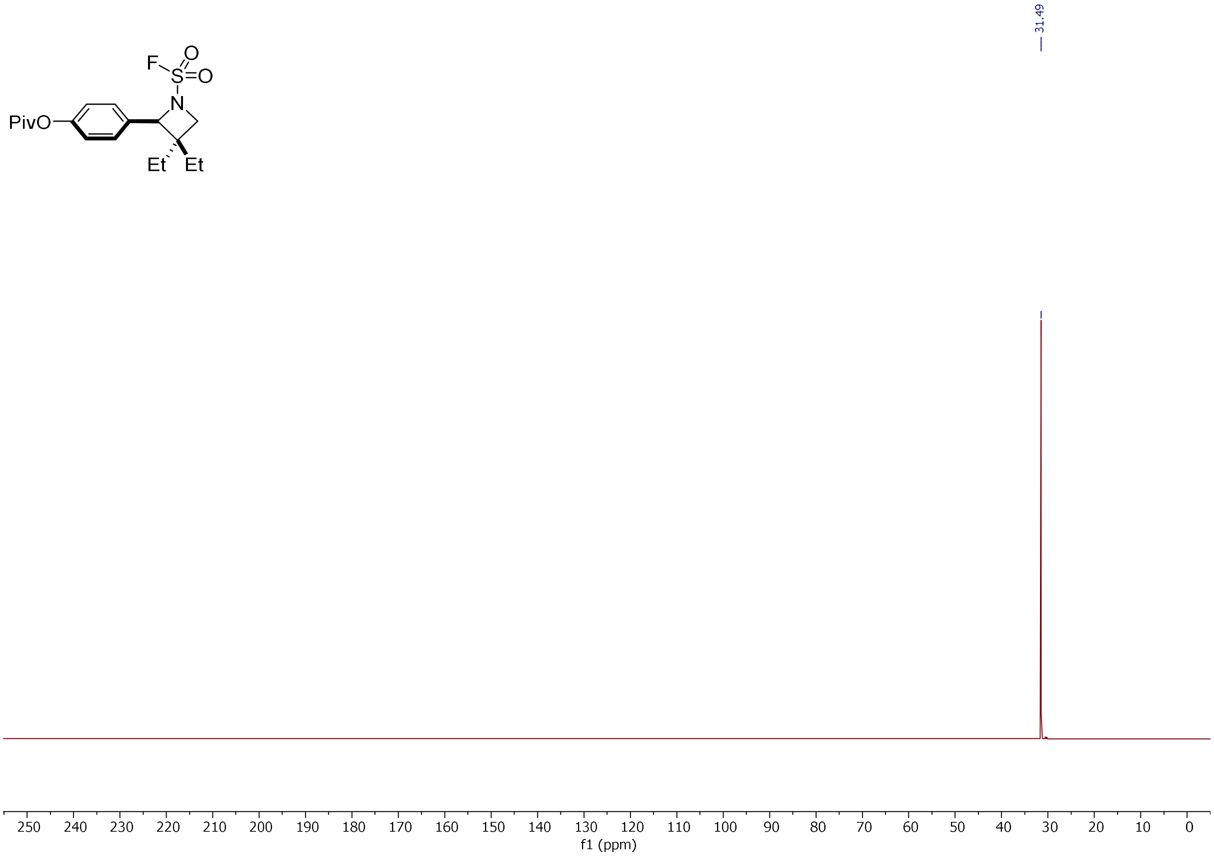

Methyl 3-(3,3-diethyl-1-(fluorosulfonyl)azetidin-2-yl)benzoate (3n)

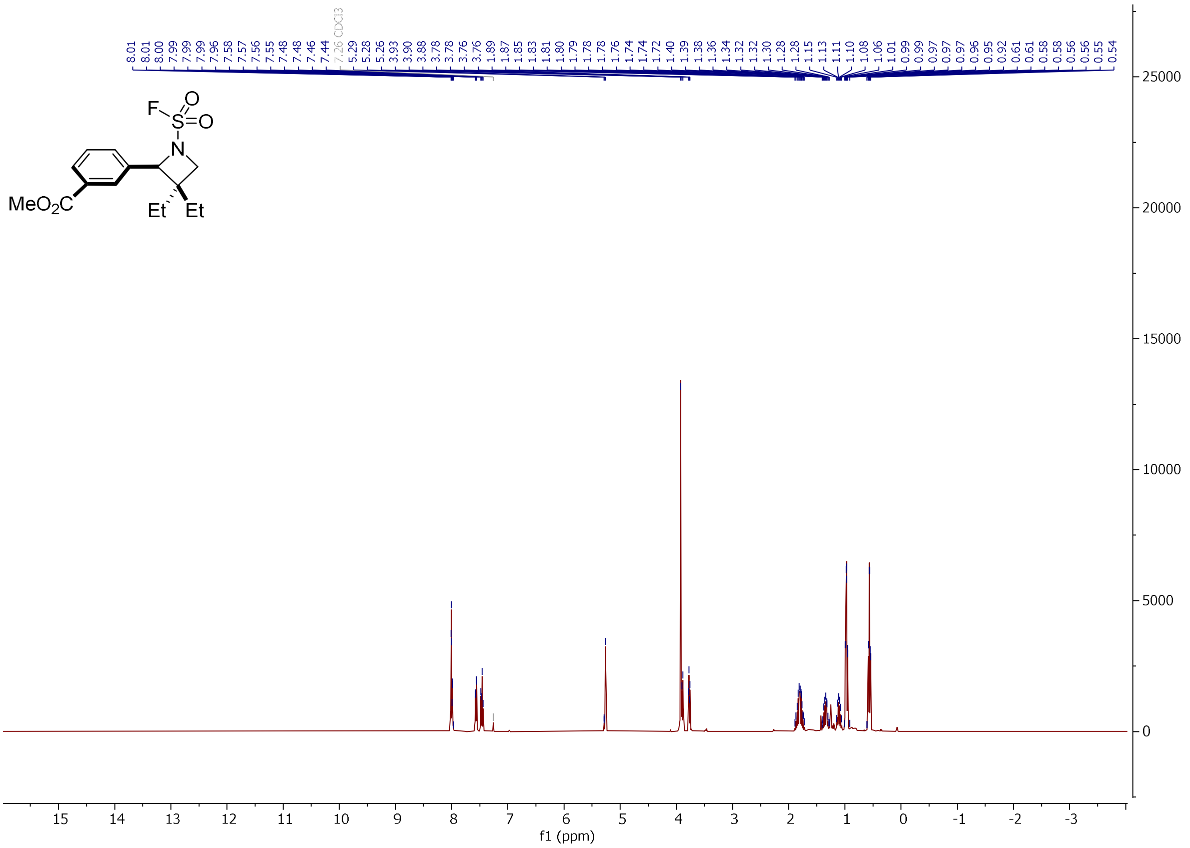

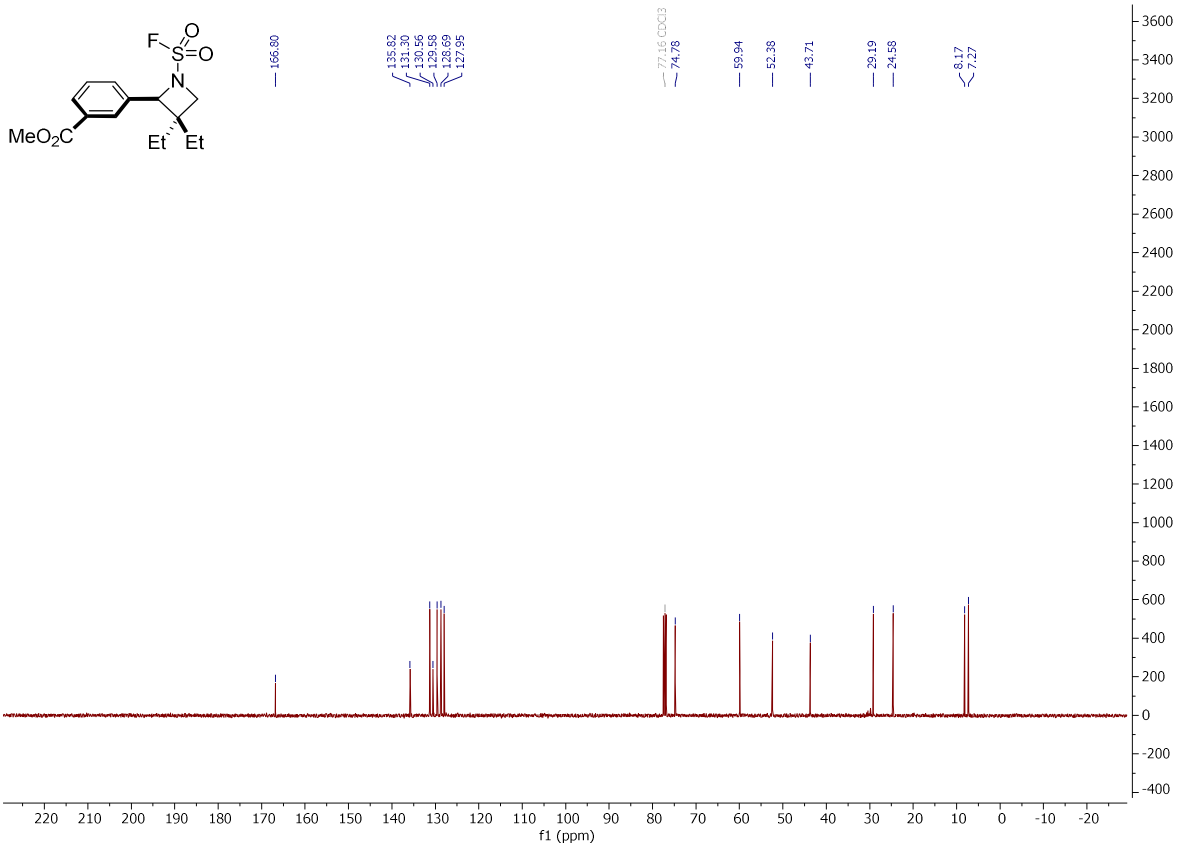

MeO2C
Et
Et
F
S
O
O
N
166.80
135.82
131.30
130.56
129.58
128.69
127.95
77.16 CDCl3
74.78
59.94
52.38
43.71
29.19
24.58
8.17
7.27
f1 (ppm)

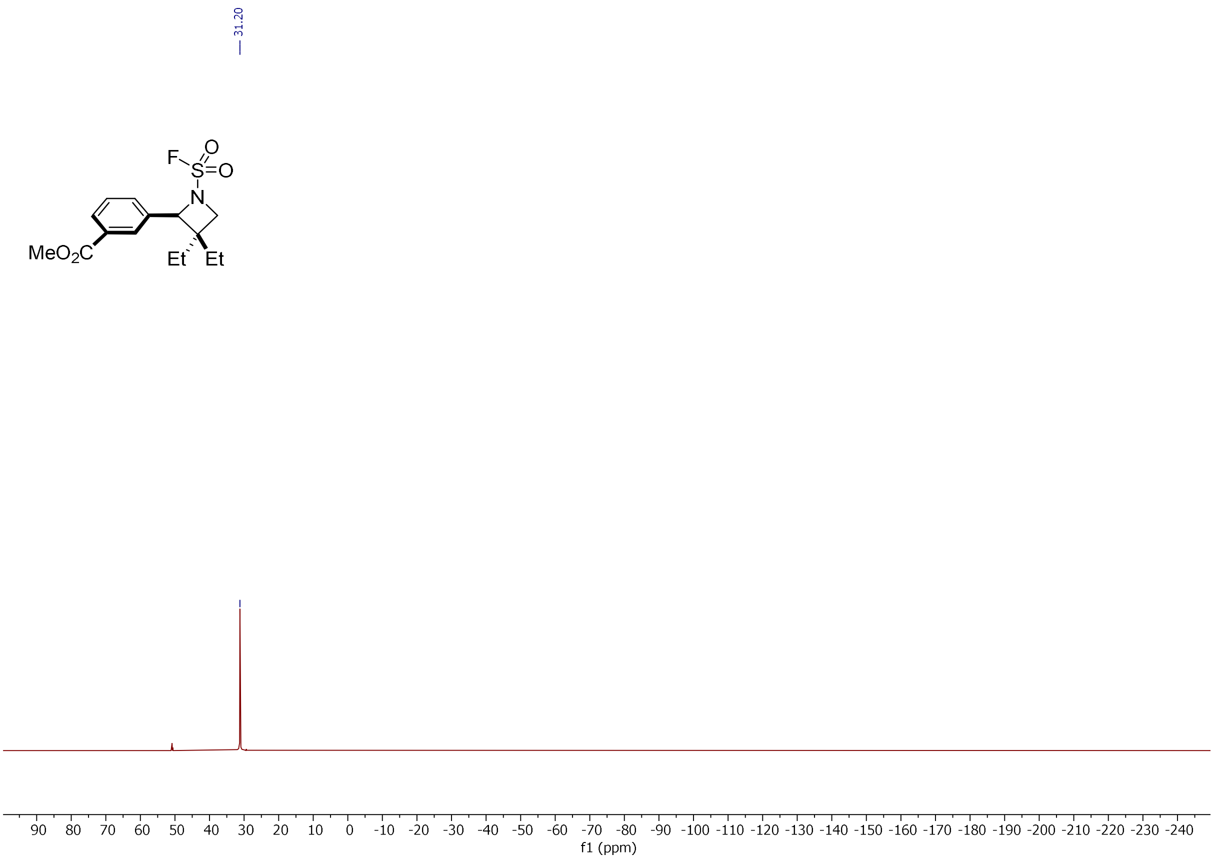

31.20
MeO2C
Et
Et
F
S
O
O
N
f1 (ppm)

3,3-Diethyl-2-(4-(trifluoromethyl)phenyl)azetidine-1-sulfonyl fluoride (3o)

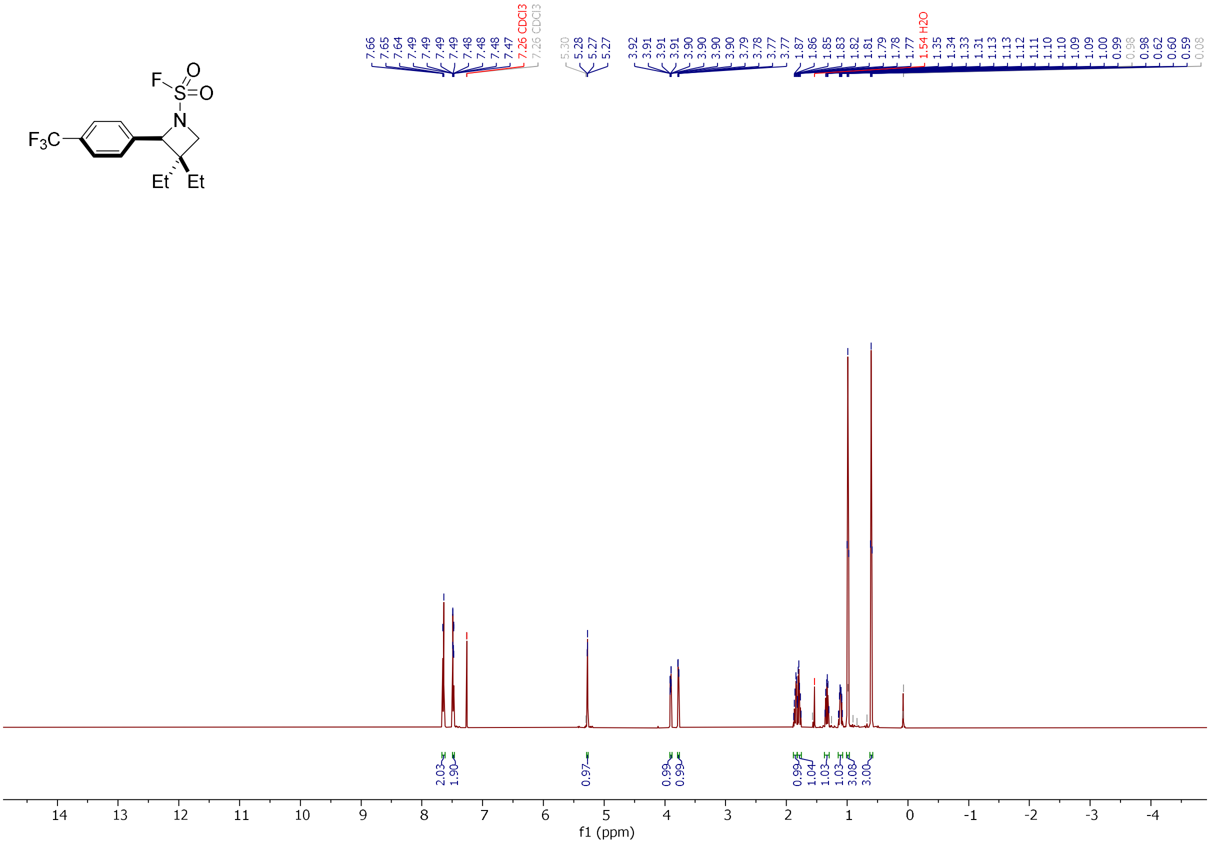

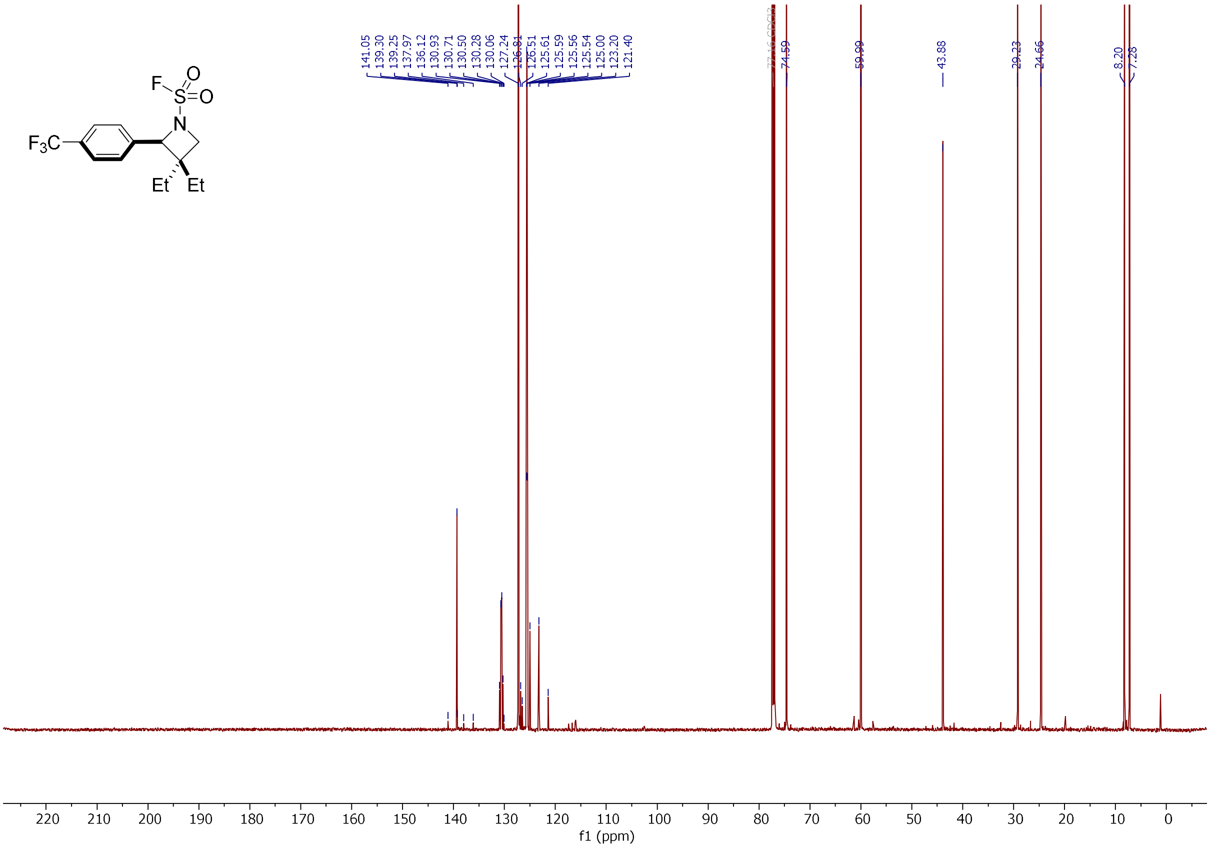

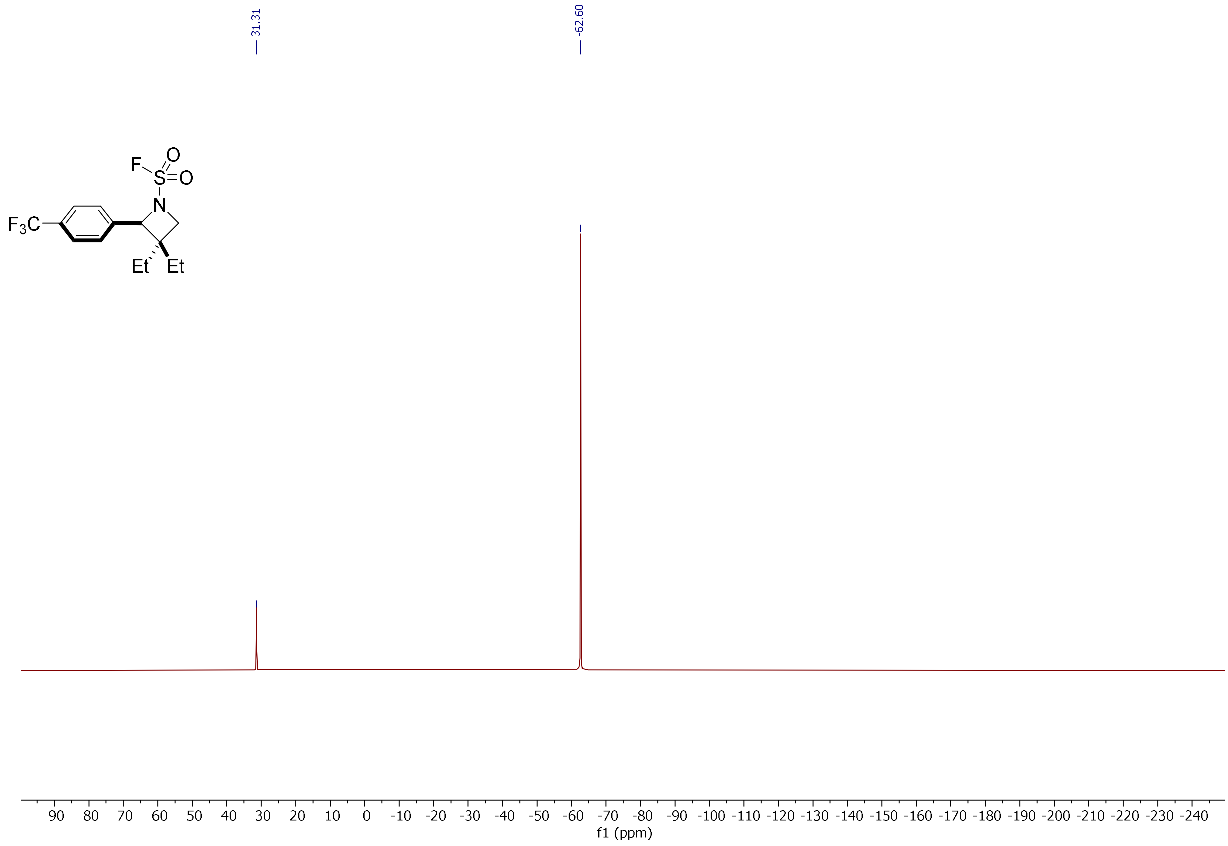
31.31
-62.60
F
O
S
O
N
F3C
Et
Et
90 80 70 60 50 40 30 20 10 0 -10 -20 -30 -40 -50 -60 -70 -80 -90 -100 -110 -120 -130 -140 -150 -160 -170 -180 -190 -200 -210 -220 -230 -240
f1 (ppm)

2-(2,2-Difluorobenzo[d][1,3]dioxol-5-yl)-3,3-diethylazetidine-1-sulfonyl fluoride (3p)

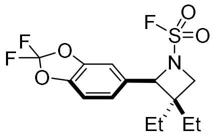

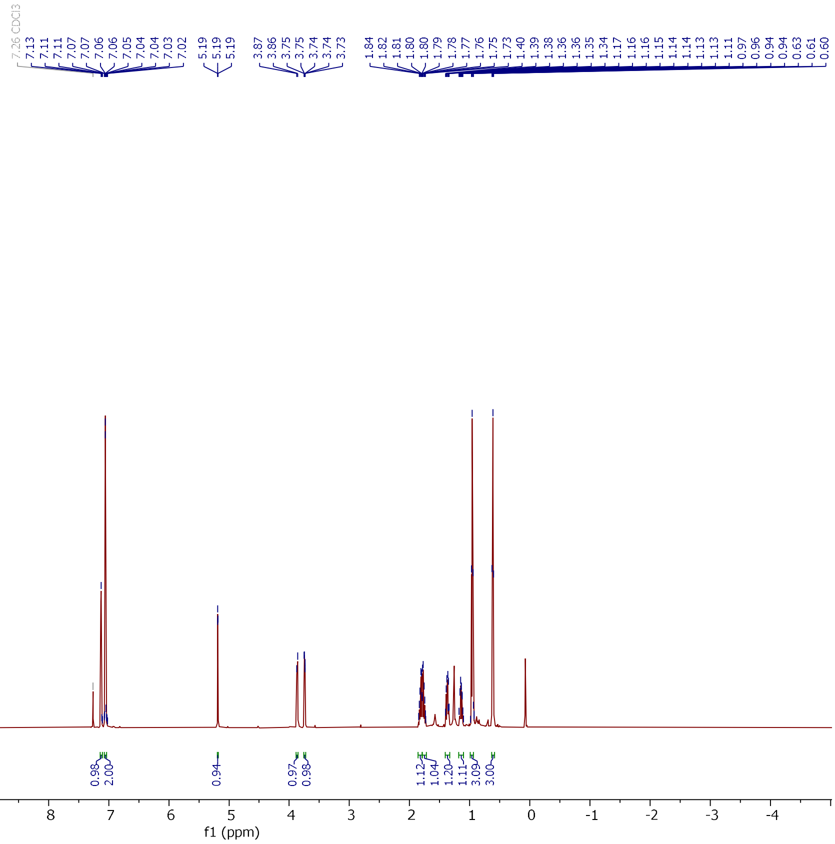

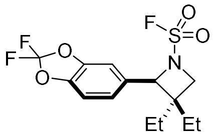

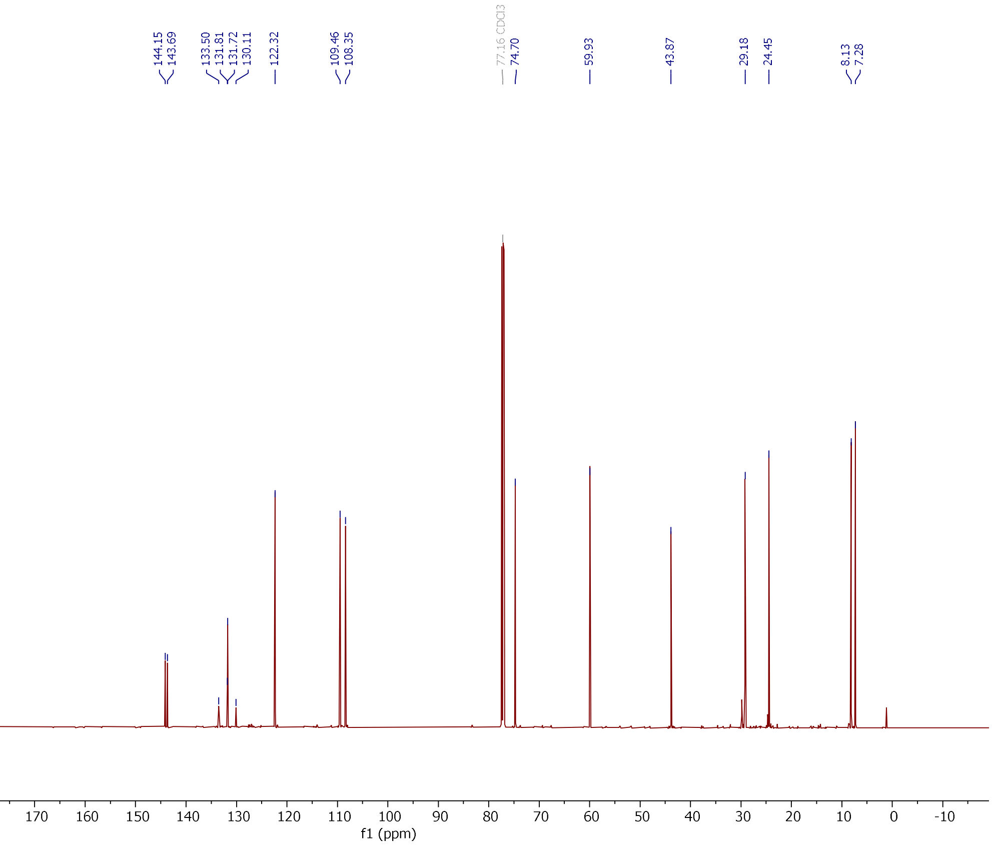

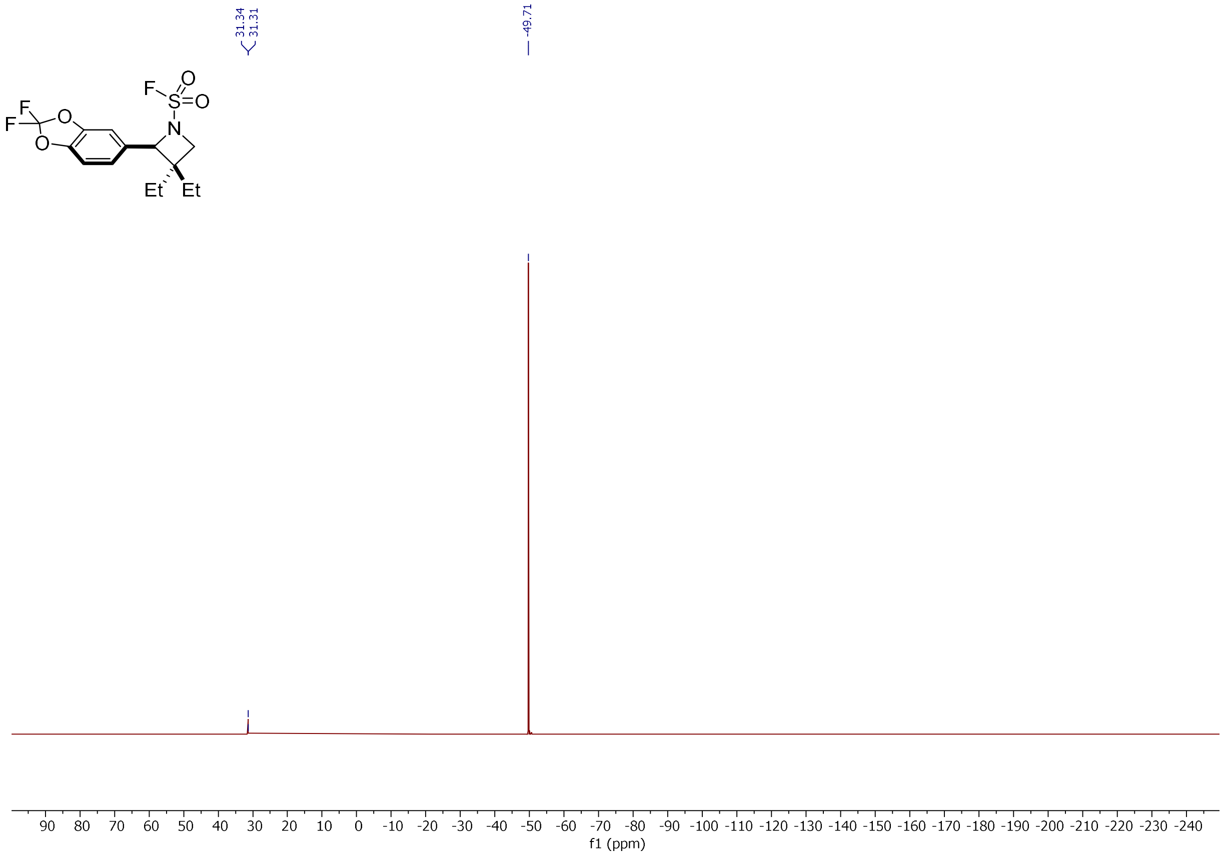

2-(4-(2-Bromoethoxy)phenyl)-3,3-diethylazetidine-1-sulfonyl fluoride (3q)

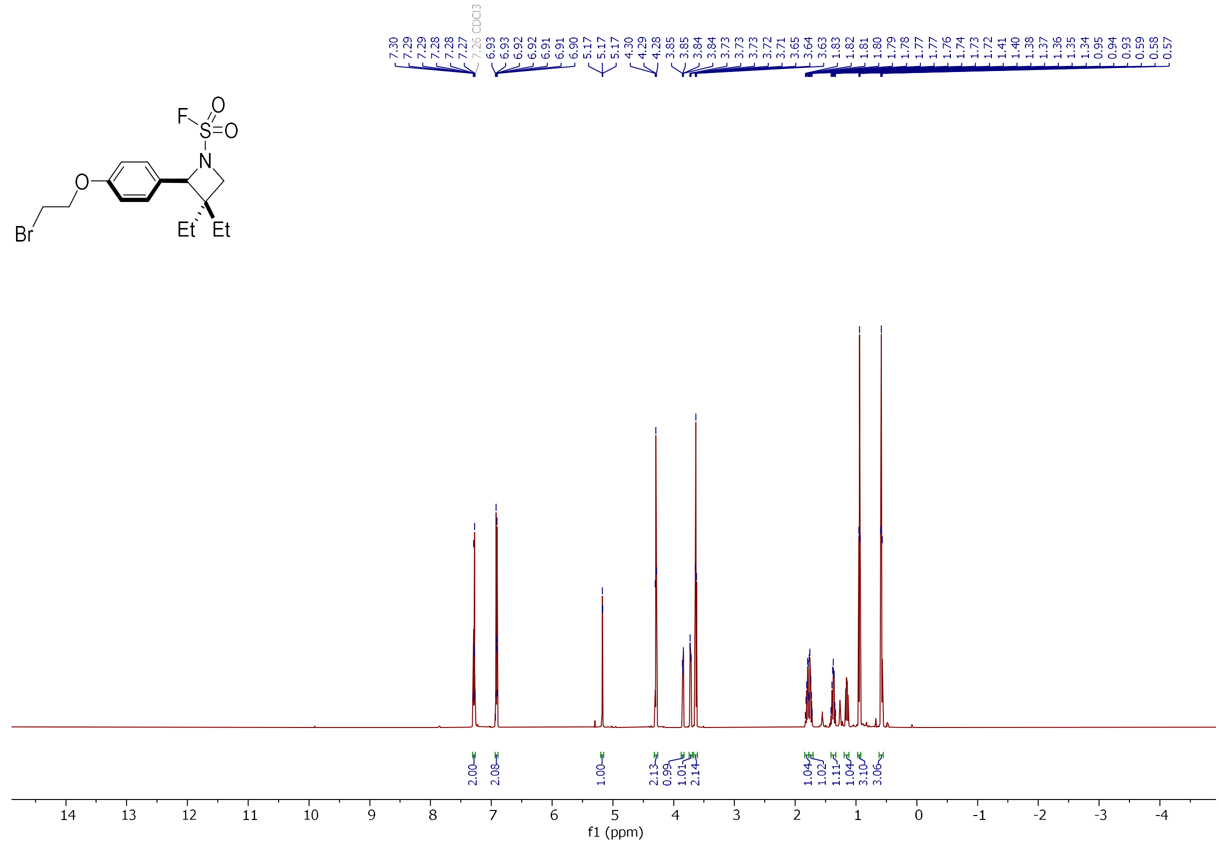

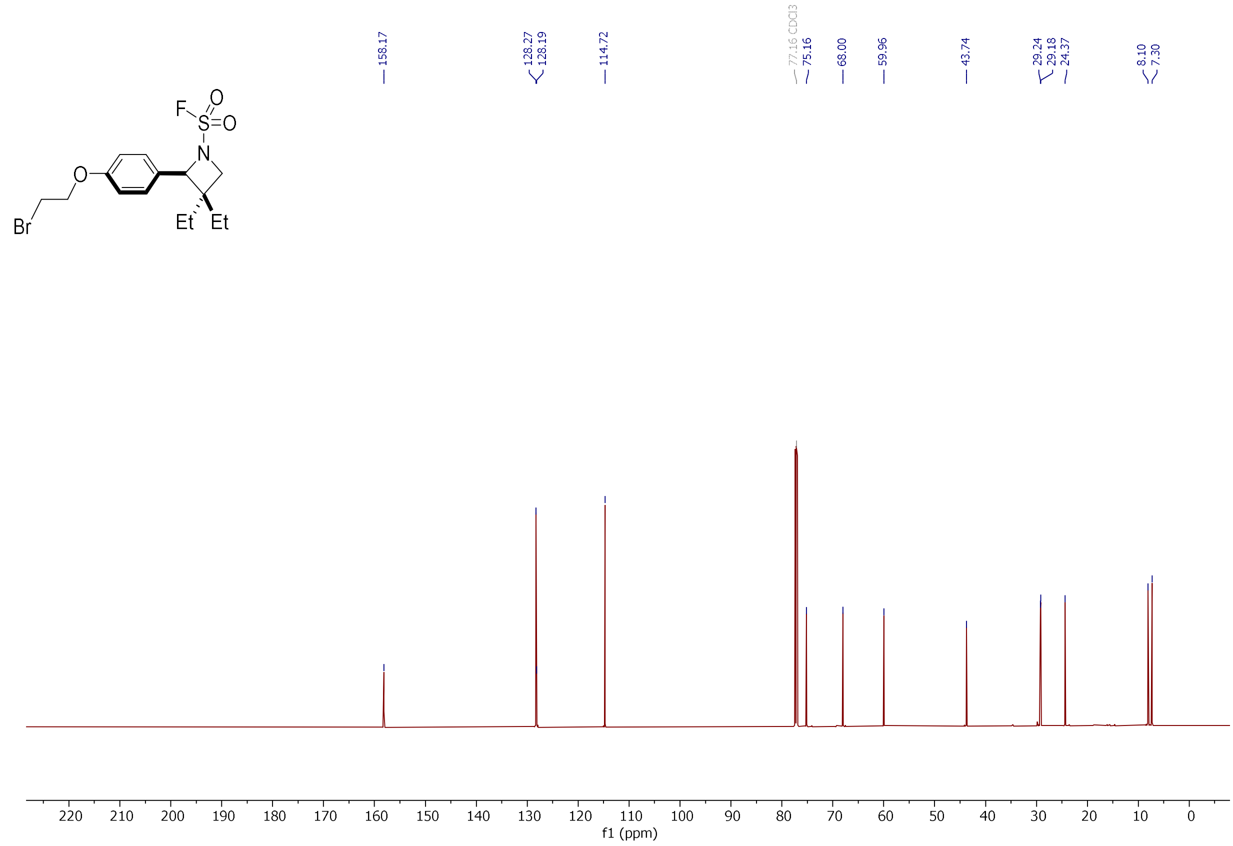
158.17
128.27
128.19
114.72
77.16 CDCl3
75.16
68.00
59.96
43.74
29.24
29.18
24.37
8.10
7.30
F
S
O
O
N
O
Br
Et
Et
220 210 200 190 180 170 160 150 140 130 120 110 100 90 80 70 60 50 40 30 20 10 0
f1 (ppm)

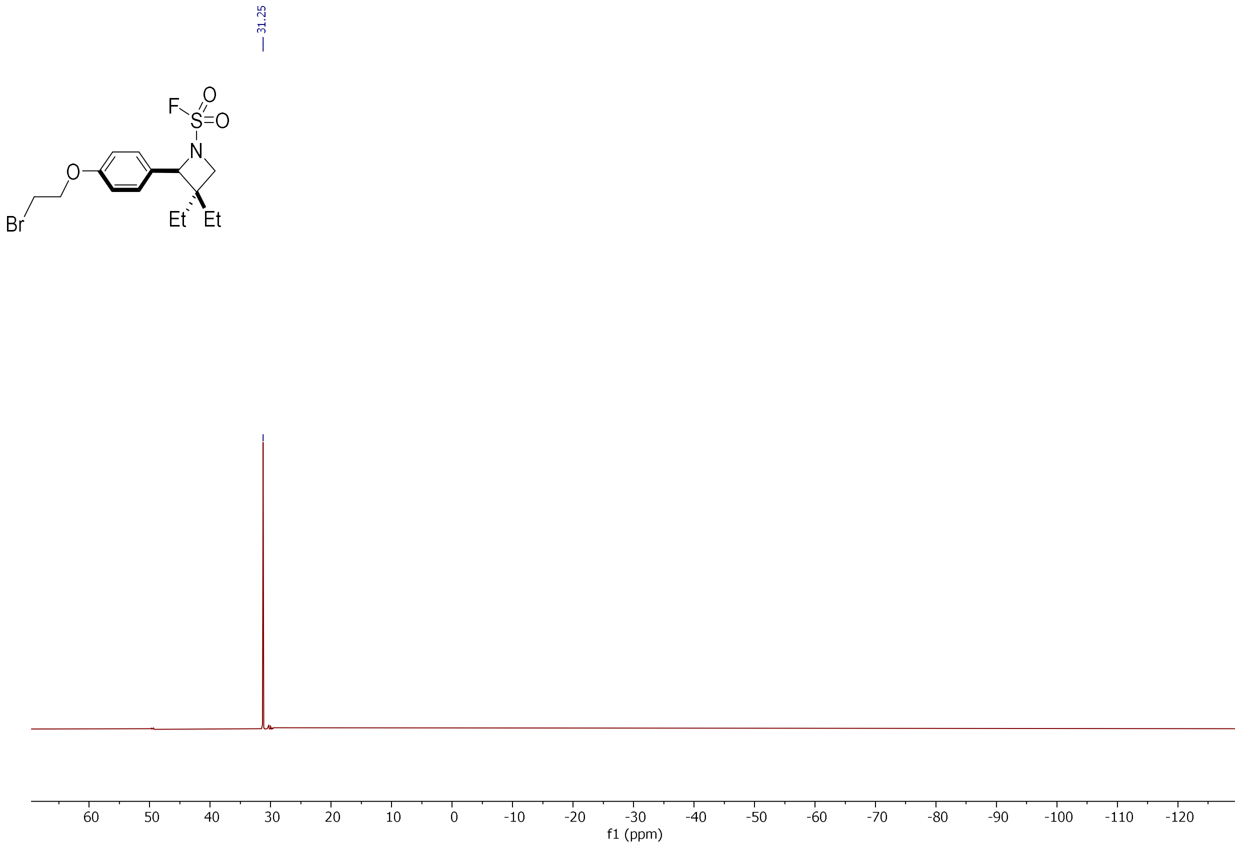
31.25
F
S
O
O
N
O
Br
Et
Et
60 50 40 30 20 10 0 -10 -20 -30 -40 -50 -60 -70 -80 -90 -100 -110 -120
f1 (ppm)

3,3-Diethyl-2-(2-methoxy-5-(4,4,5,5-tetraethyl-1,3,2-dioxaborolan-2-yl)phenyl)azetidine-1-sulfonyl fluoride (3r)

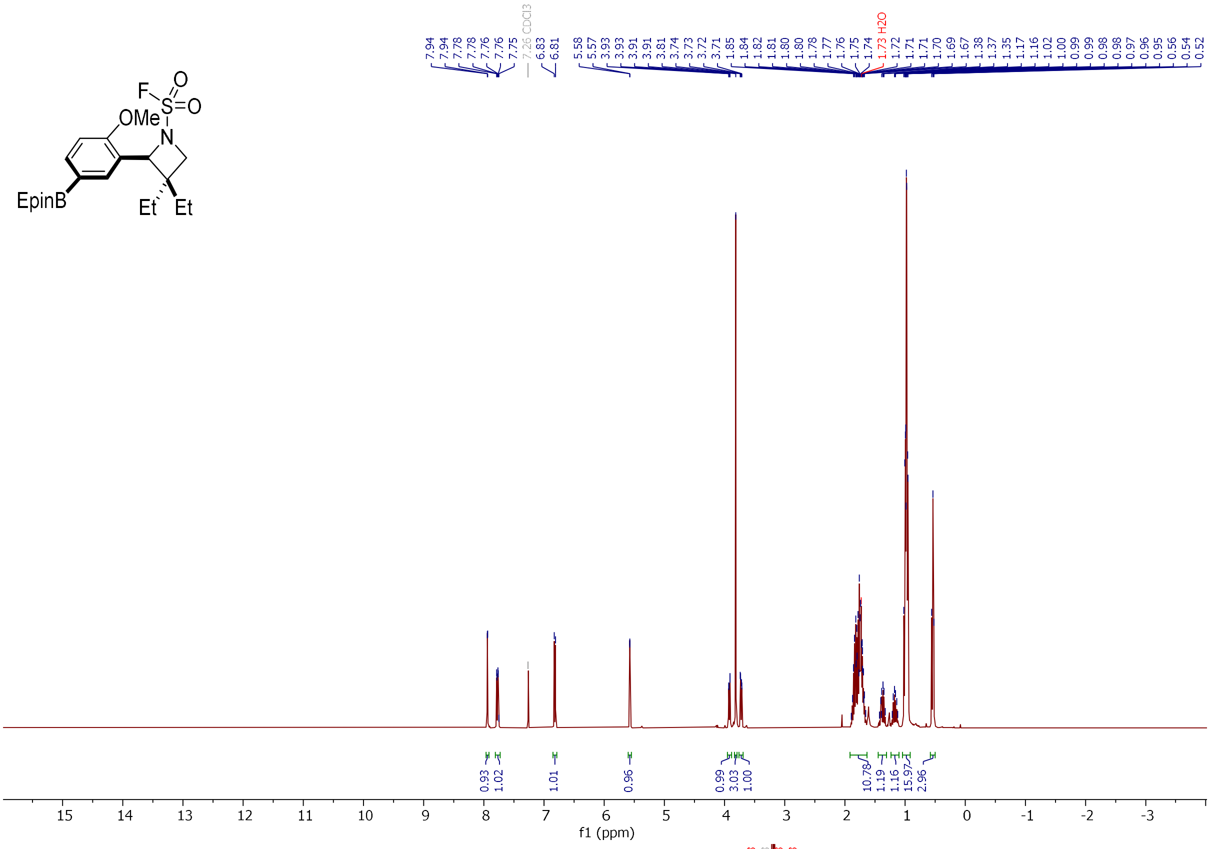

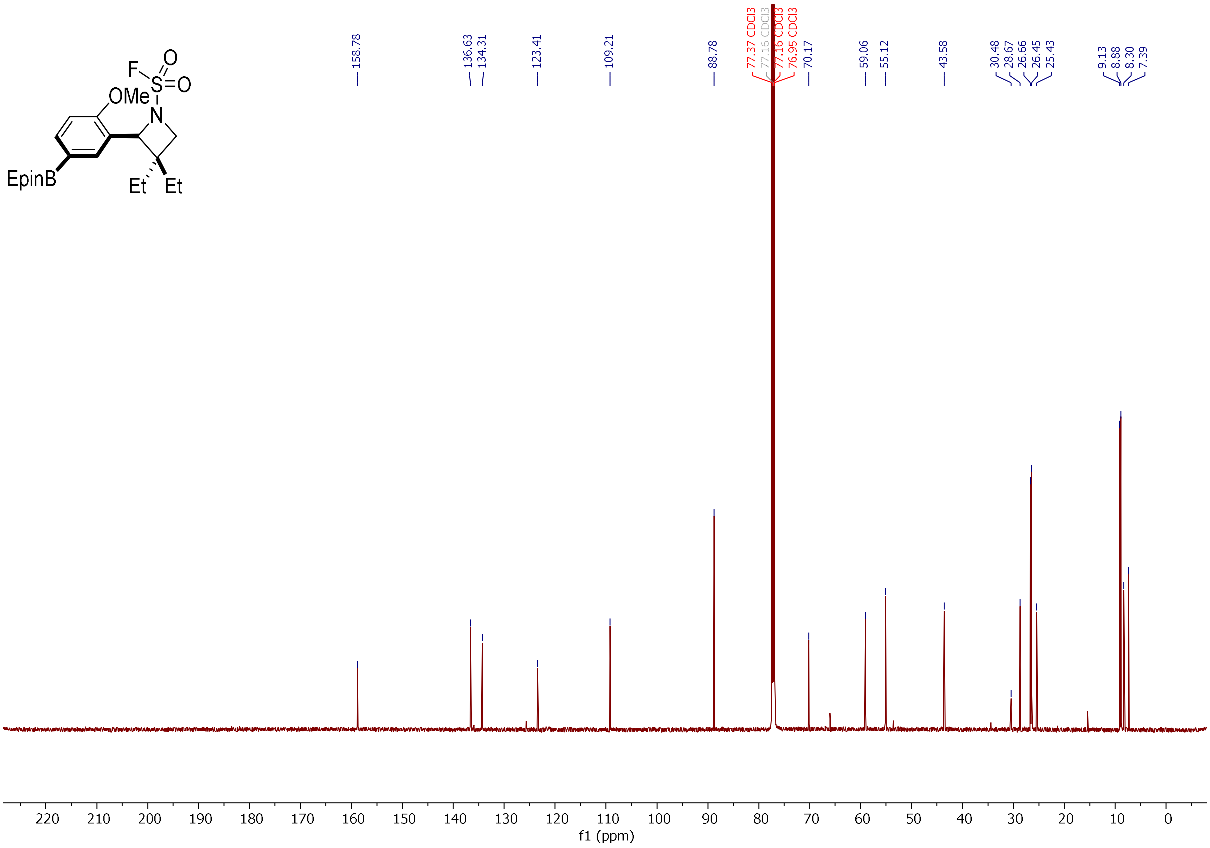

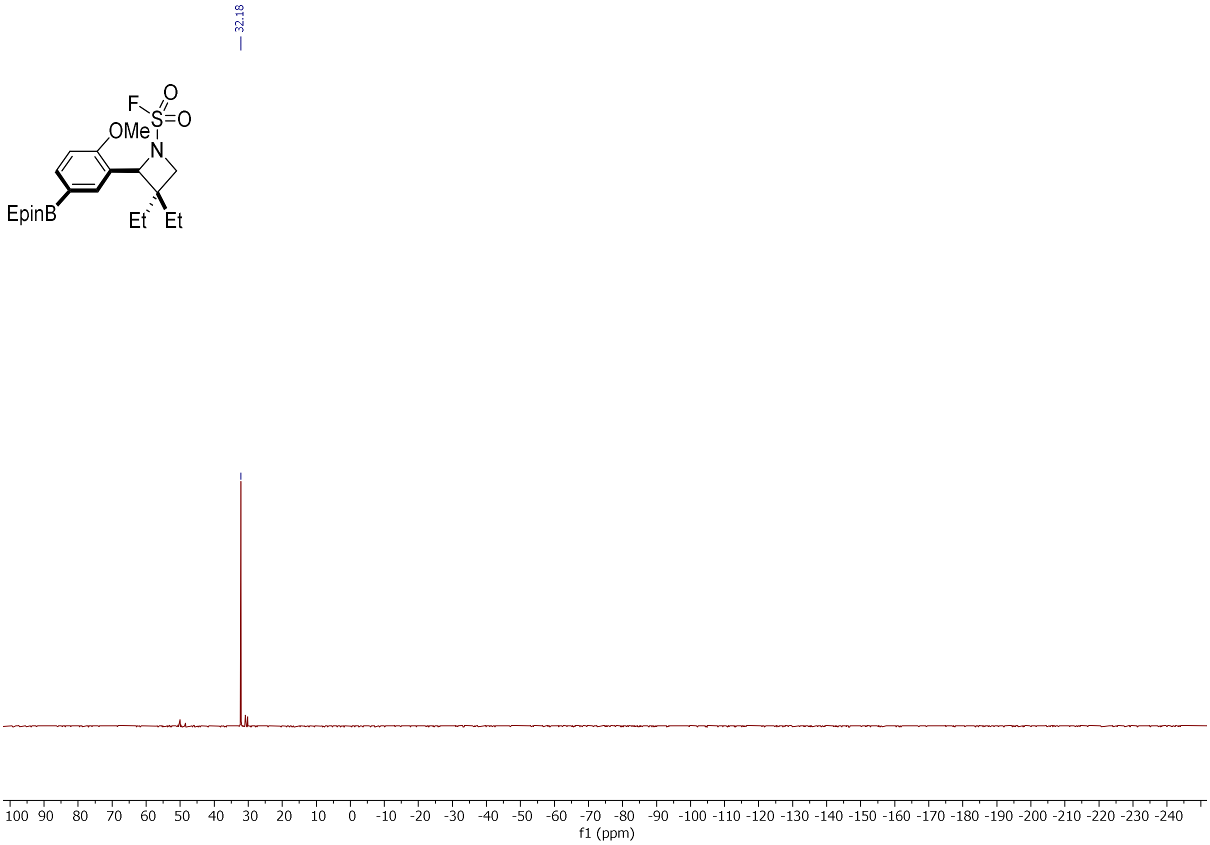

3,3-Diethyl-2-(3-hydroxy-4-methoxyphenyl)azetidine-1-sulfonyl fluoride (3s)

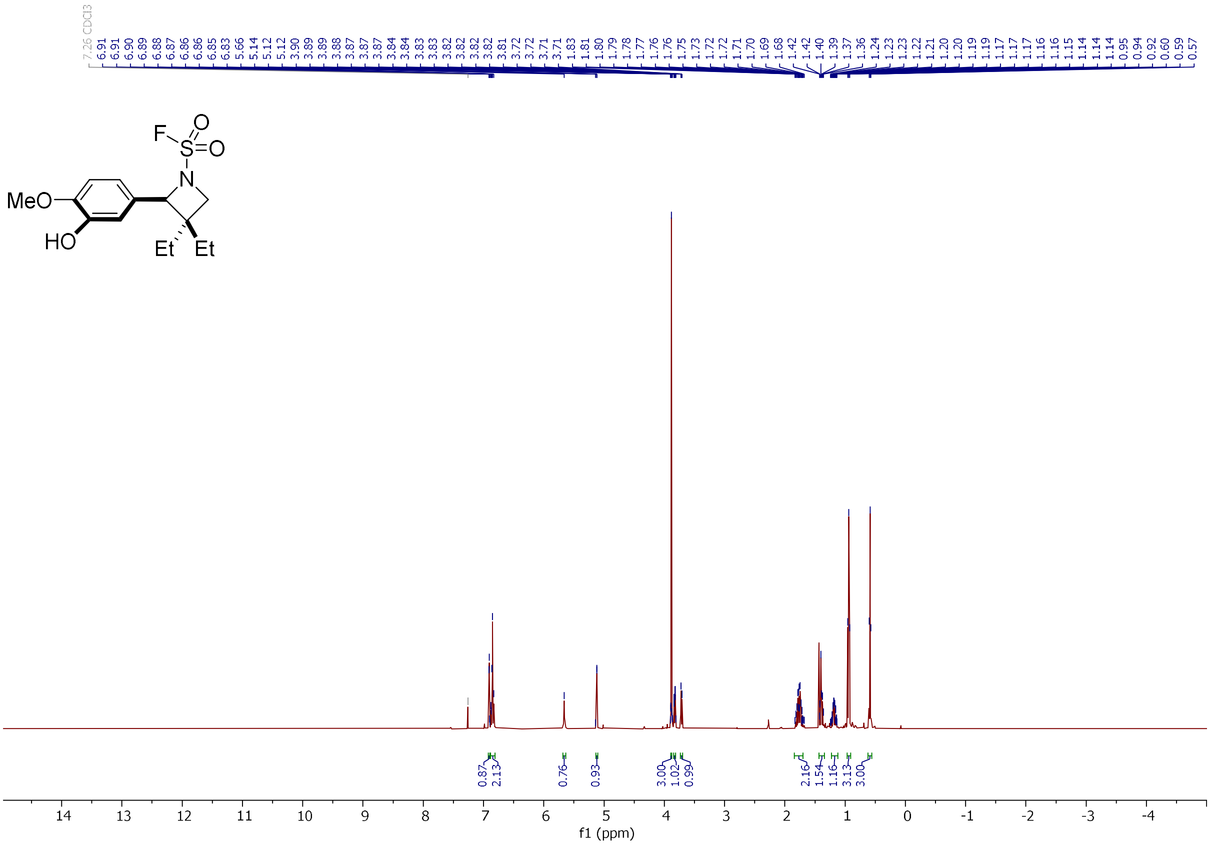

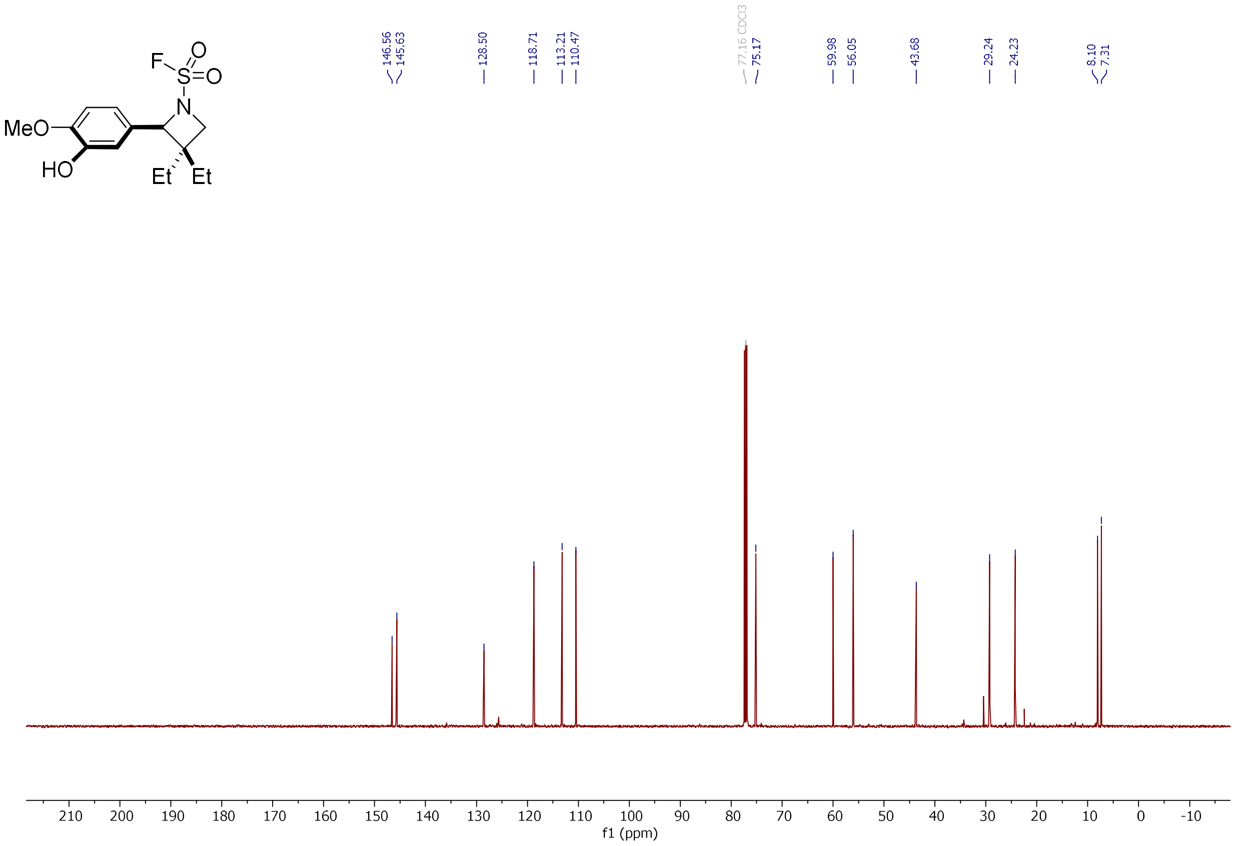
146.56
145.63
128.50
118.71
113.21
110.47
77.16 CDCl3
75.17
59.98
56.05
43.68
29.24
24.23
8.10
7.31
MeO
HO
F
S
O
N
Et
Et
210 200 190 180 170 160 150 140 130 120 110 100 90 80 70 60 50 40 30 20 10 0 -10
f1 (ppm)

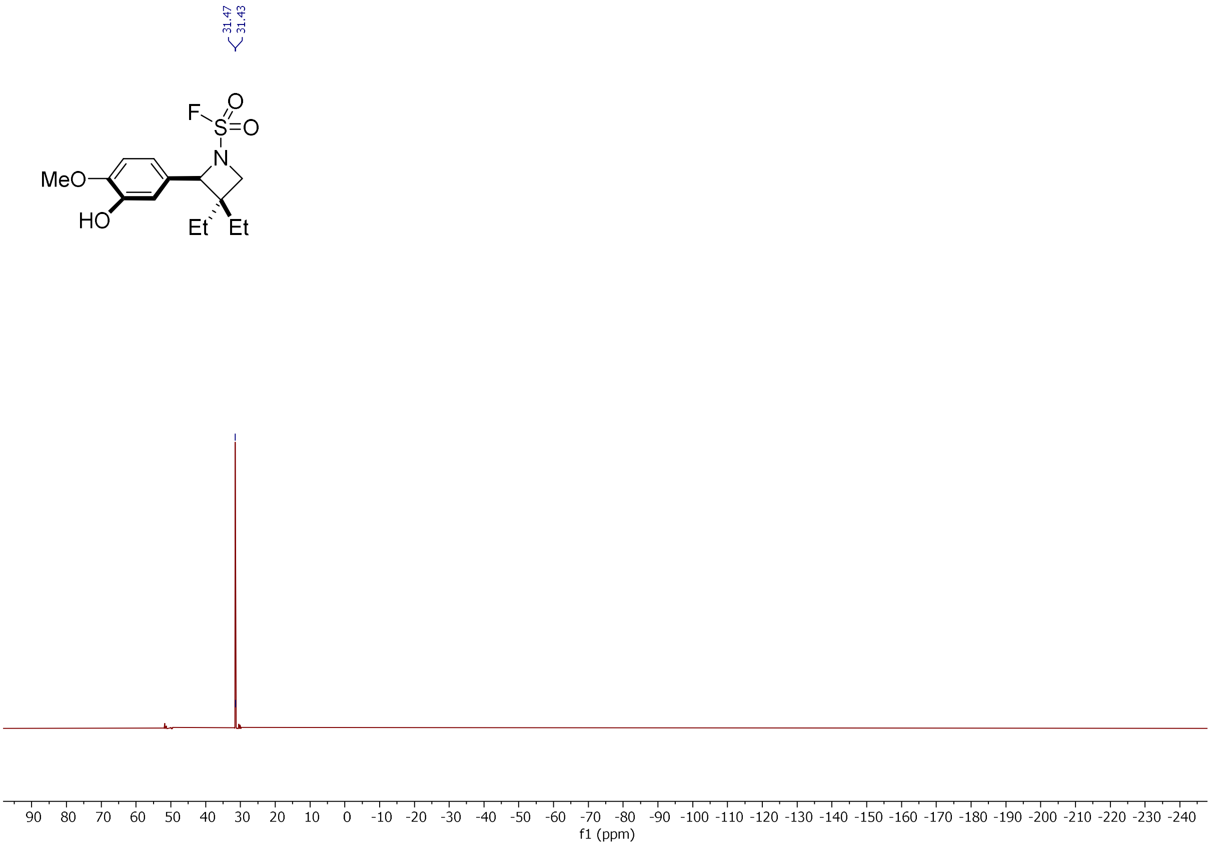
31.47
31.43
MeO
HO
F
S
O
N
Et
Et
90 80 70 60 50 40 30 20 10 0 -10 -20 -30 -40 -50 -60 -70 -80 -90 -100 -110 -120 -130 -140 -150 -160 -170 -180 -190 -200 -210 -220 -230 -240
f1 (ppm)

3,3-Diethyl-2-(6-methoxypyridin-3-yl)azetidine-1-sulfonyl fluoride (3w)

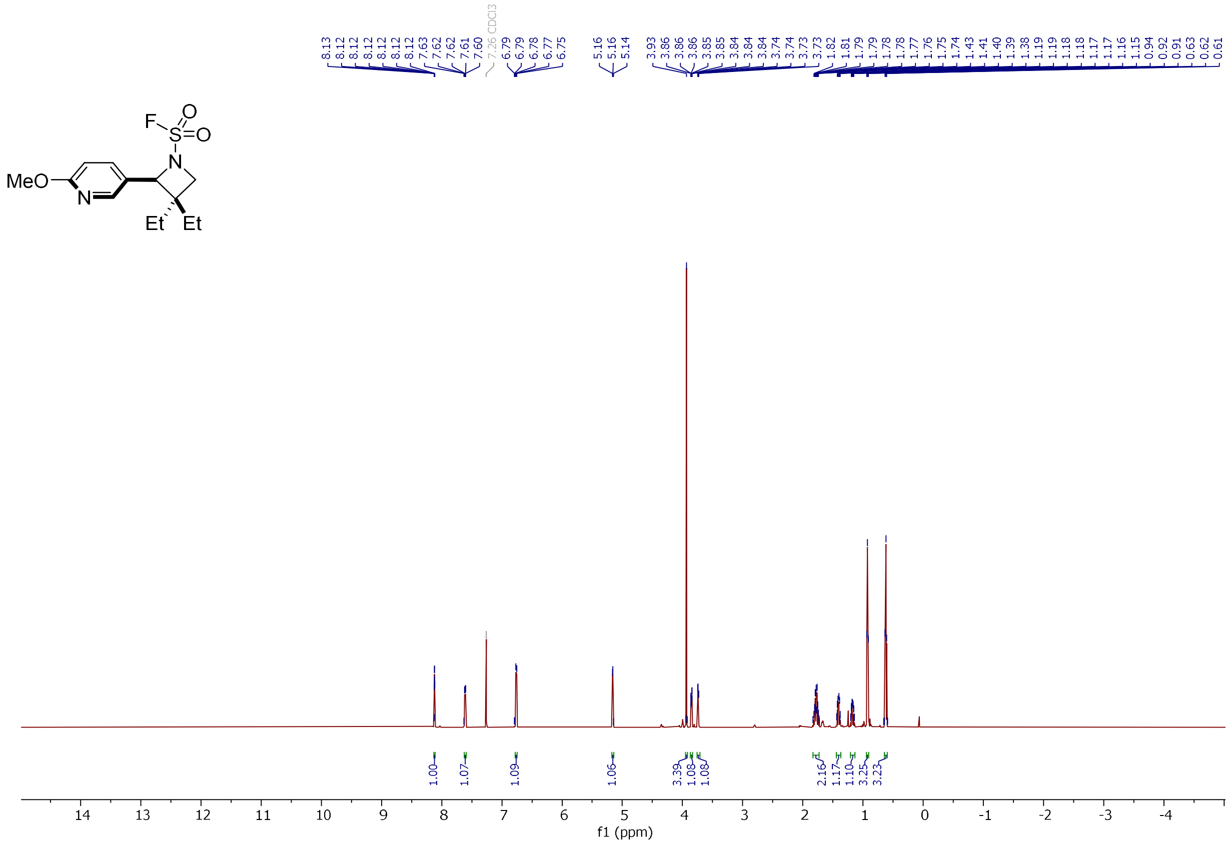

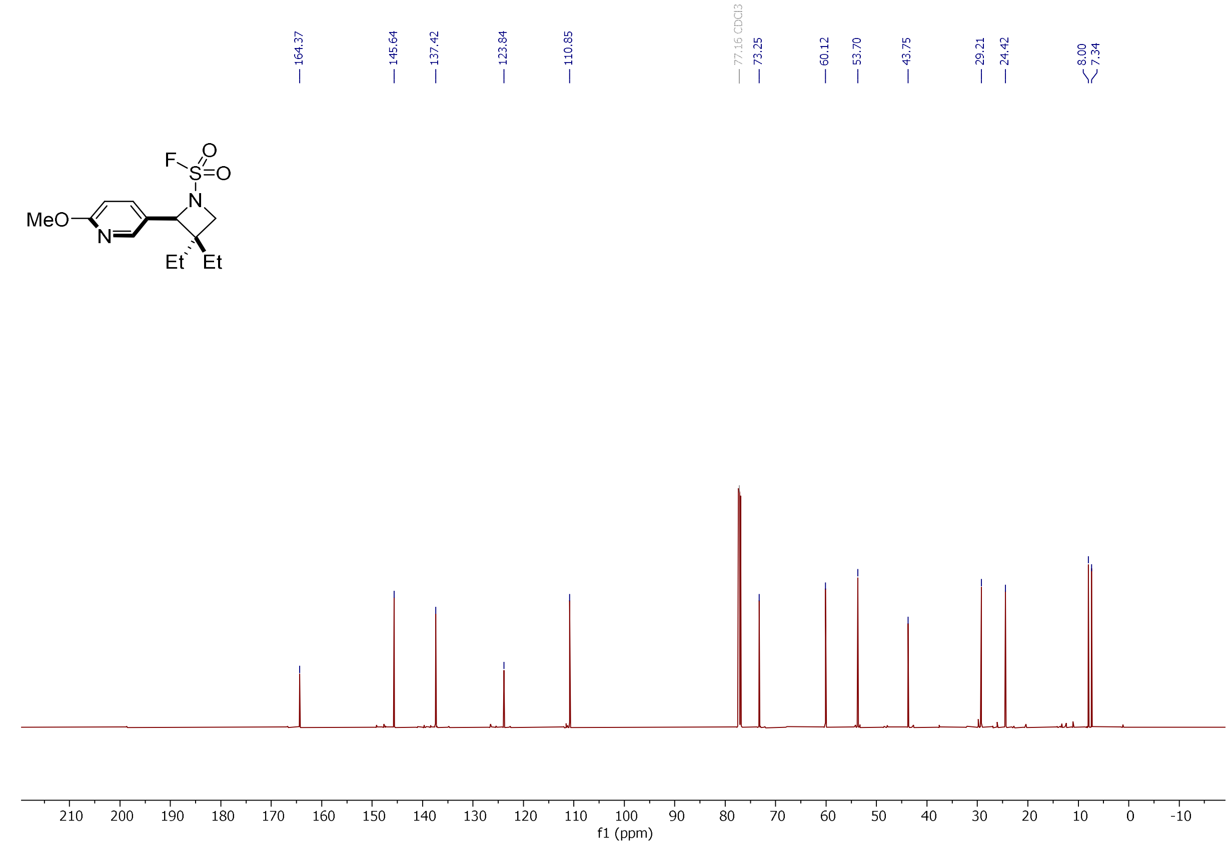

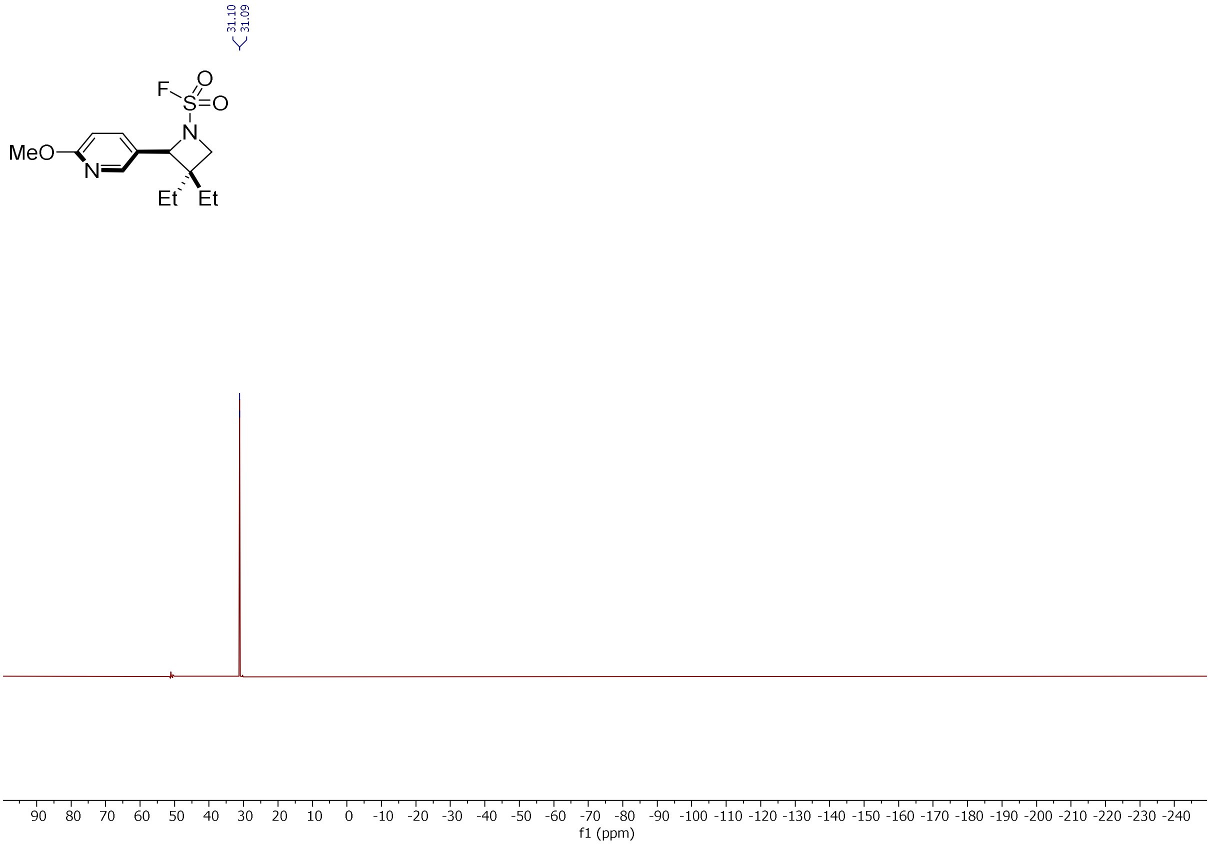

(2*R**,3*S**)-3-(2-Hydroxyethyl)-2-(4-methoxyphenyl)-3-methylazetidine-1-sulfonyl fluoride (4a) and (2*R**,3*S**)-3-(2-Hydroxyethyl)-2-(4-methoxyphenyl)-3-methylazetidine-1-sulfonyl fluoride (4a’)

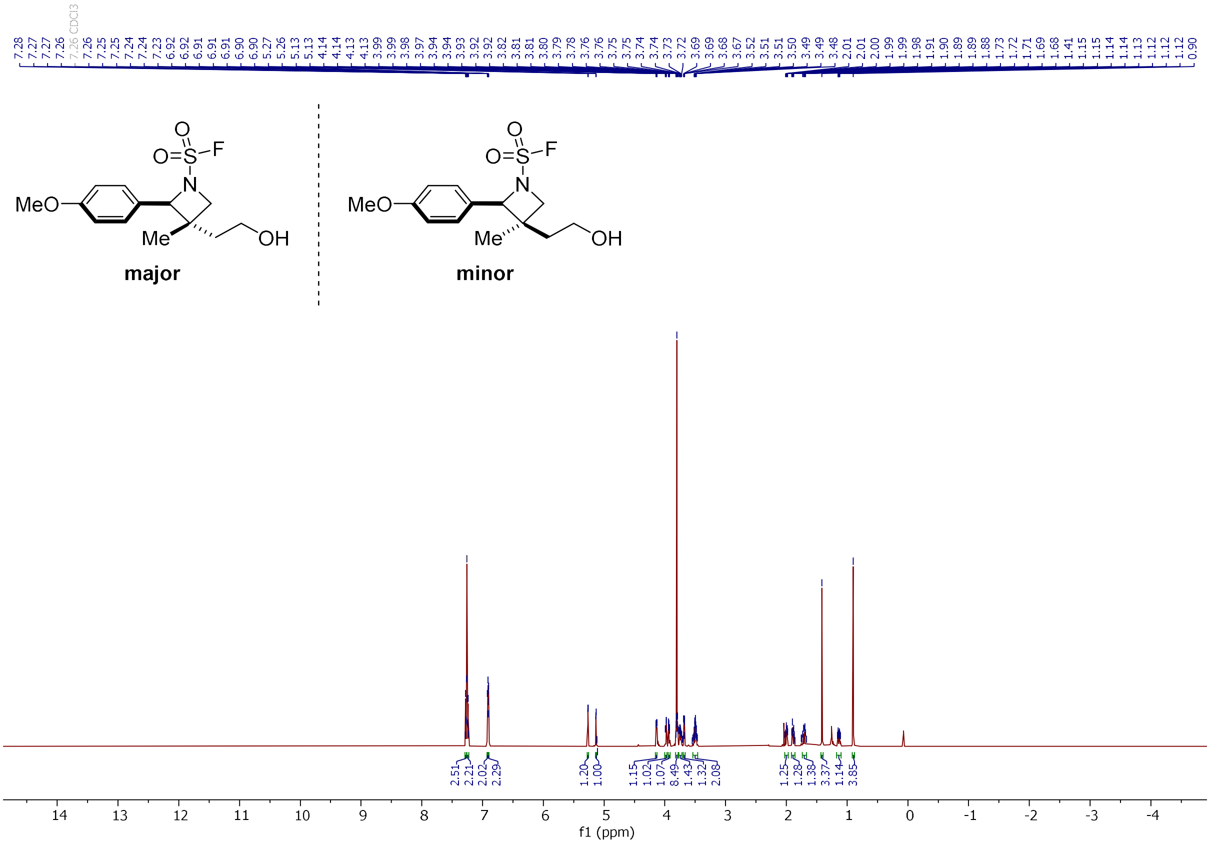

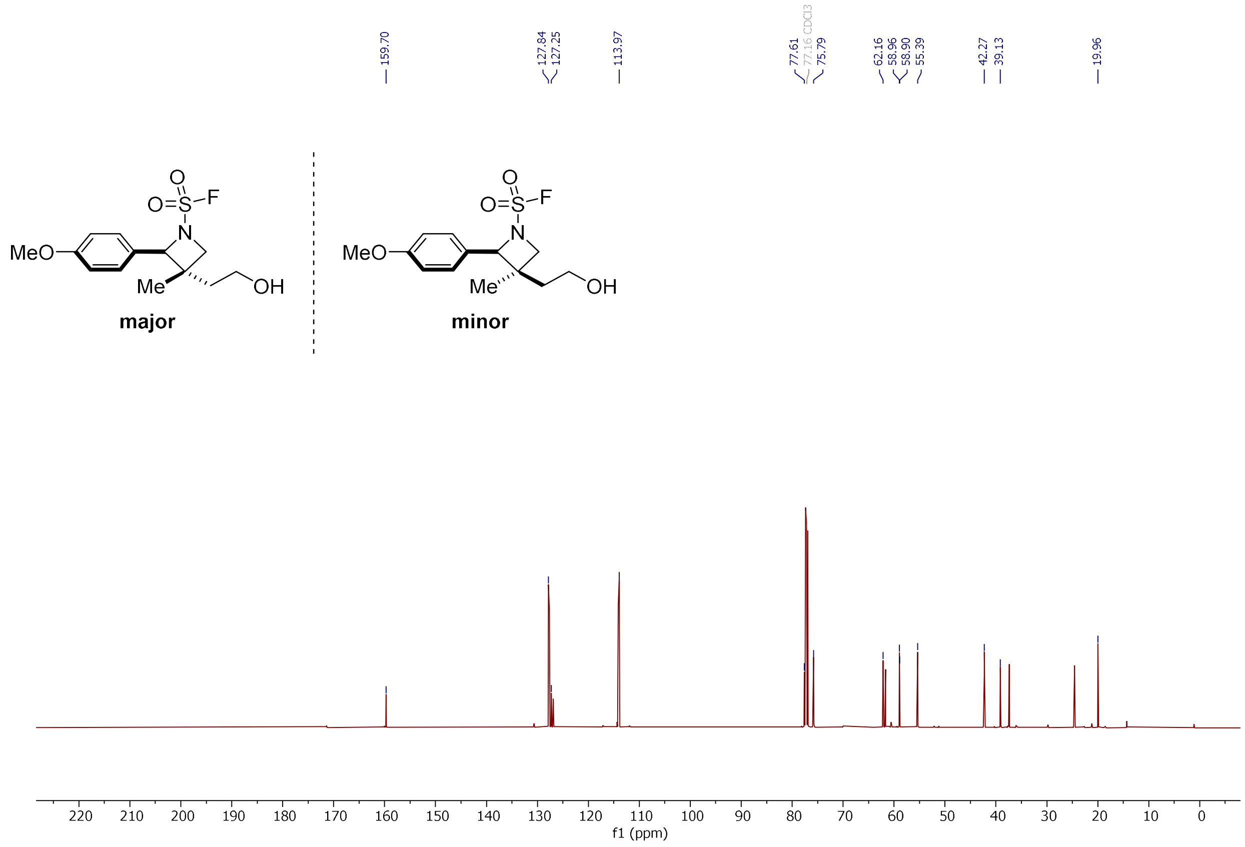
159.70
127.84
127.25
113.97
77.61
77.16 CDCl3
75.79
62.16
58.96
58.90
55.39
42.27
39.13
19.96
MeO
O=S–F
N
Me
OH
major
minor
f1 (ppm)

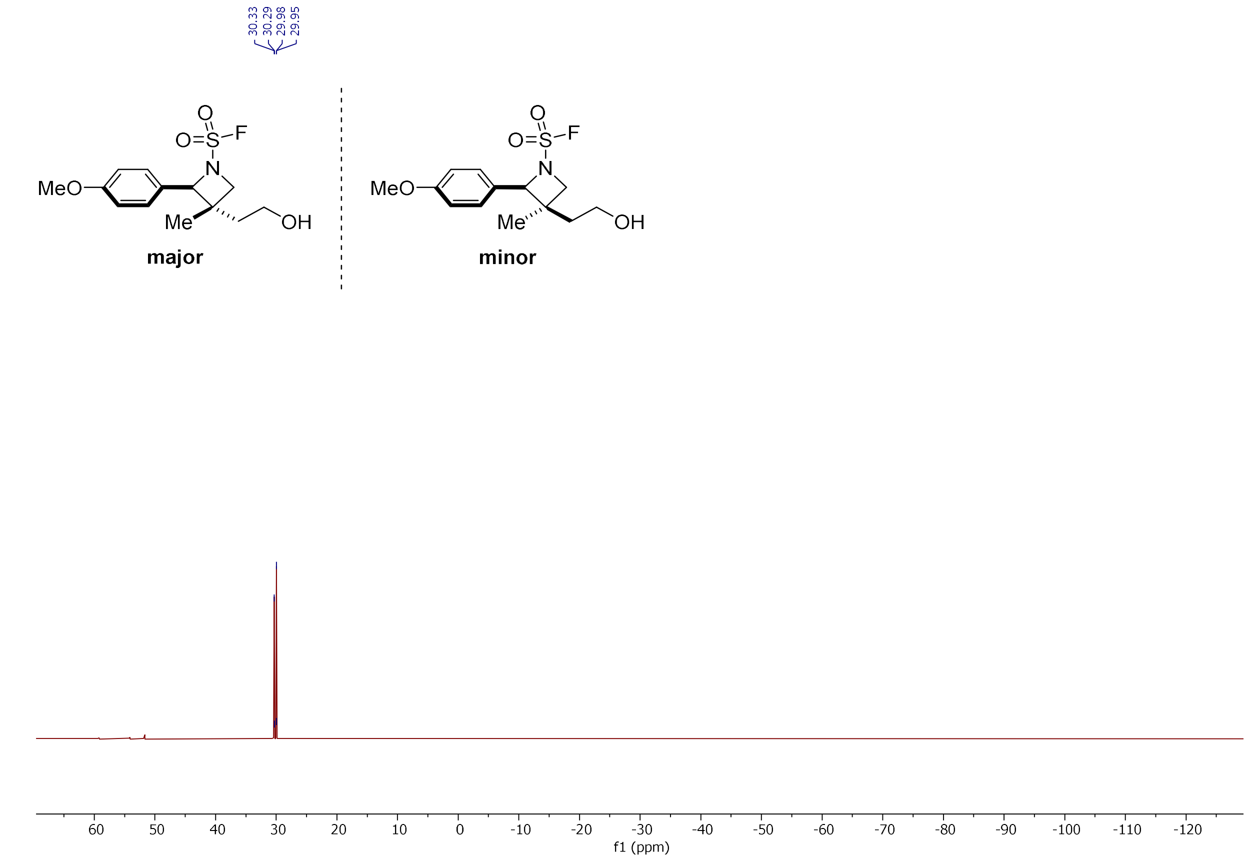
30.33
30.29
29.98
29.95
MeO
O=S–F
N
Me
OH
major
minor
f1 (ppm)

2-((2*R**,3*S**)-1-(Fluorosulfonyl)-2-(4-methoxyphenyl)-3-methylazetidin-3-yl)ethyl 4-methylbenzenesulfonate (4b)

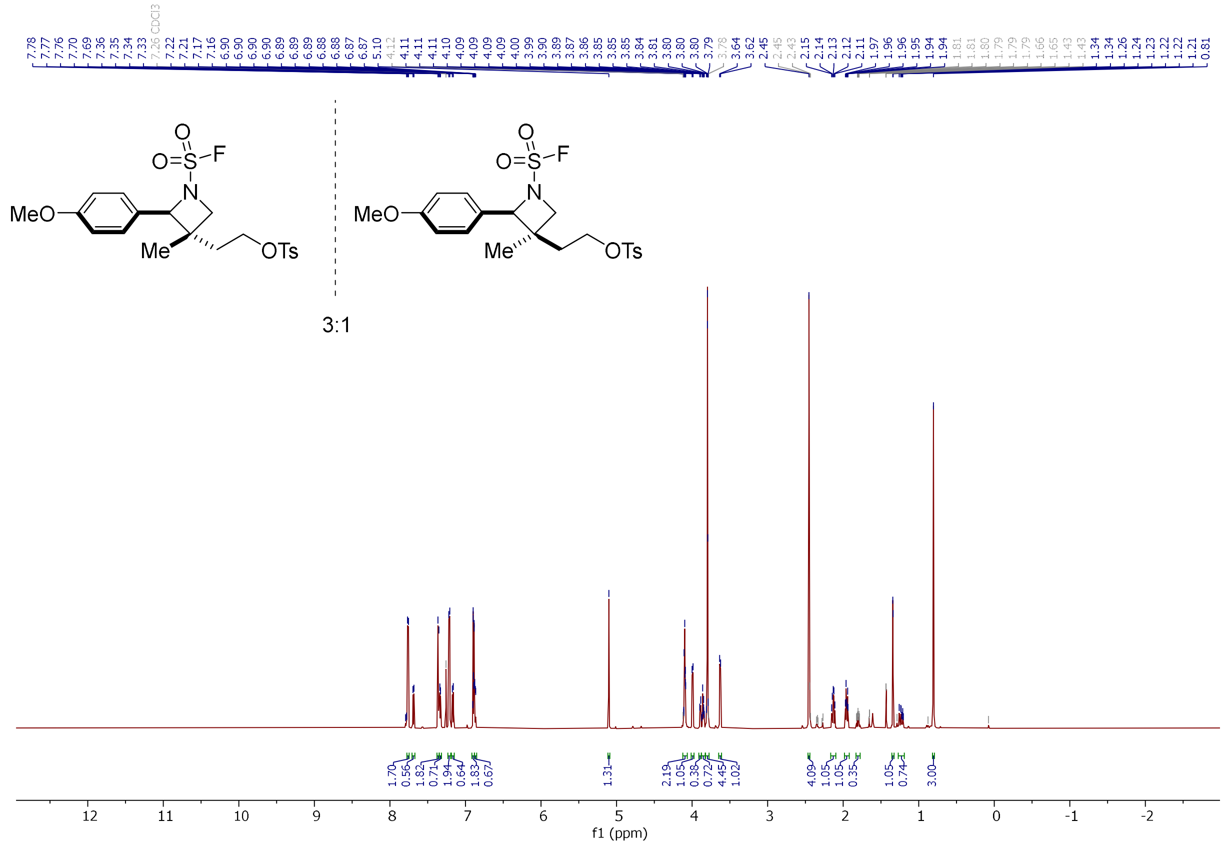

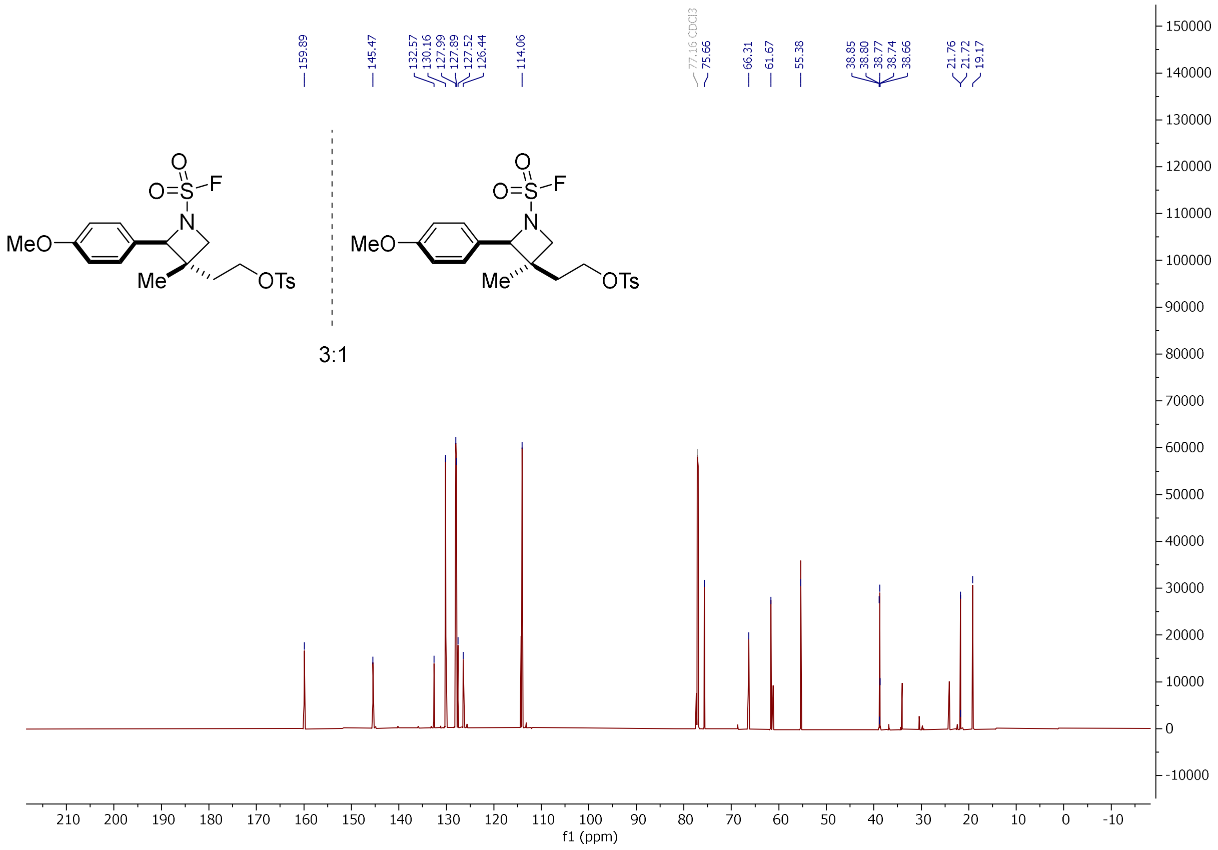

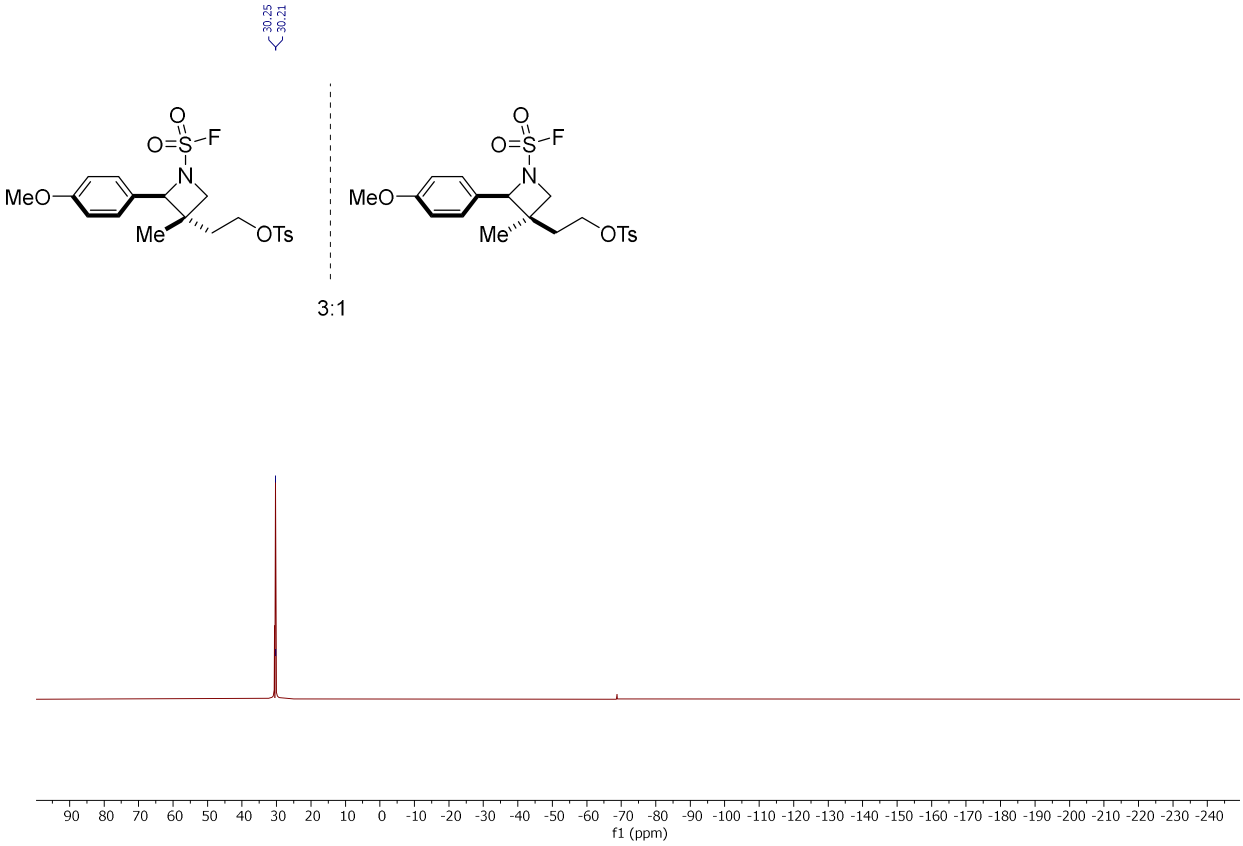

2-((2*R**,3*R**)-1-(Fluorosulfonyl)-2-(4-methoxyphenyl)-3-methylazetidin-3-yl)ethyl 4-methylbenzenesulfonate (4b’)

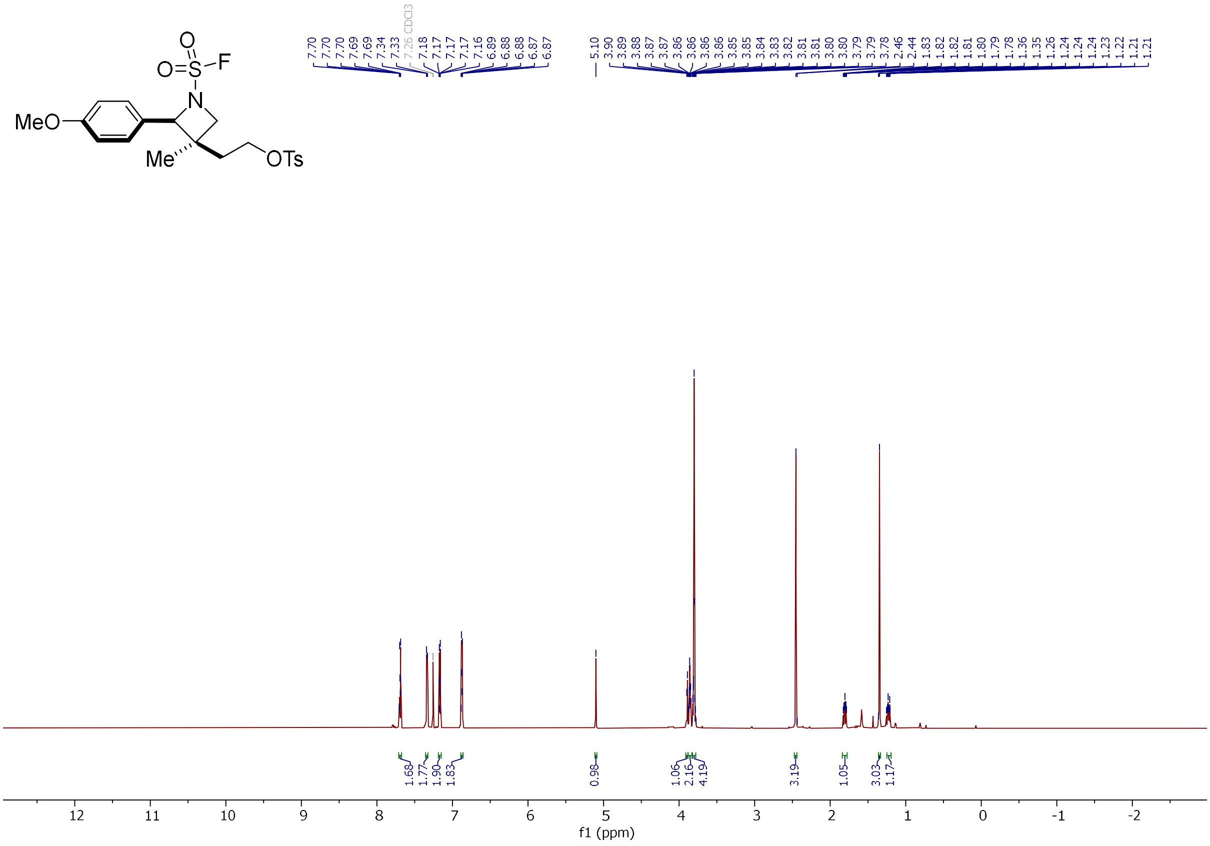

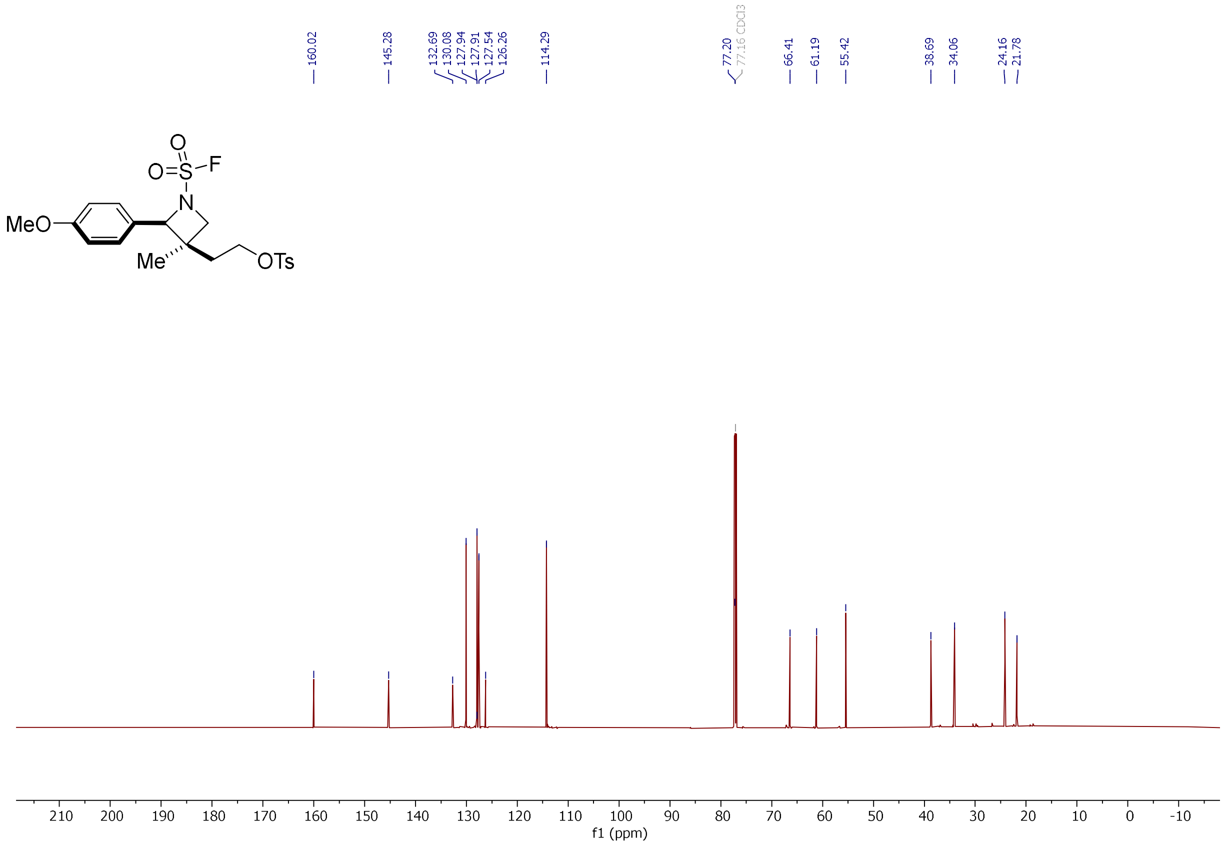
160.02
145.28
132.69
130.08
127.94
127.91
127.54
126.26
114.29
77.20
77.16 CDCl3
66.41
61.19
55.42
38.69
34.06
24.16
21.78
O=S-F
N
MeO
Me
OTs
f1 (ppm)

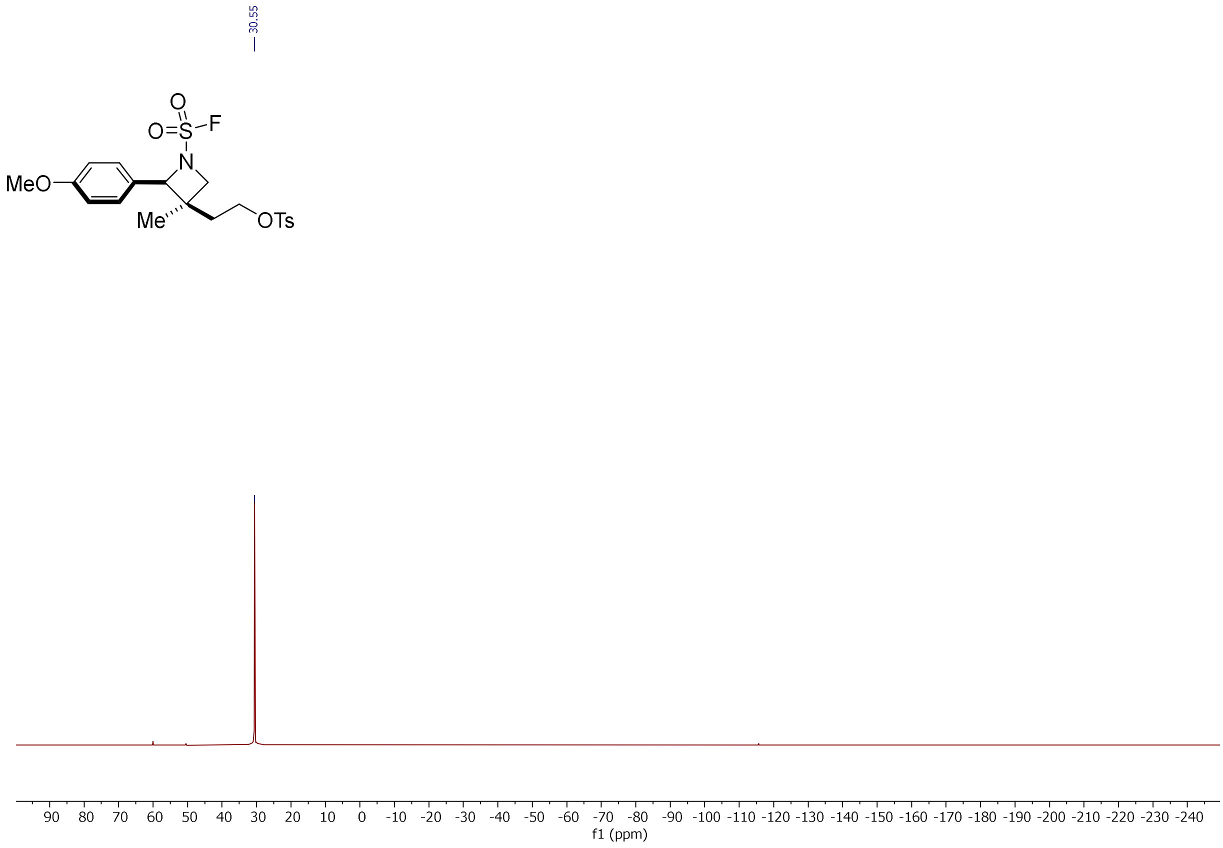
30.55
O=S-F
N
MeO
Me
OTs
f1 (ppm)

(2*R**,3*R**)-2-(4-Methoxyphenyl)-3-methyl-3-(2-((1-phenyl-1H-tetrazol-5-yl)sulfonyl)ethyl)azetidine-1-sulfonyl fluoride (4c)

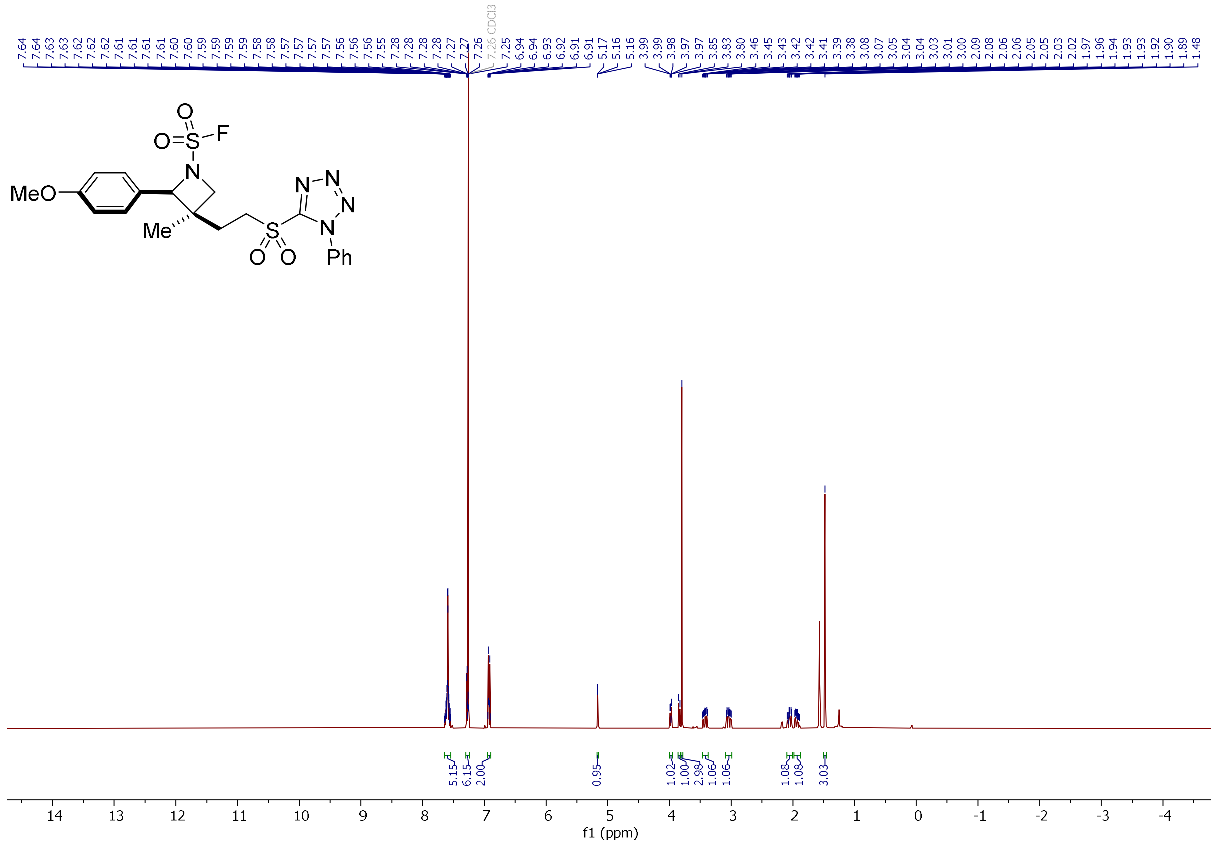

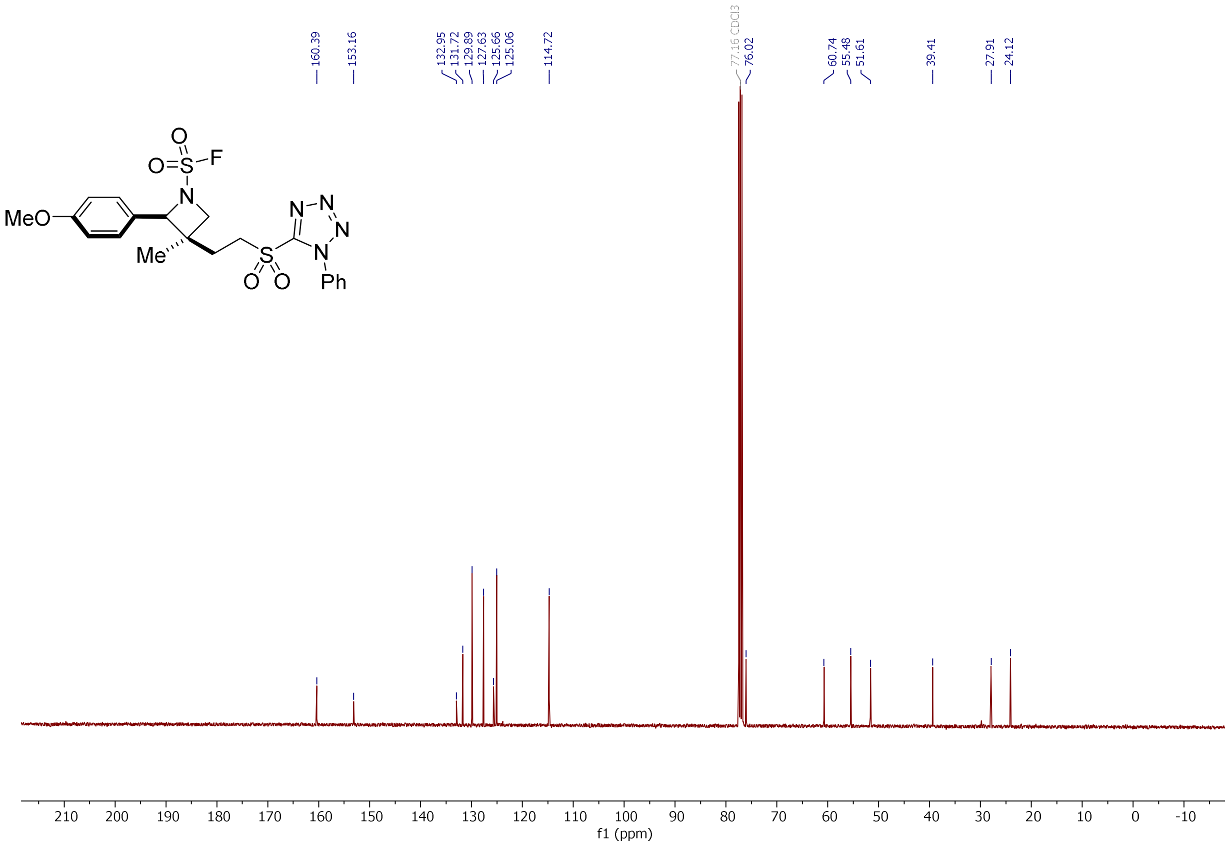

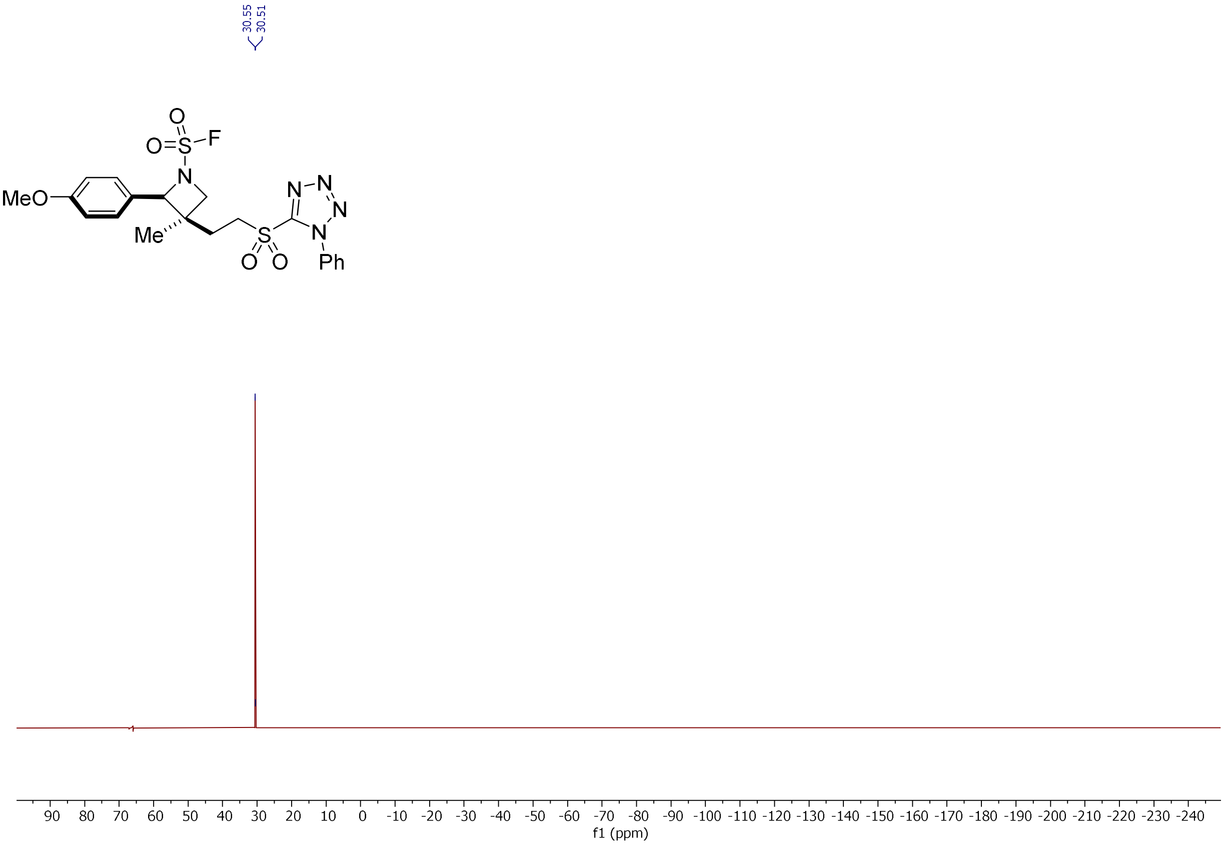

(2*R**,3*S**)-2-(4-Methoxyphenyl)-3-methyl-3-(2-((1-phenyl-1H-tetrazol-5-yl)sulfonyl)ethyl)azetidine-1-sulfonyl fluoride (4c’)

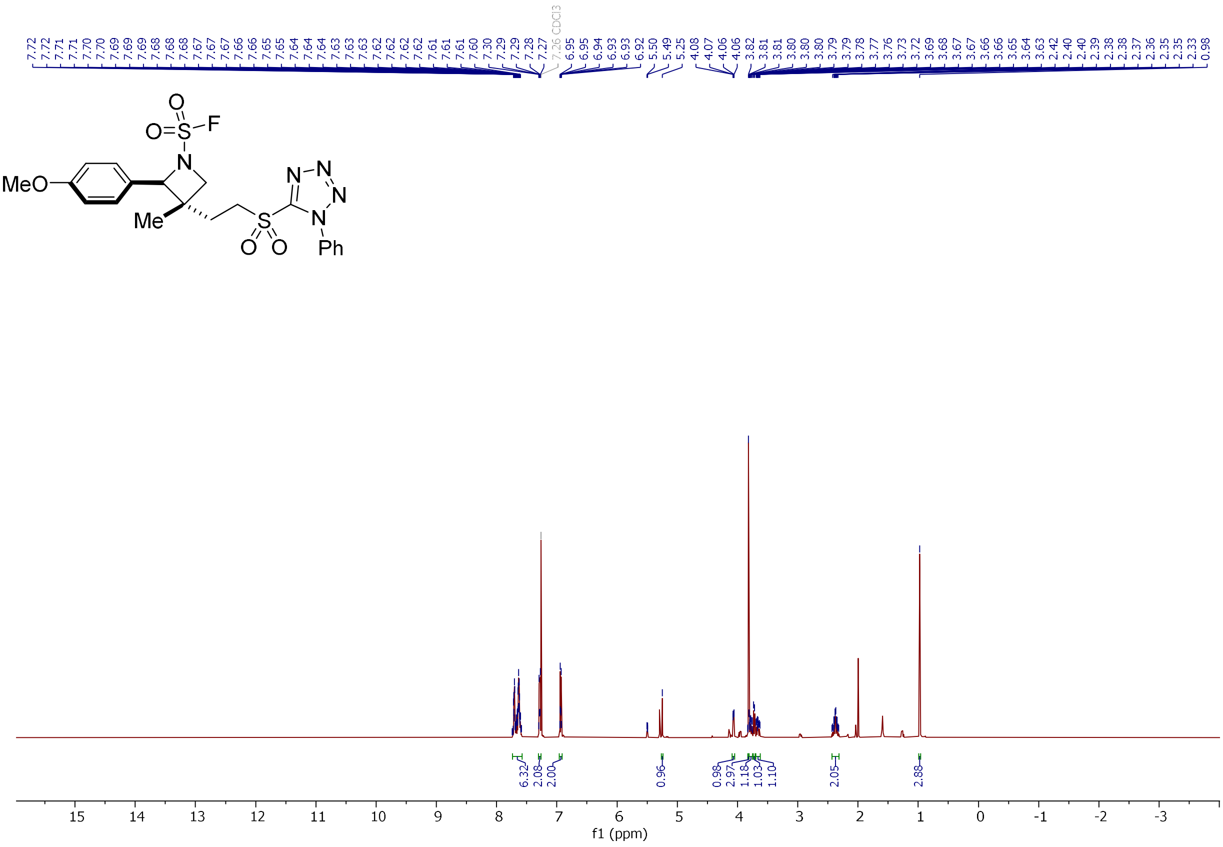

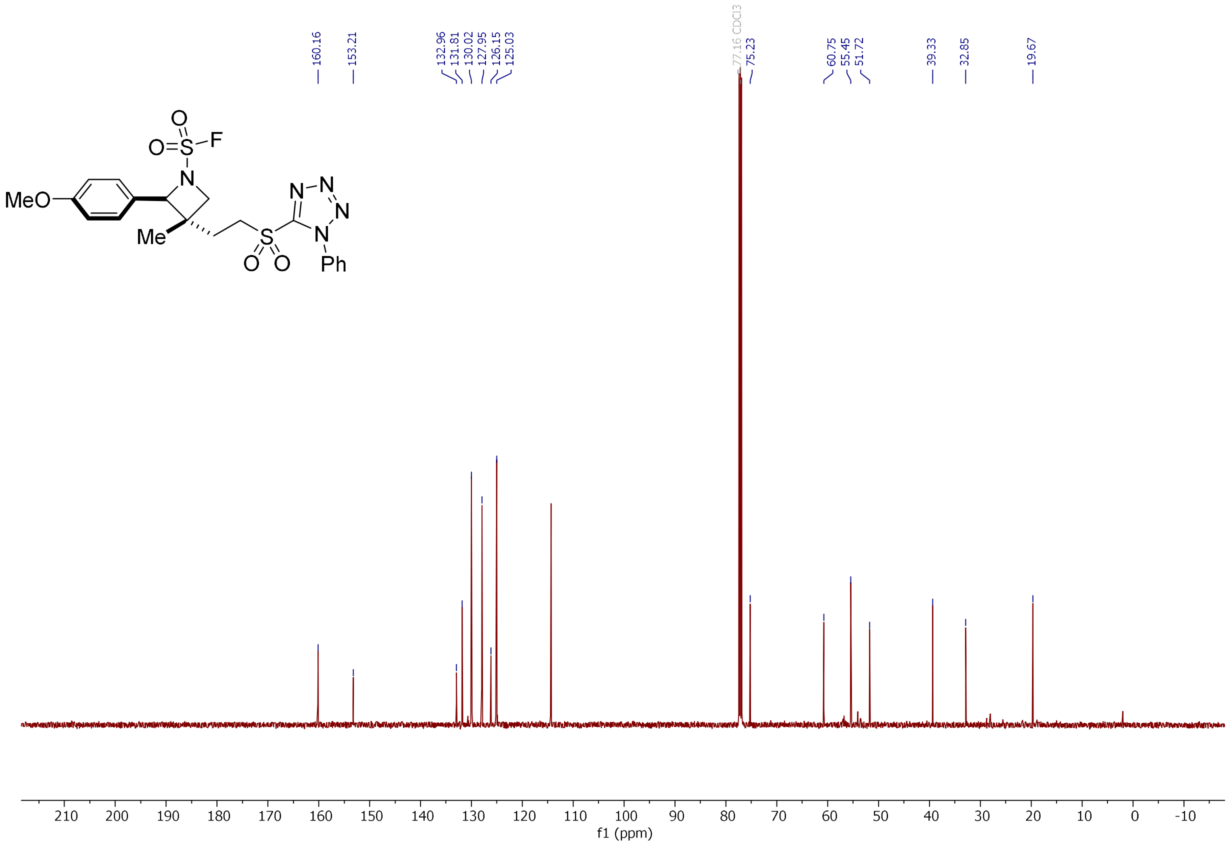
160.16
153.21
132.96
131.81
130.02
127.95
126.15
125.03
77.16 CDCl3
75.23
60.75
55.45
51.72
39.33
32.85
19.67
MeO
O
O=S−F
N
Me
N−N
N
N
Ph
S
O O
210 200 190 180 170 160 150 140 130 120 110 100 90 80 70 60 50 40 30 20 10 0 -10
f1 (ppm)

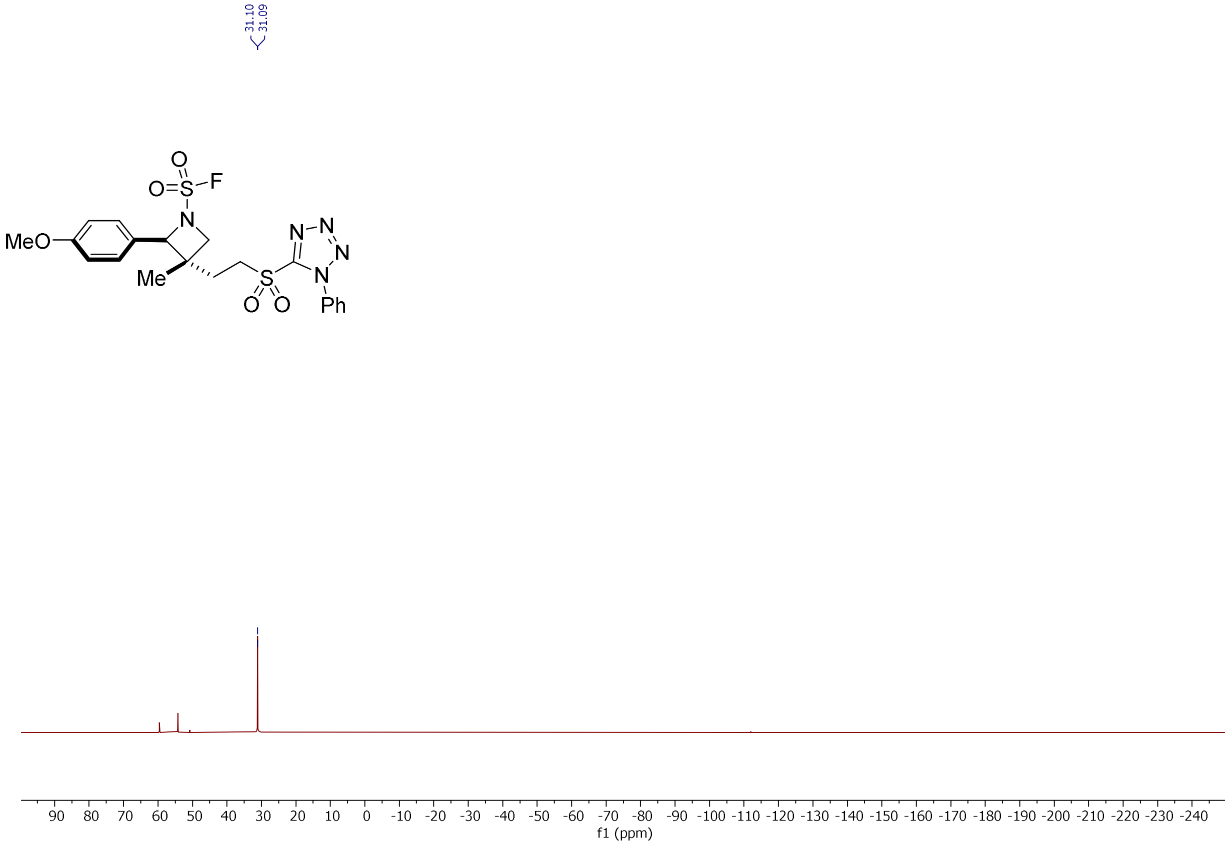
31.10
31.09
MeO
O
O=S−F
N
Me
N−N
N
N
Ph
S
O O
90 80 70 60 50 40 30 20 10 0 -10 -20 -30 -40 -50 -60 -70 -80 -90 -100 -110 -120 -130 -140 -150 -160 -170 -180 -190 -200 -210 -220 -230 -240
f1 (ppm)

(2*R**,3*S**)-2-(4-Methoxyphenyl)-3-methyl-3-(3-oxobutyl)azetidine-1-sulfonyl fluoride (4d)

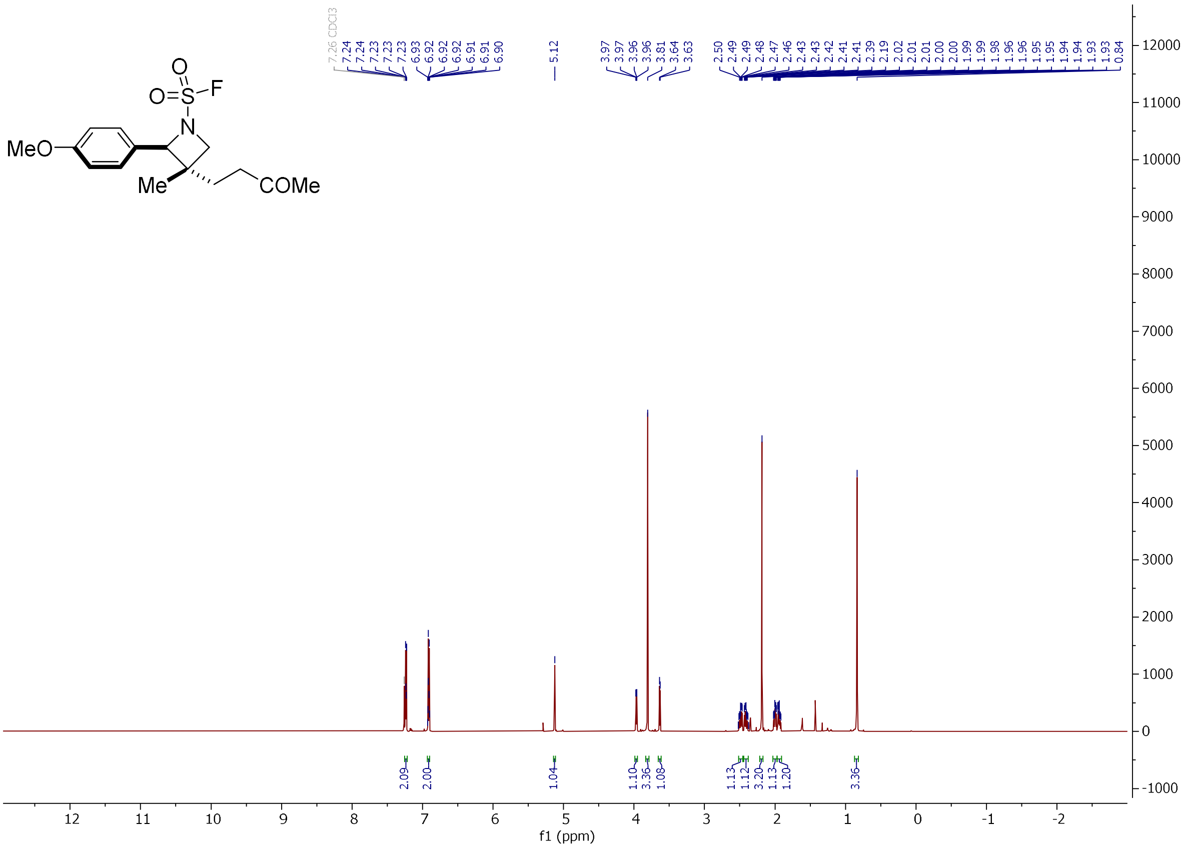

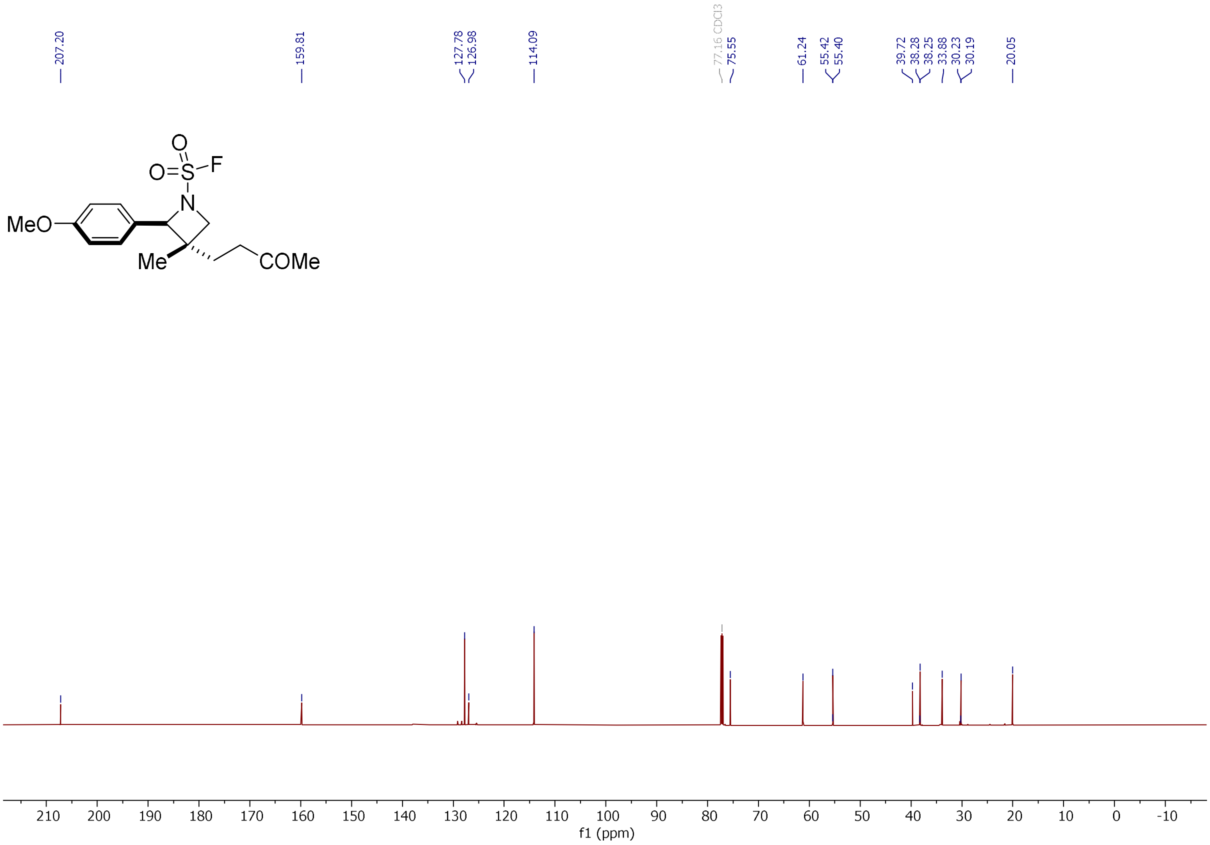

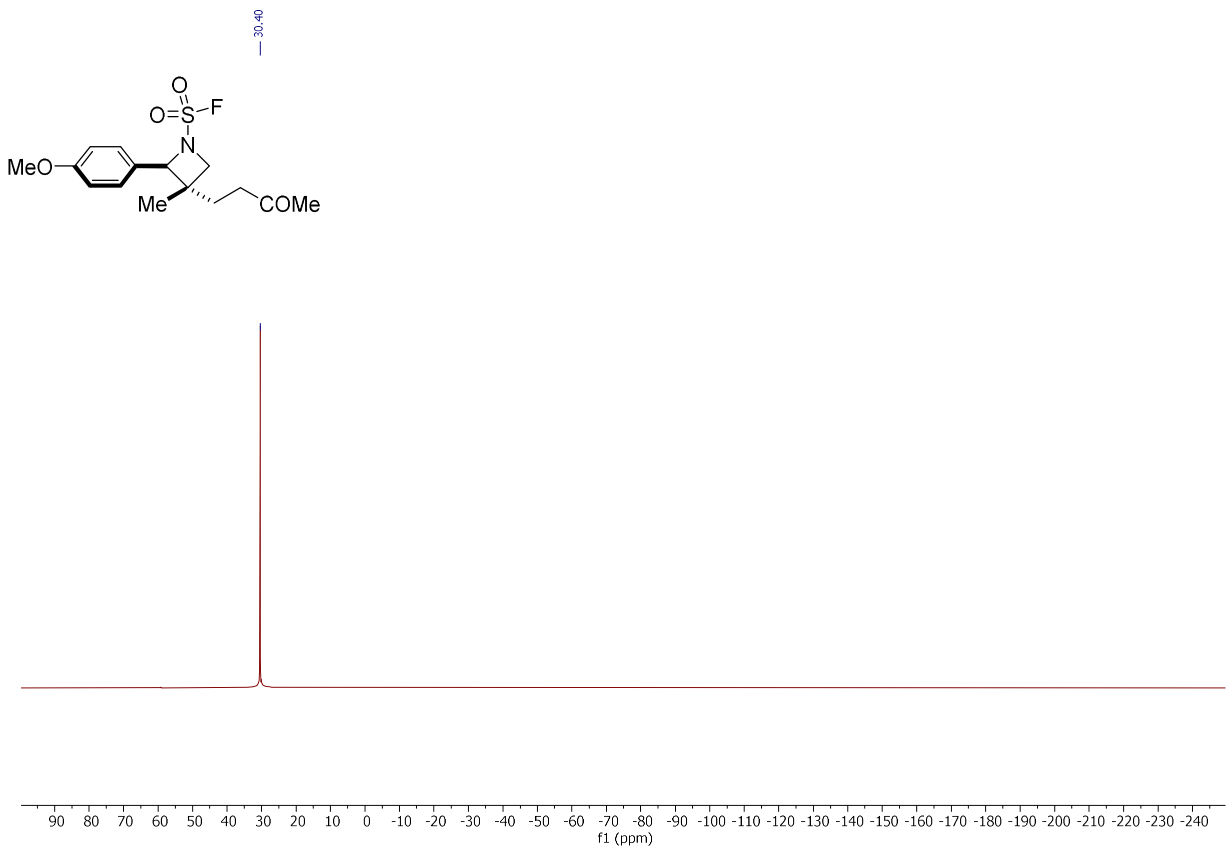

(2*R**,3*R**)-2-(4-Methoxyphenyl)-3-methyl-3-(3-oxobutyl)azetidine-1-sulfonyl fluoride (4d')

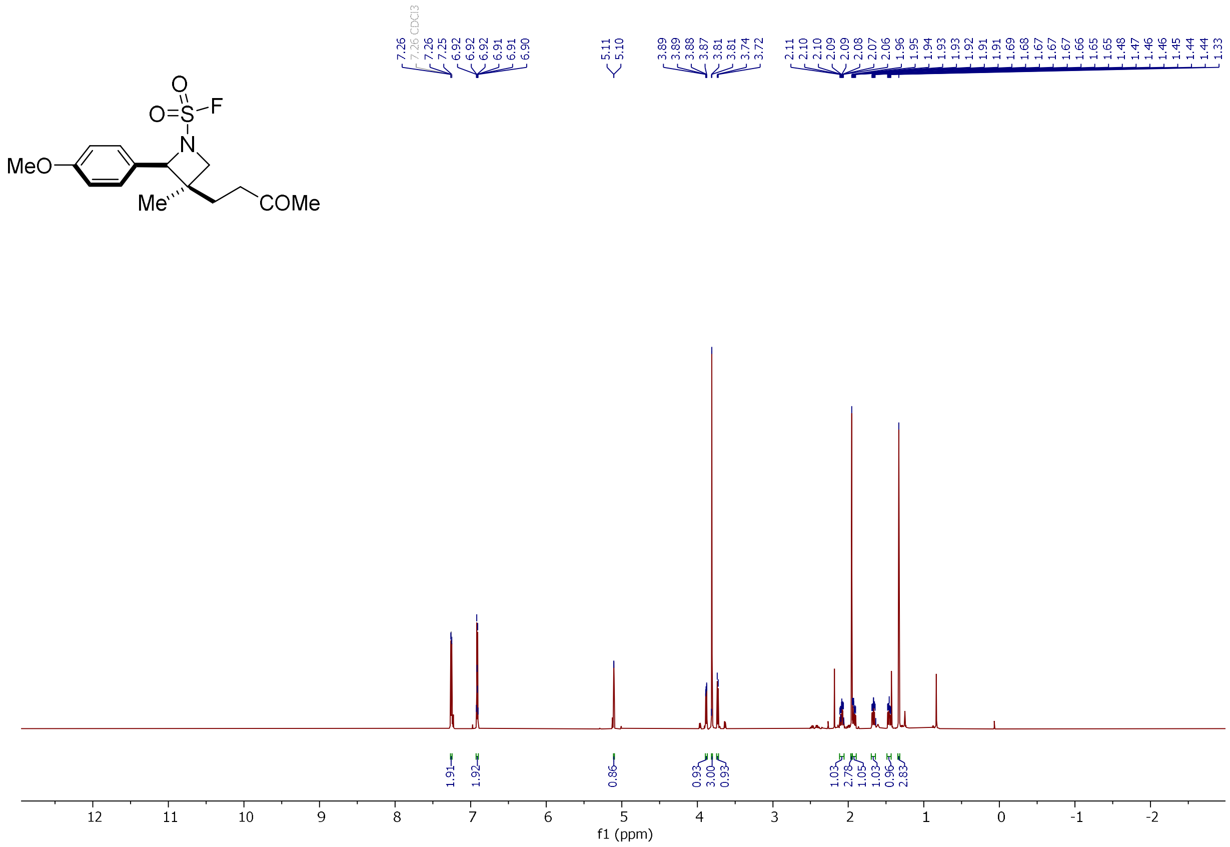

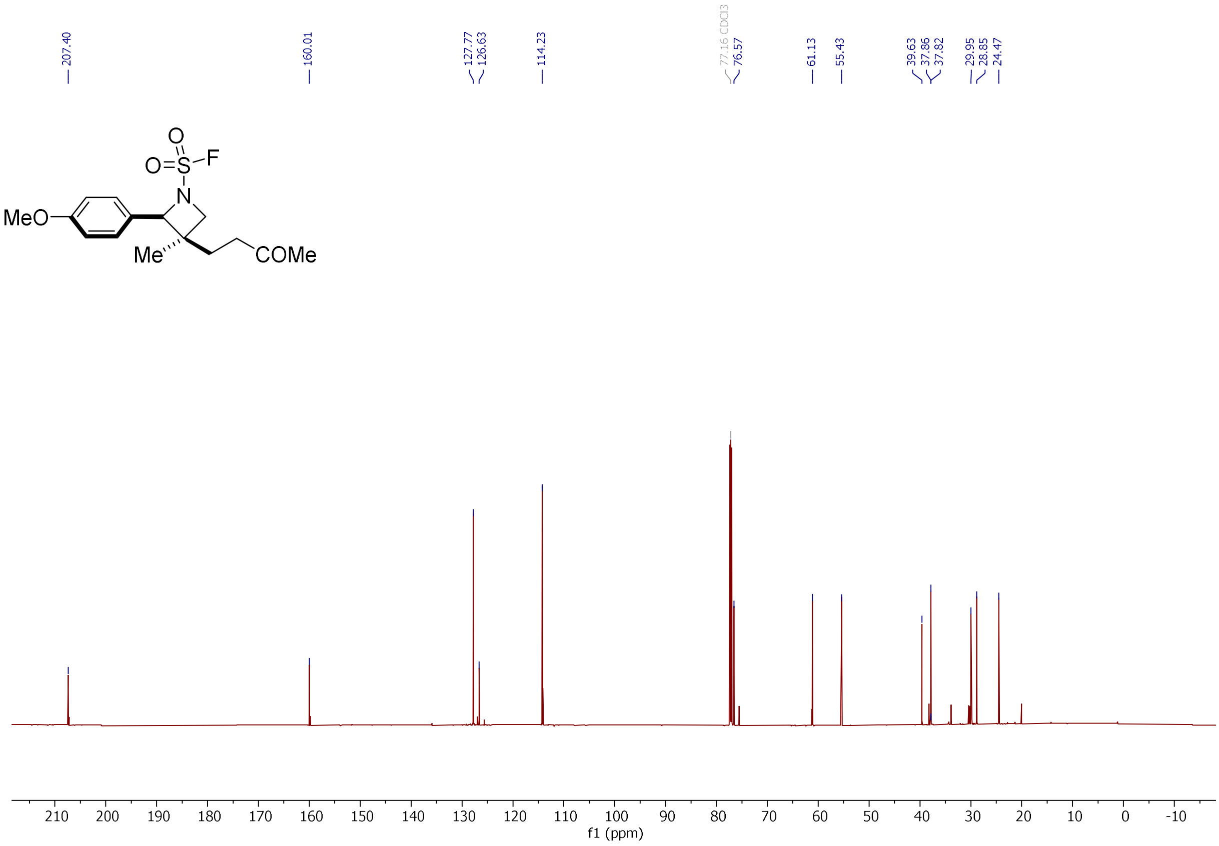
207.40
160.01
127.77
126.63
114.23
77.16 CDCl3
76.57
61.13
55.43
39.63
37.86
37.82
29.95
28.85
24.47
MeO
O=S-F
N
Me
COMe
f1 (ppm)

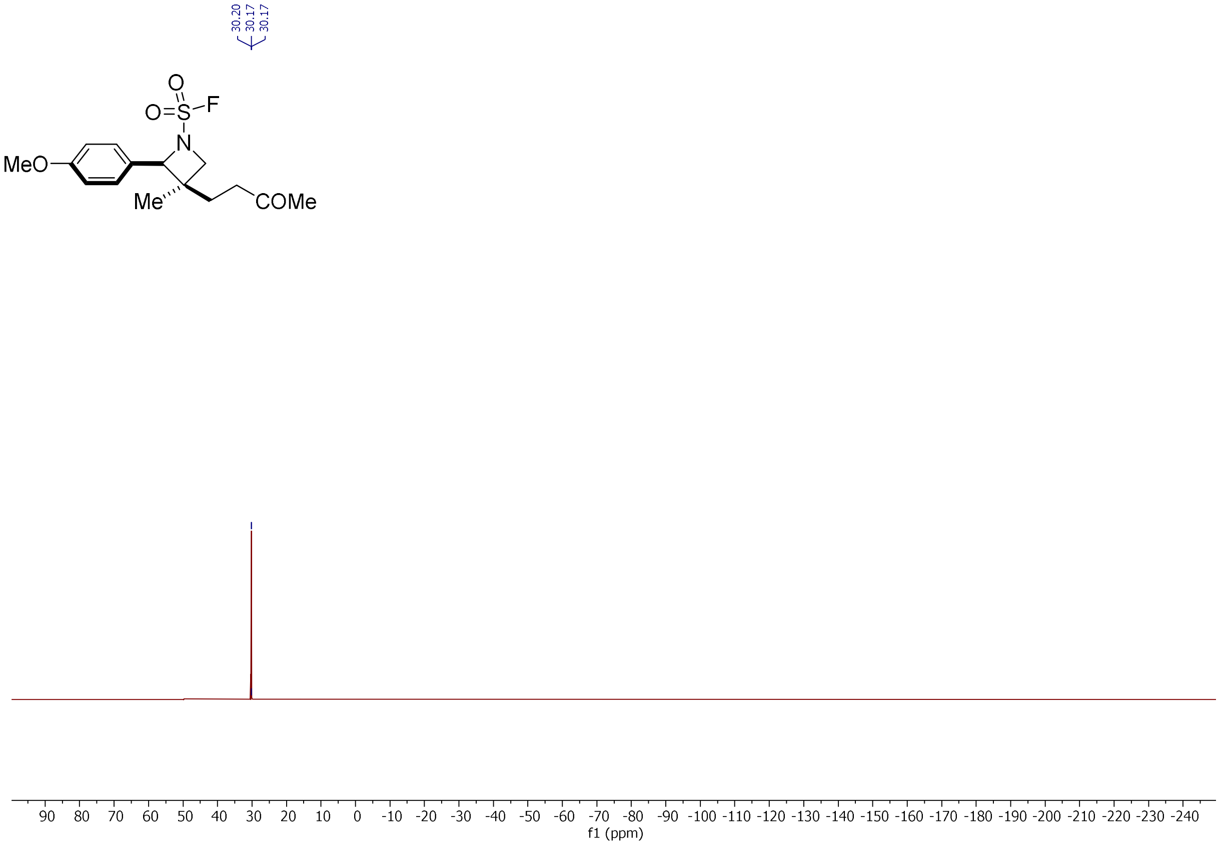
30.20
30.17
30.17
MeO
O=S-F
N
Me
COMe
f1 (ppm)

2-((2*R**,3*S**)-1-(Fluorosulfonyl)-2-(4-methoxyphenyl)-3-methylazetidin-3-yl)ethyl but-3-enoate (4e) and 2-((2*R**,3*R**)-1-(Fluorosulfonyl)-2-(4-methoxyphenyl)-3-methylazetidin-3-yl)ethyl but-3-enoate (4e')

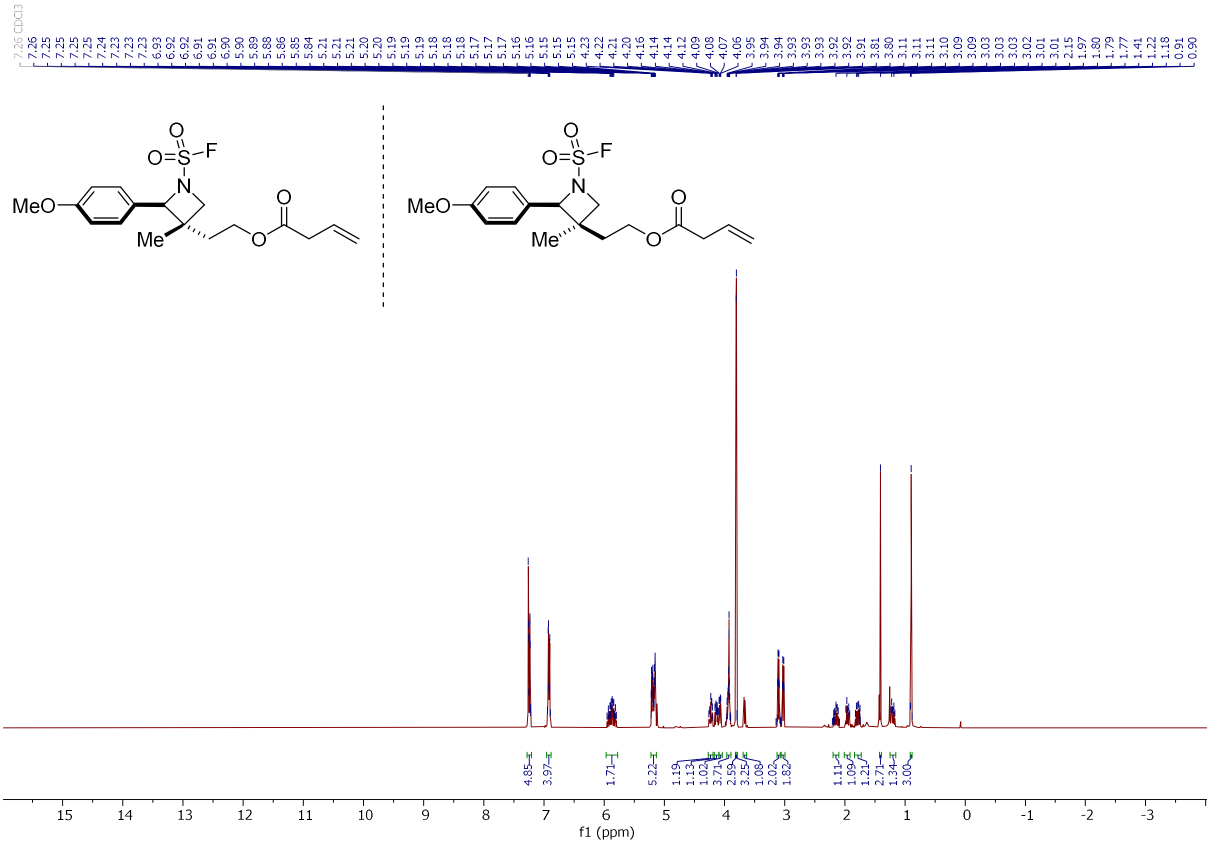

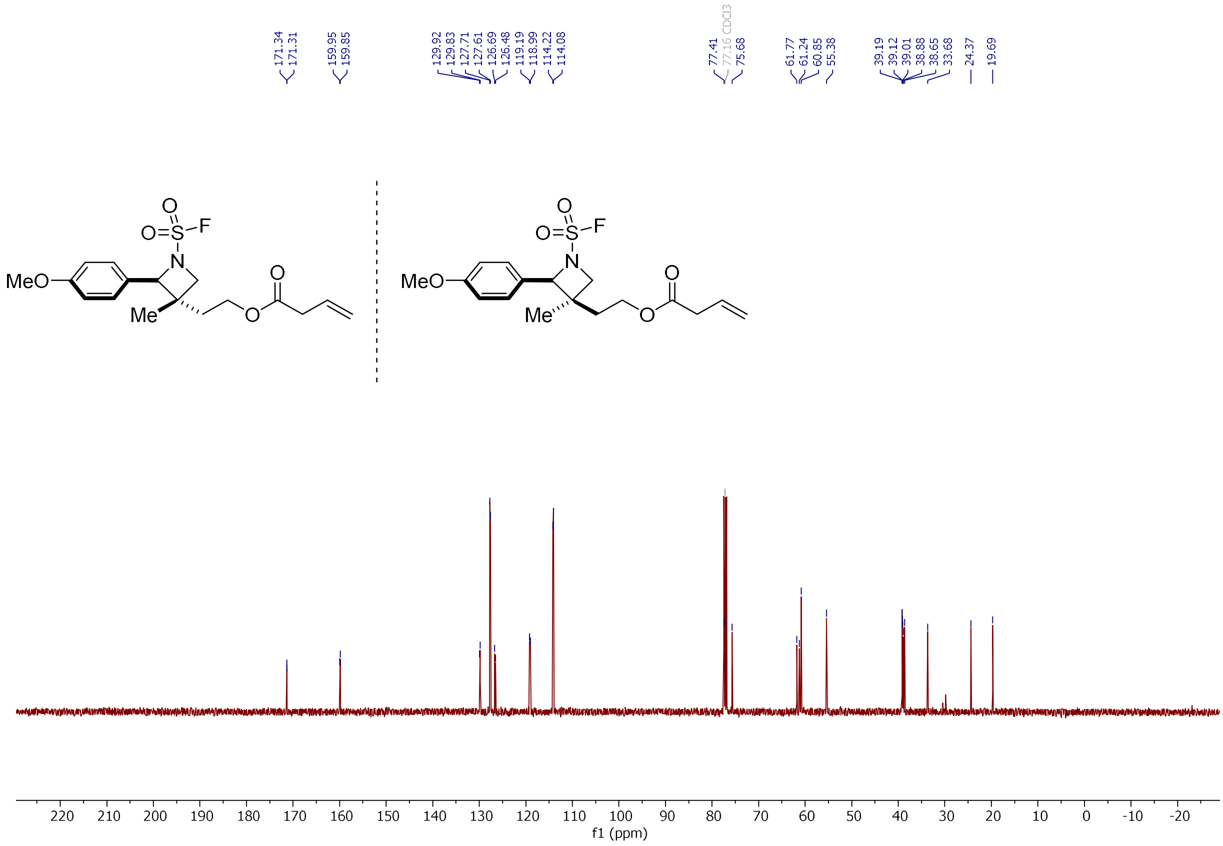
171.34
171.31
159.95
159.85
129.92
129.83
127.71
127.61
126.69
126.48
119.19
118.99
114.22
114.08
77.41
77.16 CDCl3
75.68
61.77
61.24
60.85
55.38
39.19
39.12
39.01
38.88
38.65
33.68
24.37
19.69
O=S-F
N
MeO
Me
O
f1 (ppm)

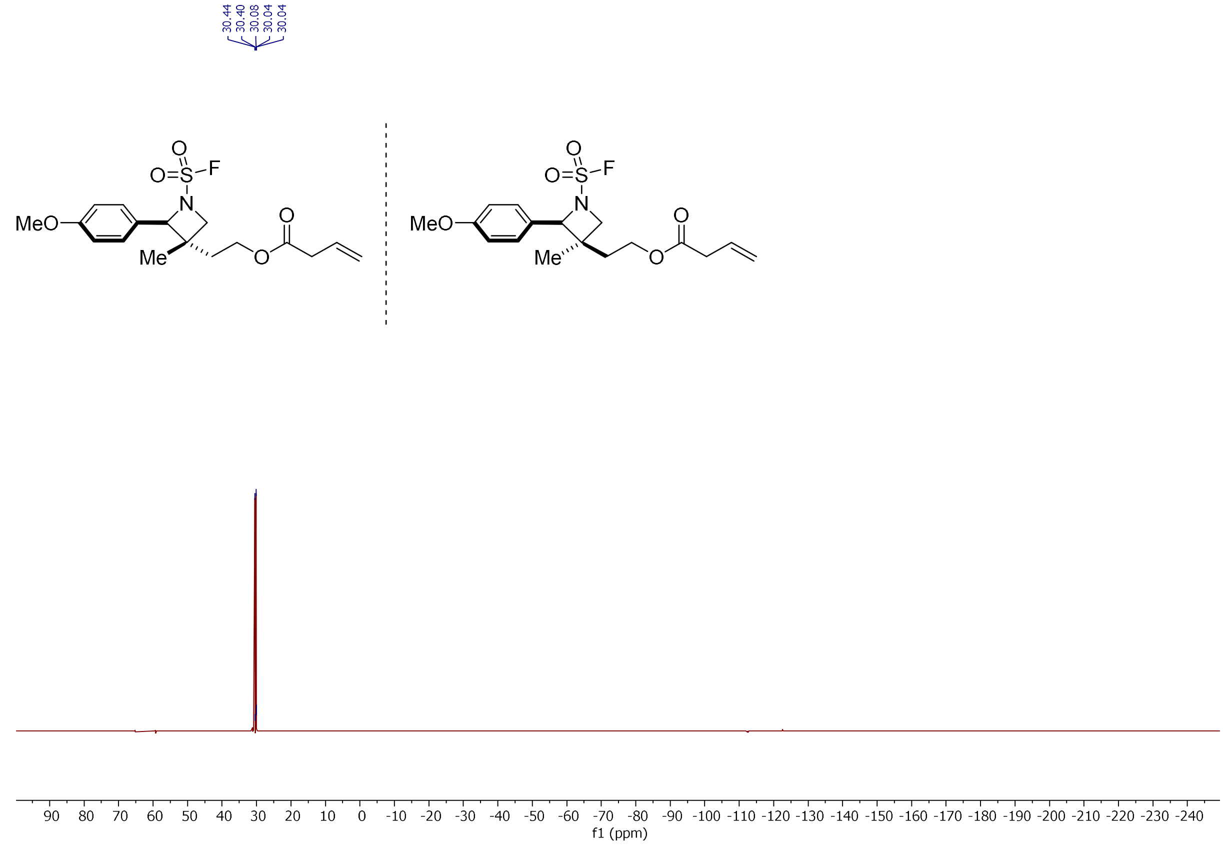
30.44
30.40
30.08
30.04
30.04
O=S-F
N
MeO
Me
O
f1 (ppm)

(1*R**,4*r**,6*R**)-6-Cyano-1-(4-methoxyphenyl)-2-azaspiro[3.3]heptane-2-sulfonyl fluoride (4f)

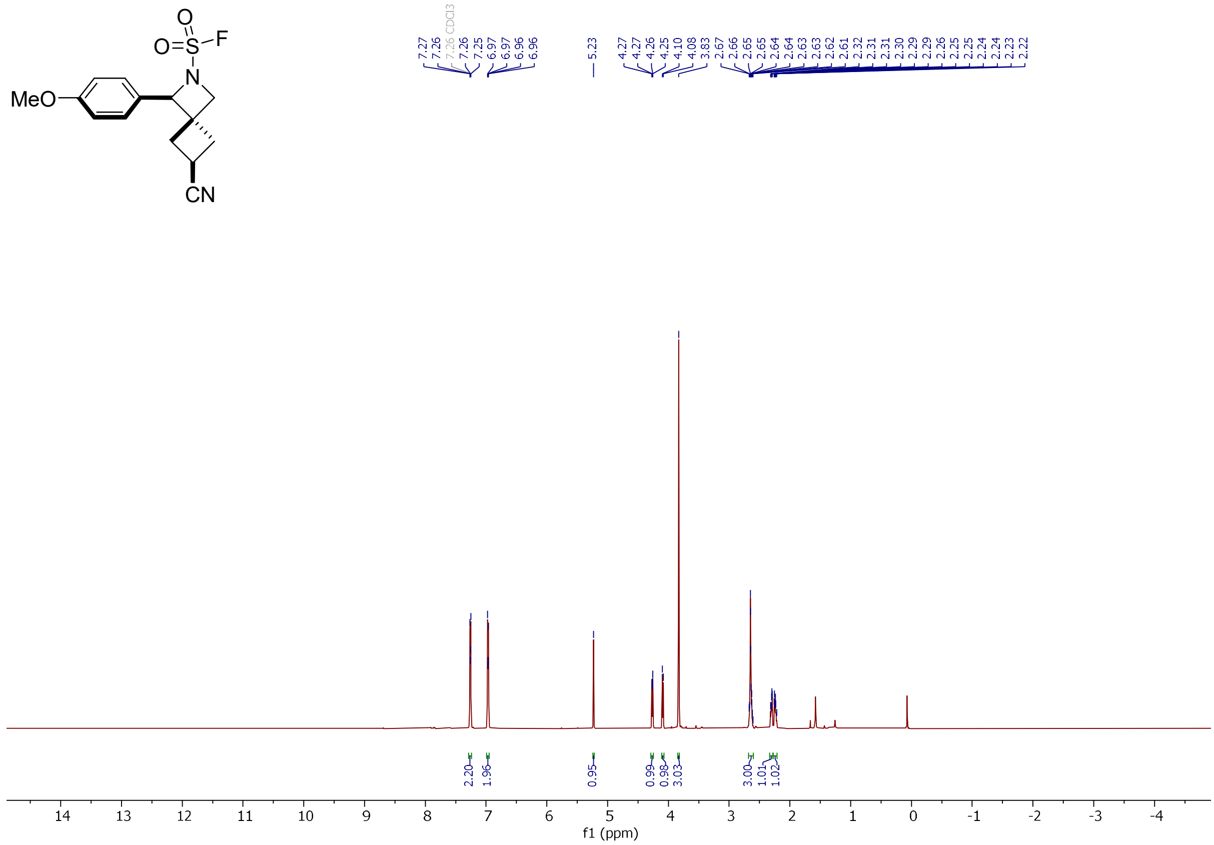

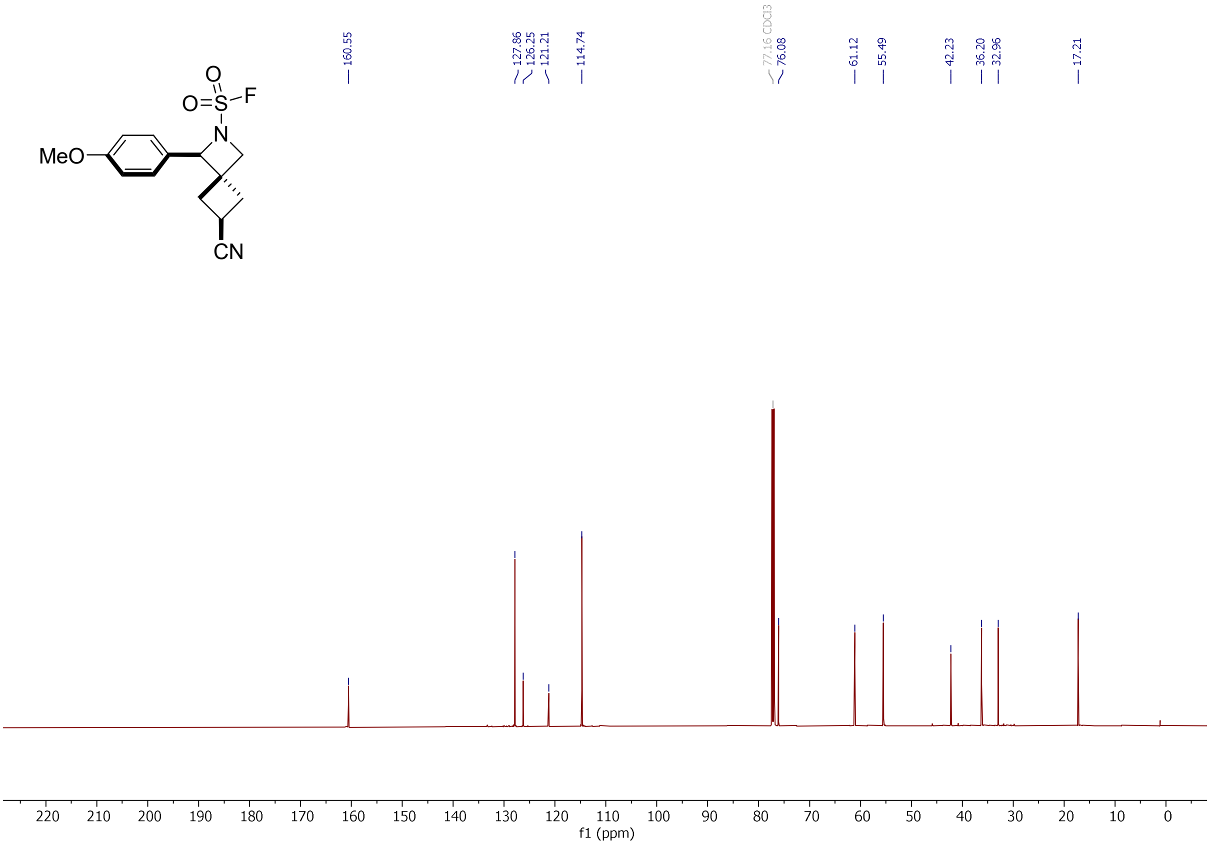
MeO
O
O=S-F
N
CN
160.55
127.86
126.25
121.21
114.74
77.16 CDCl3
76.08
61.12
55.49
42.23
36.20
32.96
17.21
220 210 200 190 180 170 160 150 140 130 120 110 100 90 80 70 60 50 40 30 20 10 0
f1 (ppm)

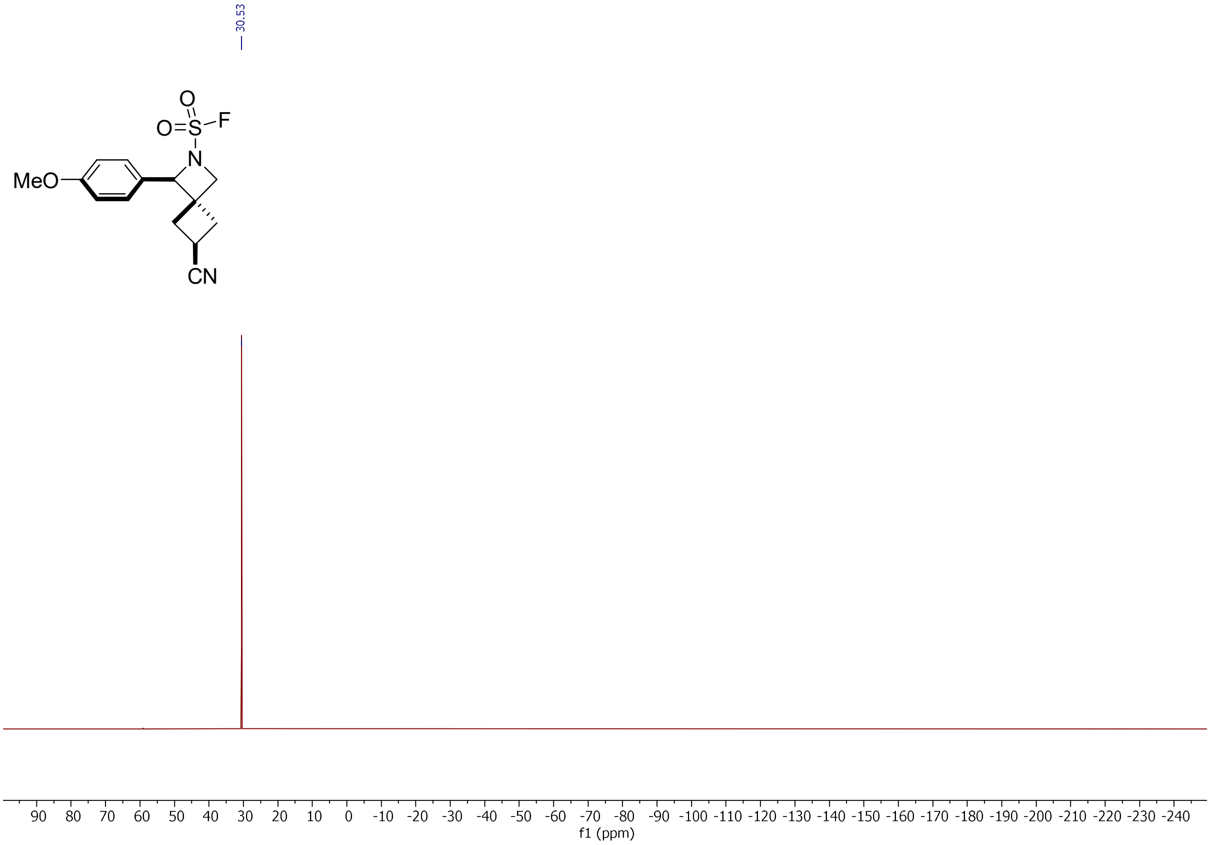
MeO
O
O=S-F
N
CN
30.53
90 80 70 60 50 40 30 20 10 0 -10 -20 -30 -40 -50 -60 -70 -80 -90 -100 -110 -120 -130 -140 -150 -160 -170 -180 -190 -200 -210 -220 -230 -240
f1 (ppm)

(1*R**,4*S**,6*S**)-6-Cyano-1-(4-methoxyphenyl)-2-azaspiro[3.3]heptane-2-sulfonyl fluoride (4f’)

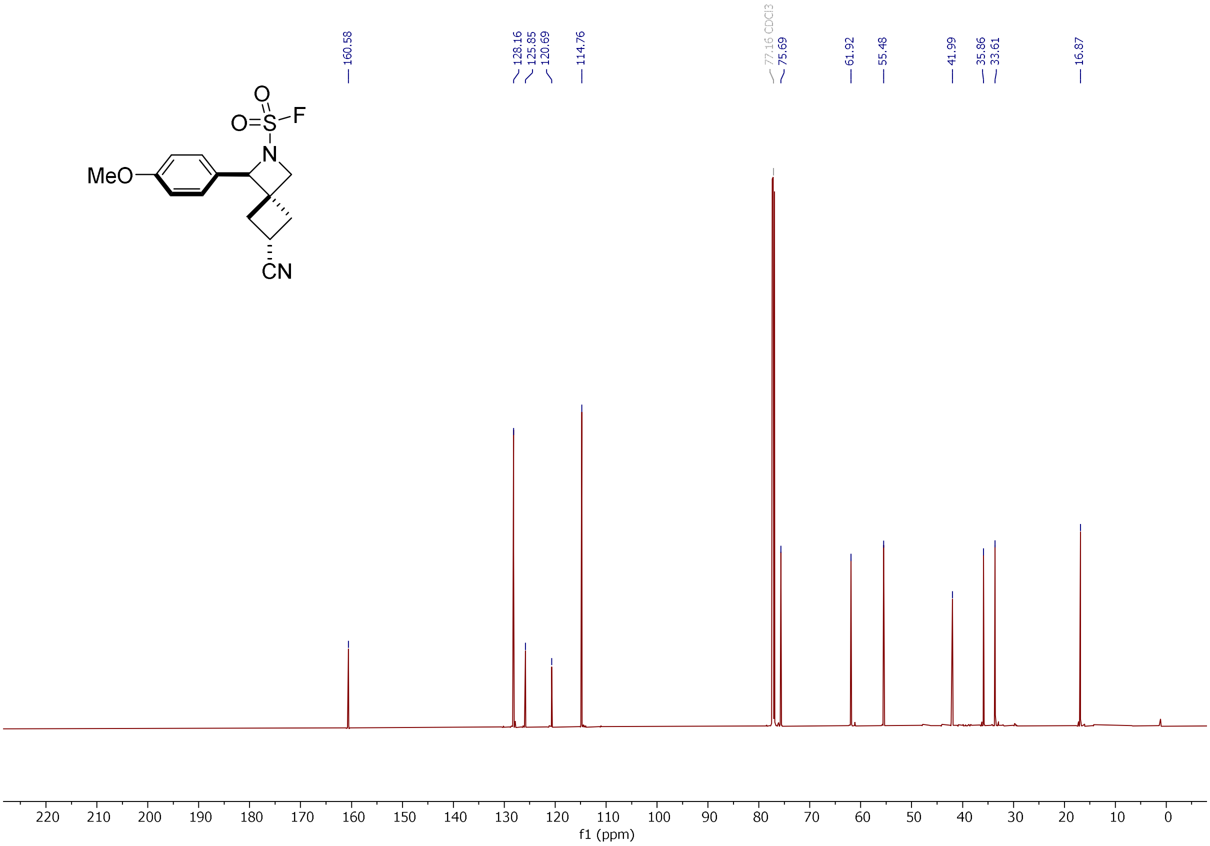

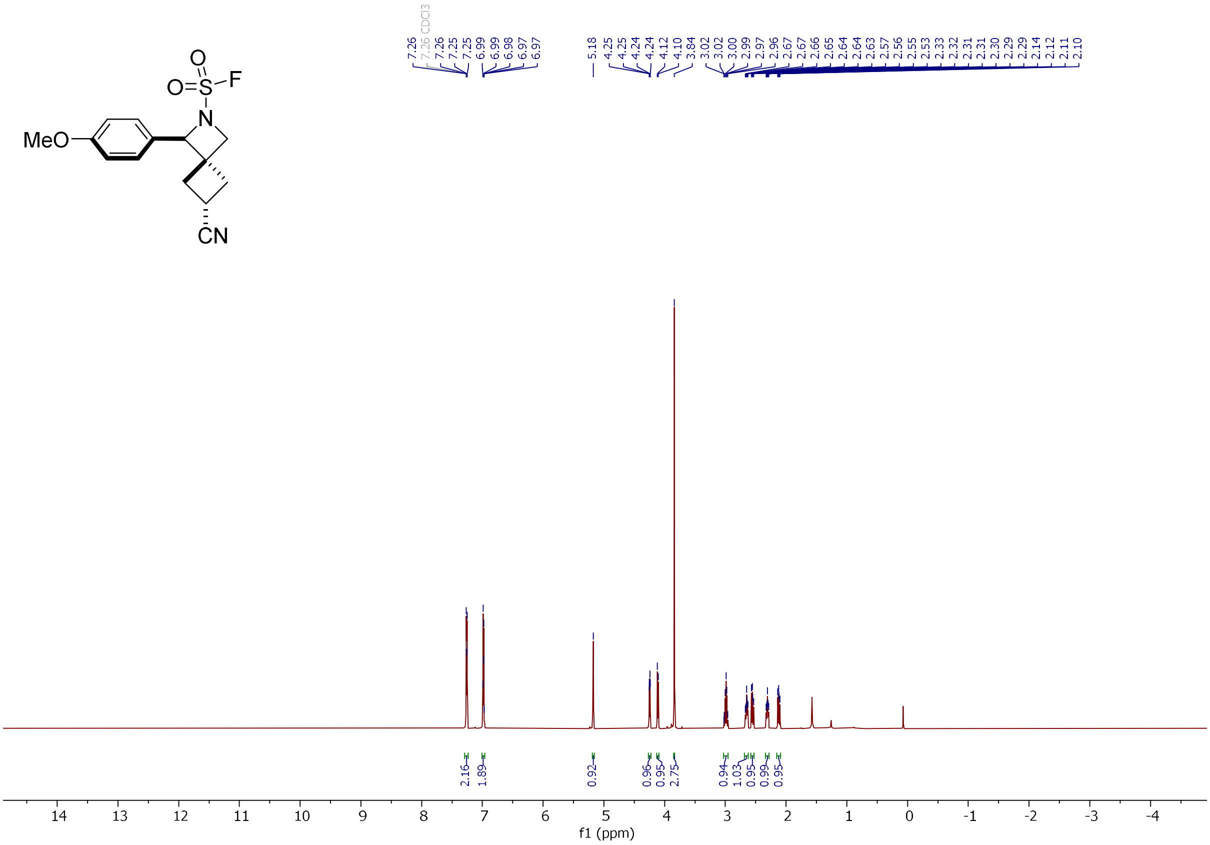

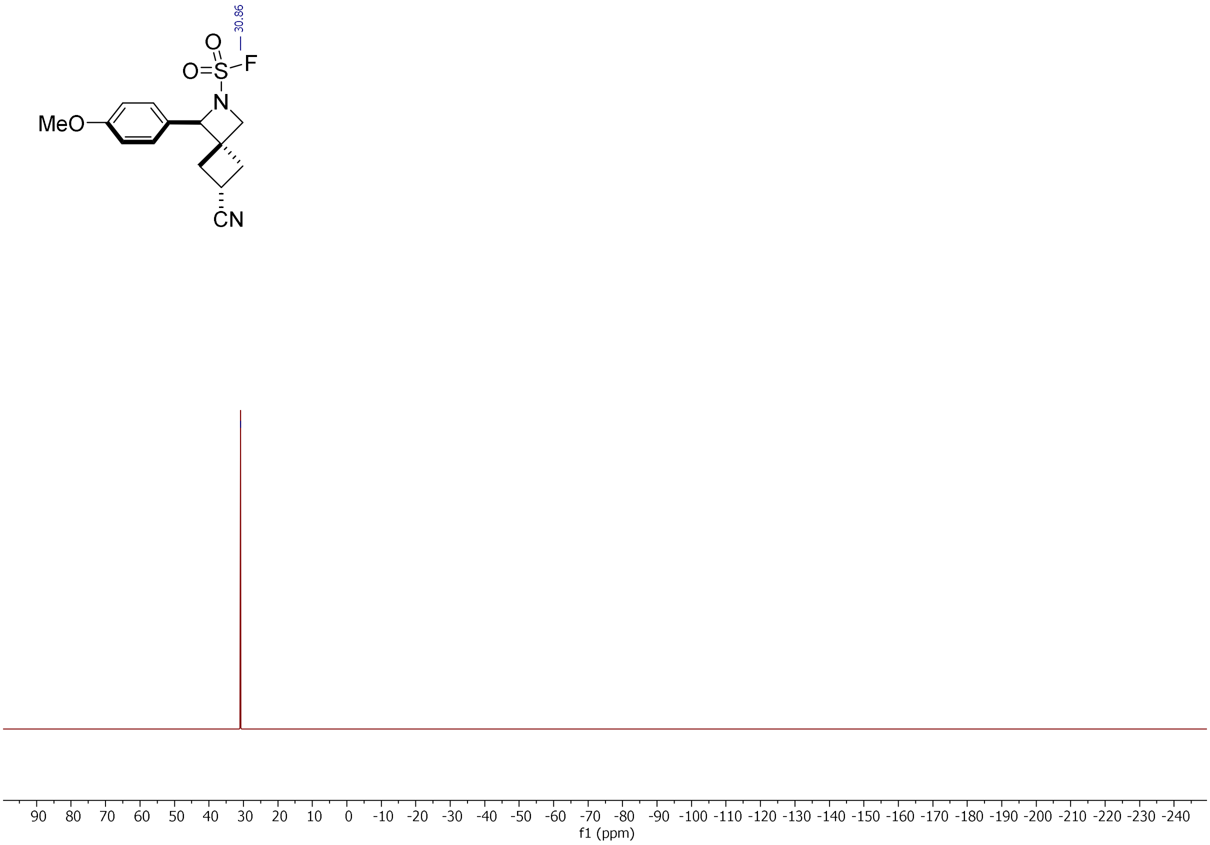

(1*R**,4*r**,6*R**)-6-Cyano-1-(4-bromophenyl)-2-azaspiro[3.3]heptane-2-sulfonyl fluoride (4g)

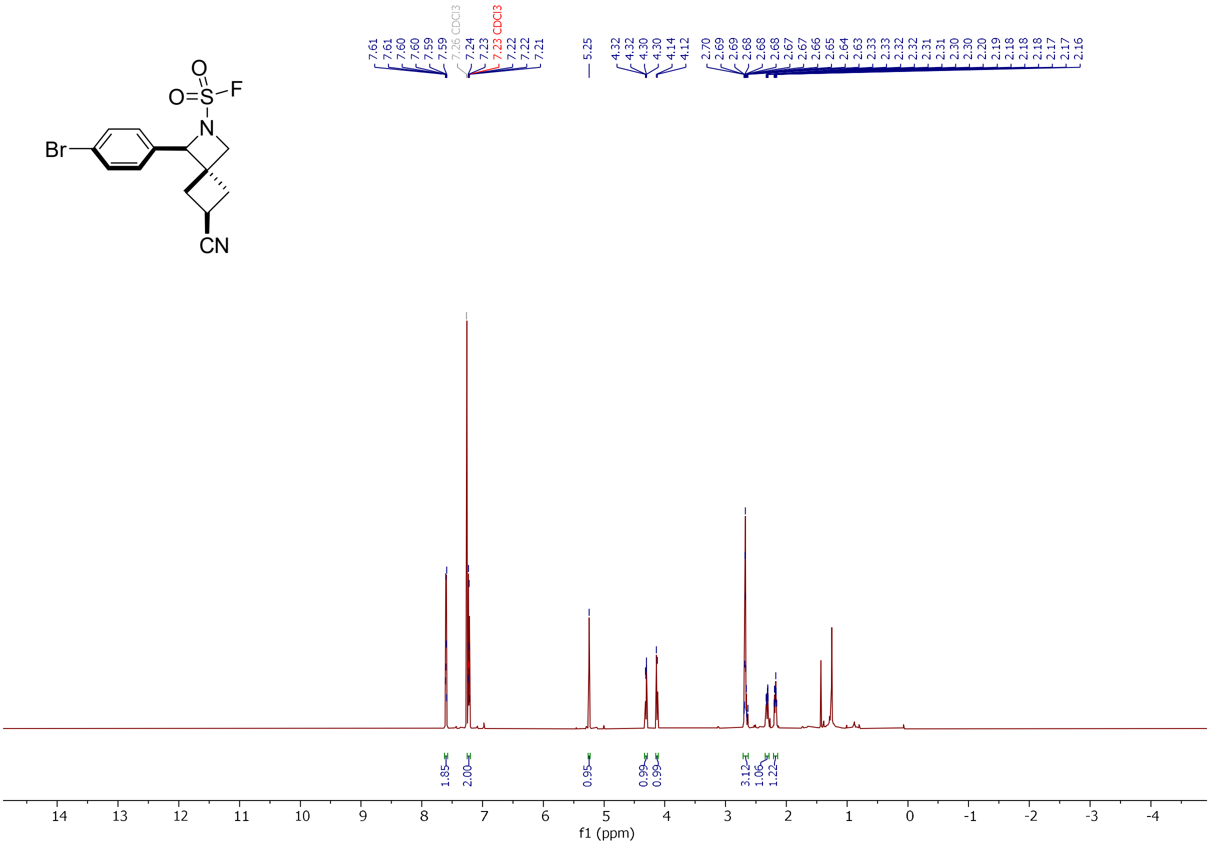

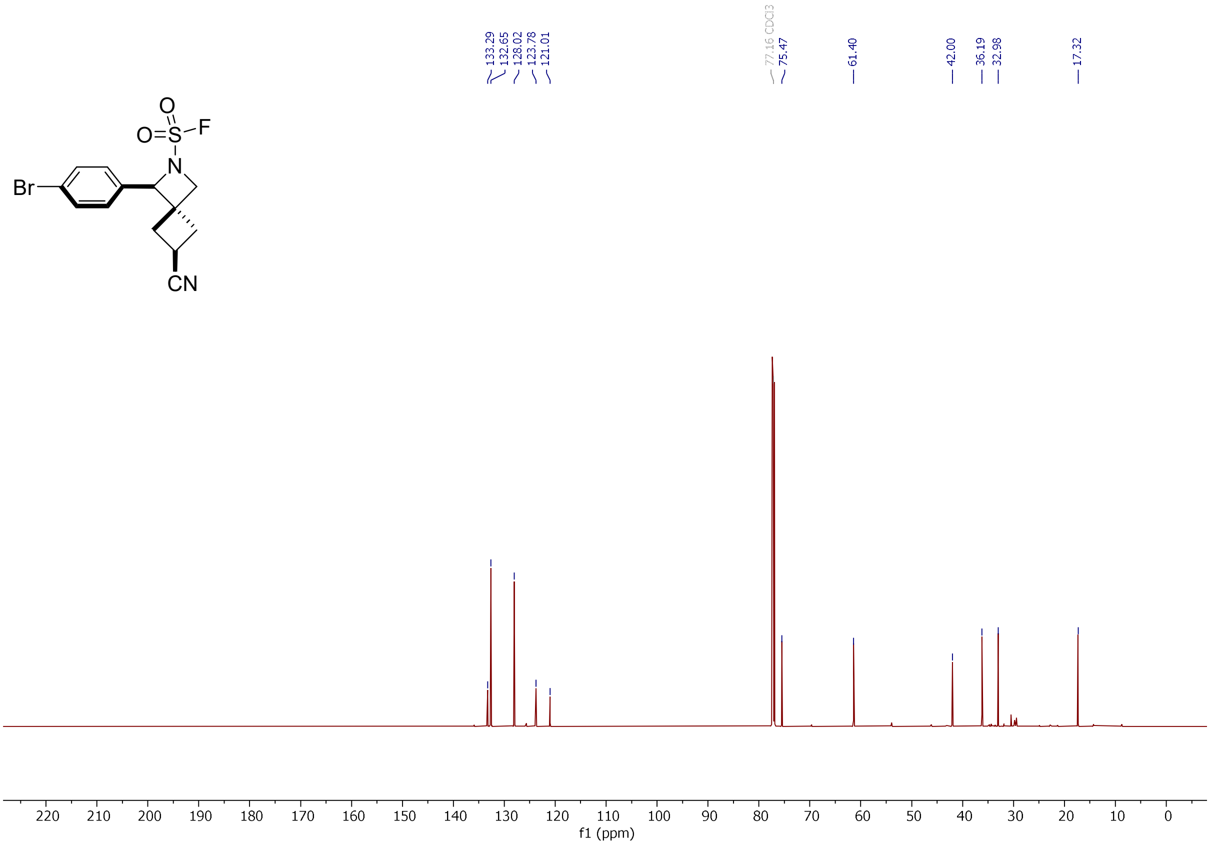
133.29
132.65
128.02
123.78
121.01
77.16 CDCl3
75.47
61.40
42.00
36.19
32.98
17.32
Br
O
O=S-F
N
CN
f1 (ppm)

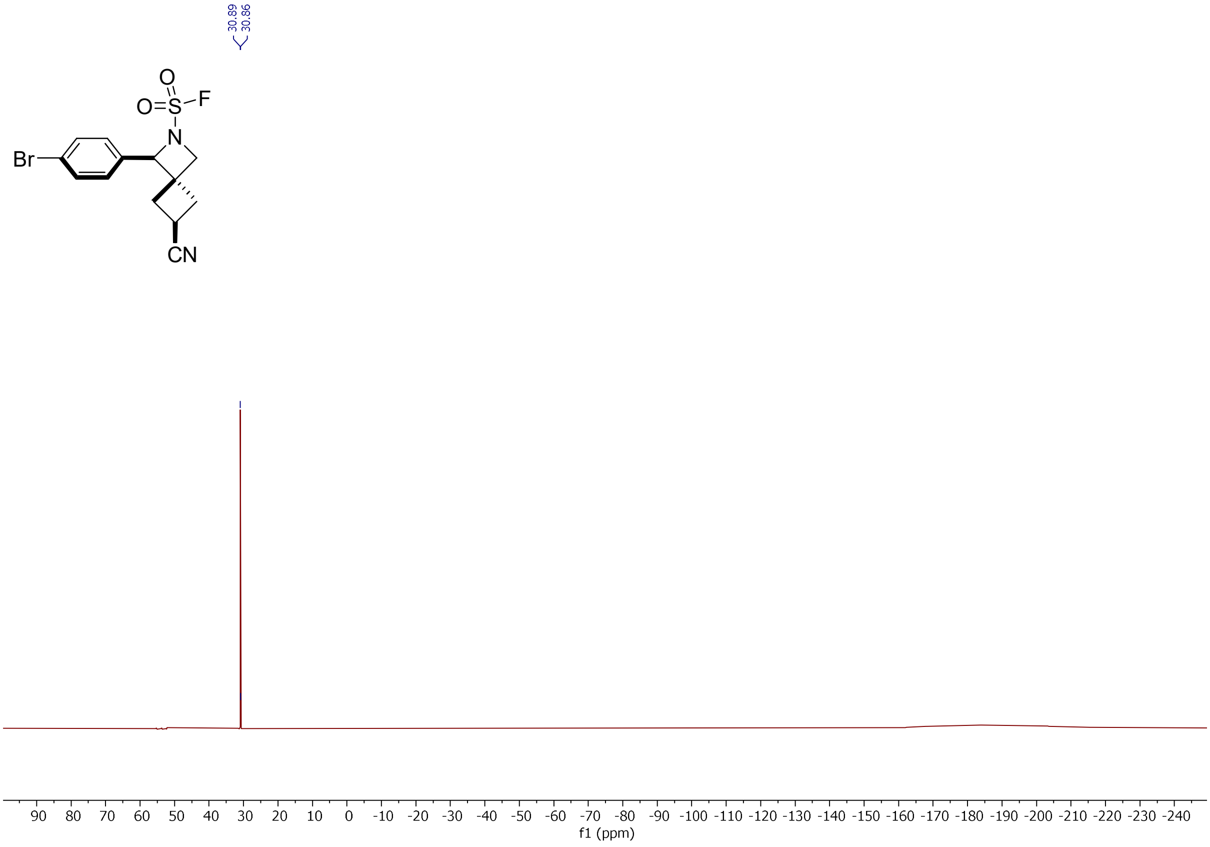
30.89
30.86
Br
O
O=S-F
N
CN
f1 (ppm)

(1R*,4s*,6S*)-6-Cyano-1-(4-bromophenyl)-2-azaspiro[3.3]heptane-2-sulfonyl fluoride (4g')

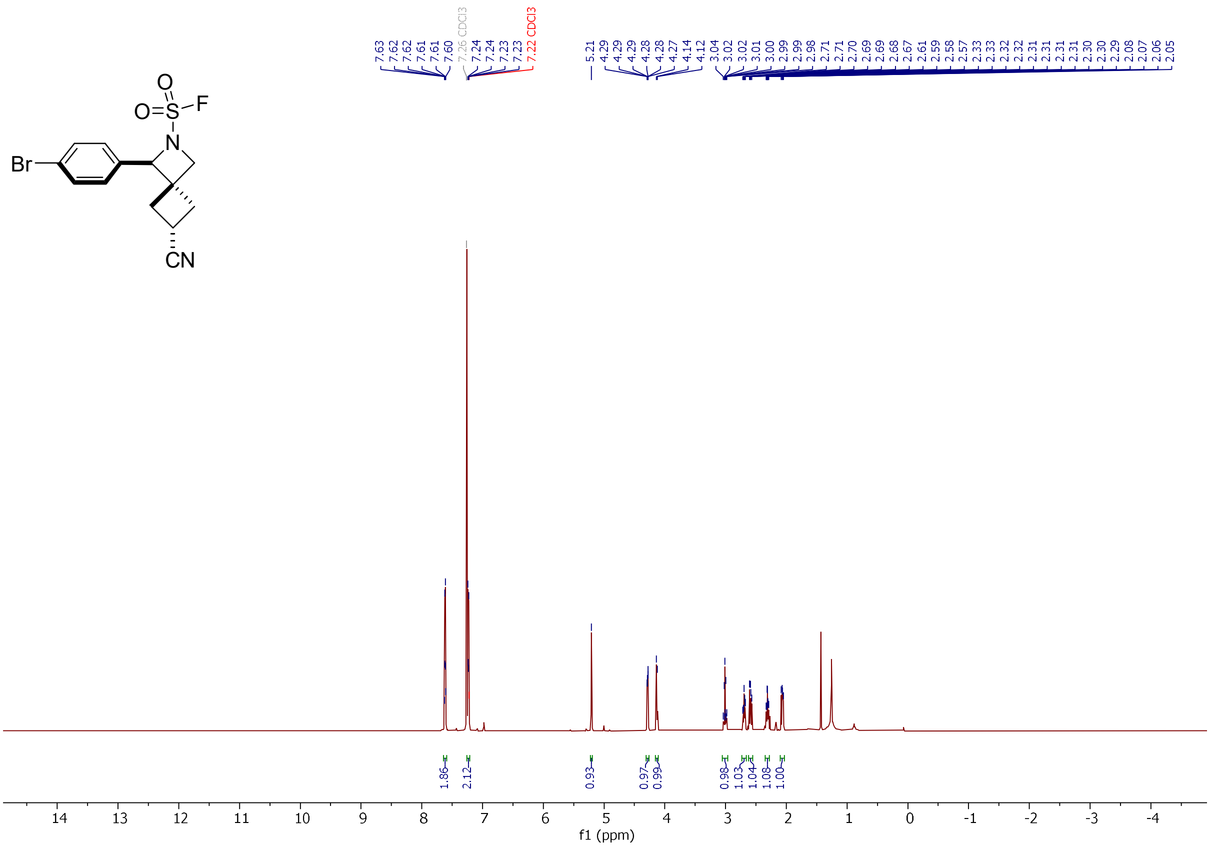

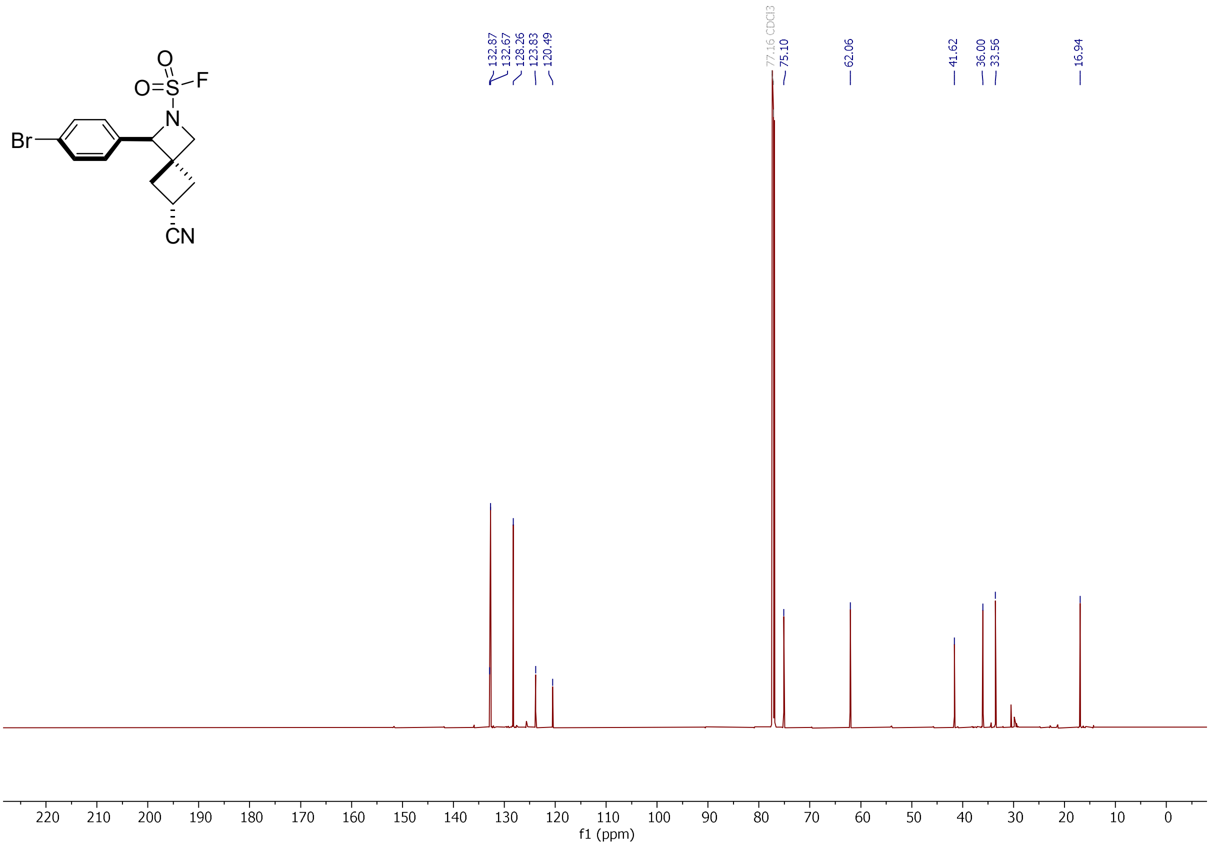

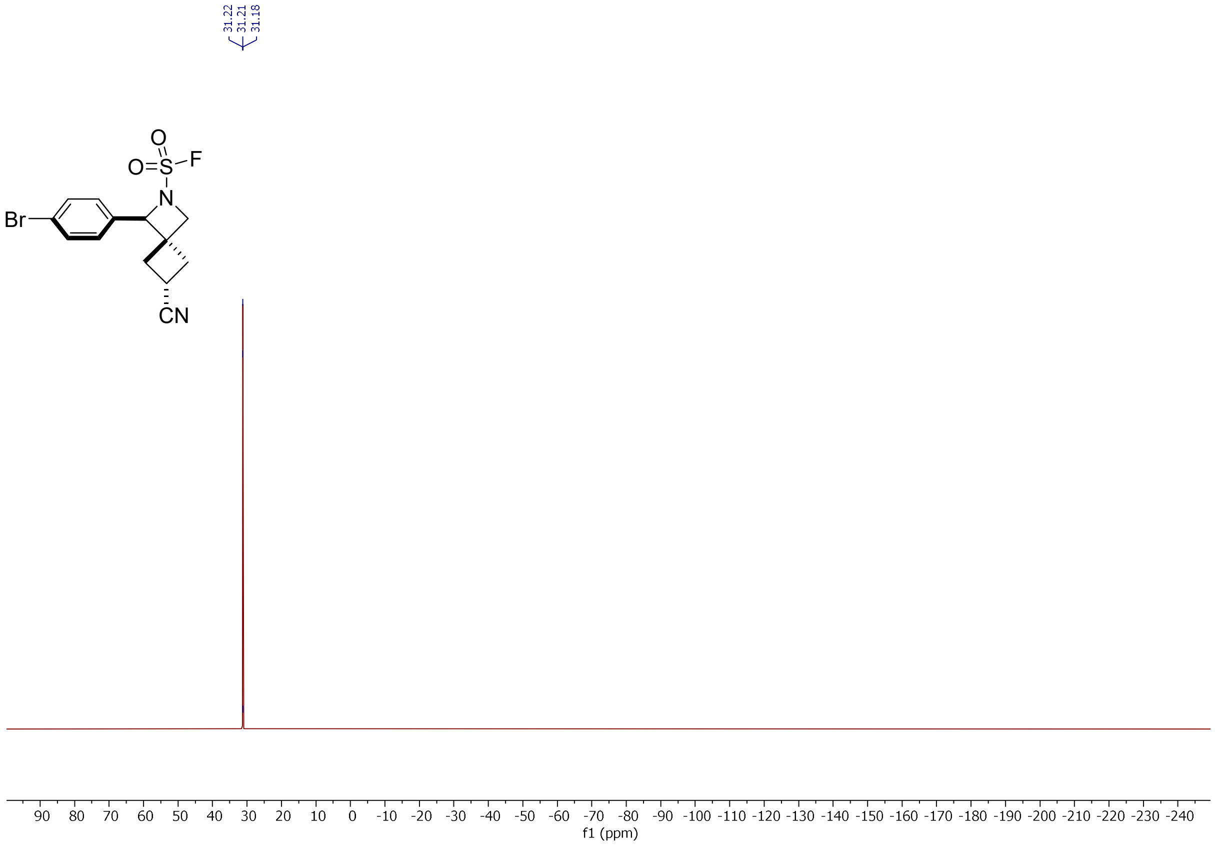
31.22
31.21
31.18
O
O=S-F
N
Br
CN
90 80 70 60 50 40 30 20 10 0 -10 -20 -30 -40 -50 -60 -70 -80 -90 -100 -110 -120 -130 -140 -150 -160 -170 -180 -190 -200 -210 -220 -230 -240
f1 (ppm)

(2*R**,*3r**,3a'*R**,6a'*S**)-2-(4-Methoxyphenyl)tetrahydro-1'H,3'H-dispiro[azetidine-3,2'-pentalene-5',2''-[1,3]dioxolane]-1-sulfonyl fluoride (4h) and (2*S**,*3r**,3a'*R**,6a'*S**)-2-(4-Methoxyphenyl)tetrahydro-1'H,3'H-dispiro[azetidine-3,2'-pentalene-5',2''-[1,3]dioxolane]-1-sulfonyl fluoride (4h')

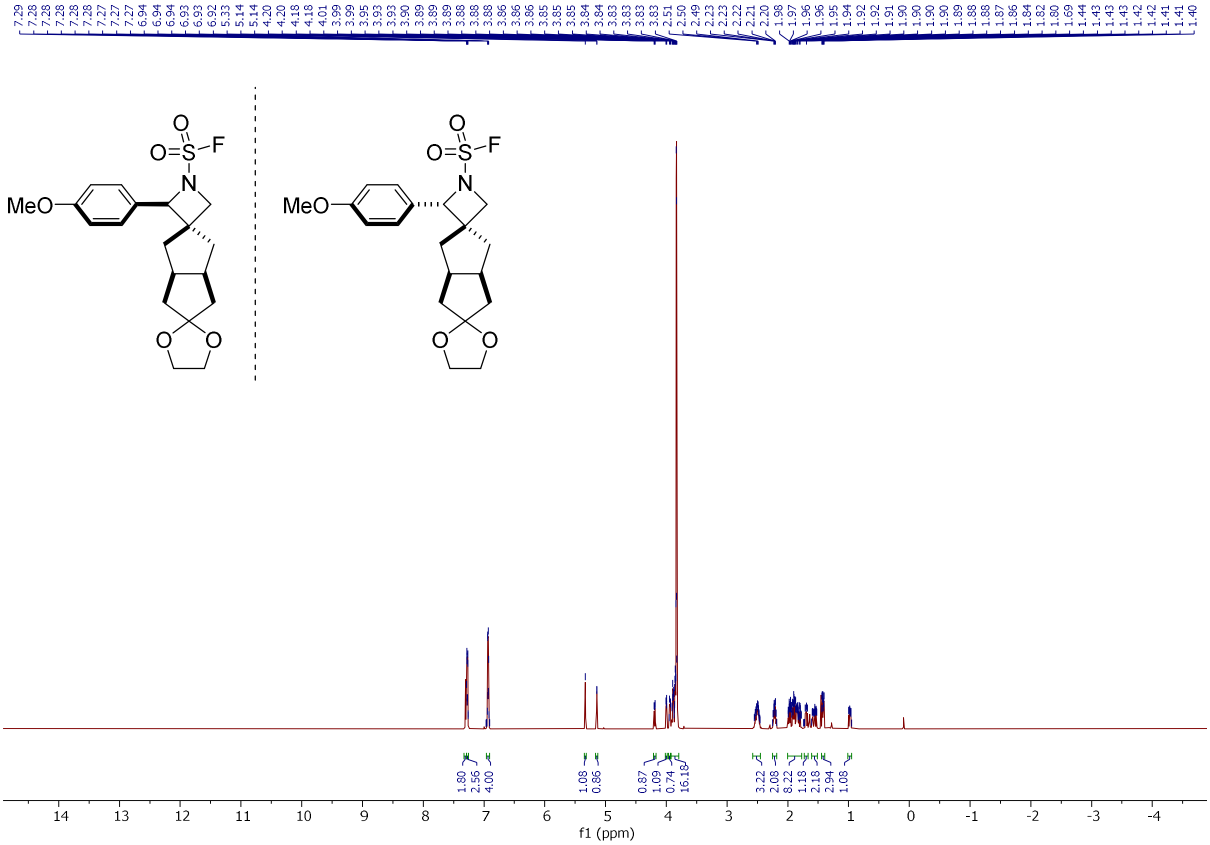

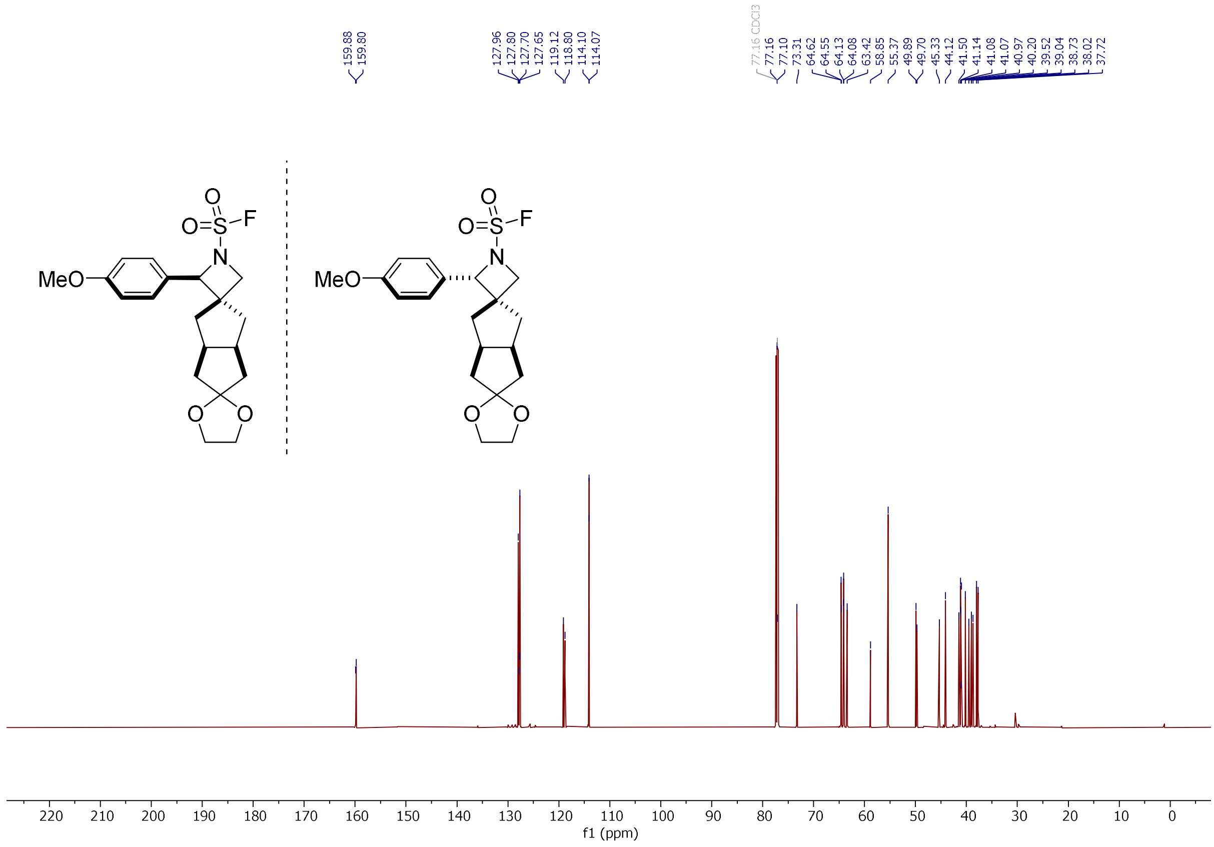

159.88
159.80
127.96
127.80
127.70
127.65
119.12
118.80
114.10
114.07
77.16 CDCl3
77.16
77.10
73.31
64.62
64.55
64.13
64.08
63.42
58.85
55.37
49.89
49.70
45.33
44.12
41.50
41.14
41.08
41.07
40.97
40.20
39.52
39.04
38.73
38.02
37.72
MeO
O
O=S–F
N
O
O
f1 (ppm)

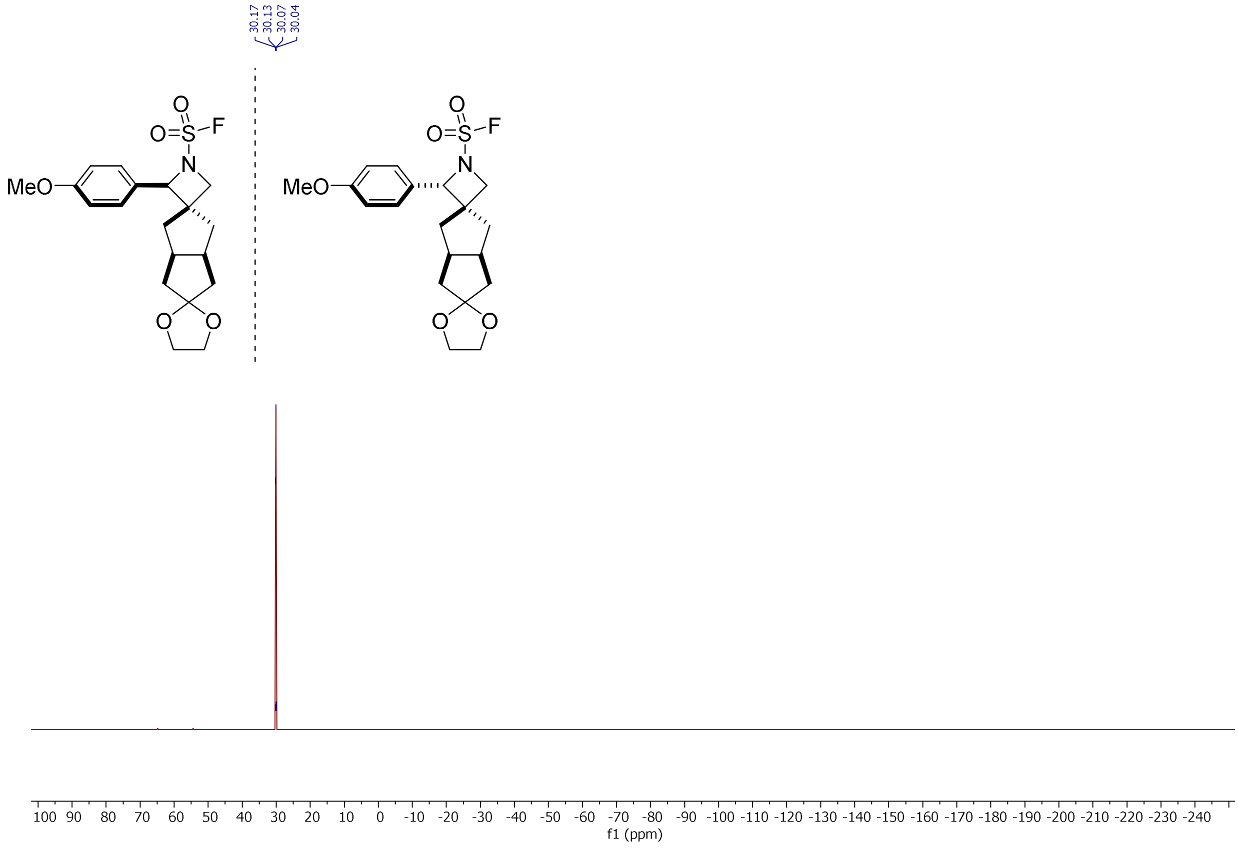

30.17
30.13
30.07
30.04
MeO
O
O=S–F
N
O
O
f1 (ppm)

1-(4-Methoxyphenyl)-2-azaspiro[3.5]nonane-2-sulfonyl fluoride (4i)

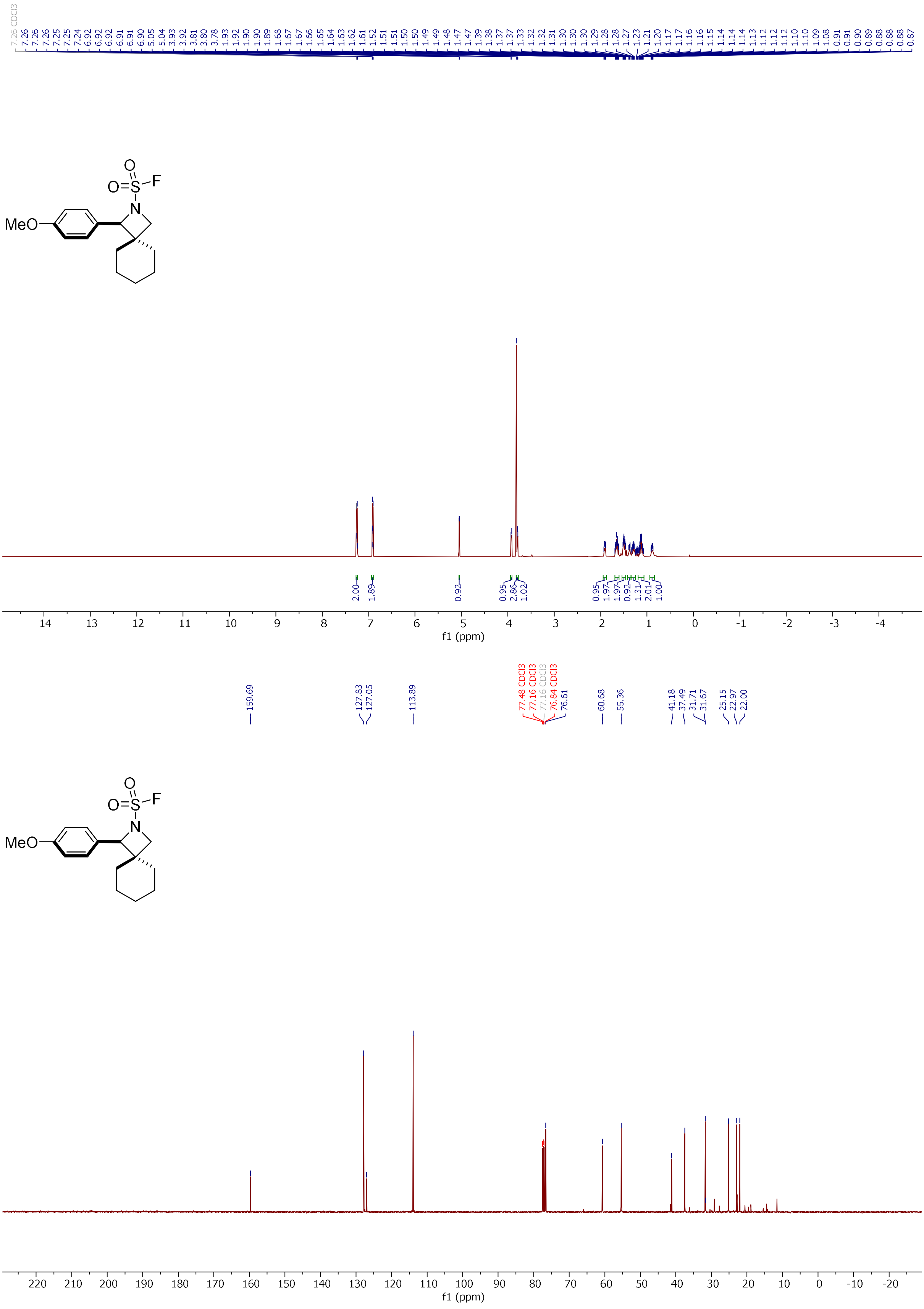

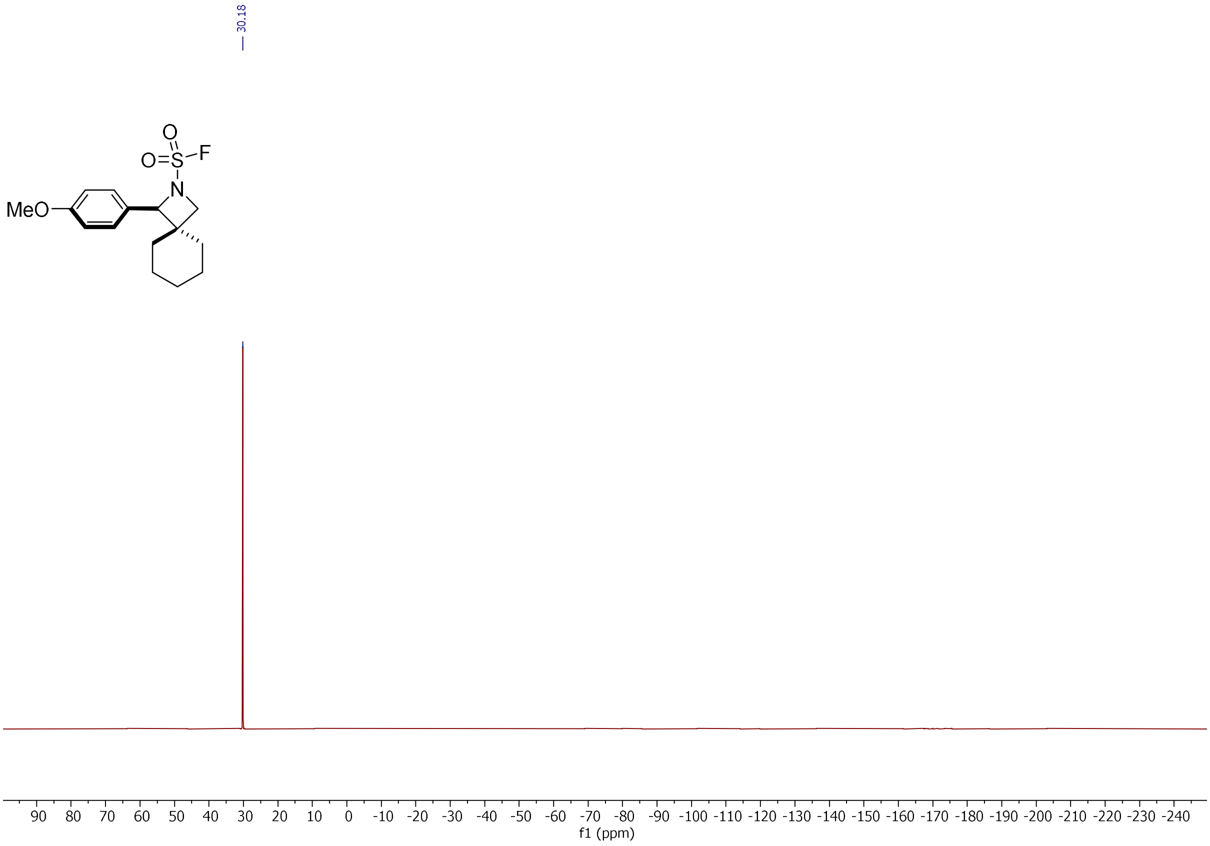
30.18
MeO
O
O=S-F
N
90 80 70 60 50 40 30 20 10 0 -10 -20 -30 -40 -50 -60 -70 -80 -90 -100 -110 -120 -130 -140 -150 -160 -170 -180 -190 -200 -210 -220 -230 -240
f1 (ppm)

1-(4-Methoxyphenyl)-7-(2,2,2-trifluoroacetyl)-2,7-diazaspiro[3.5]nonane-2-sulfonyl fluoride (4j)

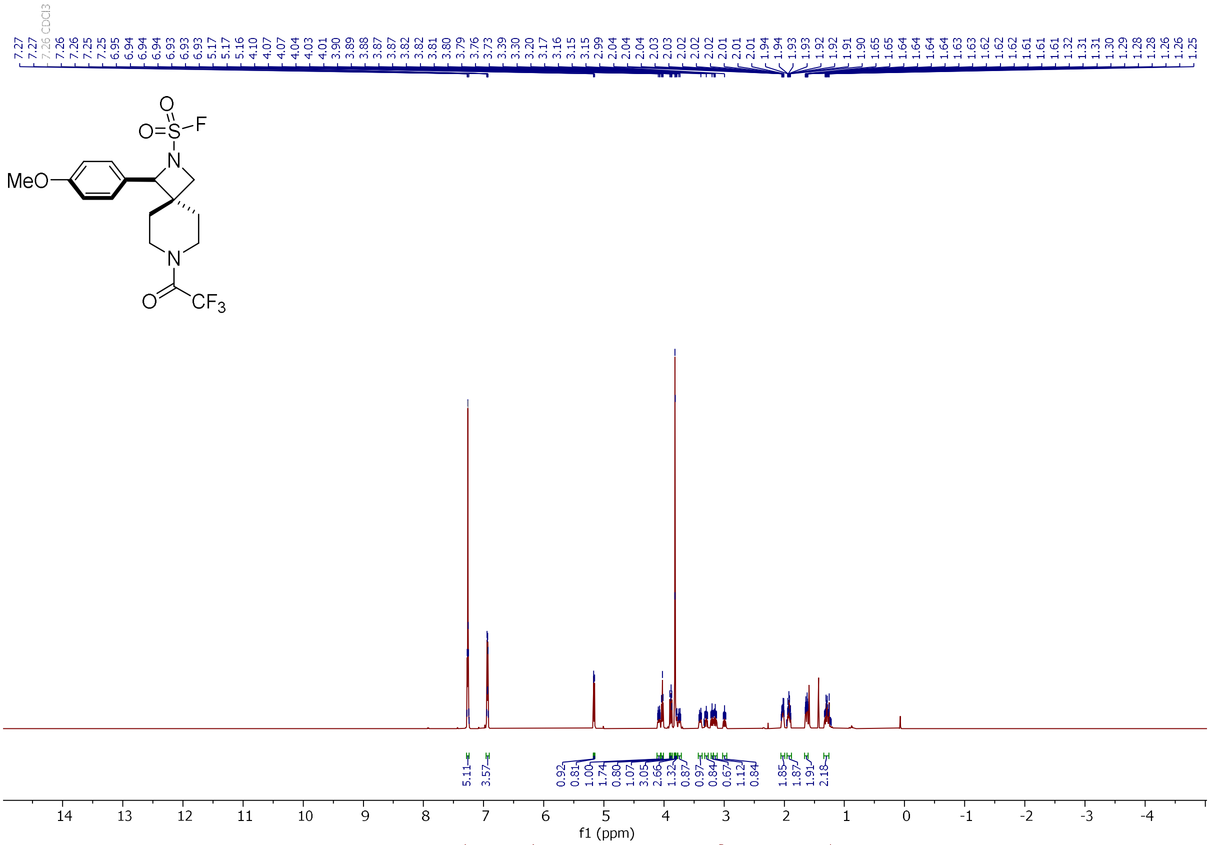

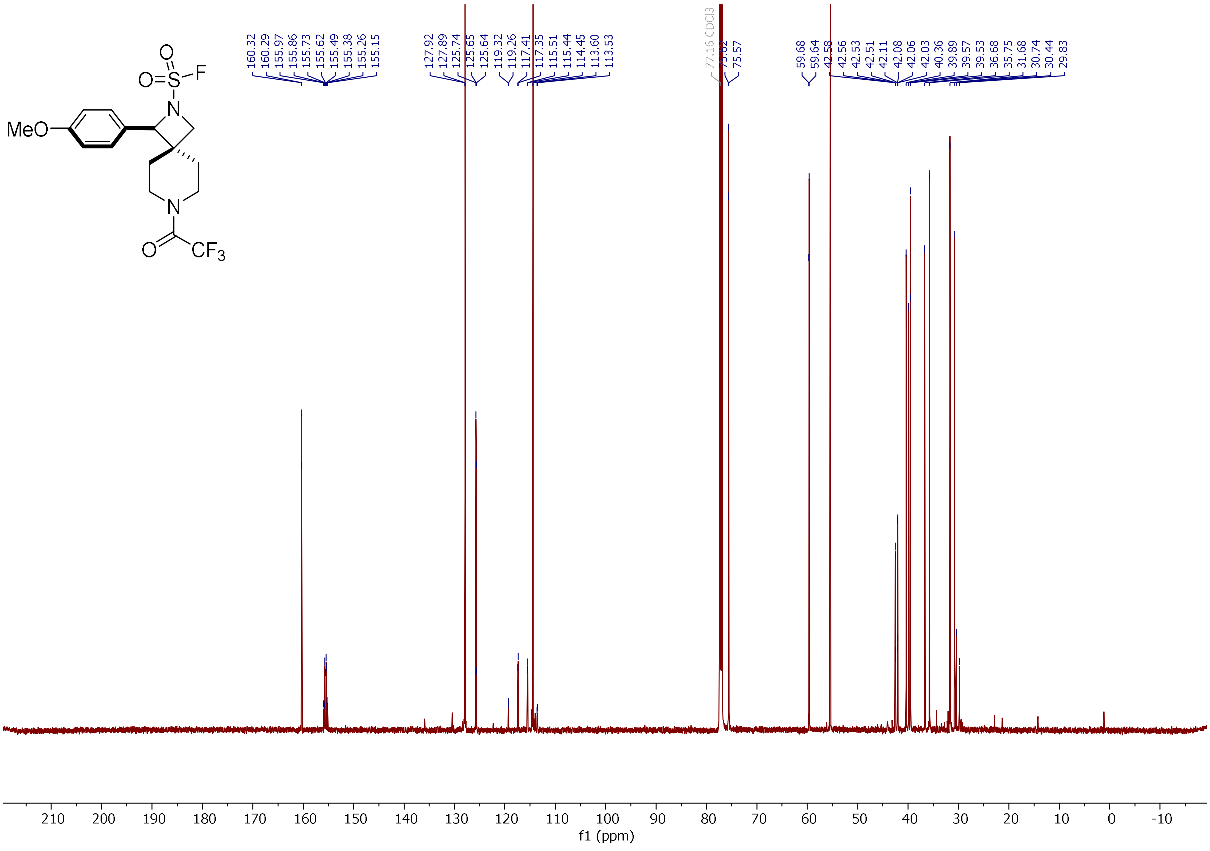

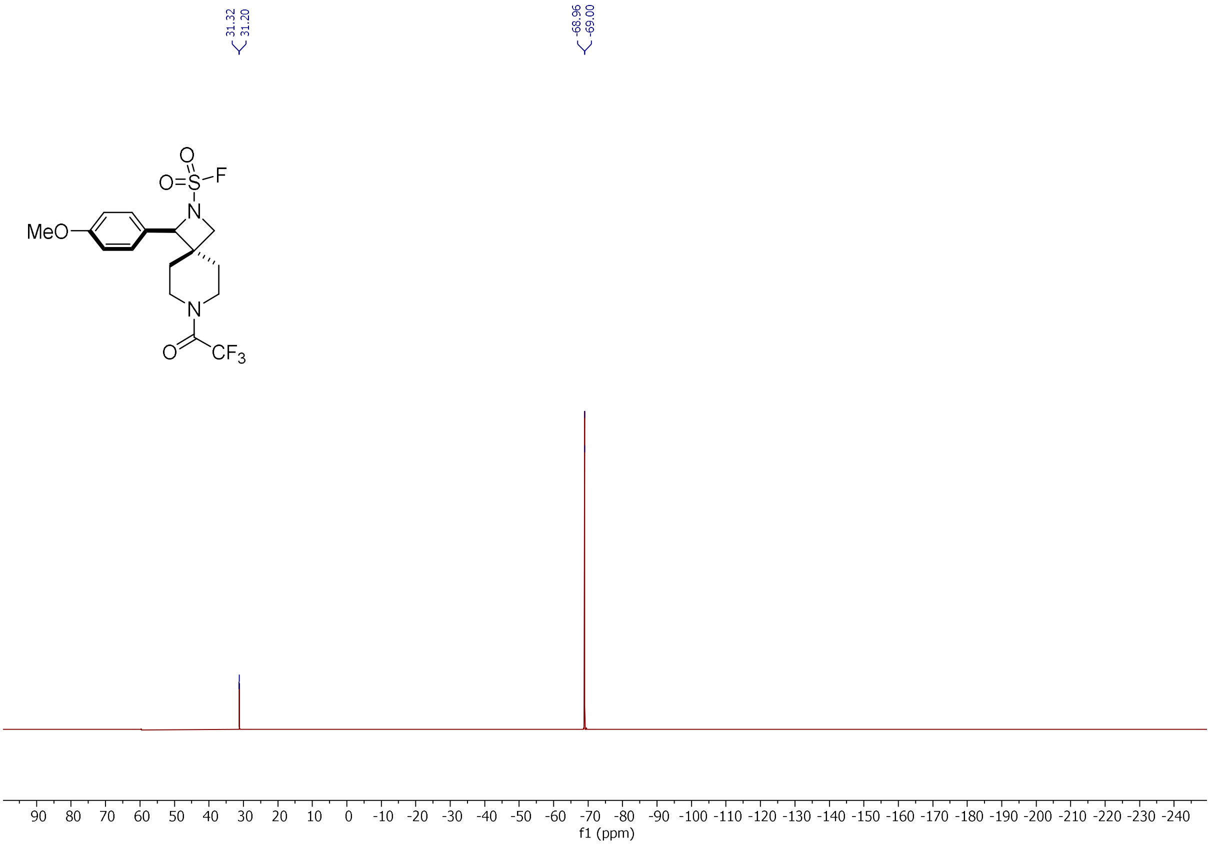

1-(4-Methoxyphenyl)-7-thia-2-azaspiro[3.5]nonane-2-sulfonyl fluoride 7,7-dioxide (4k)

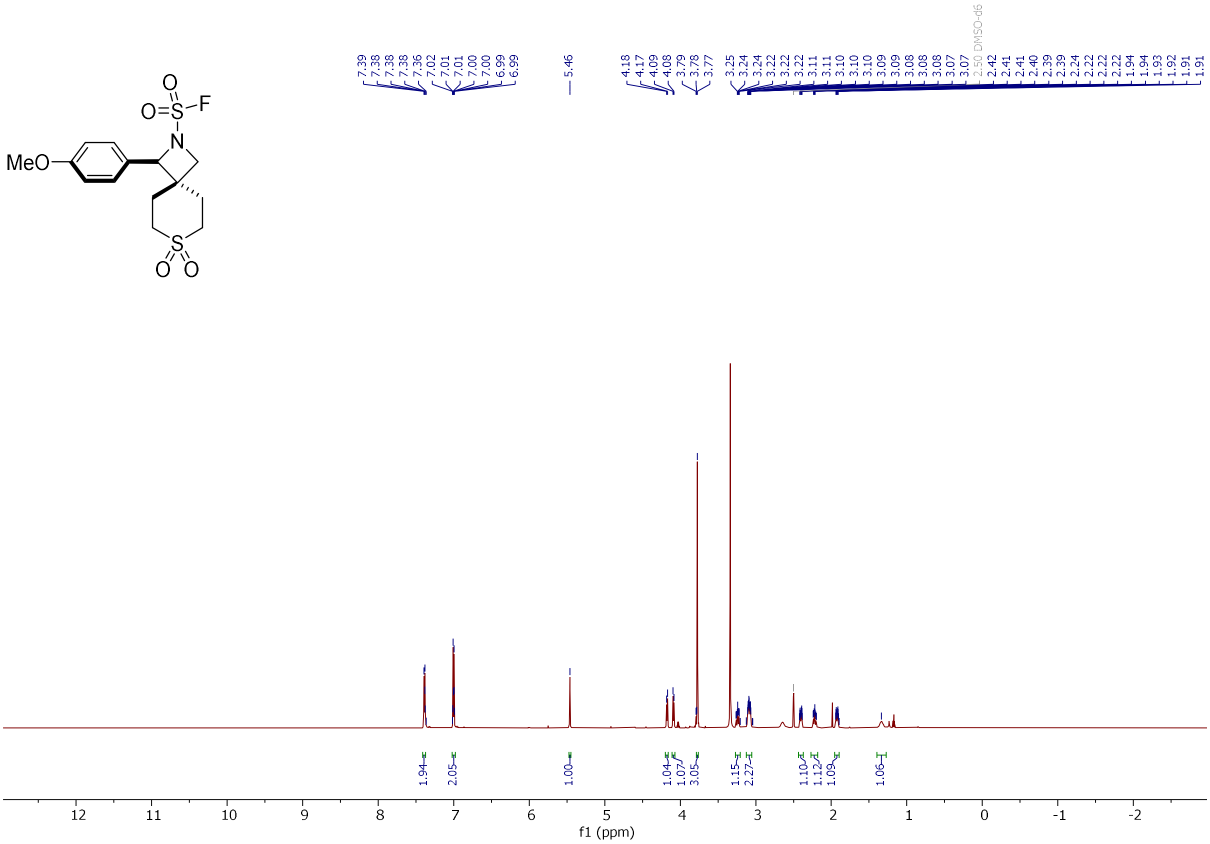

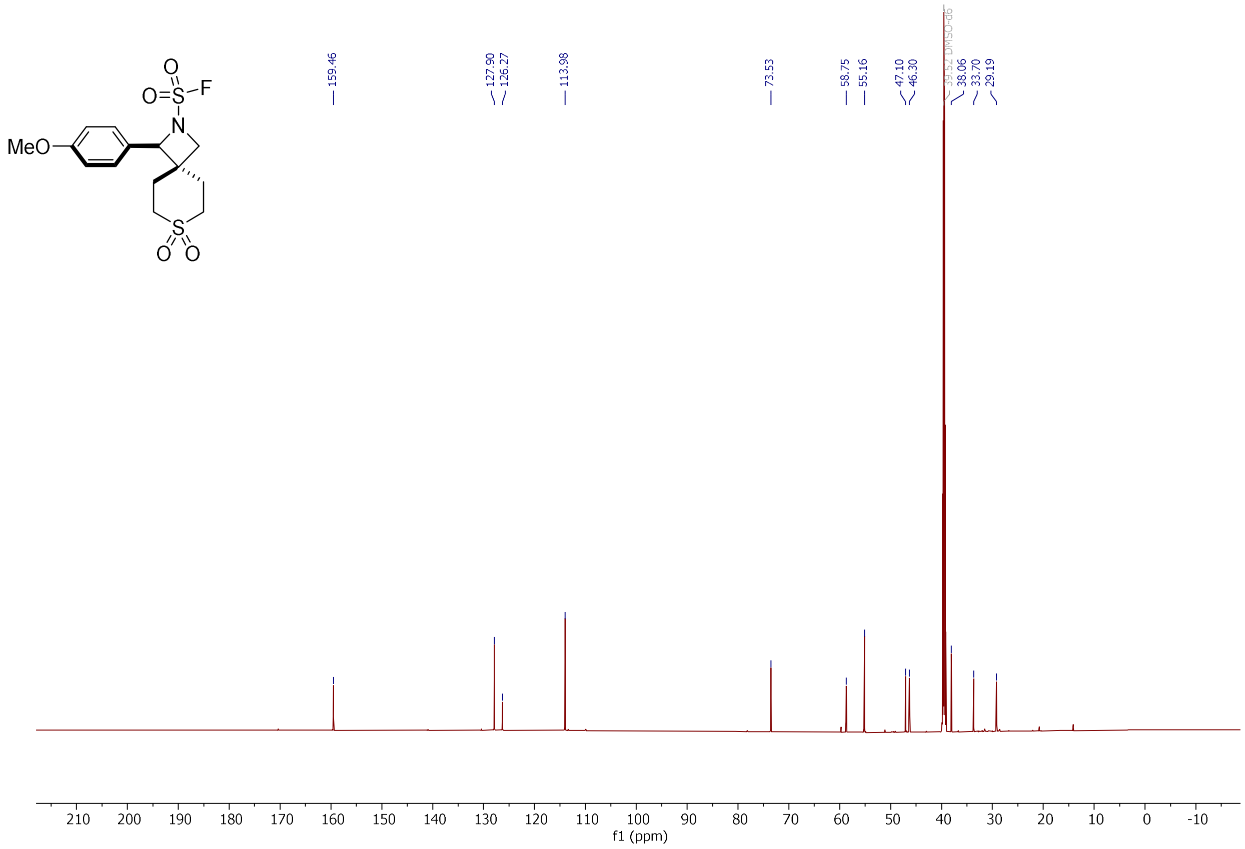
159.46
127.90
126.27
113.98
73.53
58.75
55.16
47.10
46.30
39.52 DMSO-d6
38.06
33.70
29.19
MeO
O
O=S-F
N
S
O O
210 200 190 180 170 160 150 140 130 120 110 100 90 80 70 60 50 40 30 20 10 0 -10
f1 (ppm)

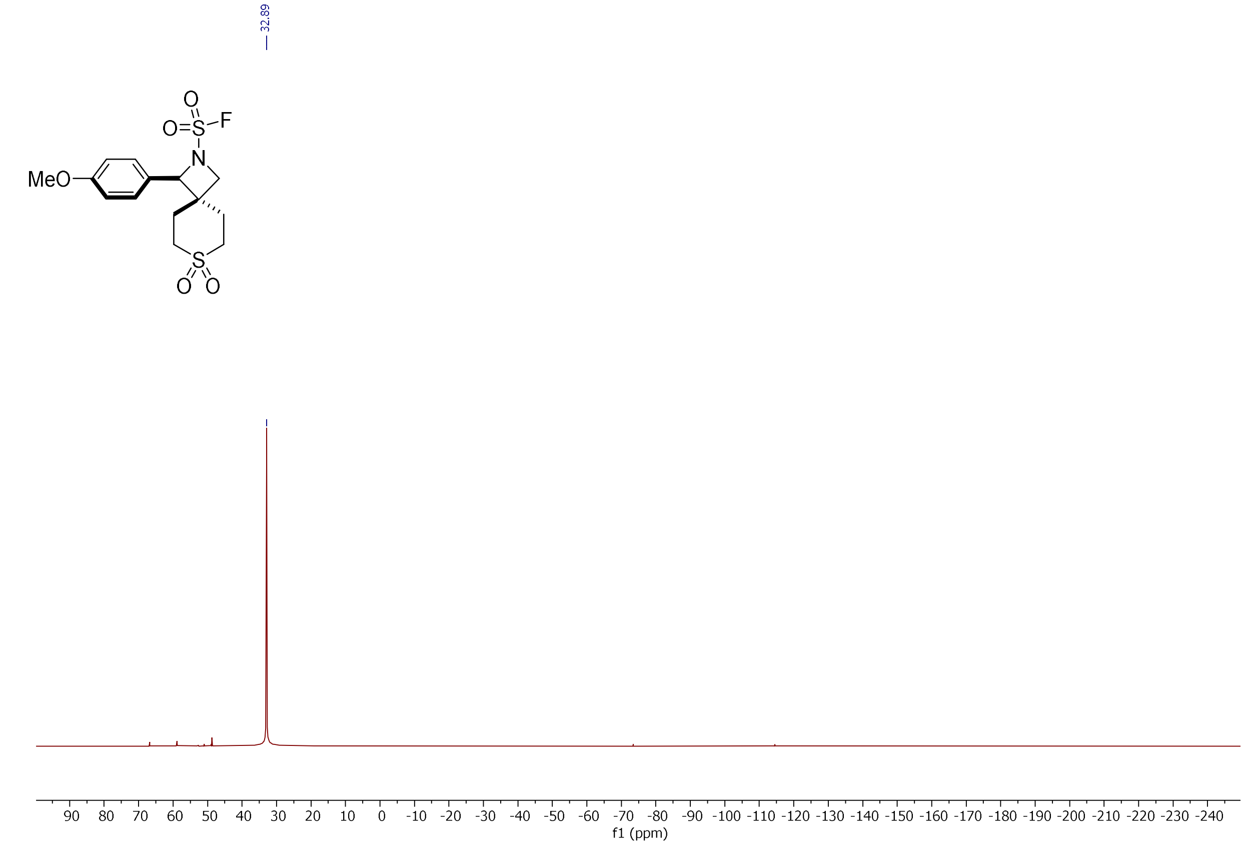
32.89
MeO
O
O=S-F
N
S
O O
90 80 70 60 50 40 30 20 10 0 -10 -20 -30 -40 -50 -60 -70 -80 -90 -100 -110 -120 -130 -140 -150 -160 -170 -180 -190 -200 -210 -220 -230 -240
f1 (ppm)

(1*R**,5*S**,7*S**)-7-(4-Methoxyphenyl)-6-azabicyclo[3.2.0]heptane-6-sulfonyl fluoride (4l)

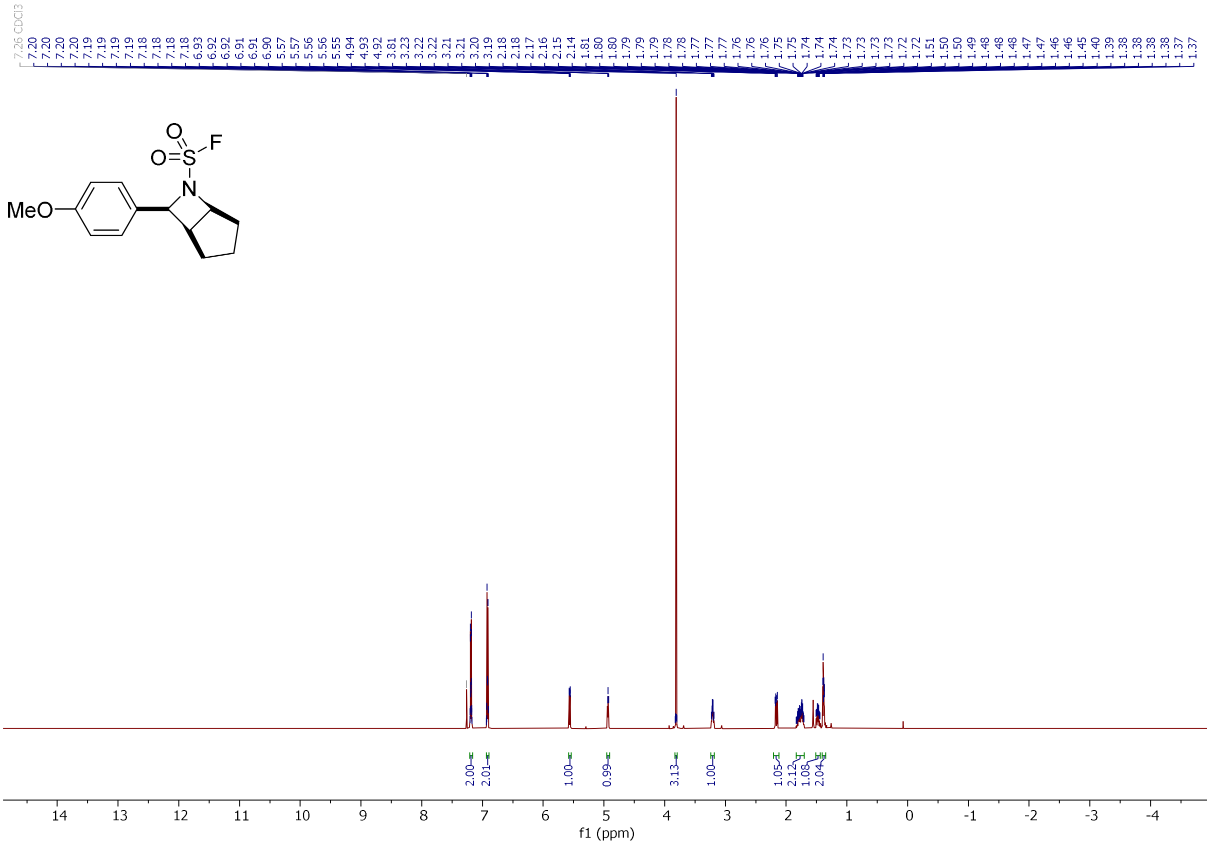

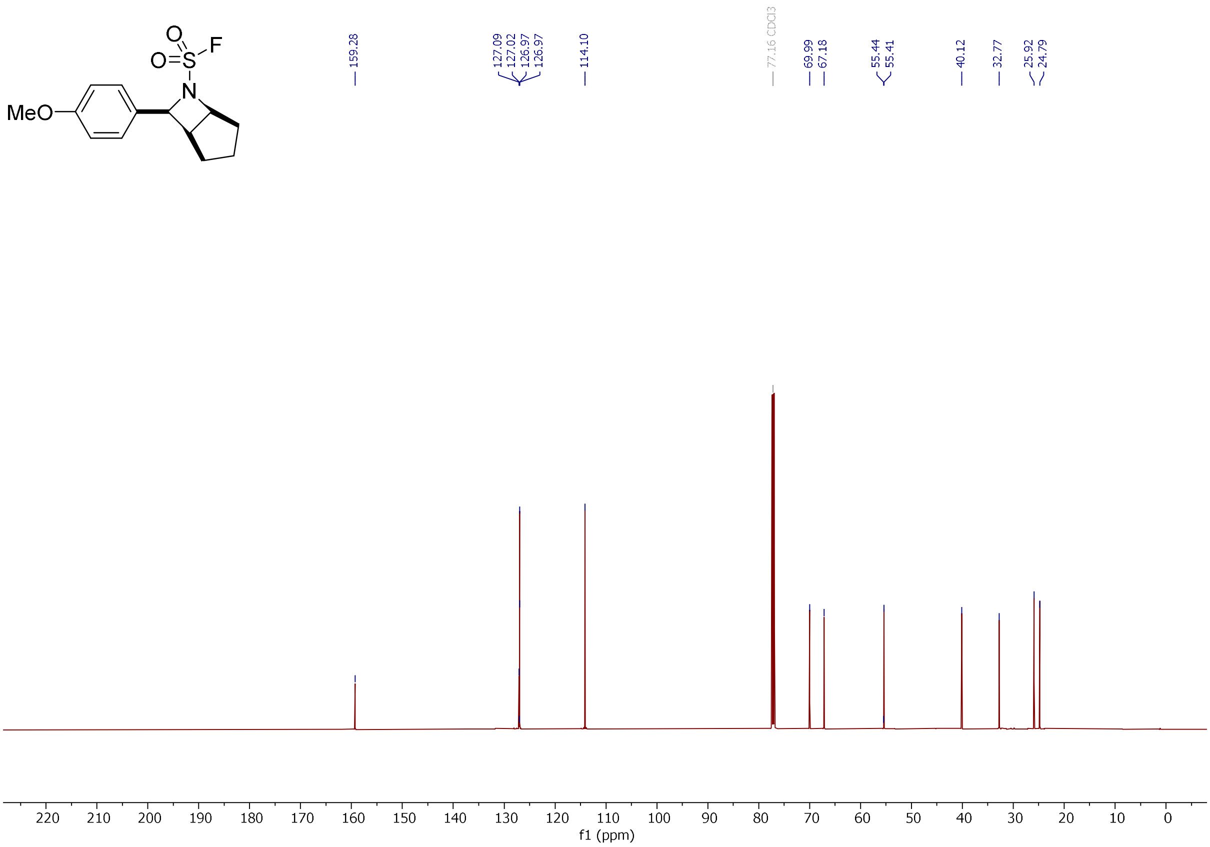

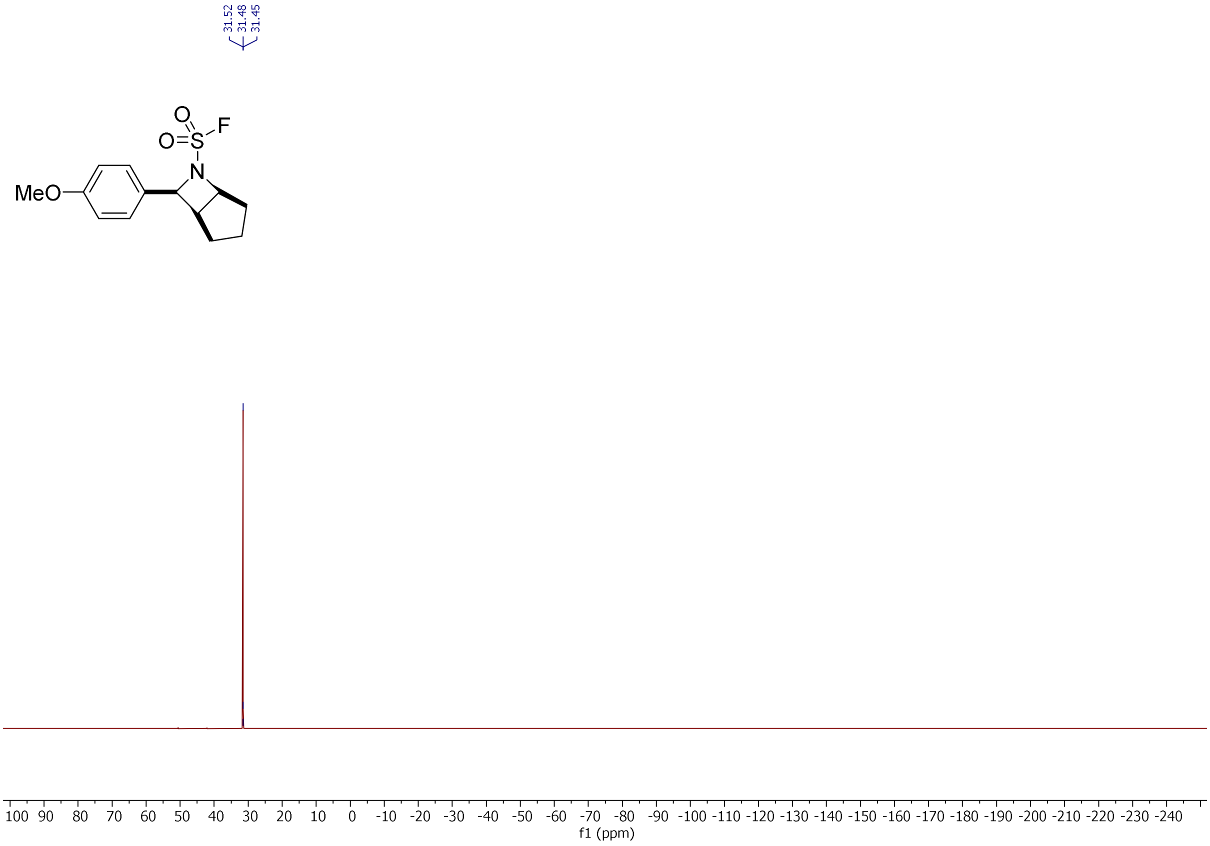

(1R*,5S*,7R*)-7-(4-Methoxyphenyl)-6-azabicyclo[3.2.0]heptane-6-sulfonyl fluoride (4l’)

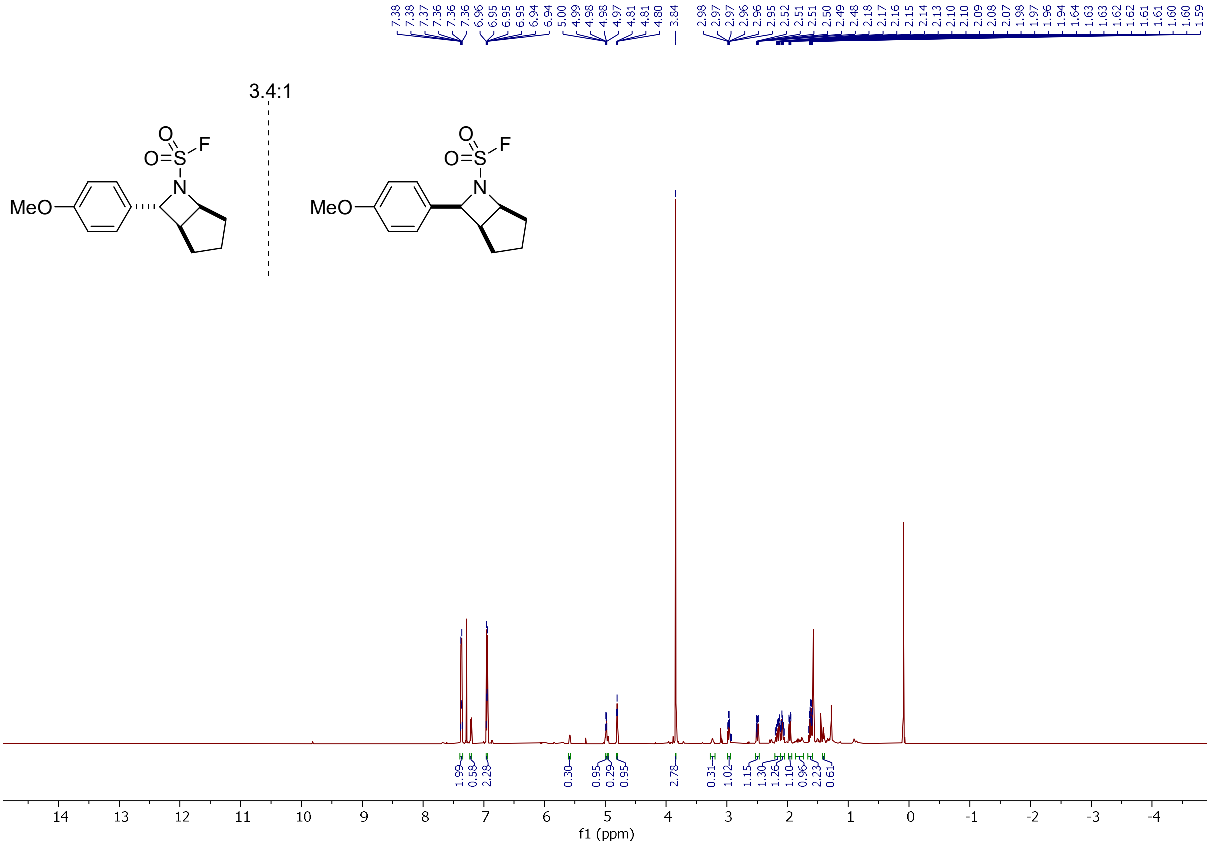

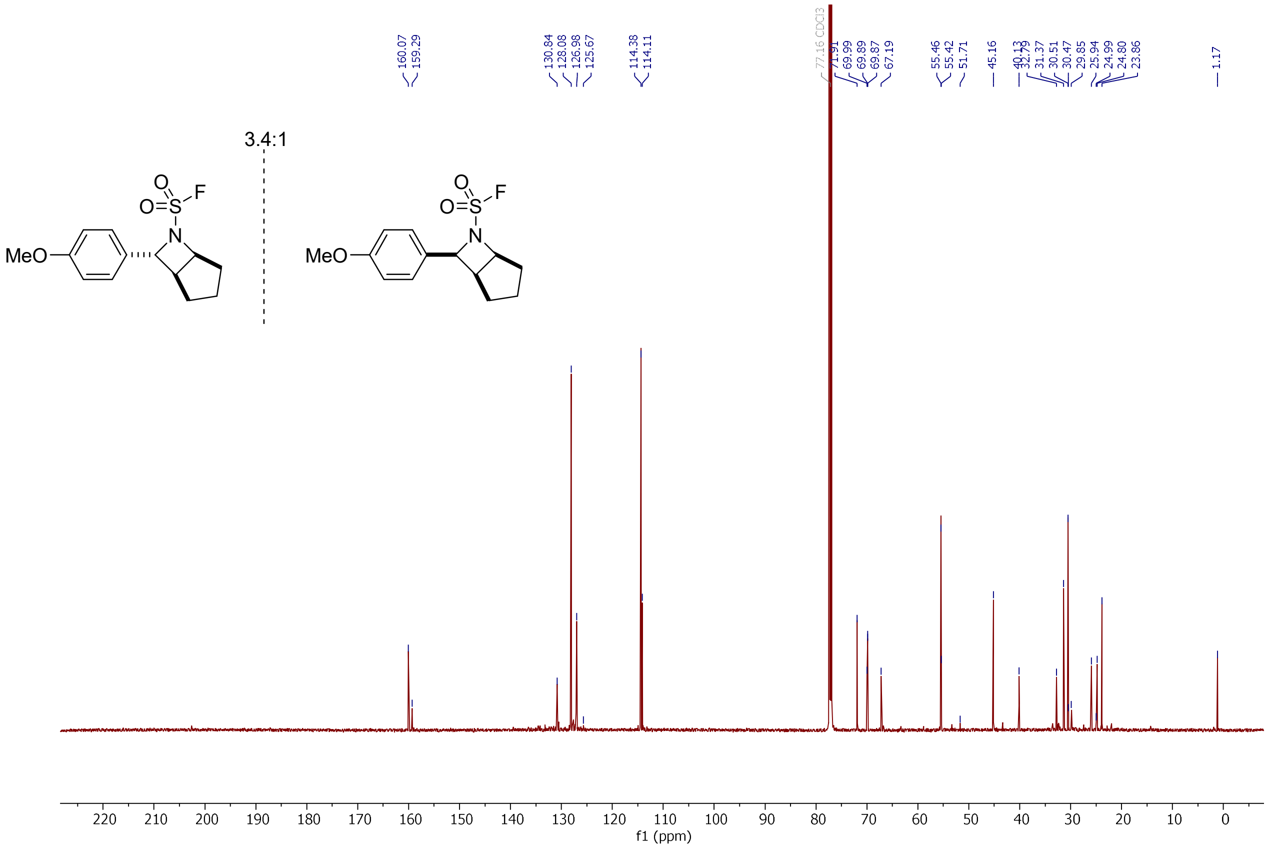
160.07
159.29
130.84
128.08
126.98
125.67
114.38
114.11
77.16 CDCl3
71.91
69.99
69.89
69.87
67.19
55.46
55.42
51.71
45.16
40.13
32.79
31.37
30.51
30.47
29.85
25.94
24.99
24.80
23.86
1.17
3.4:1
MeO
O=S
O
F
N
f1 (ppm)

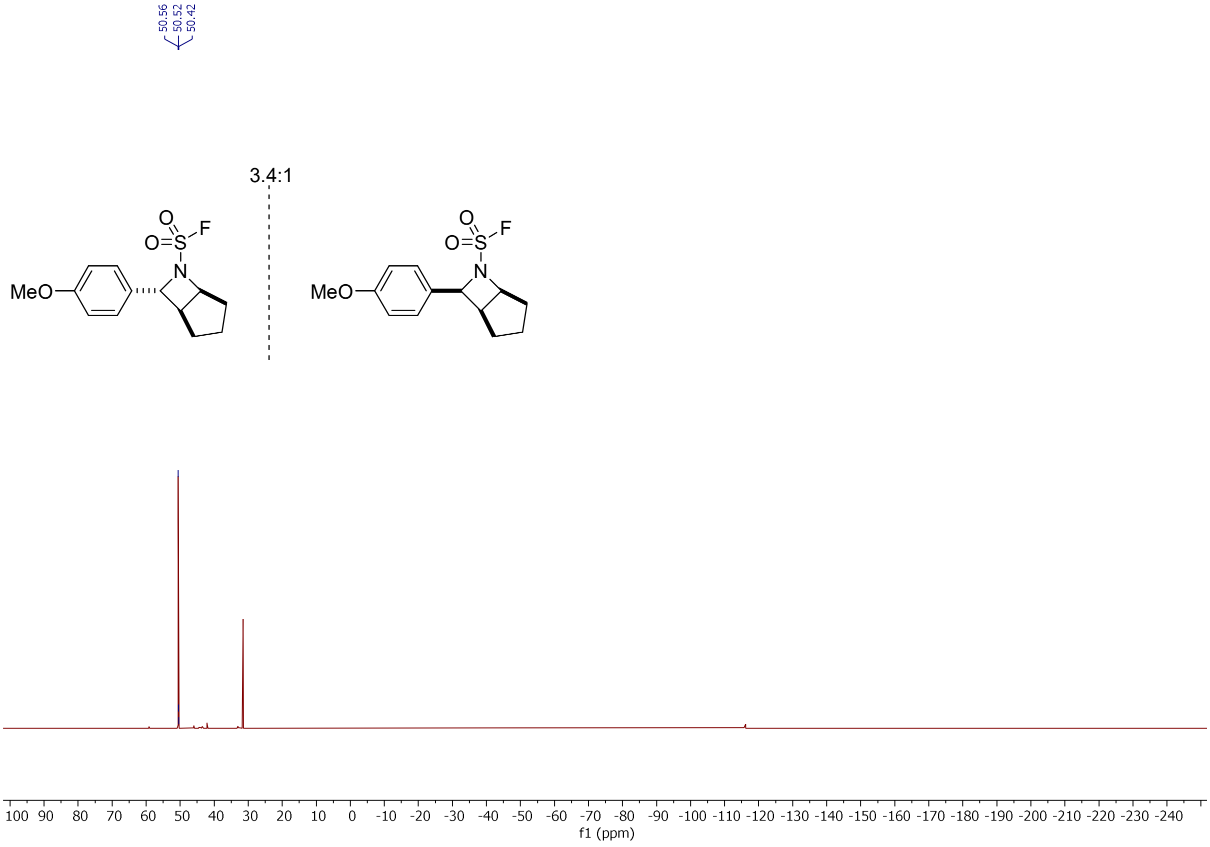
50.56
50.52
50.42
3.4:1
MeO
O=S
O
F
N
f1 (ppm)

(1*R**,6*S**,8*S**)-8-(4-Methoxyphenyl)-7-azabicyclo[4.2.0]octane-7-sulfonyl fluoride (4m)

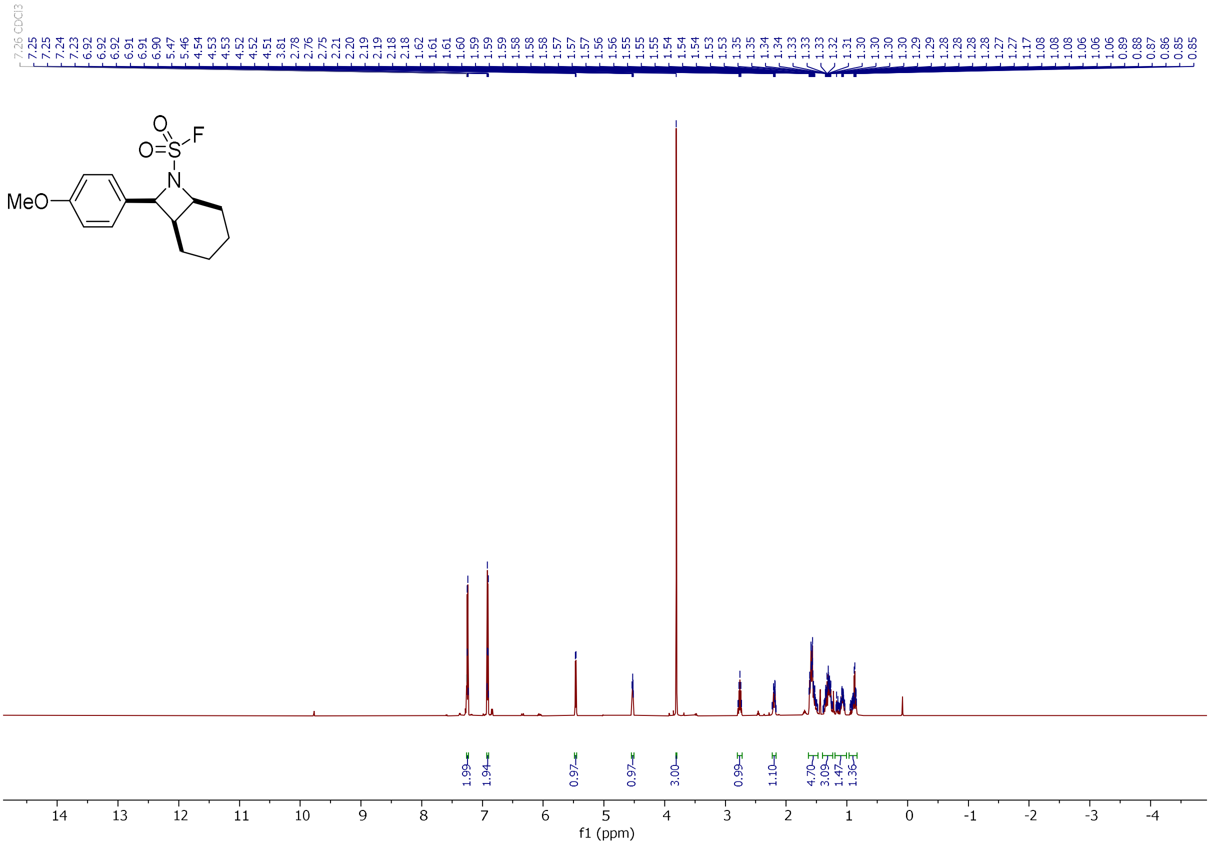

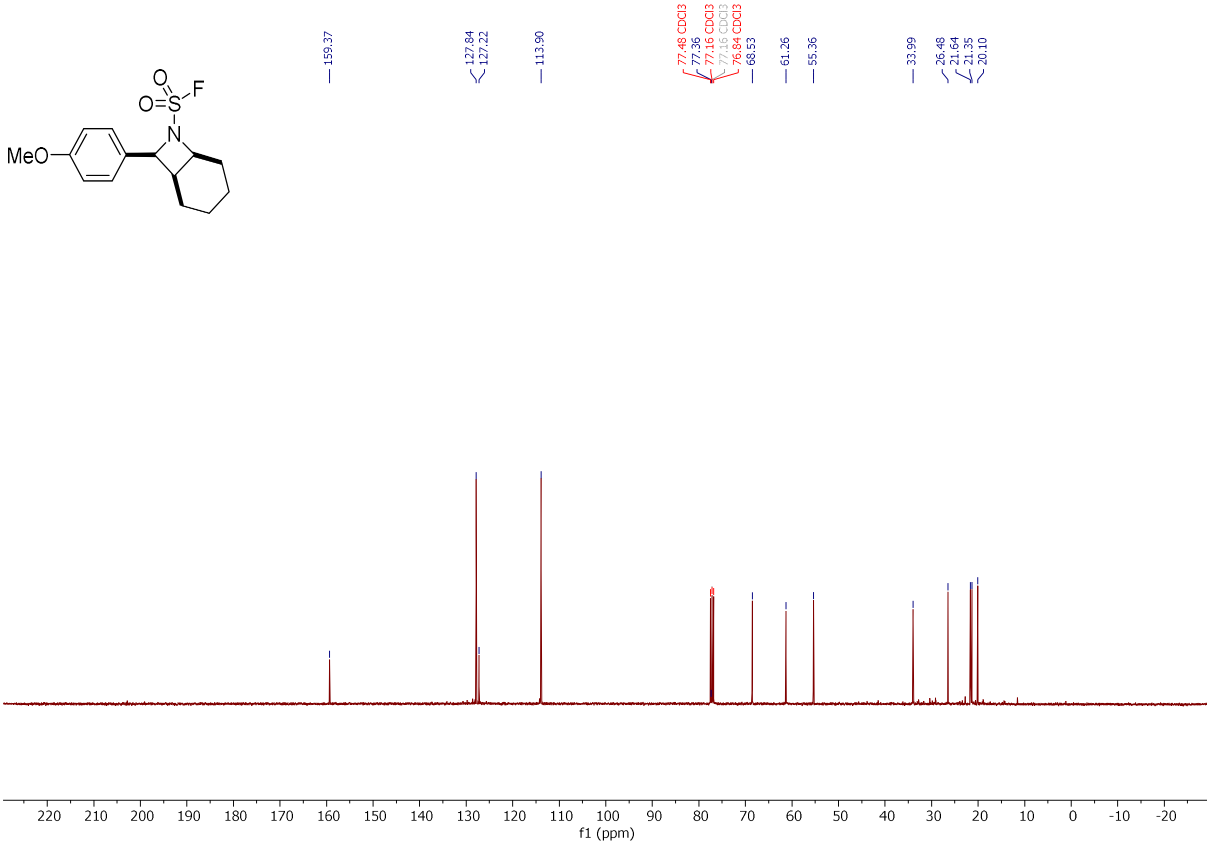

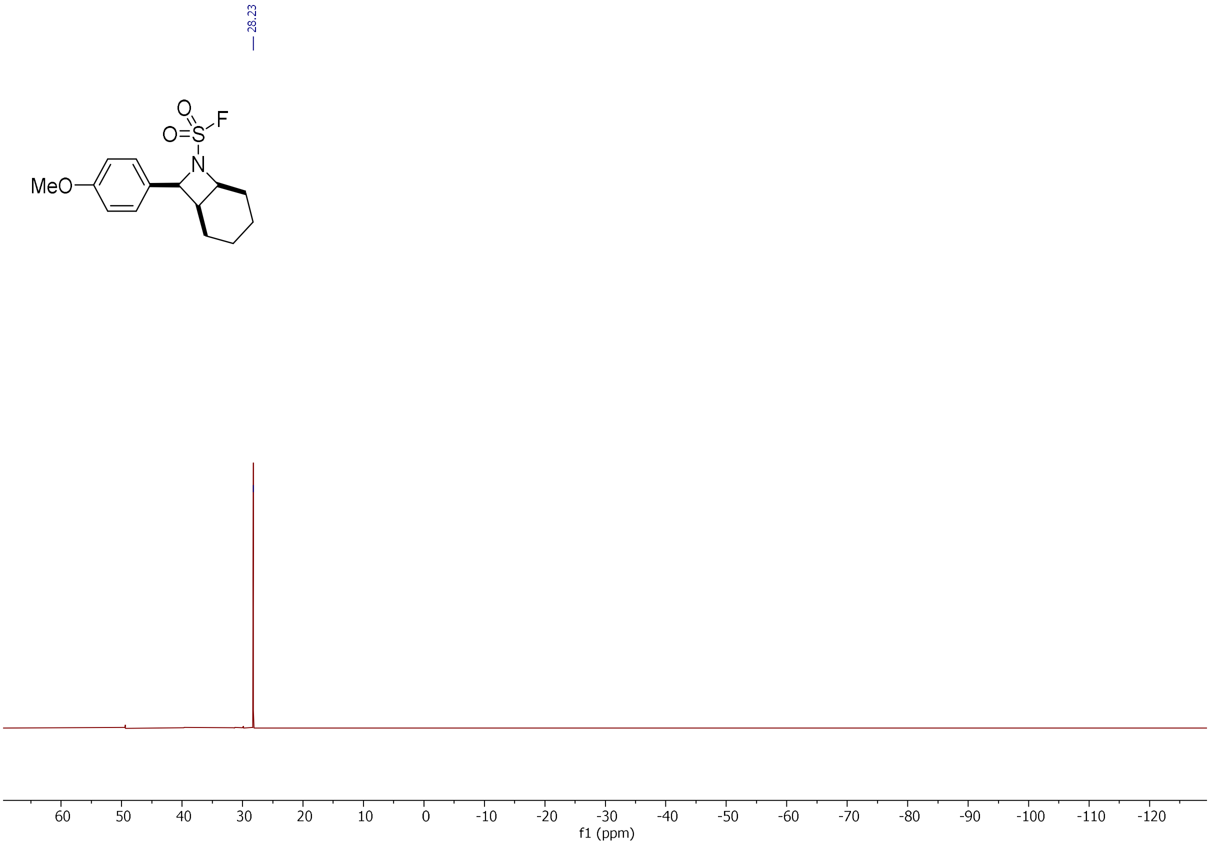
28.23
MeO
O
O=S
F
N
60 50 40 30 20 10 0 -10 -20 -30 -40 -50 -60 -70 -80 -90 -100 -110 -120
f1 (ppm)

(2*S**,3*S**,4*S**)-2,3-diethyl-4-(4-methoxyphenyl)azetidine-1-sulfonyl fluoride (4n)

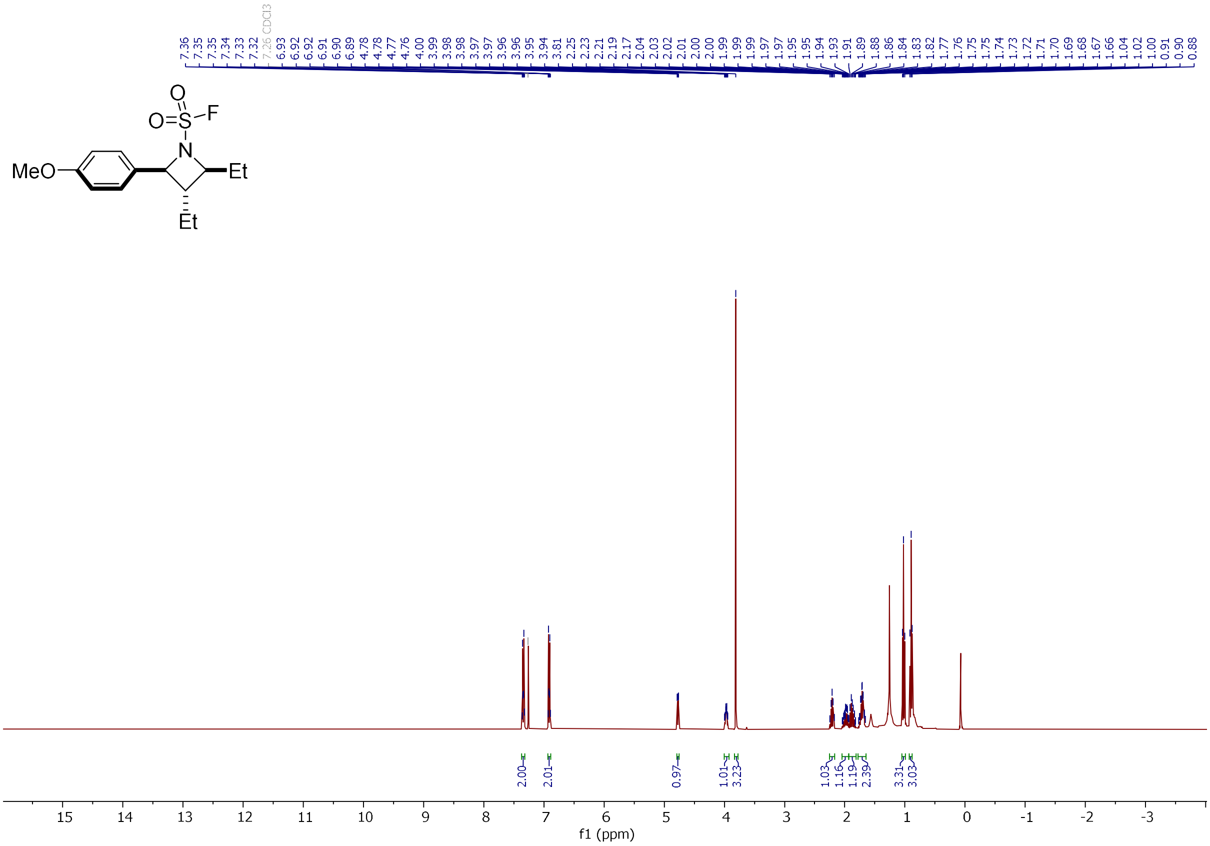

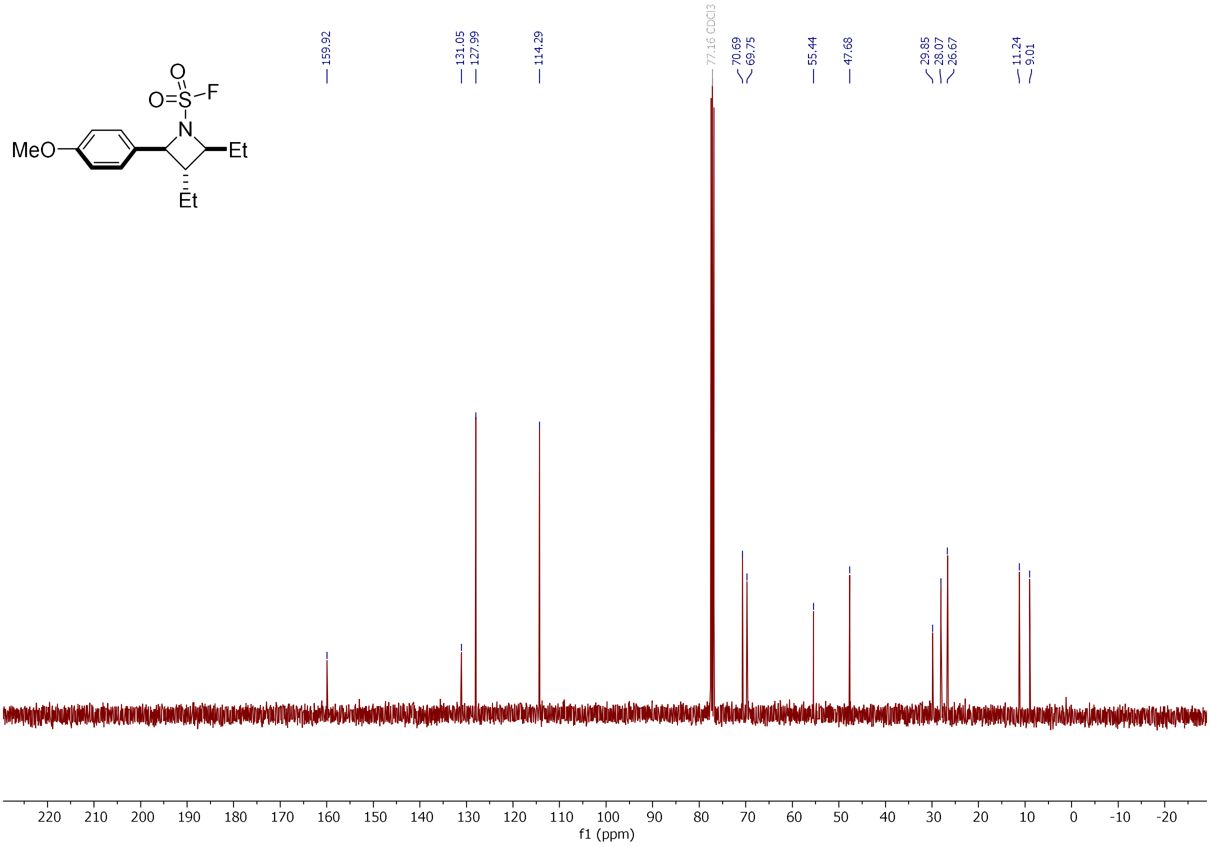

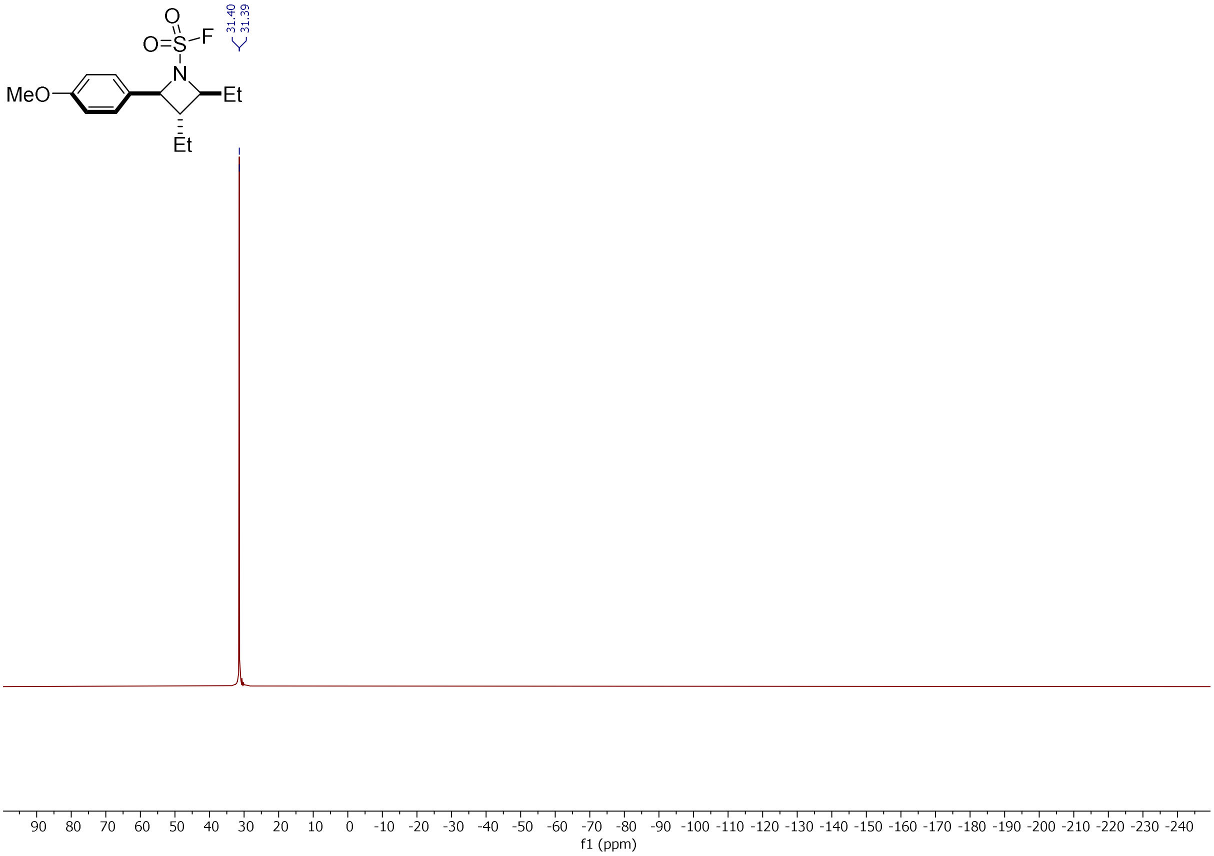

(1*R**,6*S**,8*R**)-8-(4-Methoxyphenyl)-1-methyl-7-azabicyclo[4.2.0]octane-7-sulfonyl fluoride (4o)

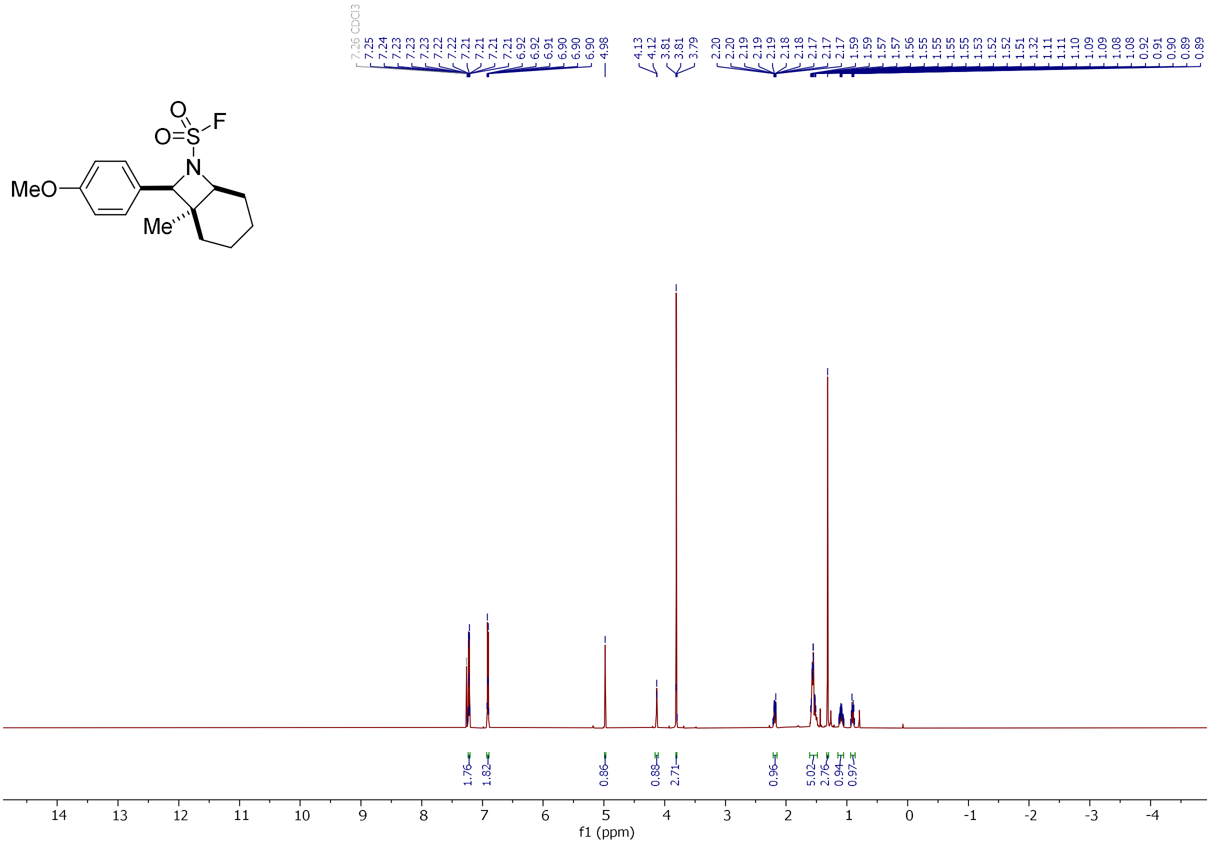

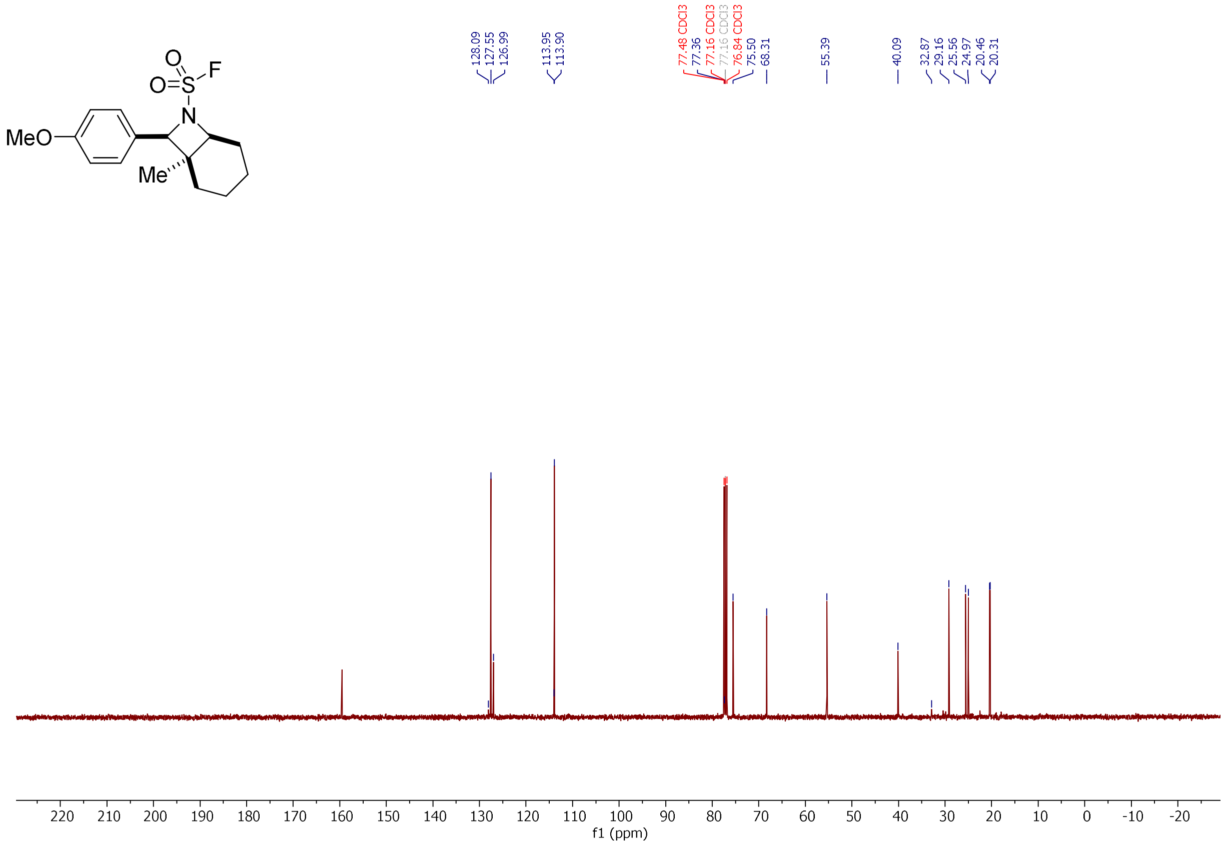
MeO
O
O=S
F
N
Me
128.09
127.55
126.99
113.95
113.90
77.48 CDCl3
77.36
77.16 CDCl3
77.16 CDCl3
76.84 CDCl3
75.50
68.31
55.39
40.09
32.87
29.16
25.56
24.97
20.46
20.31
220 210 200 190 180 170 160 150 140 130 120 110 100 90 80 70 60 50 40 30 20 10 0 -10 -20
f1 (ppm)

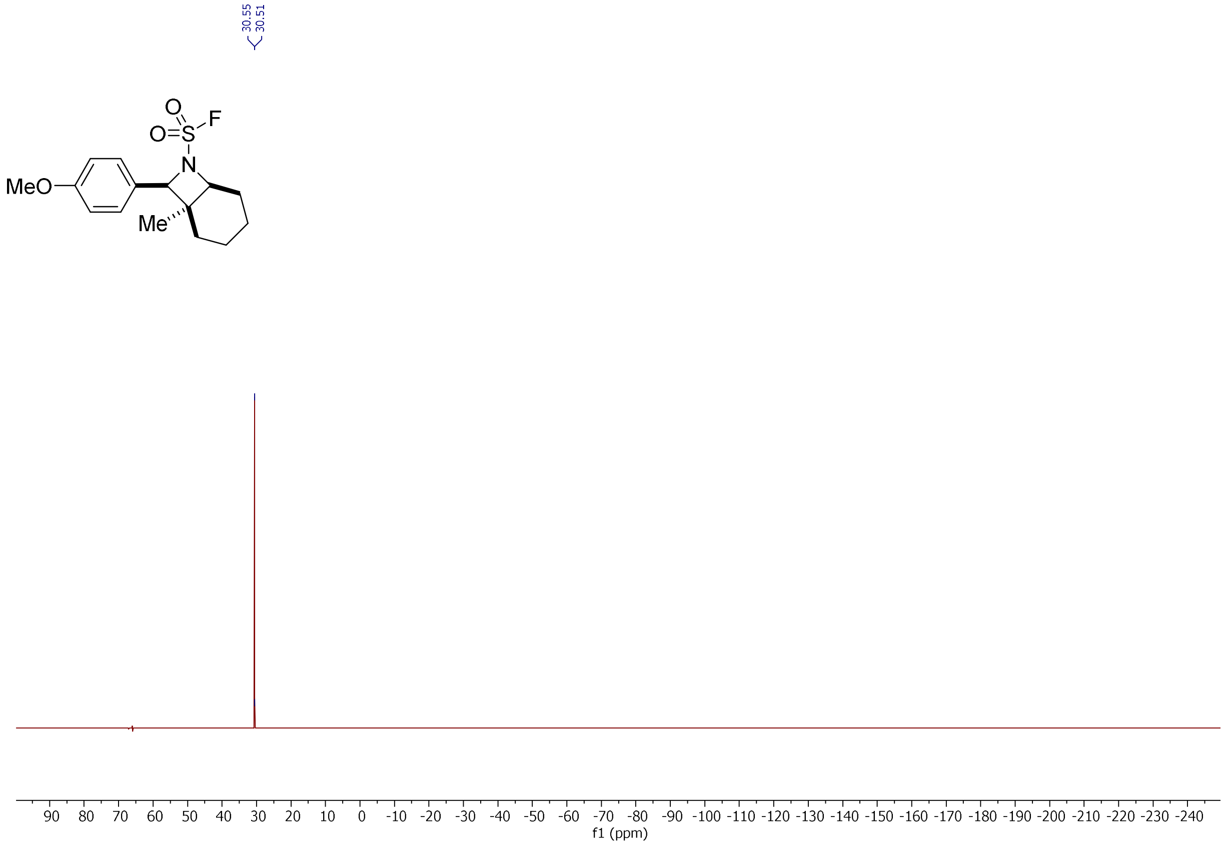
30.55
30.51
MeO
O
O=S
F
N
Me
90 80 70 60 50 40 30 20 10 0 -10 -20 -30 -40 -50 -60 -70 -80 -90 -100 -110 -120 -130 -140 -150 -160 -170 -180 -190 -200 -210 -220 -230 -240
f1 (ppm)

(1*S**,6*R**,8*S**)-8-(4-Methoxyphenyl)-1-methyl-7-azabicyclo[4.2.0]octane-7-sulfonyl fluoride (4o’)

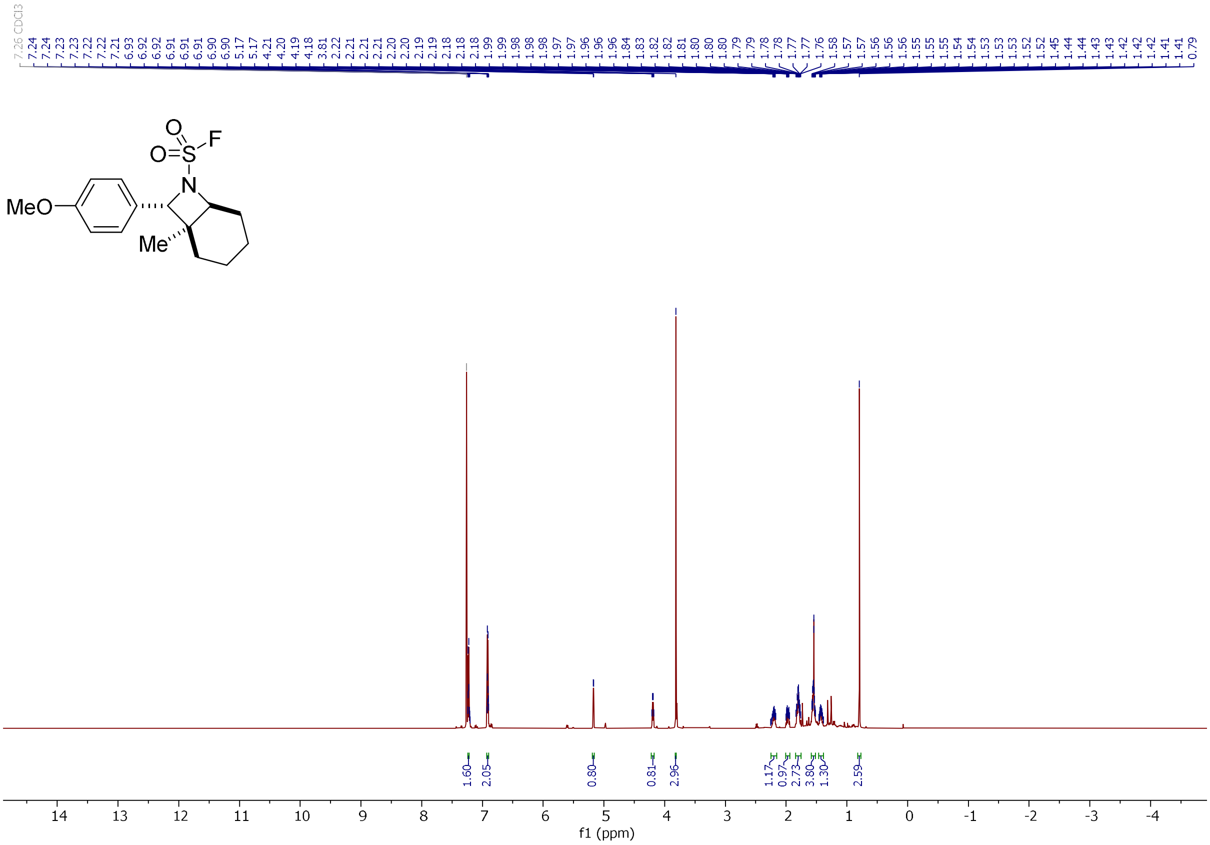

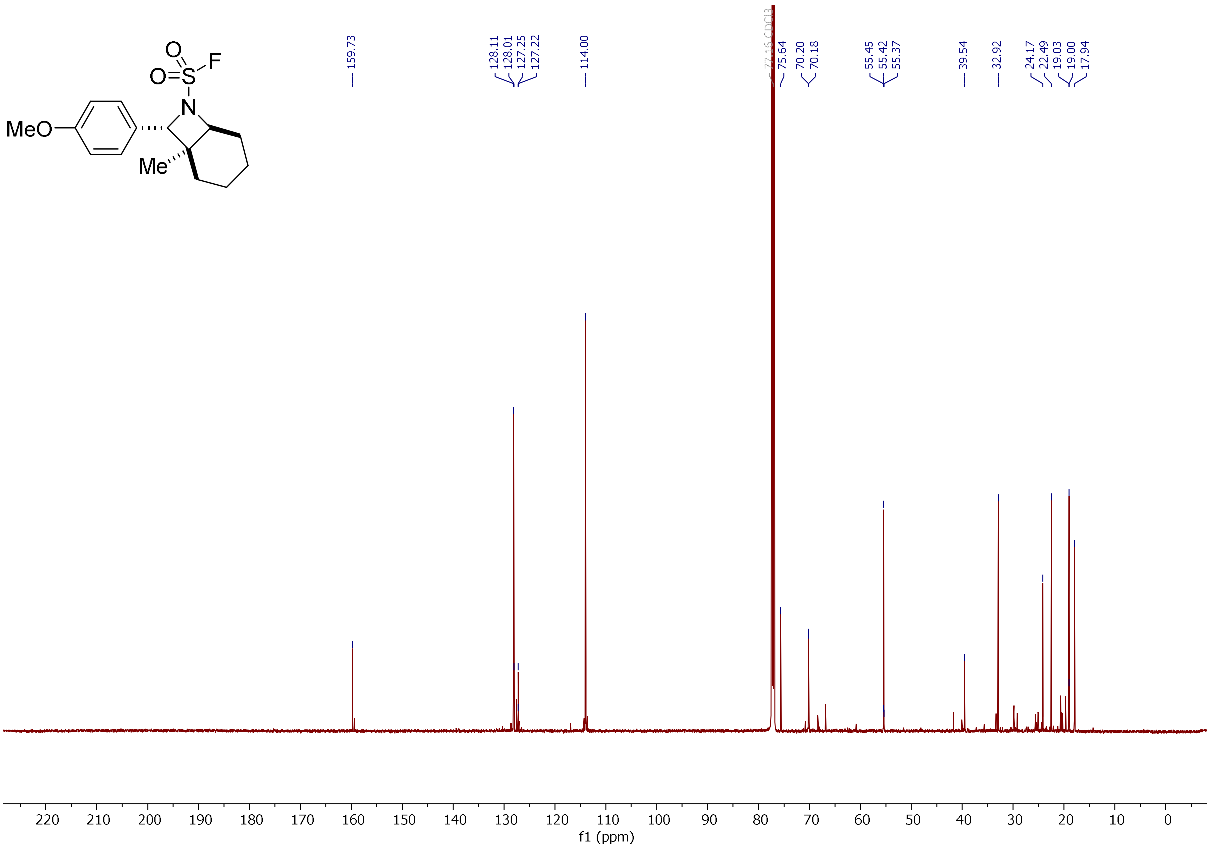

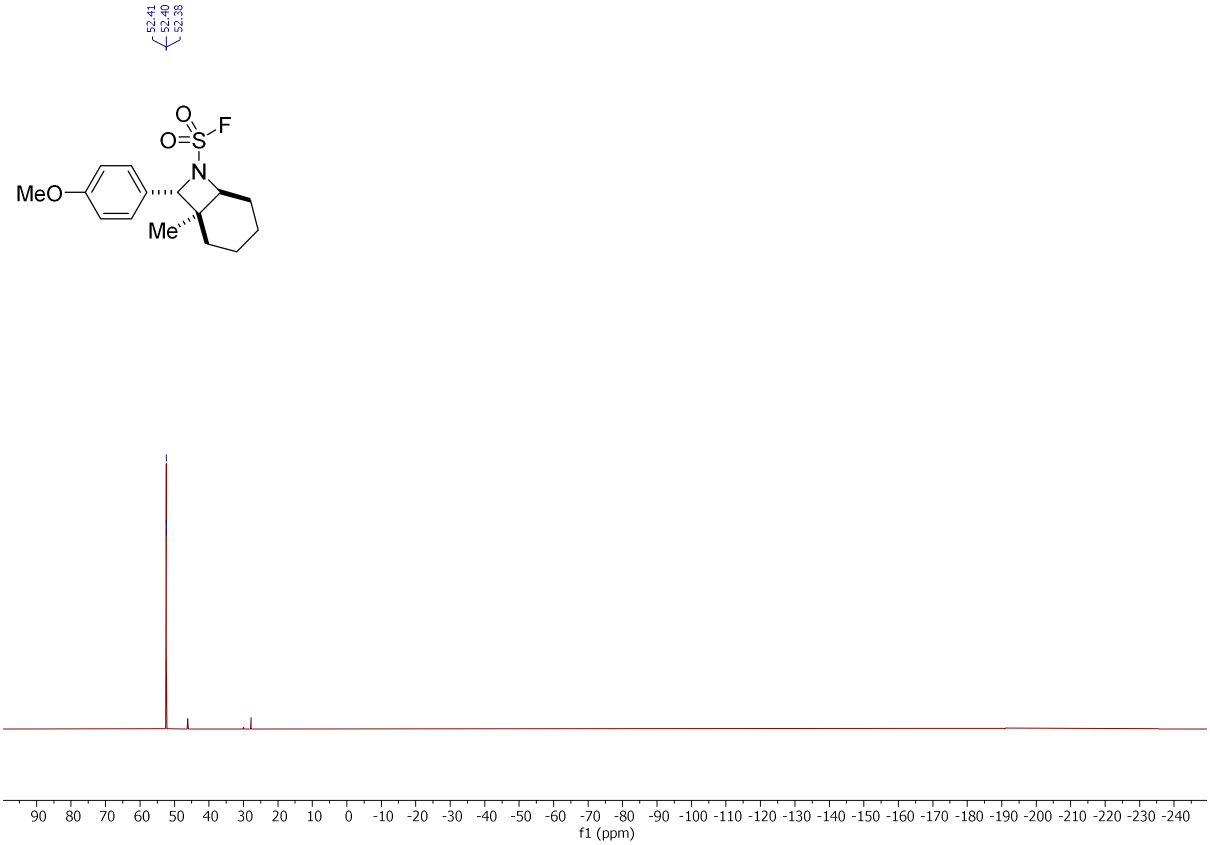
52.41
52.40
52.38
O
O=S
F
N
MeO
Me
90 80 70 60 50 40 30 20 10 0 -10 -20 -30 -40 -50 -60 -70 -80 -90 -100 -110 -120 -130 -140 -150 -160 -170 -180 -190 -200 -210 -220 -230 -240
f1 (ppm)

4-((2*R**,3*S**)-1-(Fluorosulfonyl)-2-(4-methoxyphenyl)azetidin-3-yl)phenyl acetate (4p)

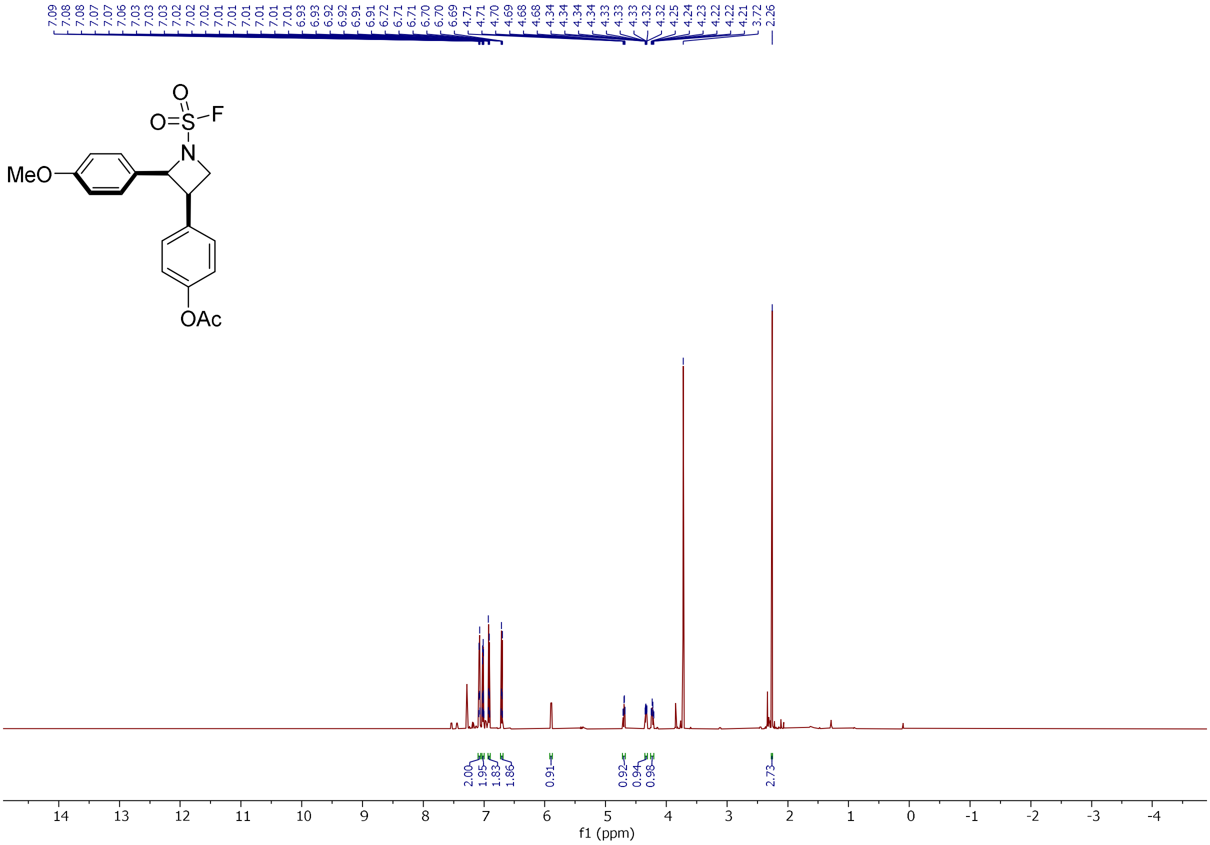

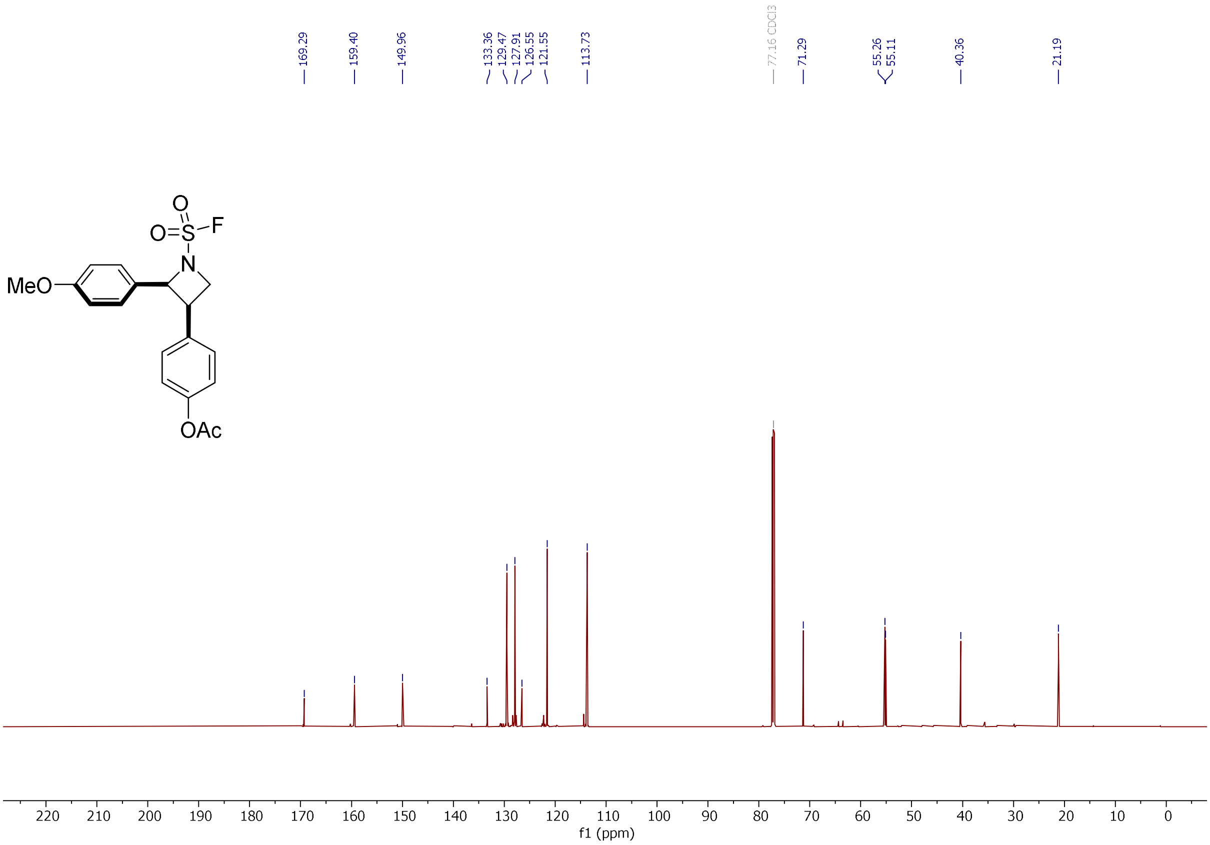

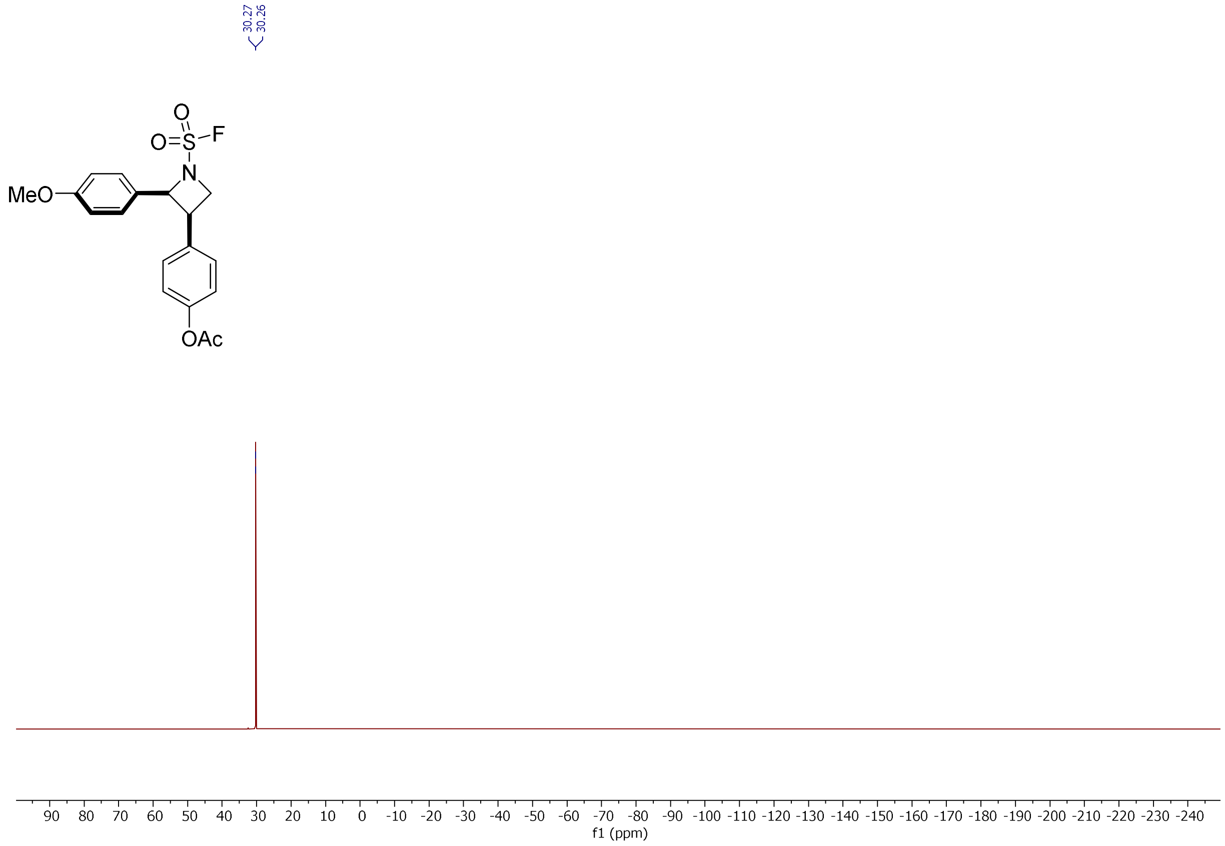
30.27
30.26
MeO
OAc
f1 (ppm)

4-((2*R**,3*R**)-1-(Fluorosulfonyl)-2-(4-methoxyphenyl)azetidin-3-yl)phenyl acetate (4p')

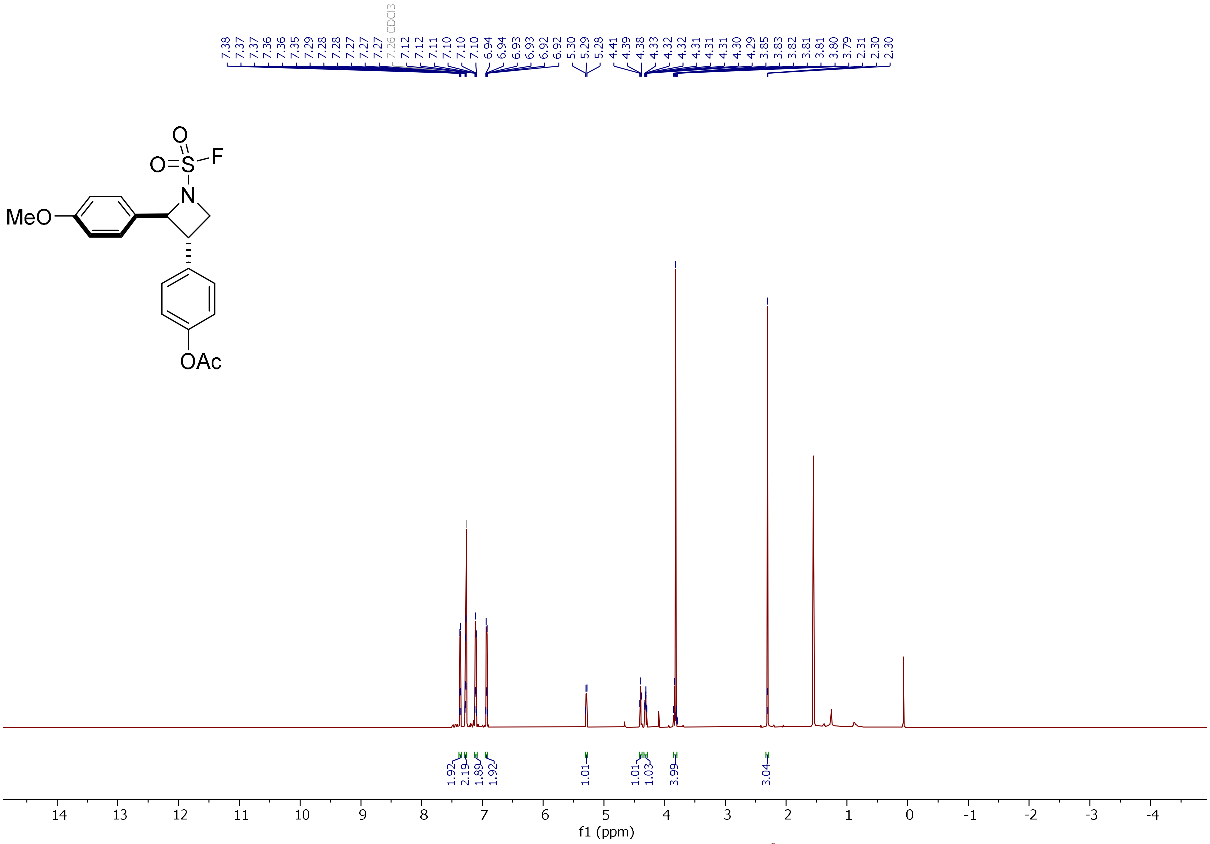

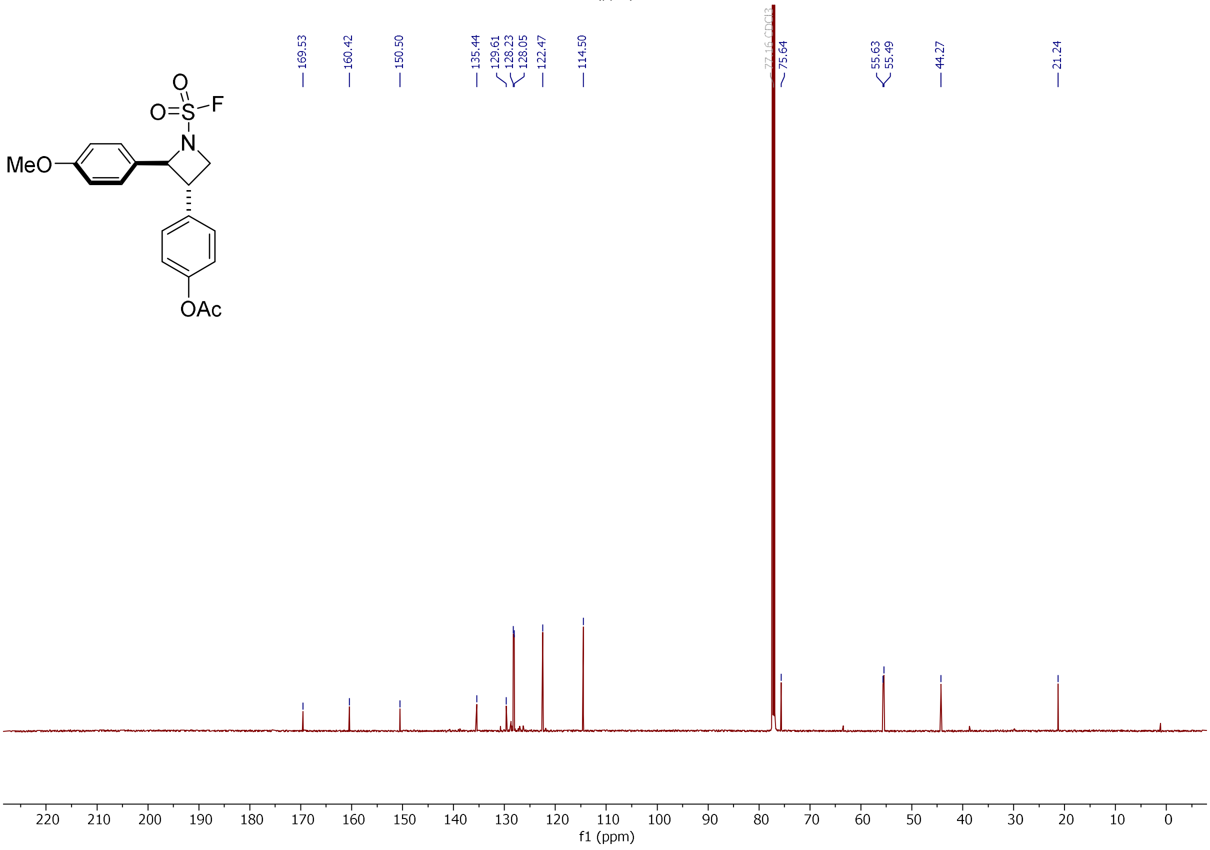

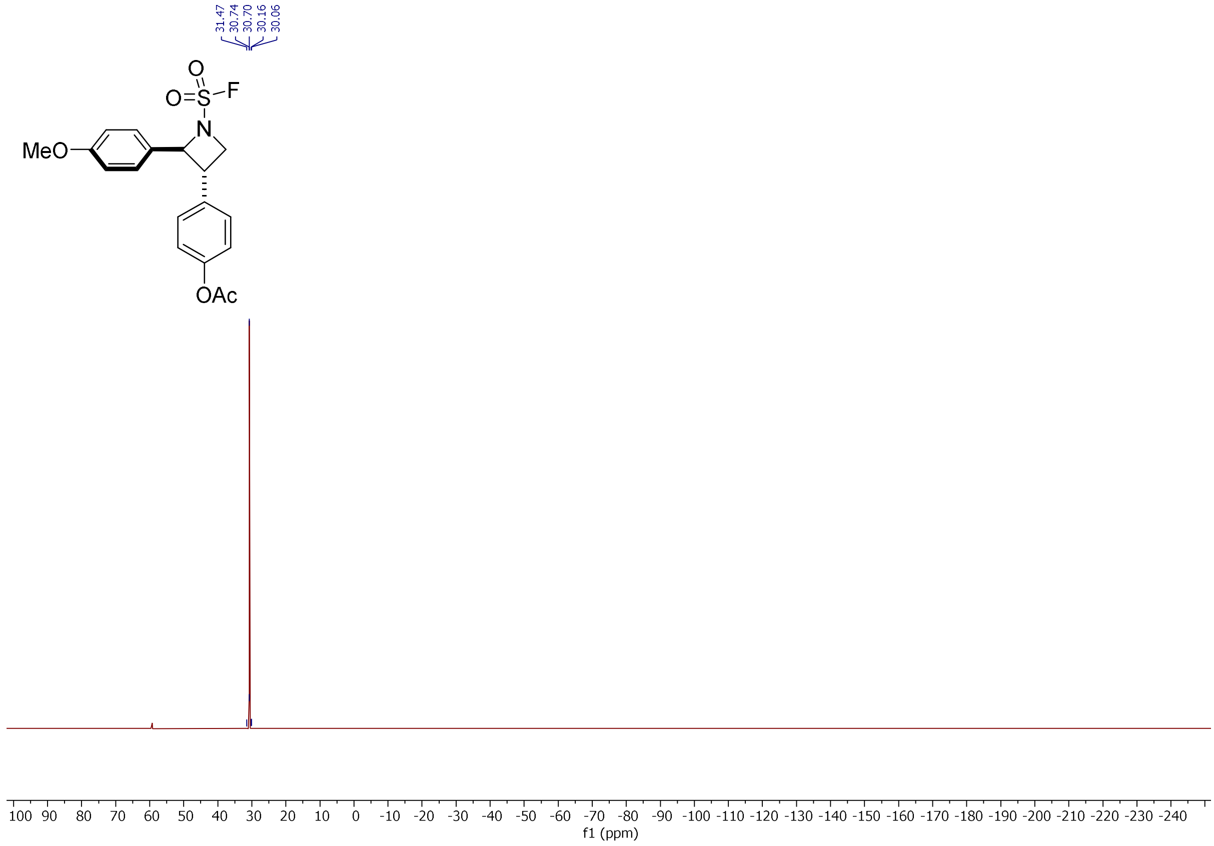

Tert-butyl (5-((2*S**,3*R**)-1-(fluorosulfonyl)-2-(4-methoxyphenyl)azetidin-3-yl)pyridin-2-yl)carbamate (4q')

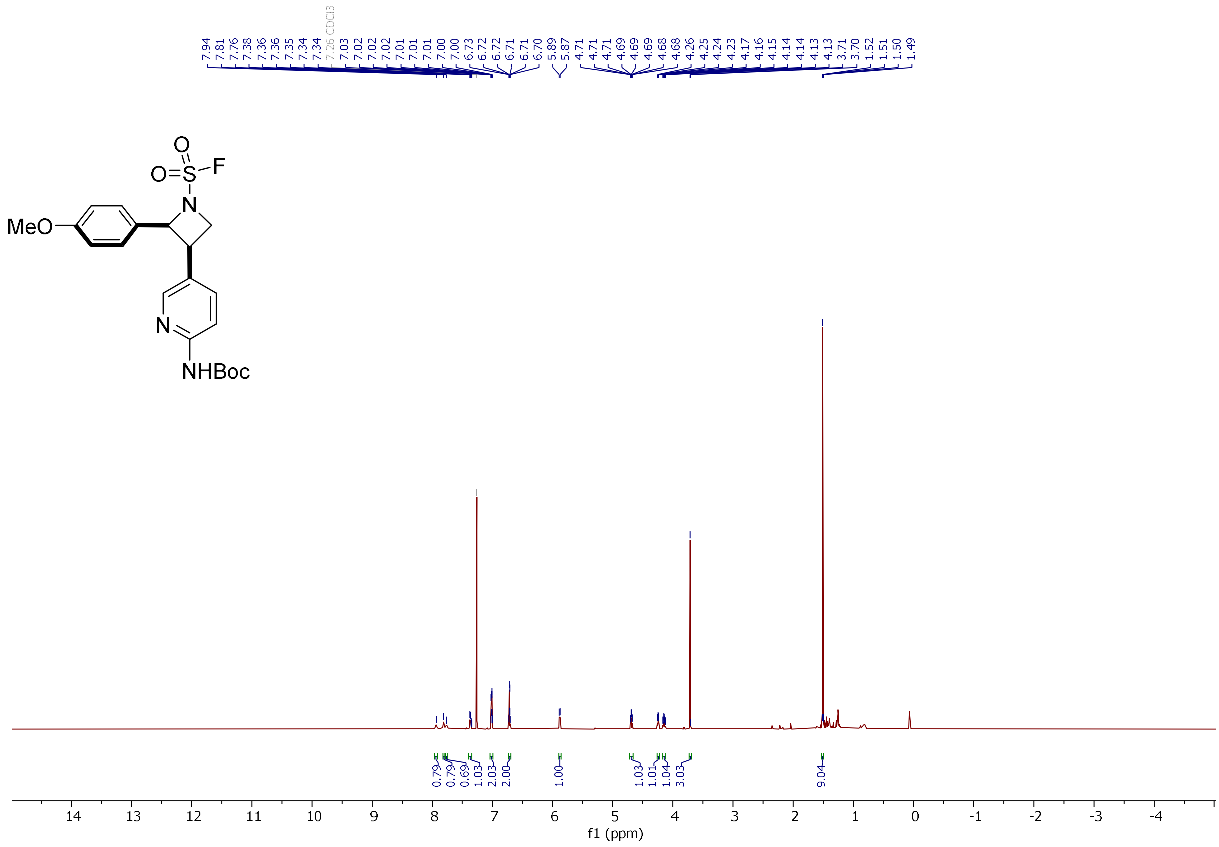

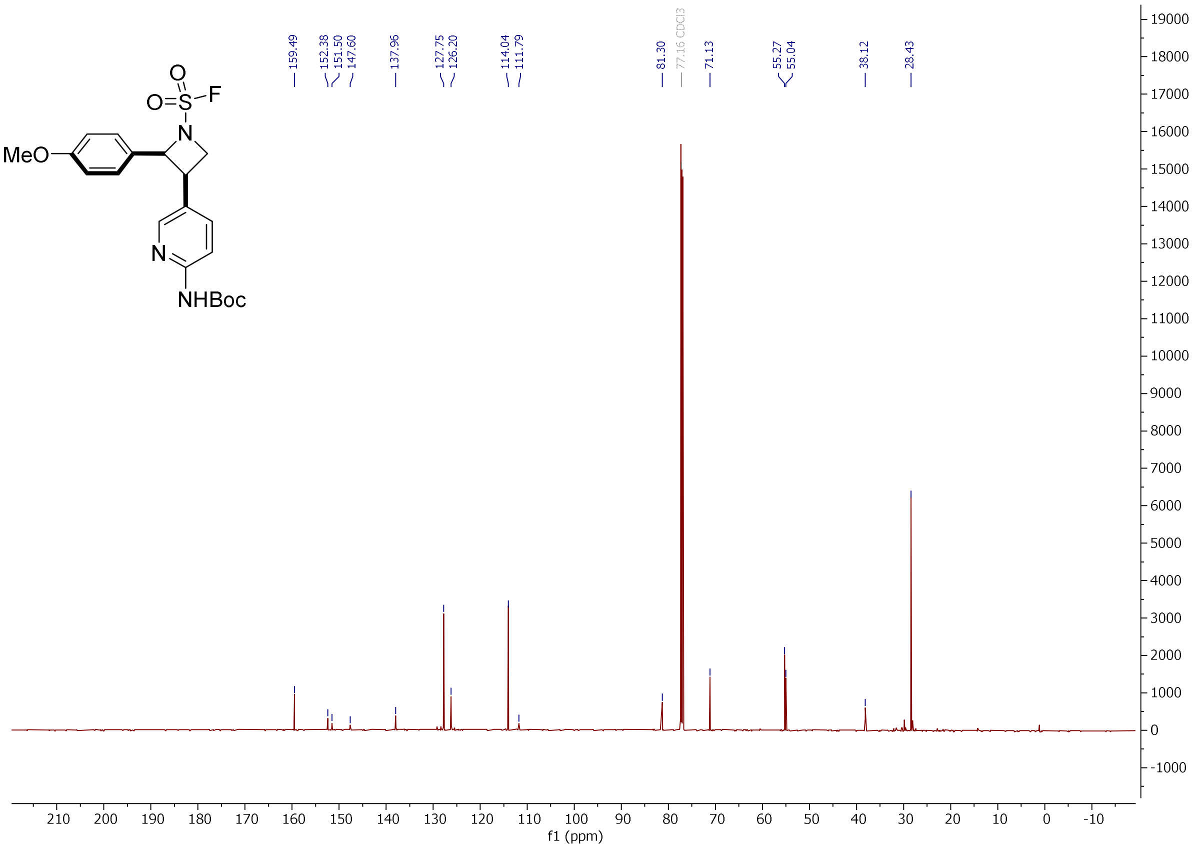
159.49
152.38
151.50
147.60
137.96
127.75
126.20
114.04
111.79
81.30
77.16 CDCl3
71.13
55.27
55.04
38.12
28.43
MeO
O
O=S–F
N
N
NHBoc
f1 (ppm)

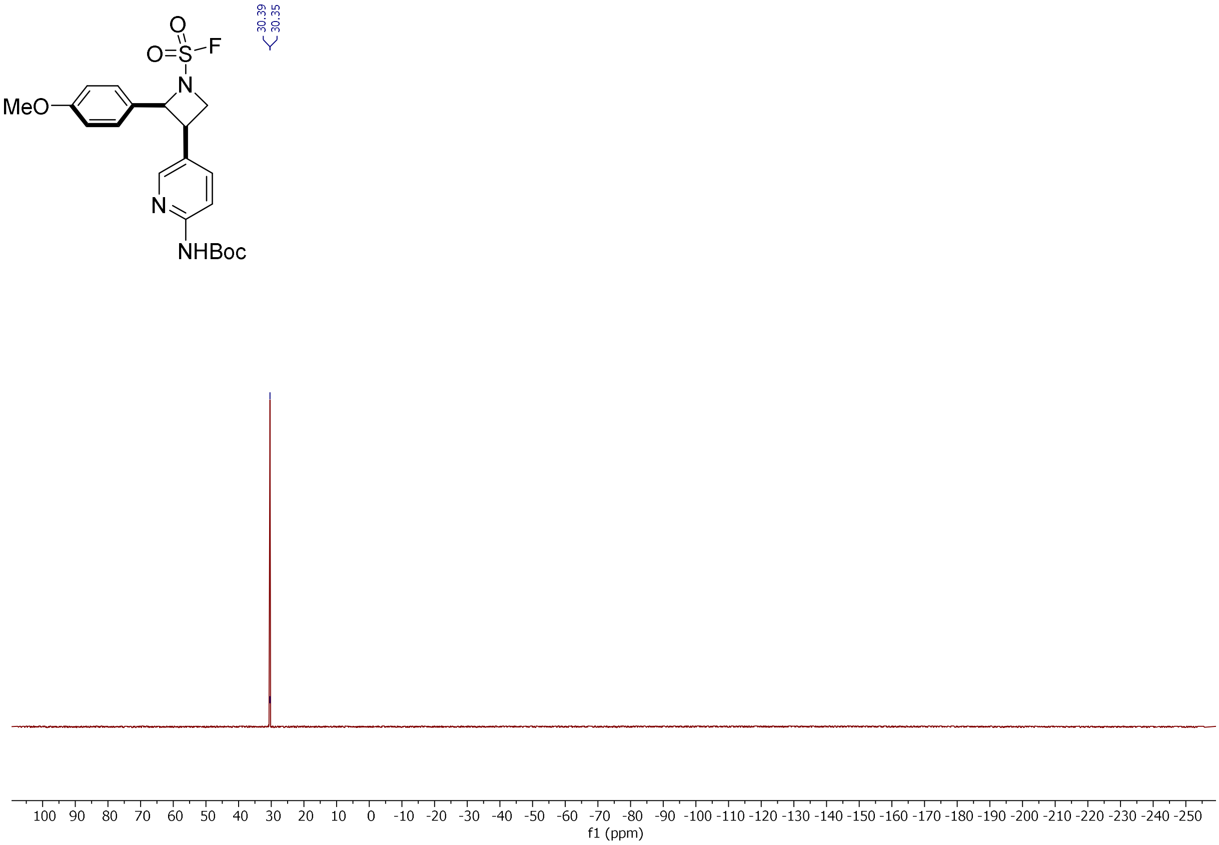
30.39
30.35
MeO
O
O=S–F
N
N
NHBoc
f1 (ppm)

Tert-butyl (tert-butoxycarbonyl)(5-((2*R**,3*R**)-1-(fluorosulfonyl)-2-(4-methoxyphenyl)azetidin-3-yl)pyridin-2-yl)carbamate (4q’)

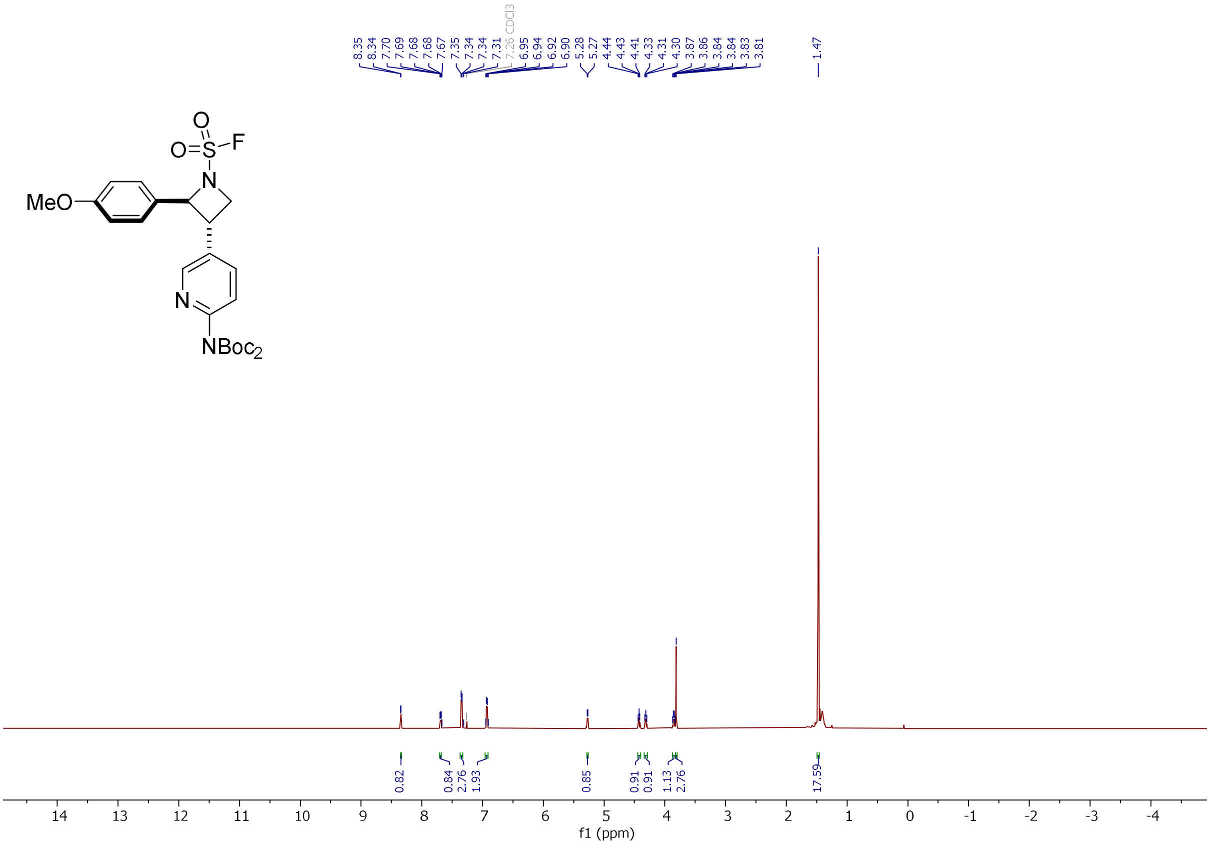

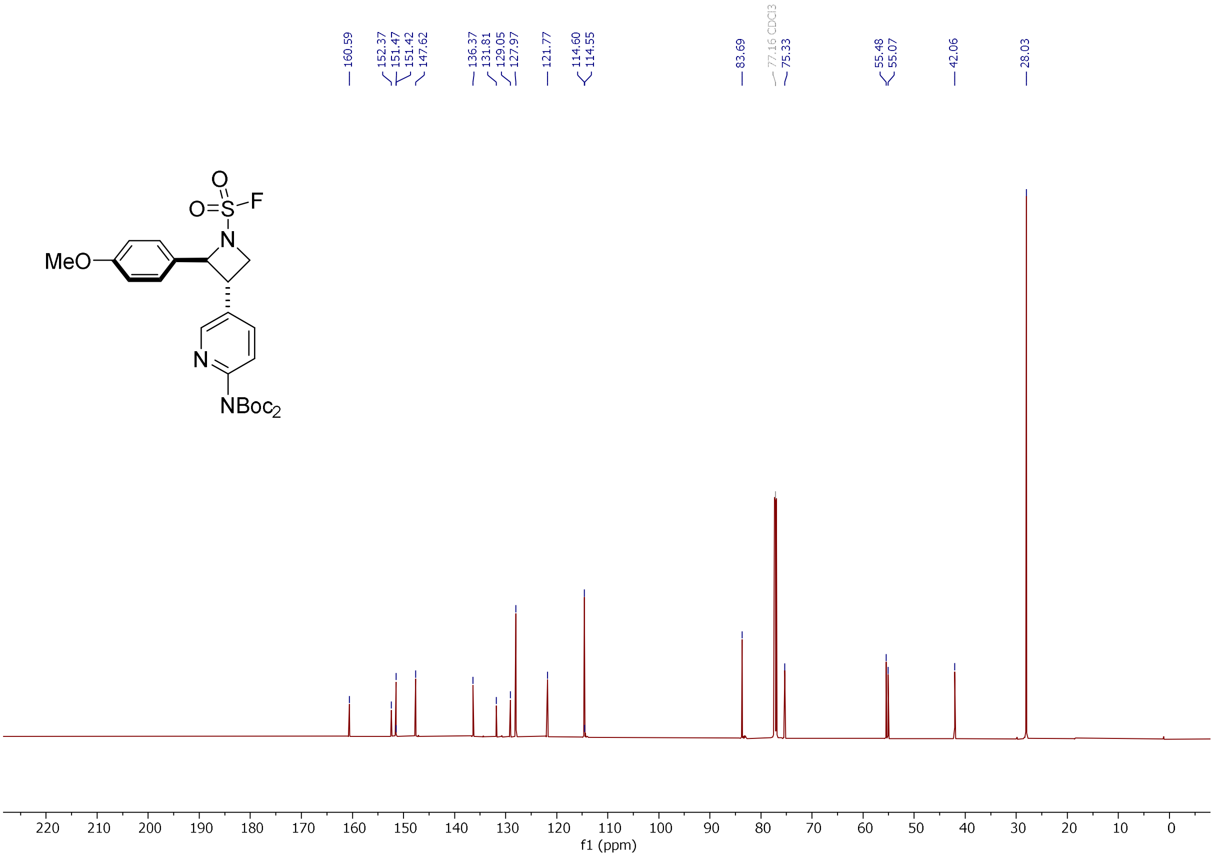

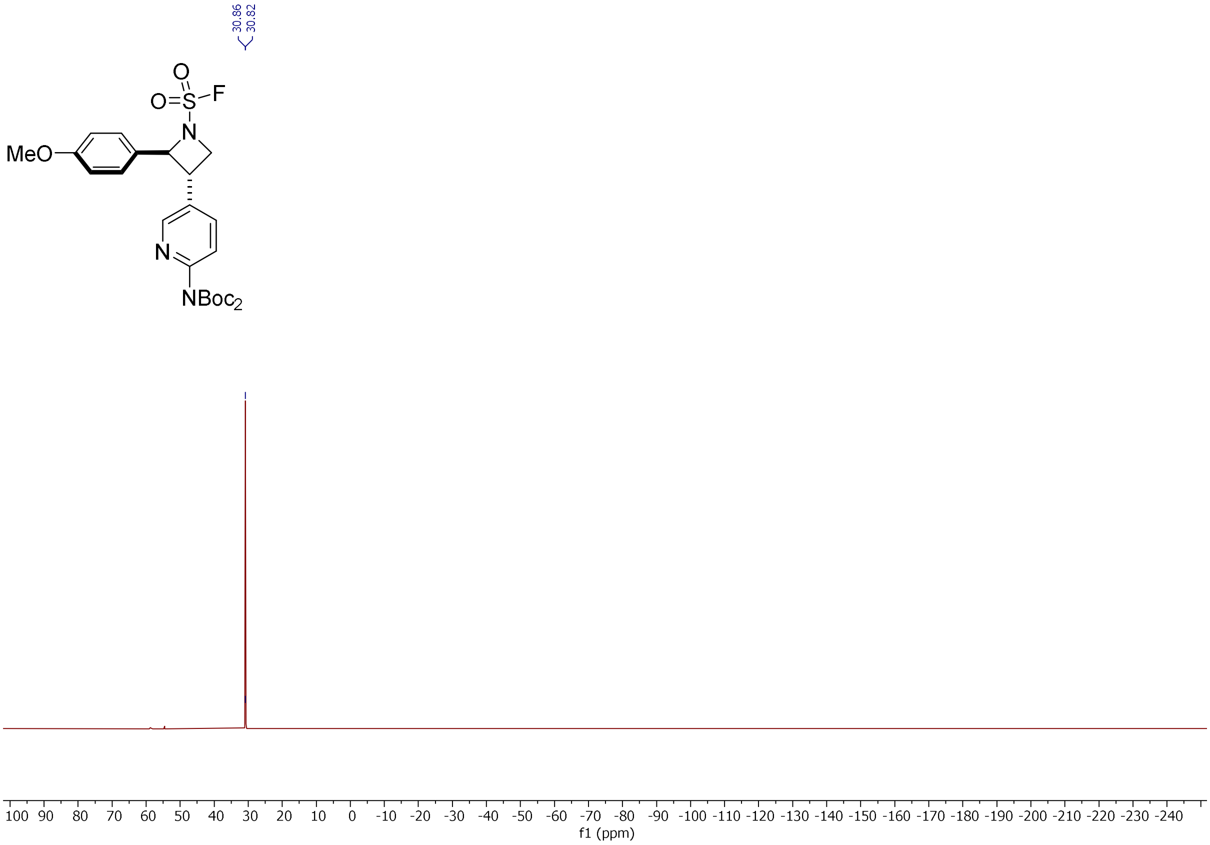

(2*R**,3*R**)-2-(4-Methoxyphenyl) -3-methyl-3-phenylazetidine-1-sulfonyl fluoride (4r)

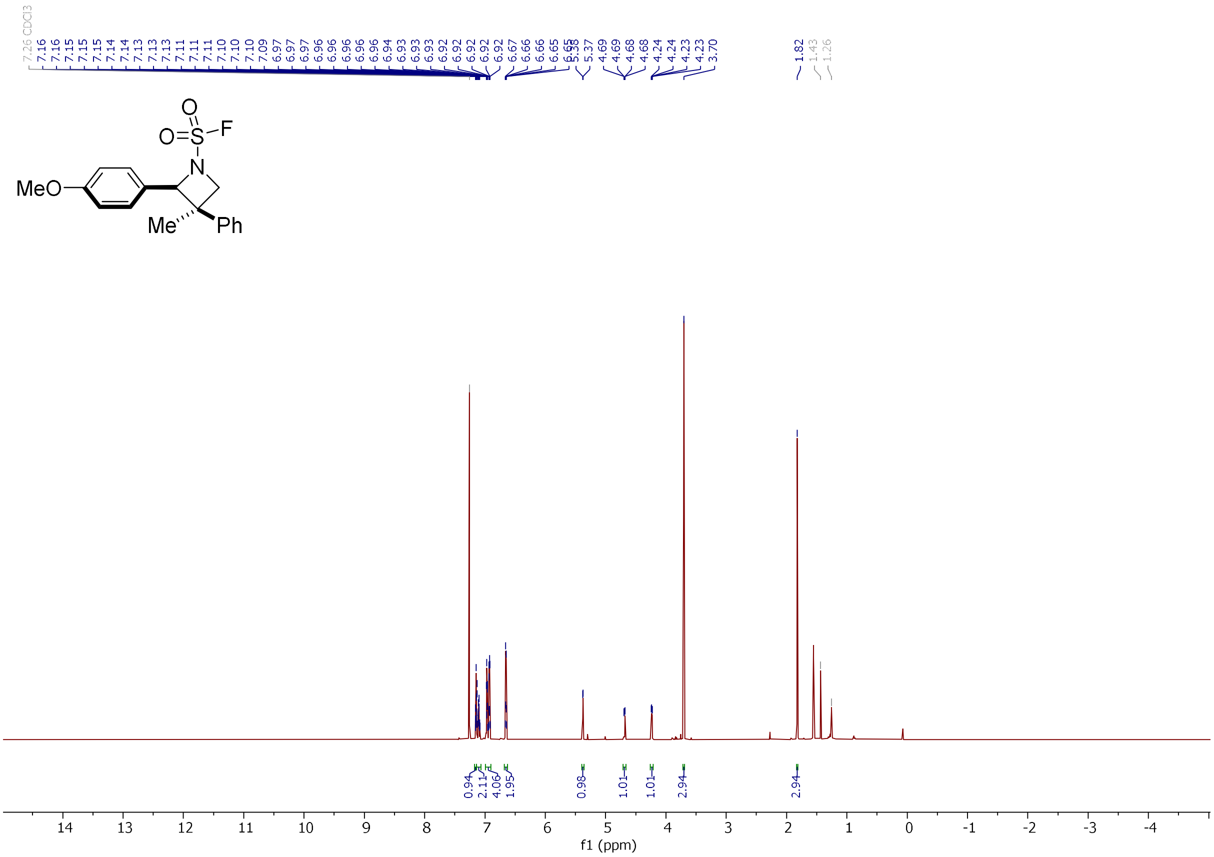

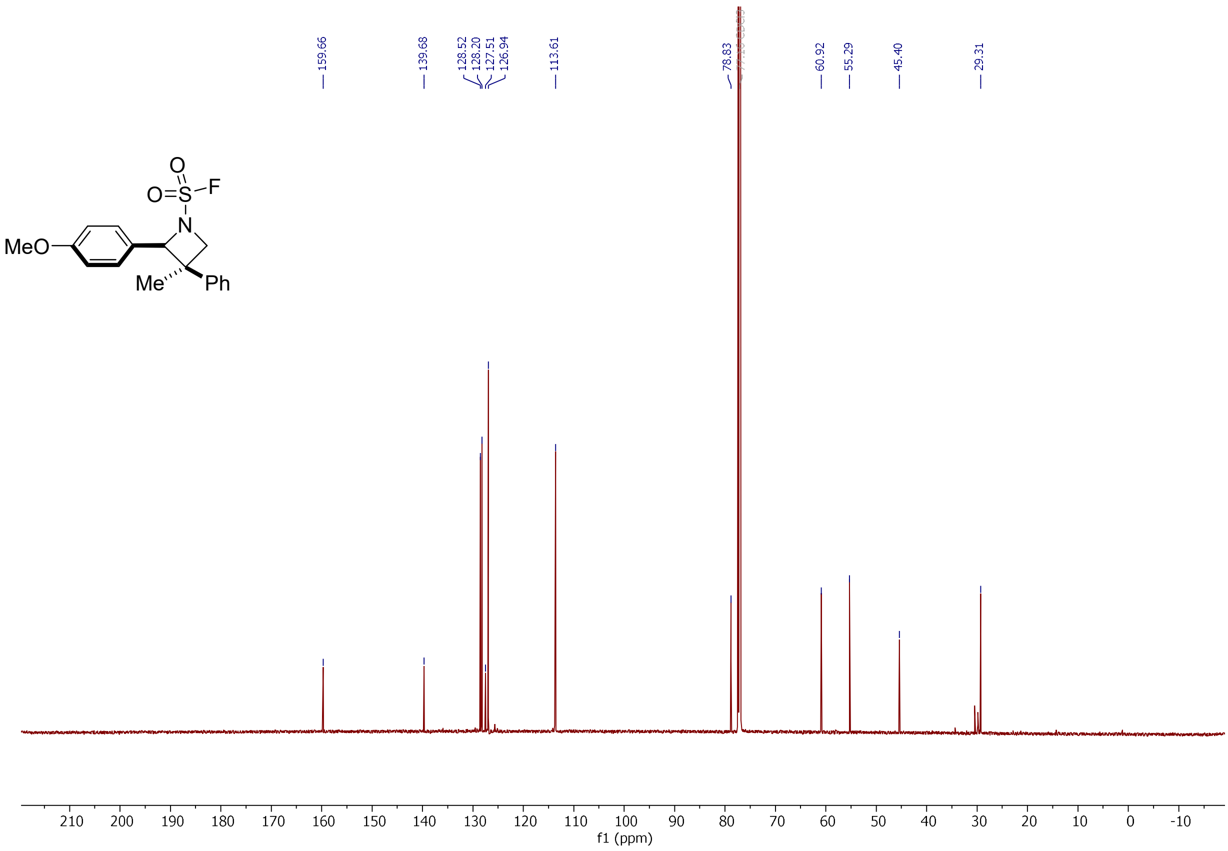
159.66
139.68
128.52
128.20
127.51
126.94
113.61
78.83
60.92
55.29
45.40
29.31
MeO
Me
Ph
f1 (ppm)

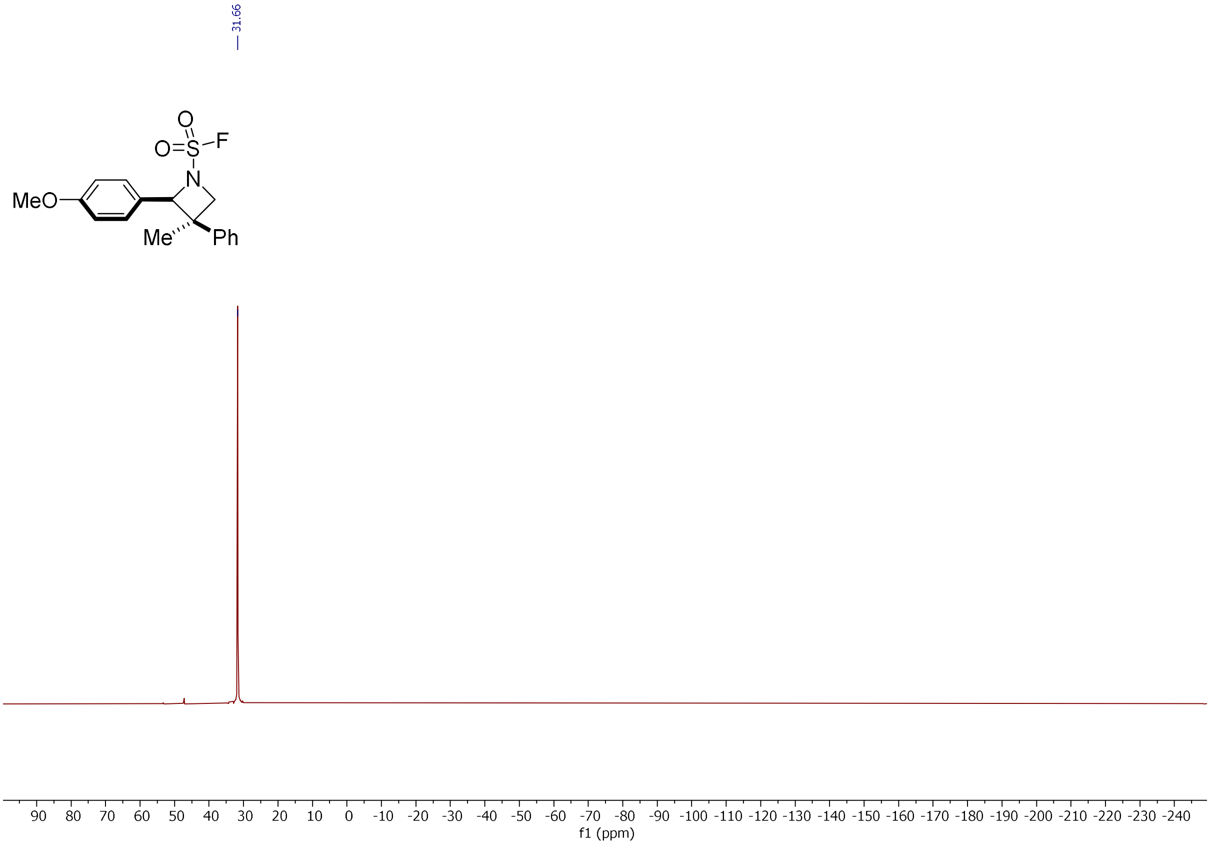
31.66
MeO
Me
Ph
f1 (ppm)

(2*R**,3*S**)-2-(4-Methoxyphenyl)-3-methyl-3-phenylazetidine-1-sulfonyl fluoride (4r’)

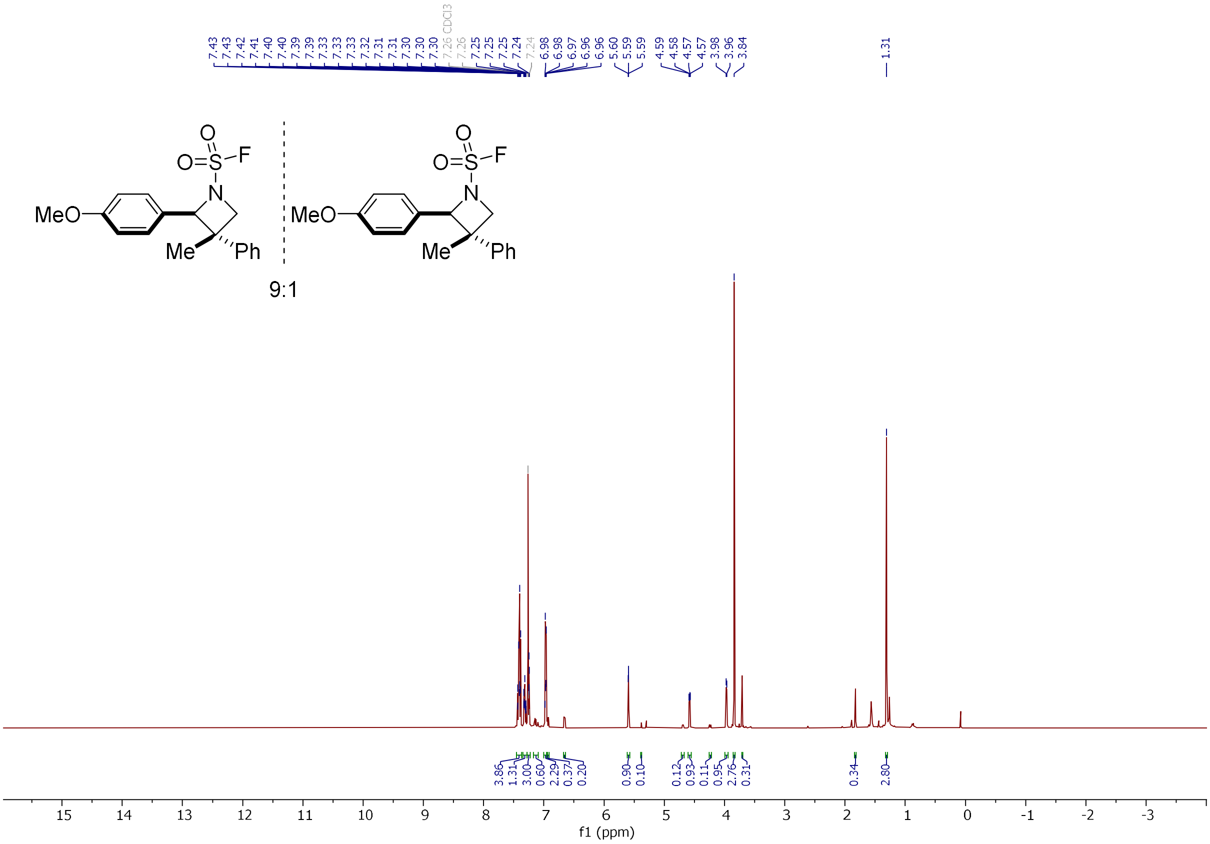

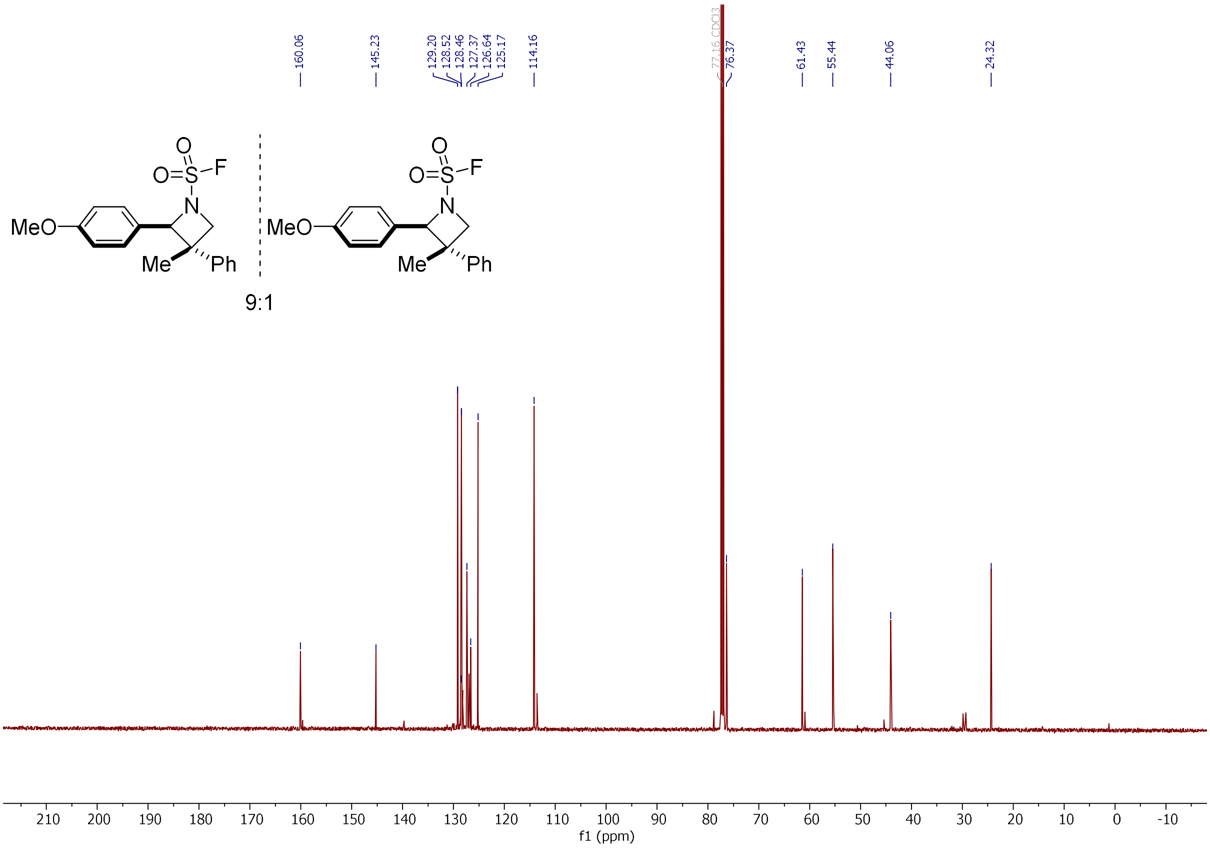
160.06
145.23
129.20
128.52
128.46
127.37
126.64
125.17
114.16
77.16 CDCl3
76.37
61.43
55.44
44.06
24.32
MeO
O=S-F
N
Me
Ph
9:1
f1 (ppm)

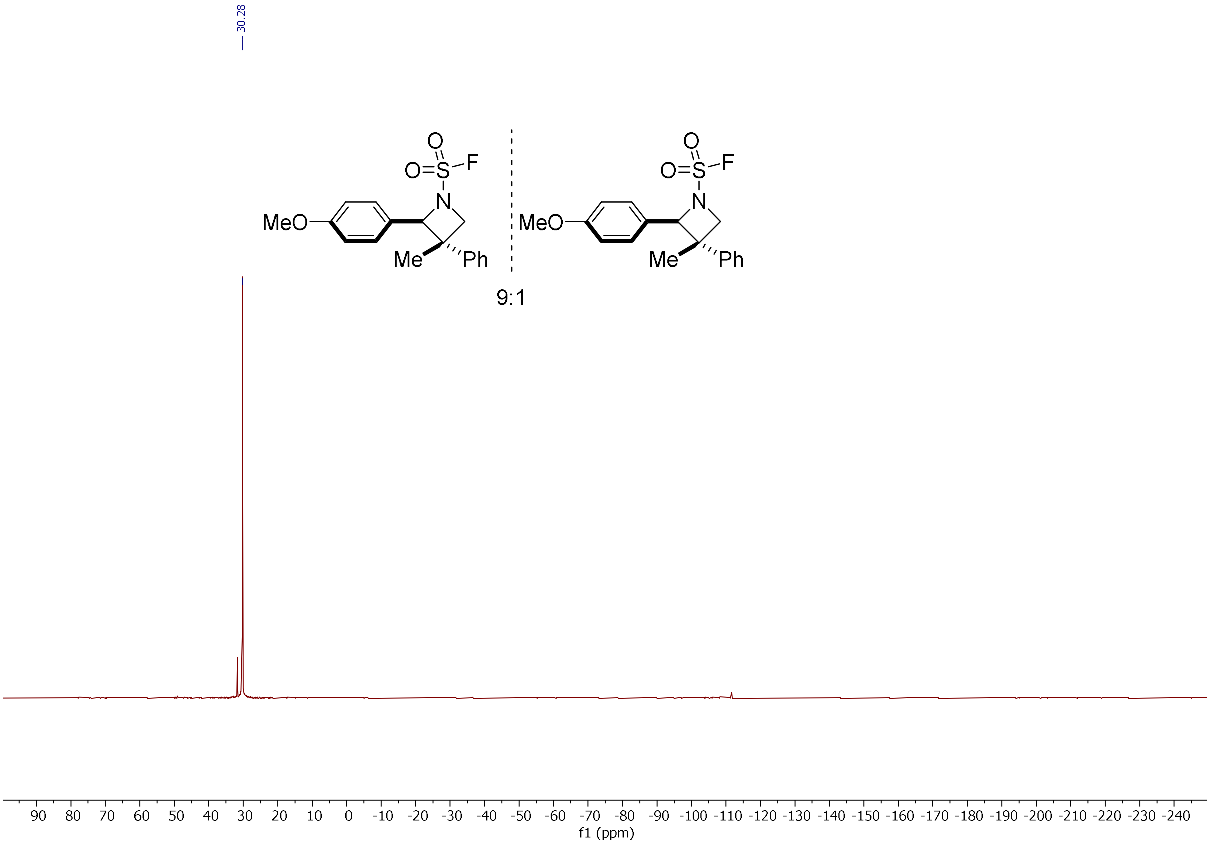
30.28
MeO
O=S-F
N
Me
Ph
9:1
f1 (ppm)

(2*R**,3*S**)-3-Hydroxy-2-(4-methoxyphenyl)-3-methylazetidine-1-sulfonyl fluoride (4s) and (2*R**,3*R**)-3-Hydroxy-2-(4-methoxyphenyl)-3-methylazetidine-1-sulfonyl fluoride (4s')

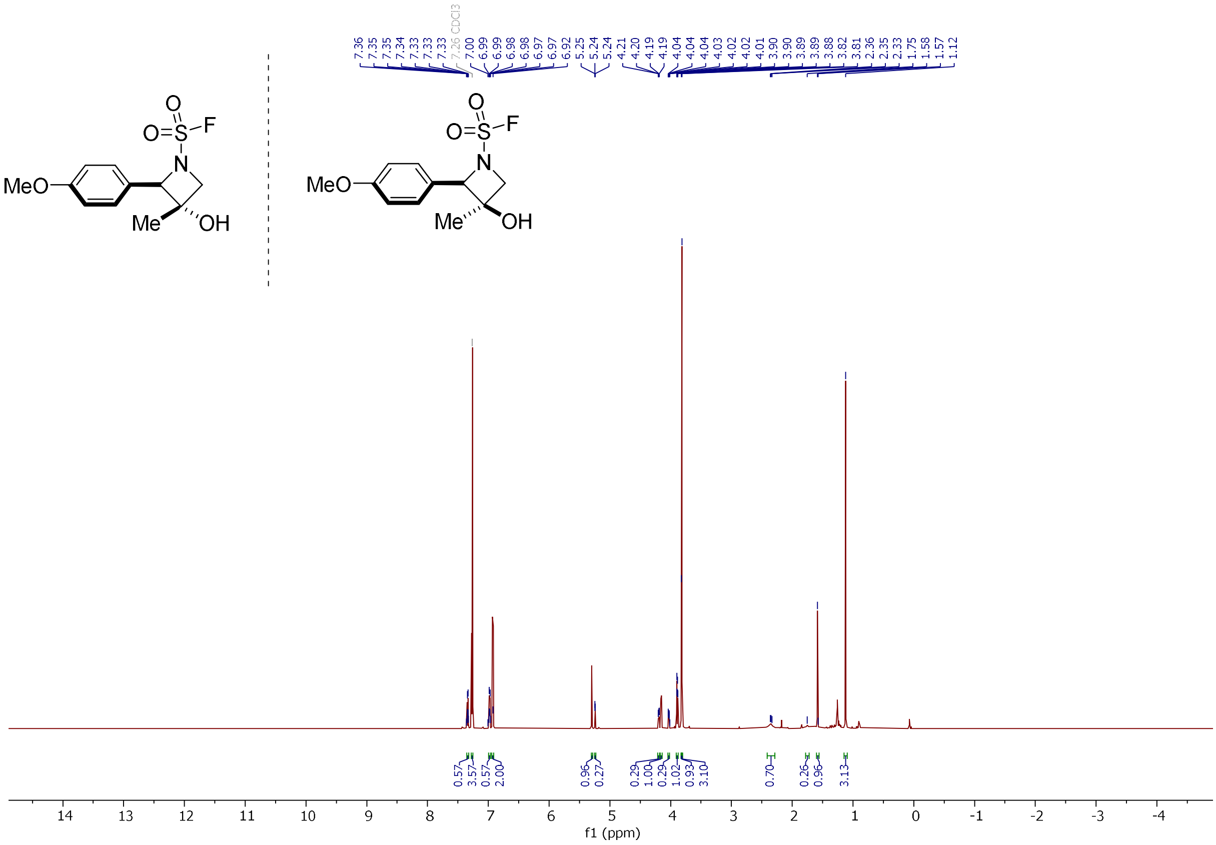

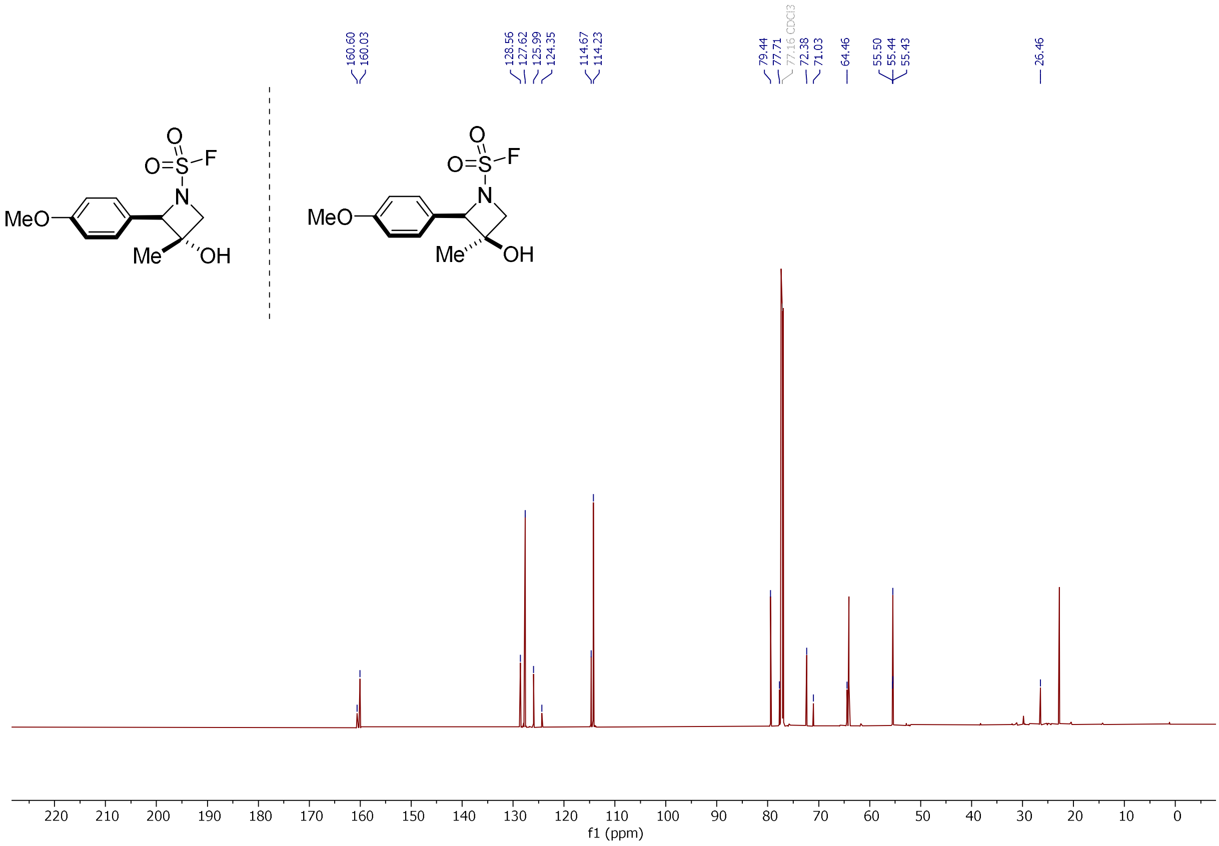
160.60
160.03
128.56
127.62
125.99
124.35
114.67
114.23
79.44
77.71
77.16 CDCl3
72.38
71.03
64.46
55.50
55.44
55.43
26.46
MeO
O
O=S-F
N
Me
OH
220 210 200 190 180 170 160 150 140 130 120 110 100 90 80 70 60 50 40 30 20 10 0
f1 (ppm)

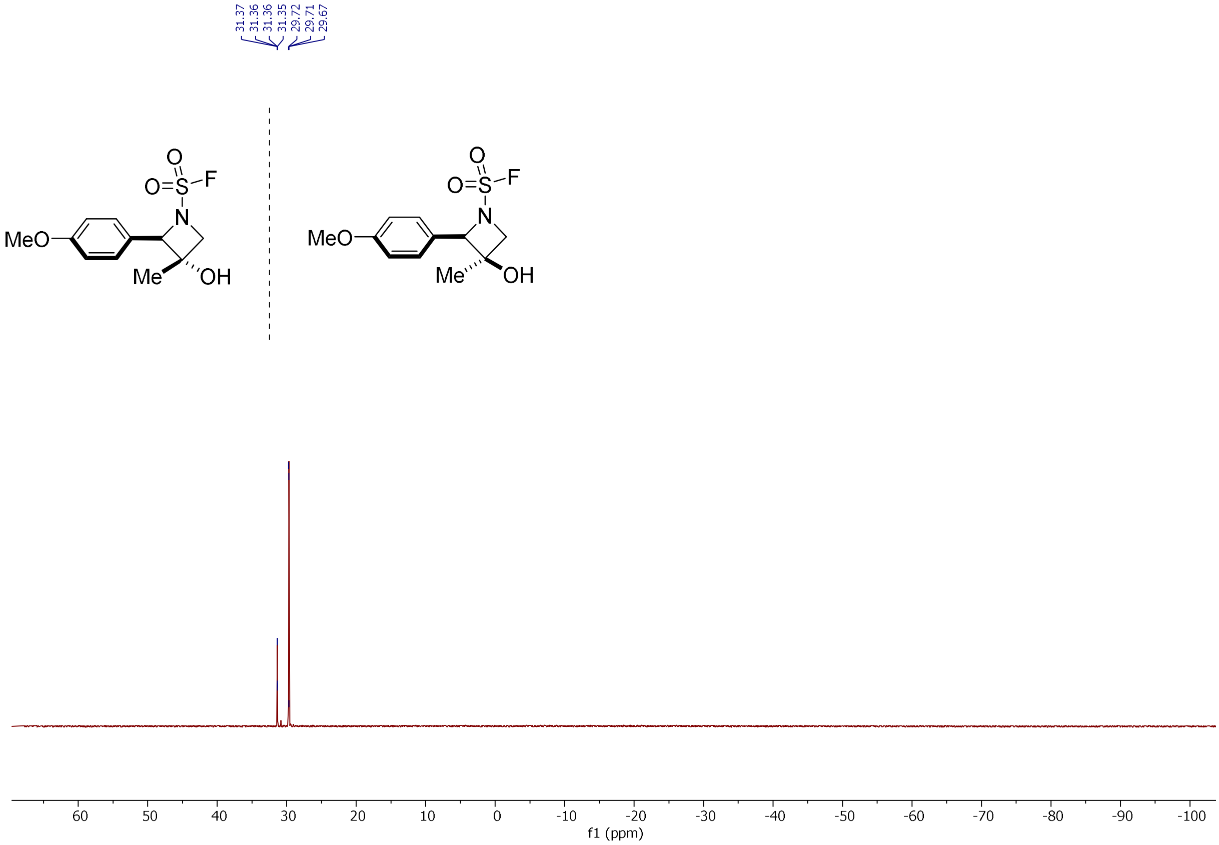
31.37
31.36
31.36
31.35
29.72
29.71
29.67
MeO
O
O=S-F
N
Me
OH
60 50 40 30 20 10 0 -10 -20 -30 -40 -50 -60 -70 -80 -90 -100
f1 (ppm)

(2*R**,3*R**)-2-(4-Methoxyphenyl)-3-(2-oxopyrrolidin-1-yl)azetidine-1-sulfonyl fluoride (4t)

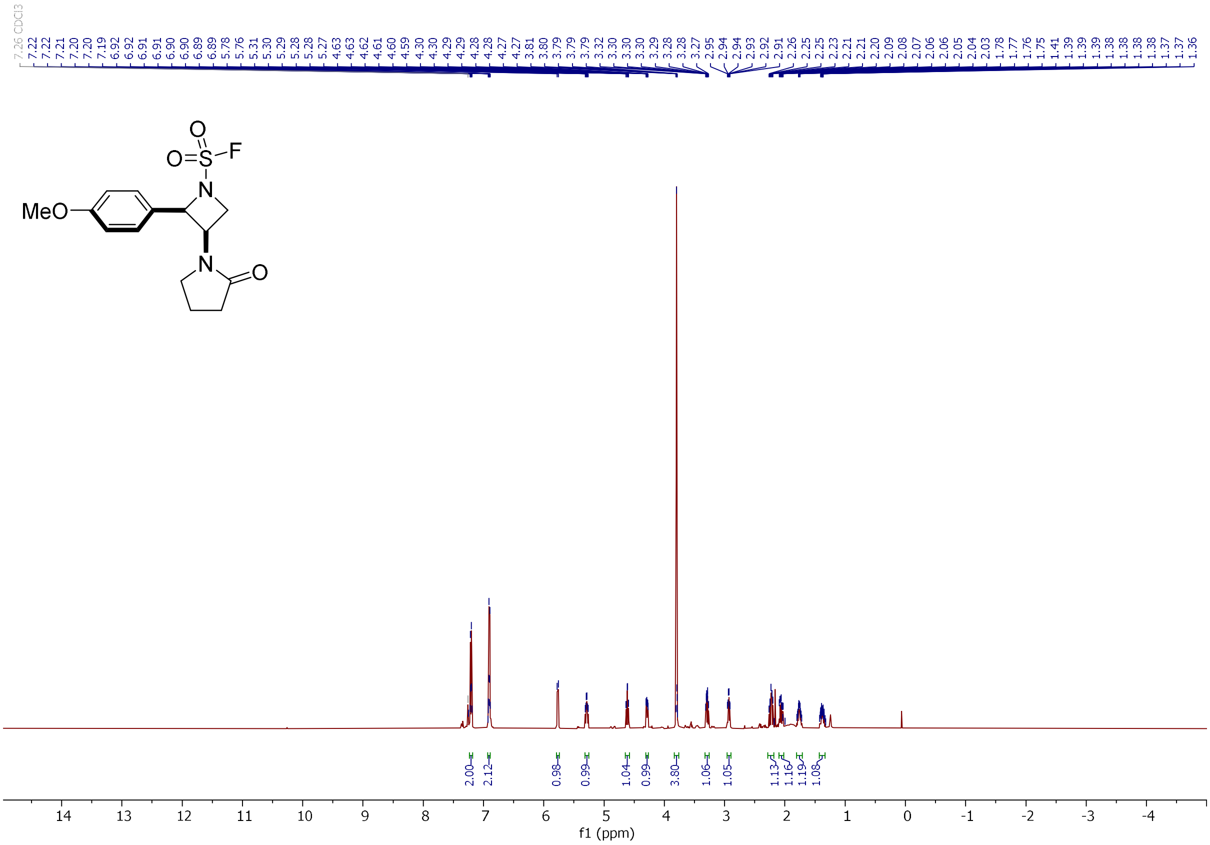

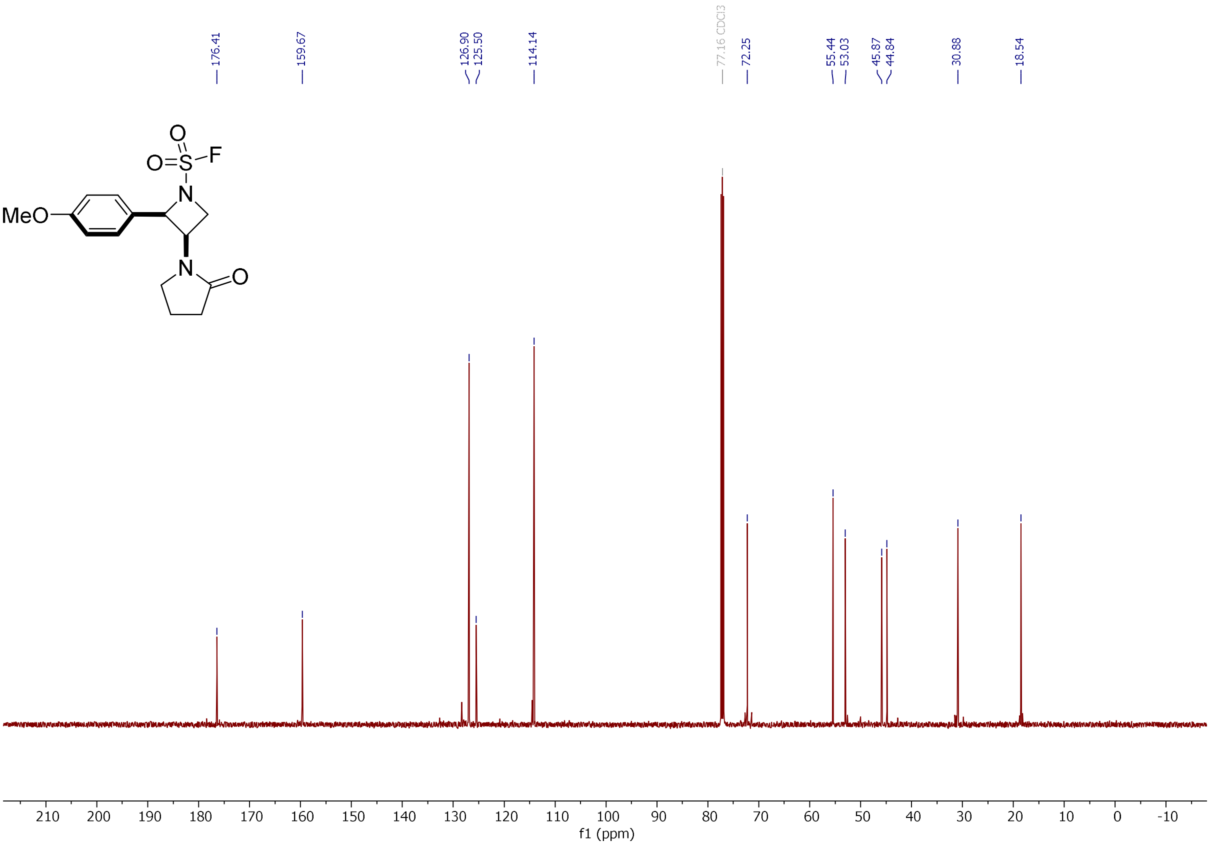

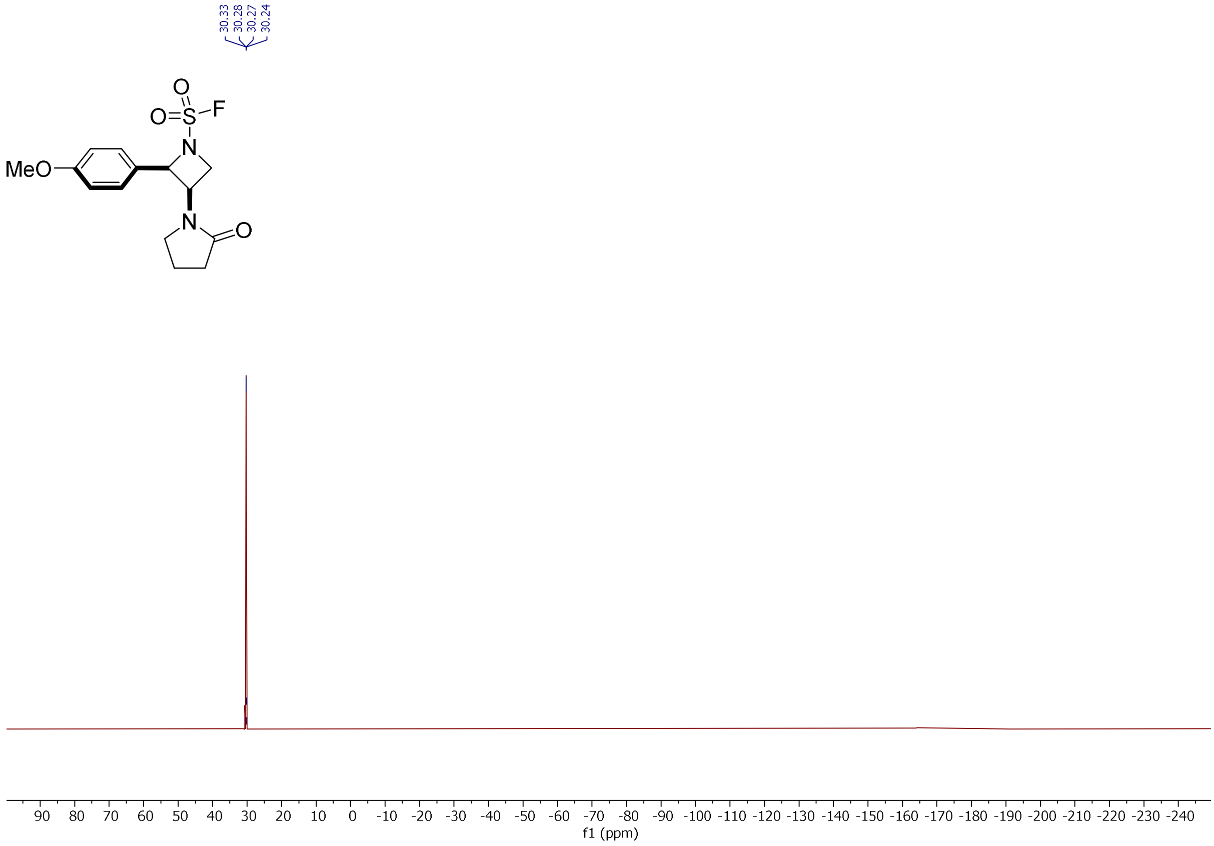

(2*S**,3*R**)-2-(4-Methoxyphenyl)-3-(2-oxopyrrolidin-1-yl)azetidine-1-sulfonyl fluoride (4t')

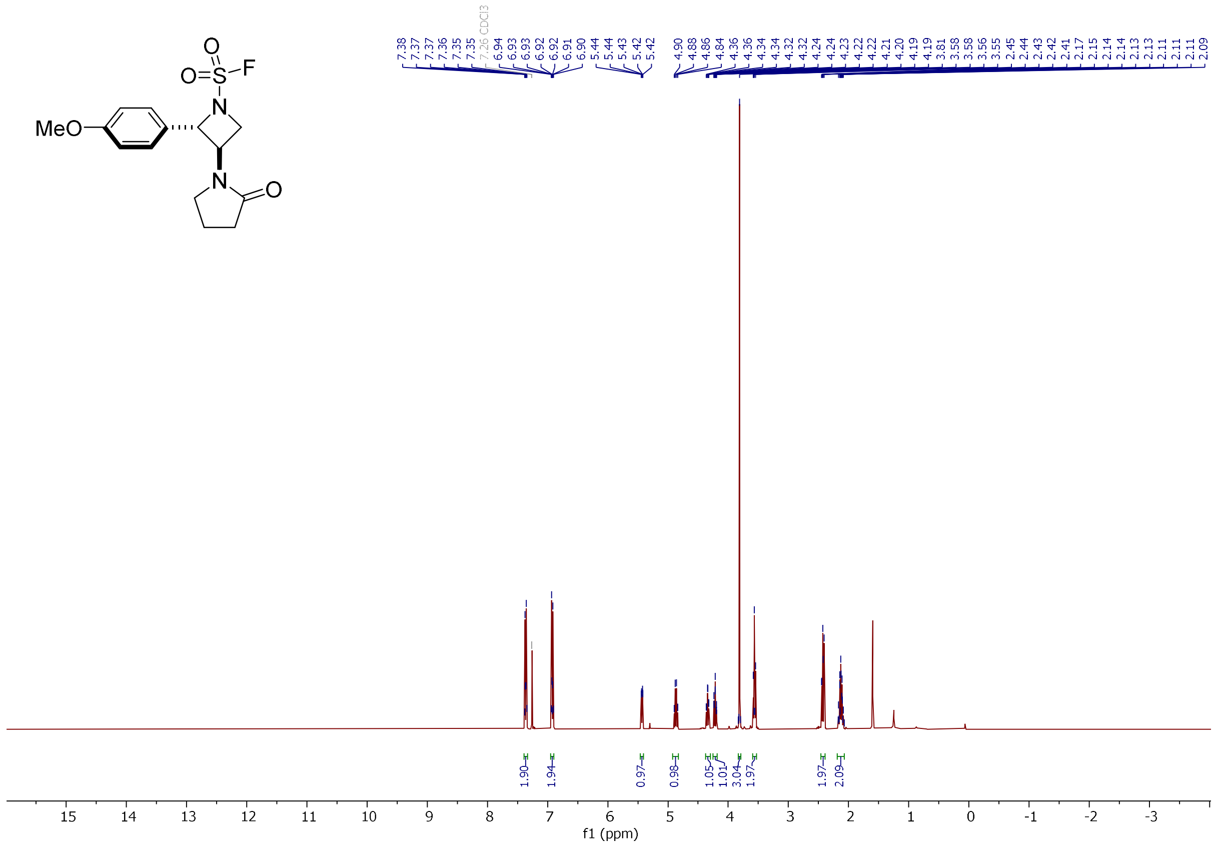

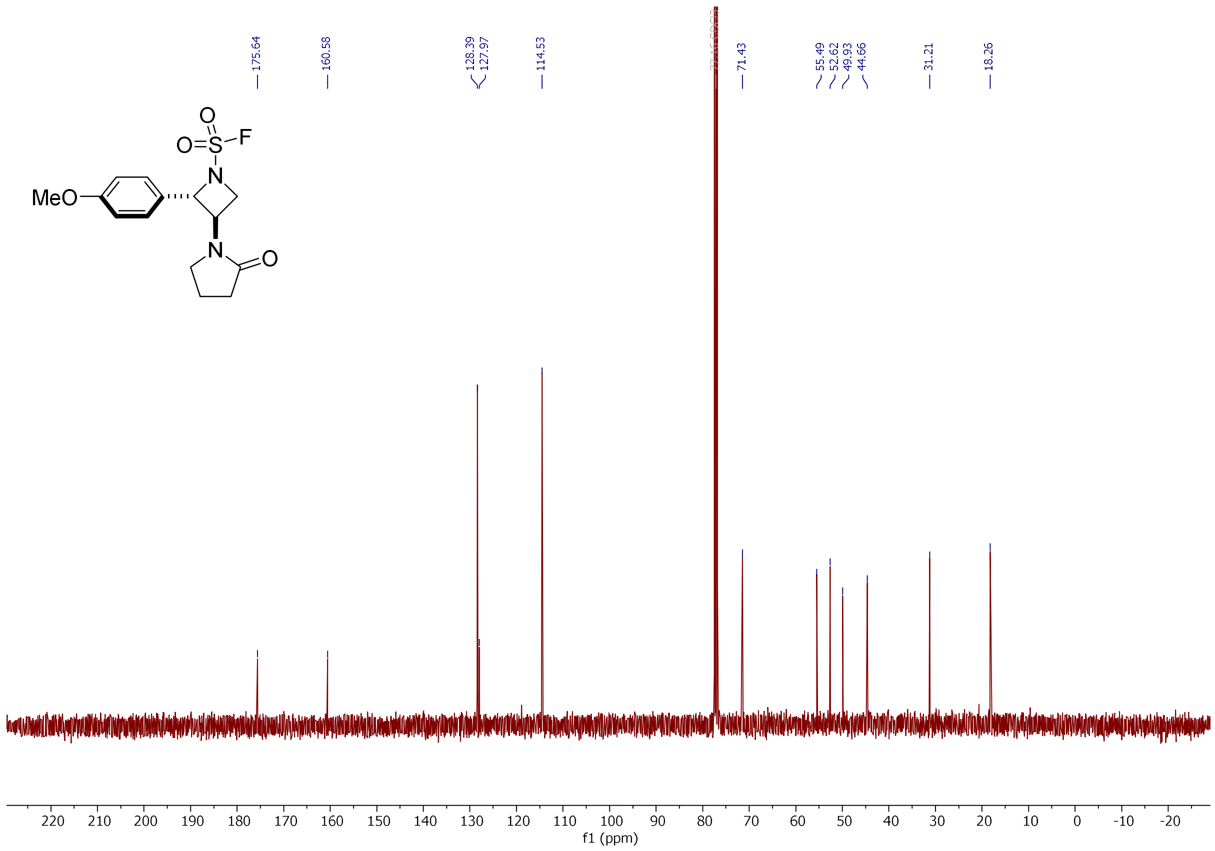
175.64
160.58
128.39
127.97
114.53
71.43
55.49
52.62
49.93
44.66
31.21
18.26
MeO
O
O=S-F
N
N
O
220 210 200 190 180 170 160 150 140 130 120 110 100 90 80 70 60 50 40 30 20 10 0 -10 -20
f1 (ppm)

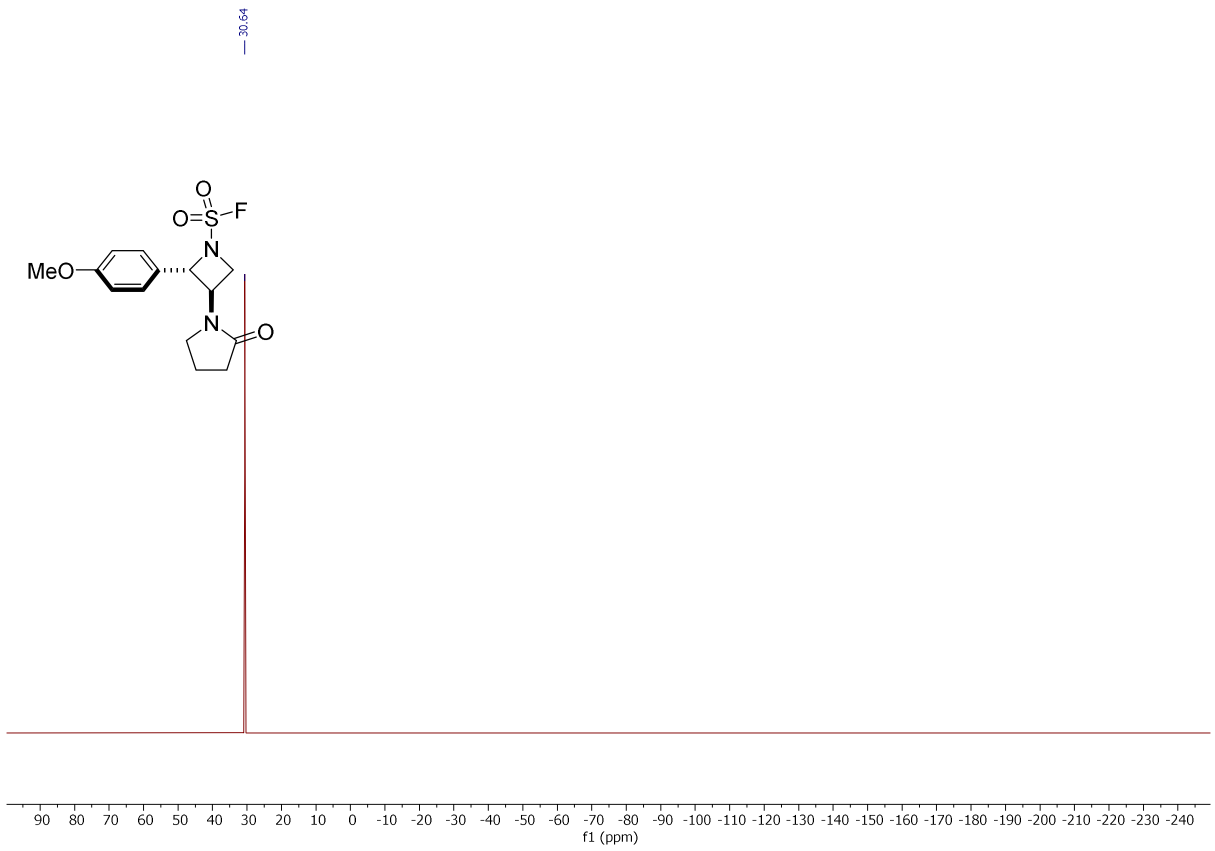
30.64
MeO
O
O=S-F
N
N
O
90 80 70 60 50 40 30 20 10 0 -10 -20 -30 -40 -50 -60 -70 -80 -90 -100 -110 -120 -130 -140 -150 -160 -170 -180 -190 -200 -210 -220 -230 -240
f1 (ppm)

(2*R**,3*R**)-3-(1H-Imidazol-1-yl)-2-(4-methoxyphenyl)azetidine-1-sulfonyl fluoride (4u)

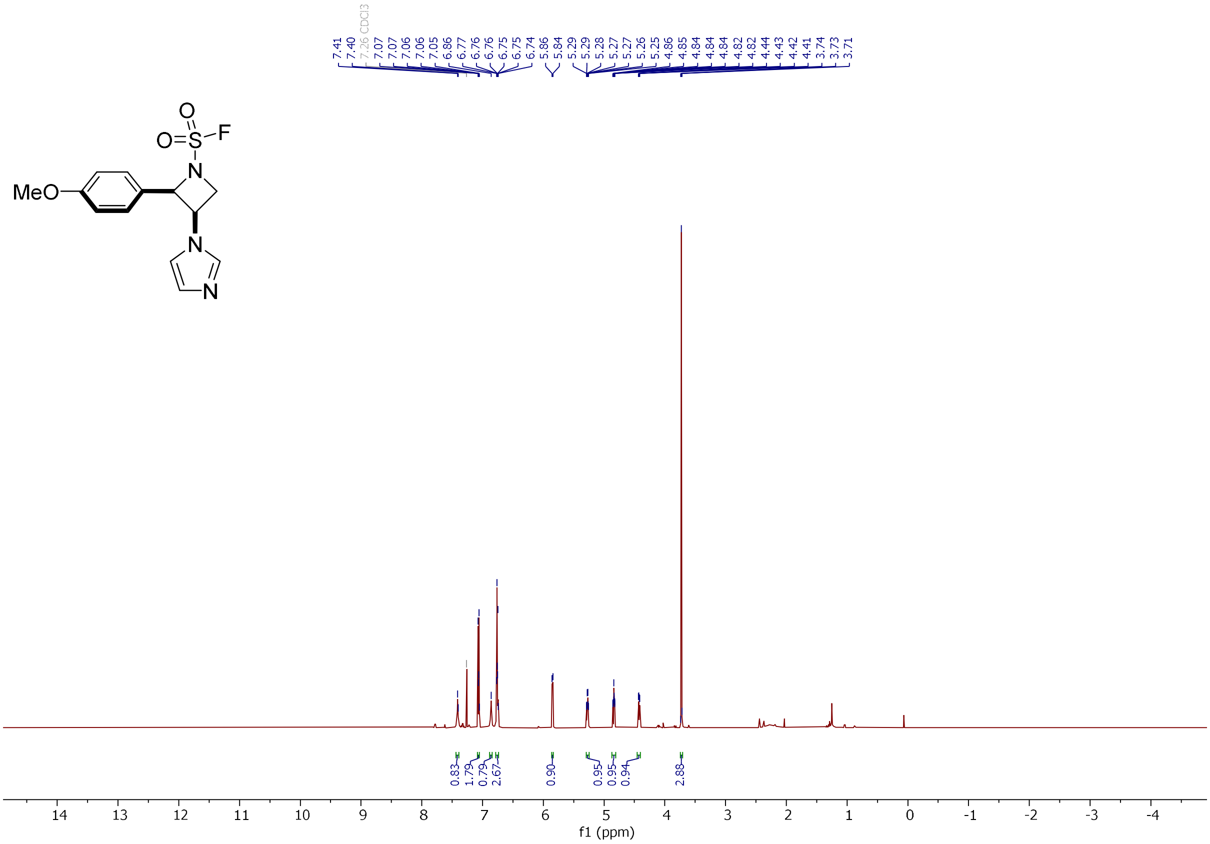

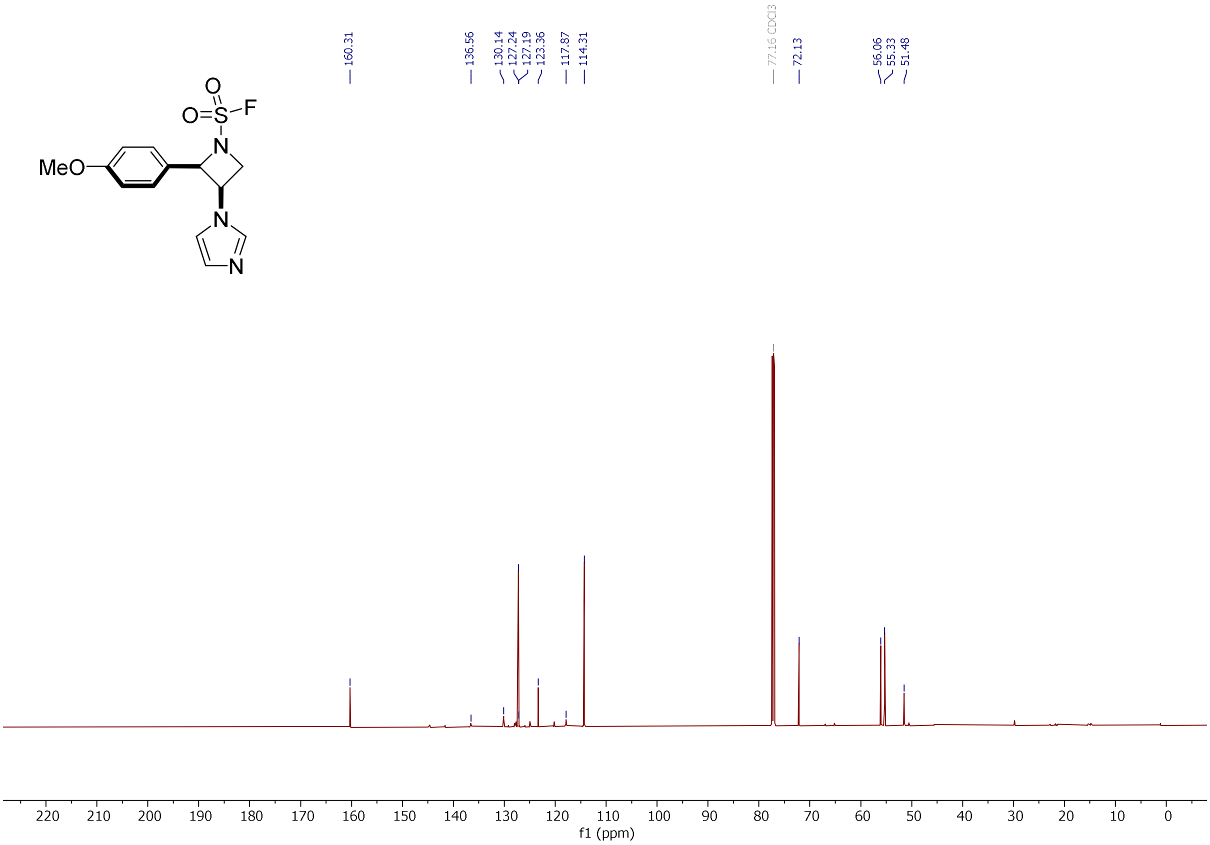
160.31
136.56
130.14
127.24
127.19
123.36
117.87
114.31
77.16 CDCl3
72.13
56.06
55.33
51.48
MeO
O
O=S-F
N
N
N
220 210 200 190 180 170 160 150 140 130 120 110 100 90 80 70 60 50 40 30 20 10 0
f1 (ppm)

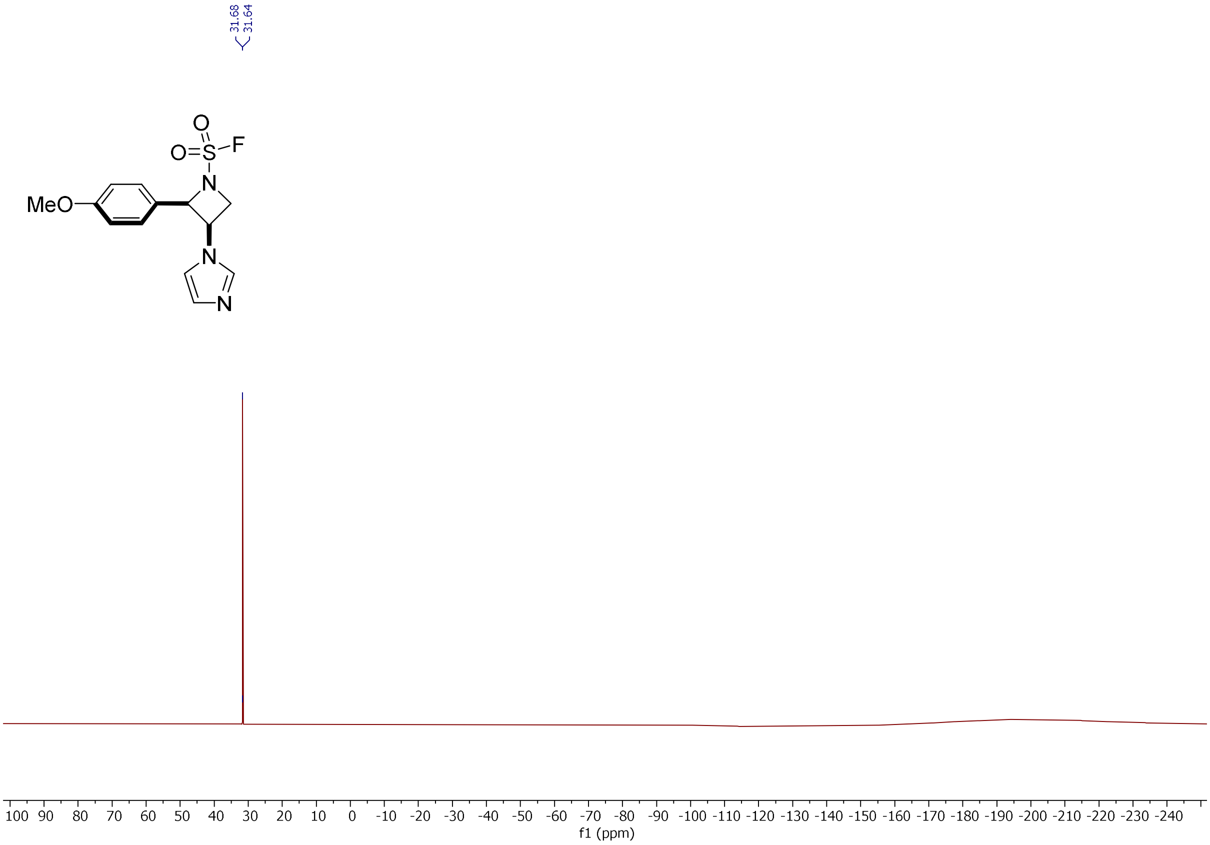
31.68
31.64
MeO
O
O=S-F
N
N
N
100 90 80 70 60 50 40 30 20 10 0 -10 -20 -30 -40 -50 -60 -70 -80 -90 -100 -110 -120 -130 -140 -150 -160 -170 -180 -190 -200 -210 -220 -230 -240
f1 (ppm)

(2R*,3S*)-3-(1H-Imidazol-1-yl)-2-(4-methoxyphenyl)azetidine-1-sulfonyl fluoride (4u’)

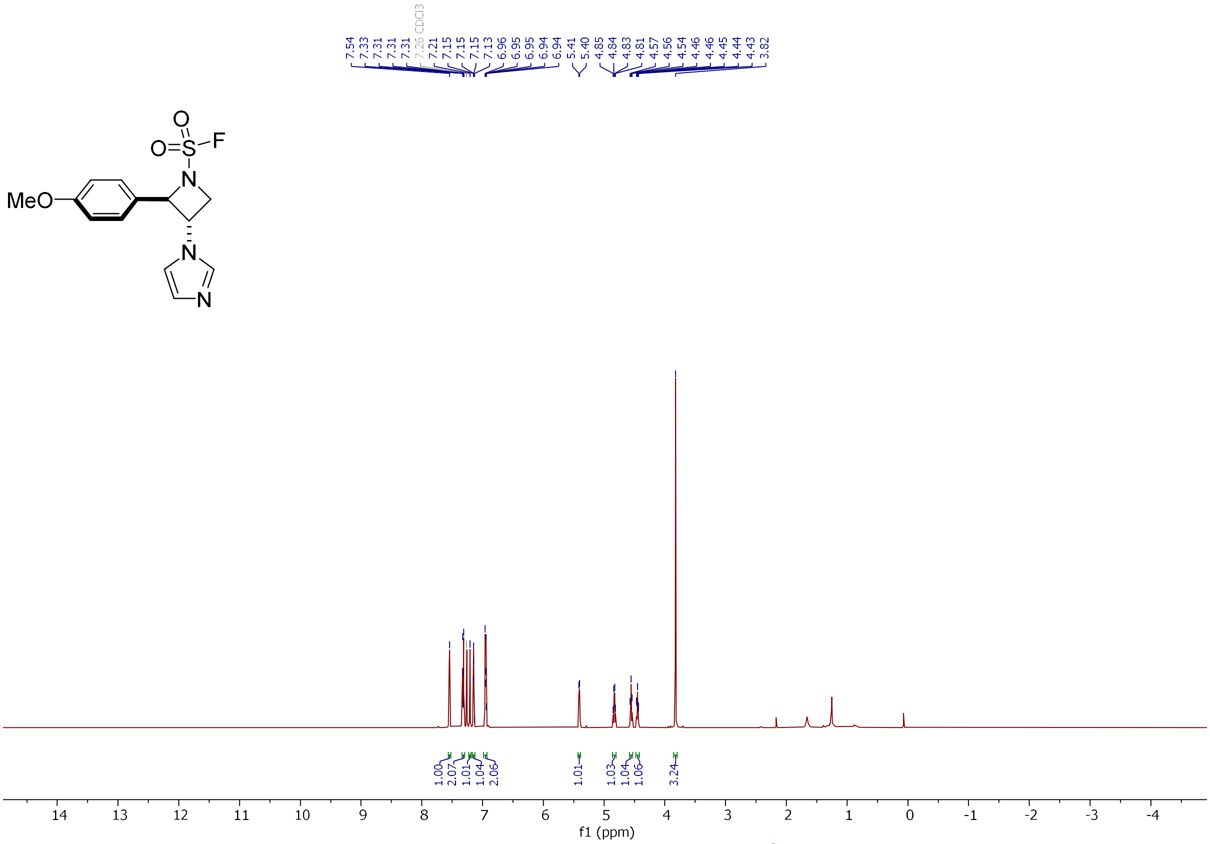

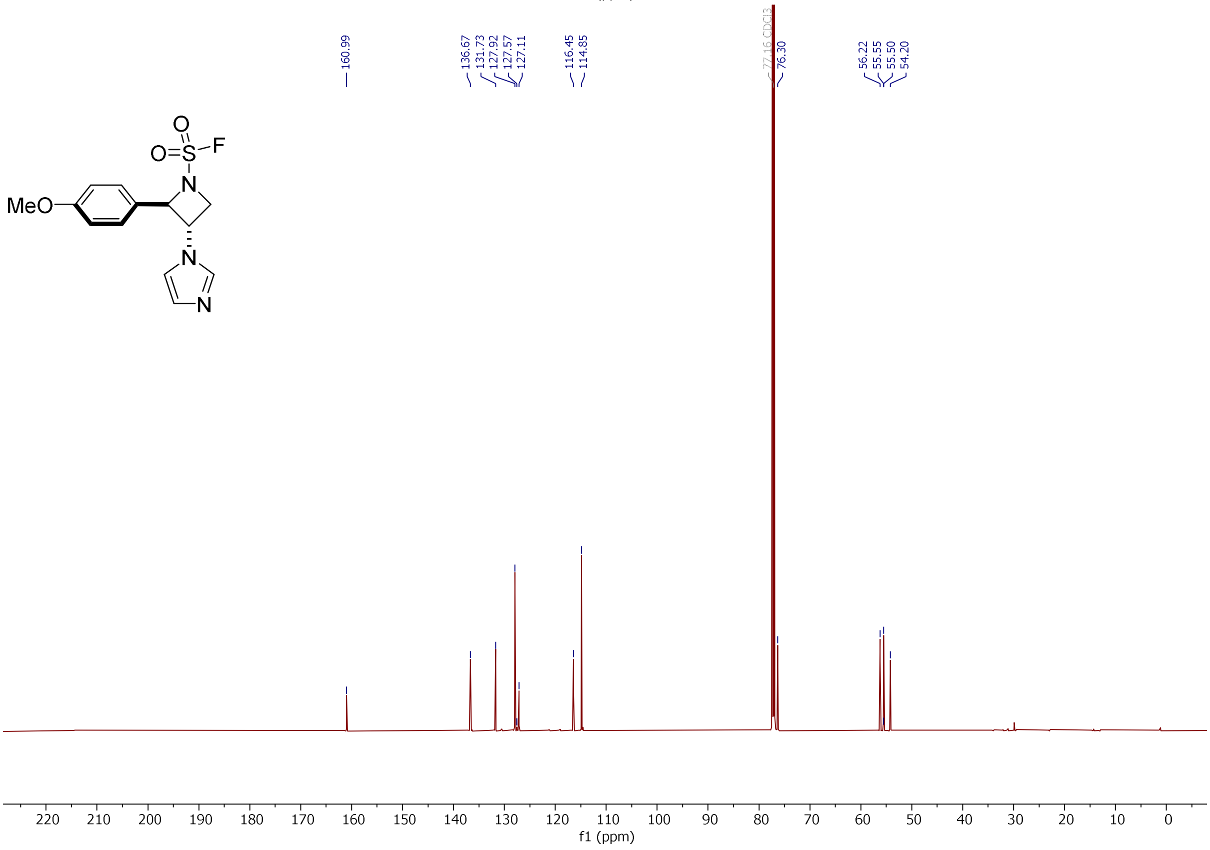

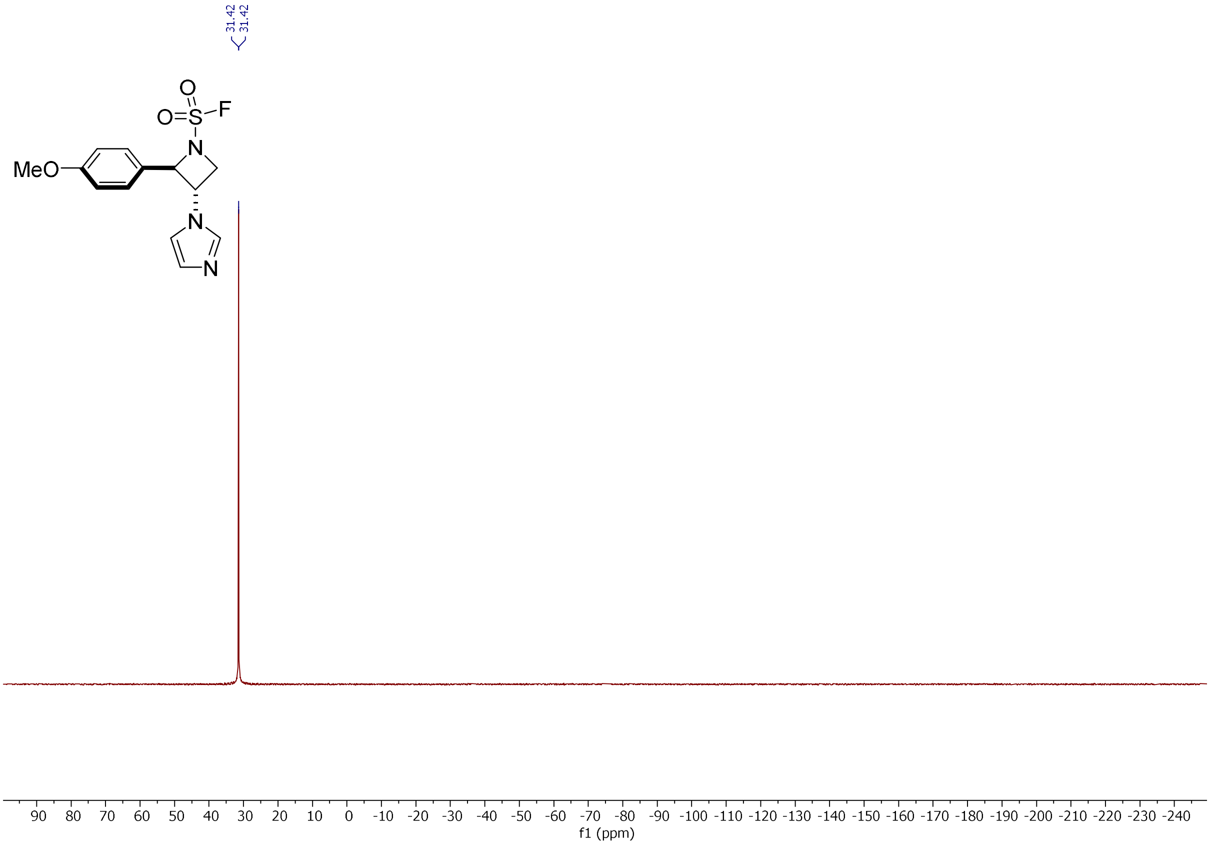
31.42
31.42
MeO
O
O=S
F
N
N
N
90 80 70 60 50 40 30 20 10 0 -10 -20 -30 -40 -50 -60 -70 -80 -90 -100 -110 -120 -130 -140 -150 -160 -170 -180 -190 -200 -210 -220 -230 -240
f1 (ppm)

(2*S**,3*S**)-3-(3,5-Dimethyl-1H-pyrazol-1-yl)-2-(4-methoxyphenyl)azetidine-1-sulfonyl fluoride (4v)

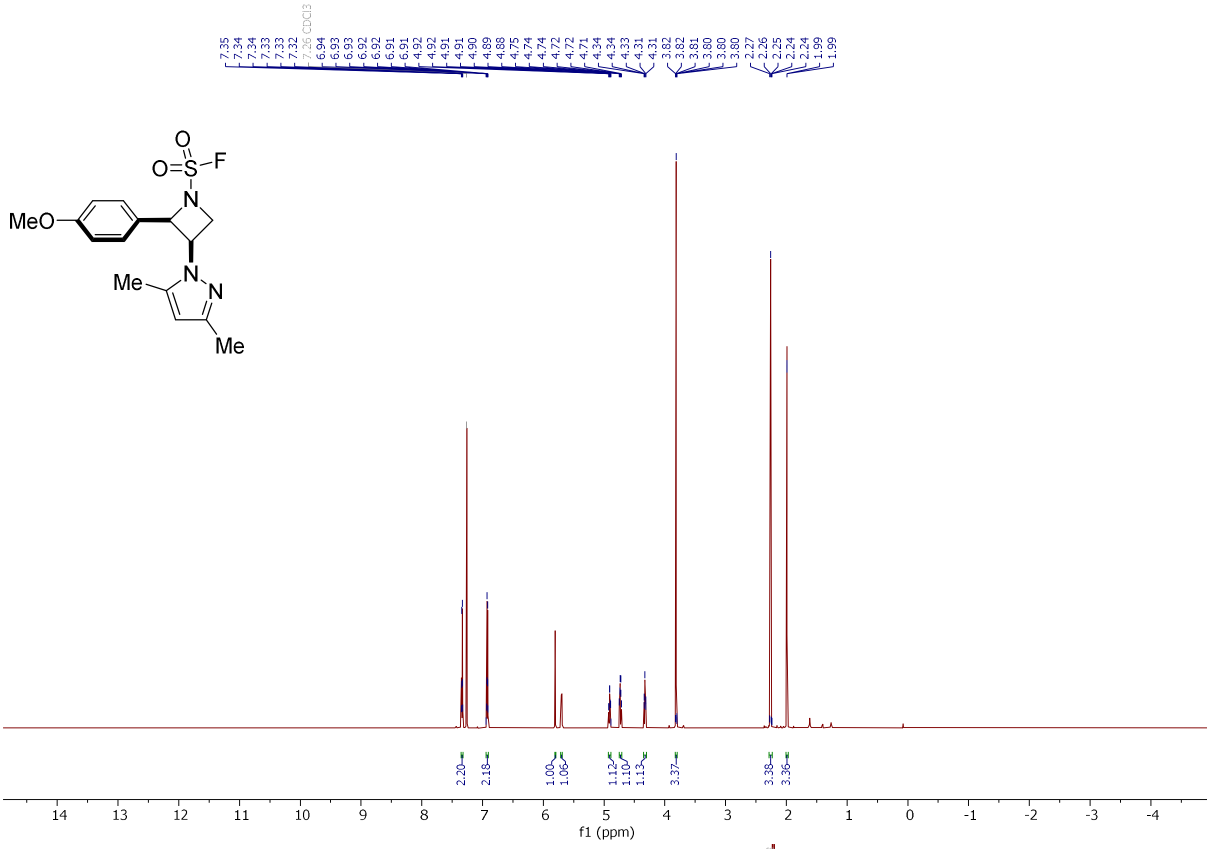

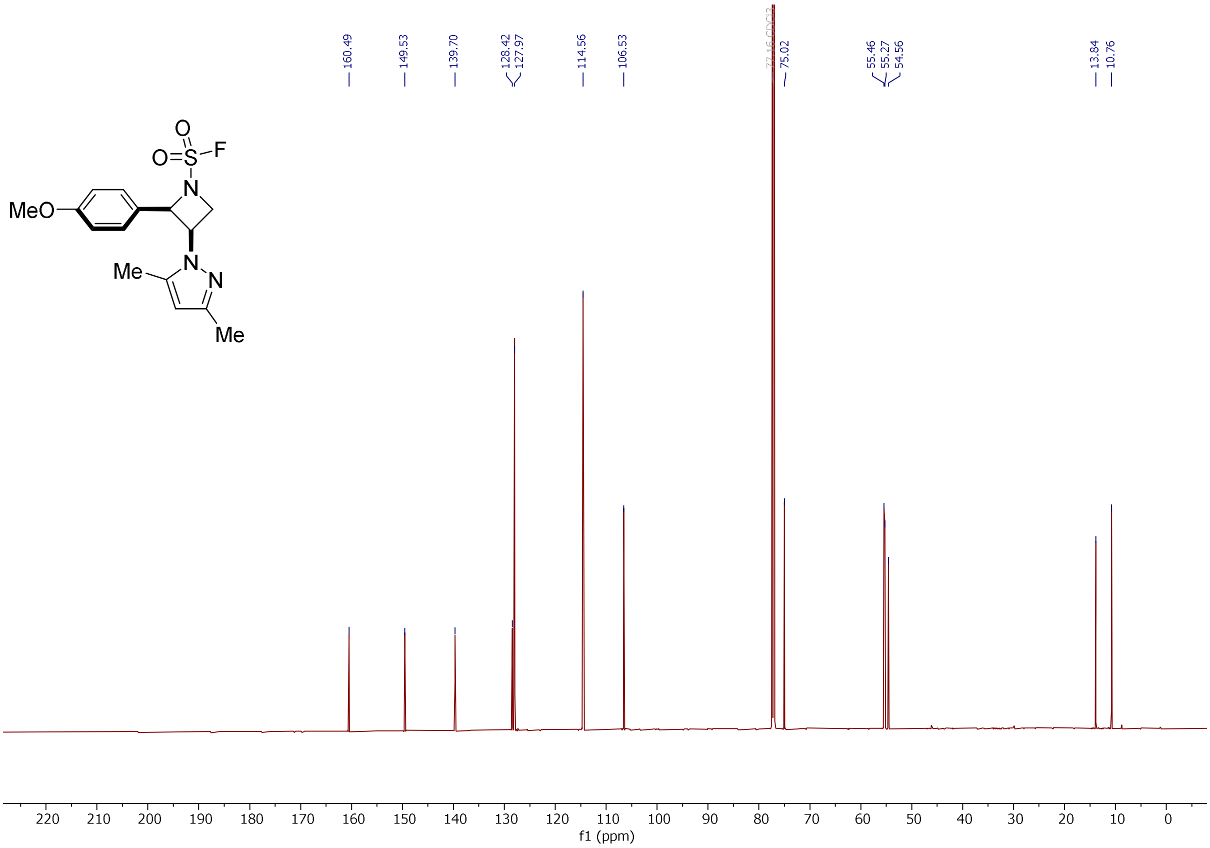

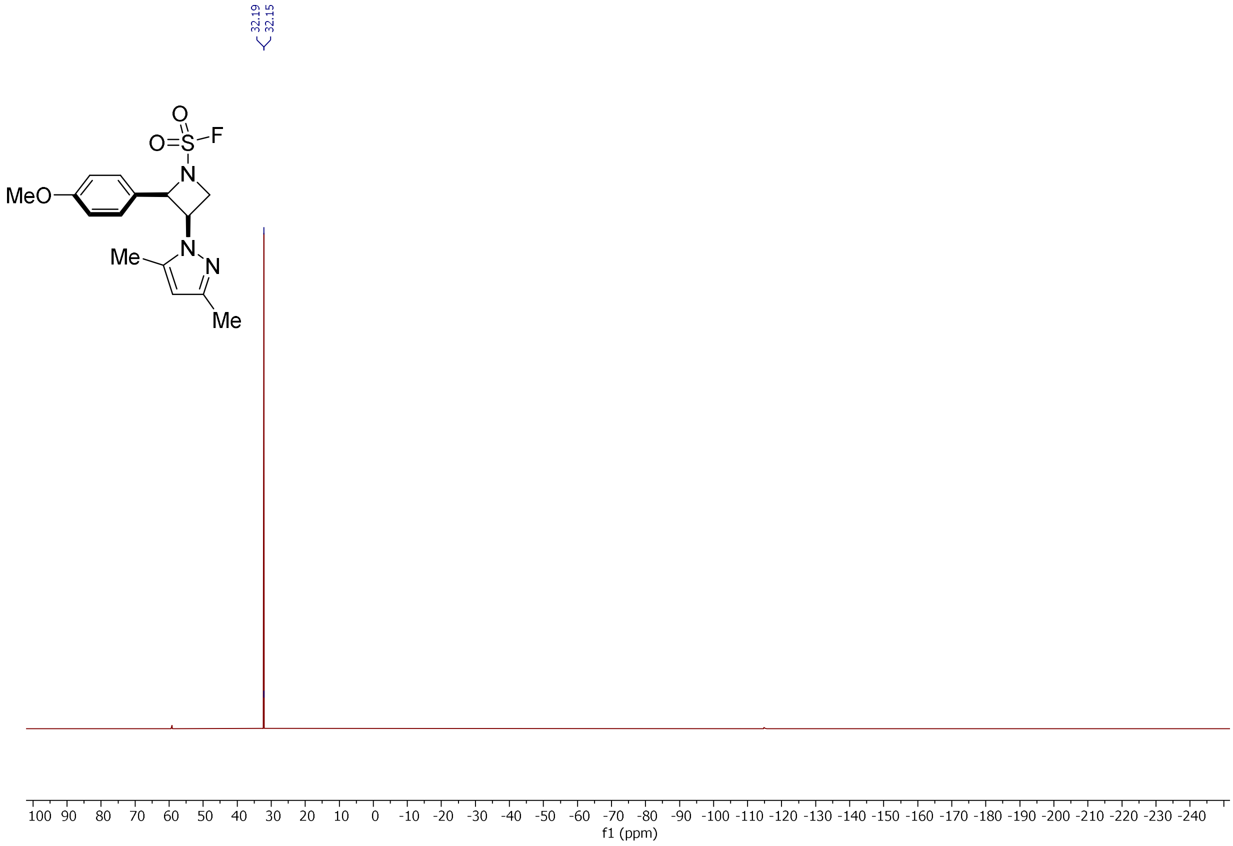

(2*S**,3*R**)-3-(3,5-Dimethyl-1H-pyrazol-1-yl)-2-(4-methoxyphenyl)azetidine-1-sulfonyl fluoride (4v’)

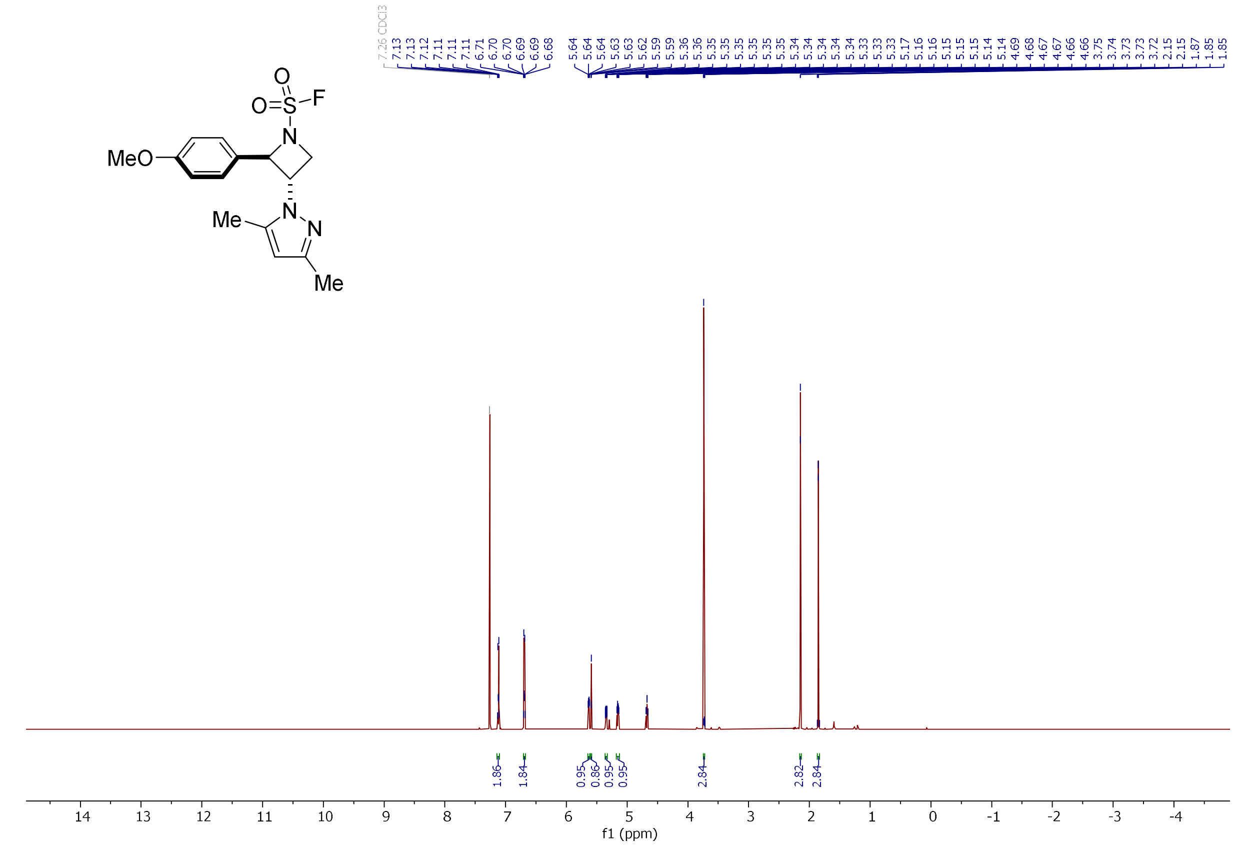

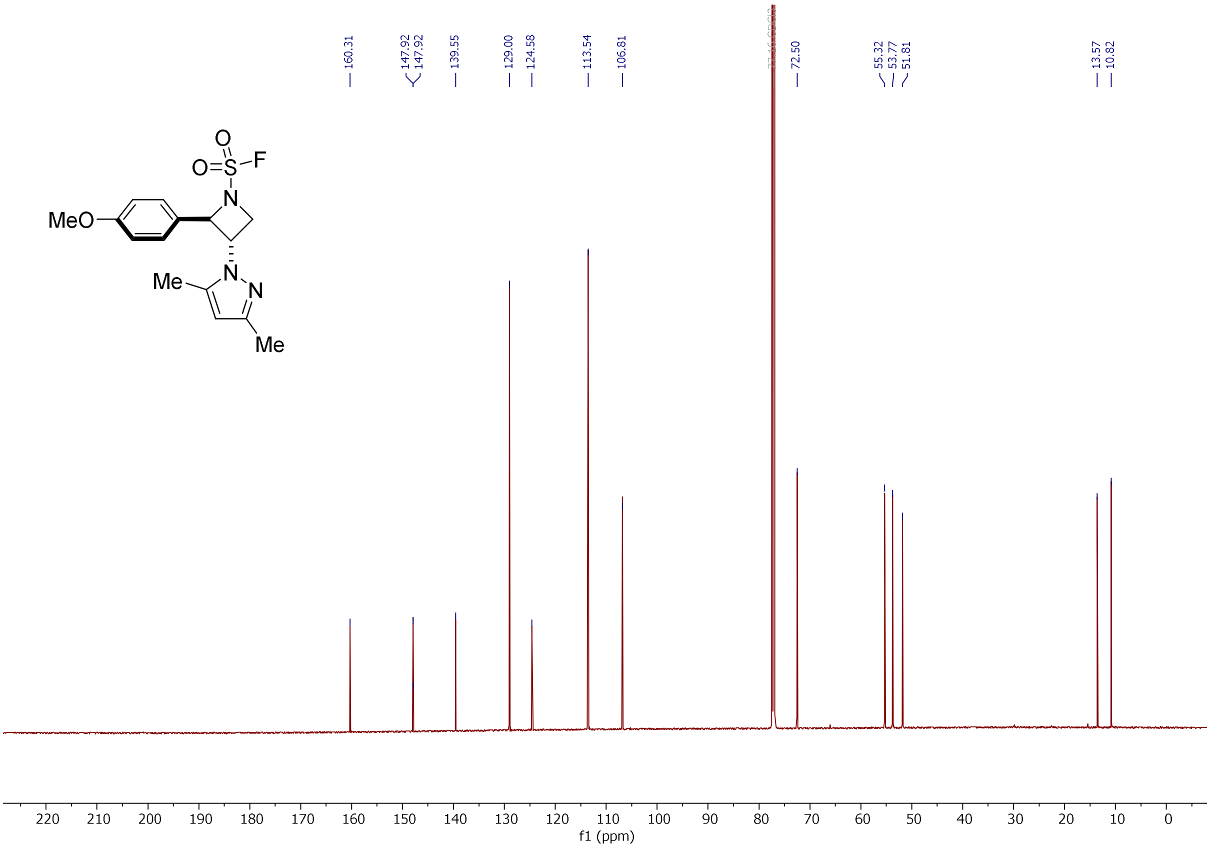
160.31
147.92
147.92
139.55
129.00
124.58
113.54
106.81
72.50
55.32
53.77
51.81
13.57
10.82
O
O=S-F
N
MeO
N
Me
N
Me
220 210 200 190 180 170 160 150 140 130 120 110 100 90 80 70 60 50 40 30 20 10 0
f1 (ppm)

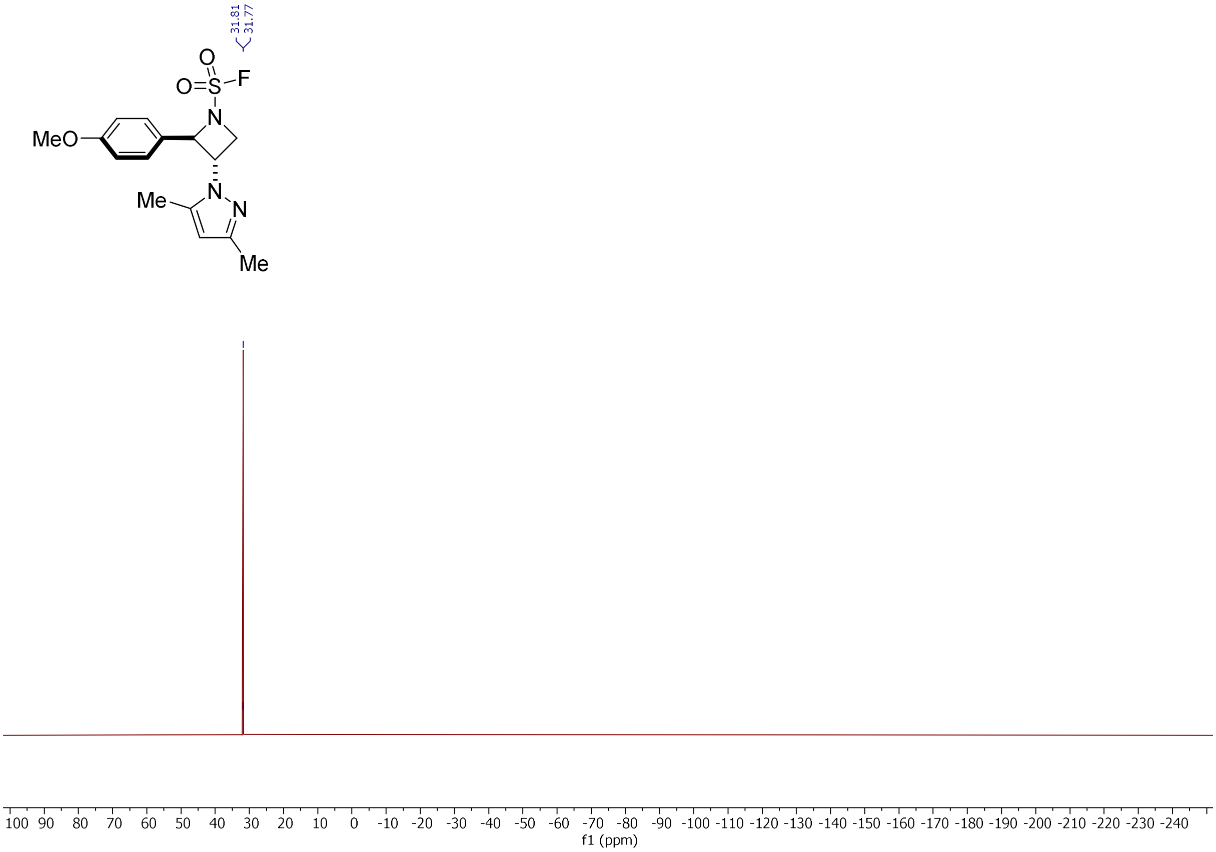
31.81
31.77
O
O=S-F
N
MeO
N
Me
N
Me
100 90 80 70 60 50 40 30 20 10 0 -10 -20 -30 -40 -50 -60 -70 -80 -90 -100 -110 -120 -130 -140 -150 -160 -170 -180 -190 -200 -210 -220 -230 -240
f1 (ppm)

Methyl (2*R**,3*S**)-3-acetamide-1-(fluorosulfonyl)-2-(4-methoxyphenyl)azetidine-3-carboxylate (4w) and Methyl (2*S**,3*S**)-3-acetamide-1-(fluorosulfonyl)-2-(4-methoxyphenyl)azetidine-3-carboxylate (4w')

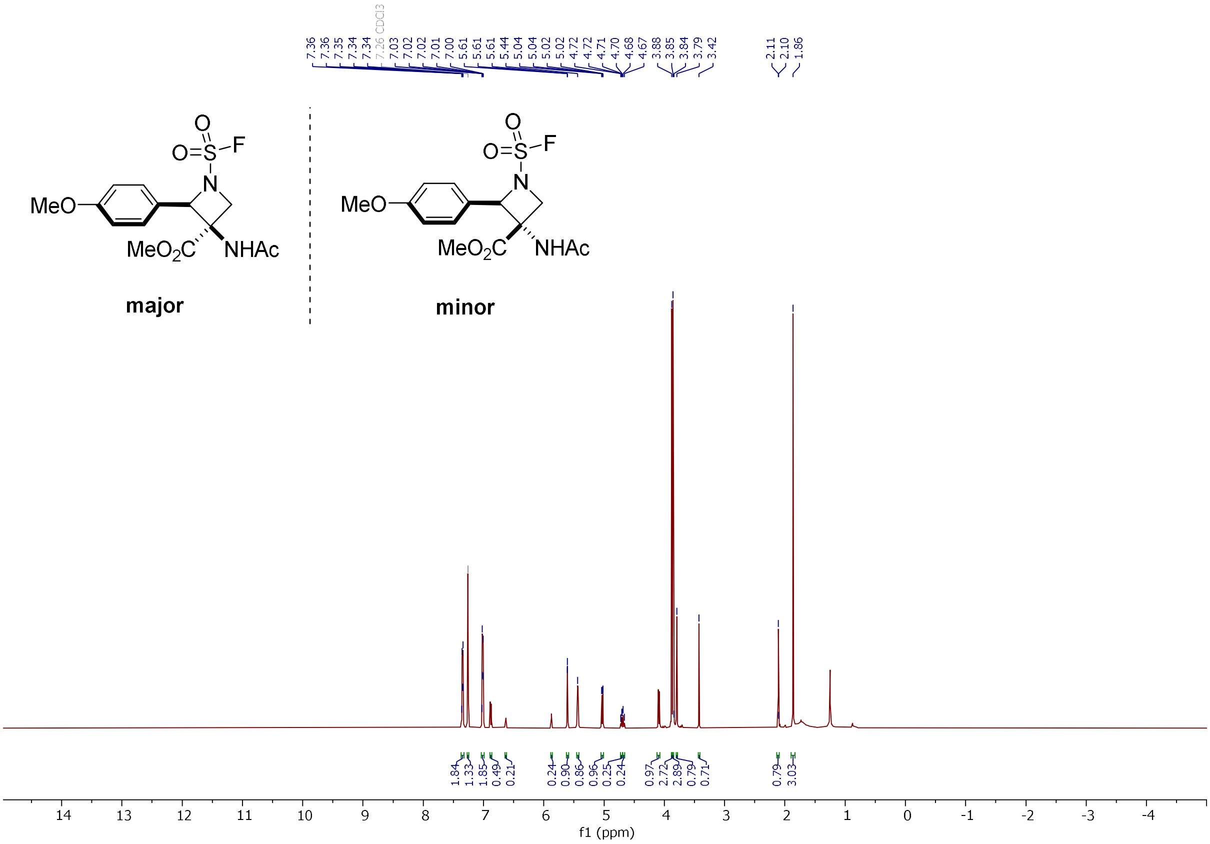

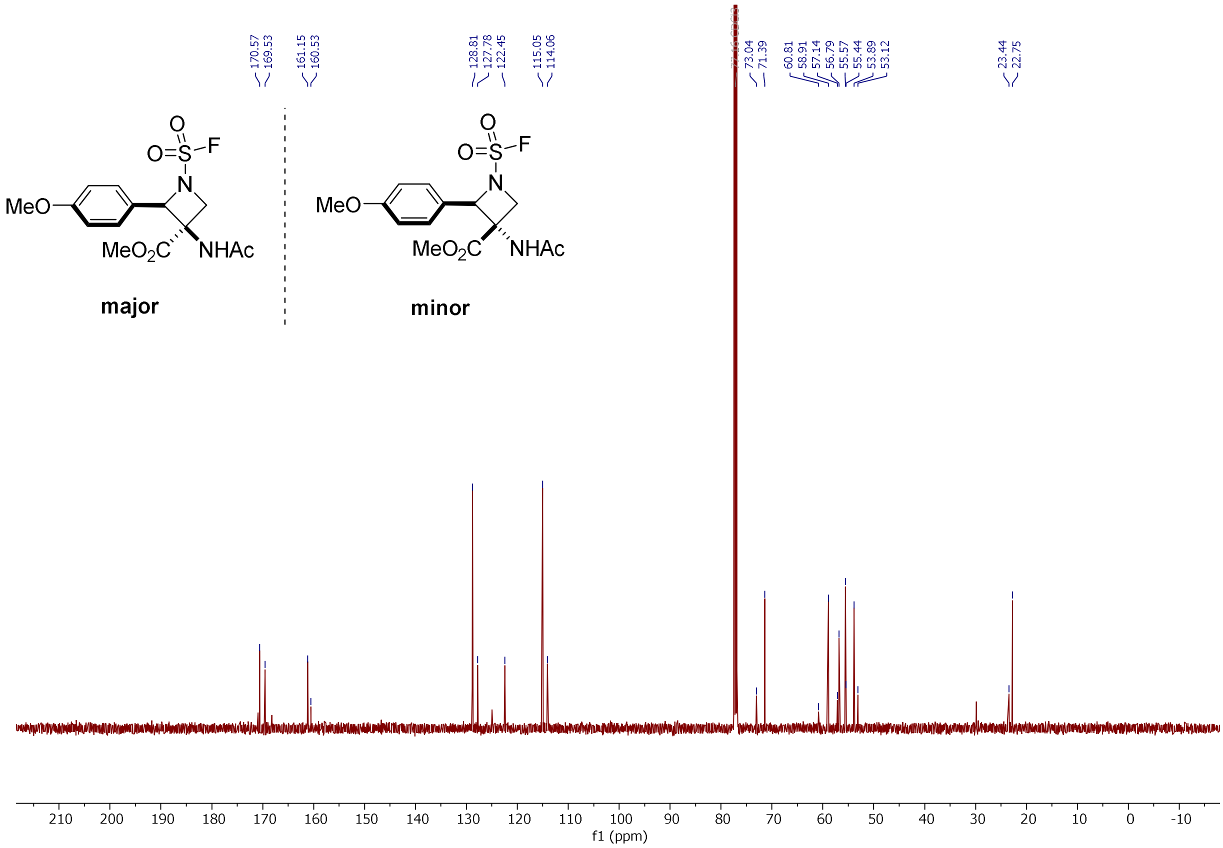

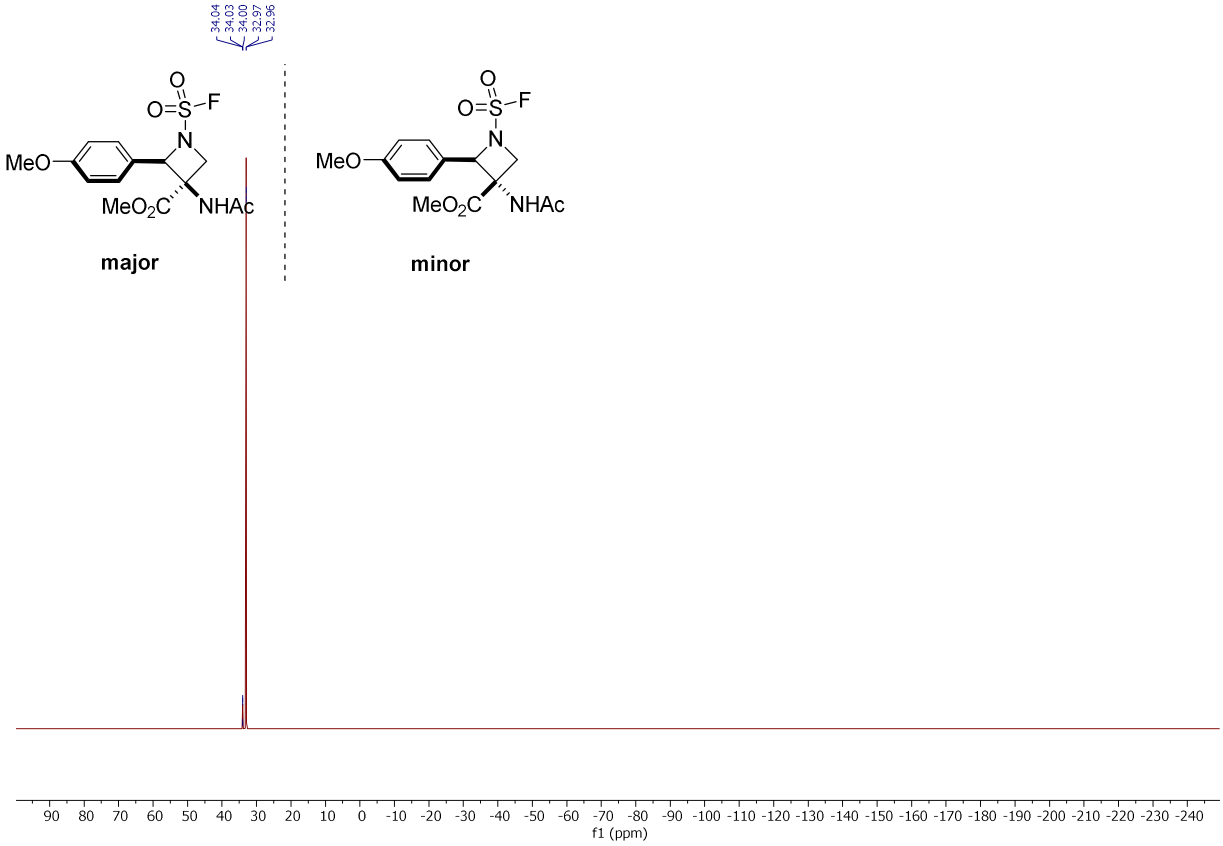
34.04
34.03
34.00
32.97
32.96
MeO
O=S-F
N
MeO2C
NHAc
major
minor
90 80 70 60 50 40 30 20 10 0 -10 -20 -30 -40 -50 -60 -70 -80 -90 -100 -110 -120 -130 -140 -150 -160 -170 -180 -190 -200 -210 -220 -230 -240
f1 (ppm)

Tert-butyl (tert-butoxycarbonyl)((2*R**,3*R**)-1-(fluorosulfonyl)-2-(4-methoxyphenyl)azetidin-3-yl)carbamate (4x) and Tert-butyl (tert-butoxycarbonyl)((2*R**,3*S**)-1-(fluorosulfonyl)-2-(4-methoxyphenyl)azetidin-3-yl)carbamate (4x')

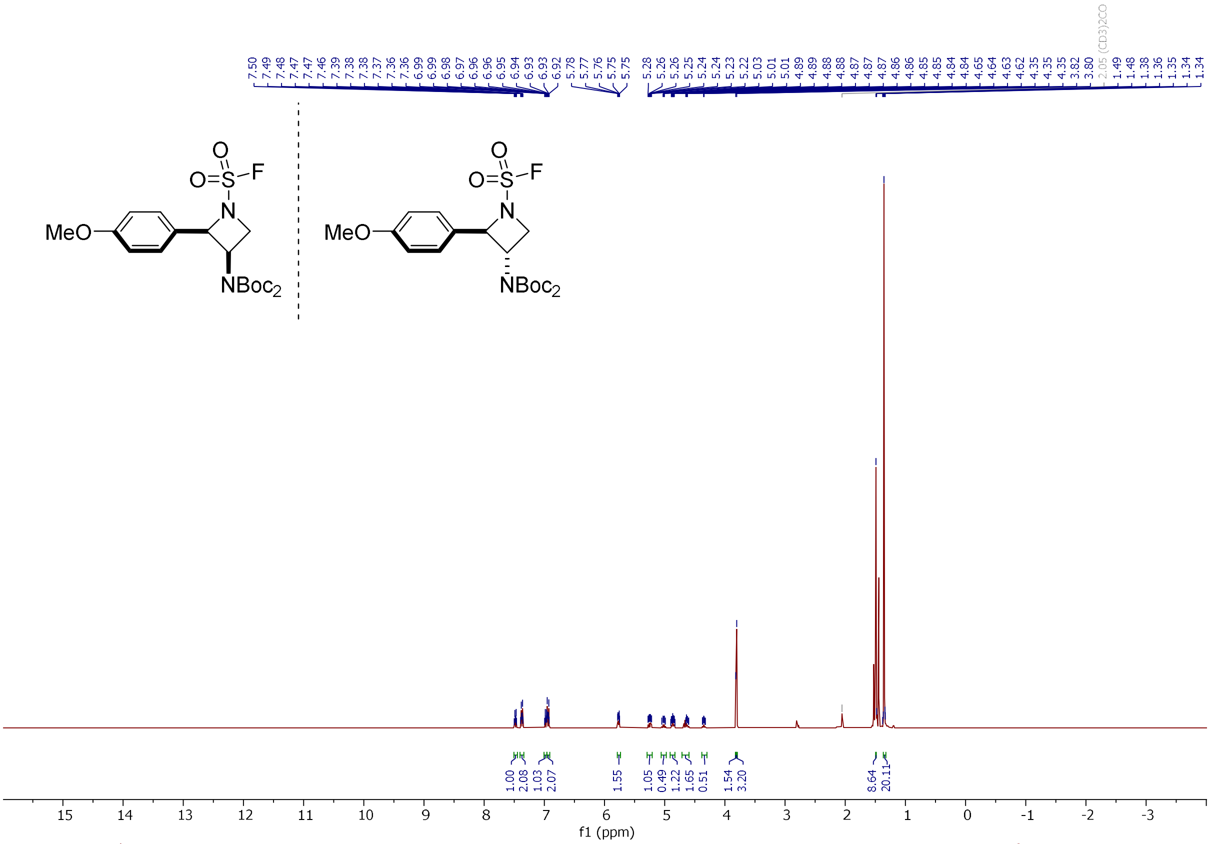

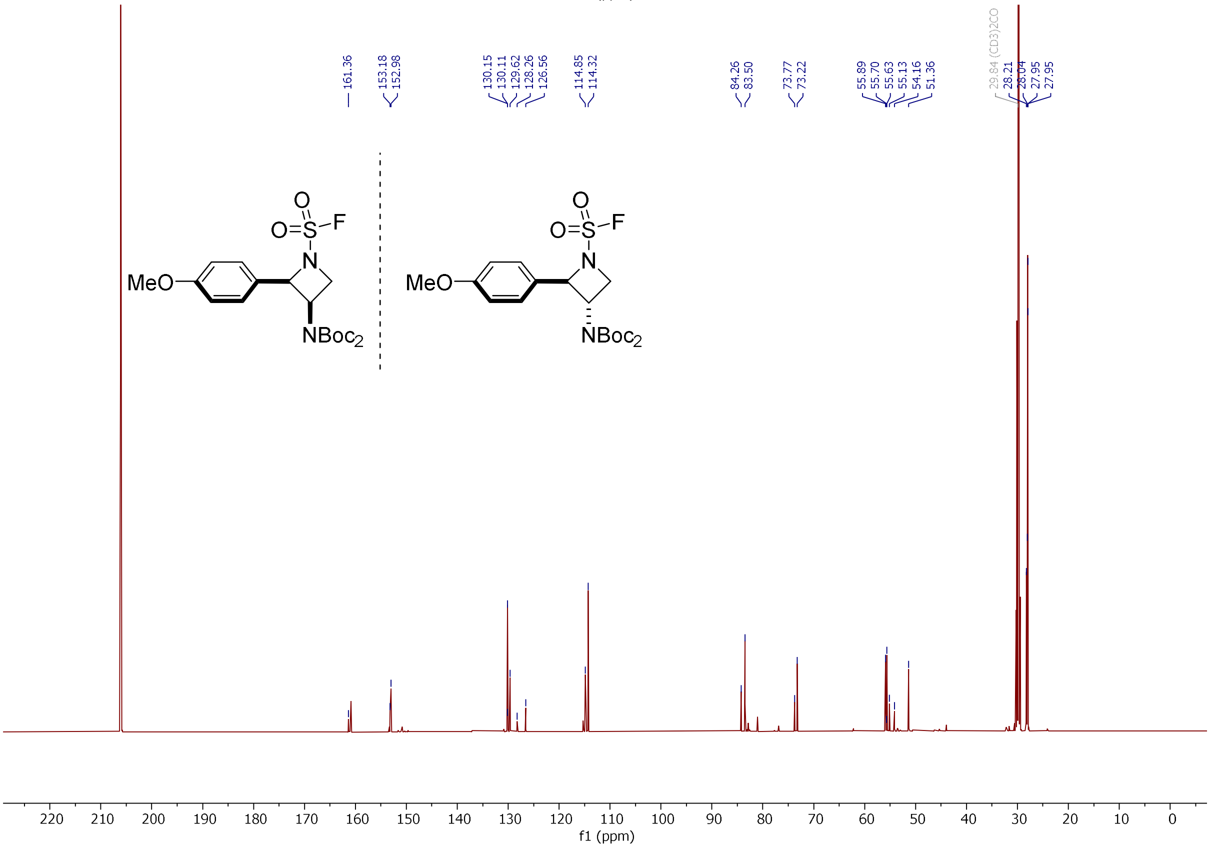

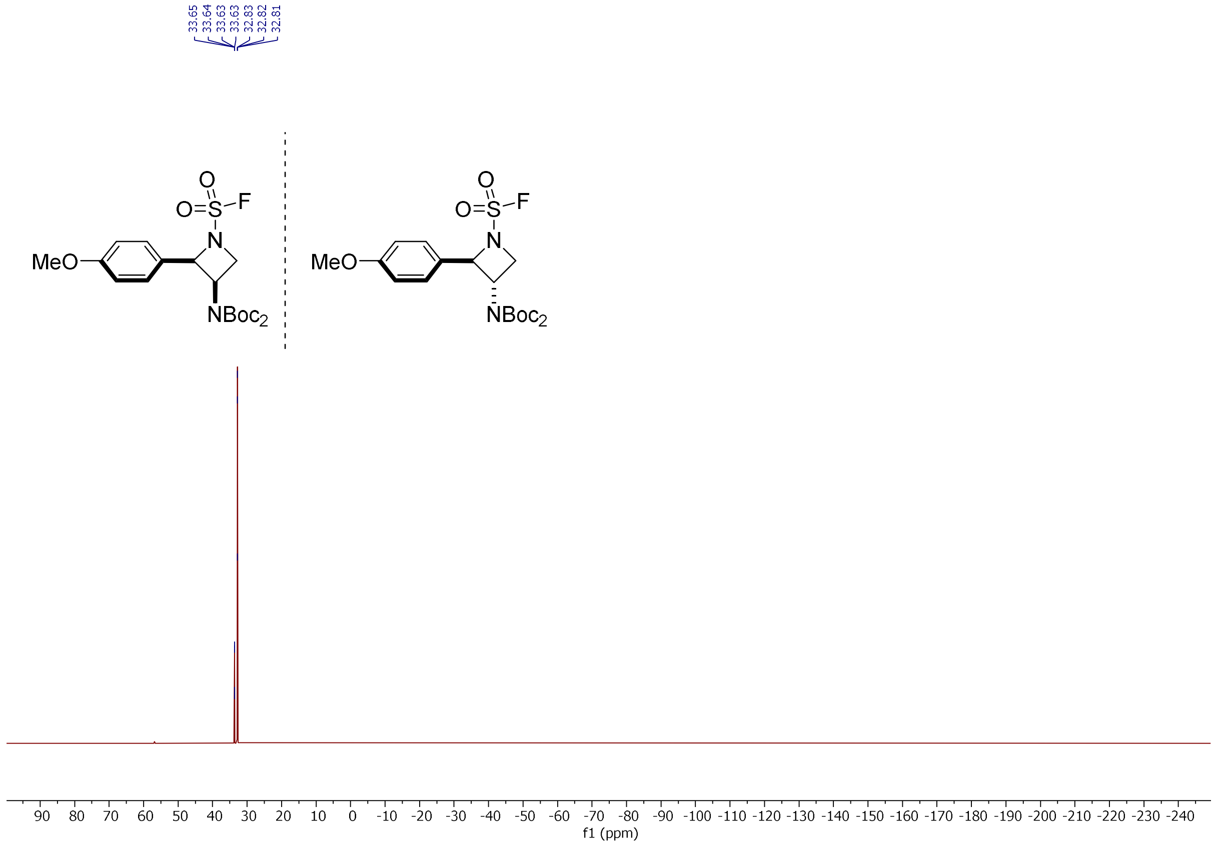

(2*R**,3*S**)-3-(1,3-Dioxoisoindolin-2-yl)-2-(4-methoxyphenyl)azetidine-1-sulfonyl fluoride (4y)

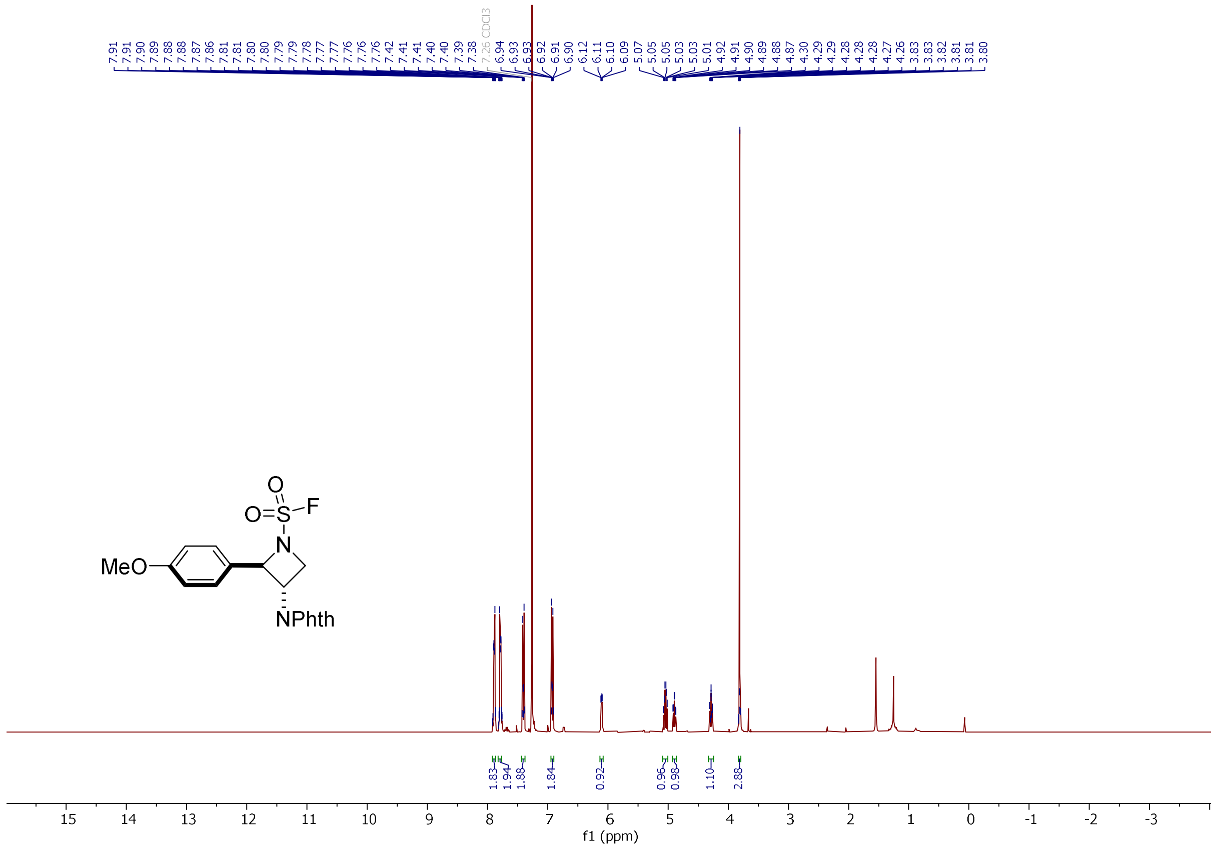

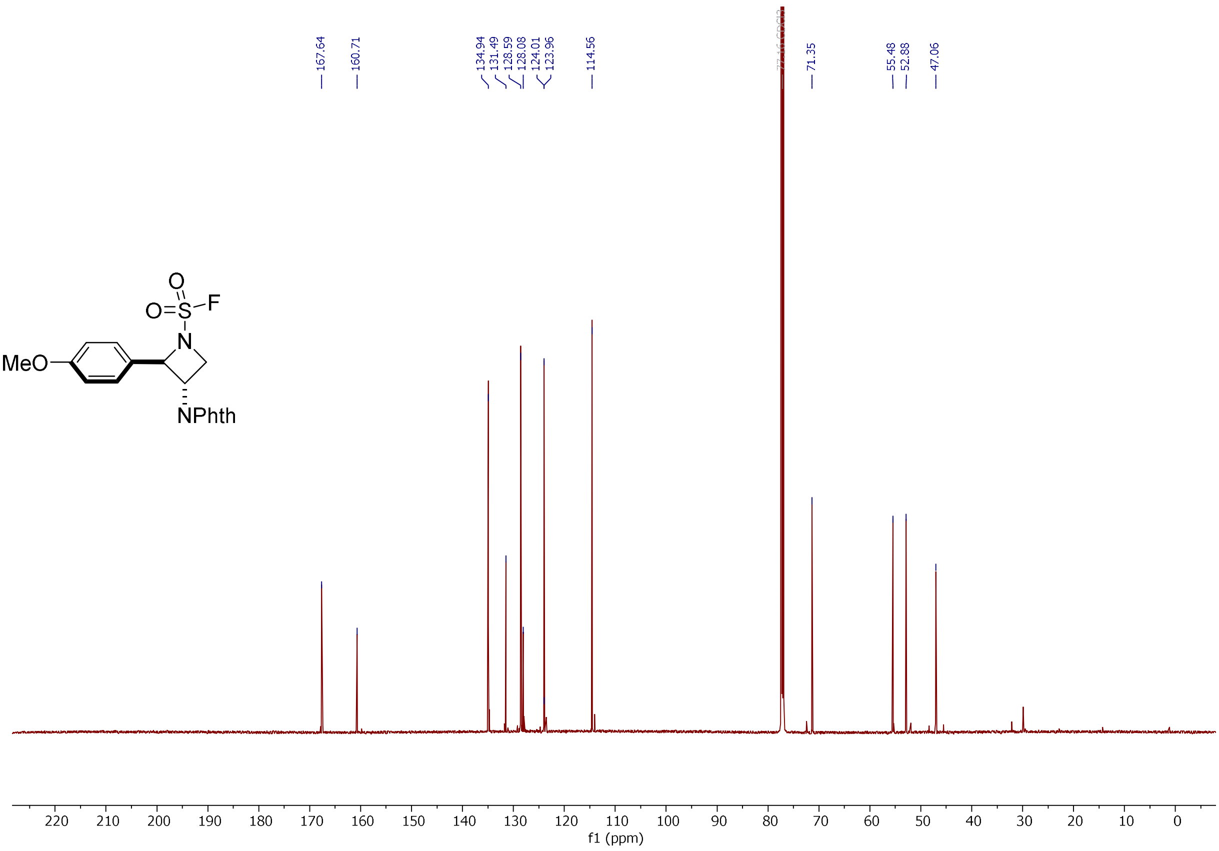

167.64
160.71
134.94
131.49
128.59
128.08
124.01
123.96
114.56
71.35
55.48
52.88
47.06
O
O=S–F
N
MeO
NPhth
220 210 200 190 180 170 160 150 140 130 120 110 100 90 80 70 60 50 40 30 20 10 0
f1 (ppm)

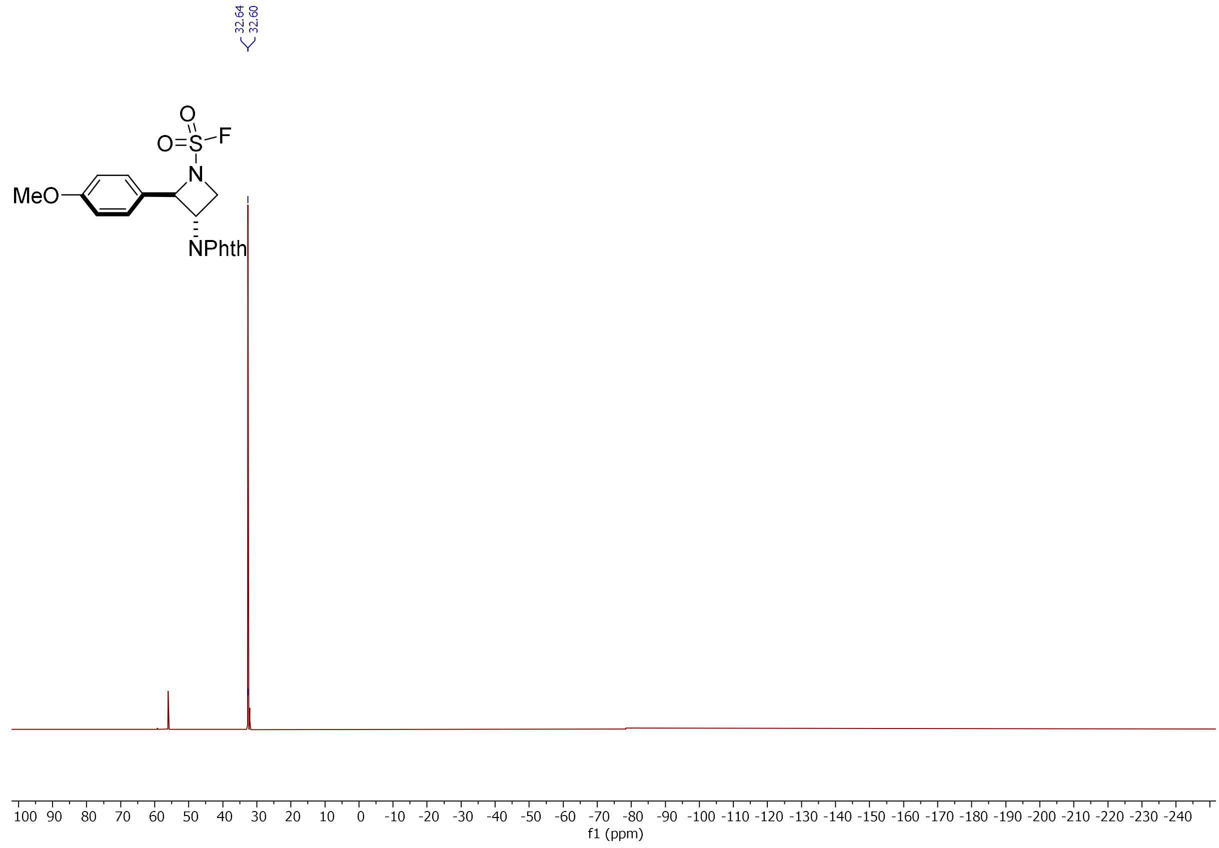

32.64
32.60
O
O=S–F
N
MeO
NPhth
100 90 80 70 60 50 40 30 20 10 0 -10 -20 -30 -40 -50 -60 -70 -80 -90 -100 -110 -120 -130 -140 -150 -160 -170 -180 -190 -200 -210 -220 -230 -240
f1 (ppm)

(2*R**,3*R**)-3-(1,3-Dioxoisoindolin-2-yl)-2-(4-methoxyphenyl)azetidine-1-sulfonyl fluoride (4y’)

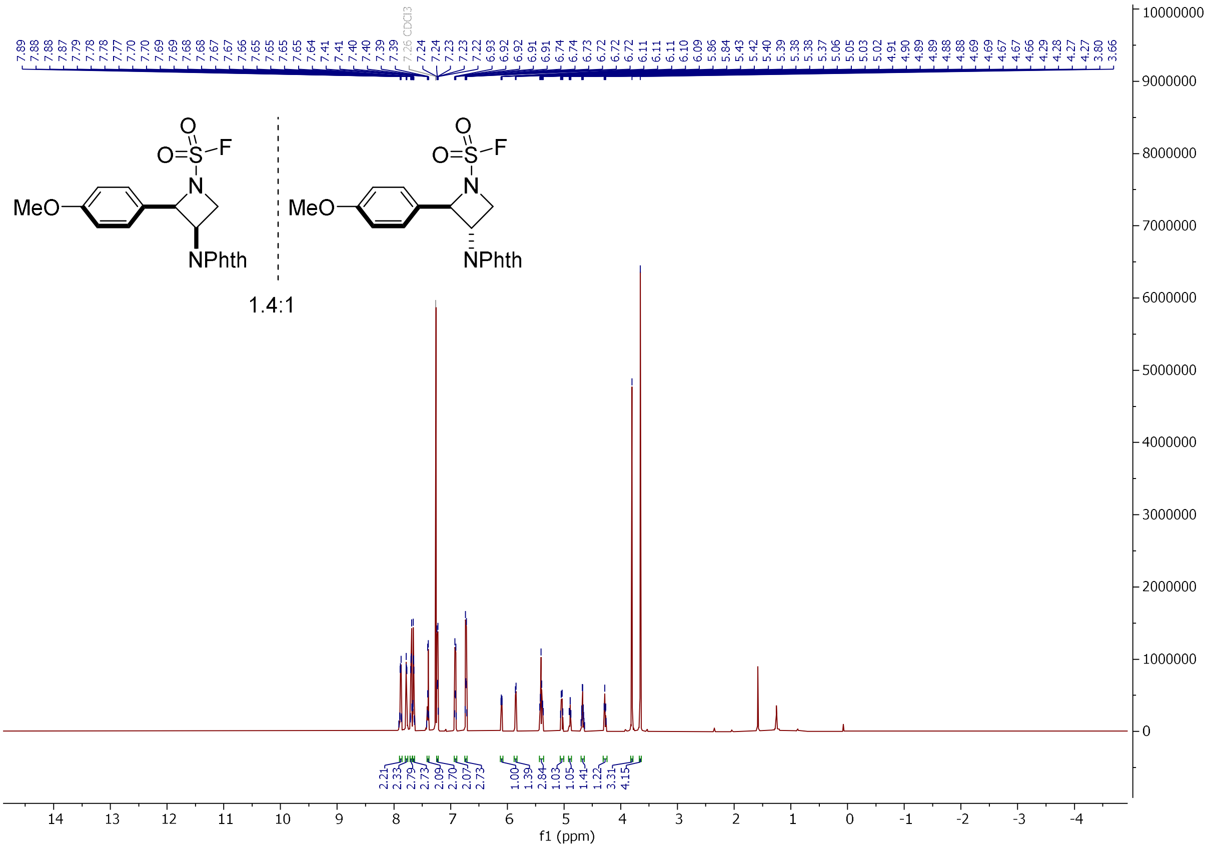

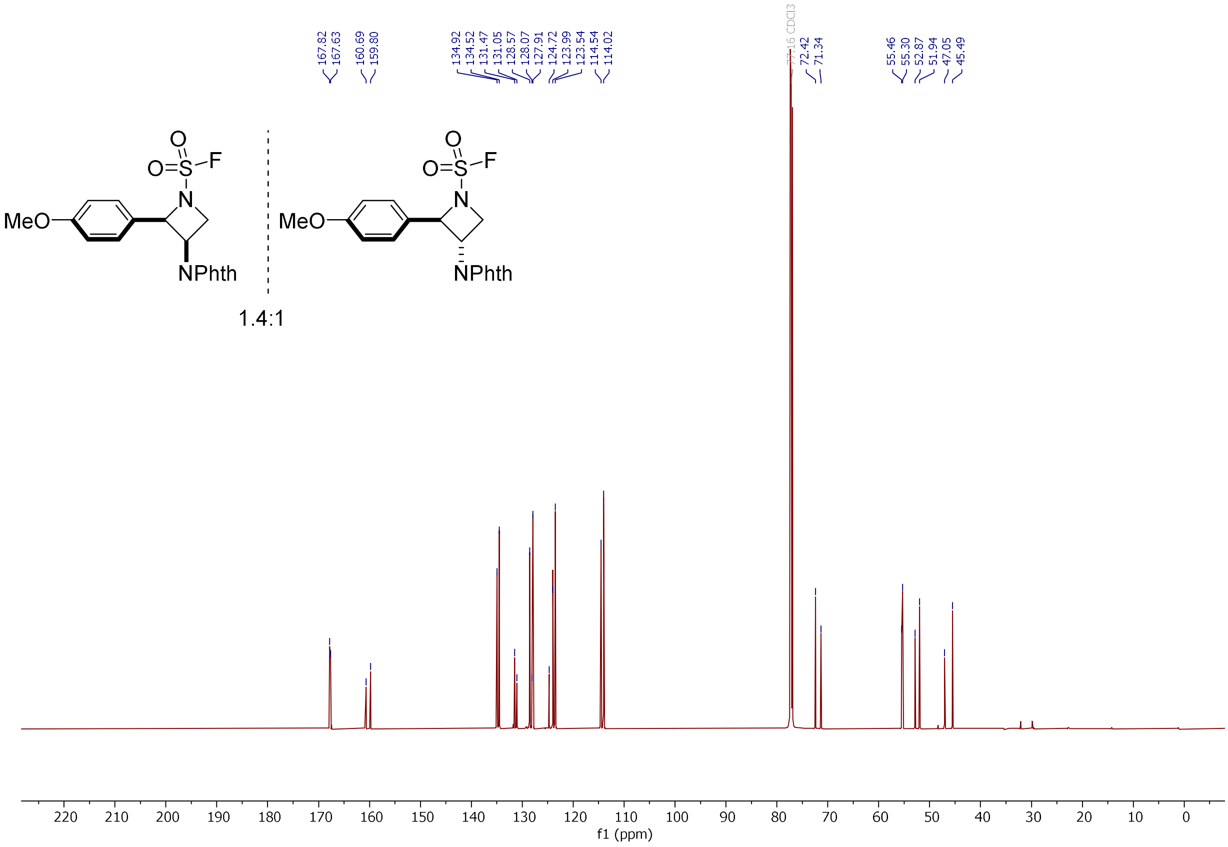

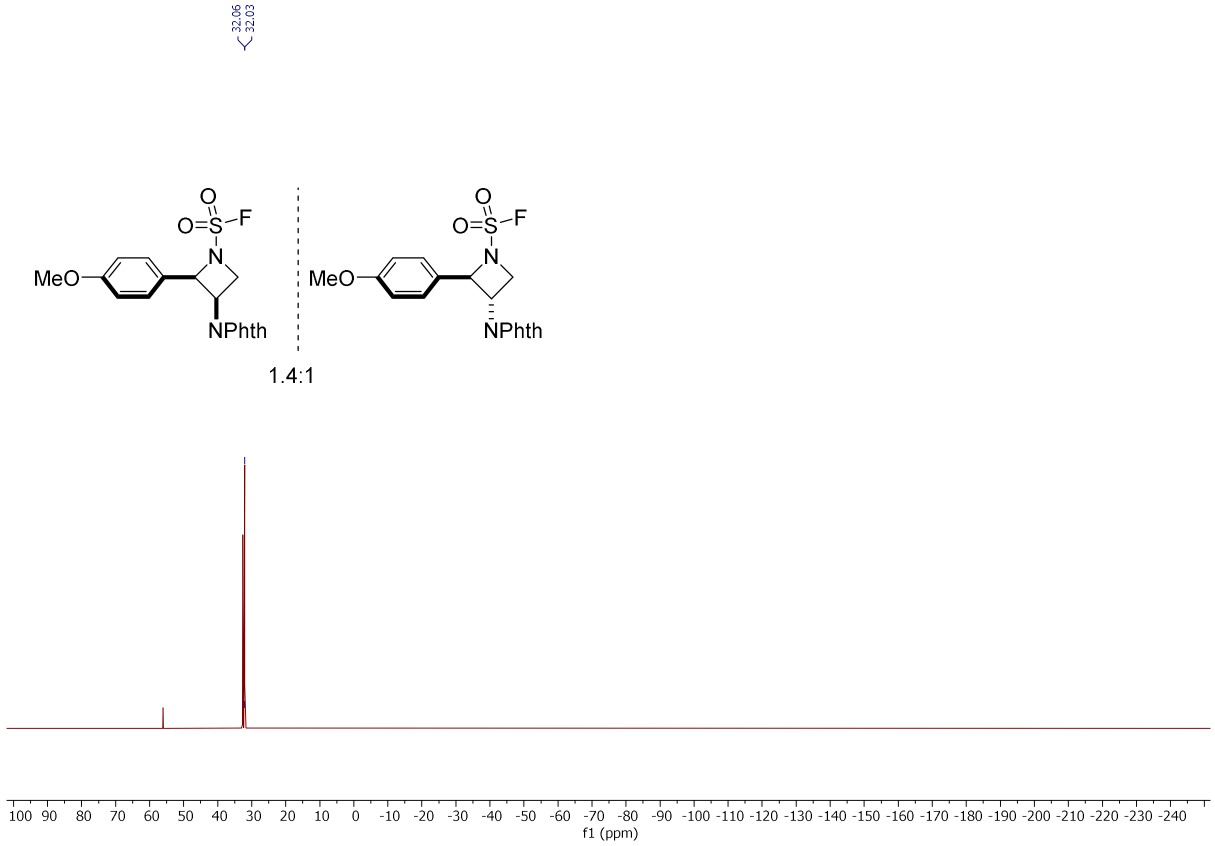
32.06
32.03
MeO
NPhth
1.4:1
f1 (ppm)

(2*R**,3*S**)-1-(Fluorosulfonyl)-2-(4-methoxyphenyl)-3-methylazetidin-3-yl acetate (4z)

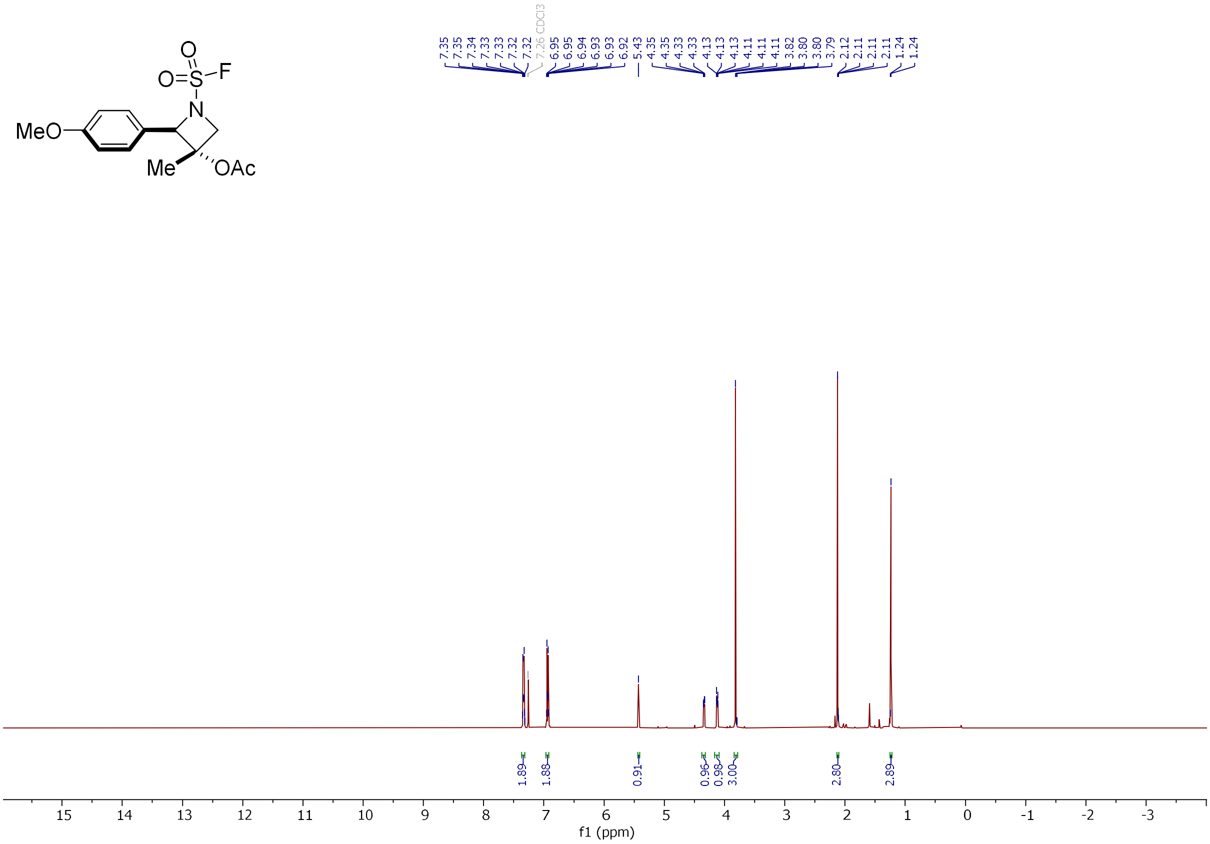

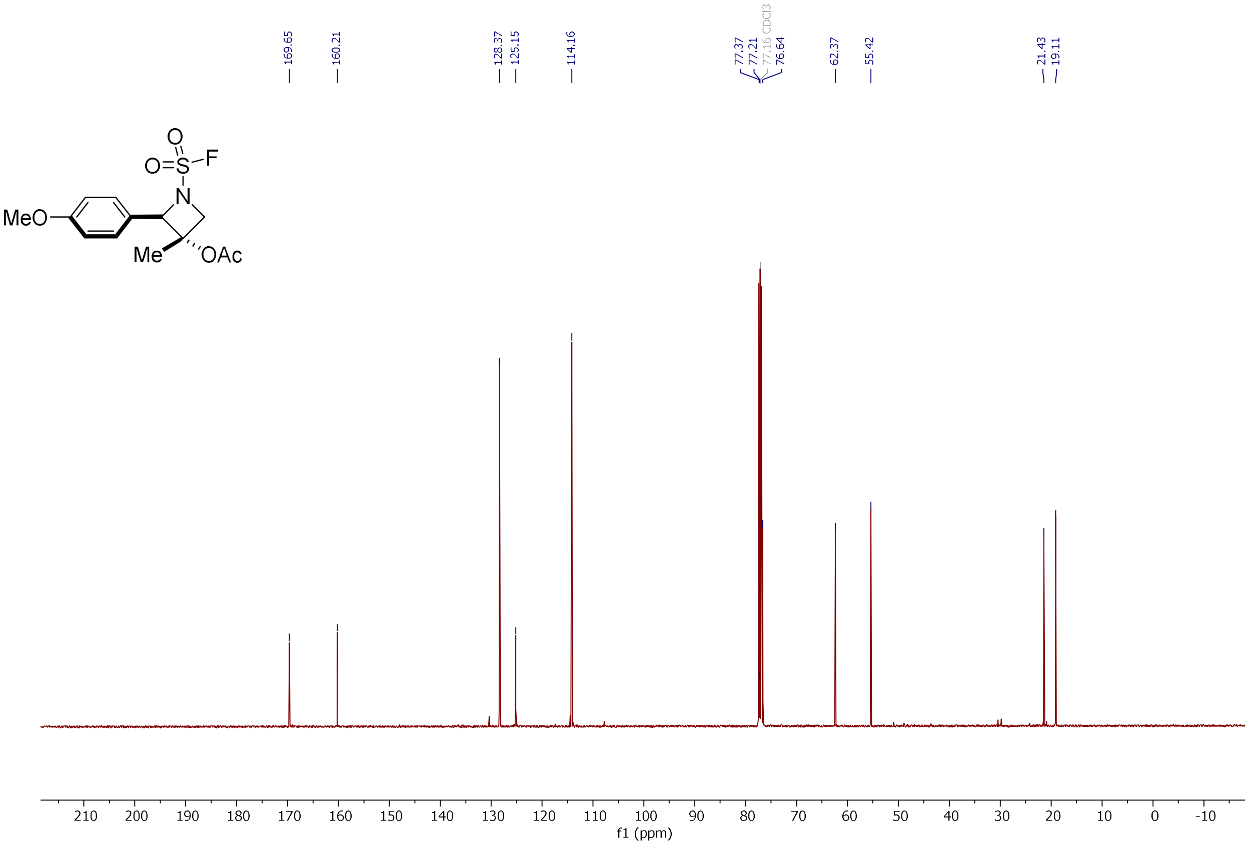

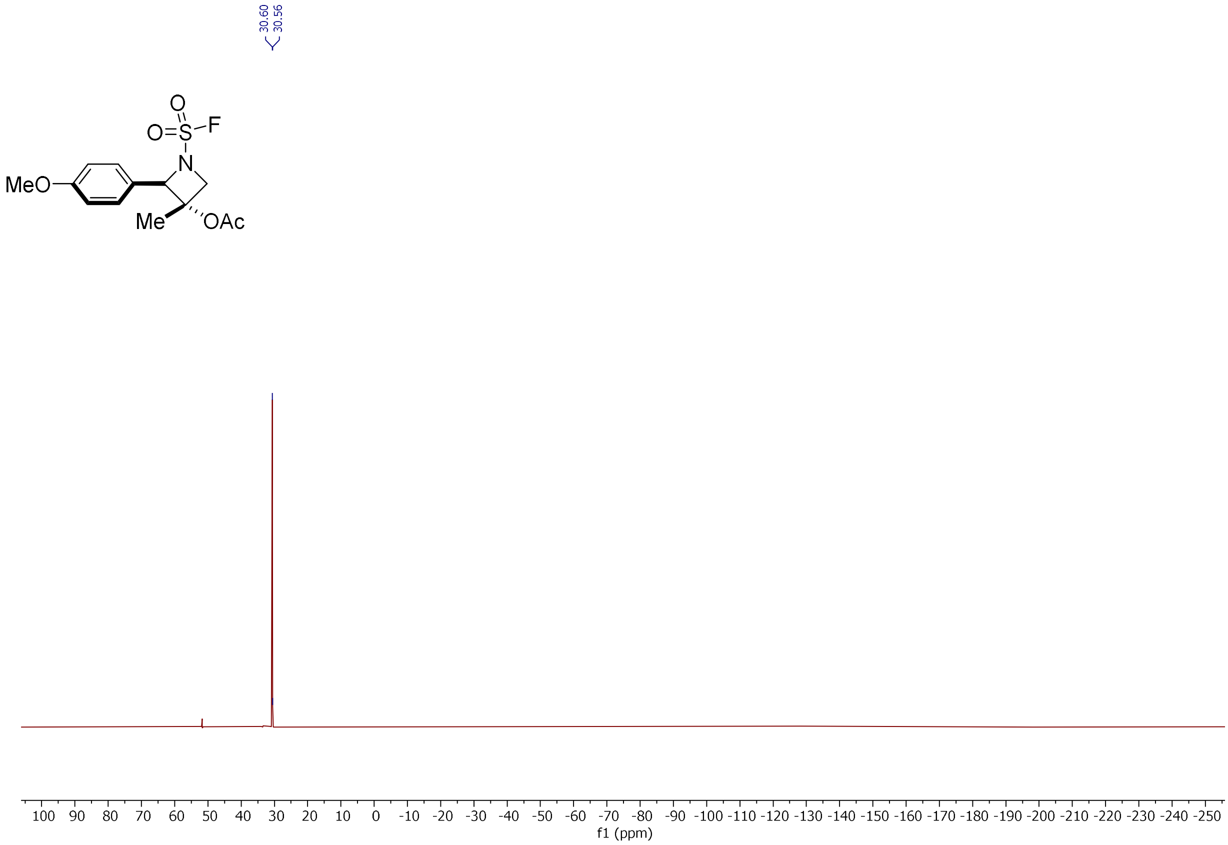

(2*R**,3*R**)-1-(Fluorosulfonyl)-2-(4-methoxyphenyl)-3-methylazetidin-3-yl acetate (4z′)

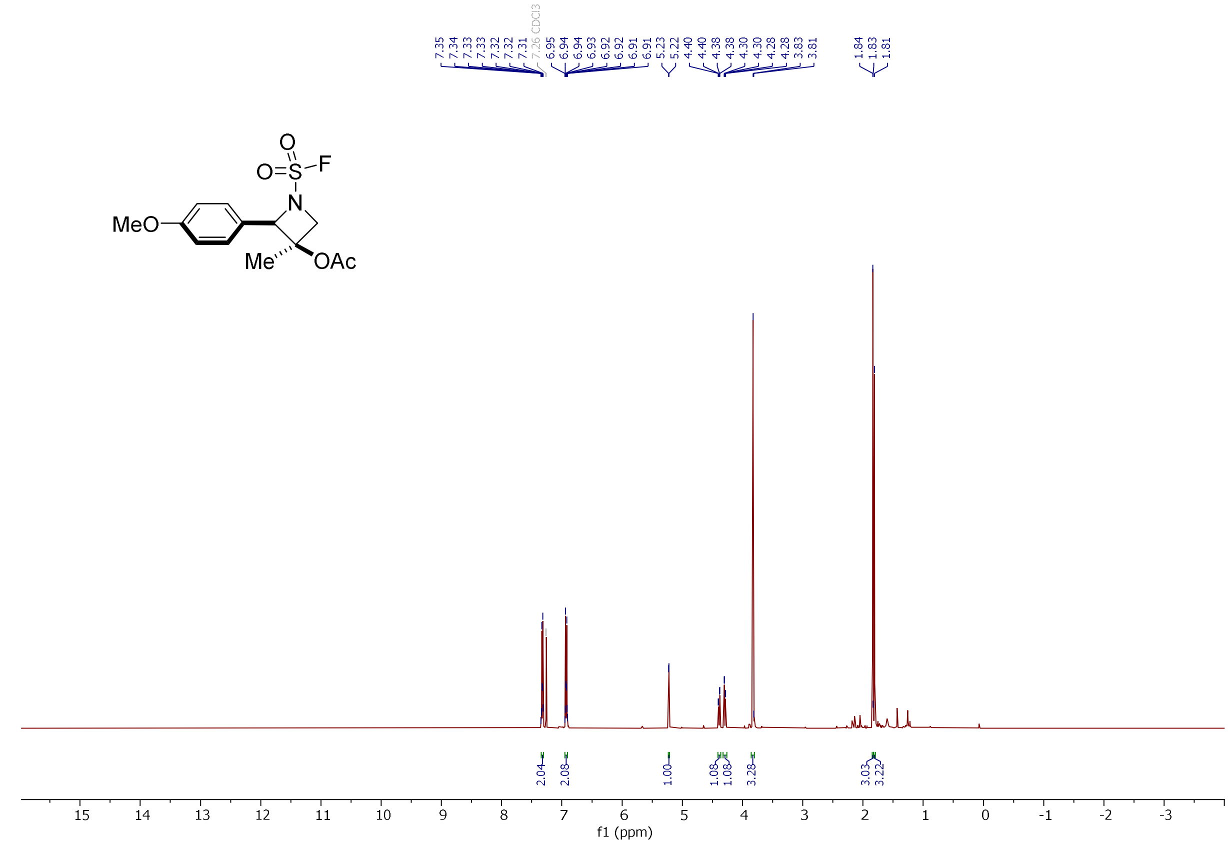

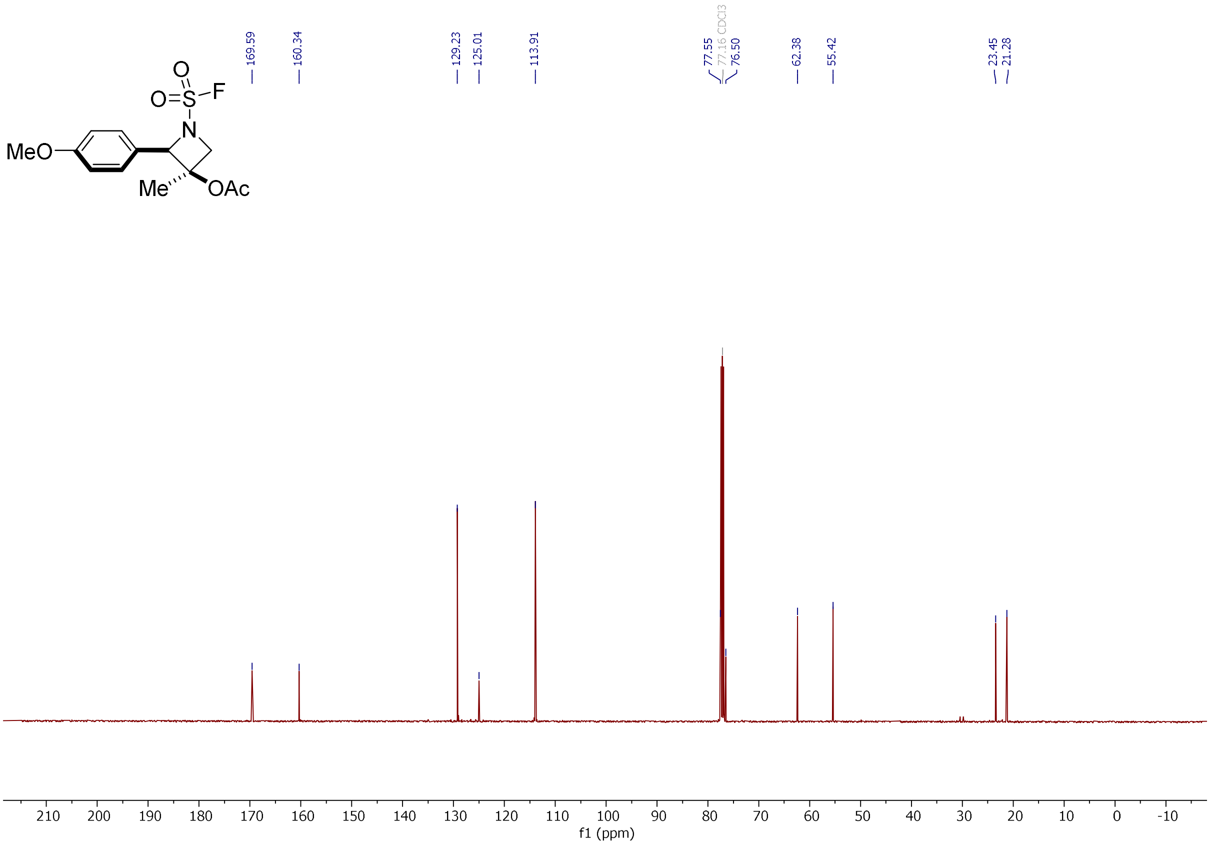
O
O=S-F
N
MeO
Me
OAc
169.59
160.34
129.23
125.01
113.91
77.55
77.16 CDCl3
76.50
62.38
55.42
23.45
21.28
210
200
190
180
170
160
150
140
130
120
110
100
90
80
70
60
50
40
30
20
10
0
-10
f1 (ppm)

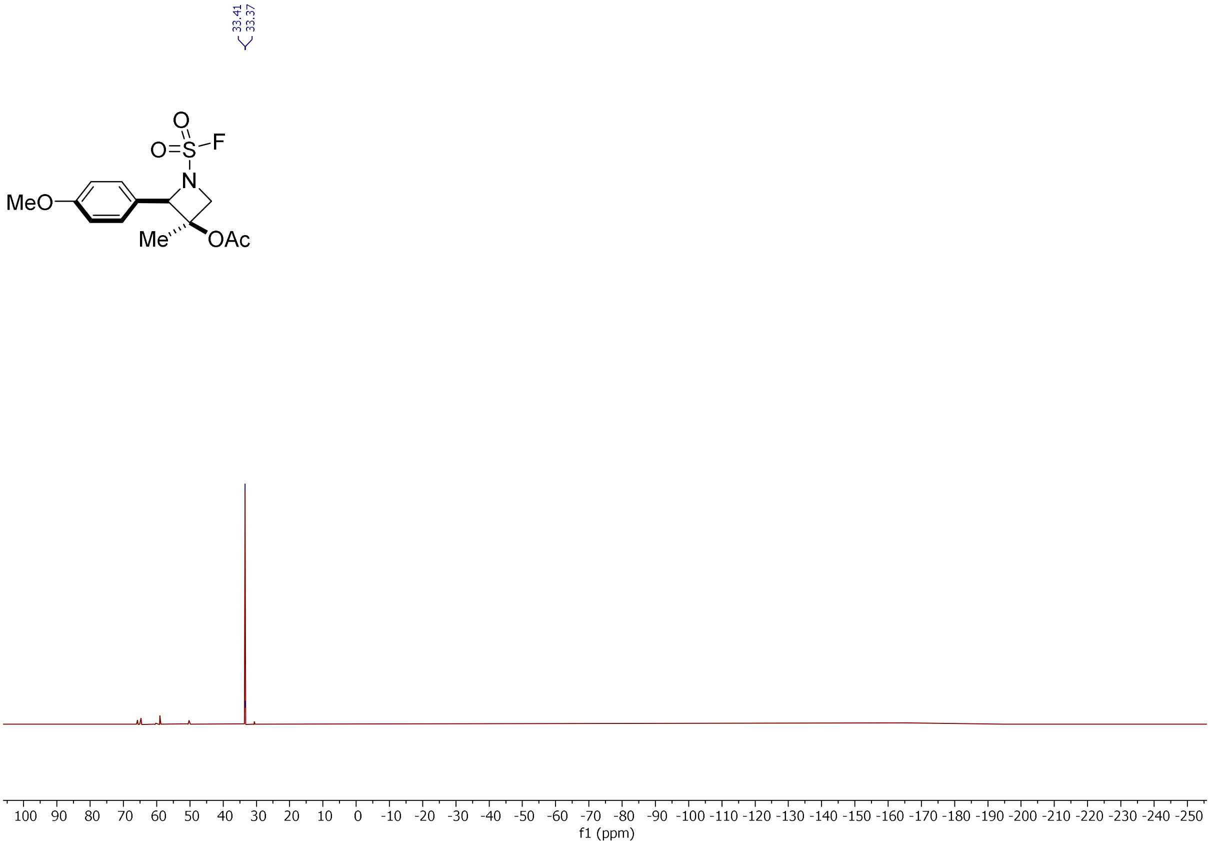
33.41
33.37
O
O=S-F
N
MeO
Me
OAc
100 90 80 70 60 50 40 30 20 10 0 -10 -20 -30 -40 -50 -60 -70 -80 -90 -100 -110 -120 -130 -140 -150 -160 -170 -180 -190 -200 -210 -220 -230 -240 -250
f1 (ppm)

(2*S**,2a*S**,7a*S**)-2-(4-Methoxyphenyl)-2a,7a-dihydrobenzofuro[2,3-*b*]azete-1(2*H*)-sulfonyl fluoride (4aa)

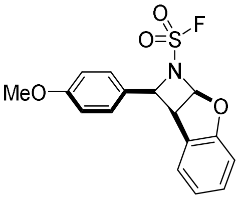

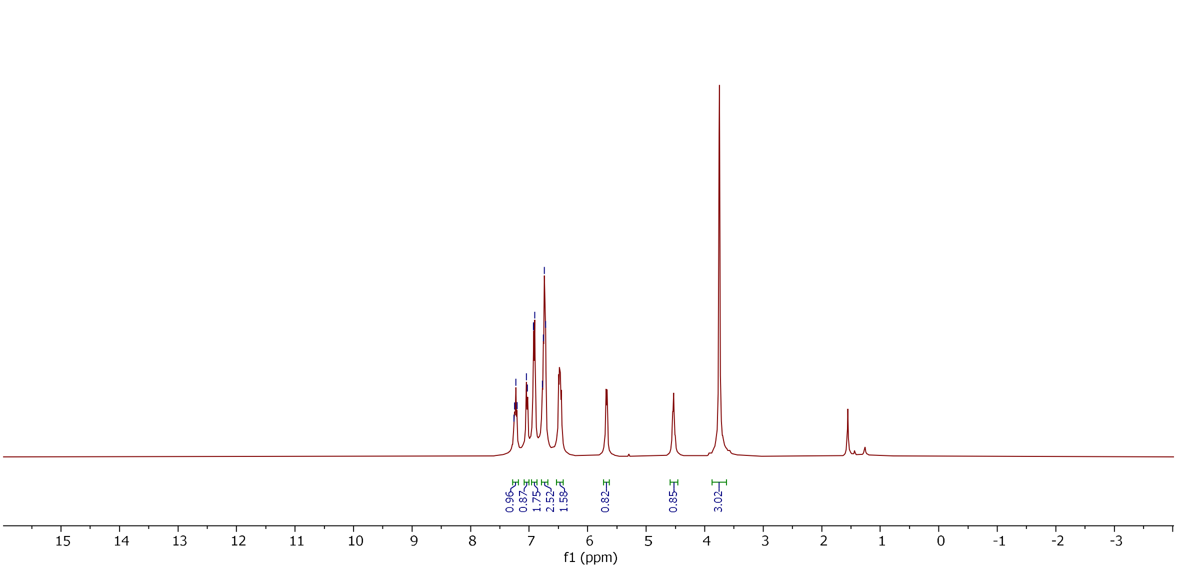

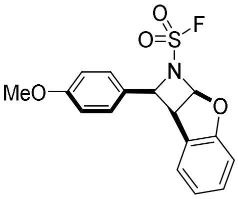

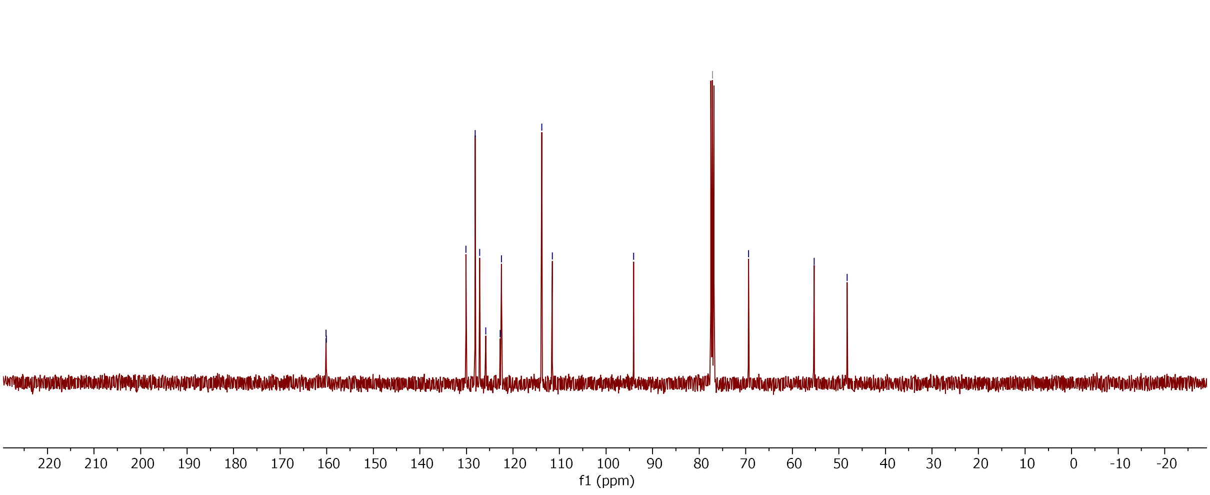

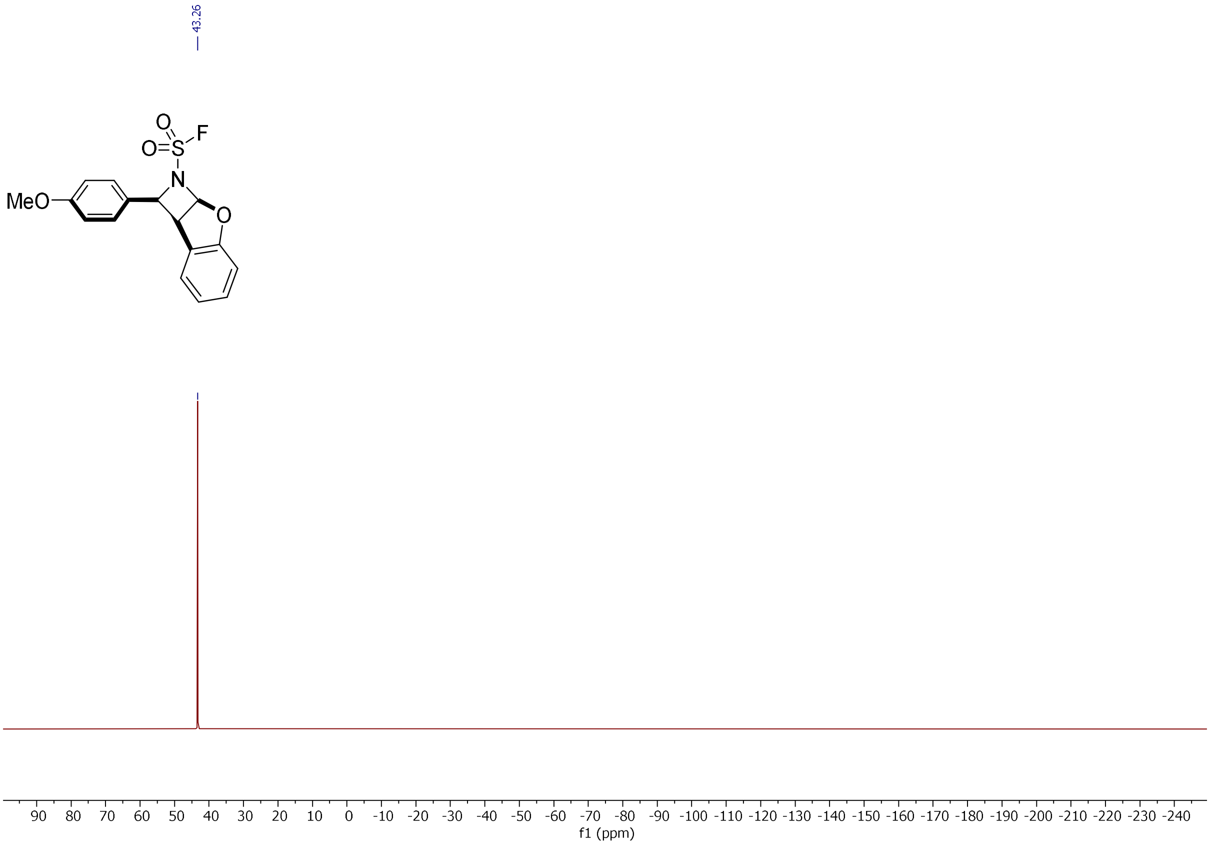

(1*R**,6*S**,8*R**)-8-(4-Methoxyphenyl)-2-oxa-7-azabicyclo[4.2.0]octane-7-sulfonyl fluoride (4ab)

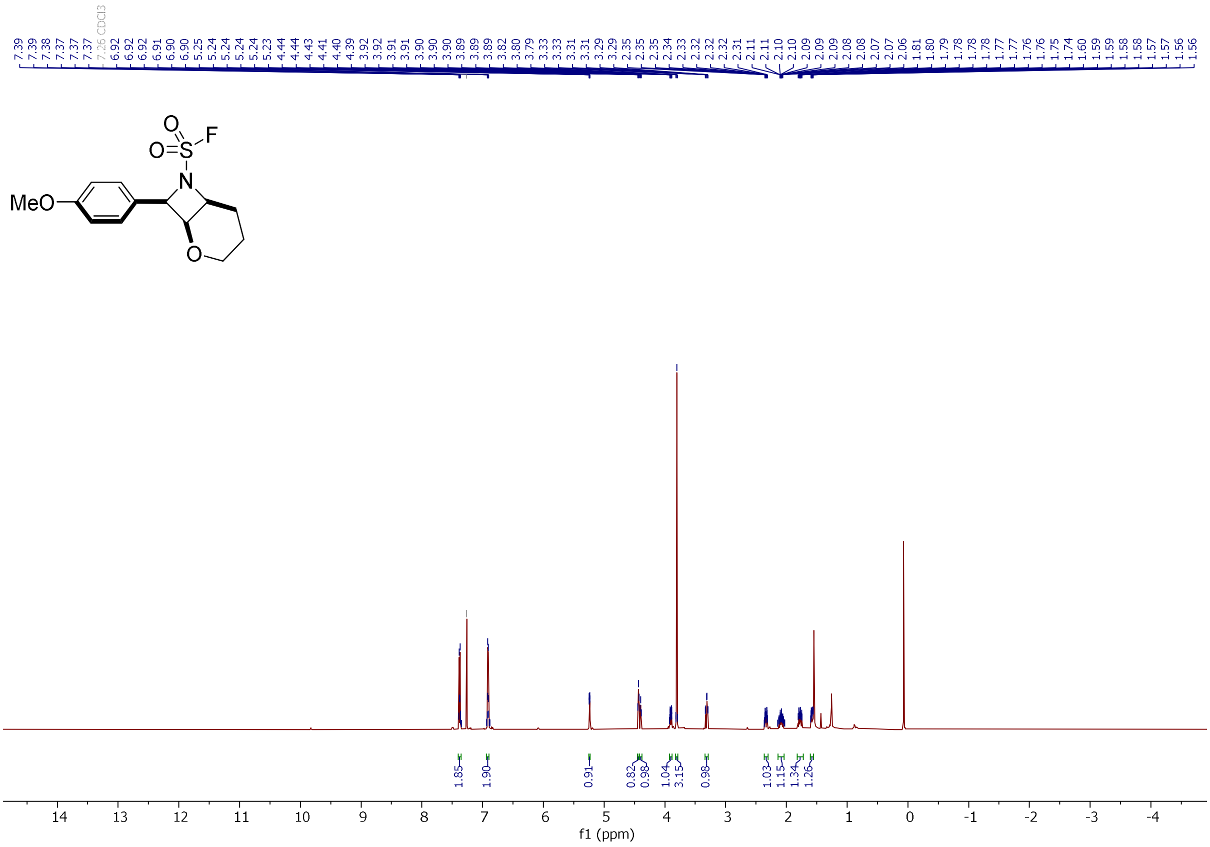

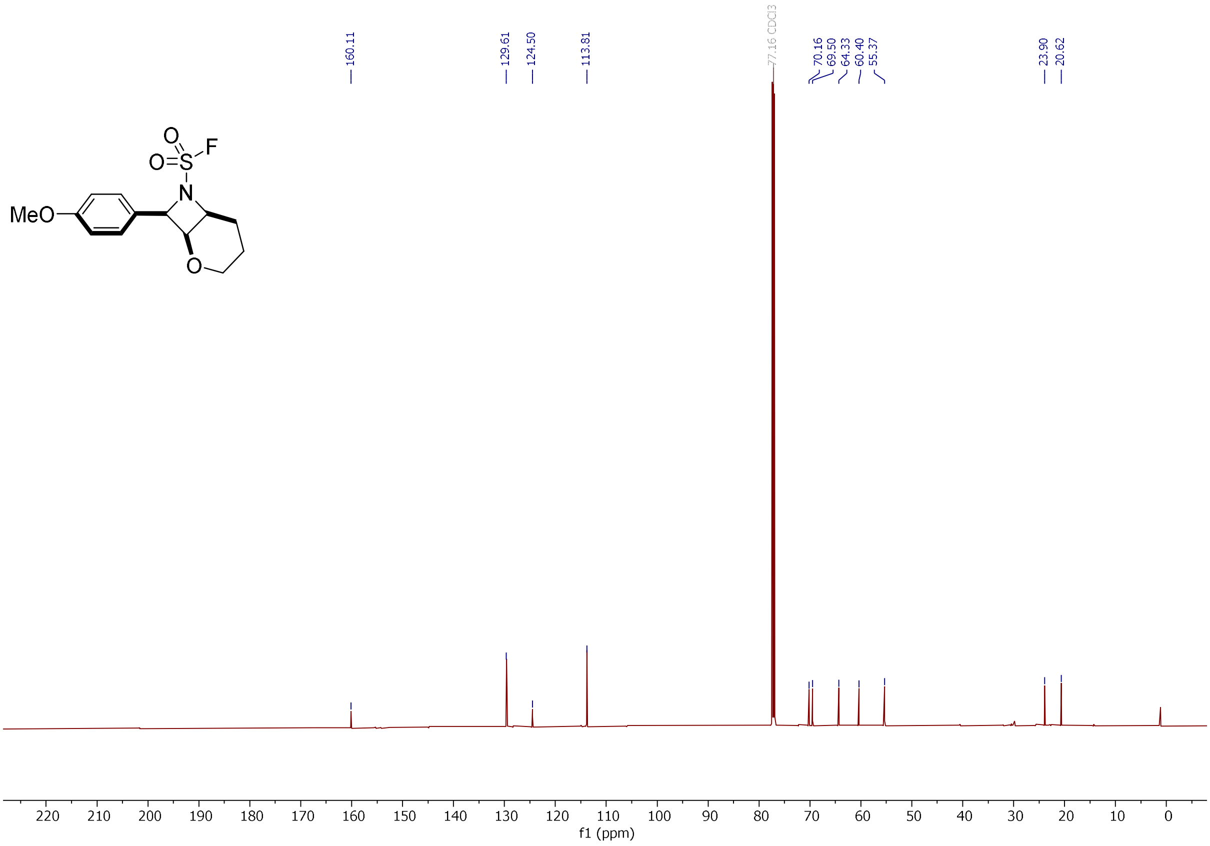
160.11
129.61
124.50
113.81
77.16 CDCl3
70.16
69.50
64.33
60.40
55.37
23.90
20.62
MeO
f1 (ppm)

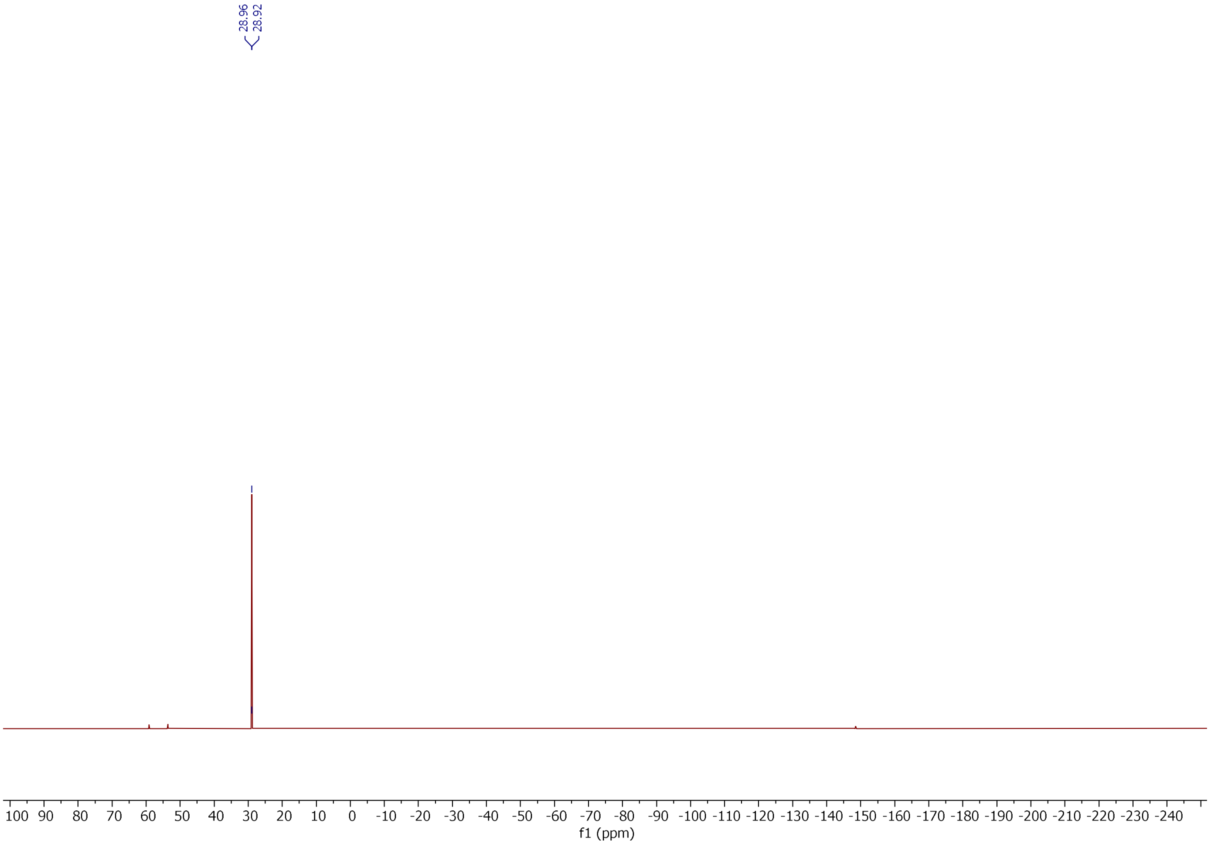
28.96
28.92
f1 (ppm)

(1*R**,6*S**,8*S**)-8-(4-Methoxyphenyl)-2-oxa-7-azabicyclo[4.2.0]octane-7-sulfonyl fluoride (4ab’)

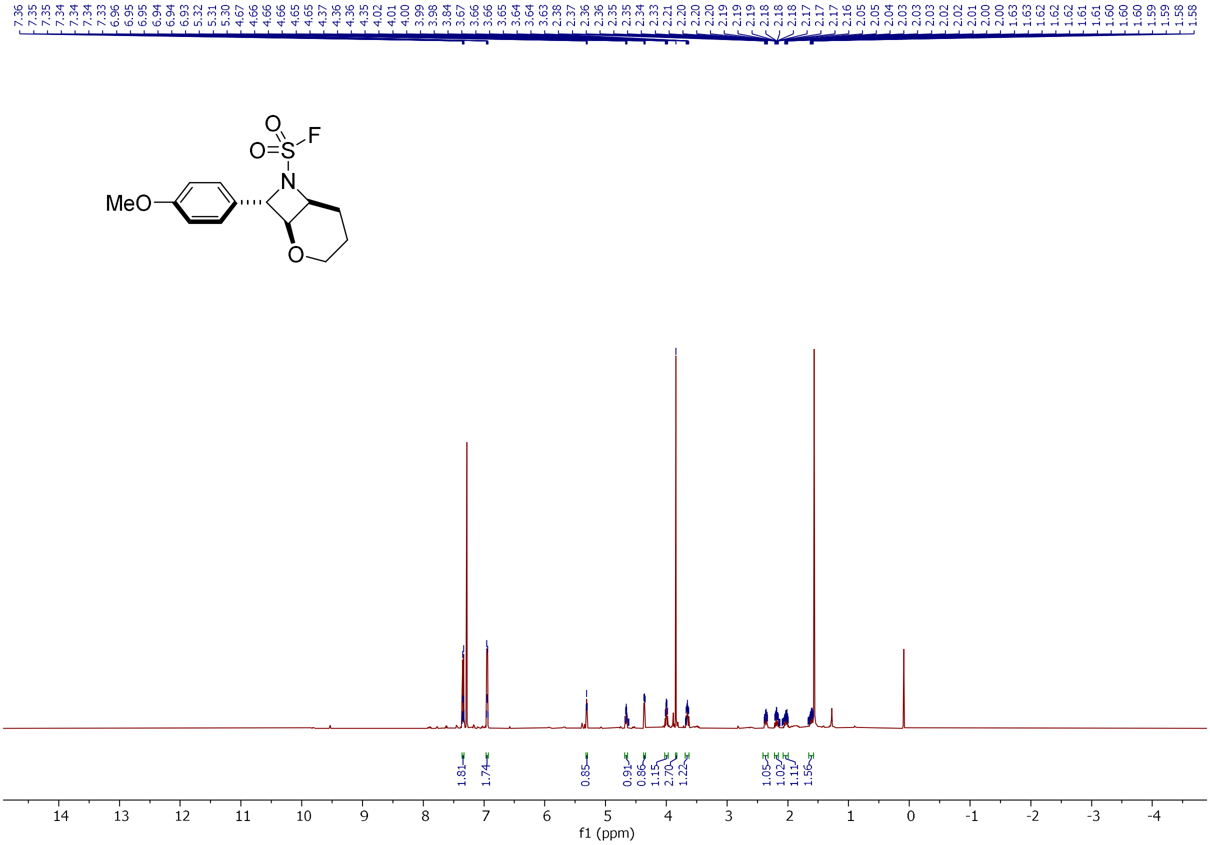

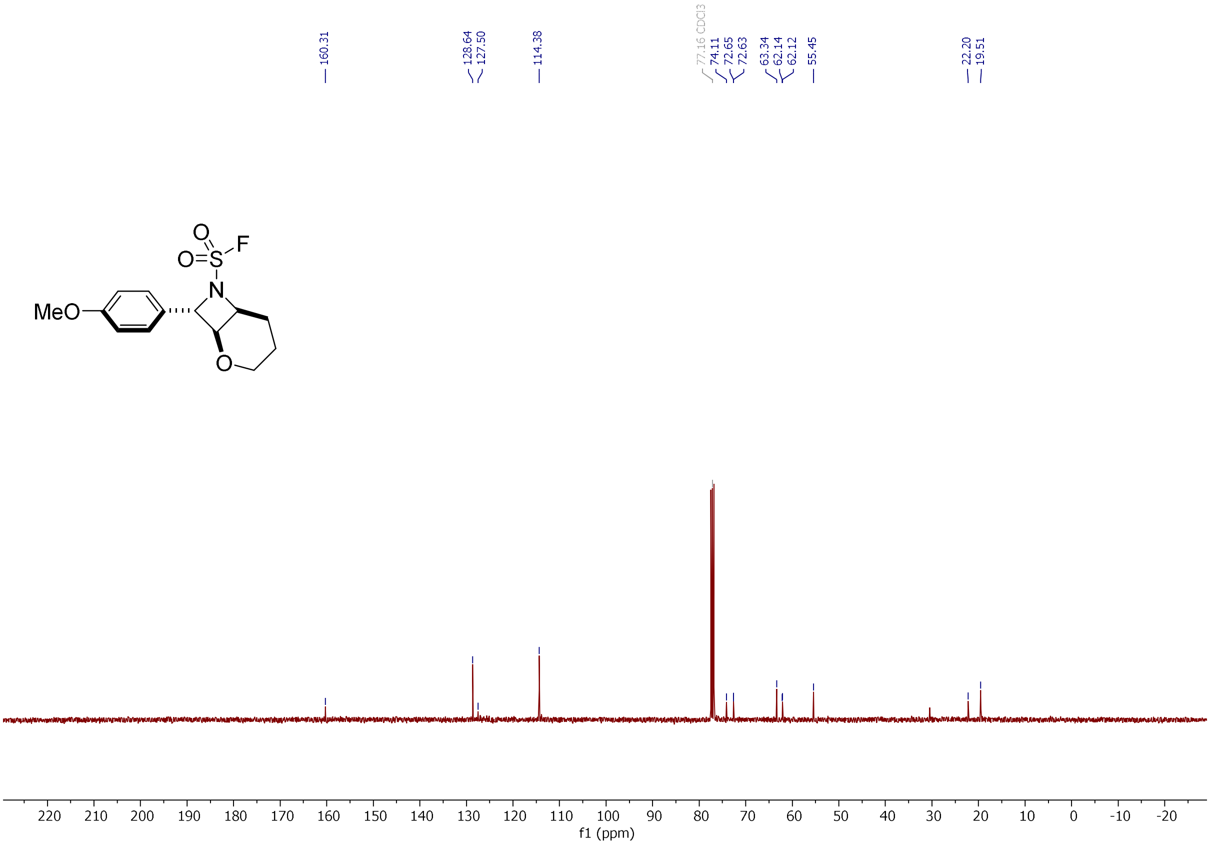

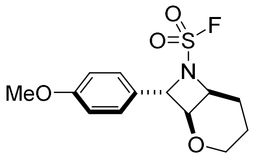
MeO
O
O=S
F
N
O

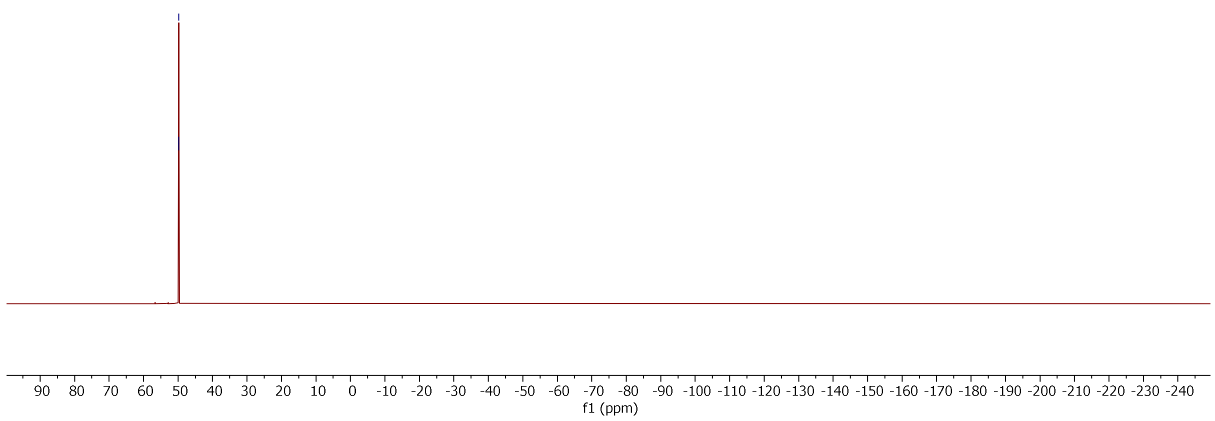
49.78
49.77
49.76
90 80 70 60 50 40 30 20 10 0 -10 -20 -30 -40 -50 -60 -70 -80 -90 -100 -110 -120 -130 -140 -150 -160 -170 -180 -190 -200 -210 -220 -230 -240
f1 (ppm)

3,3-diethyl-2-(5-hydroxy-6-methoxypyridin-3-yl)azetidine-1-sulfonyl fluoride (3t)

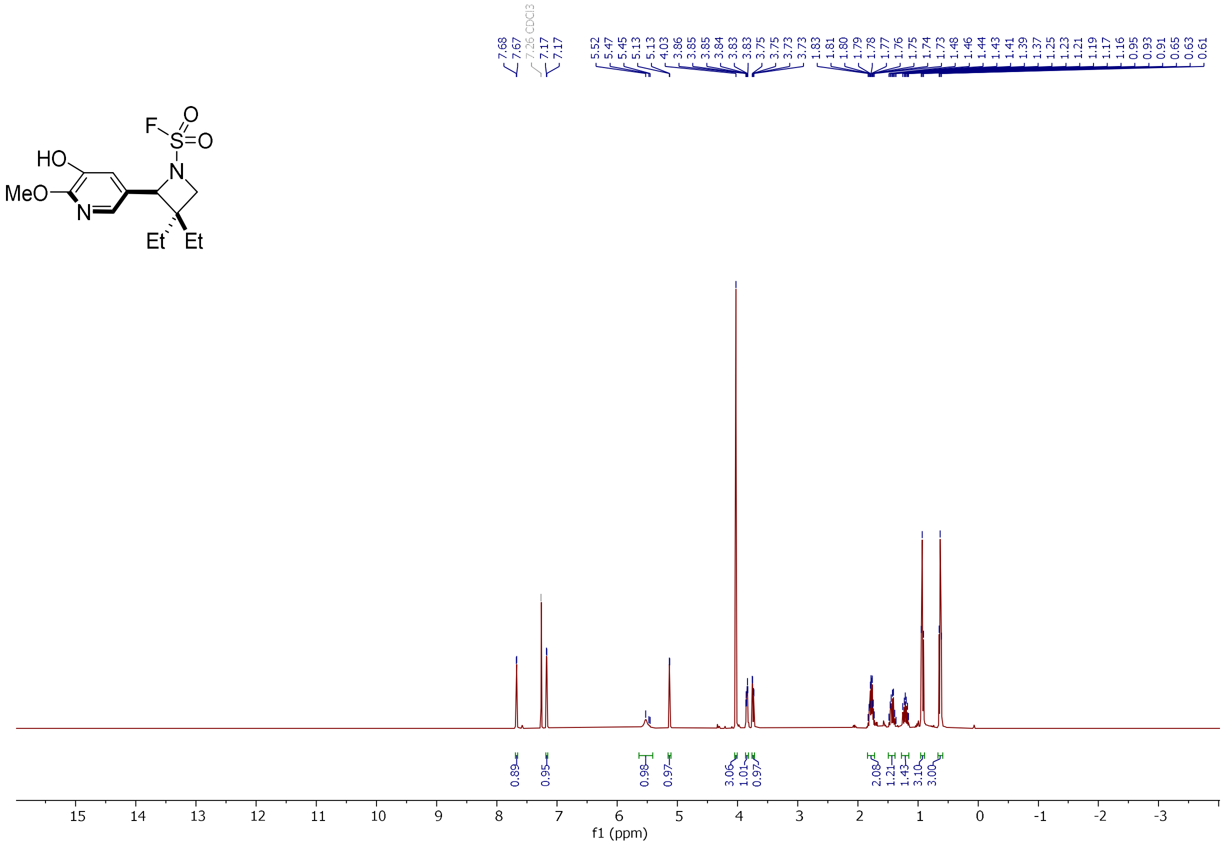

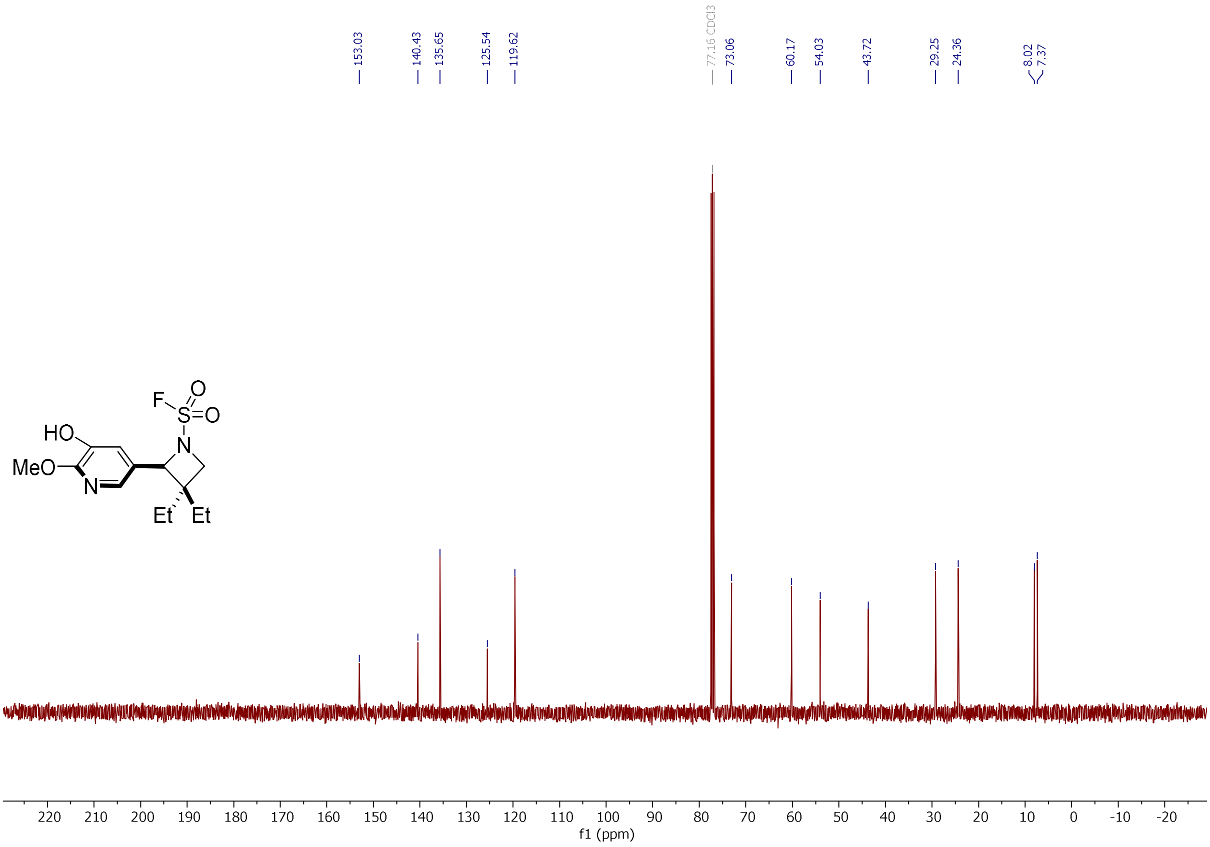

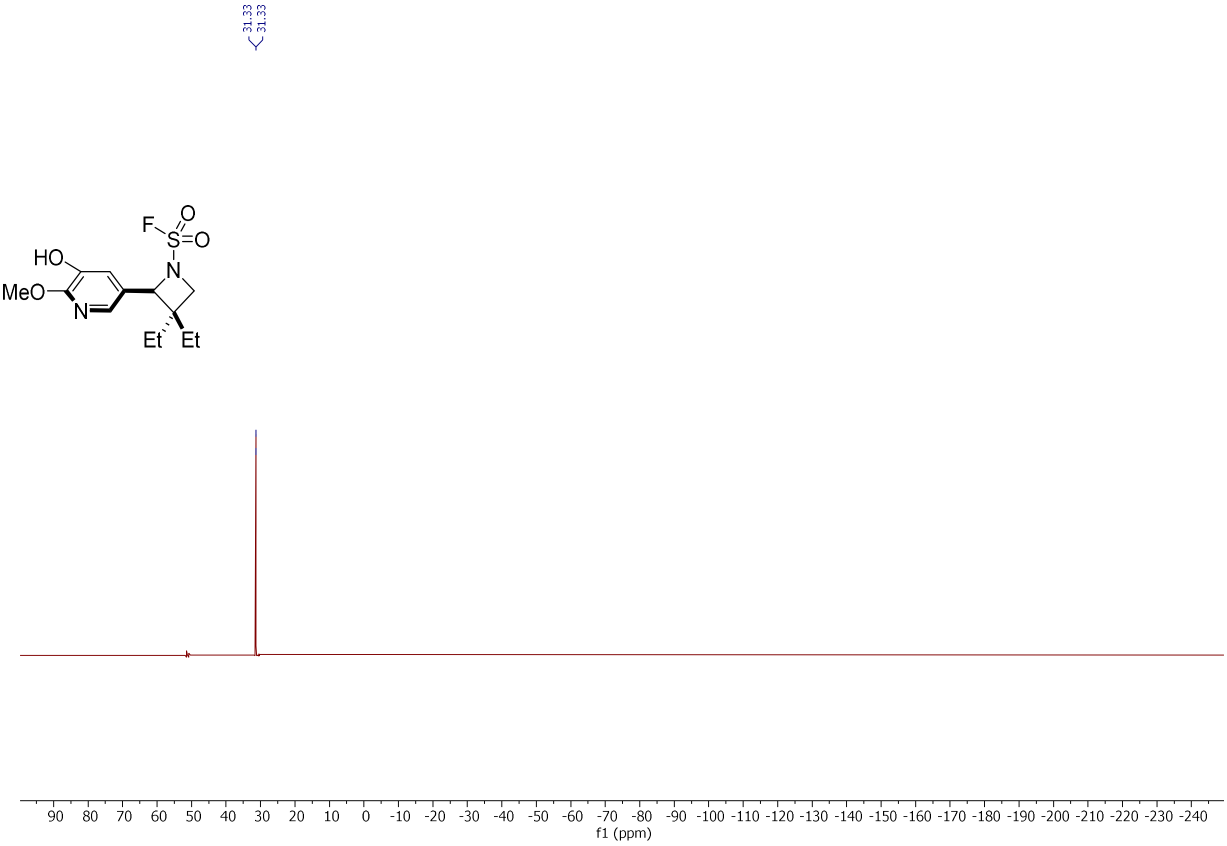

2-(2,4-Dimethoxypyrimidin-5-yl)-3,3-diethylazetidine-1-sulfonyl fluoride (3u)

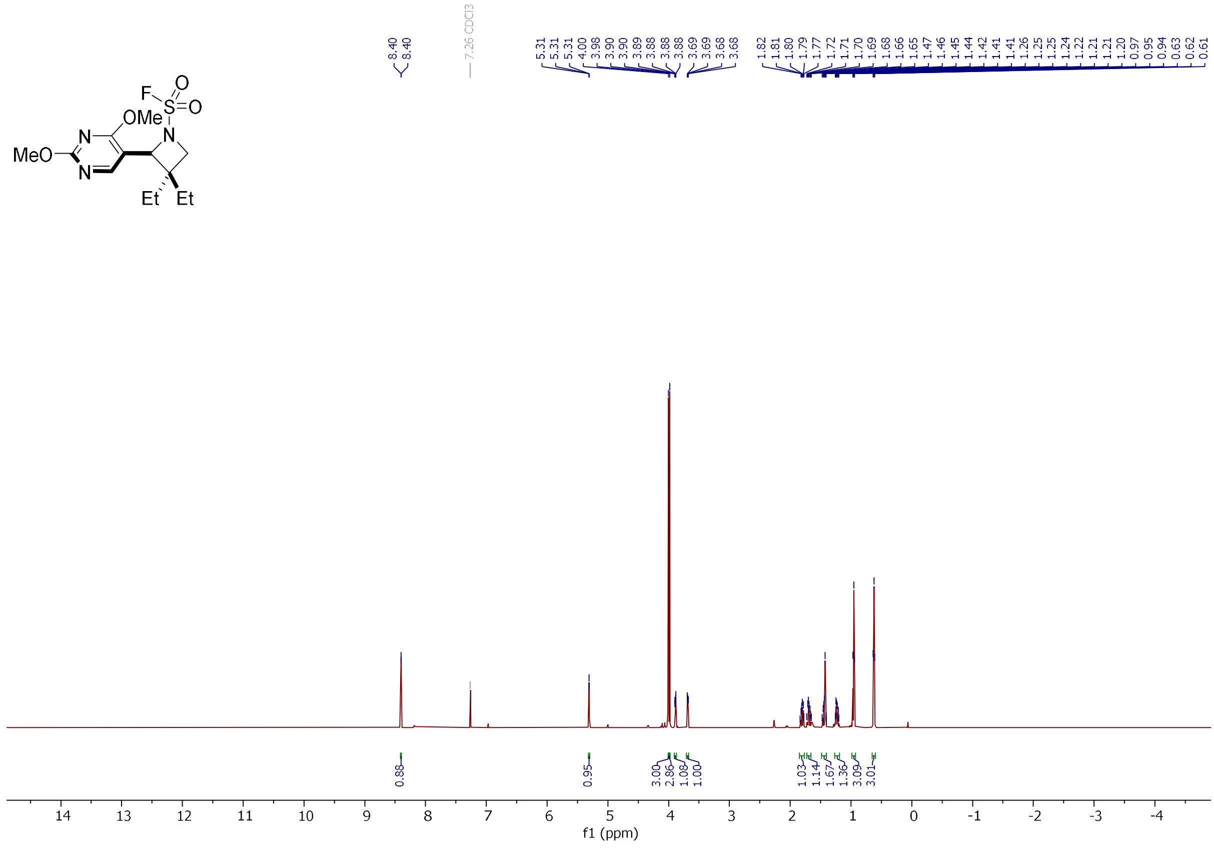

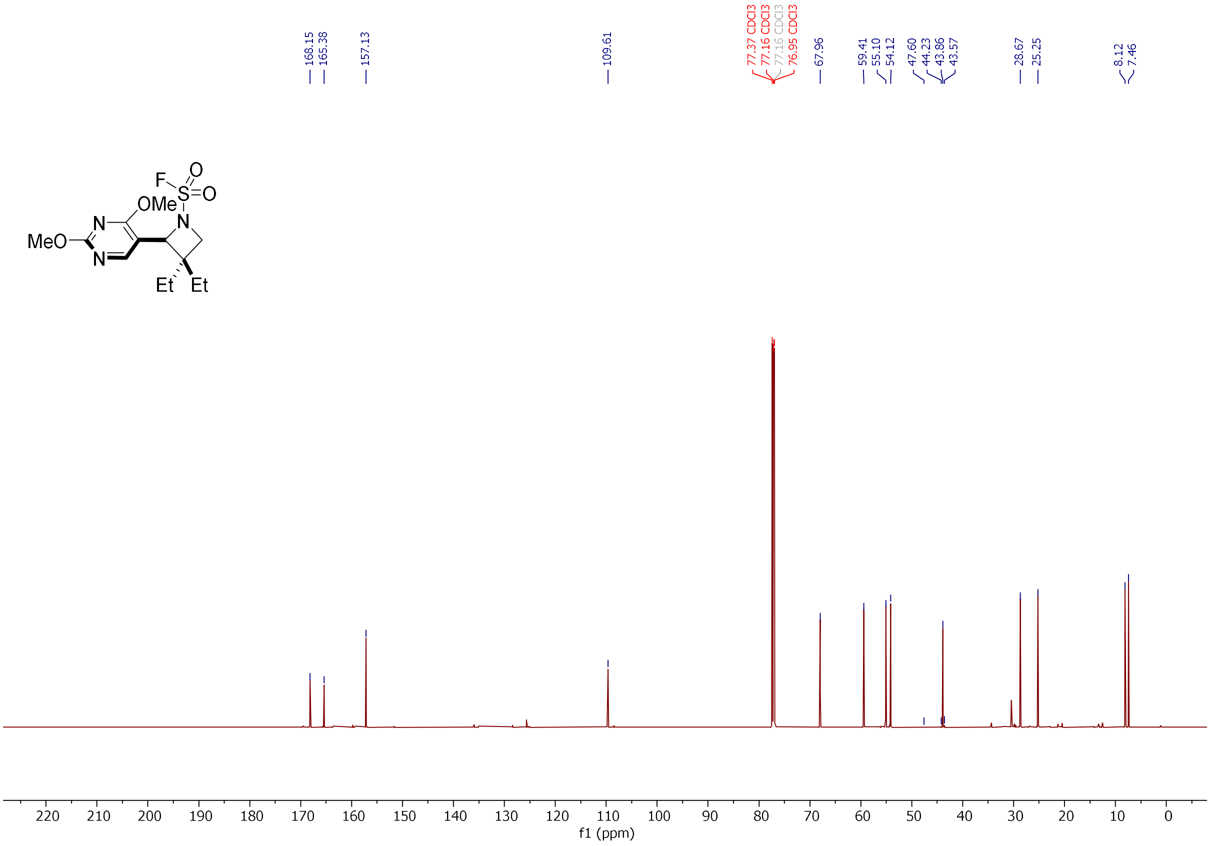
168.15
165.38
157.13
109.61
77.37 CDCl3
77.16 CDCl3
77.16 CDCl3
76.95 CDCl3
67.96
59.41
55.10
54.12
47.60
44.23
43.86
43.57
28.67
25.25
8.12
7.46
F
O
S
O
OMe
N
MeO
N
N
Et
Et
220 210 200 190 180 170 160 150 140 130 120 110 100 90 80 70 60 50 40 30 20 10 0
f1 (ppm)

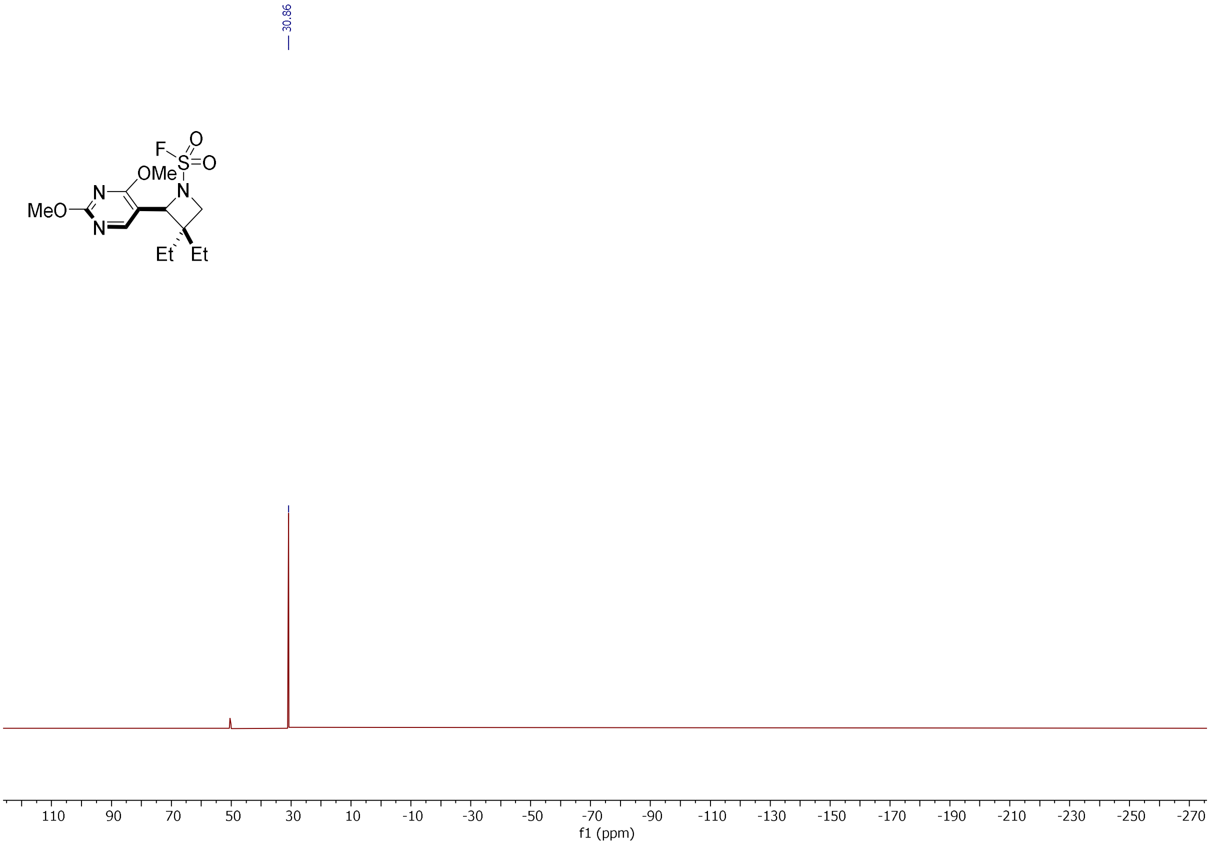
30.86
F
O
S
O
OMe
N
MeO
N
N
Et
Et
110 90 70 50 30 10 -10 -30 -50 -70 -90 -110 -130 -150 -170 -190 -210 -230 -250 -270
f1 (ppm)

3,3-Diethyl-2-(2-methoxy-1-methyl-4-oxo-1,4-dihydropyrimidin-5-yl)azetidine-1-sulfonyl fluoride (3v)

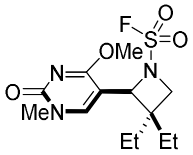

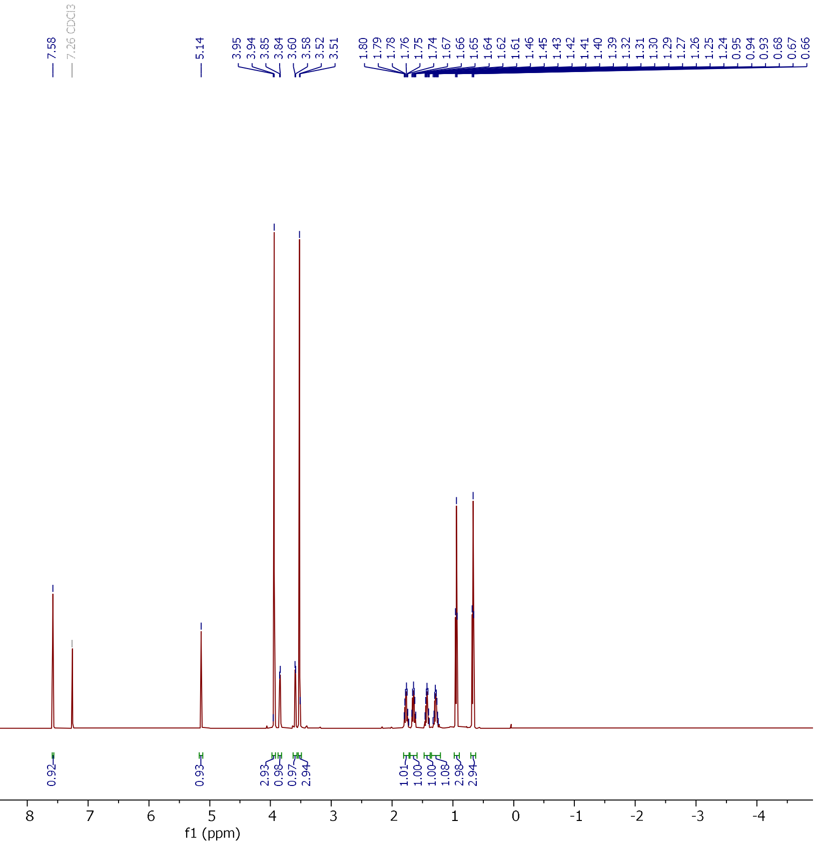

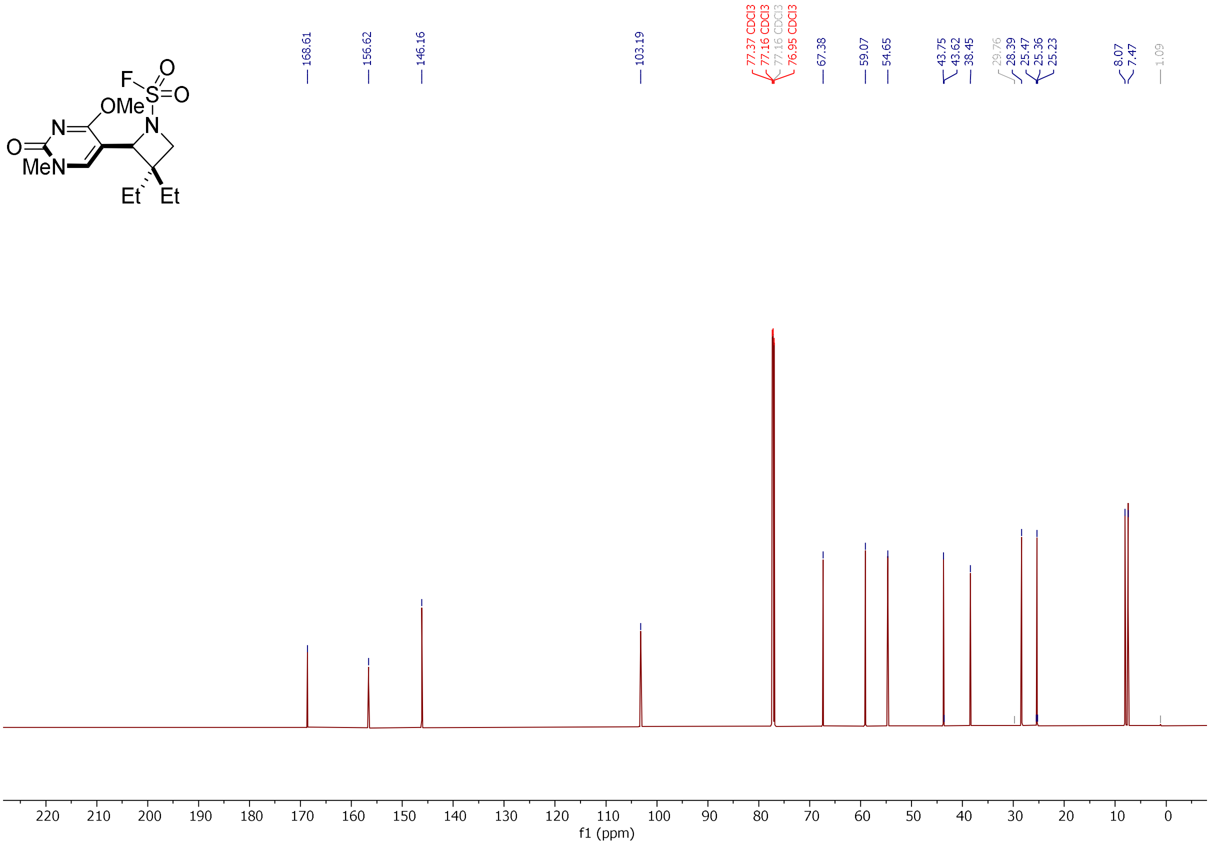
168.61
156.62
146.16
103.19
77.37 CDCl3
77.16 CDCl3
77.16 CDCl3
76.95 CDCl3
67.38
59.07
54.65
43.75
43.62
38.45
29.76
28.39
25.47
25.36
25.23
8.07
7.47
1.09
F
O
S
O
OMe
N
N
O
MeN
Et
Et
f1 (ppm)

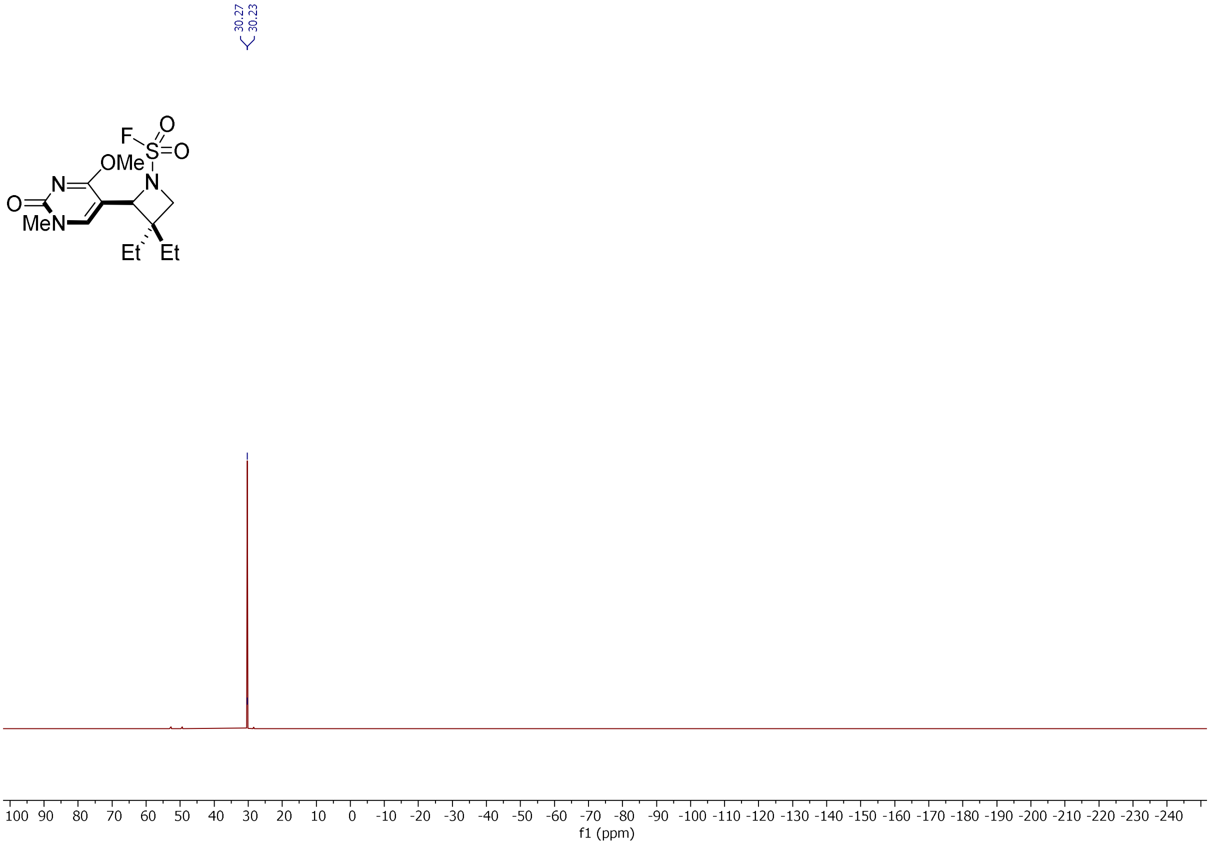
30.27
30.23
F
O
S
O
OMe
N
N
O
MeN
Et
Et
f1 (ppm)

2-(benzo[d]oxazol-5-yl)-3,3-diethylazetidine-1-sulfonyl fluoride (3x)

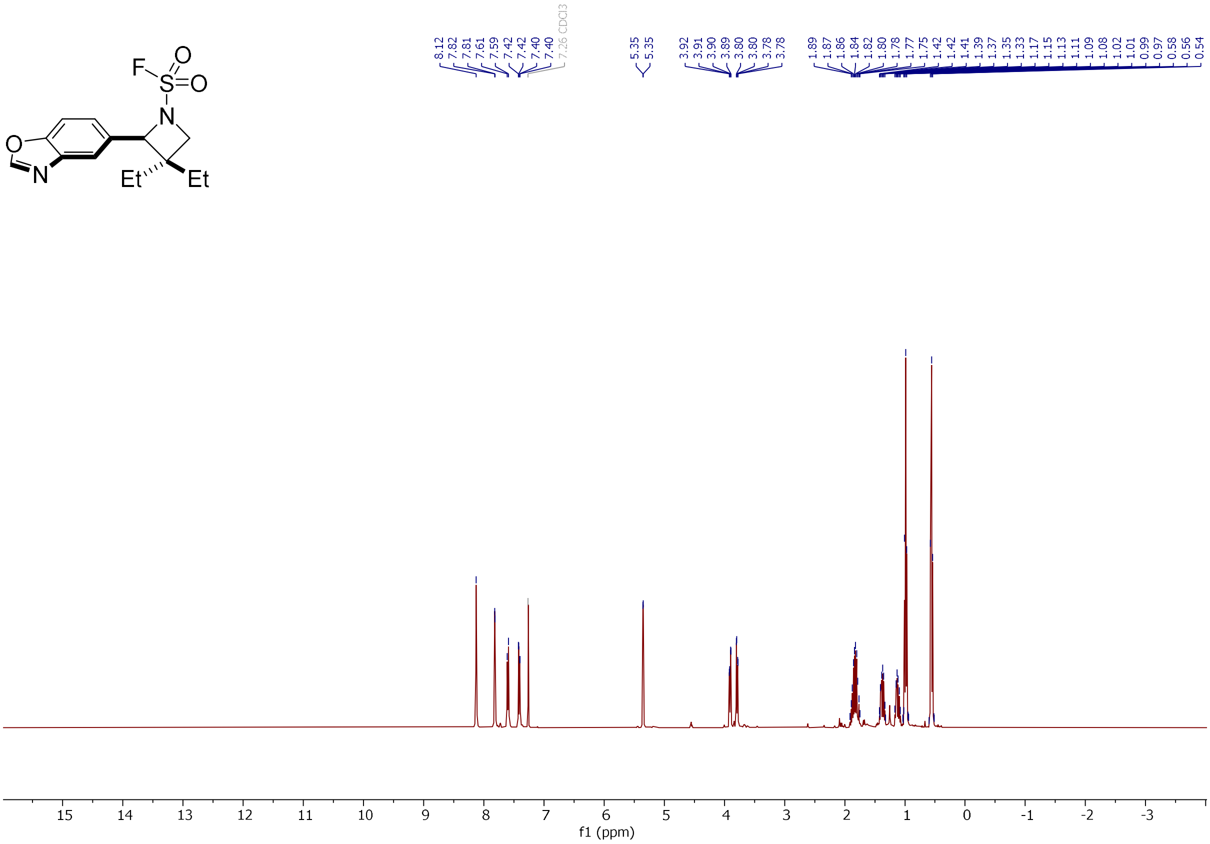

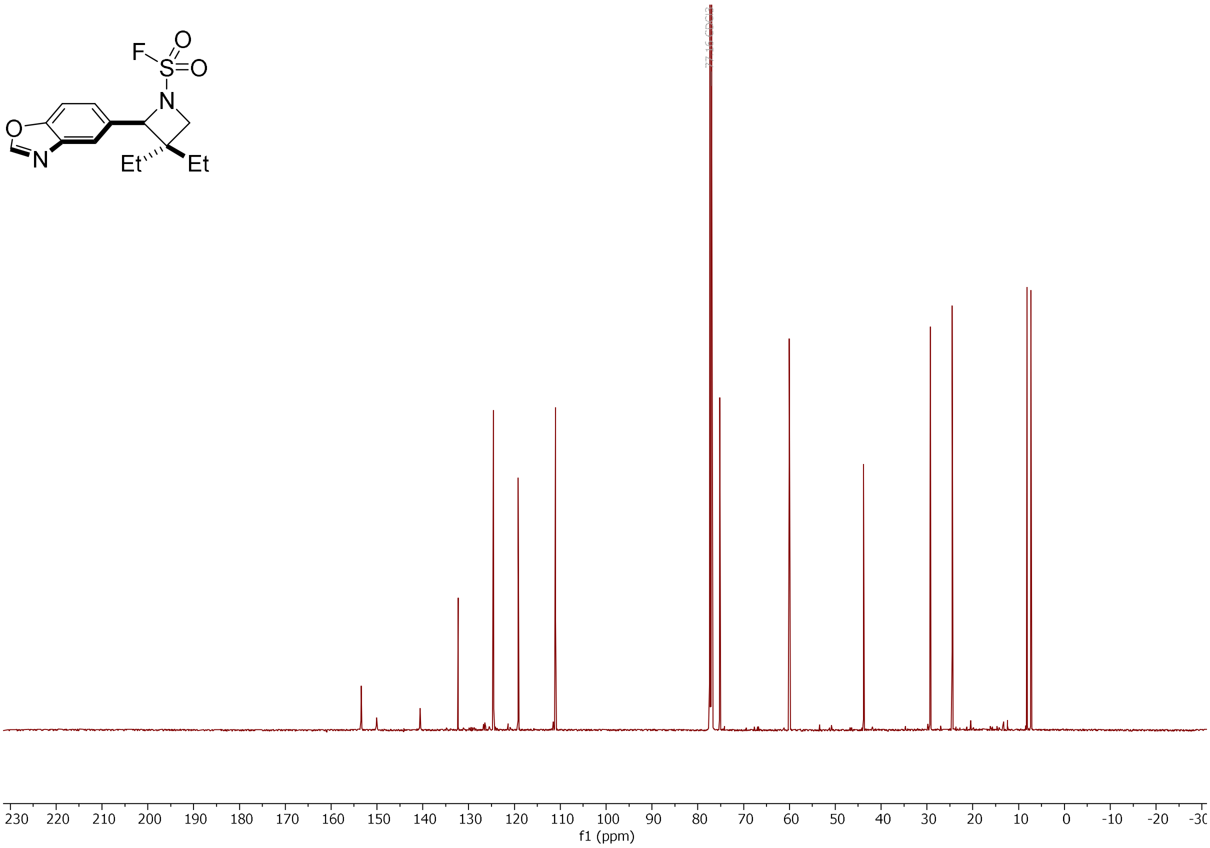

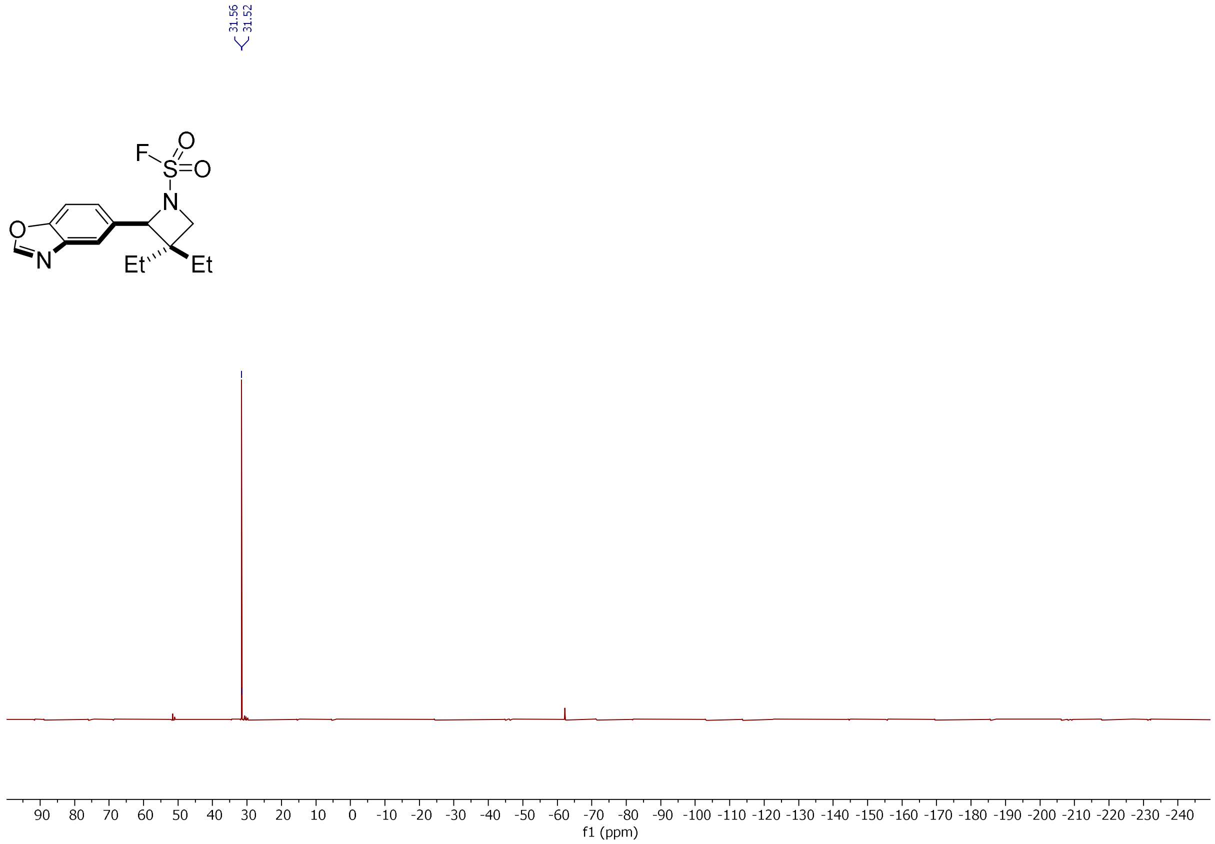
31.56
31.52
F
S
O
O
N
O
N
Et
Et
90 80 70 60 50 40 30 20 10 0 -10 -20 -30 -40 -50 -60 -70 -80 -90 -100 -110 -120 -130 -140 -150 -160 -170 -180 -190 -200 -210 -220 -230 -240
f1 (ppm)

Derivitisation

3,3-Diethyl-2-(4-methoxyphenyl) azetidine (5)

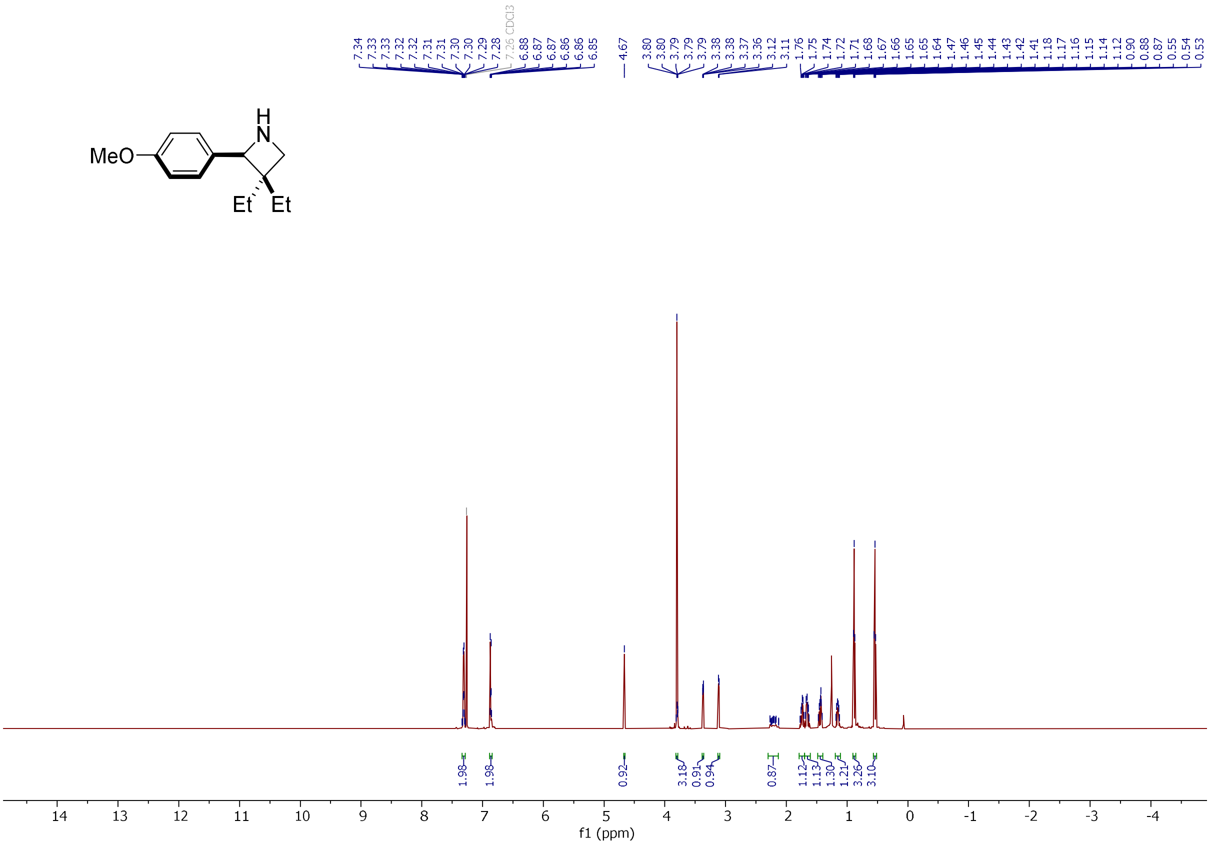

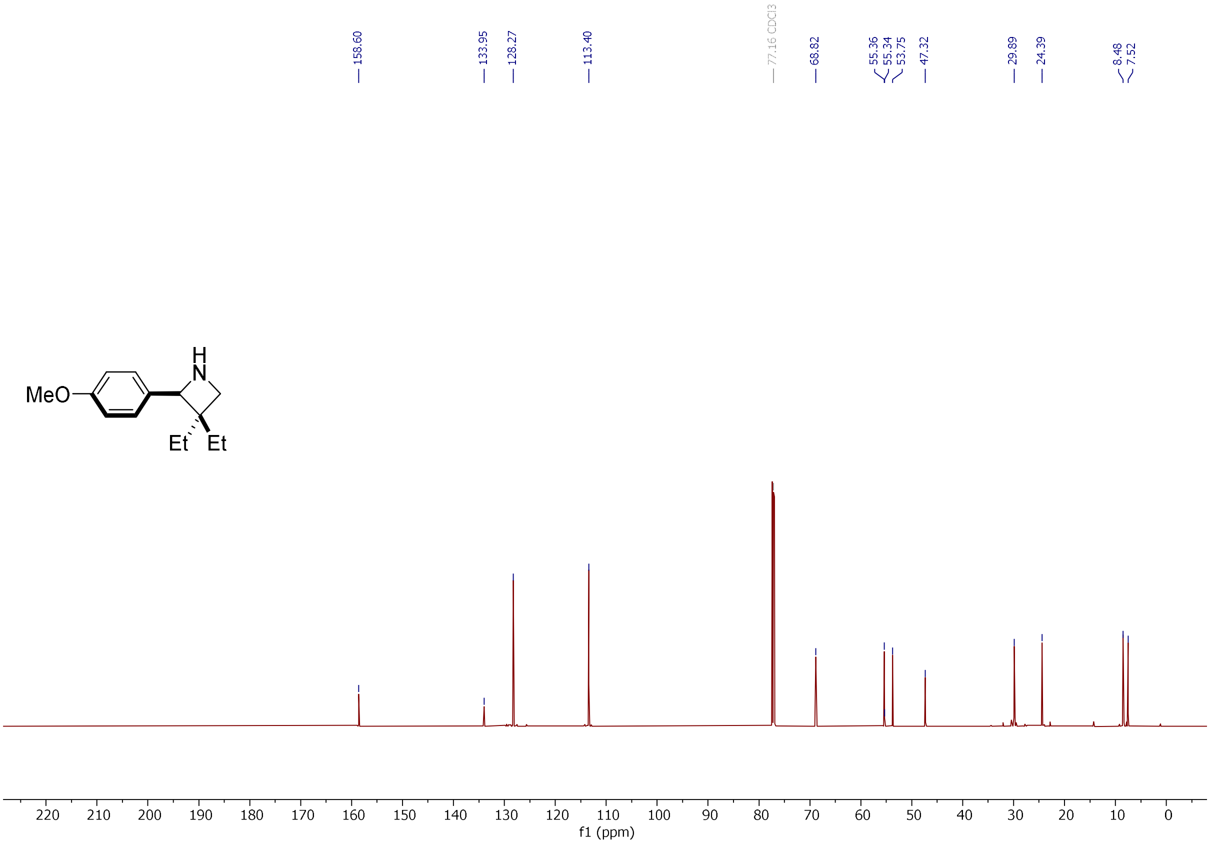

3-(4-((3,3-Diethyl-2-(4-methoxyphenyl)azetidin-1-yl)sulfonyl)piperazin-1-yl)benzo[d]isothiazole (7)

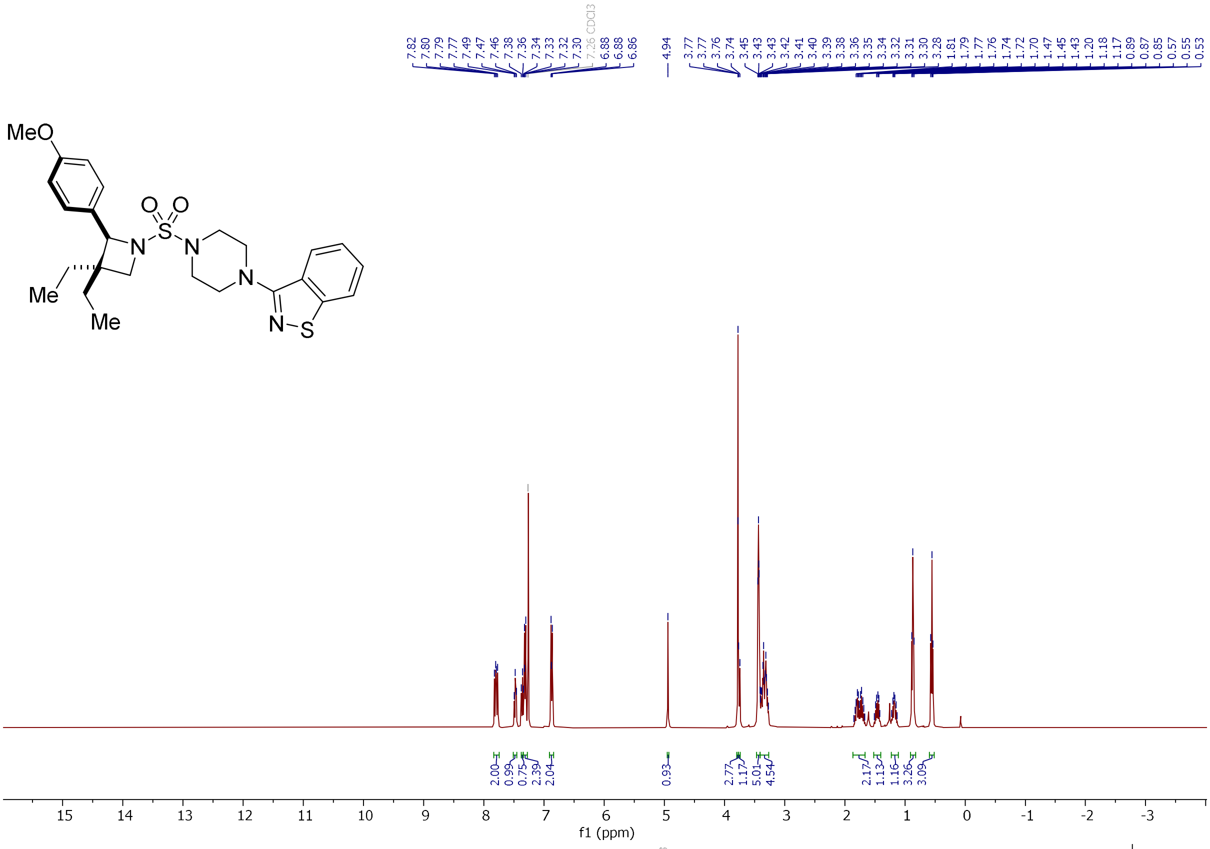

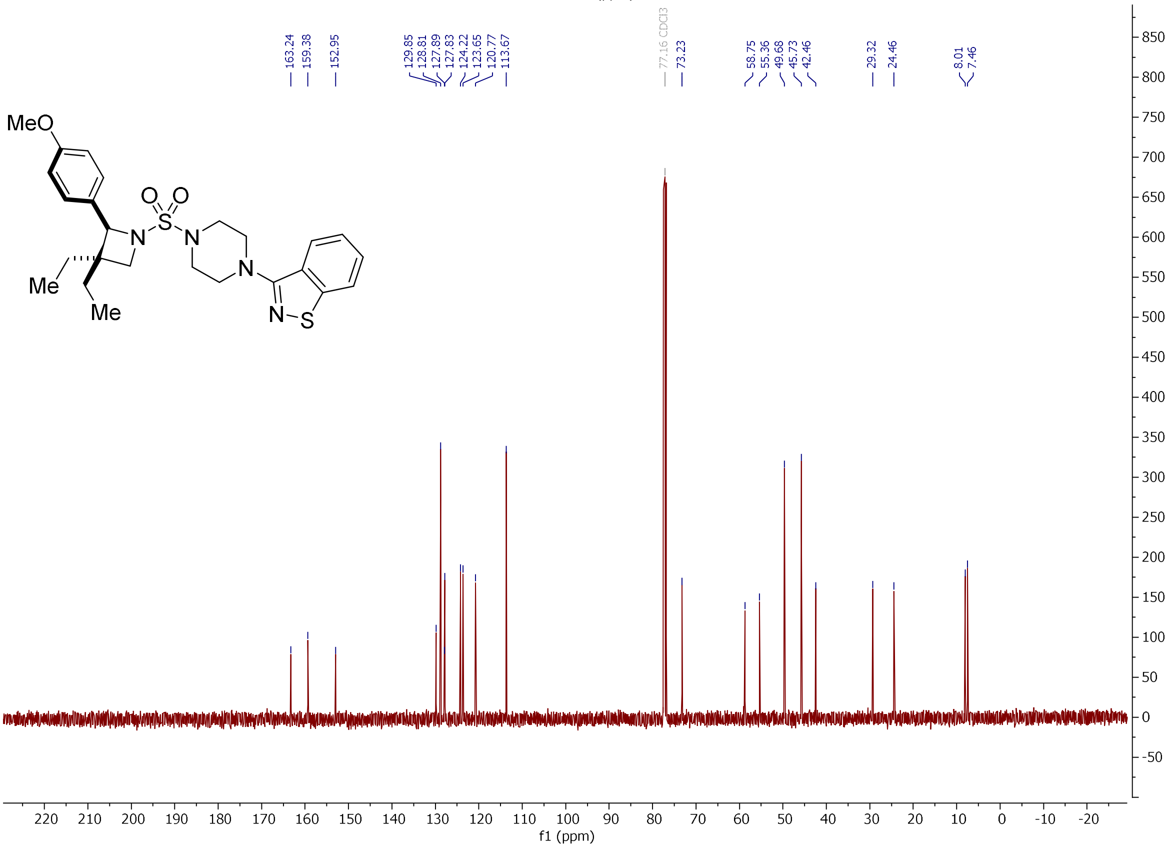

Methyl 3,3-diethyl-1-(fluorosulfonyl)azetidine-2-carboxylate (8)

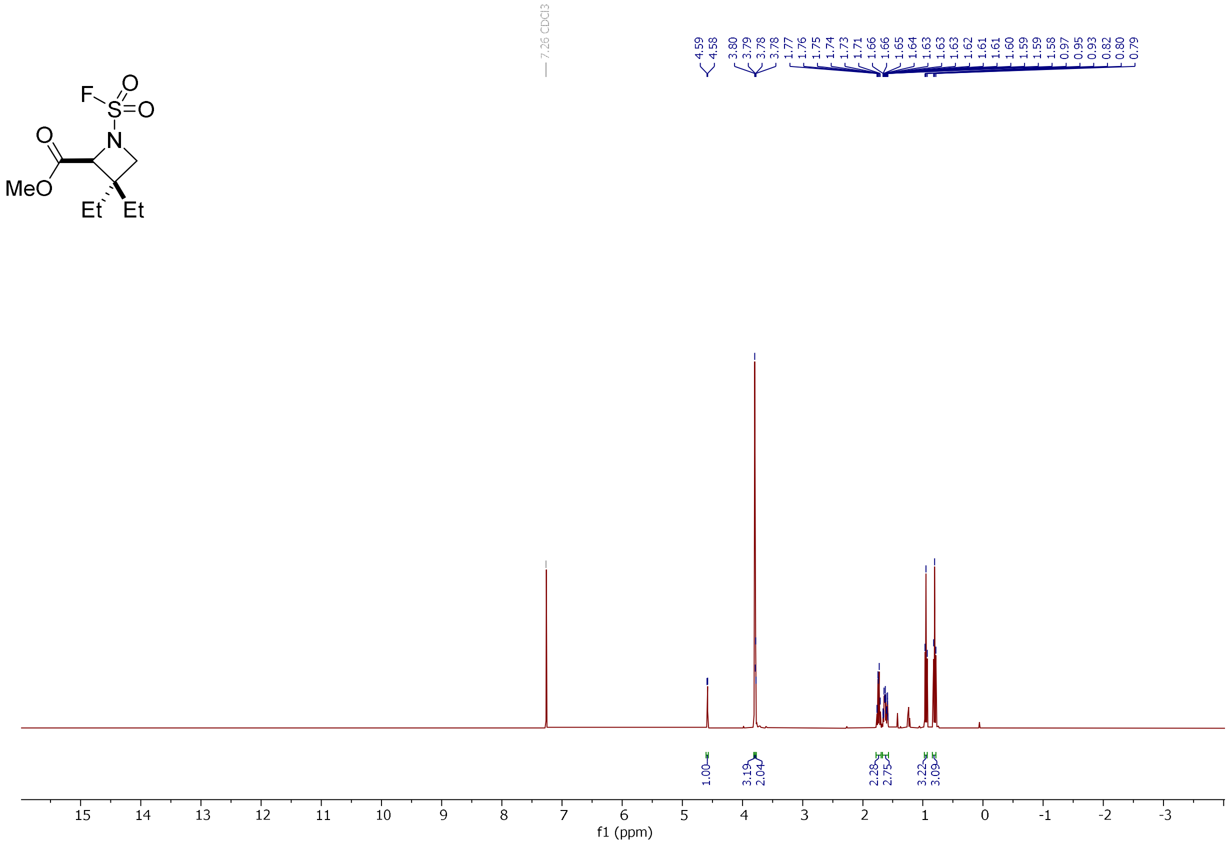

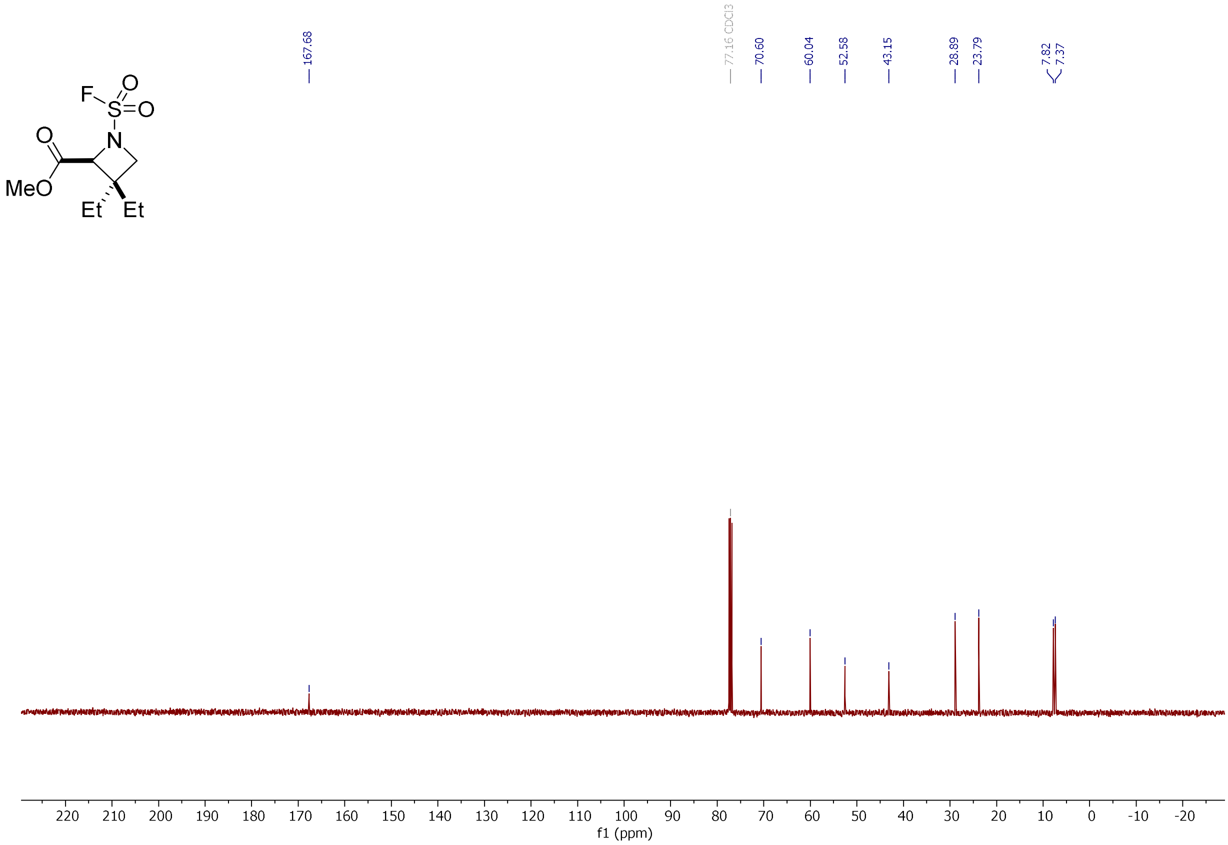

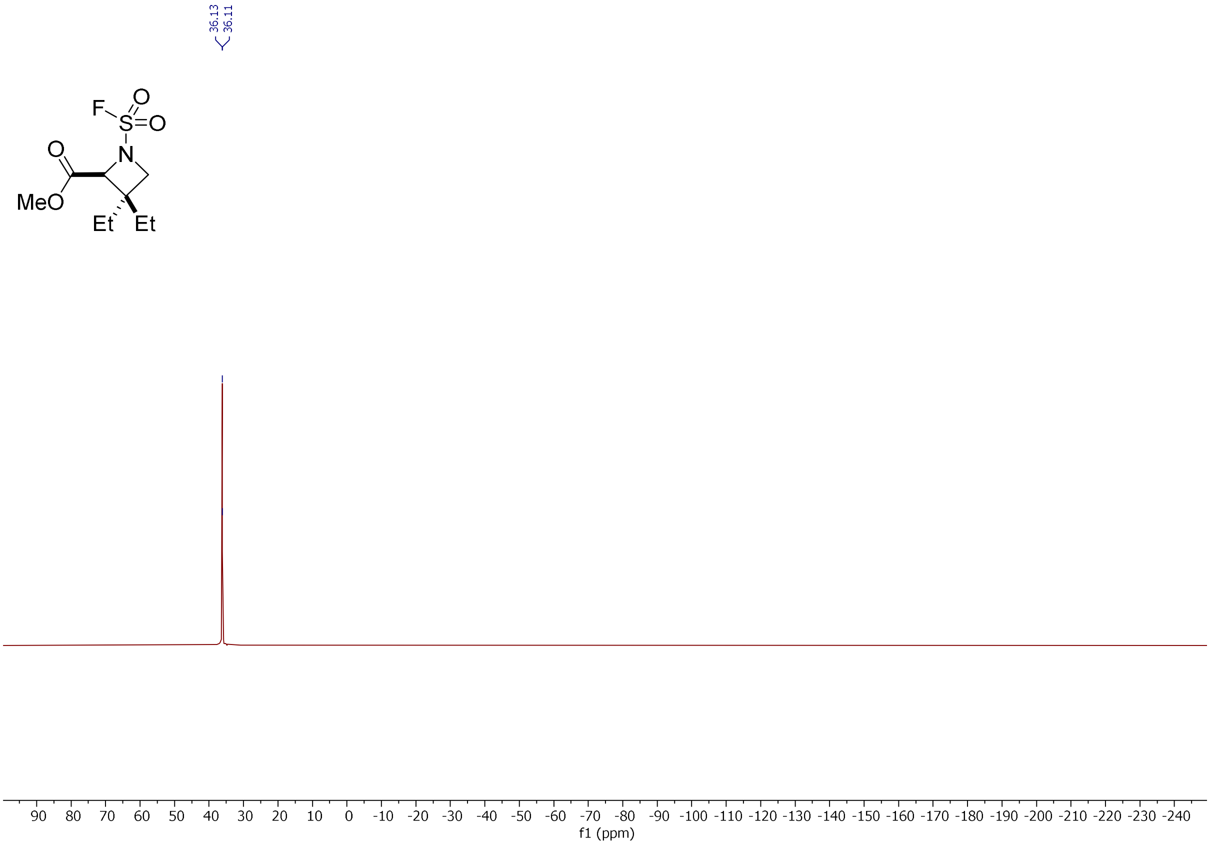

Benzyl ((*S**)-1-((*R**)-3,3-diethyl-2-(4-methoxyphenyl)azetidin-1-yl)-1-oxo-3-phenylpropan-2-yl)carbamate (6) and Benzyl ((*S**)-1-((*S**)-3,3-diethyl-2-(4-methoxyphenyl)azetidin-1-yl)-1-oxo-3-phenylpropan-2-yl)carbamate

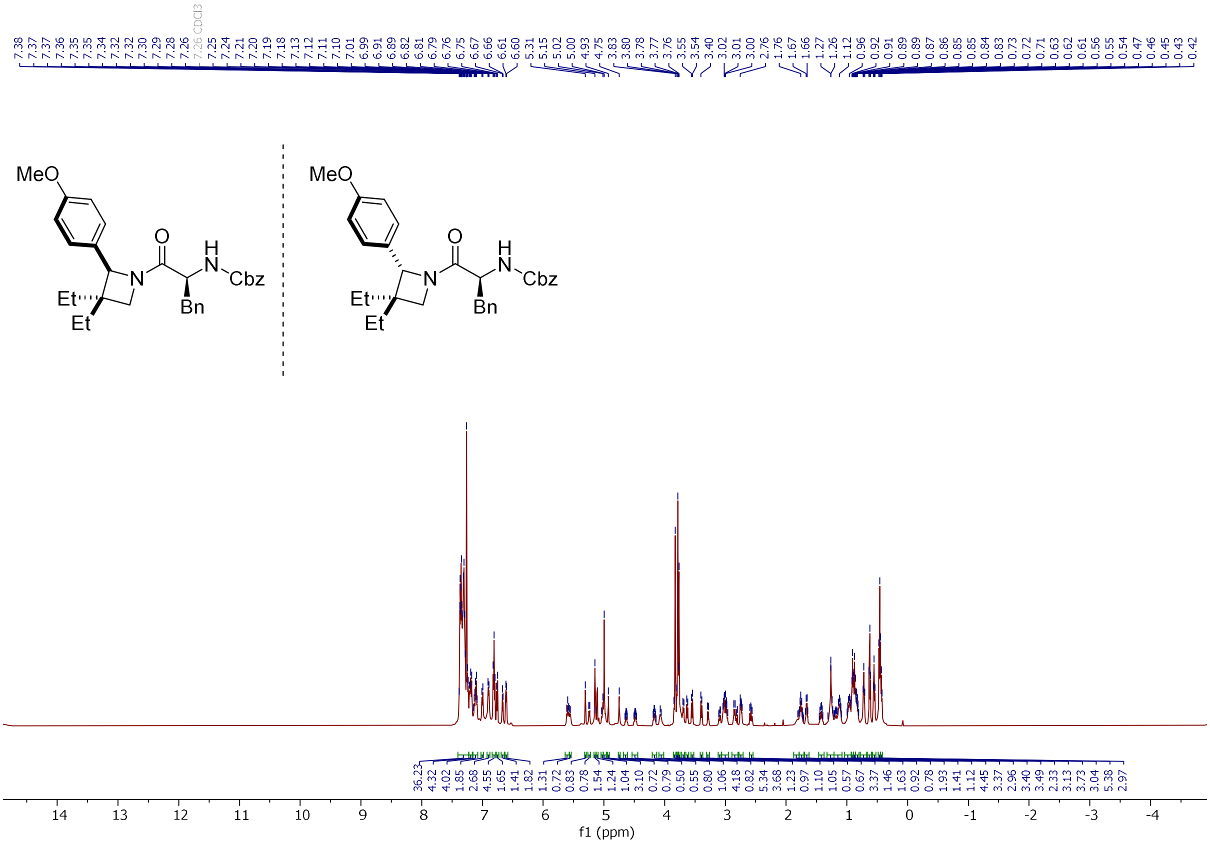

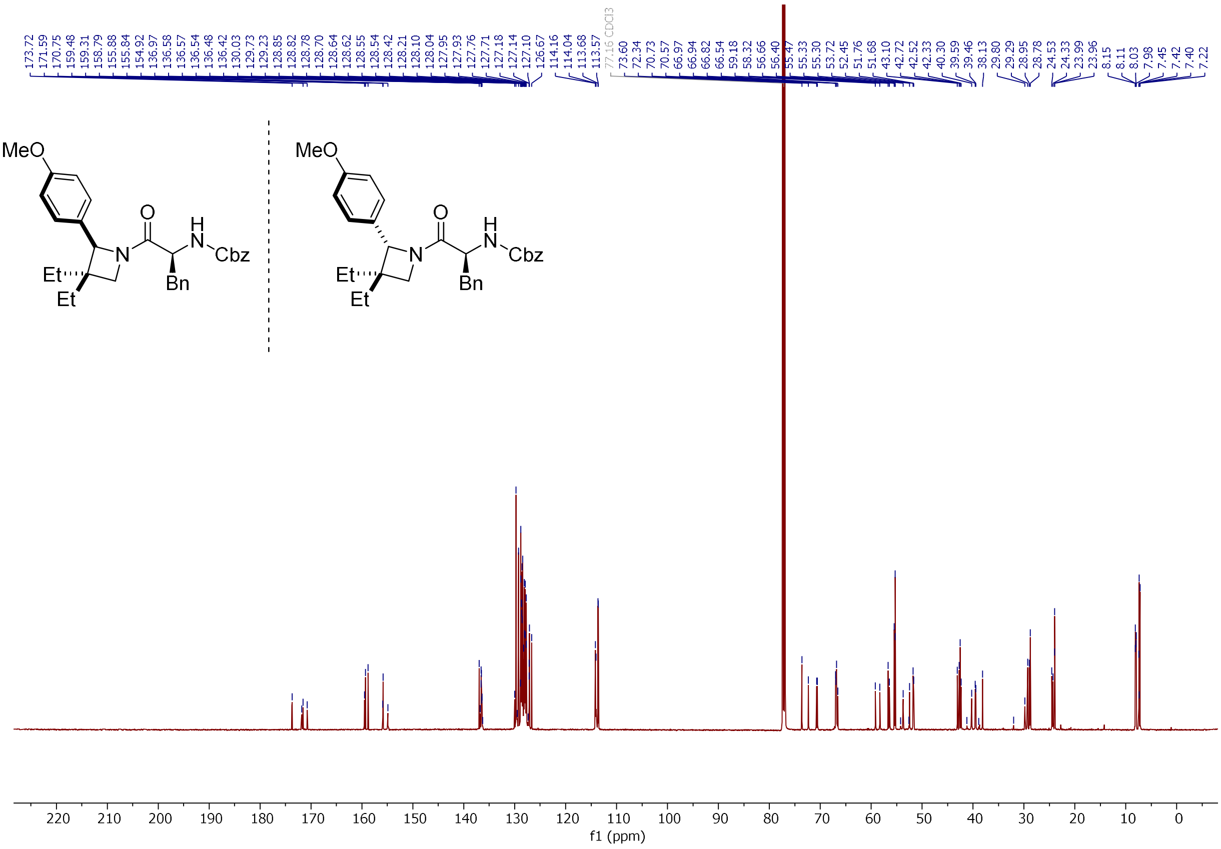

3,3-Diethyl-2-(4-methoxy-3-(quinolin-3-yl)phenyl)azetidine-1-sulfonyl fluoride (6’)

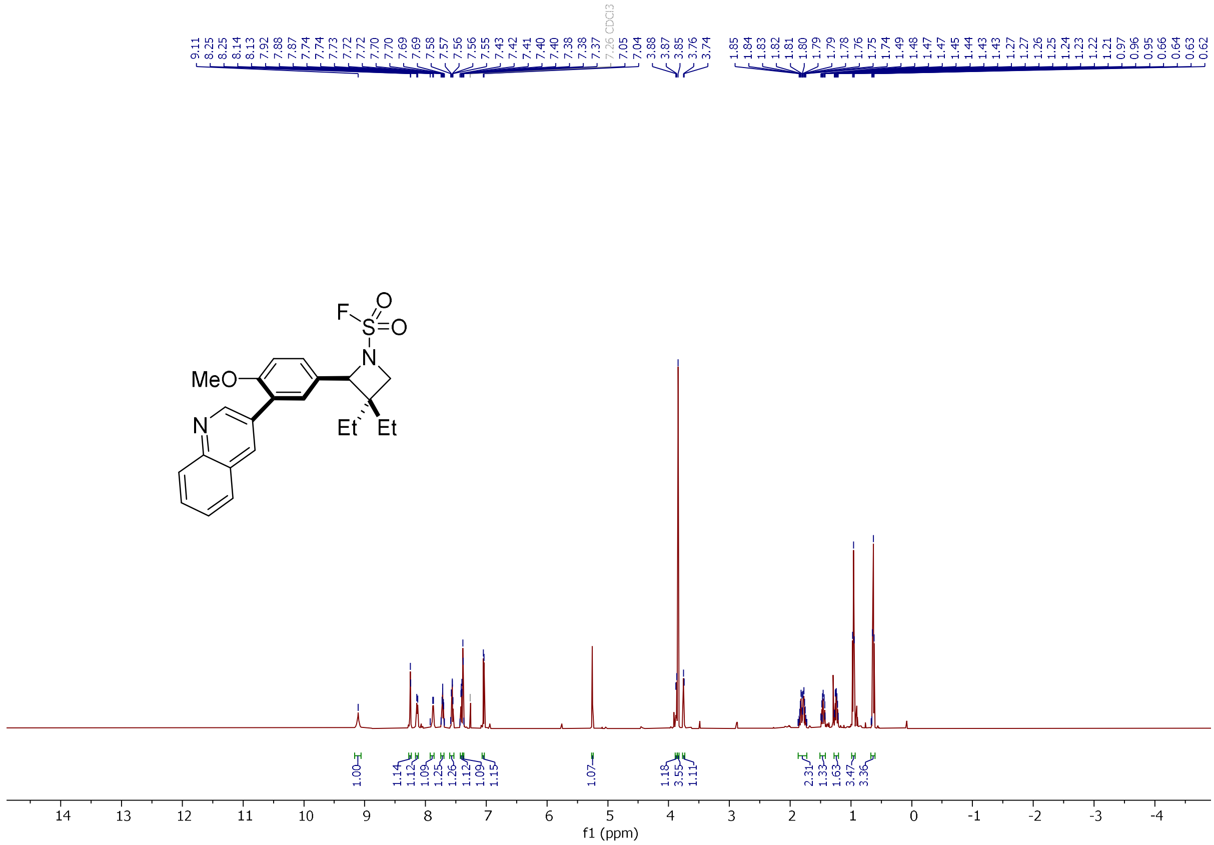

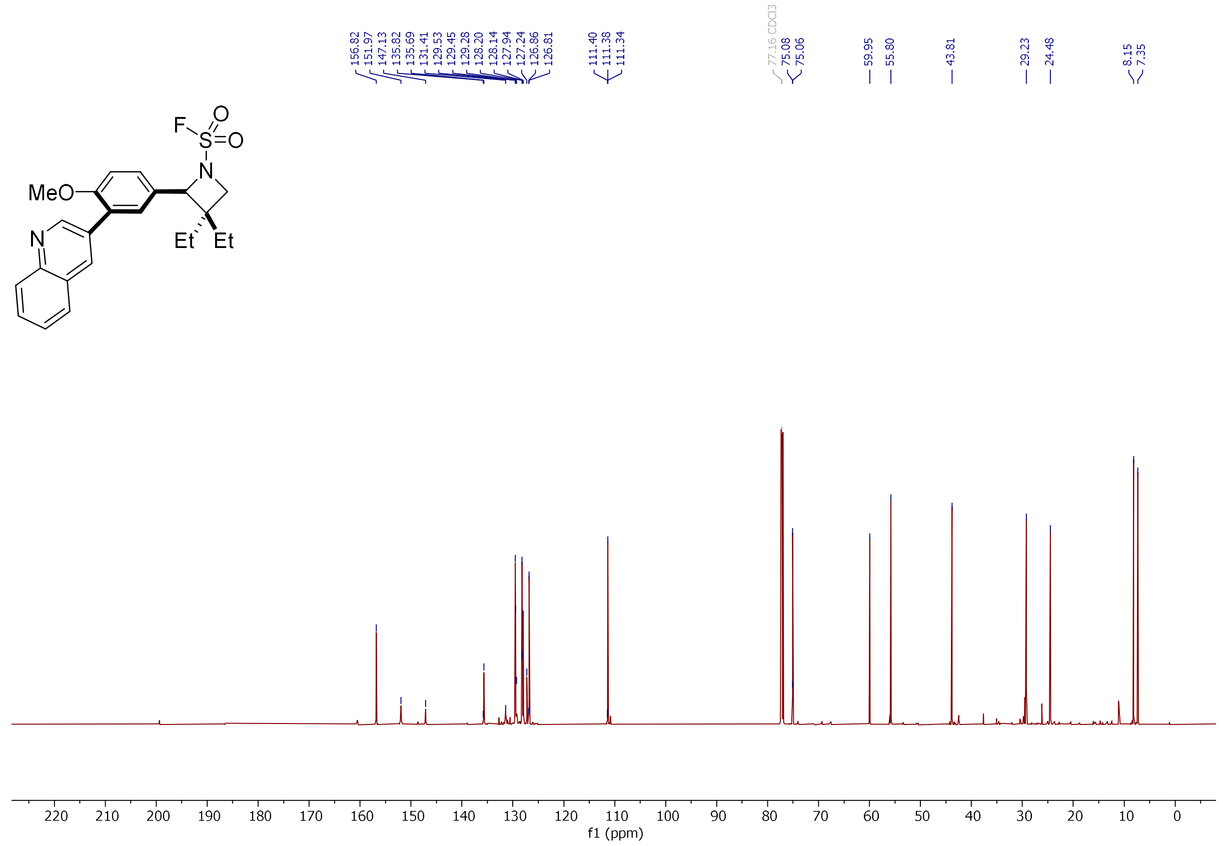
156.82
151.97
147.13
135.82
135.69
131.41
129.53
129.45
129.28
128.20
128.14
127.94
127.24
126.86
126.81
111.40
111.38
111.34
77.16 CDCl3
75.08
75.06
59.95
55.80
43.81
29.23
24.48
8.15
7.35
F
S
O
O
N
MeO
N
Et
Et
220 210 200 190 180 170 160 150 140 130 120 110 100 90 80 70 60 50 40 30 20 10 0
f1 (ppm)

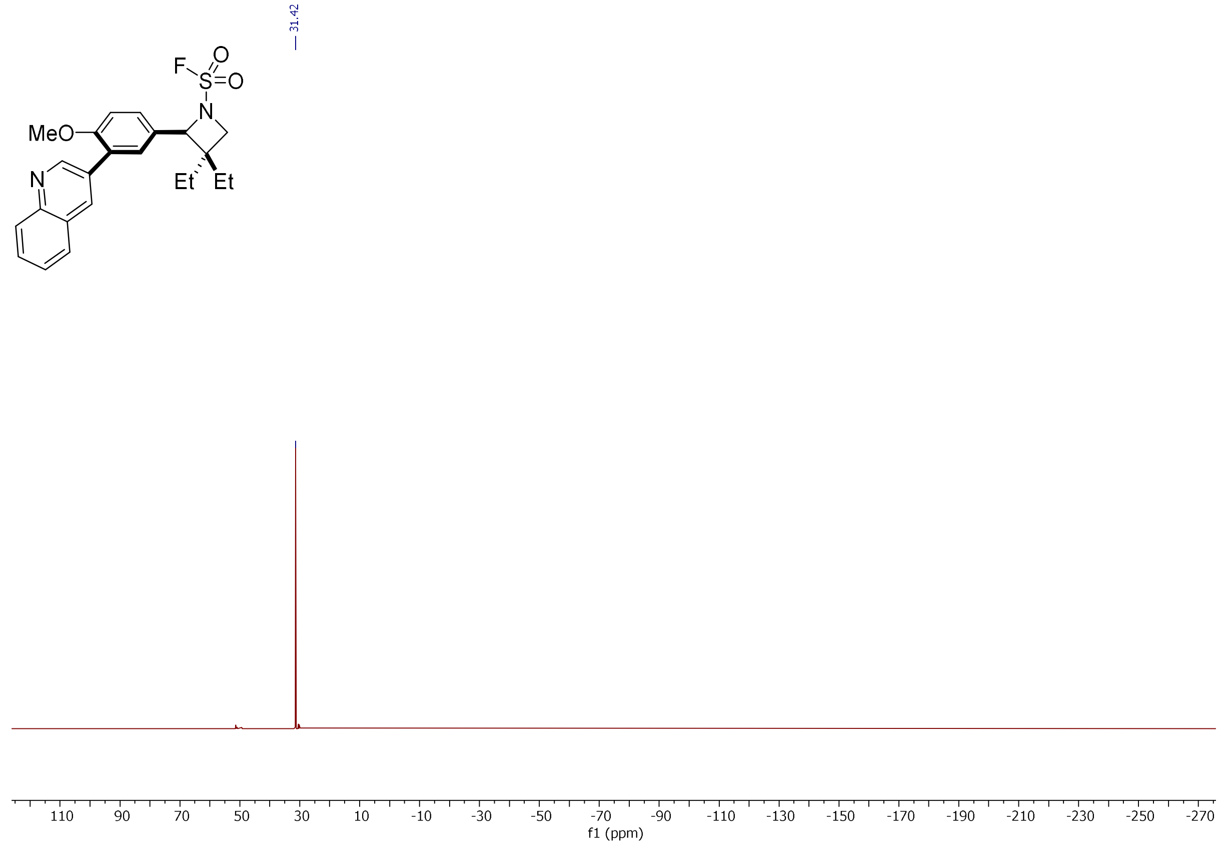
31.42
F
S
O
O
N
MeO
N
Et
Et
110 90 70 50 30 10 -10 -30 -50 -70 -90 -110 -130 -150 -170 -190 -210 -230 -250 -270
f1 (ppm)

Sulfamoyl fluoride Imines

(4-Methoxybenzylidene)sulfamoyl fluoride (1)

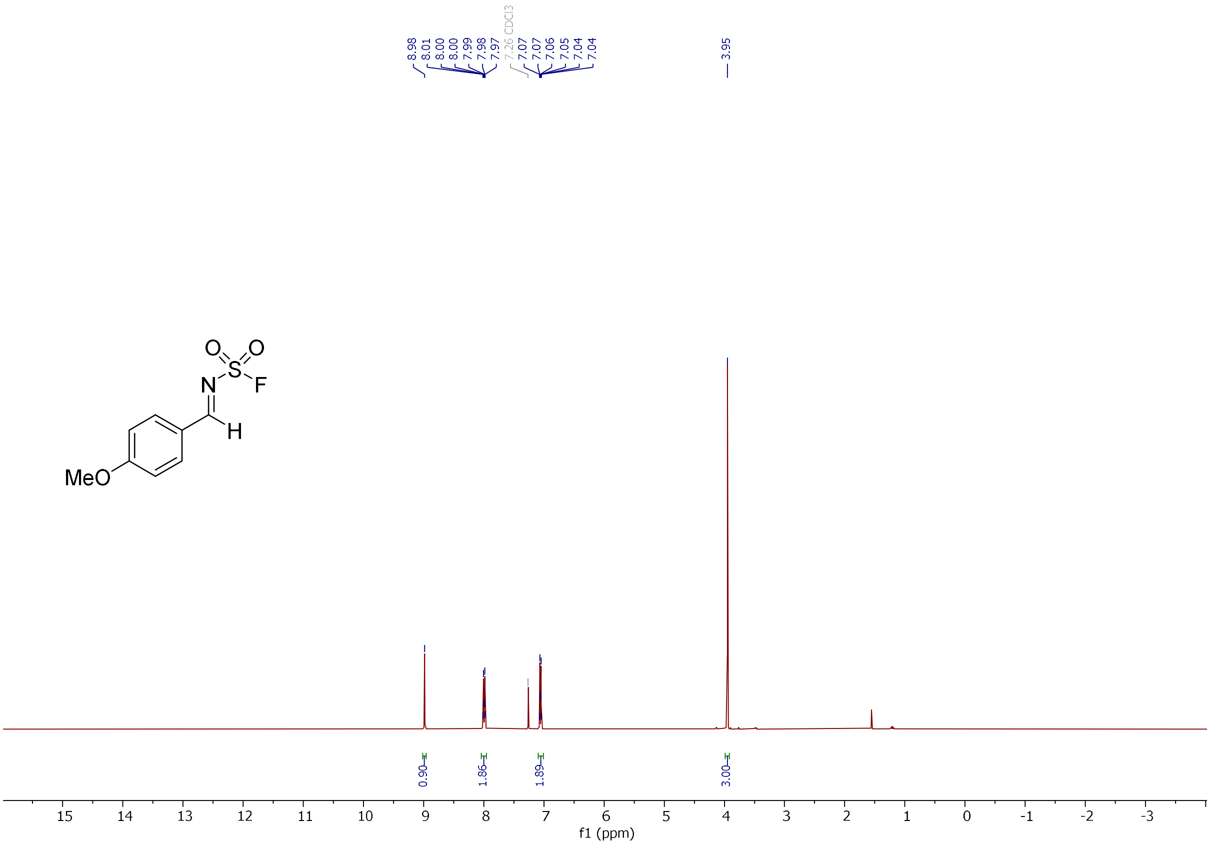

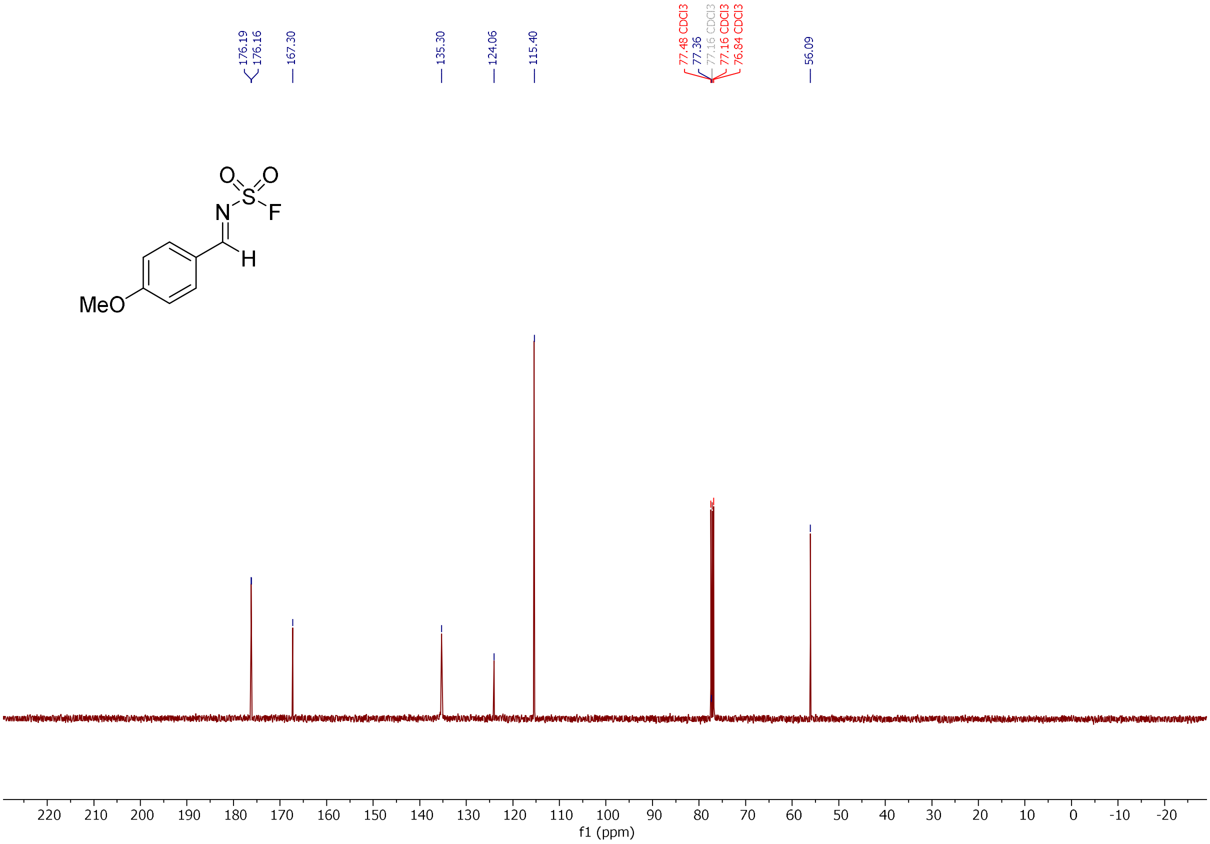

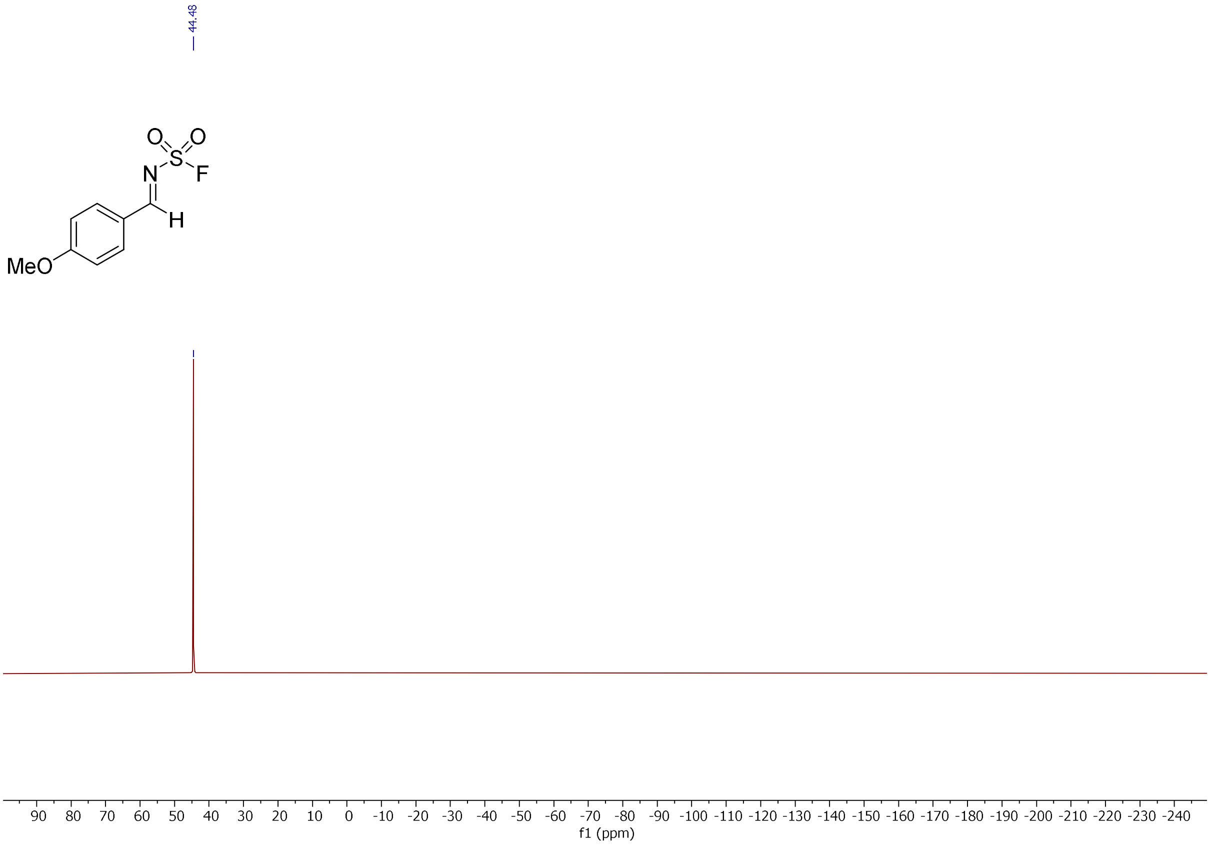

44.48
O
S
O
N
F
H
MeO
90 80 70 60 50 40 30 20 10 0 -10 -20 -30 -40 -50 -60 -70 -80 -90 -100 -110 -120 -130 -140 -150 -160 -170 -180 -190 -200 -210 -220 -230 -240
f1 (ppm)

(2-Methoxybenzylidene)sulfamoyl fluoride (I-2)

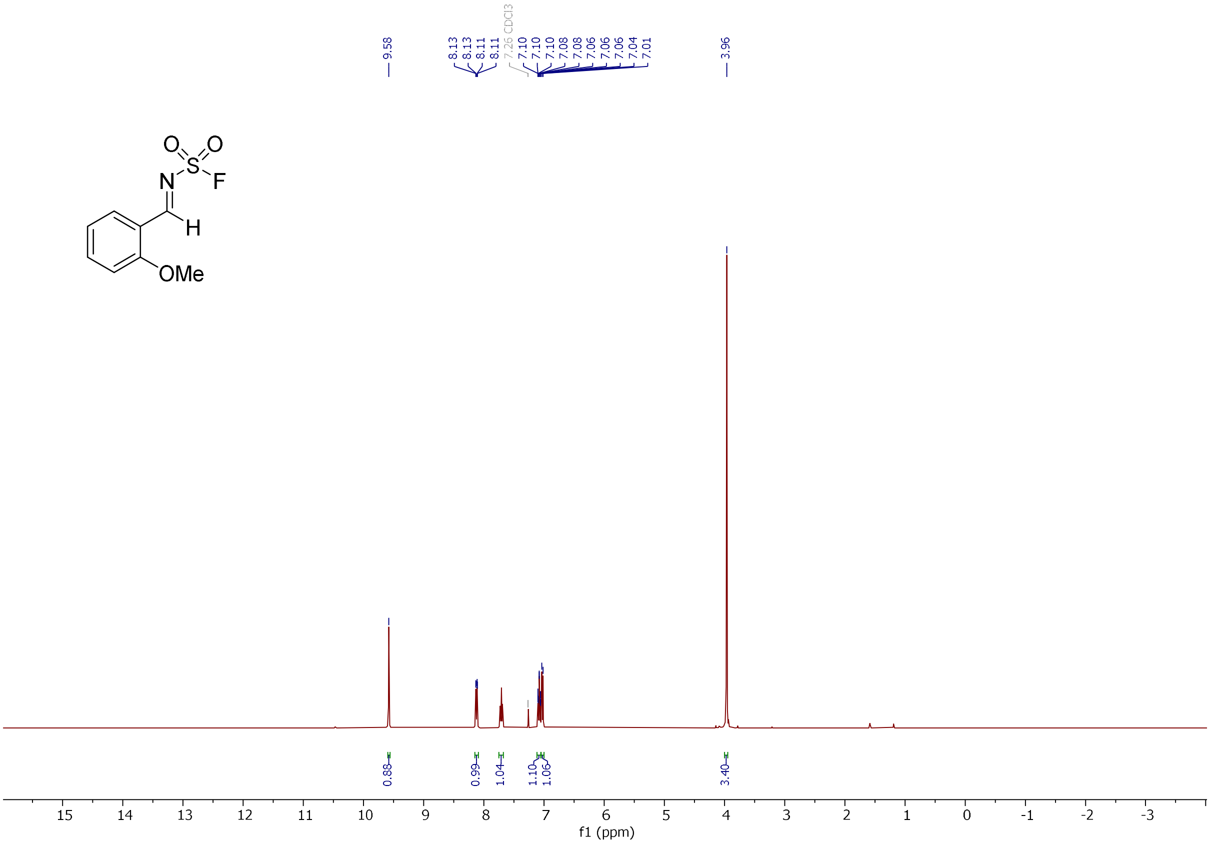

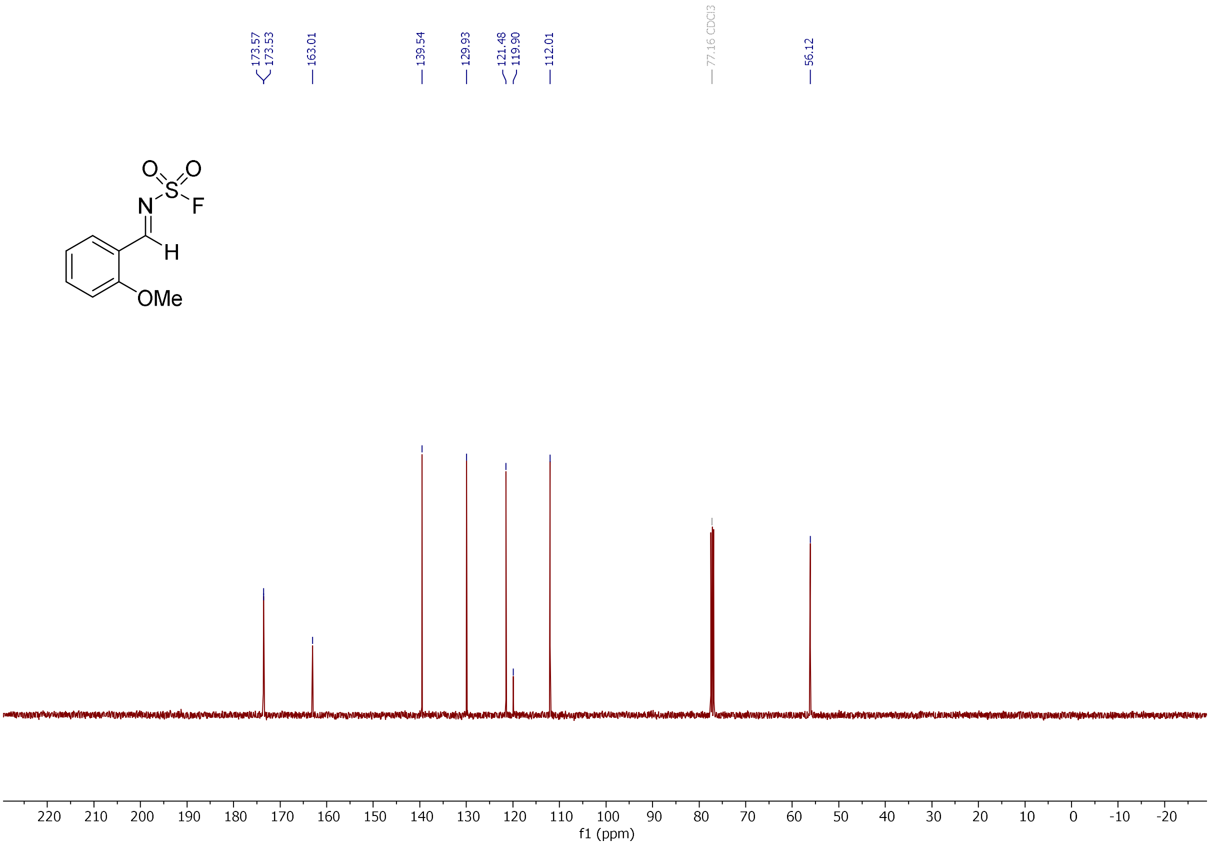

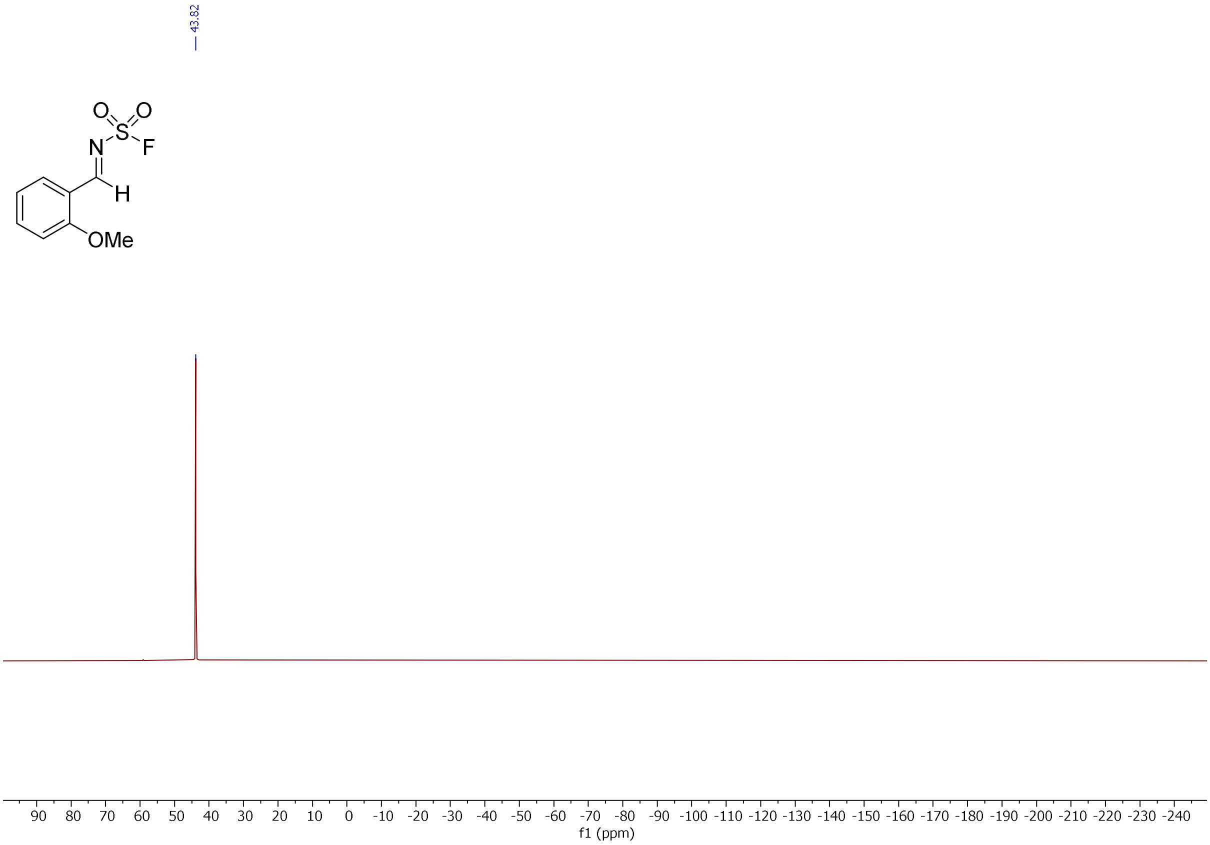

(2-Fluoro-6-methoxybenzylidene)sulfamoyl fluoride (I-3)

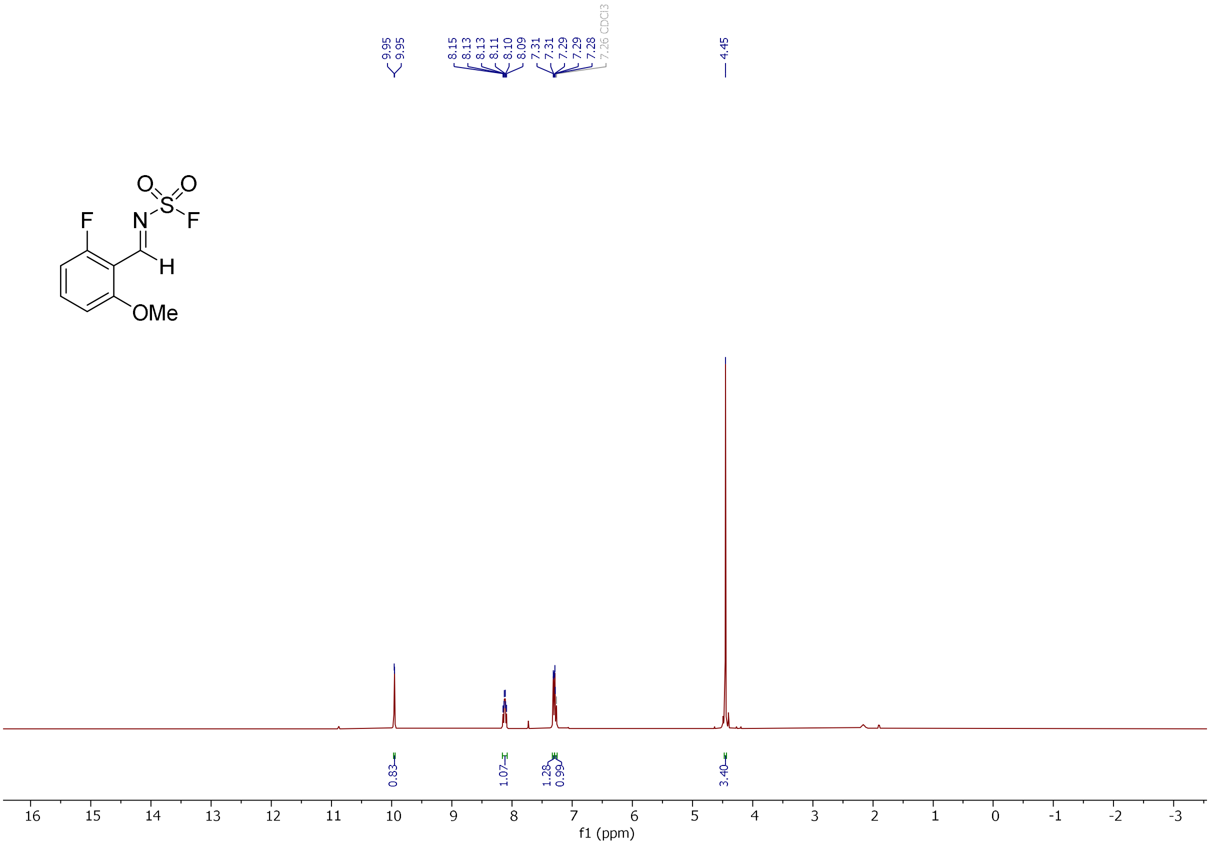

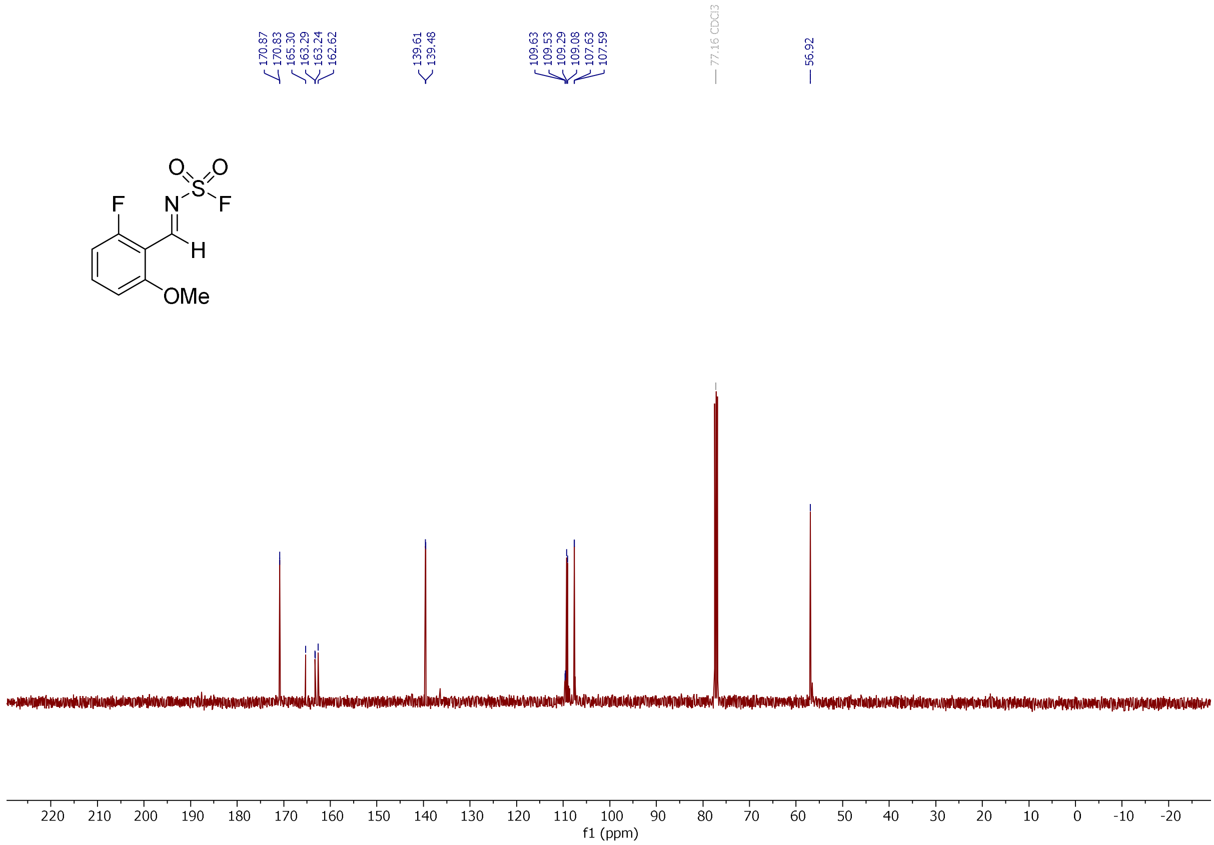
170.87
170.83
165.30
163.29
163.24
162.62
139.61
139.48
109.63
109.53
109.29
109.08
107.63
107.59
77.16 CDCl3
56.92
O
S
O
F
N
F
H
OMe
220 210 200 190 180 170 160 150 140 130 120 110 100 90 80 70 60 50 40 30 20 10 0 -10 -20
f1 (ppm)

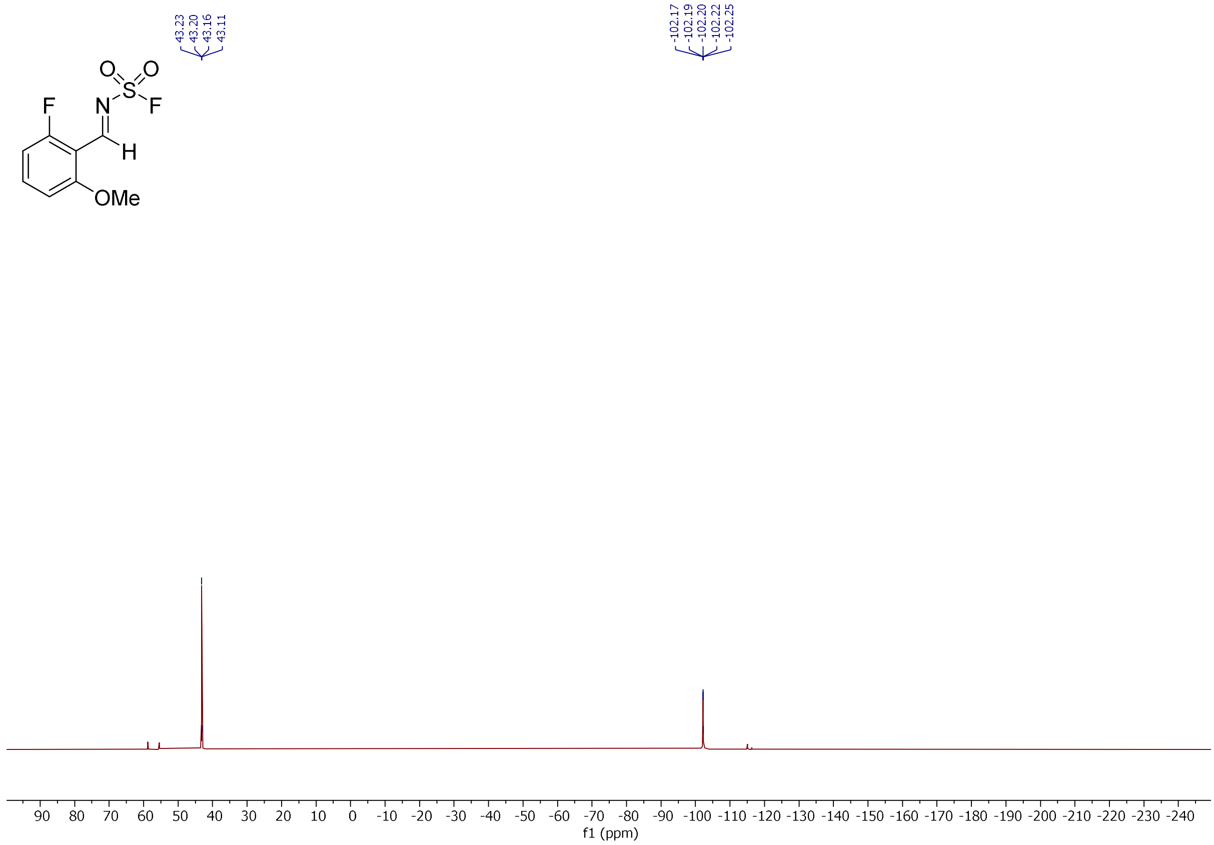
43.23
43.20
43.16
43.11
-102.17
-102.19
-102.20
-102.22
-102.25
O
S
O
F
N
F
H
OMe
90 80 70 60 50 40 30 20 10 0 -10 -20 -30 -40 -50 -60 -70 -80 -90 -100 -110 -120 -130 -140 -150 -160 -170 -180 -190 -200 -210 -220 -230 -240
f1 (ppm)

Benzylidenesulfamoyl fluoride (I-4)

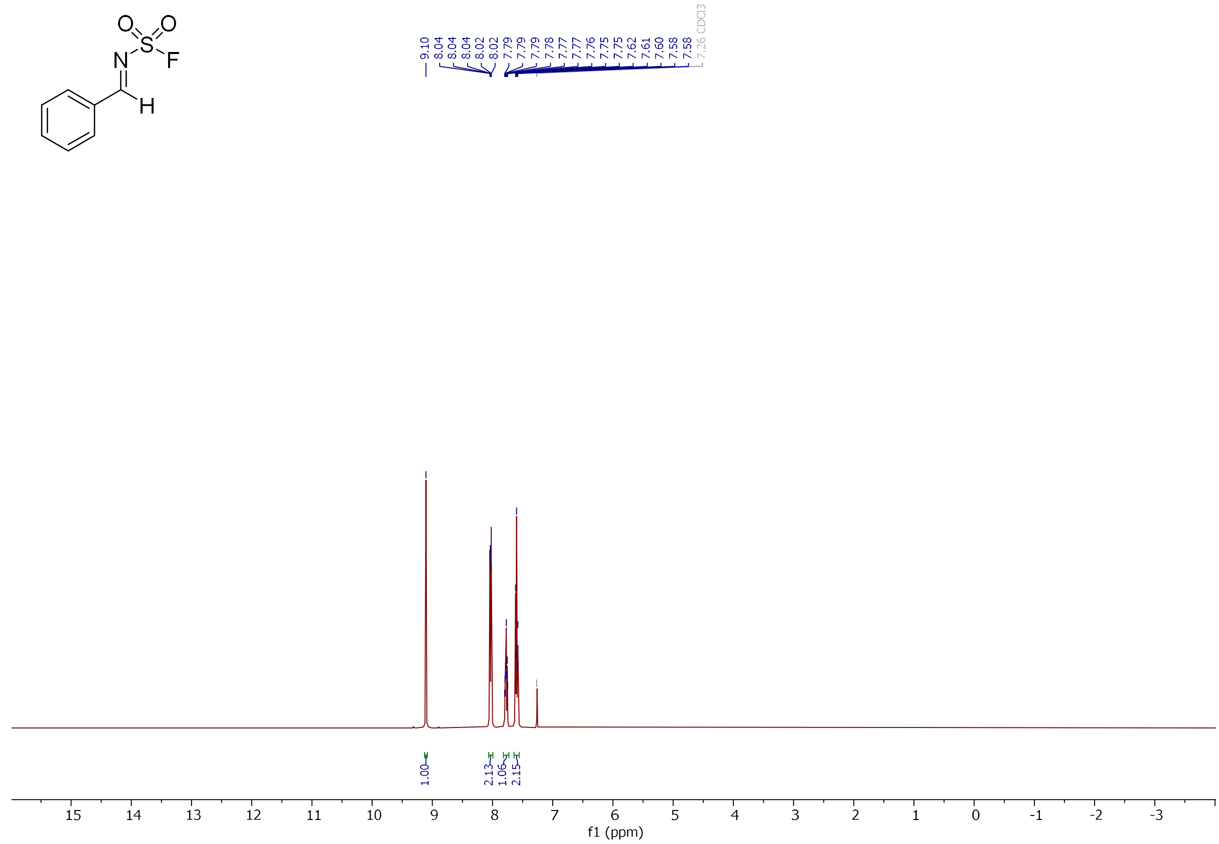

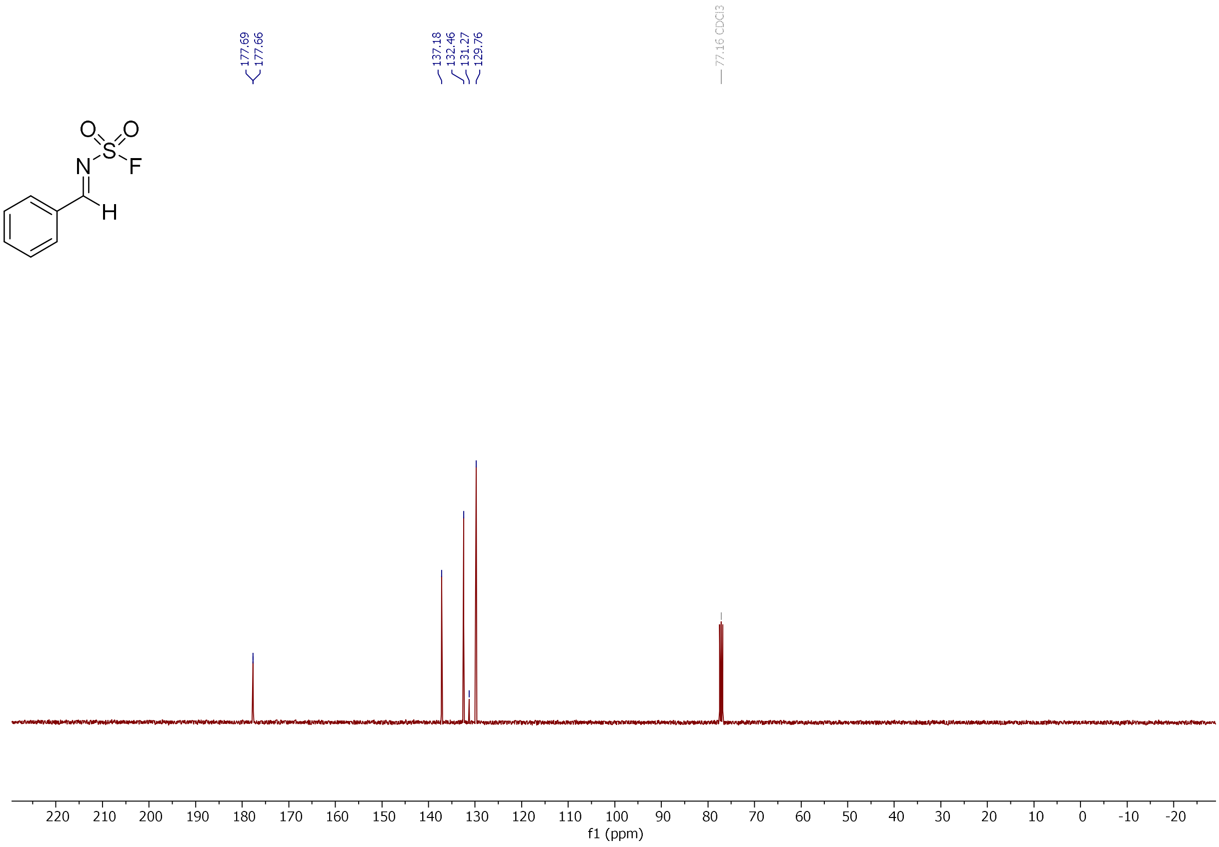

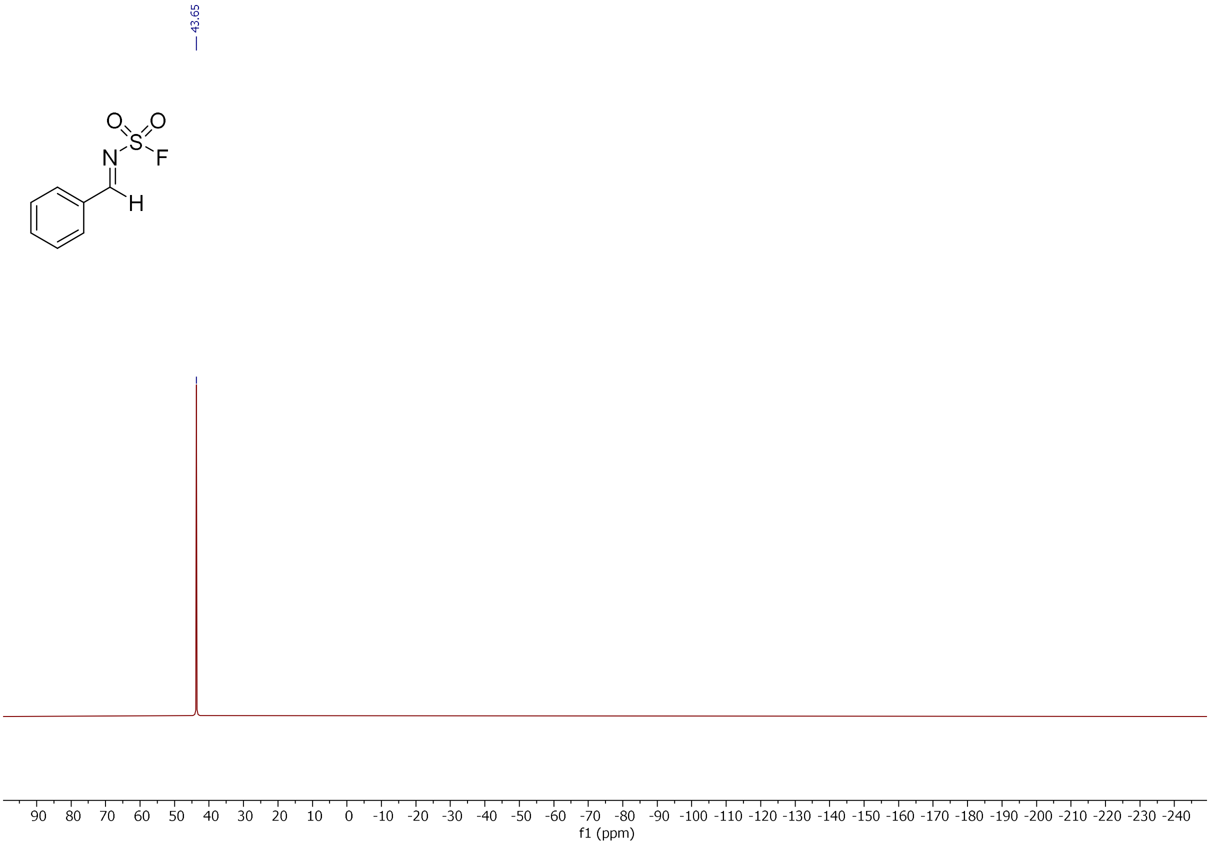

(4-Methylbenzylidene)sulfamoyl fluoride (I-5)

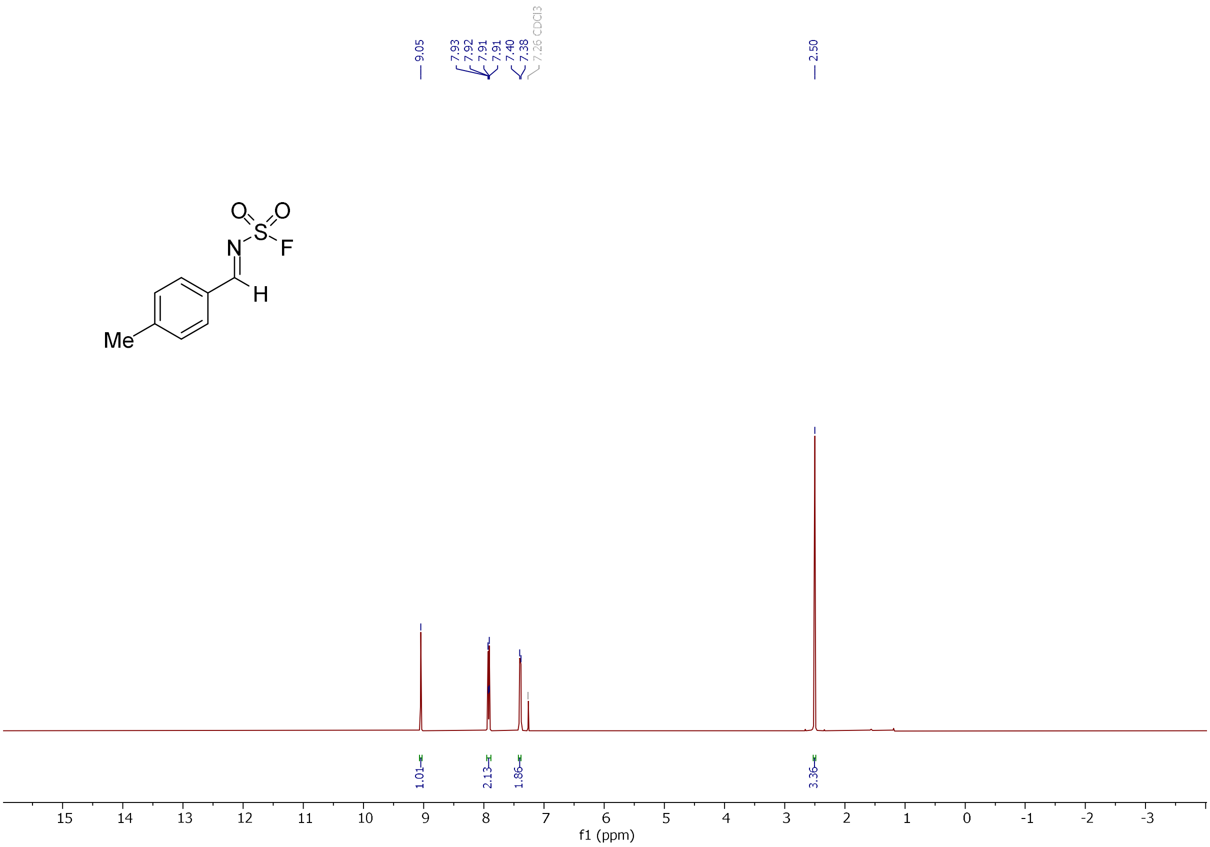

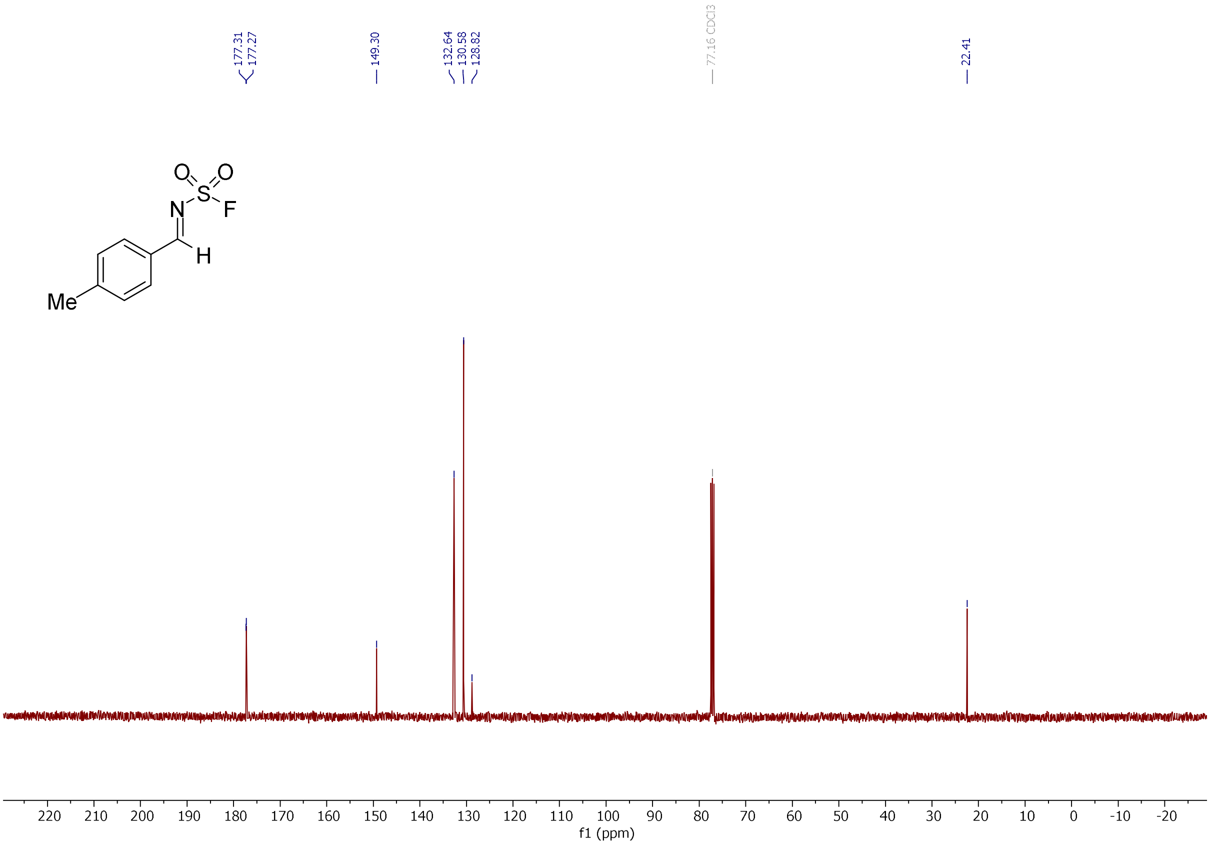
177.31
177.27
149.30
132.64
130.58
128.82
77.16 CDCl3
22.41
Me
N
S
O
O
F
H
f1 (ppm)

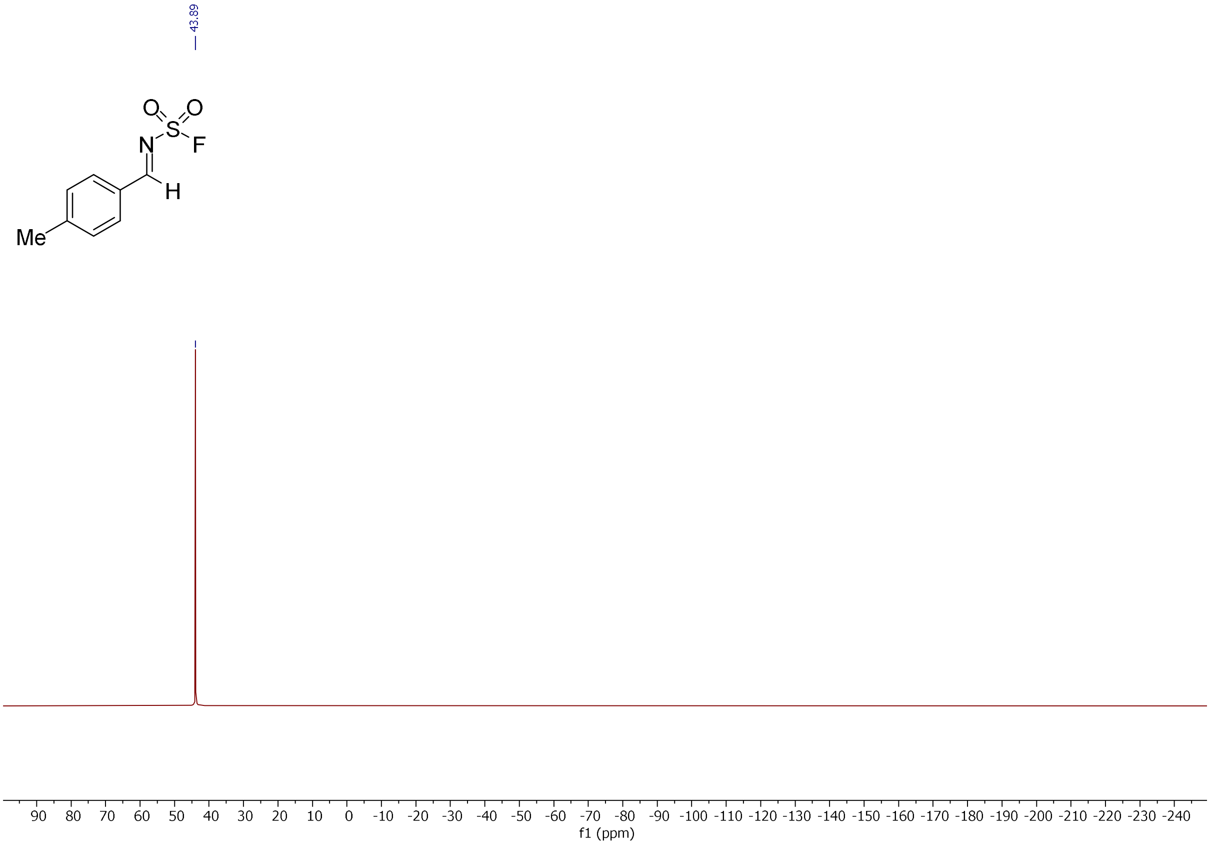
43.89
Me
N
S
O
O
F
H
f1 (ppm)

(4-(Trimethylsilyl)benzylidene)sulfamoyl fluoride (I-6)

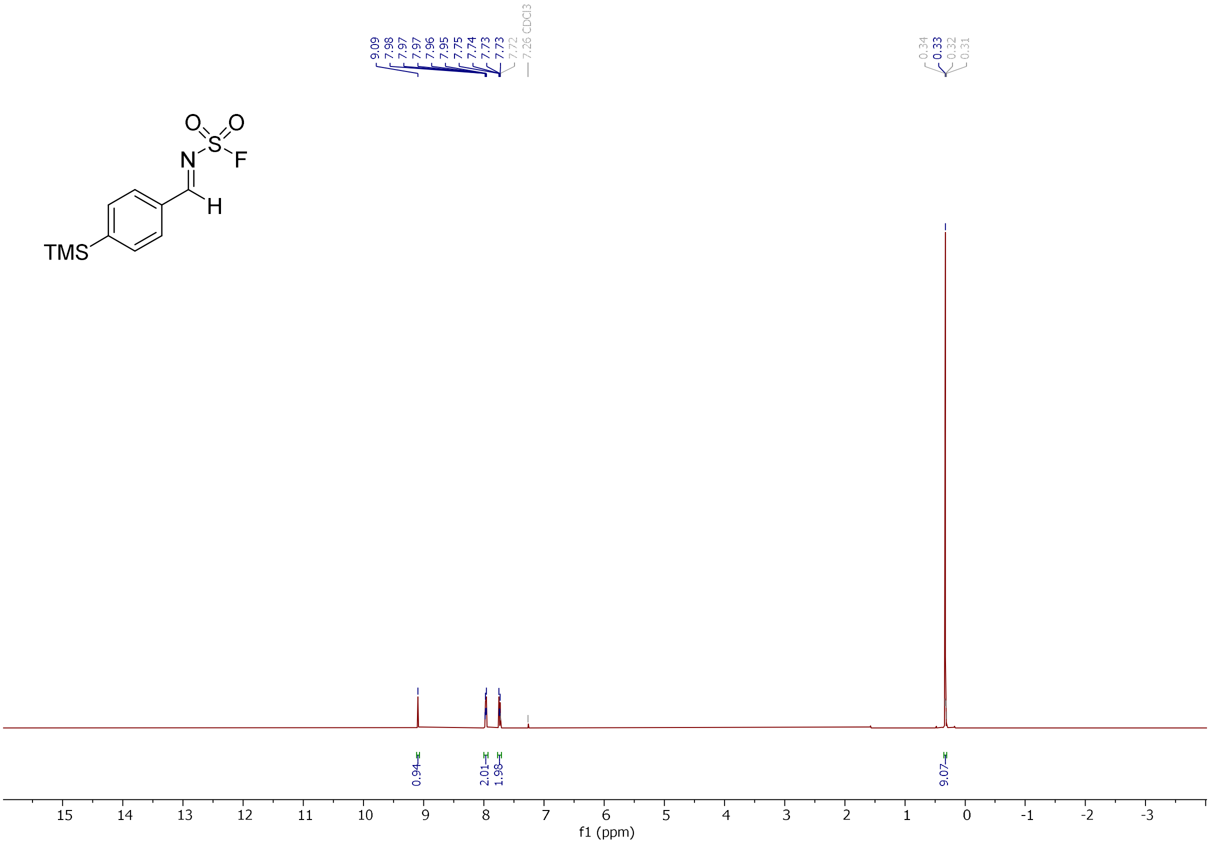

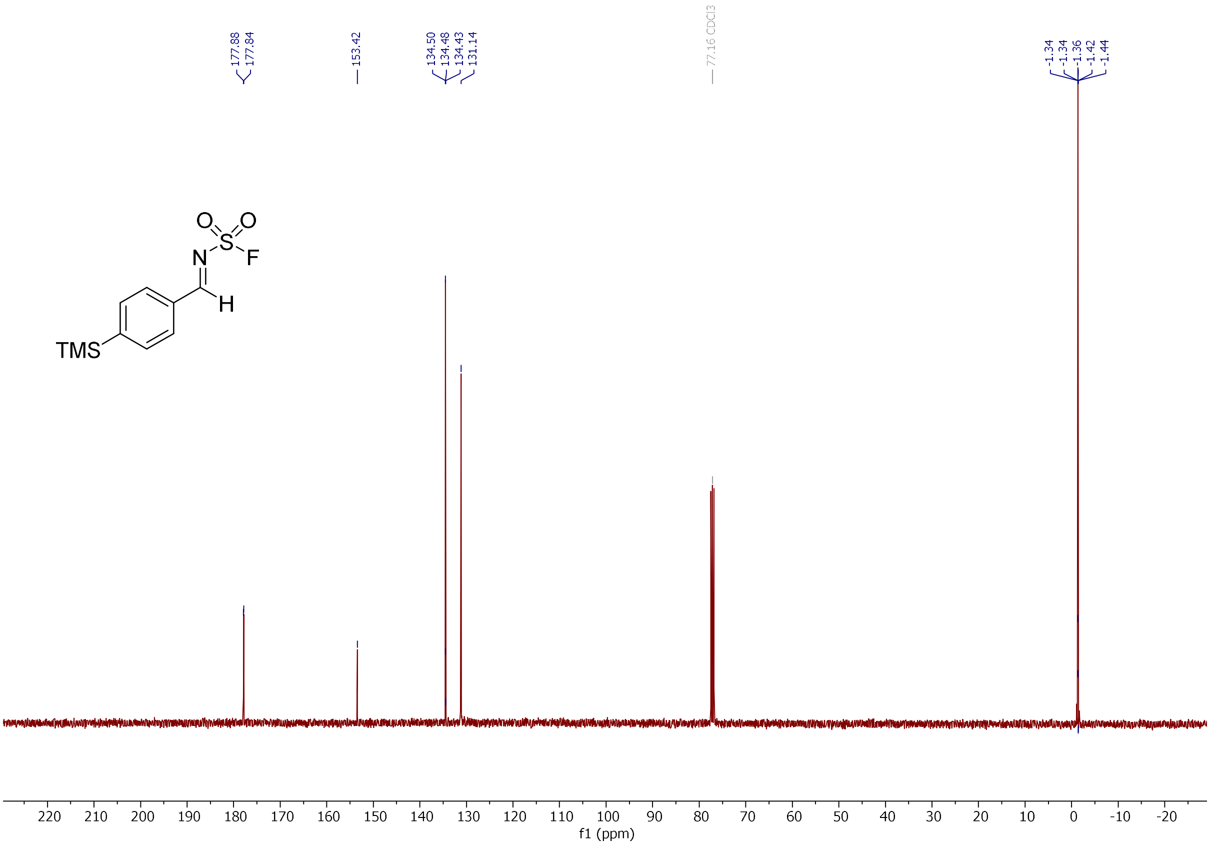

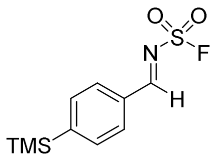
TMS
H
N
S
O
O
F

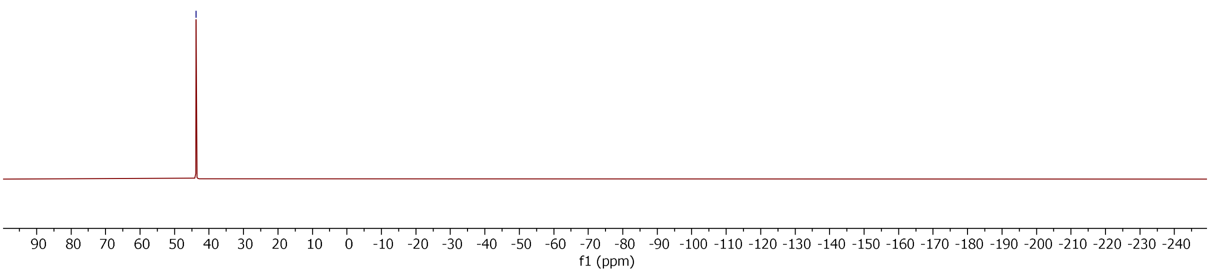
43.74
90 80 70 60 50 40 30 20 10 0 -10 -20 -30 -40 -50 -60 -70 -80 -90 -100 -110 -120 -130 -140 -150 -160 -170 -180 -190 -200 -210 -220 -230 -240
f1 (ppm)

(4-Fluorobenzylidene)sulfamoyl fluoride (I-7)

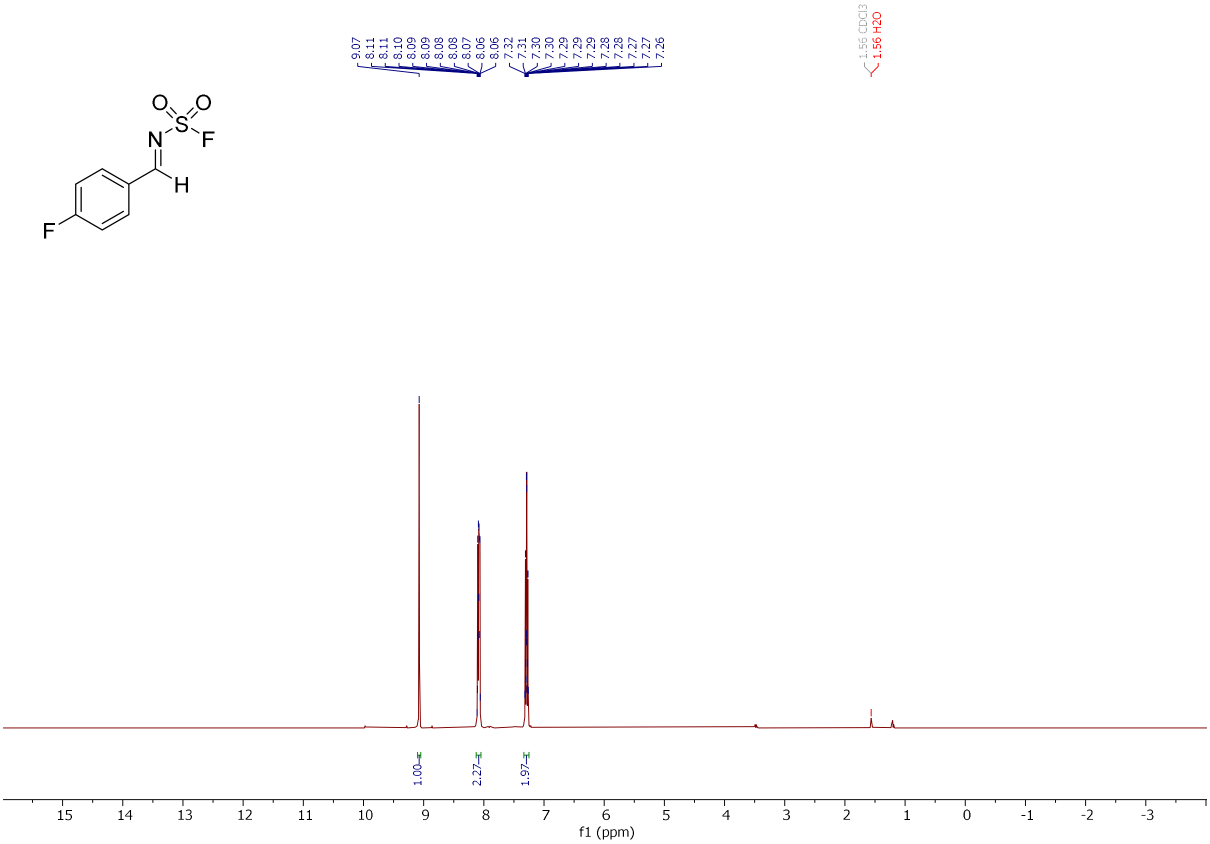

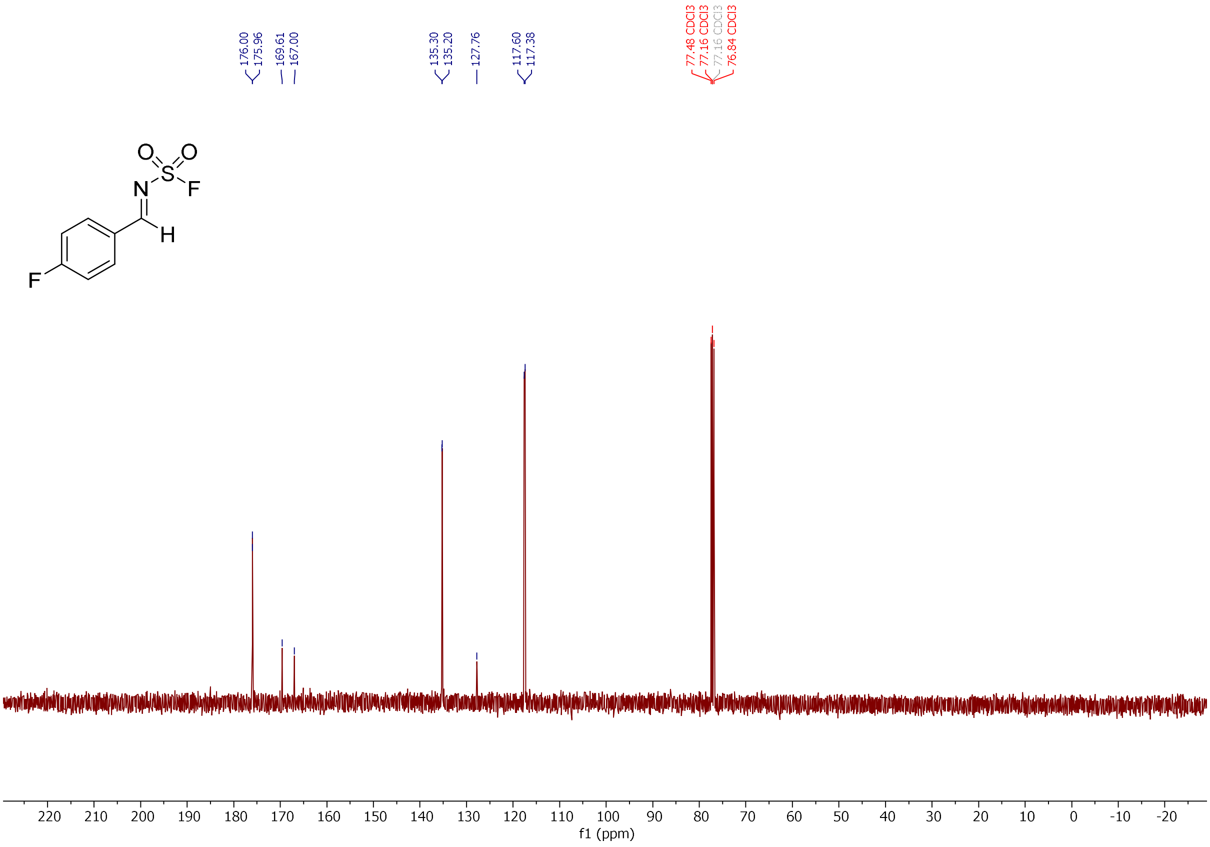

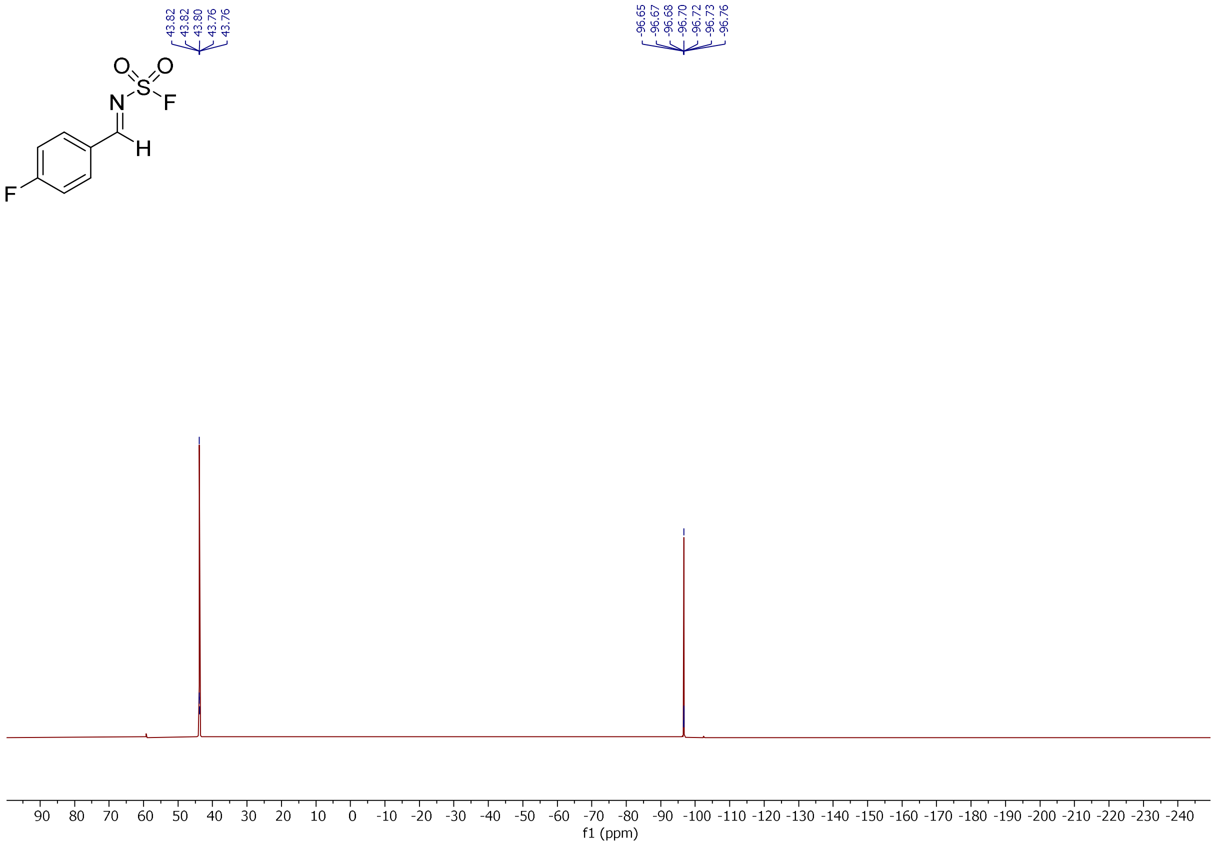

(4-Chlorobenzylidene)sulfamoyl fluoride (I-8)

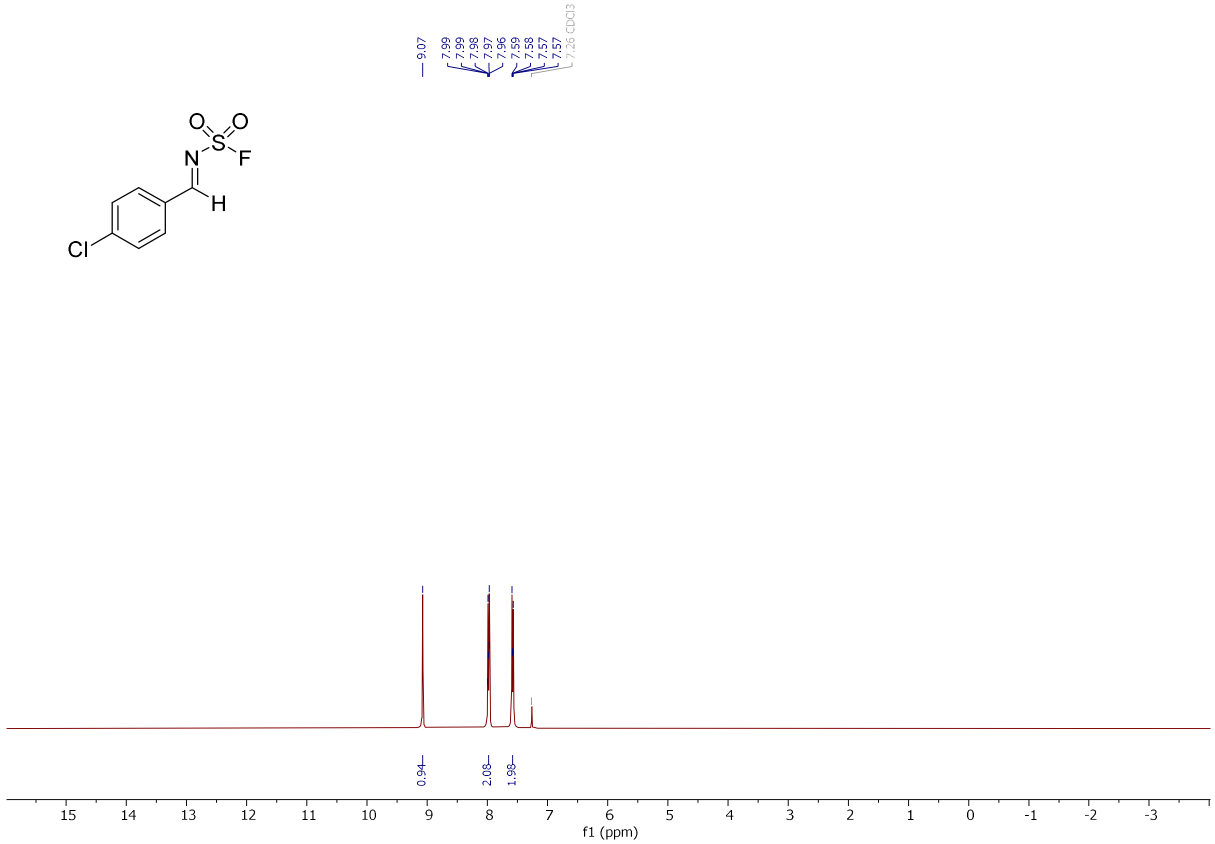

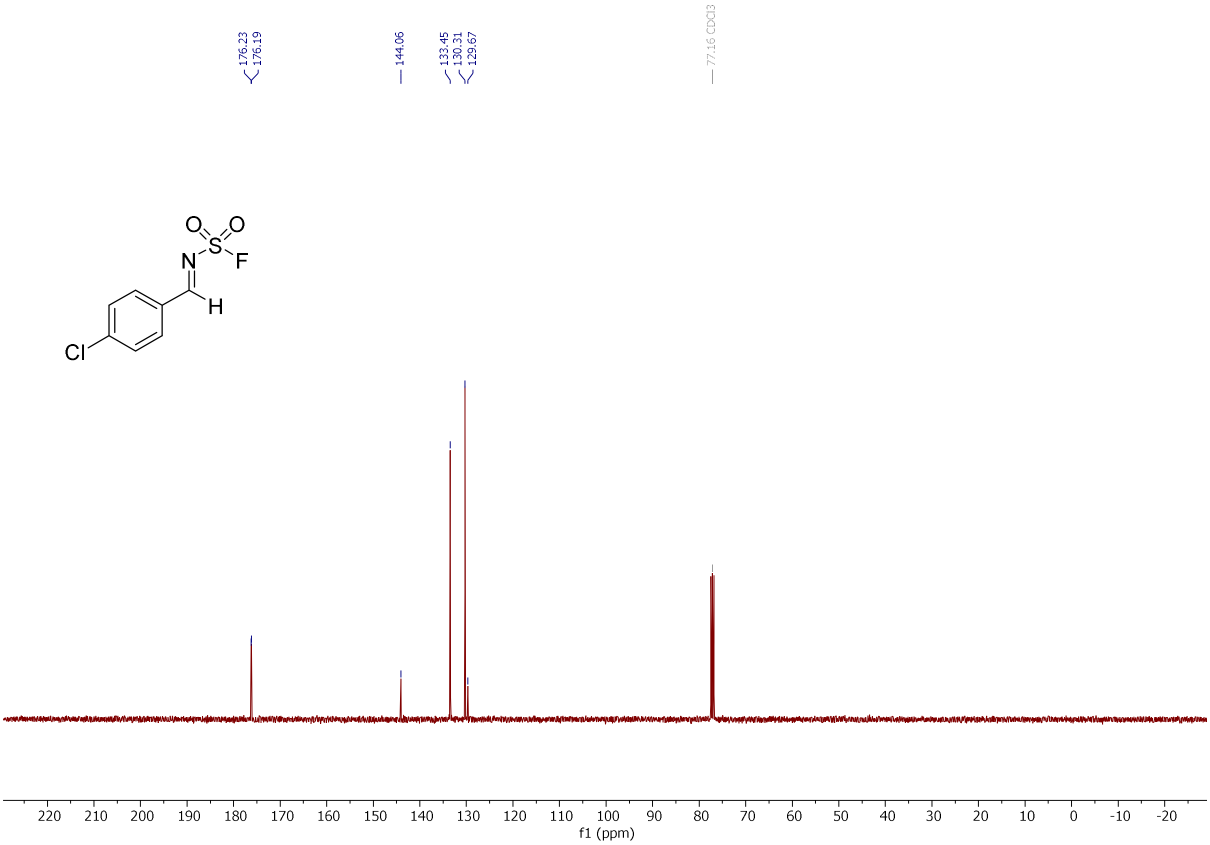
176.23
176.19
144.06
133.45
130.31
129.67
77.16 CDCl3
Cl
N
S
O
O
F
H
f1 (ppm)

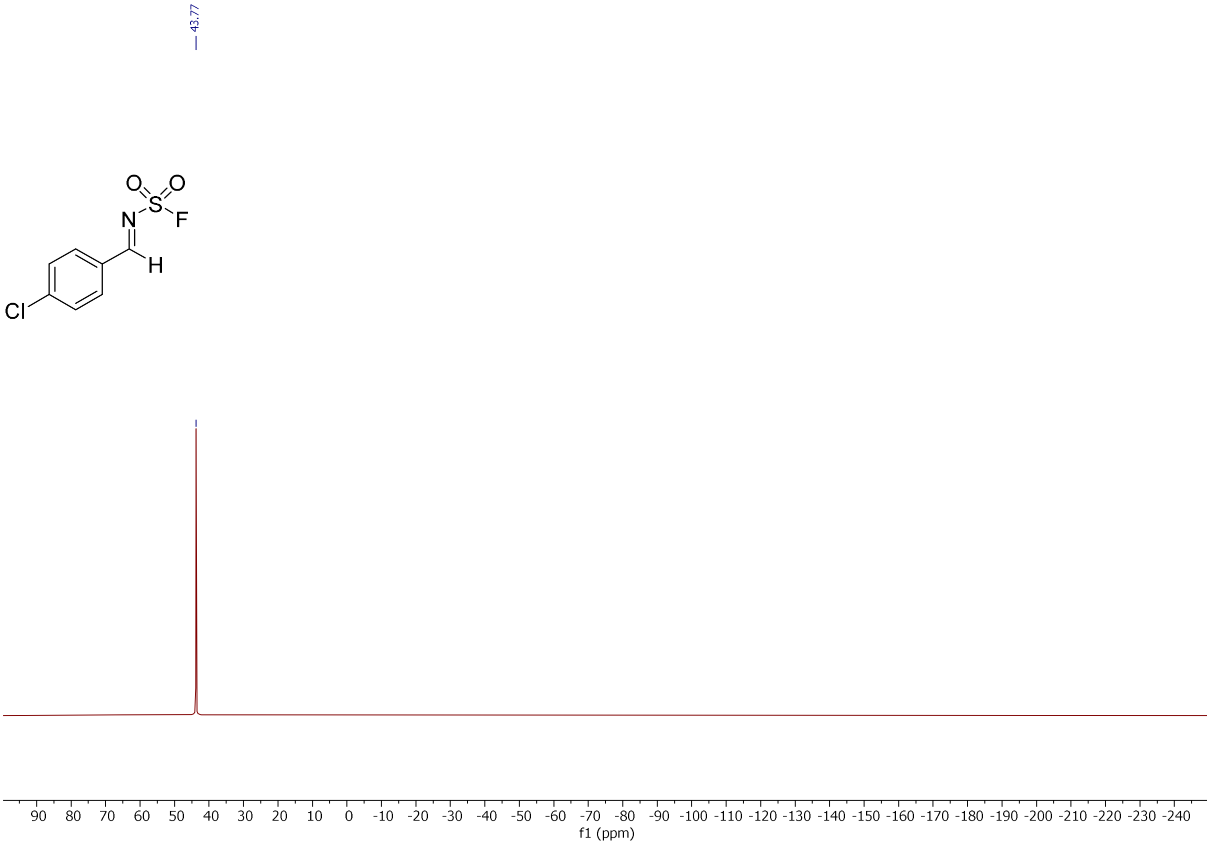
43.77
Cl
N
S
O
O
F
H
f1 (ppm)

(4-Bromobenzylidene)sulfamoyl fluoride (I-9)

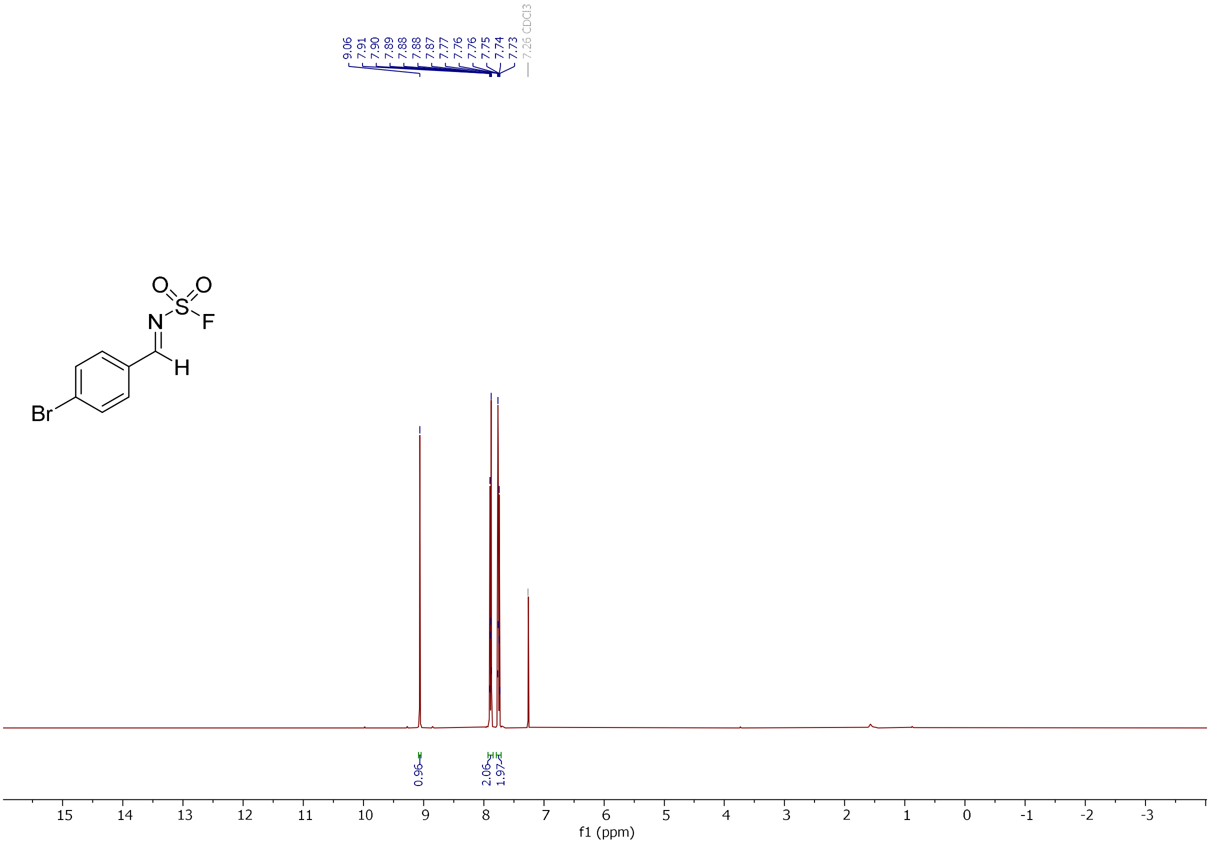

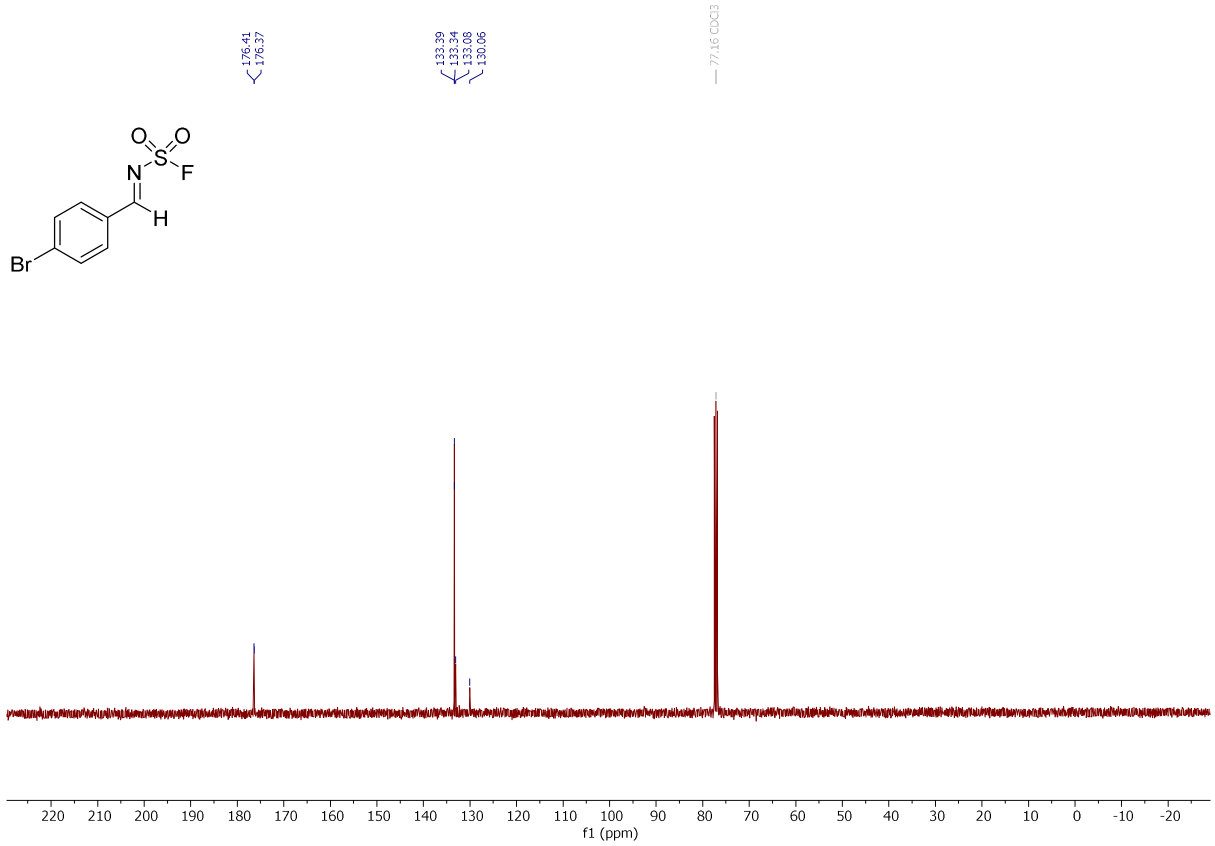
176.41
176.37
133.39
133.34
133.08
130.06
77.16 CDCl3
Br
N
S
F
O
O
H
220 210 200 190 180 170 160 150 140 130 120 110 100 90 80 70 60 50 40 30 20 10 0 -10 -20
f1 (ppm)

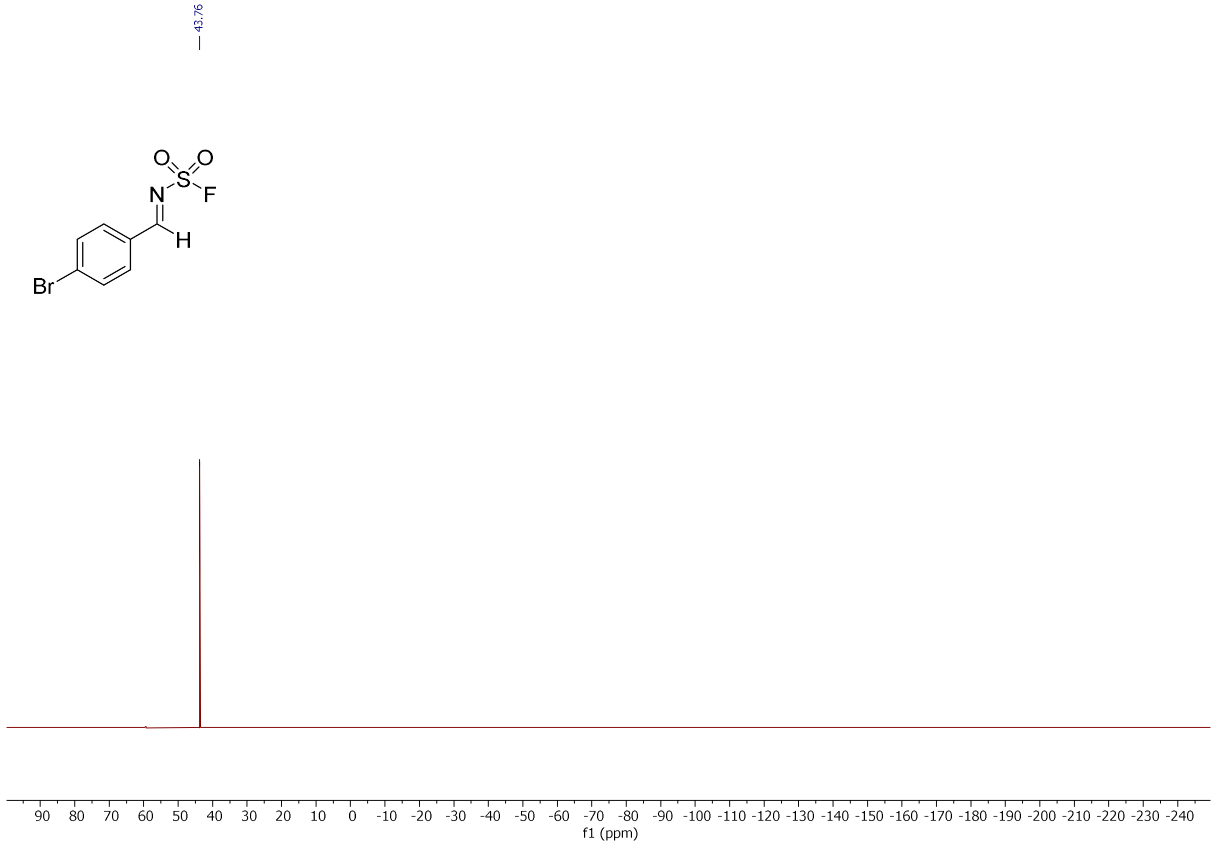
43.76
Br
N
S
F
O
O
H
90 80 70 60 50 40 30 20 10 0 -10 -20 -30 -40 -50 -60 -70 -80 -90 -100 -110 -120 -130 -140 -150 -160 -170 -180 -190 -200 -210 -220 -230 -240
f1 (ppm)

([1,1'-Biphenyl]-4-ylmethylene)sulfamoyl fluoride (I-10)

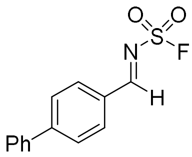

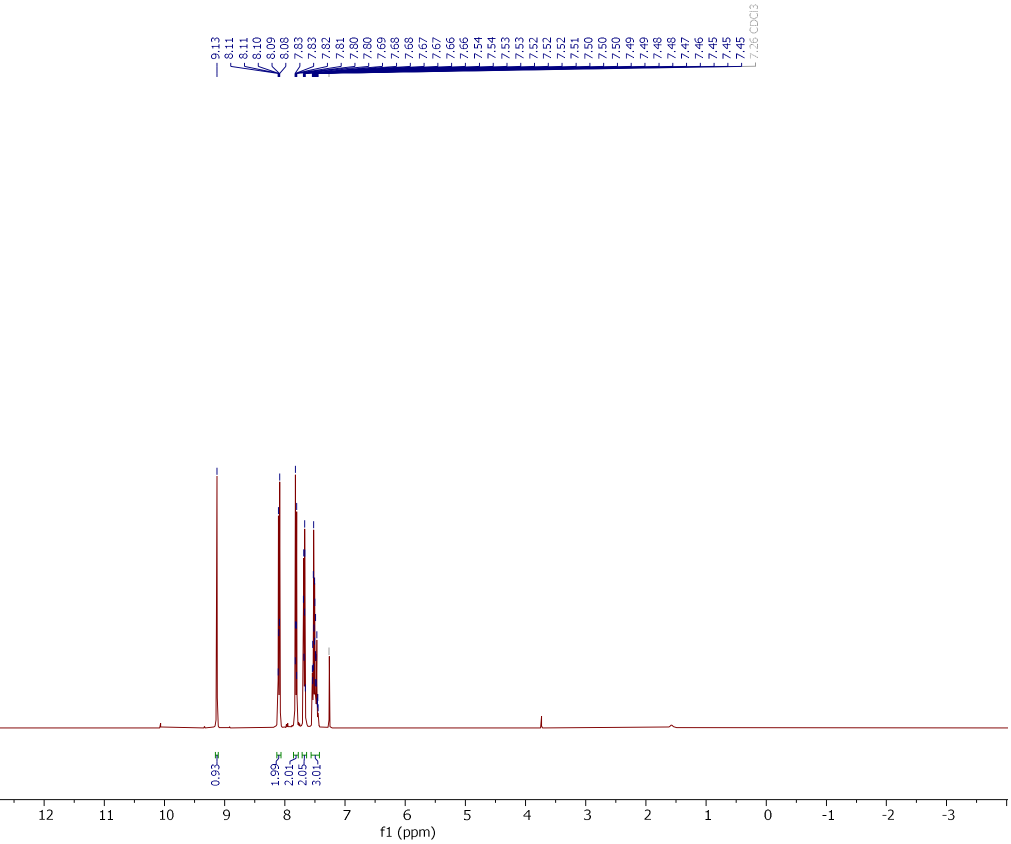

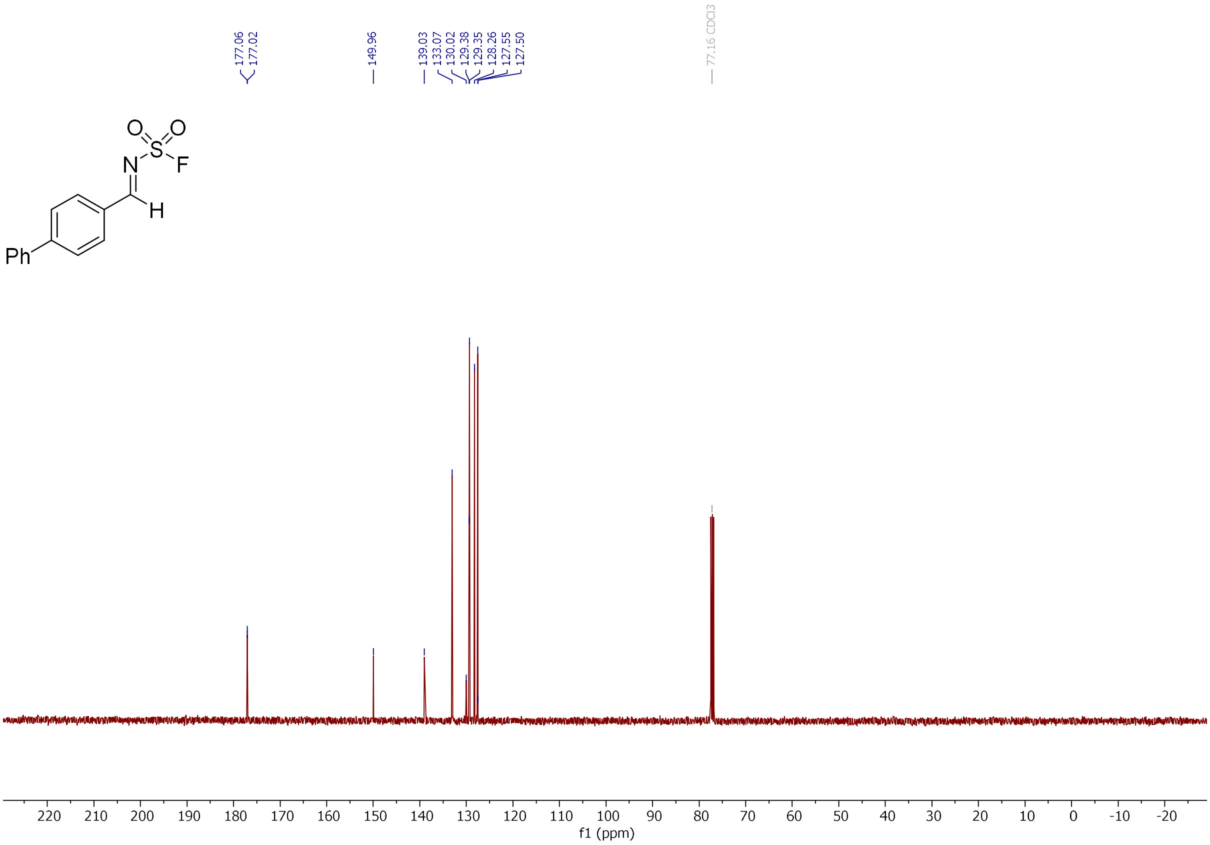
177.06
177.02
149.96
139.03
133.07
130.02
129.38
129.35
128.26
127.55
127.50
77.16 CDCl3
O
O
S
N
F
H
Ph
220 210 200 190 180 170 160 150 140 130 120 110 100 90 80 70 60 50 40 30 20 10 0 -10 -20
f1 (ppm)

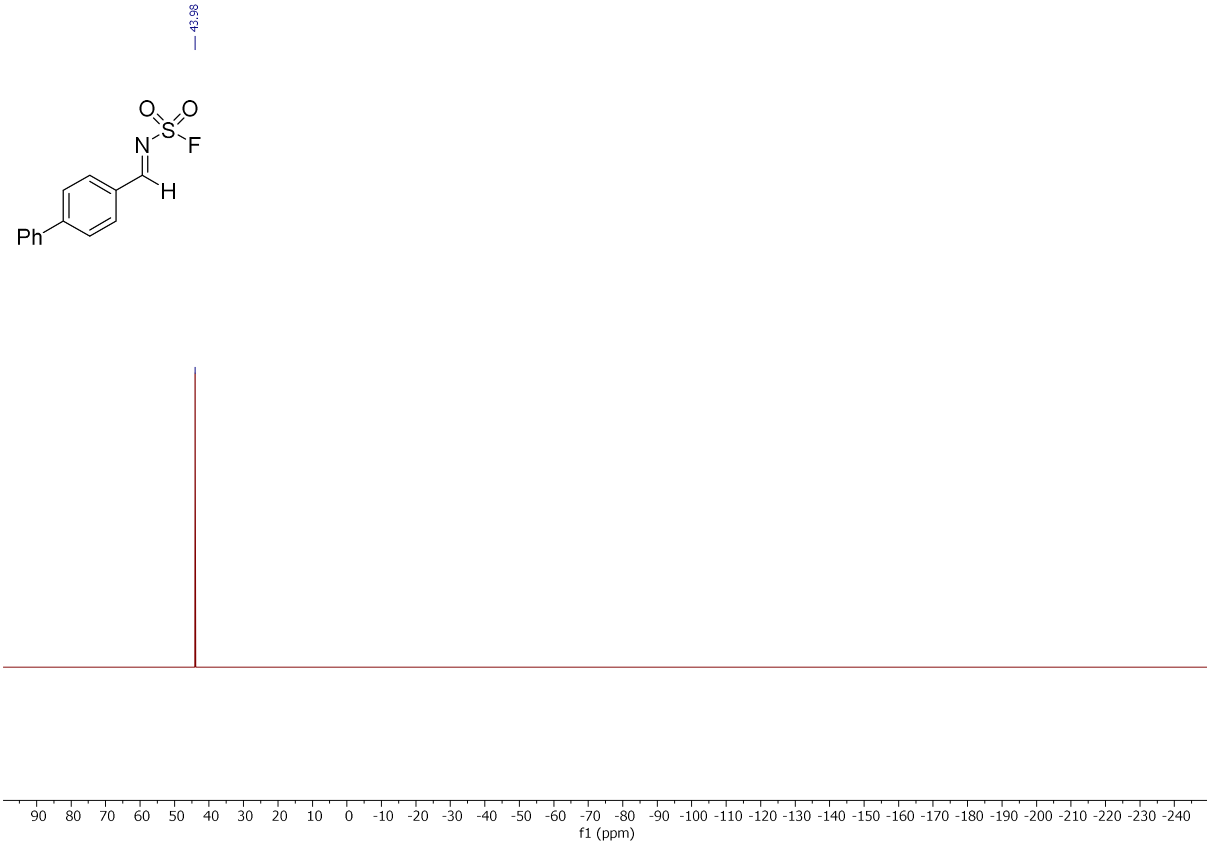
43.98
O
O
S
N
F
H
Ph
90 80 70 60 50 40 30 20 10 0 -10 -20 -30 -40 -50 -60 -70 -80 -90 -100 -110 -120 -130 -140 -150 -160 -170 -180 -190 -200 -210 -220 -230 -240
f1 (ppm)

(2-Methylbenzylidene)sulfamoyl fluoride (I-11)

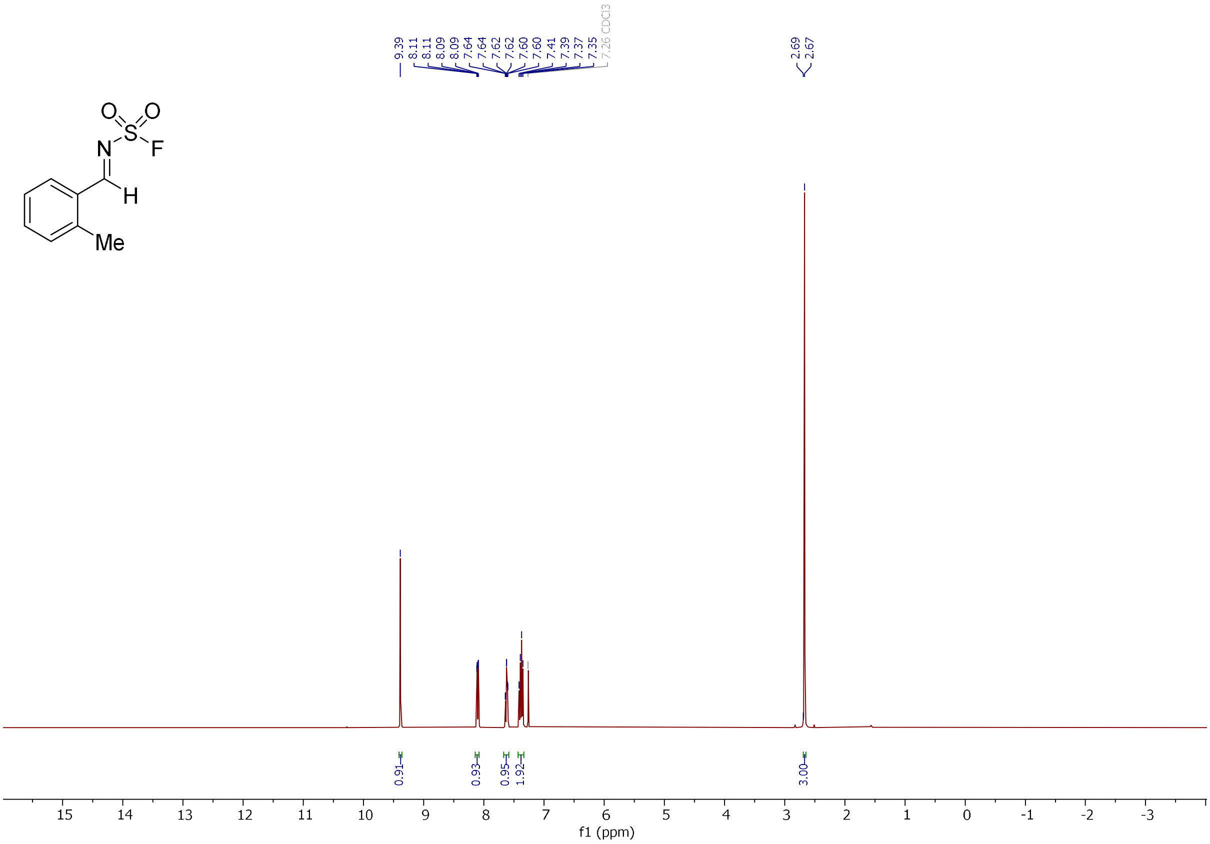

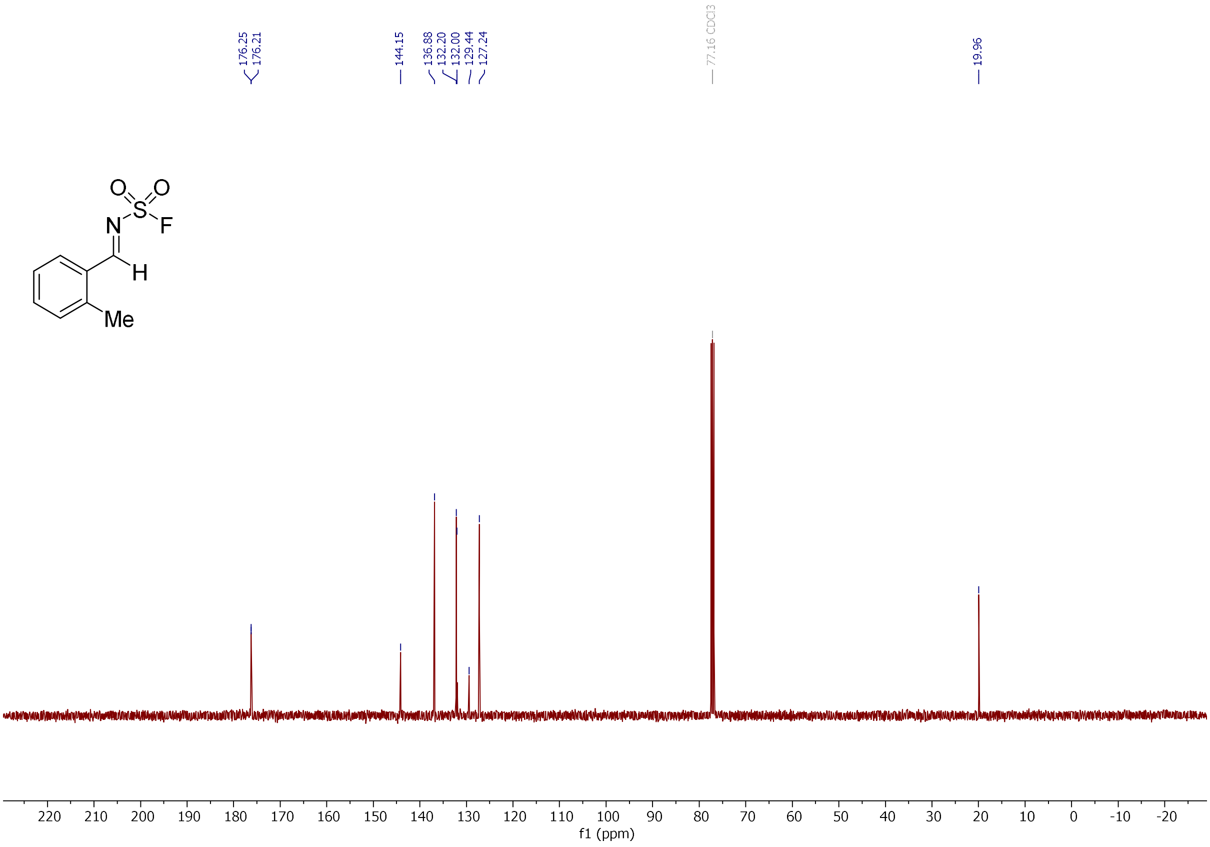

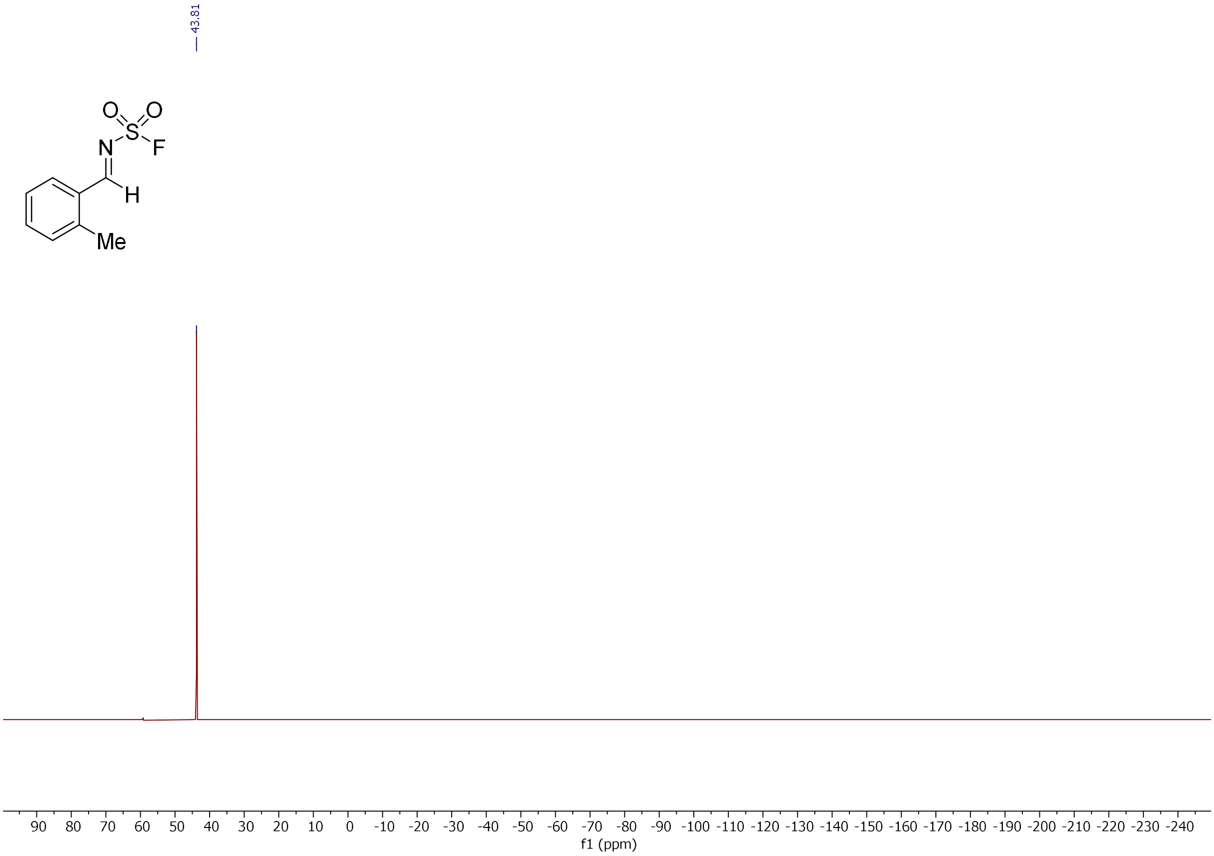

(4-(1,3-Dioxoisoindolin-2-yl)benzylidene)sulfamoyl fluoride (I-12)

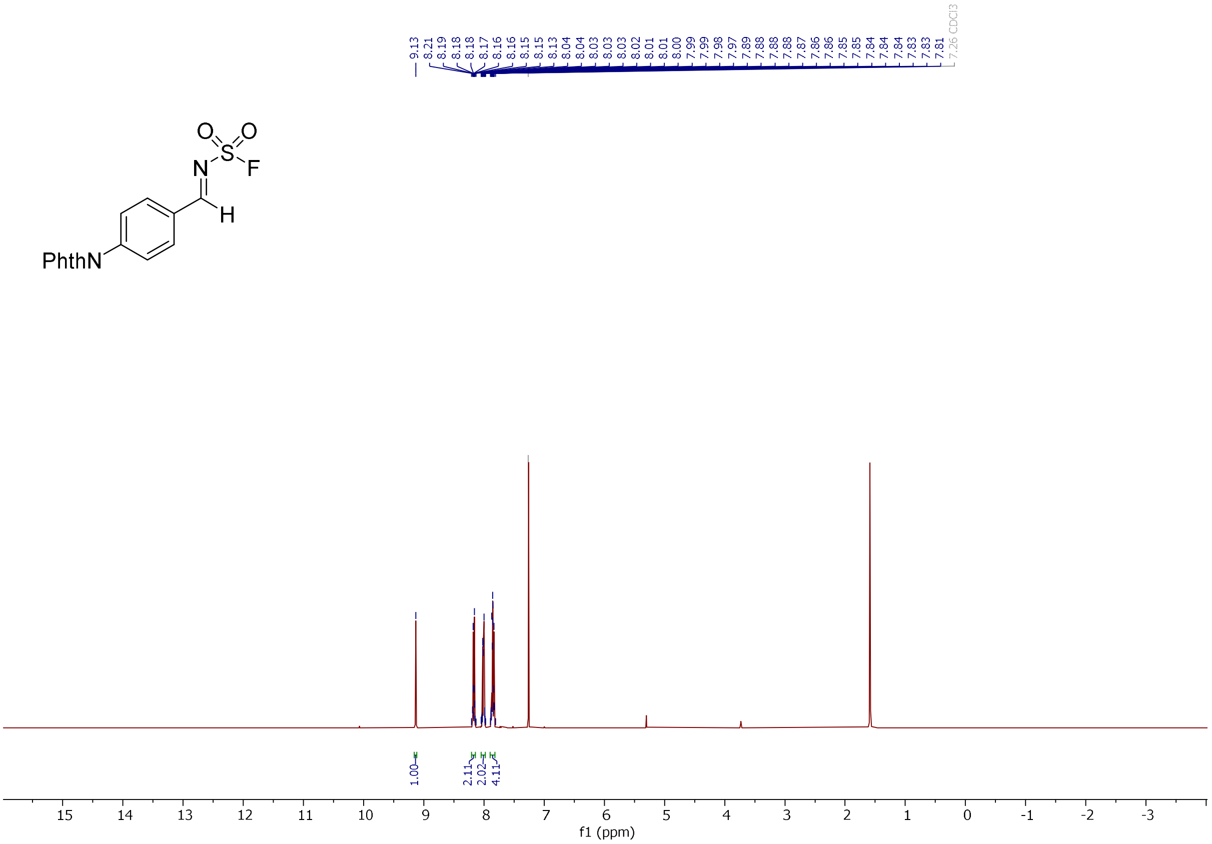

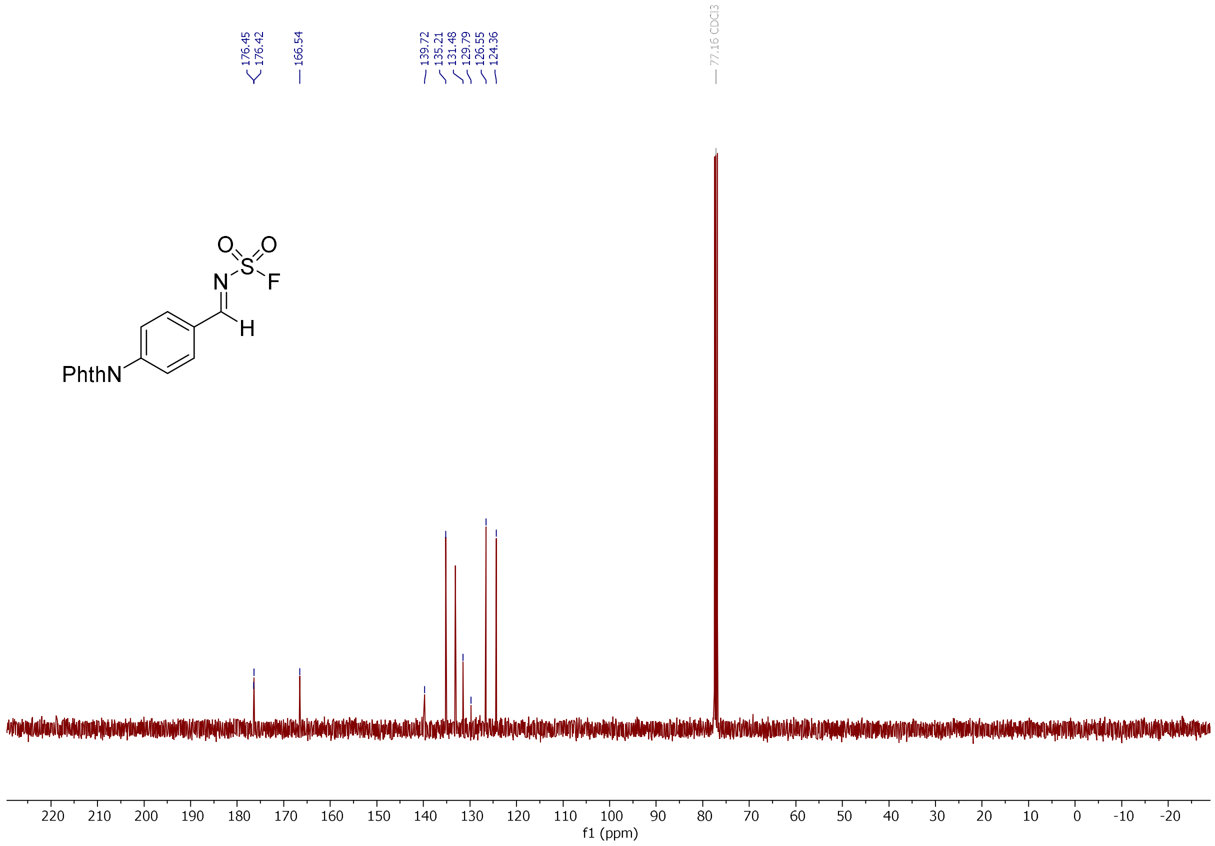
176.45
176.42
166.54
139.72
135.21
131.48
129.79
126.55
124.36
77.16 CDCl3
O
S
O
N
F
H
PhthN
220 210 200 190 180 170 160 150 140 130 120 110 100 90 80 70 60 50 40 30 20 10 0 -10 -20
f1 (ppm)

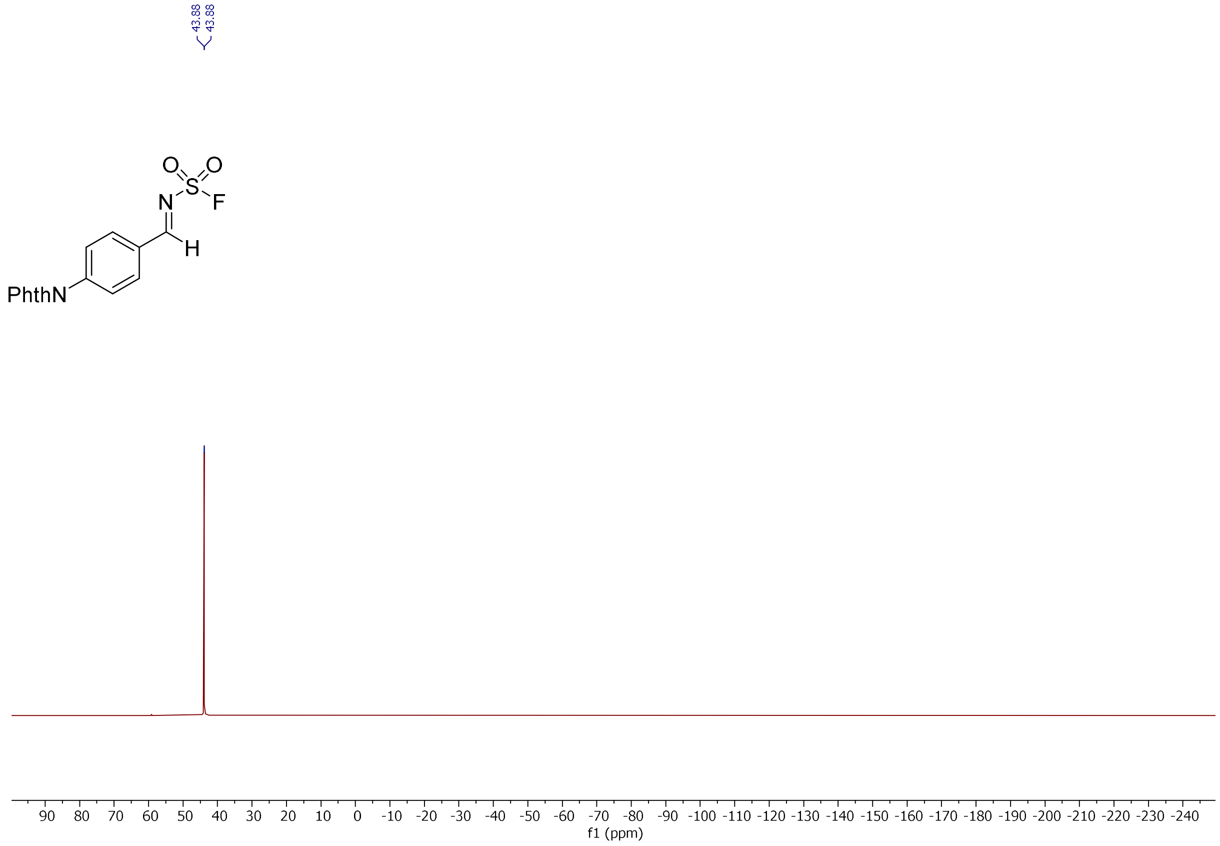
43.88
43.88
O
S
O
N
F
H
PhthN
90 80 70 60 50 40 30 20 10 0 -10 -20 -30 -40 -50 -60 -70 -80 -90 -100 -110 -120 -130 -140 -150 -160 -170 -180 -190 -200 -210 -220 -230 -240
f1 (ppm)

4-(((Fluorosulfonyl)imino)methyl)phenyl pivalate (I-13)

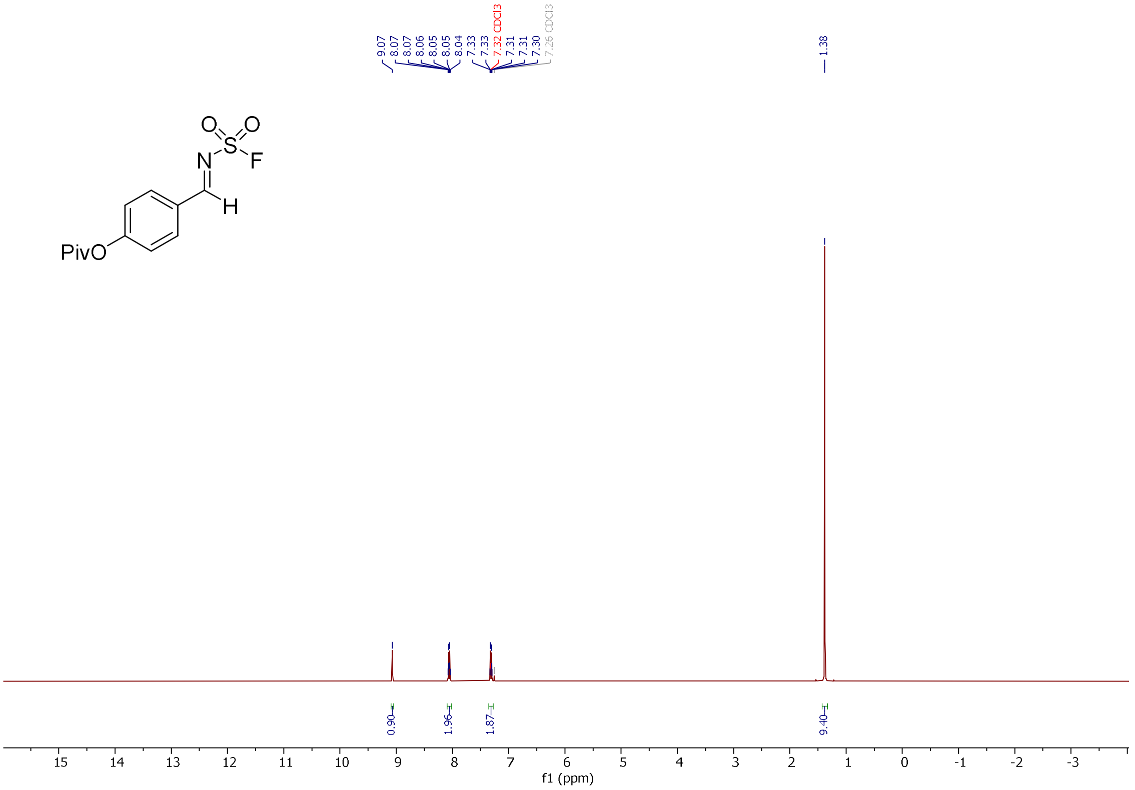

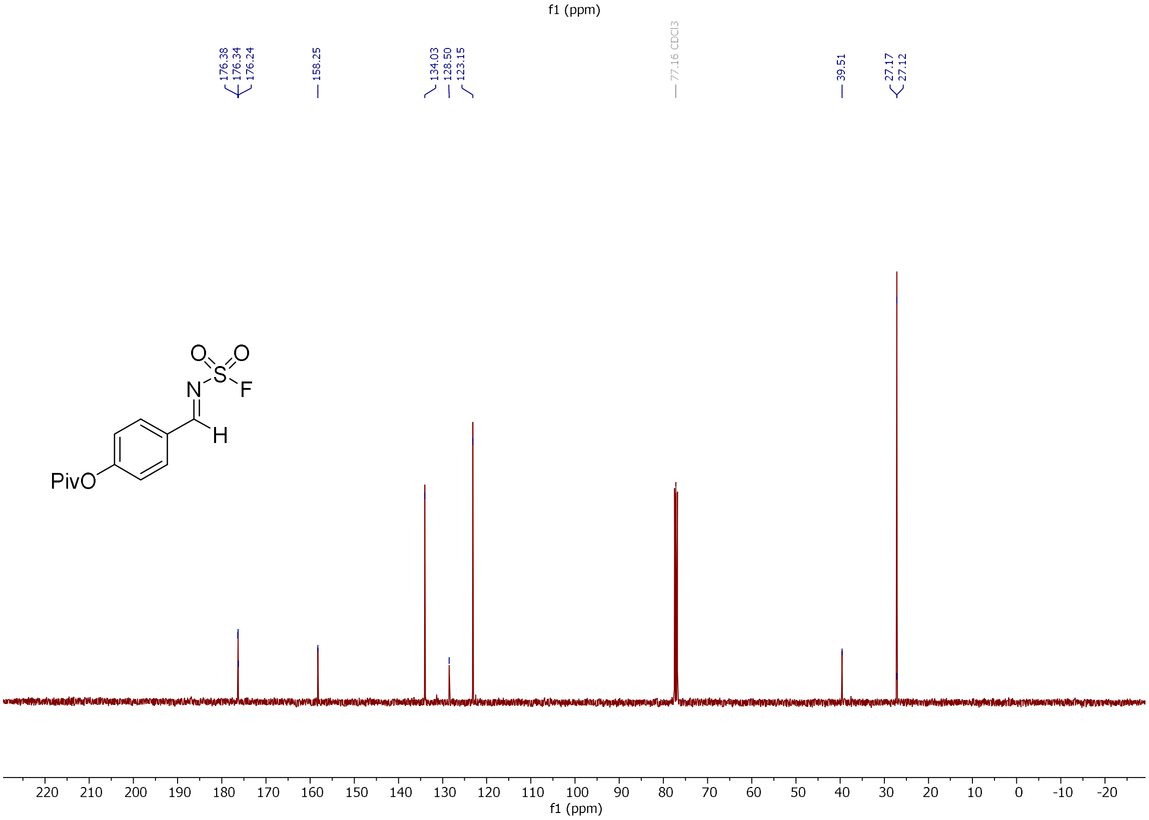

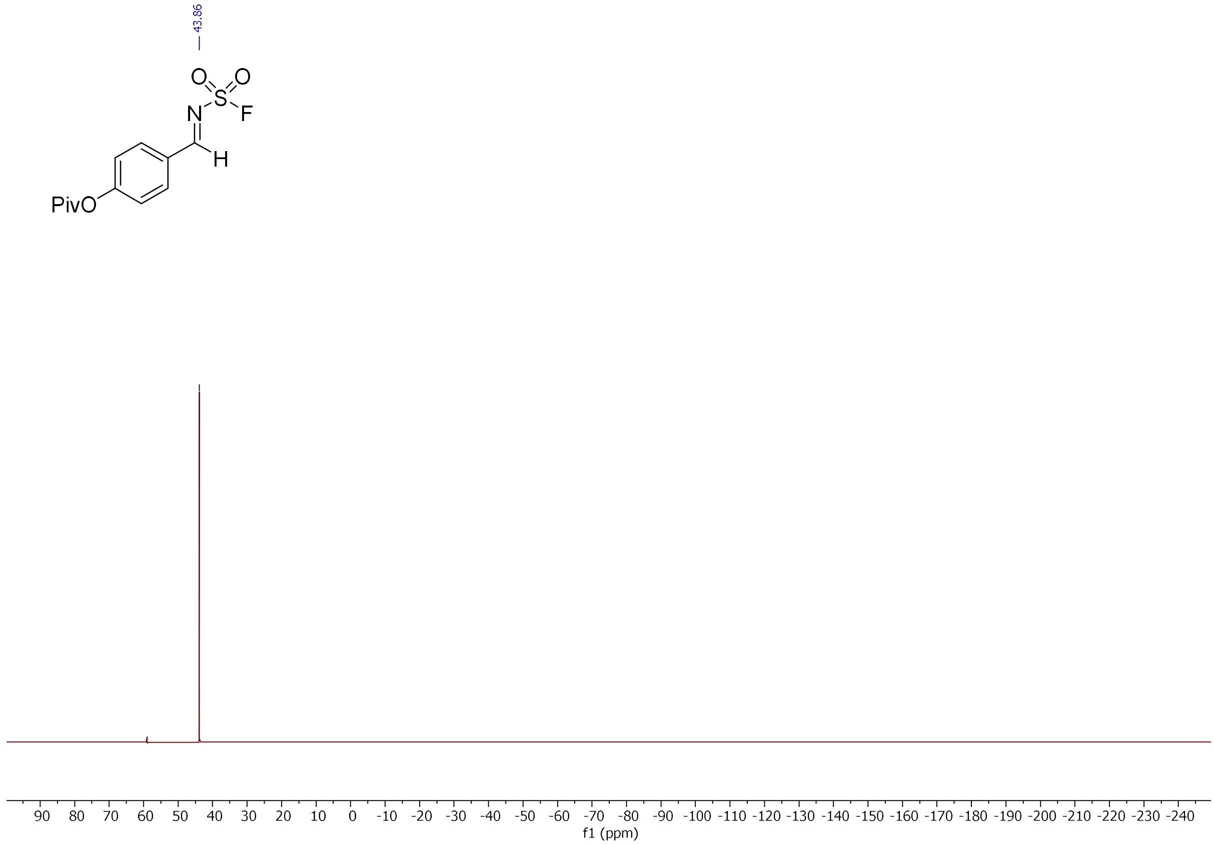

Methyl 3-(((fluorosulfonyl)imino)methyl)benzoate (I-14)

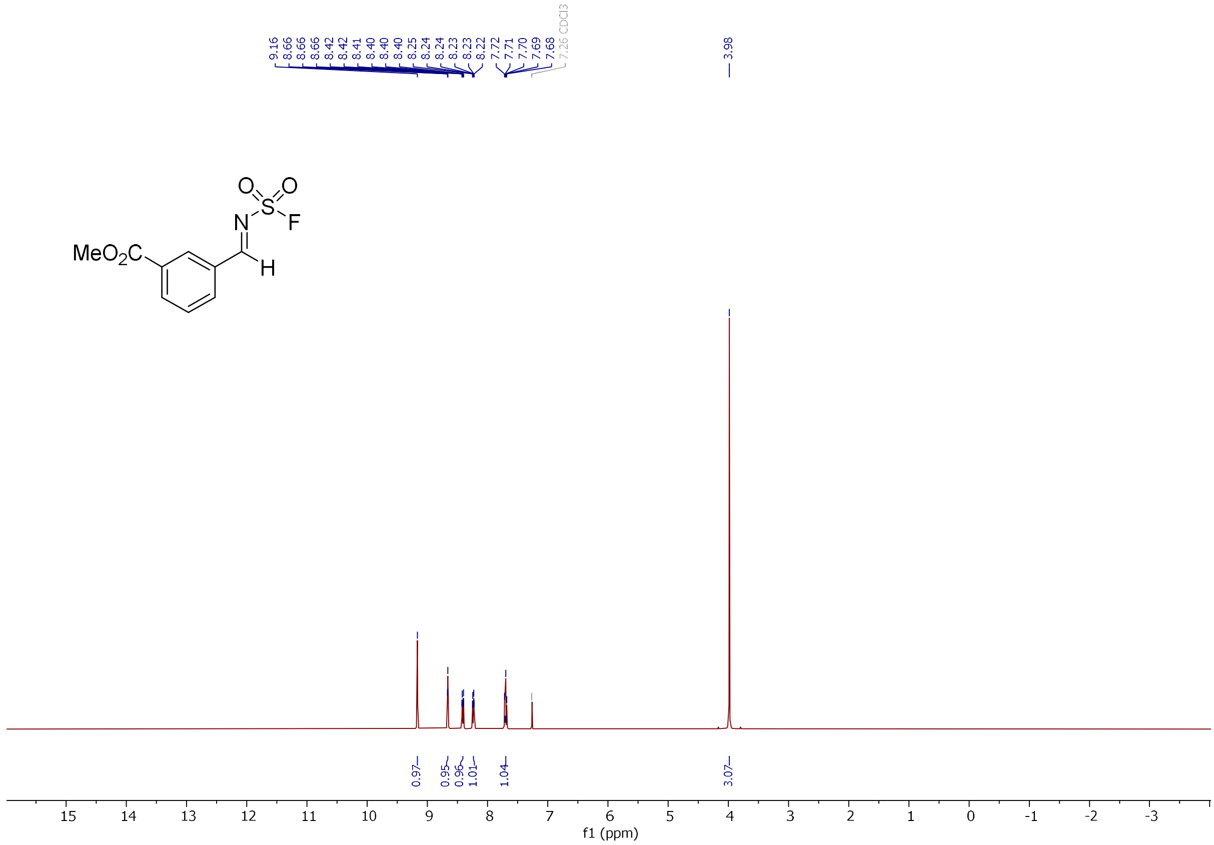

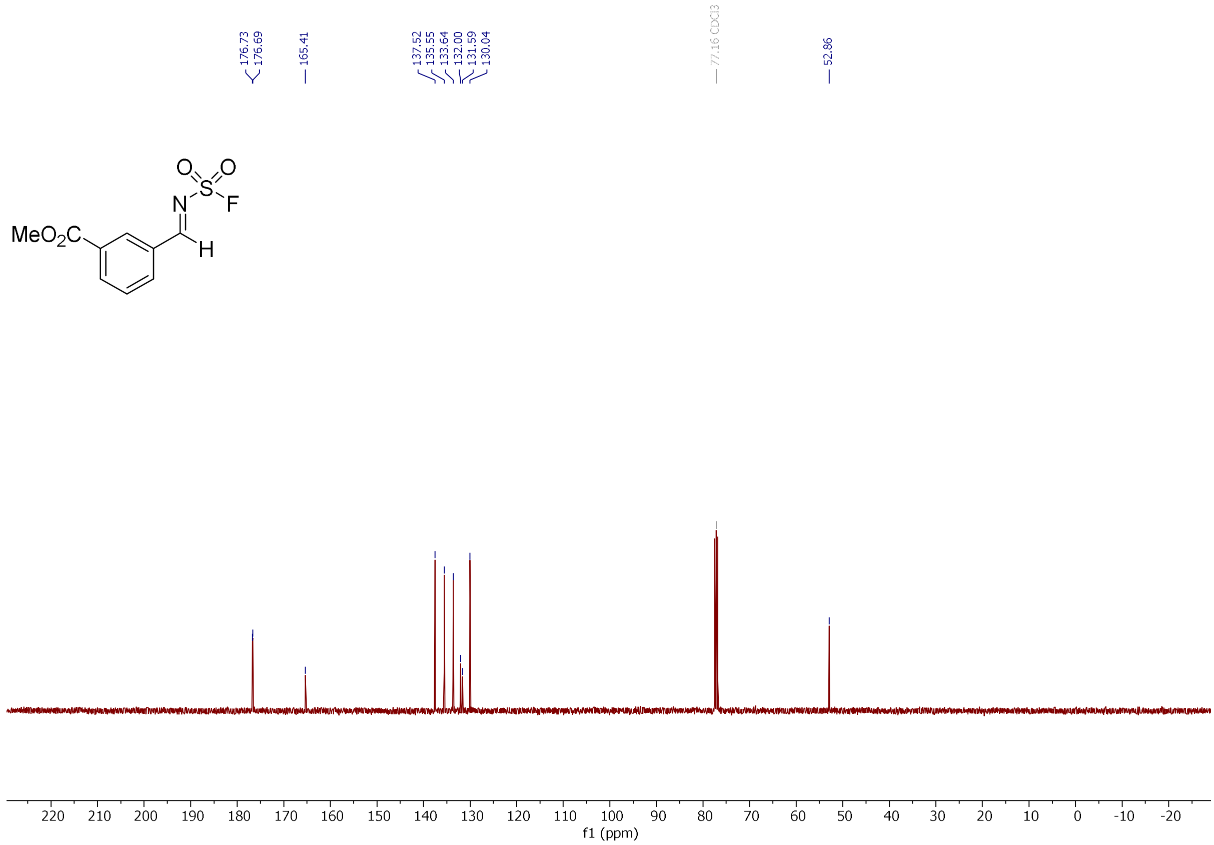
176.73
176.69
165.41
137.52
135.55
133.64
132.00
131.59
130.04
77.16 CDCl3
52.86
MeO2C
N
S
O
O
F
H
220 210 200 190 180 170 160 150 140 130 120 110 100 90 80 70 60 50 40 30 20 10 0 -10 -20
f1 (ppm)

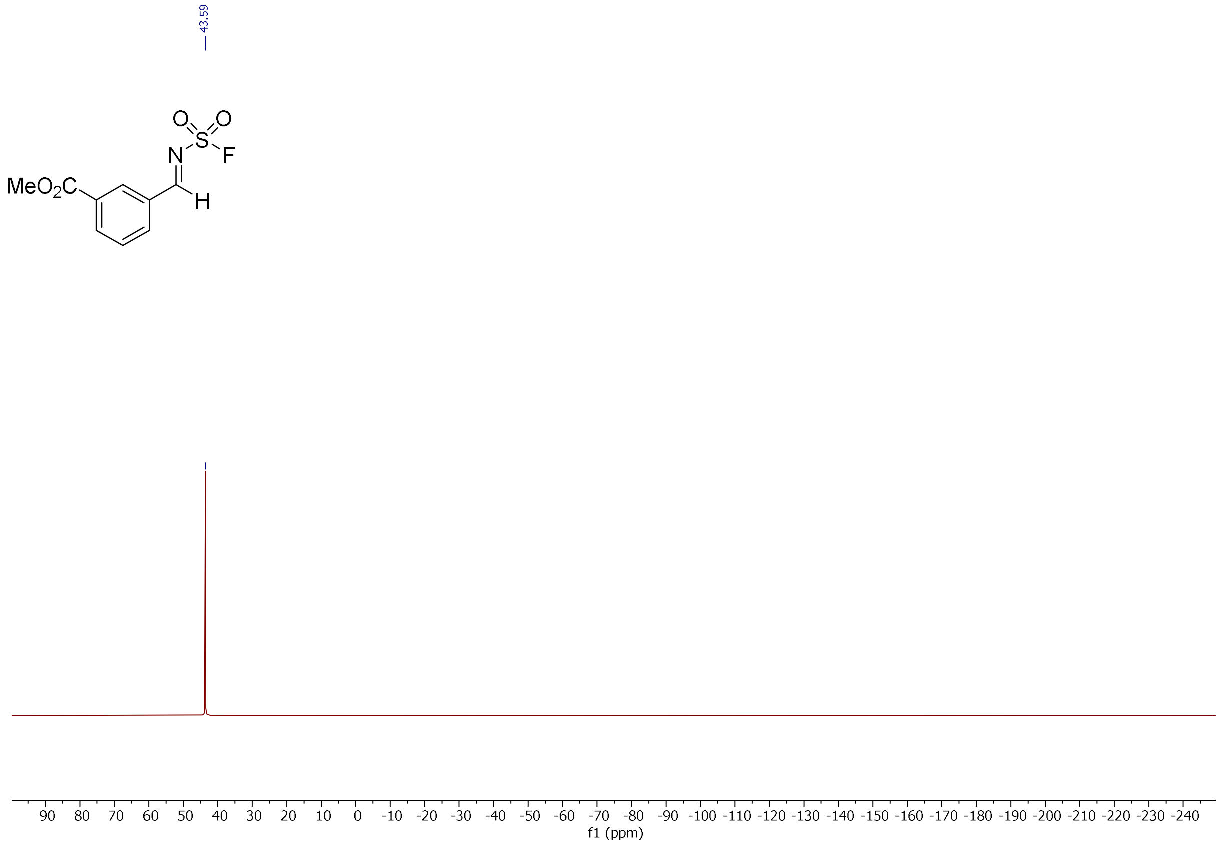
43.59
MeO2C
N
S
O
O
F
H
90 80 70 60 50 40 30 20 10 0 -10 -20 -30 -40 -50 -60 -70 -80 -90 -100 -110 -120 -130 -140 -150 -160 -170 -180 -190 -200 -210 -220 -230 -240
f1 (ppm)

(4-(Trifluoromethyl)benzylidene)sulfamoyl fluoride (I-15)

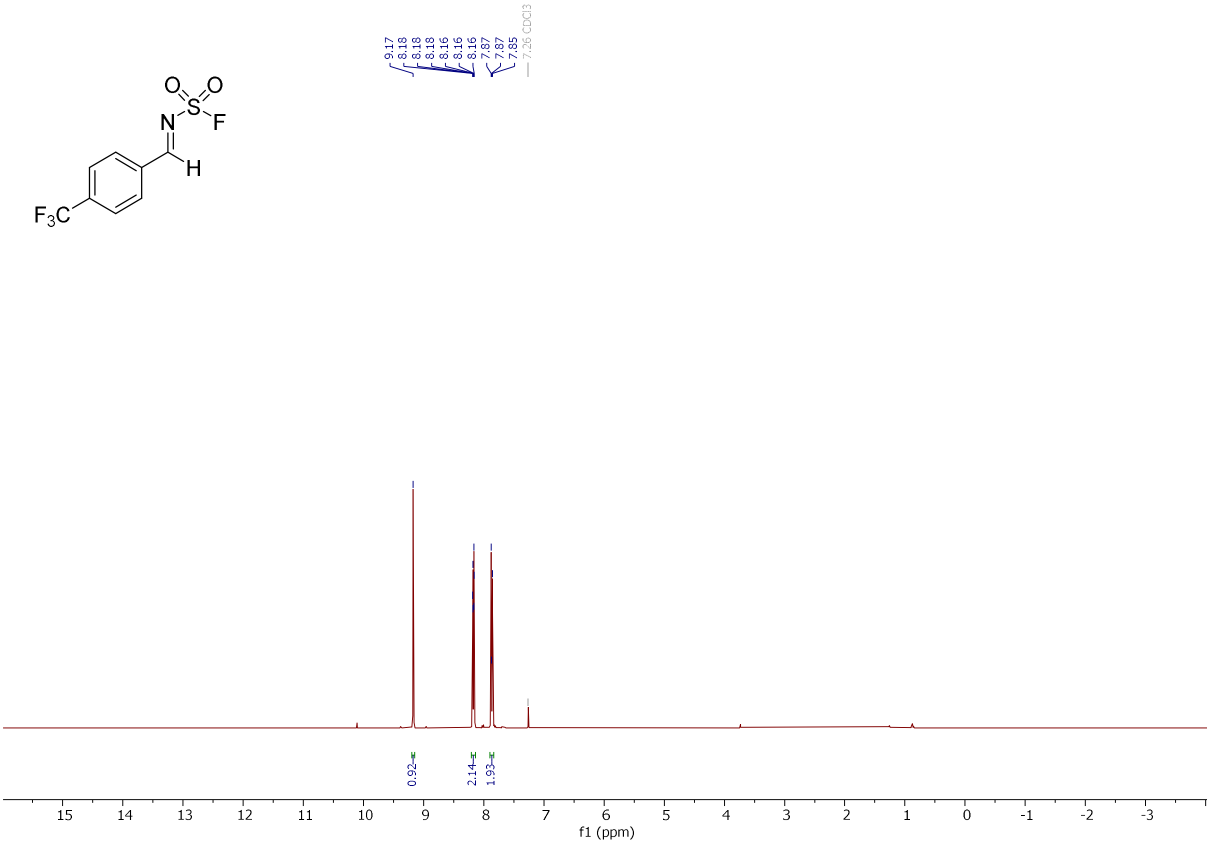

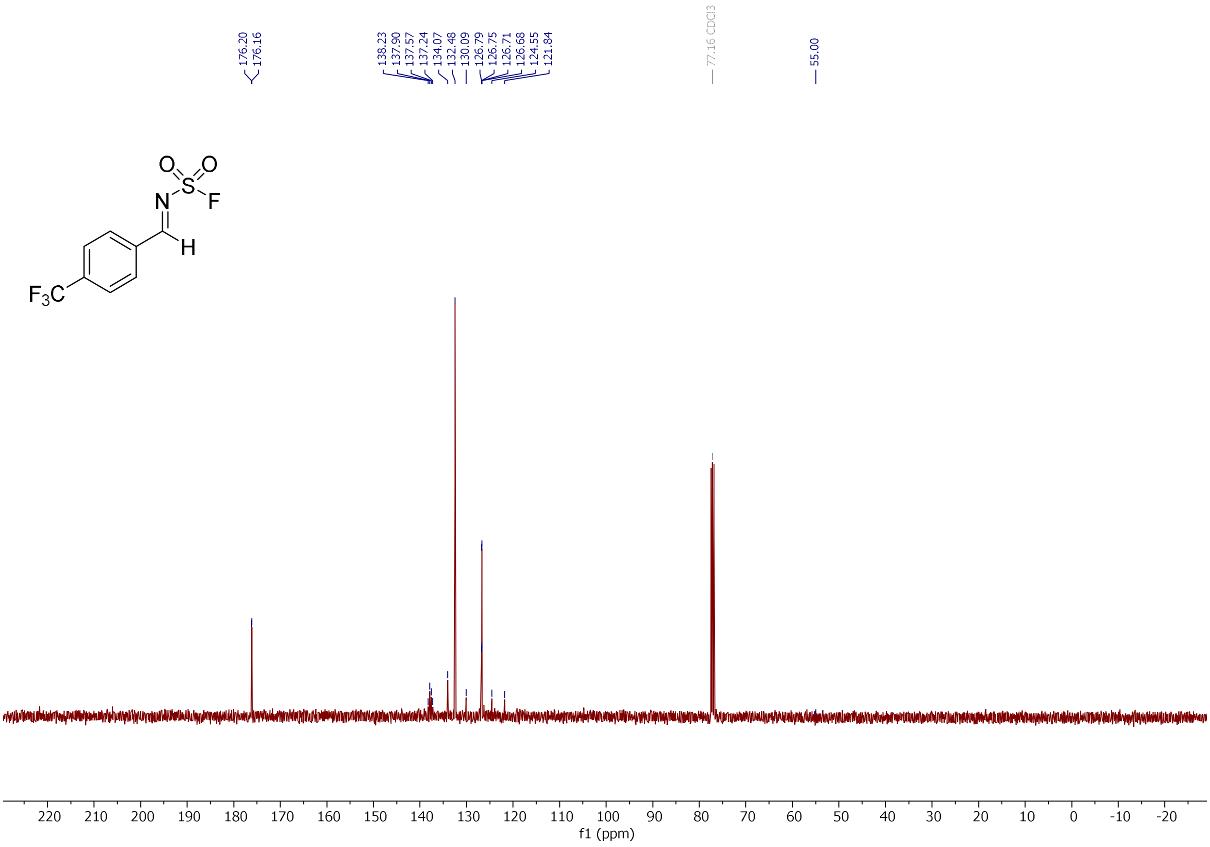

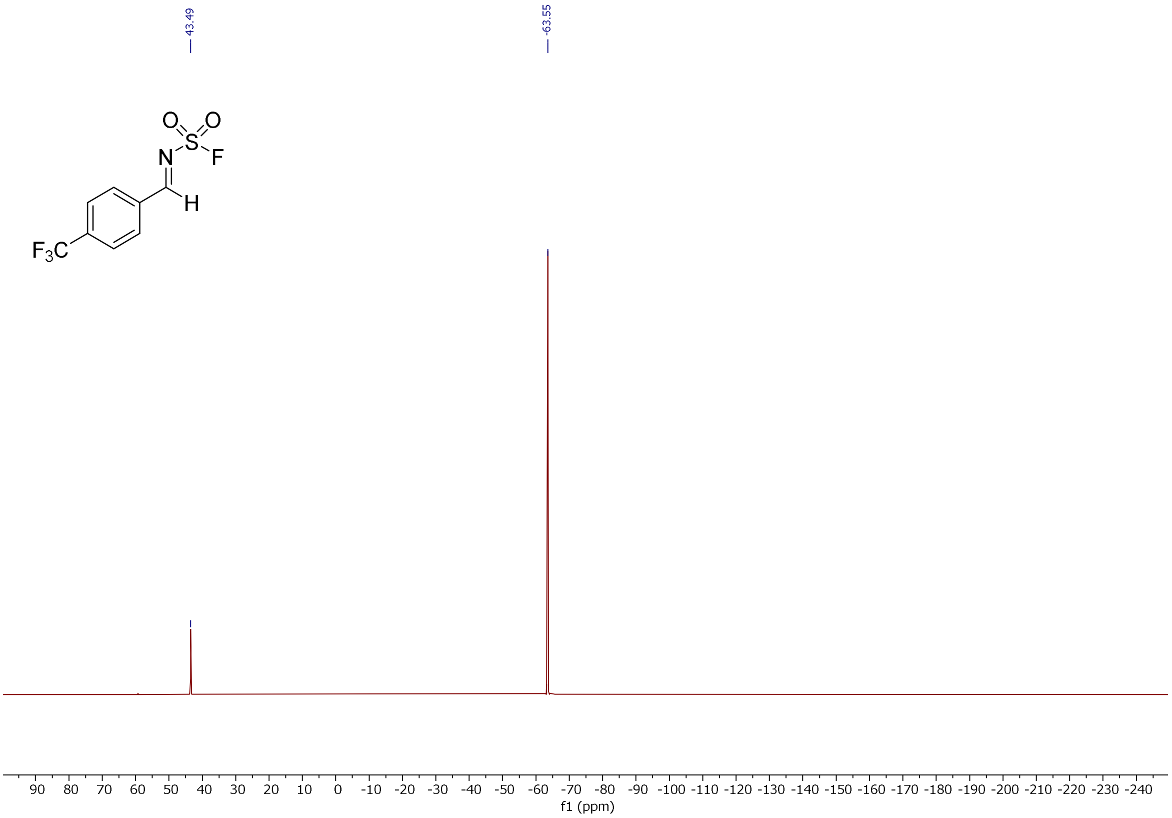

((2,2-Difluorobenzo[d][1,3]dioxol-5-yl)methylene) sulfamoyl fluoride (I-16)

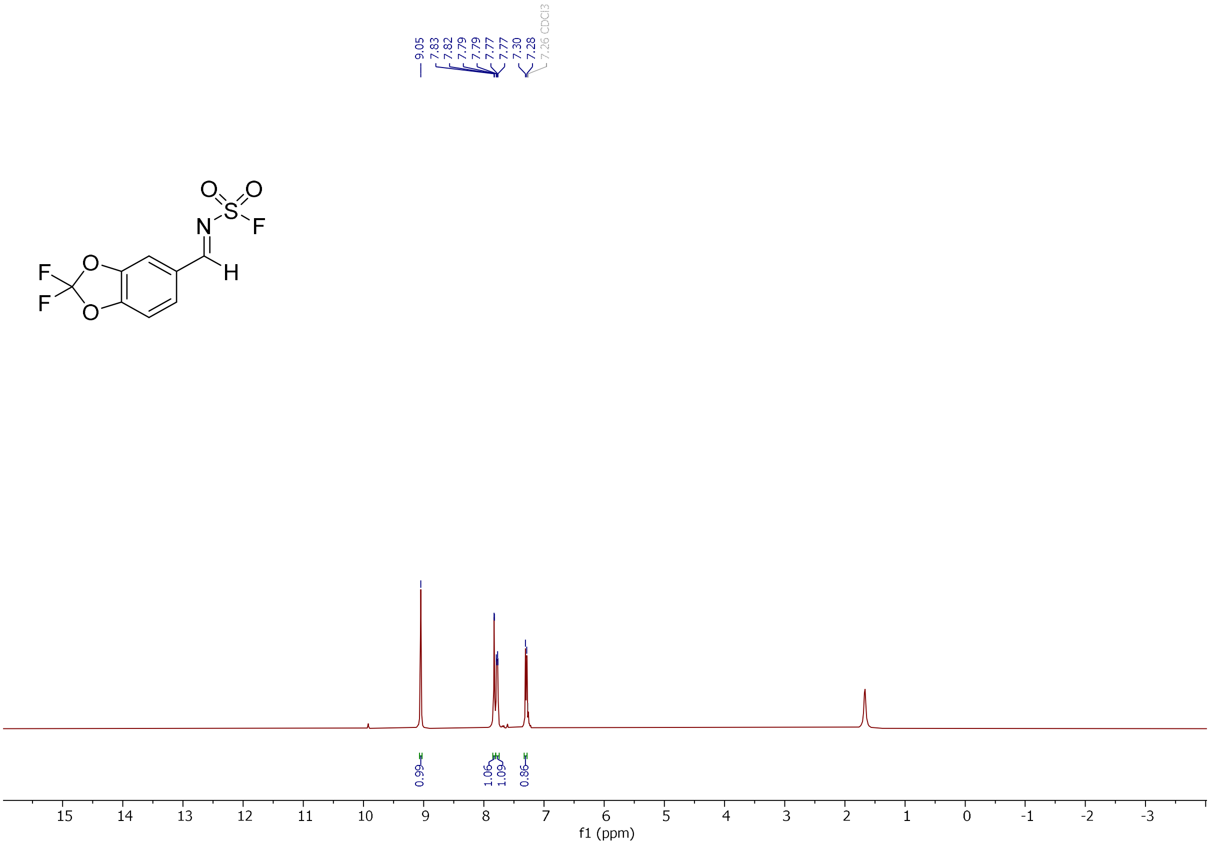

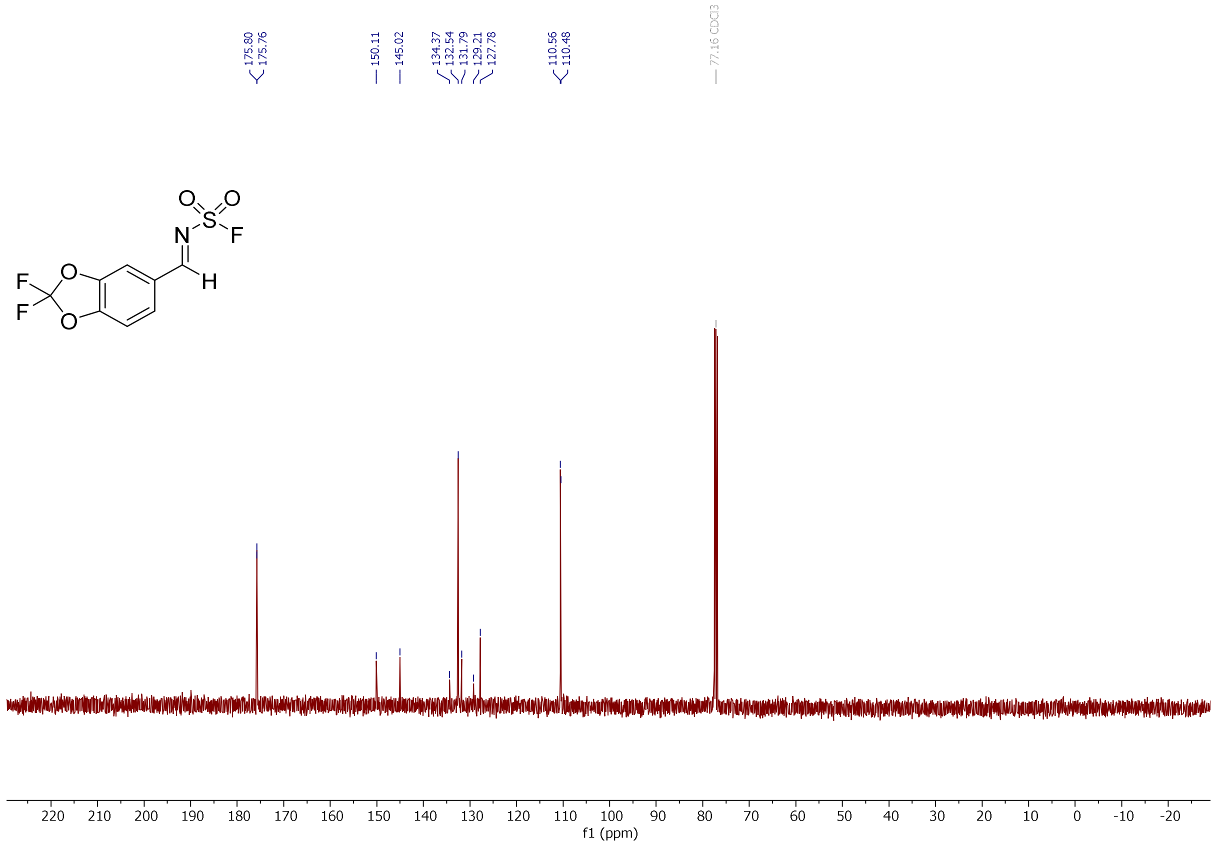
175.80
175.76
150.11
145.02
134.37
132.54
131.79
129.21
127.78
110.56
110.48
77.16 CDCl3
f1 (ppm)

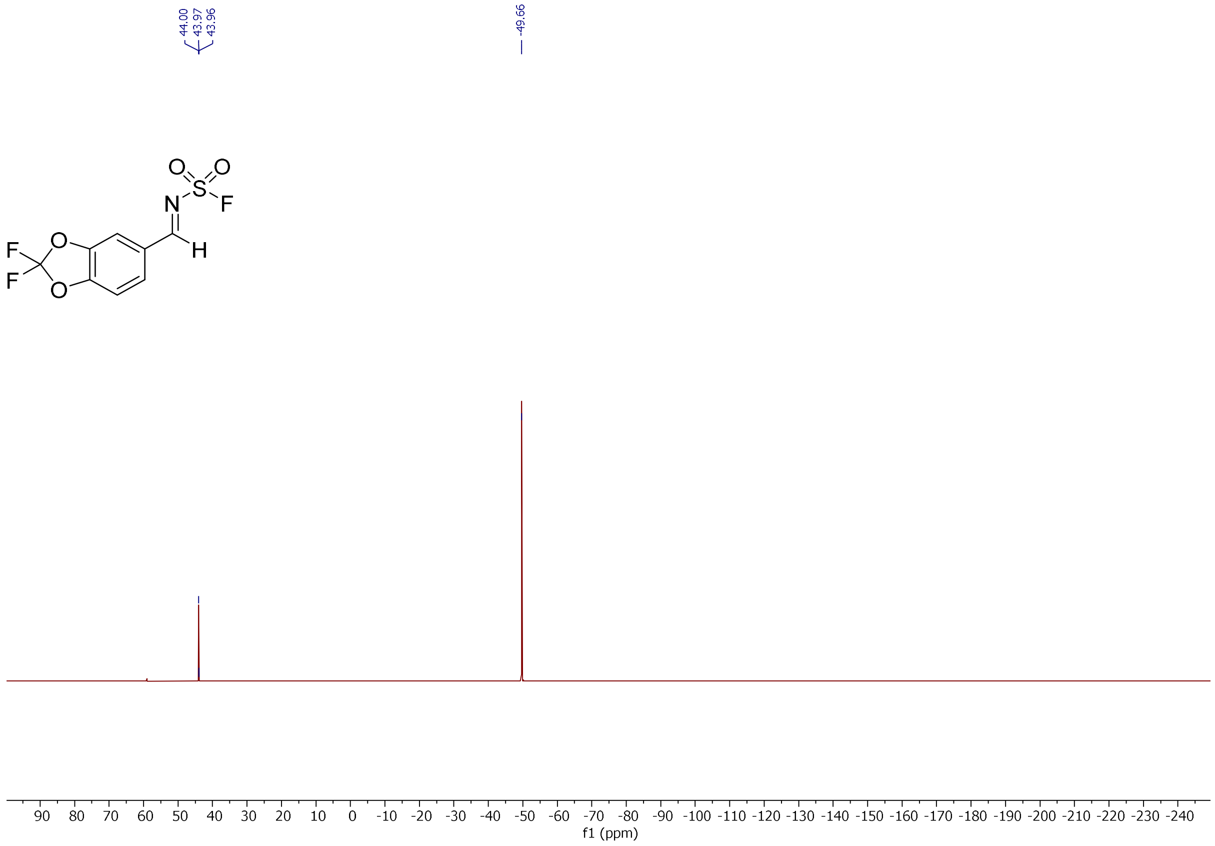
44.00
43.97
43.96
-49.66
f1 (ppm)

(4-(2-Bromoethoxy)benzylidene)sulfamoyl fluoride (I-17)

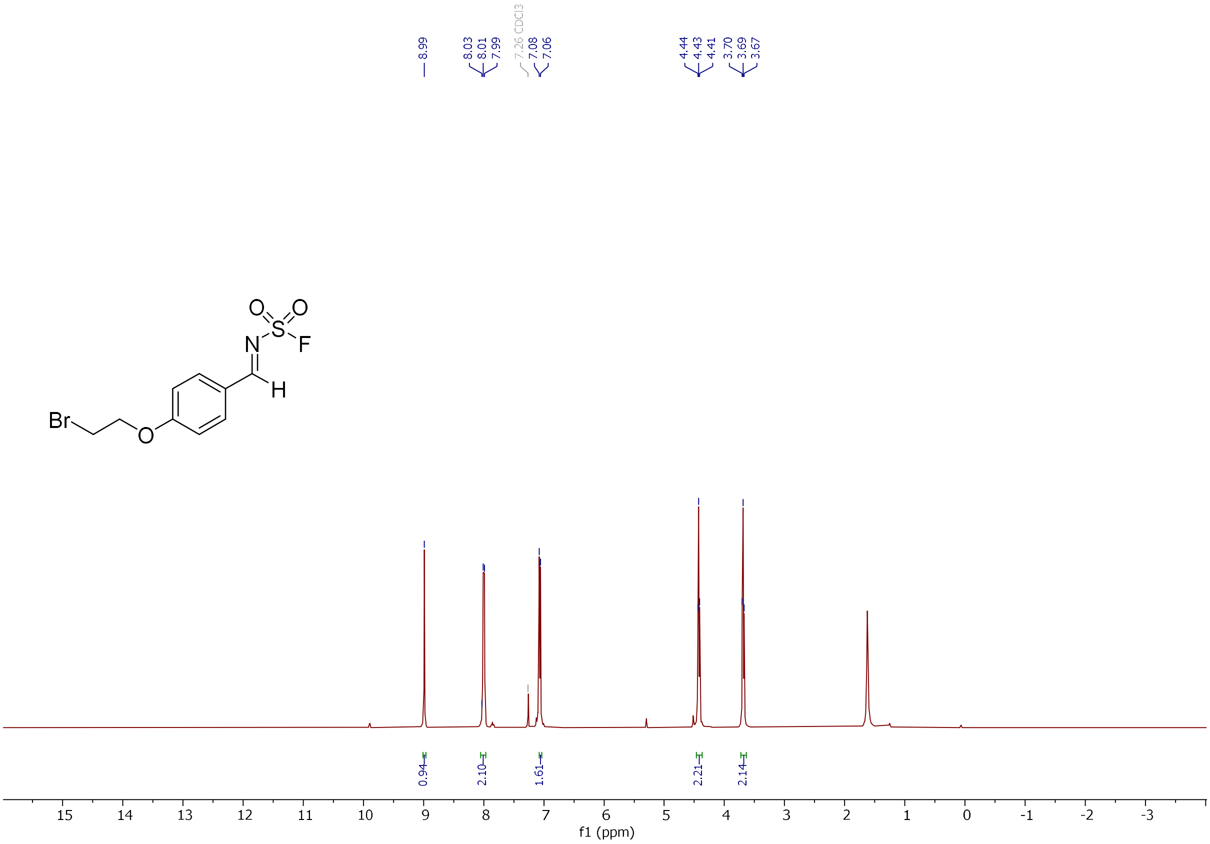

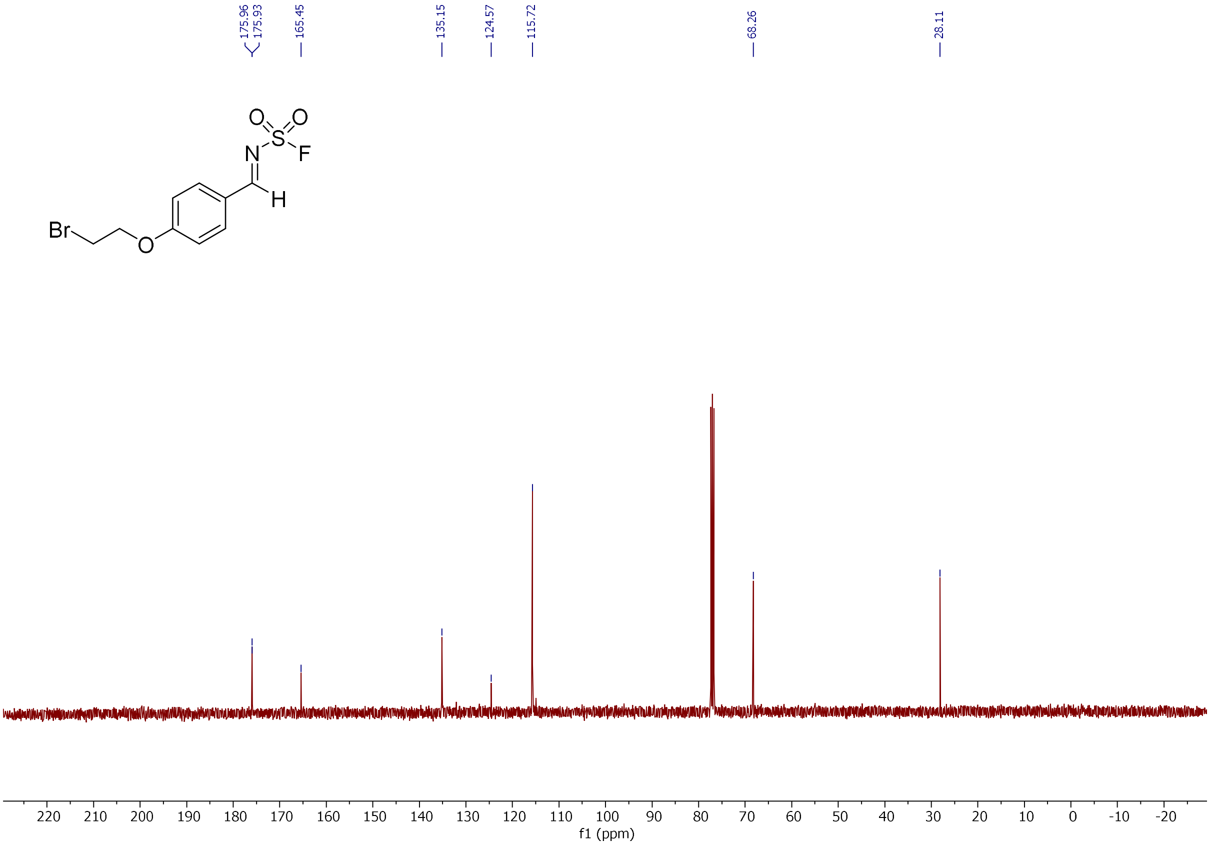

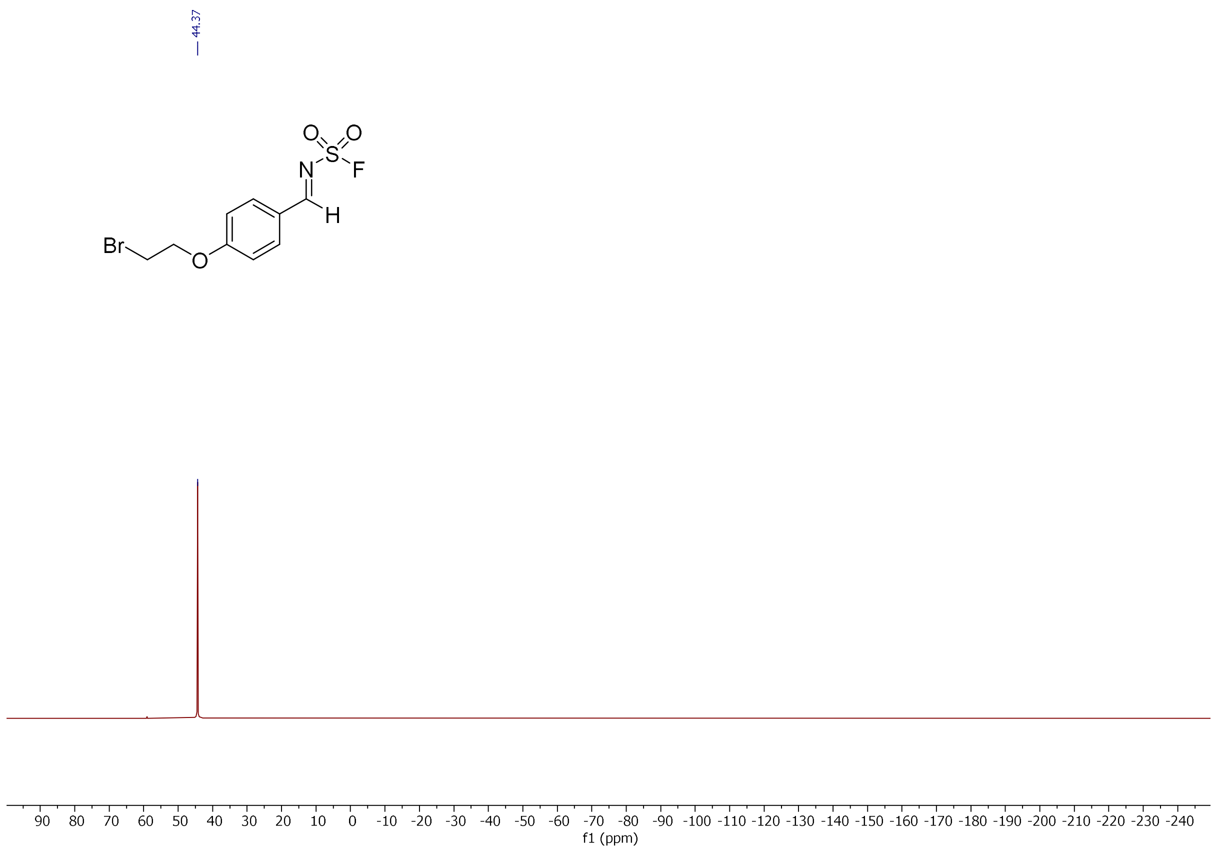

(2-Methoxy-5-(4,4,5,5-tetraethyl-1,3,2-dioxaborolan-2-yl)benzylidene)sulfamoyl fluoride (I-18)

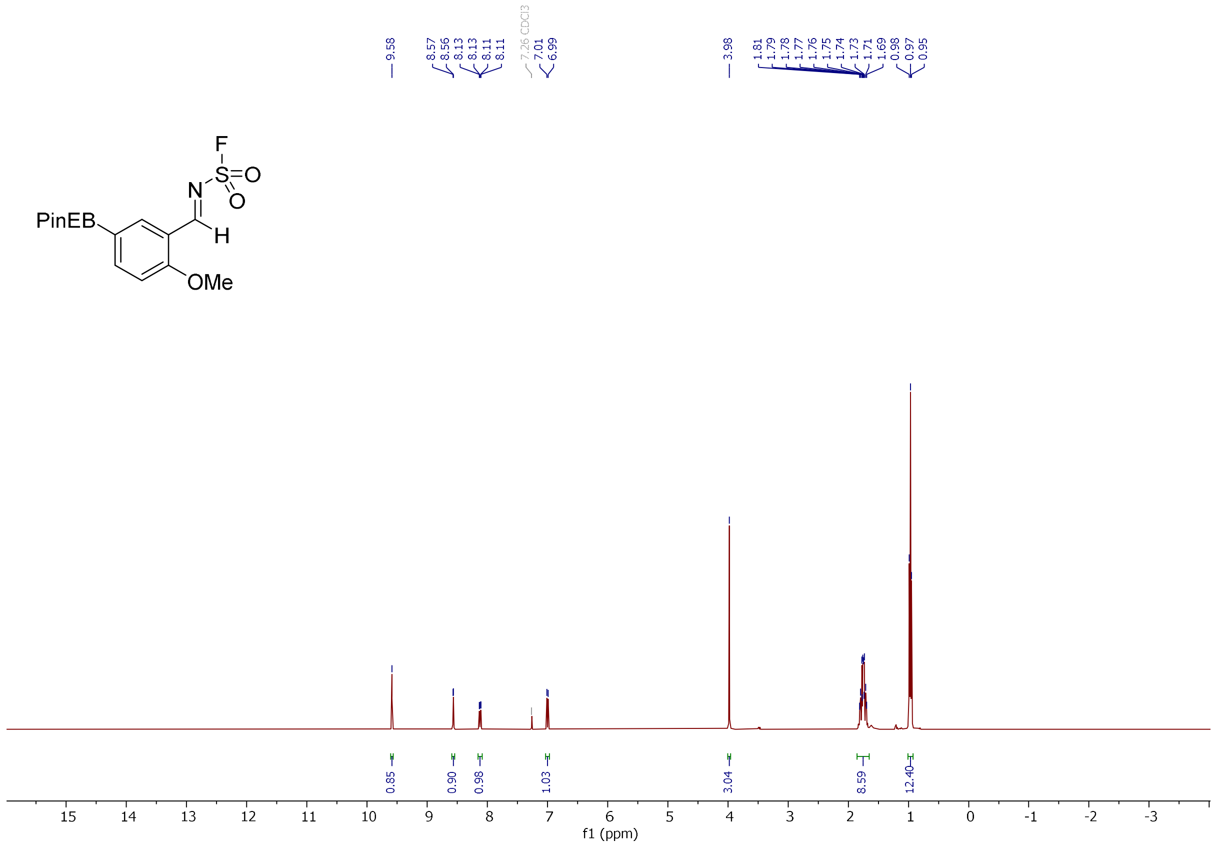

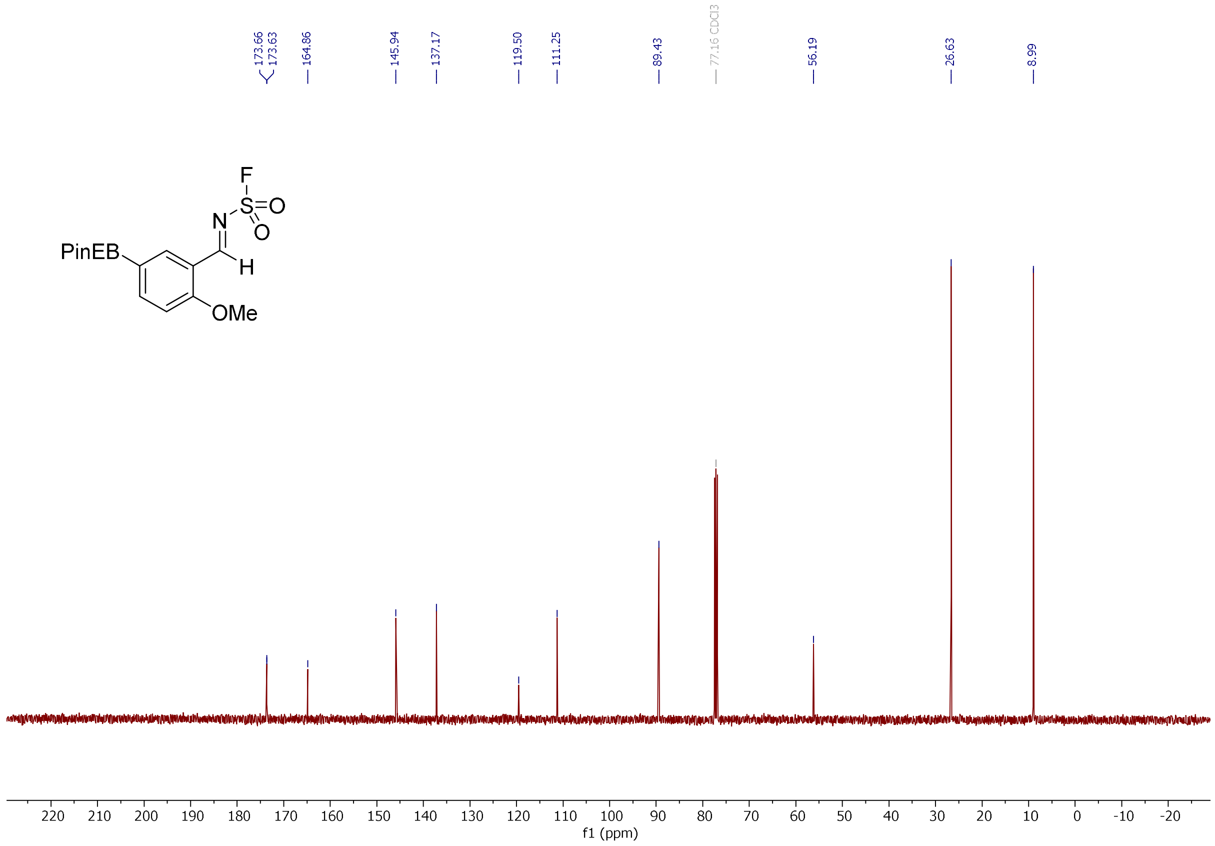
173.66
173.63
164.86
145.94
137.17
119.50
111.25
89.43
77.16 CDCl3
56.19
26.63
8.99
F
N
S
O
O
PinEB
H
OMe
f1 (ppm)

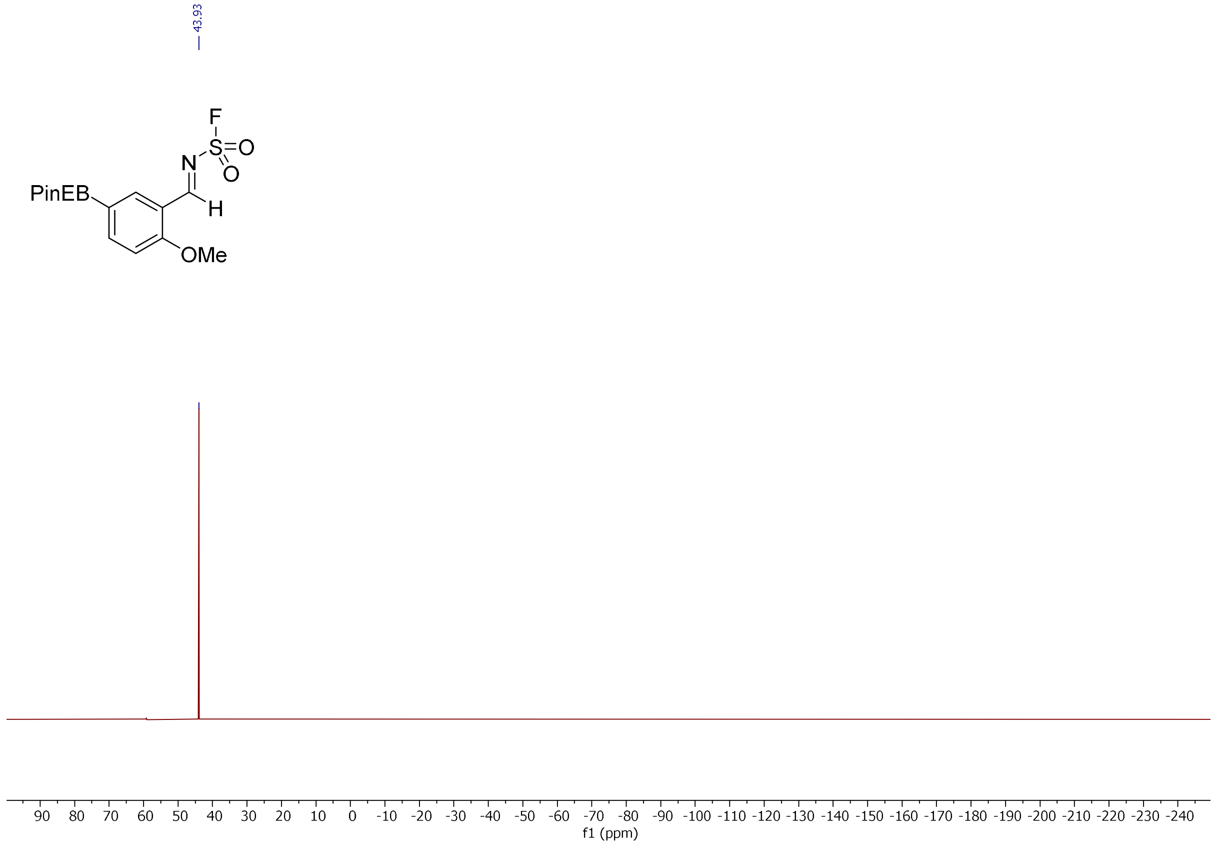
43.93
F
N
S
O
O
PinEB
H
OMe
f1 (ppm)

(4-Methoxy-3-(4,4,5,5-tetramethyl-1,3,2-dioxaborolan-2-yl)benzylidene)sulfamoyl fluoride (I-19)

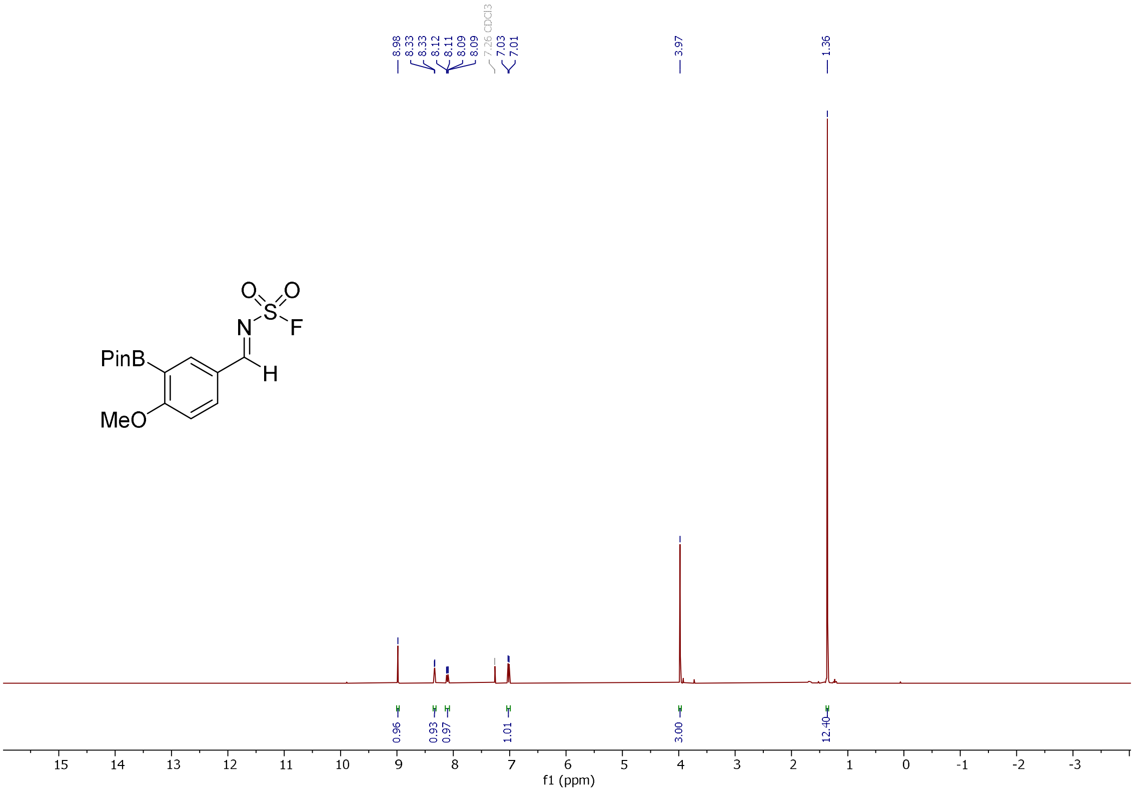

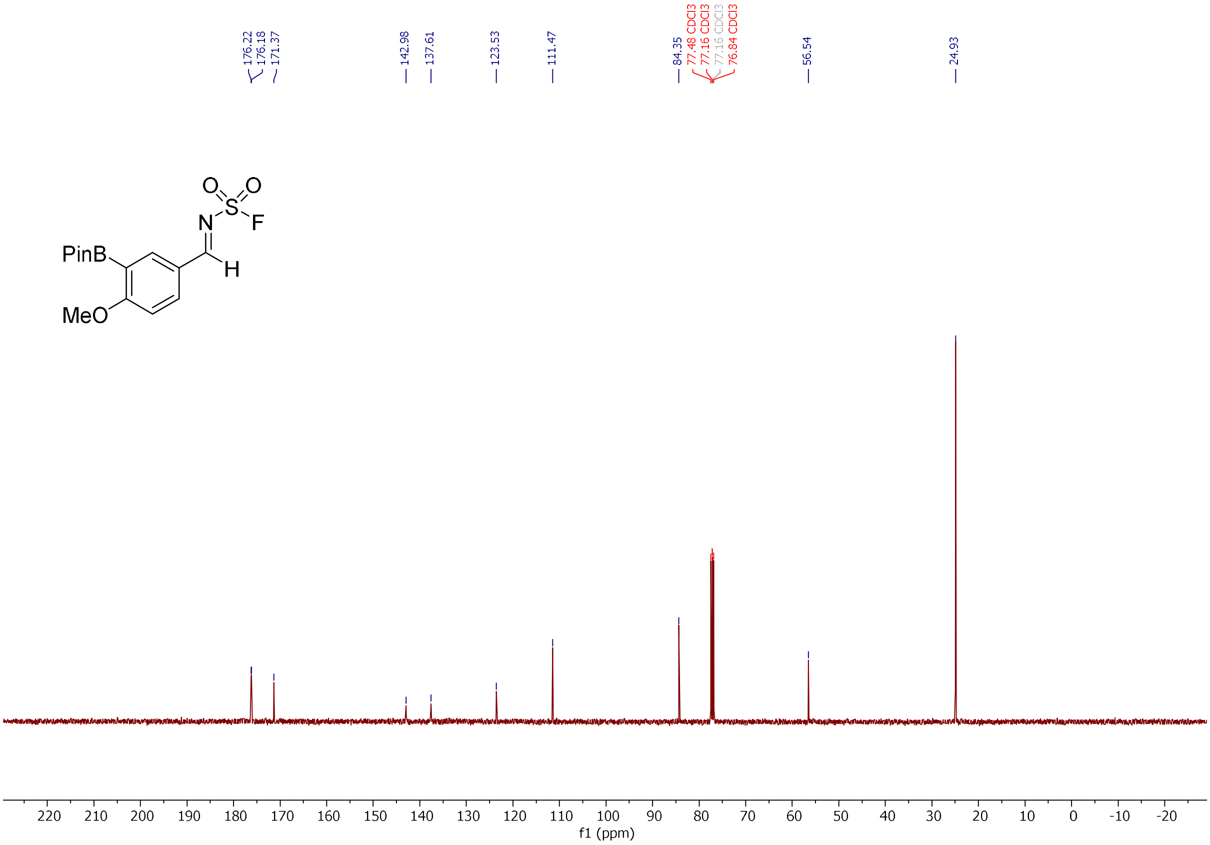

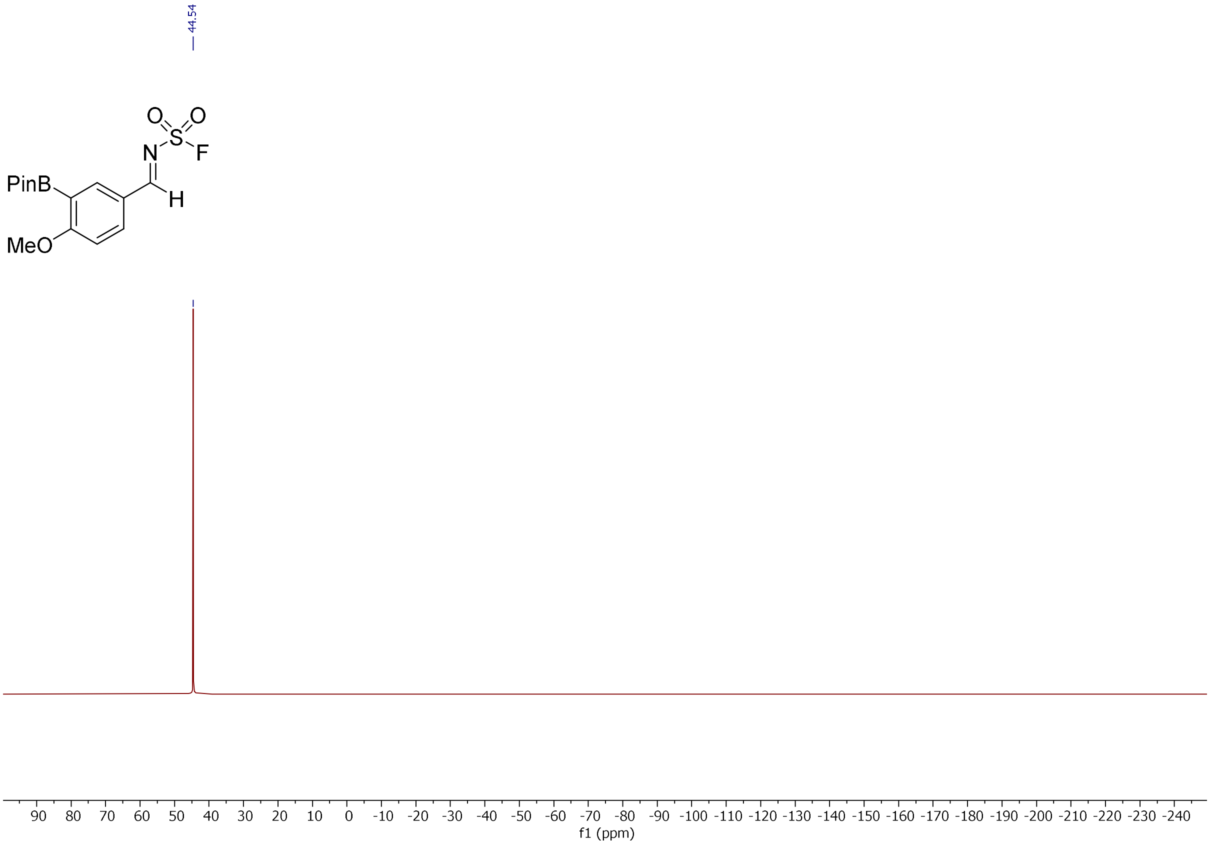

((6-Methoxypyridin-3-yl)methylene)sulfamoyl fluoride (I-20)

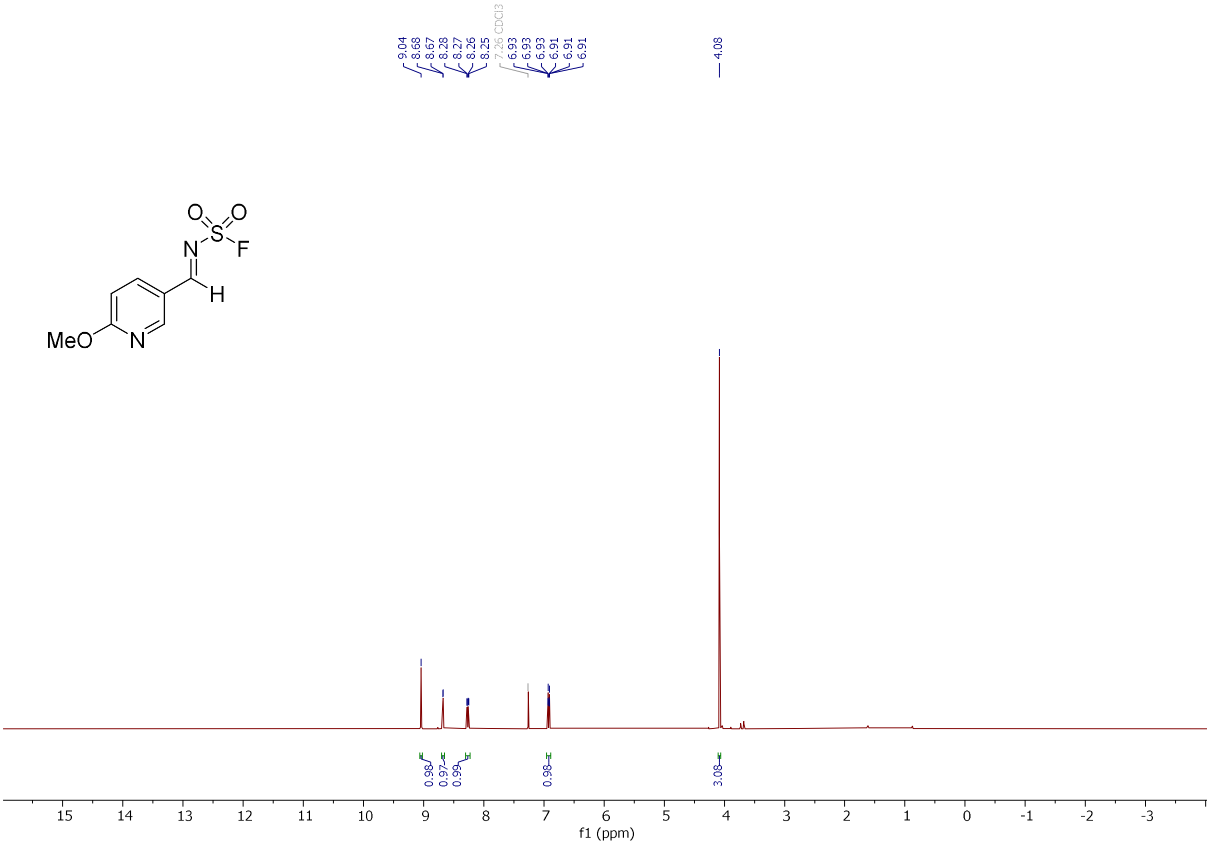

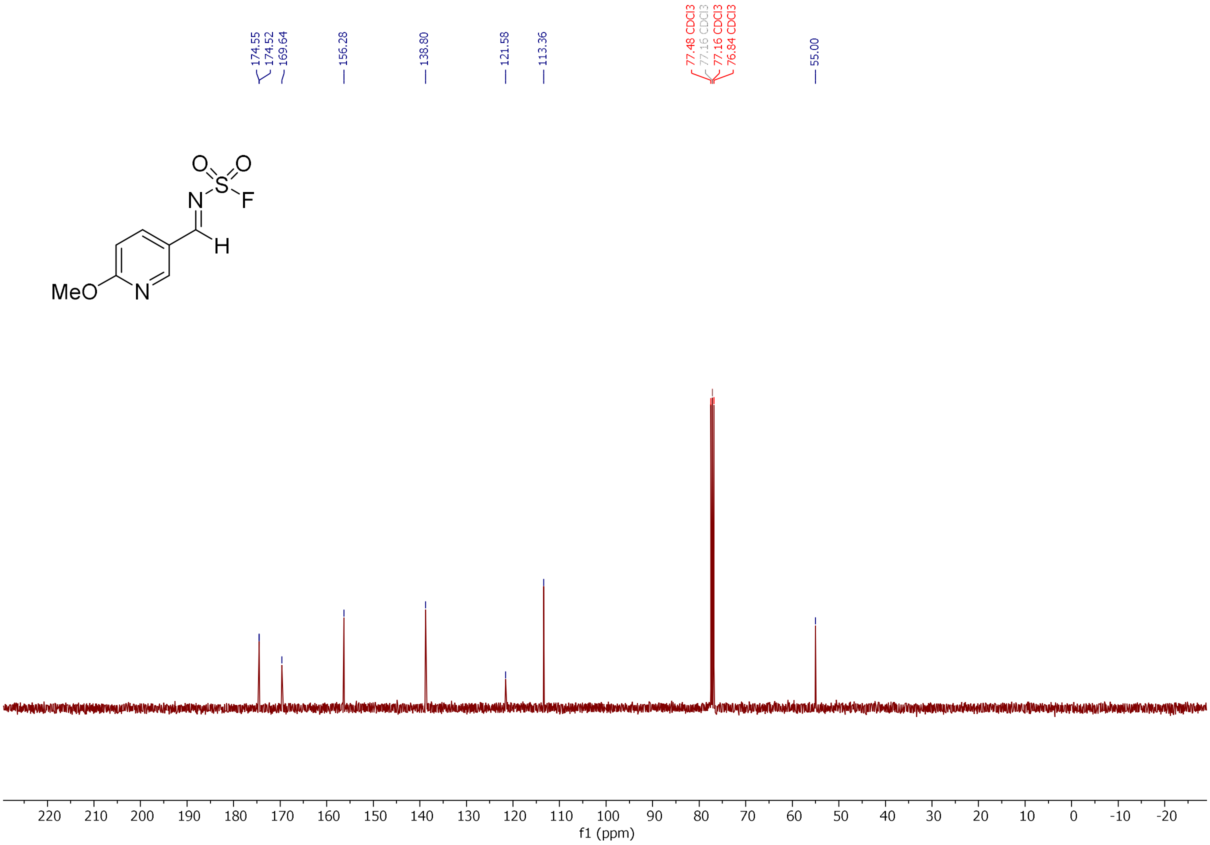
174.55
174.52
169.64
156.28
138.80
121.58
113.36
77.48 CDCl3
77.16 CDCl3
77.16 CDCl3
76.84 CDCl3
55.00
MeO
N
H
N
S
O
O
F
f1 (ppm)

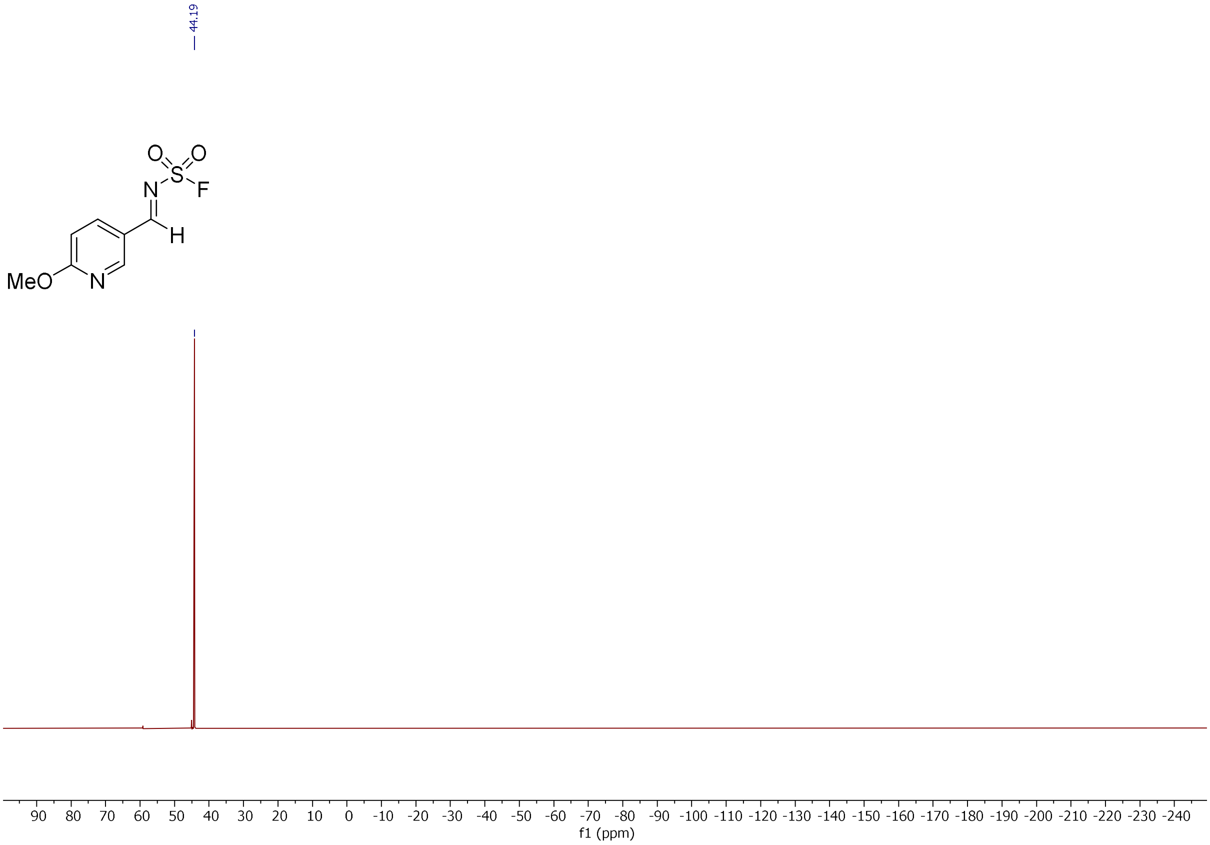
44.19
MeO
N
H
N
S
O
O
F
f1 (ppm)

Aldehydes

4-(Trimethylsilyl)benzaldehyde (A-21)

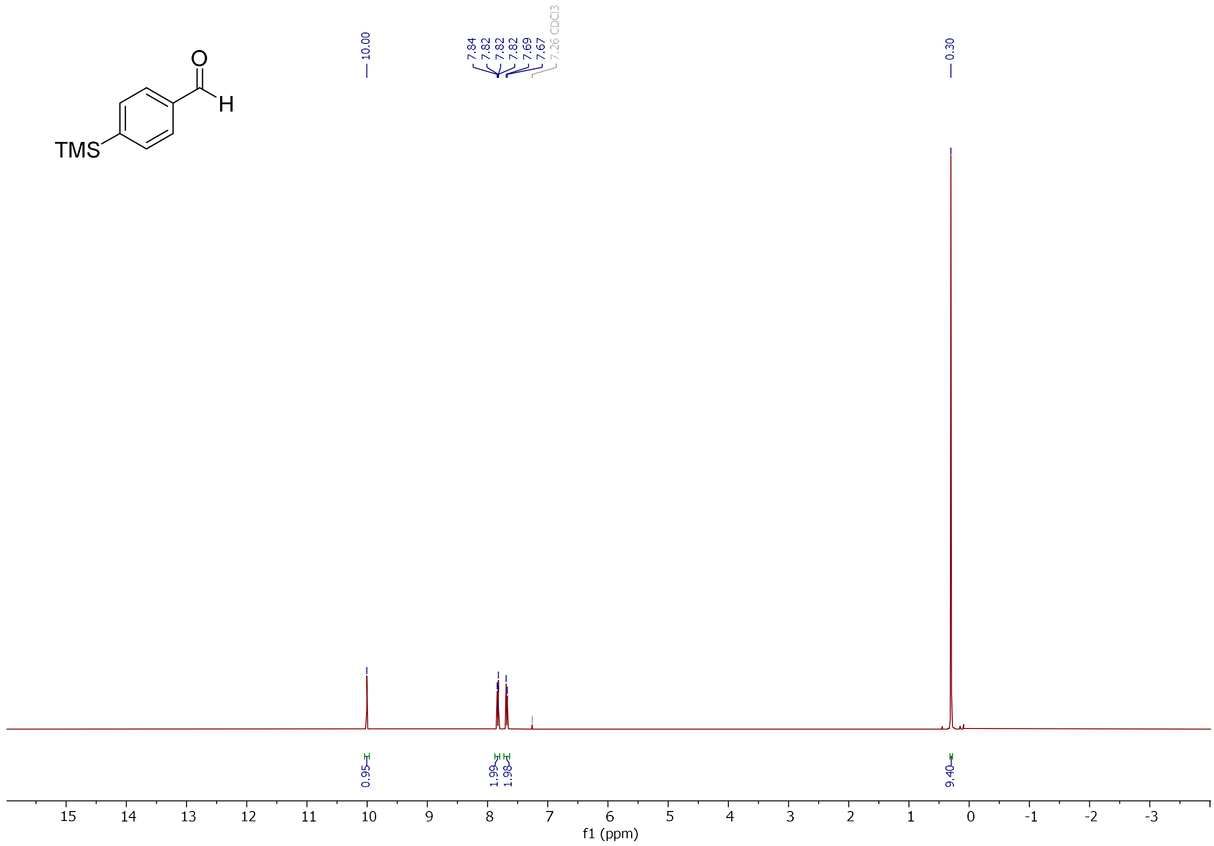

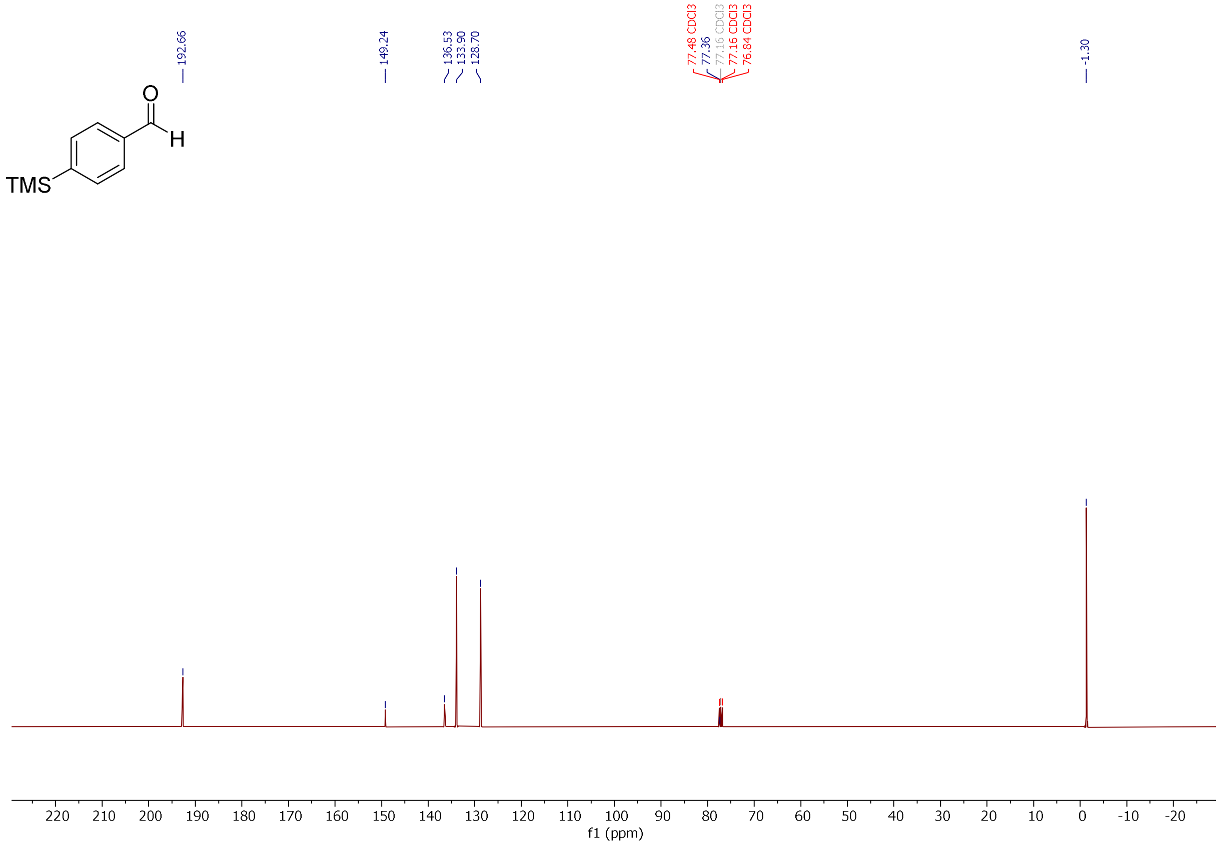

4-(1,3-Dioxoisoindolin-2-yl)benzaldehyde (A-22)

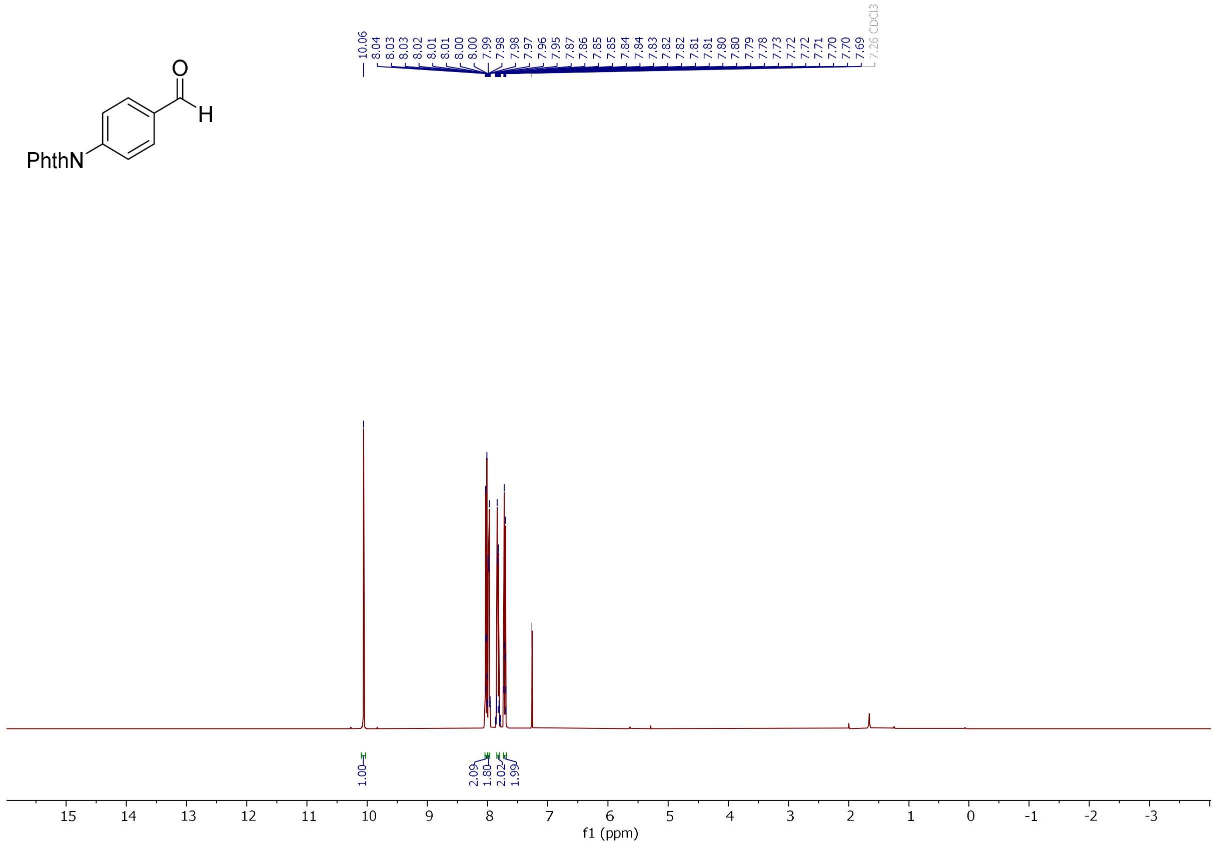

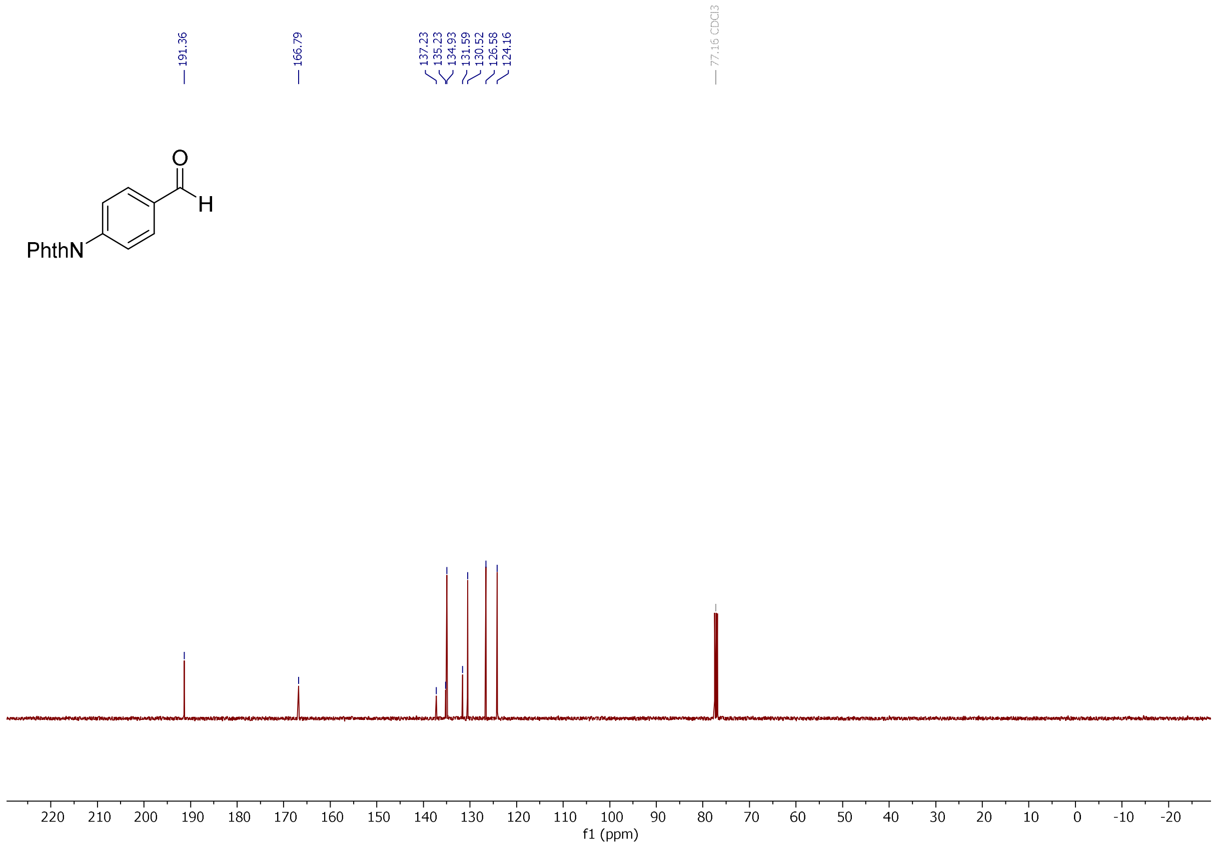

4-Formylphenyl pivalate (A-23)

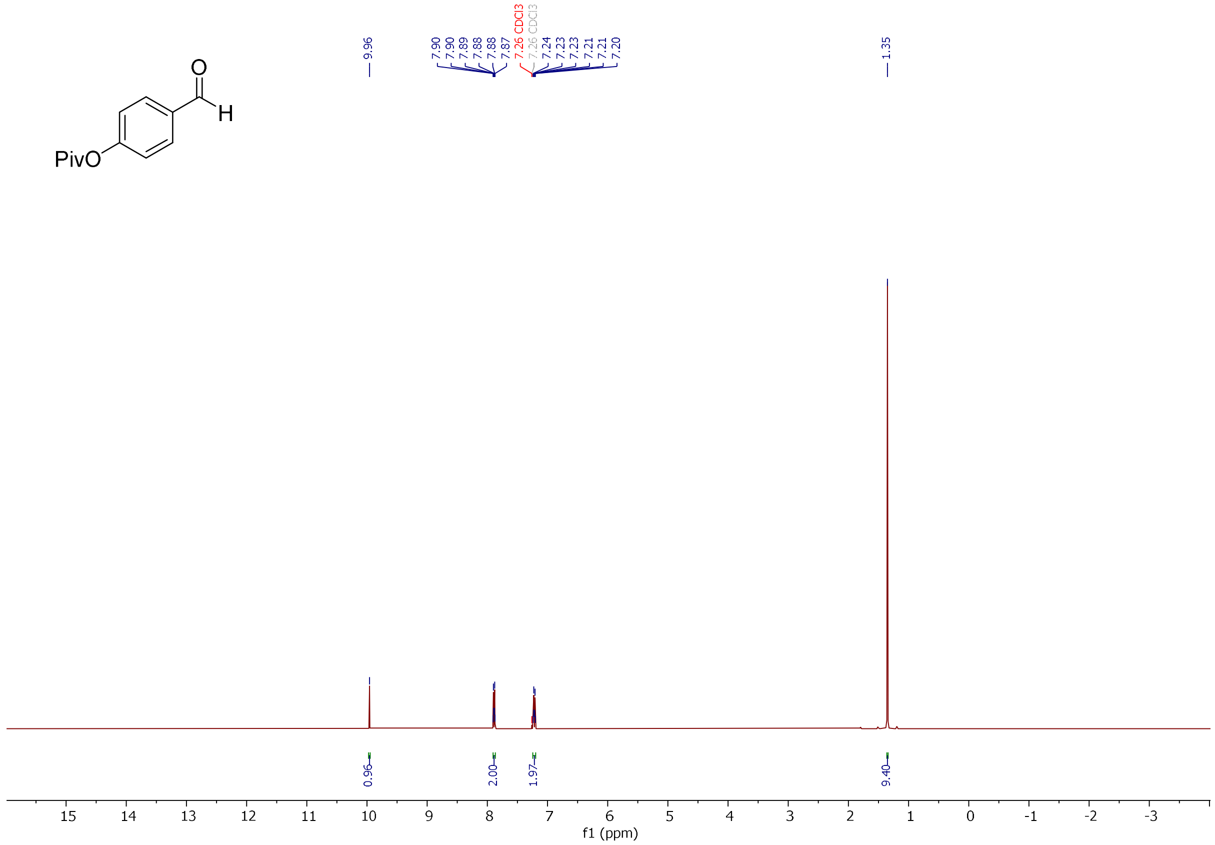

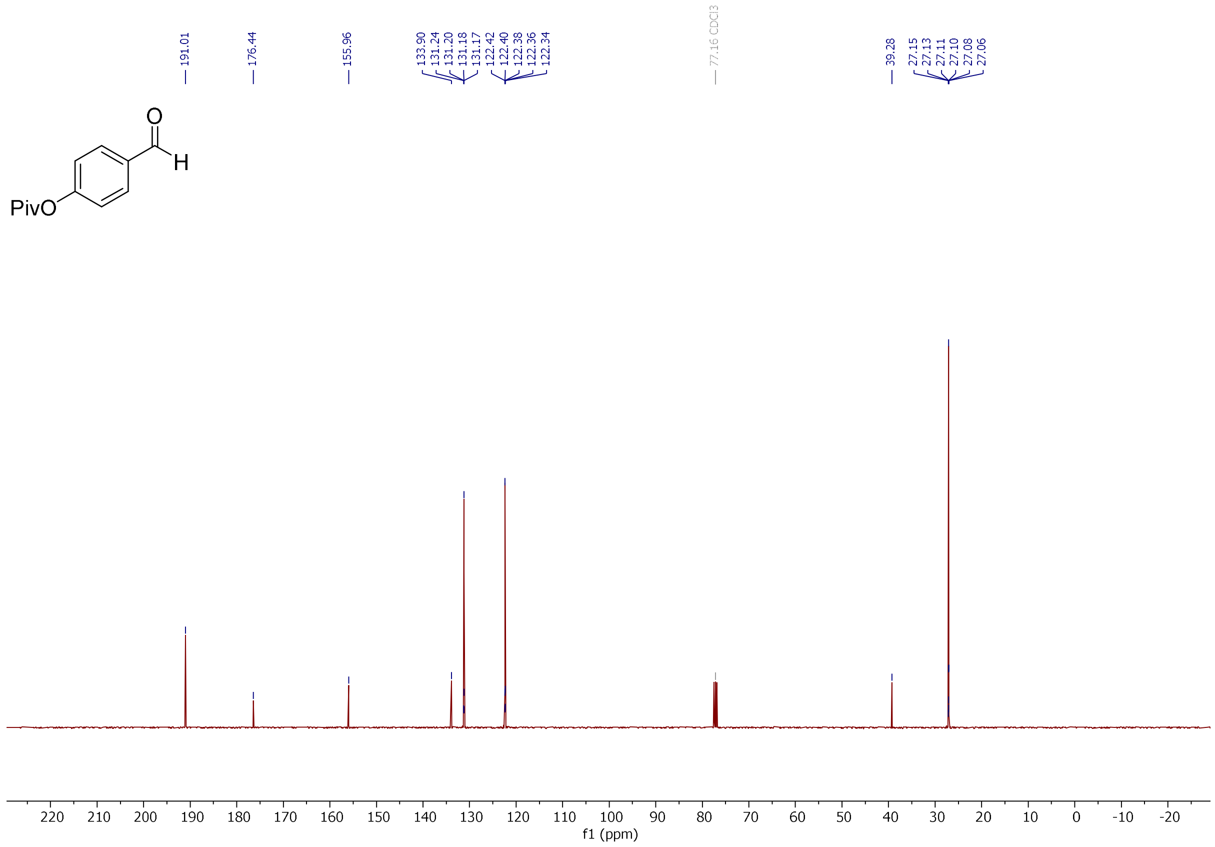

2-Methoxy-5-(4,4,5,5-tetraethyl-1,3,2-dioxaborolan-2-yl)benzaldehyde (A-24)

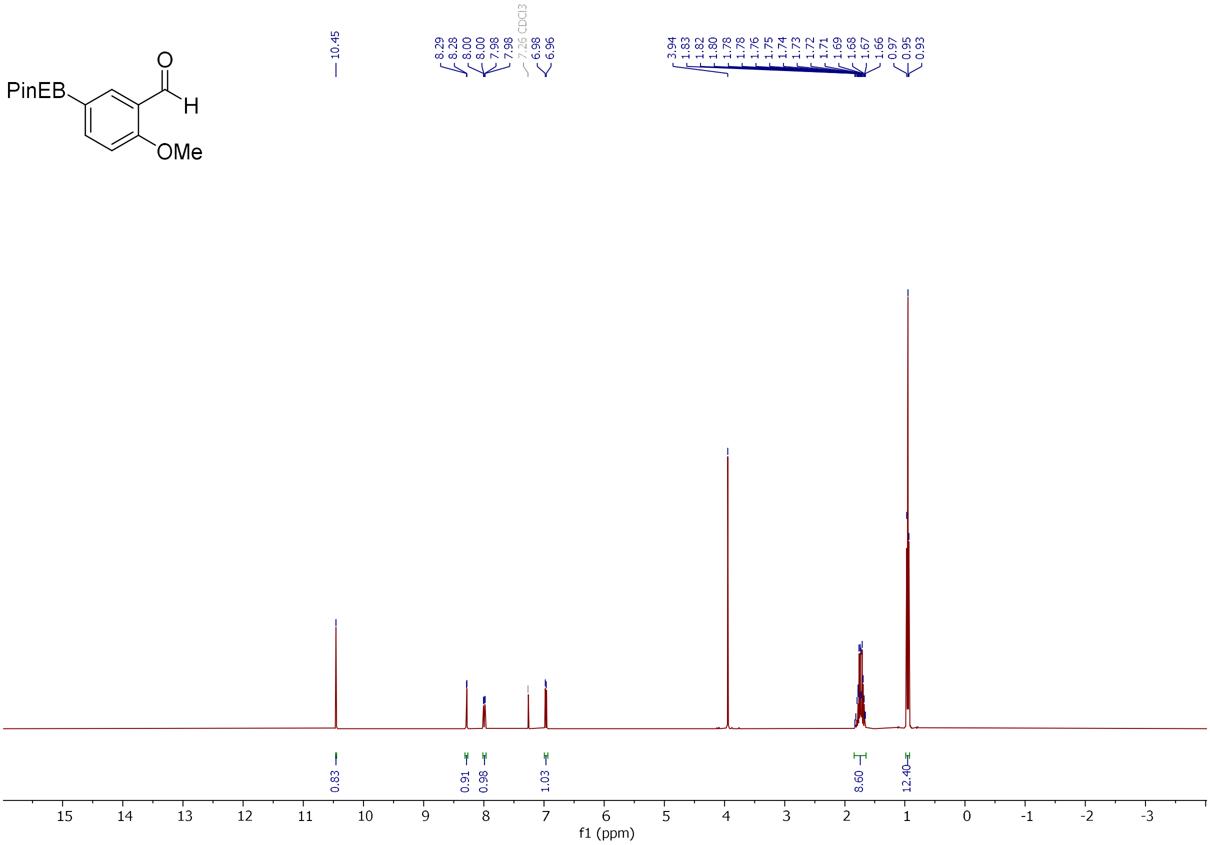

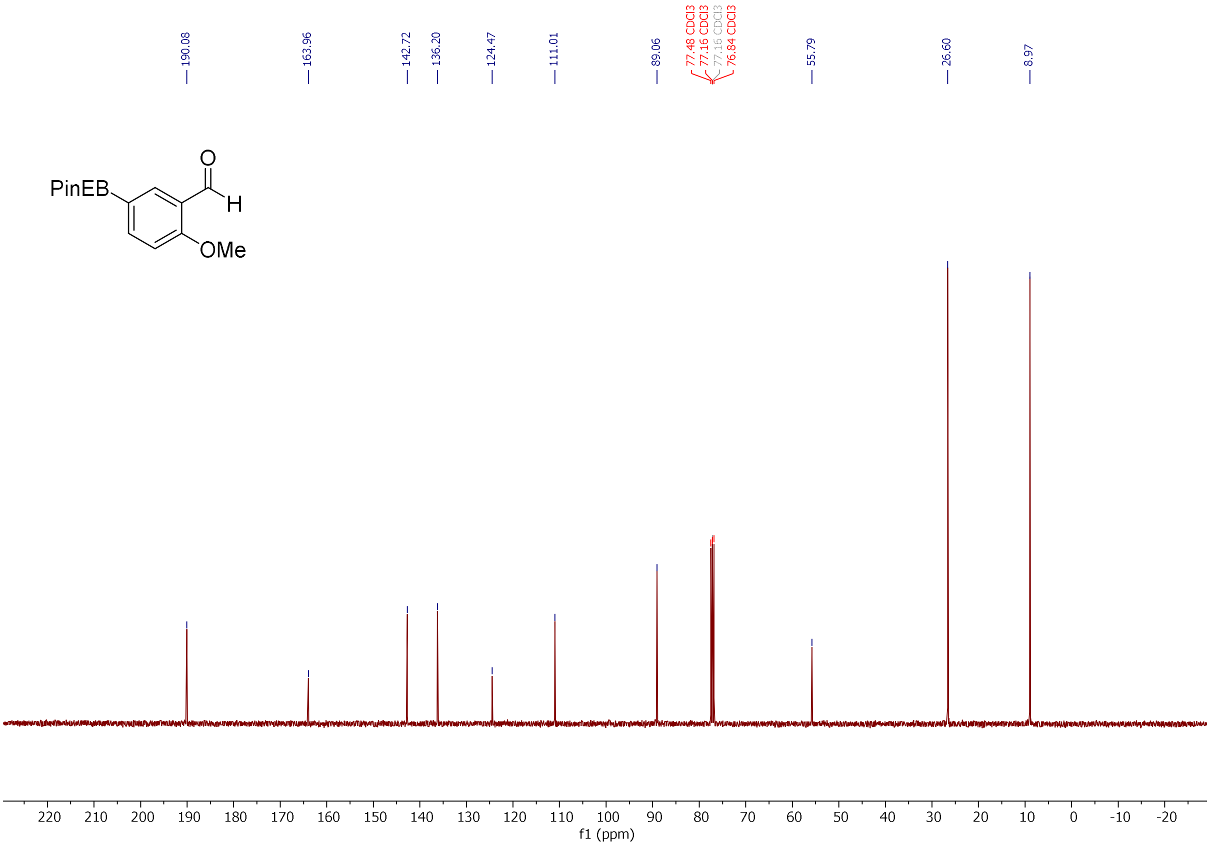

4-Methoxy-3-(4,4,5,5-tetramethyl-1,3,2-dioxaborolan-2-yl)benzaldehyde (A-25)

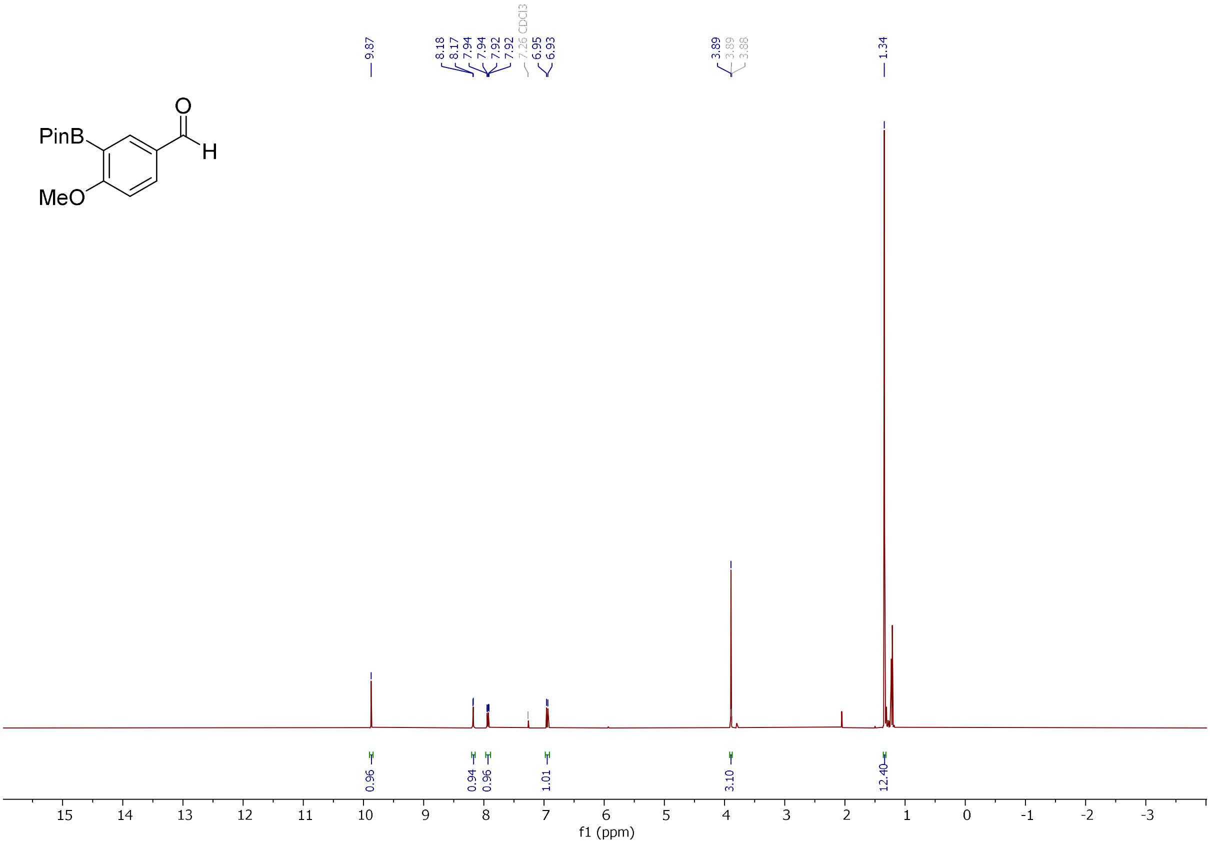

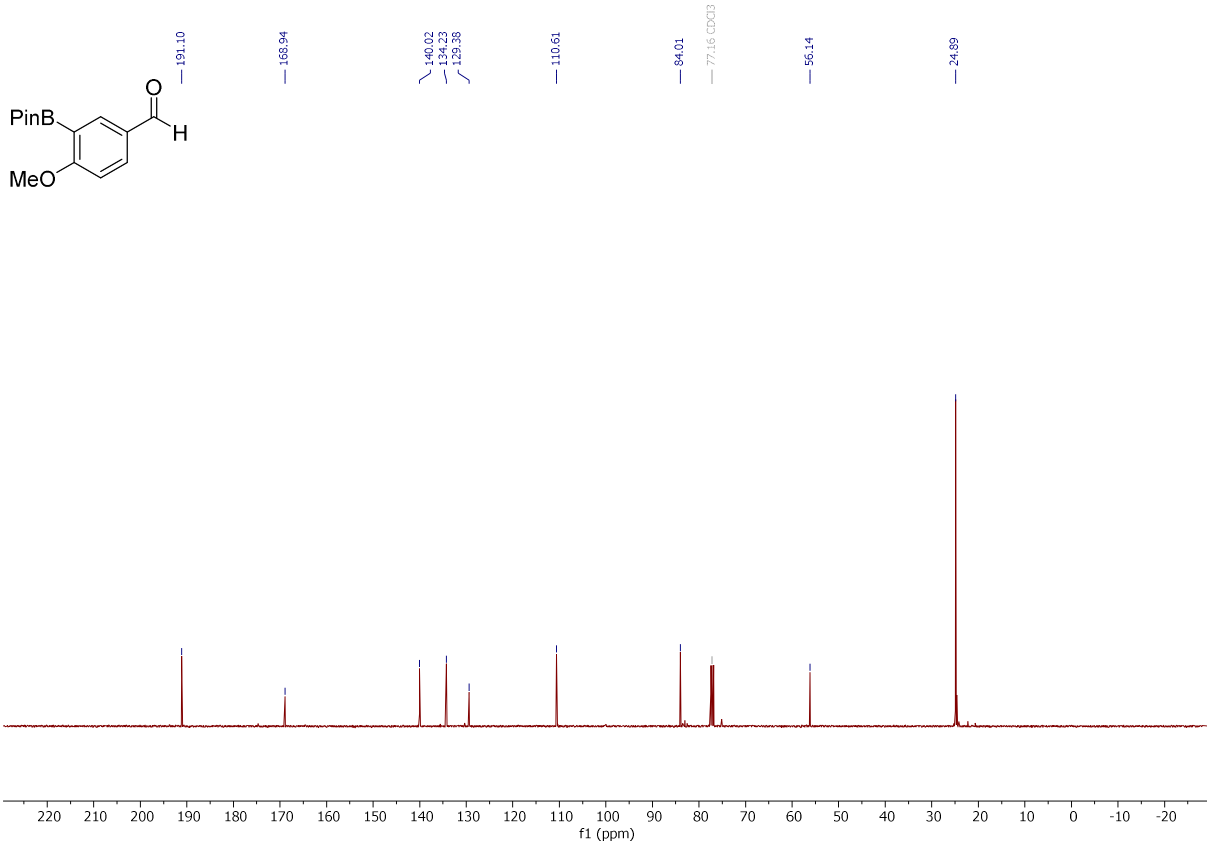

2,4-Dimethoxypyrimidine-5-carbaldehyde (A-26)

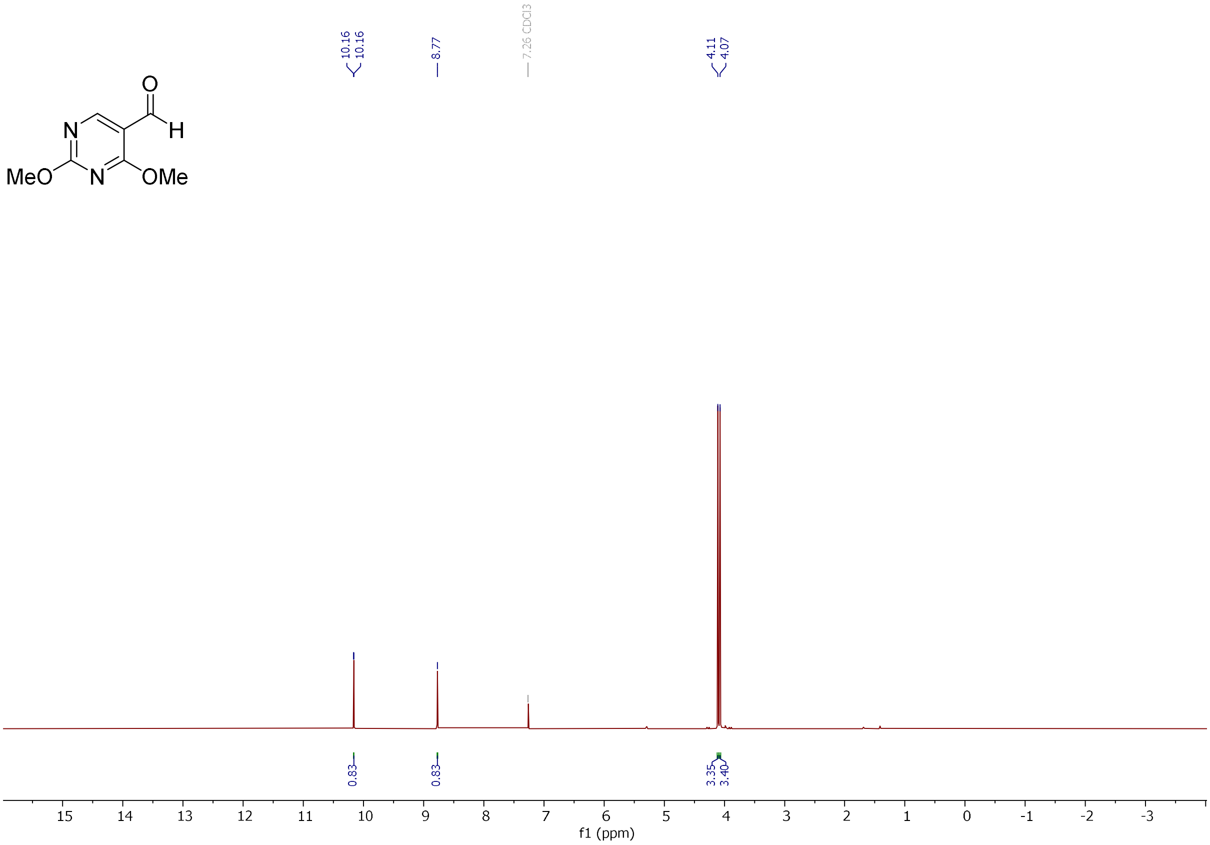

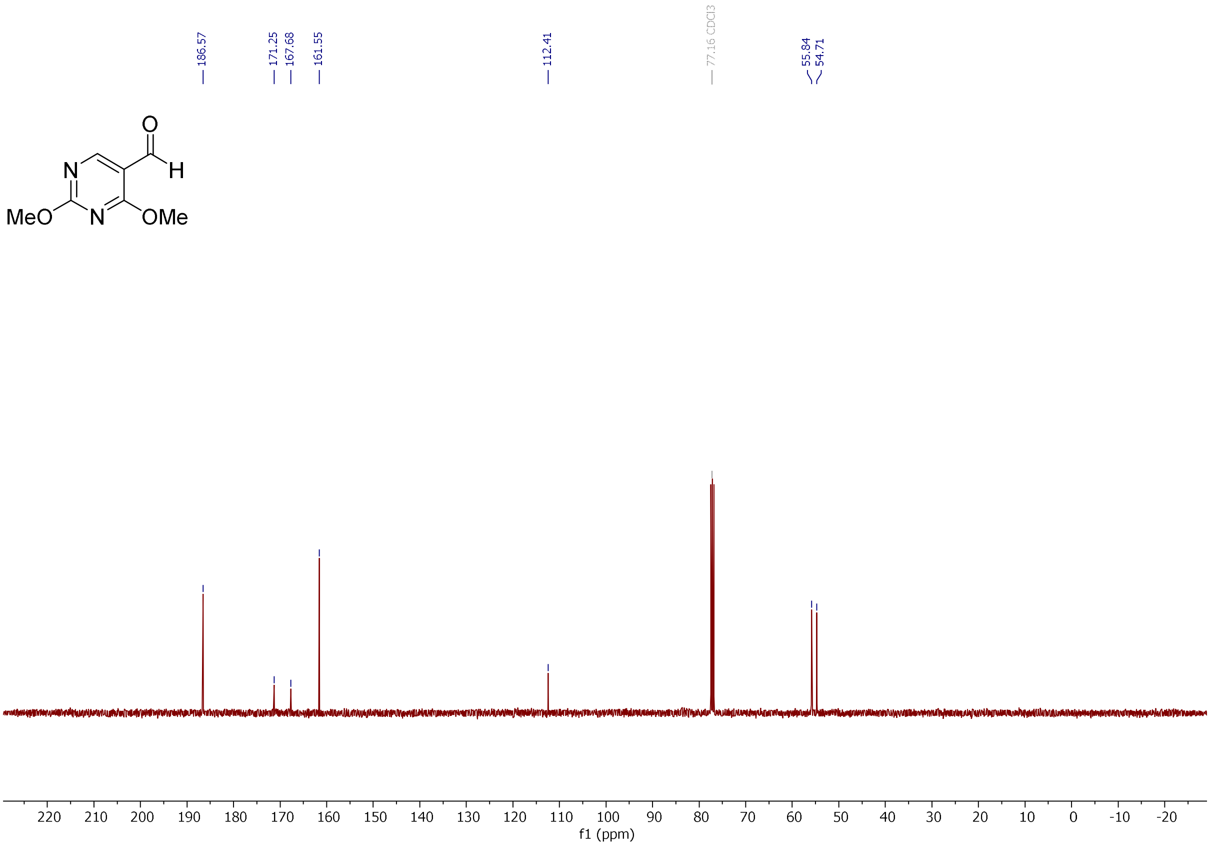

5-Bromo-6-methoxynicotinaldehyde (A-27)

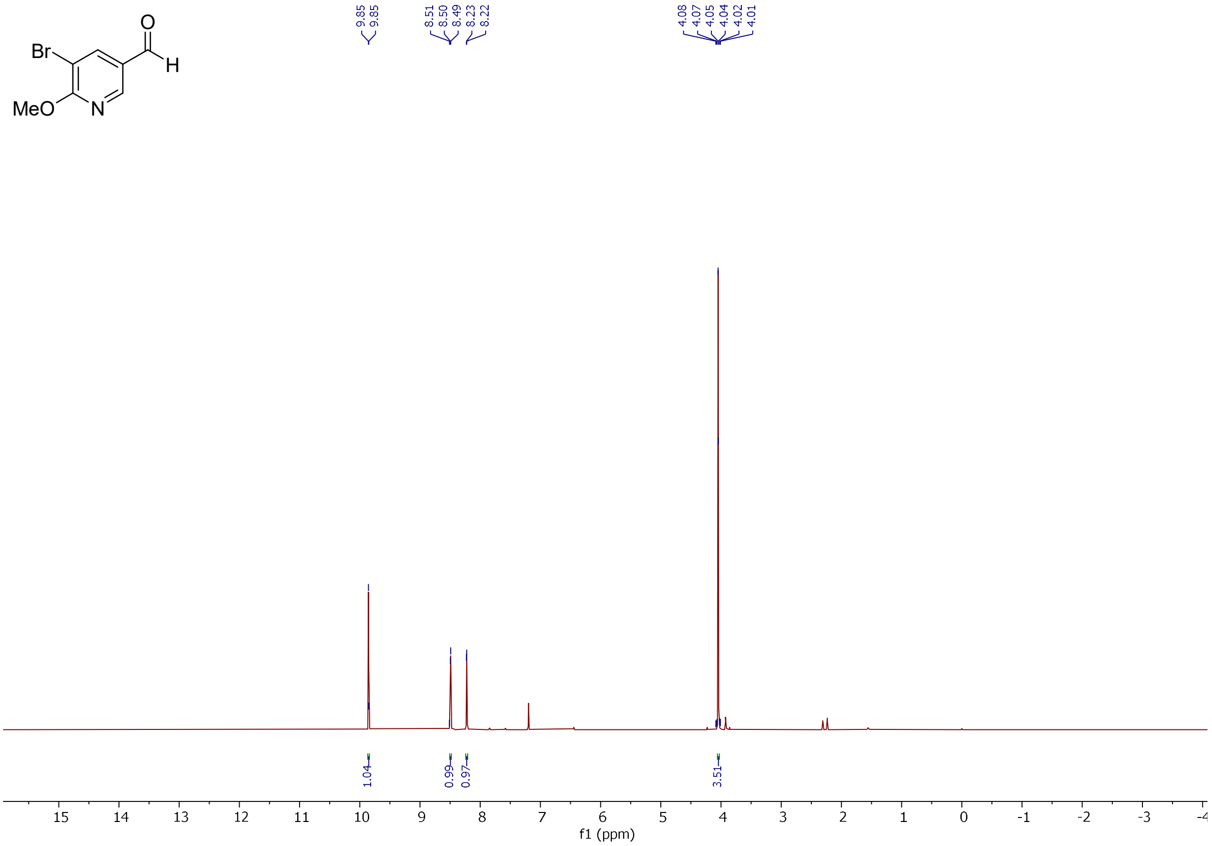

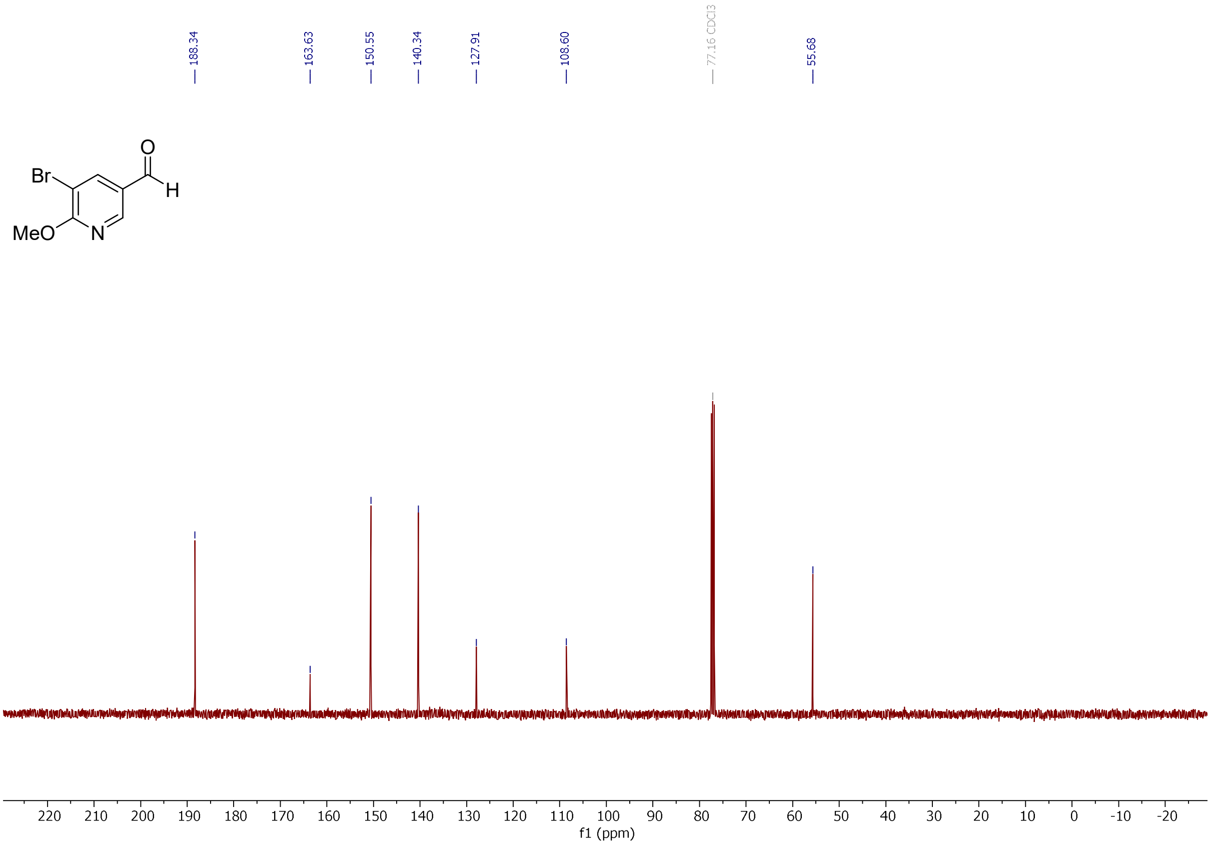

6-Methoxy-5-(4,4,5,5-tetramethyl-1,3,2-dioxaborolan-2-yl)nicotinaldehyde (A-28)

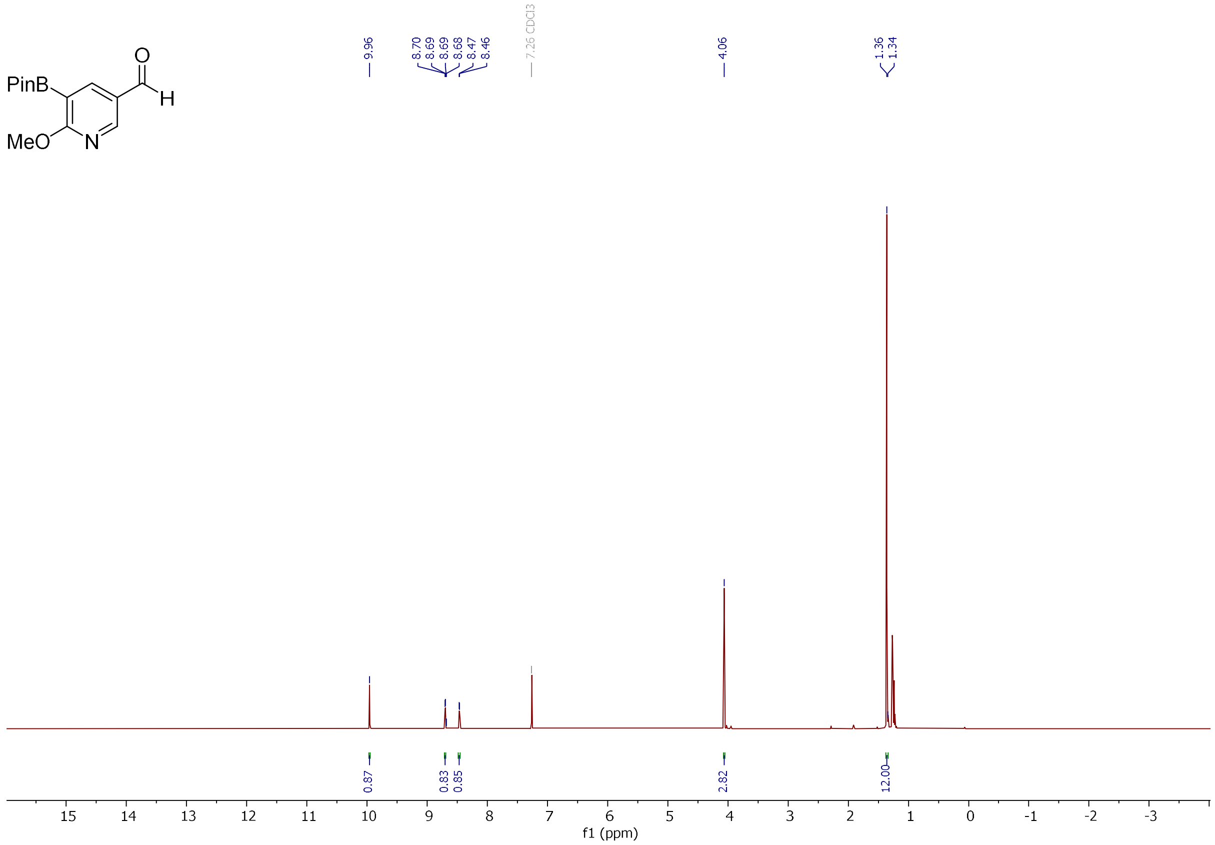

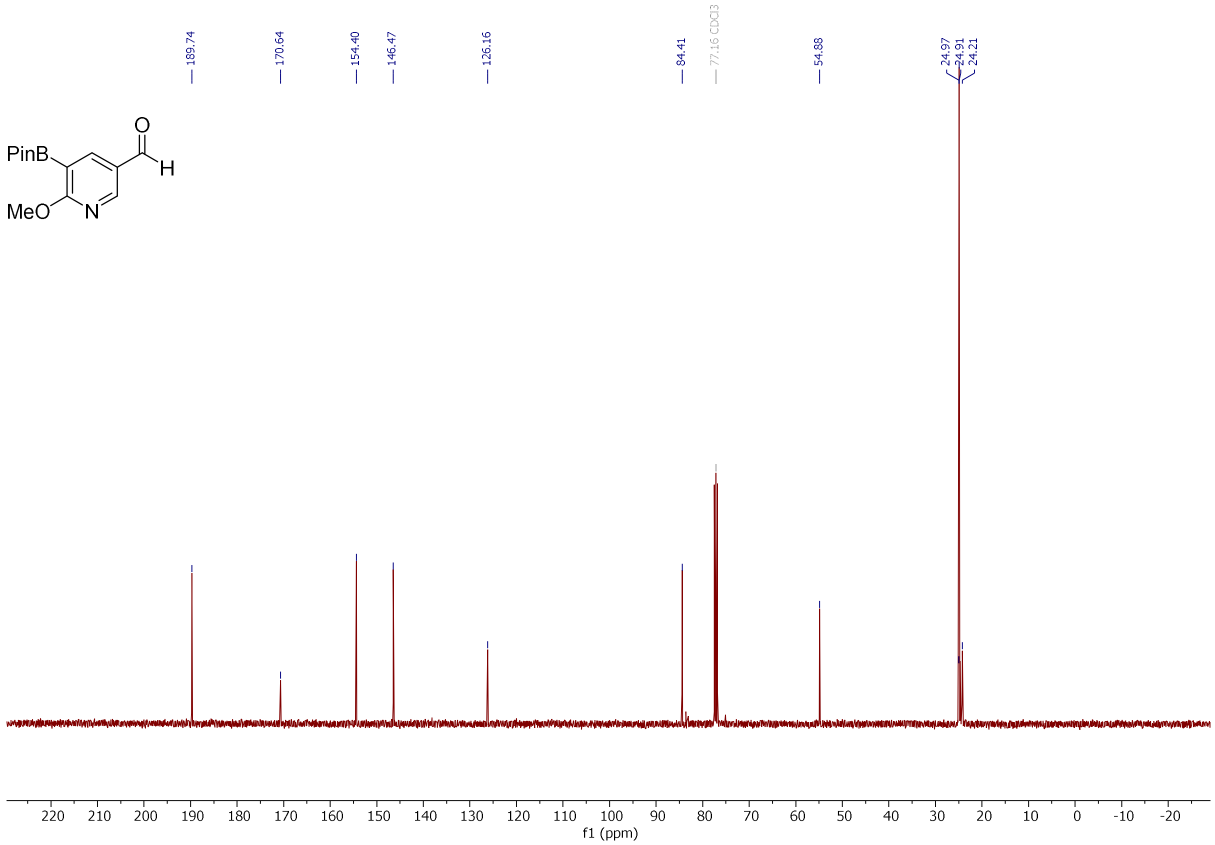

Alkenes

3-Methylbut-3-en-1-yl 4-methylbenzenesulfonate (O-29)

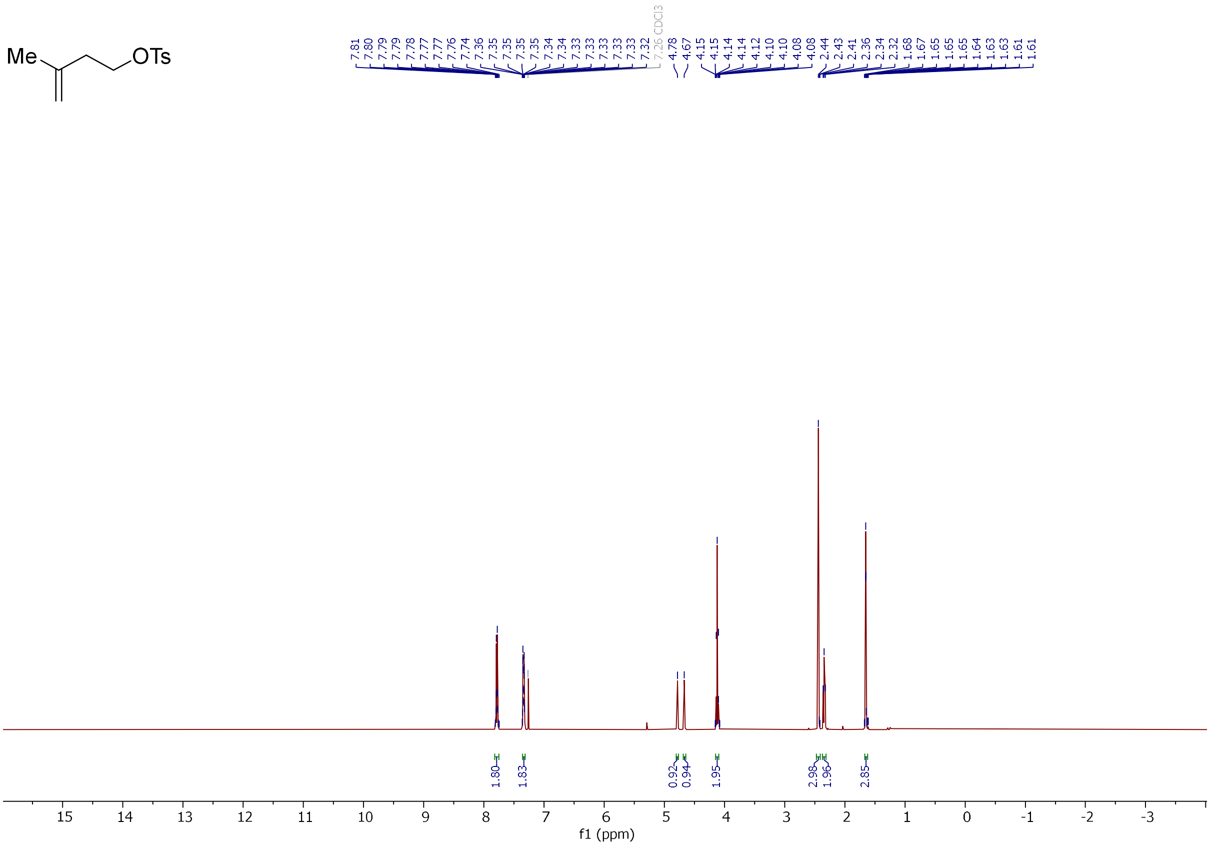

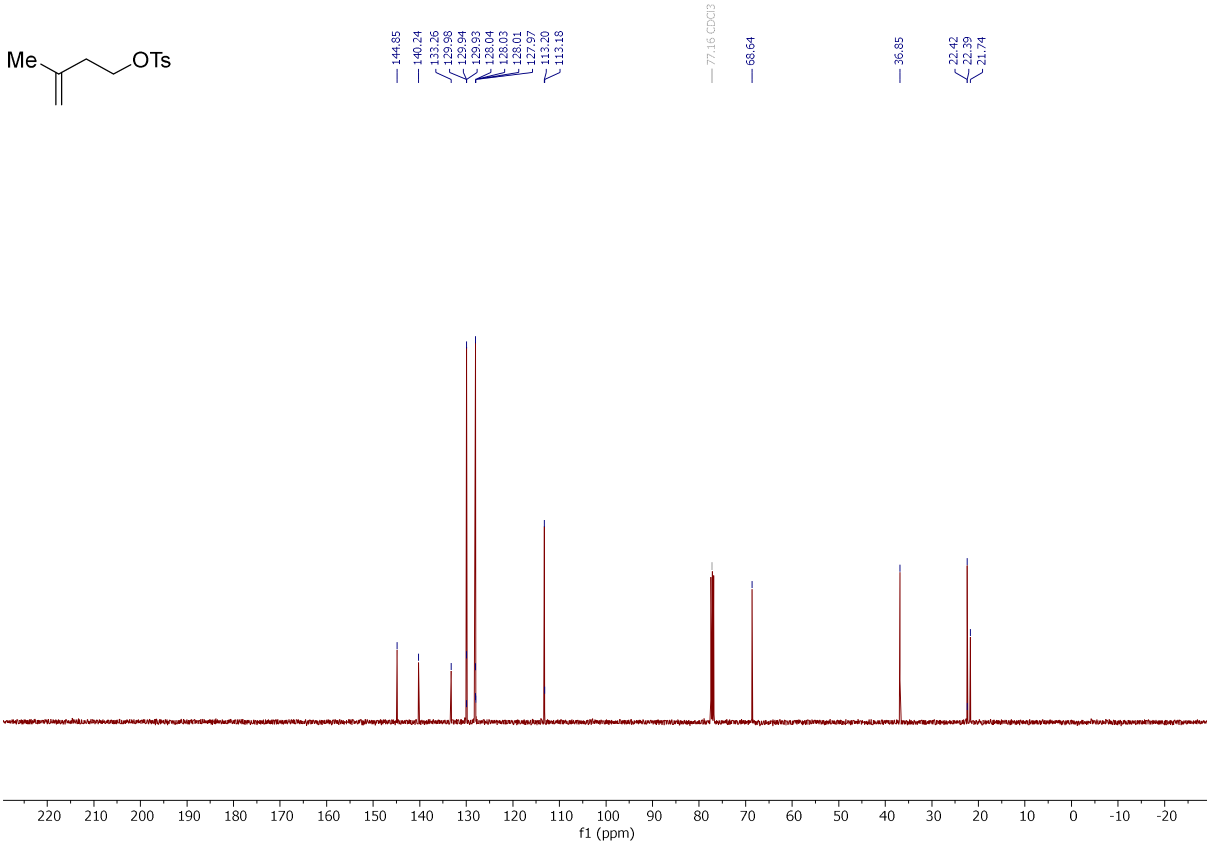

5-((3-Methylbut-3-en-1-yl)thio)-1-phenyl-1H-tetrazole (O-30)

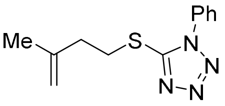

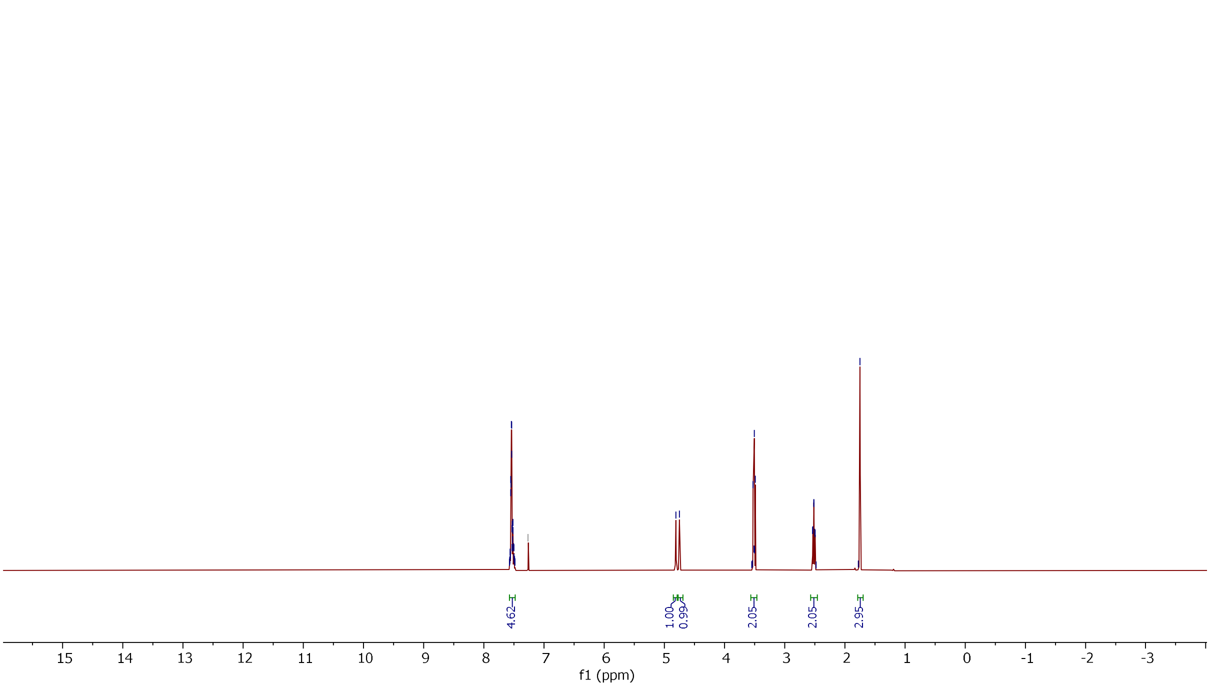

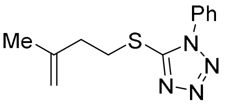

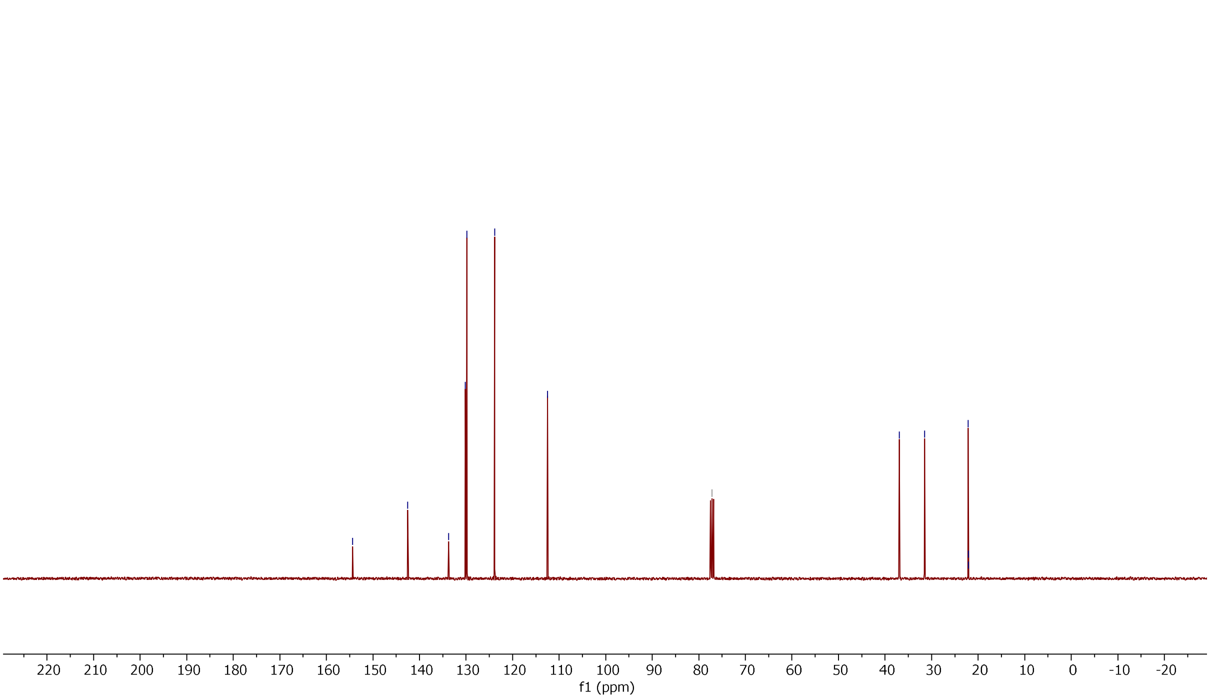

5-((3-Methylbut-3-en-1-yl)sulfonyl)-1-phenyl-1H-tetrazole (O-31)

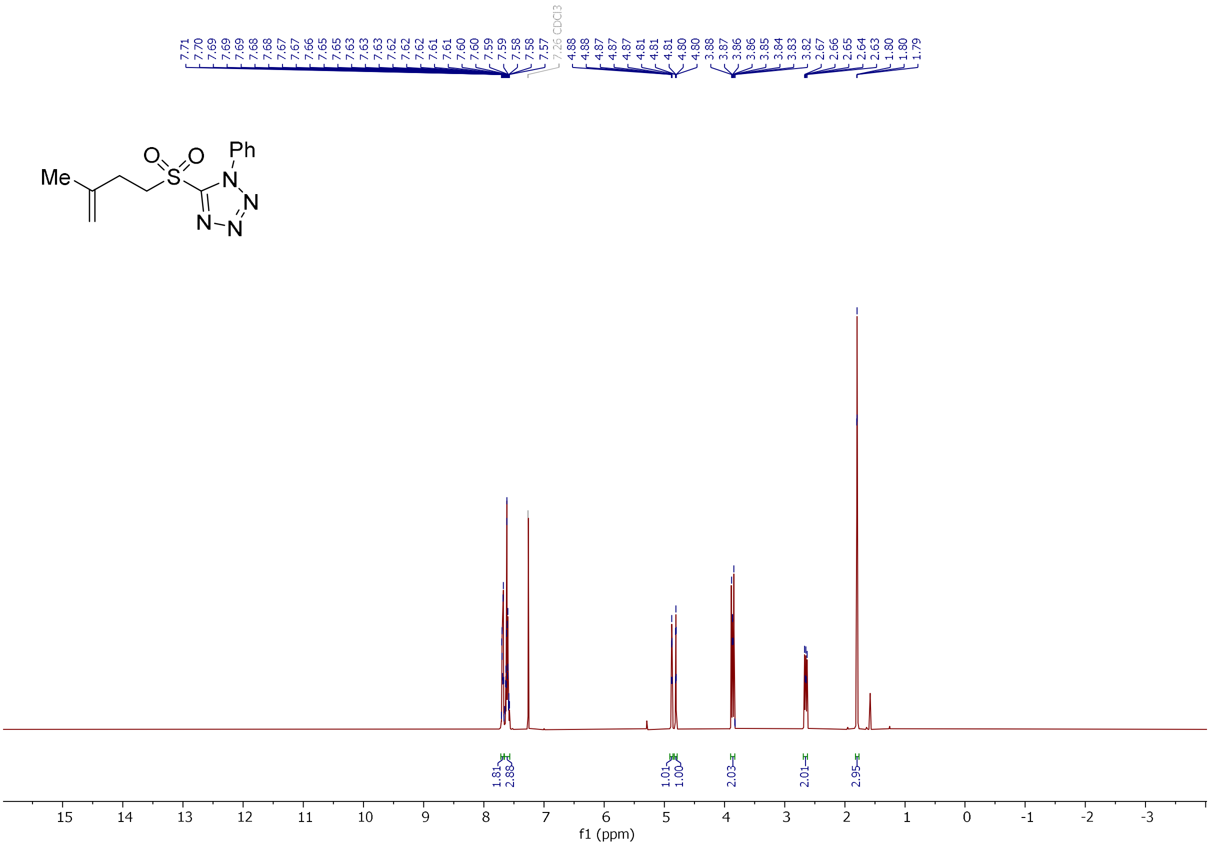

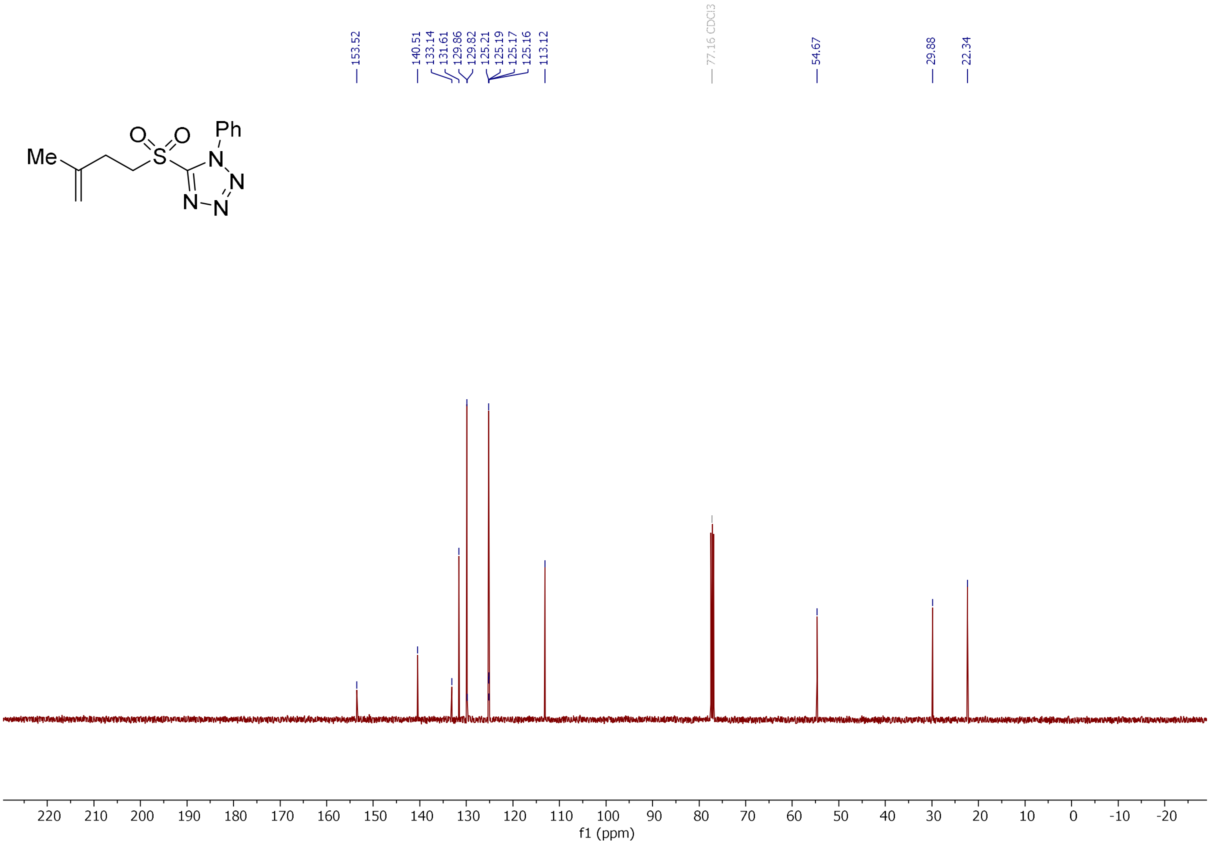

3-Methylbut-3-en-1-yl but-3-enoate (O-32)

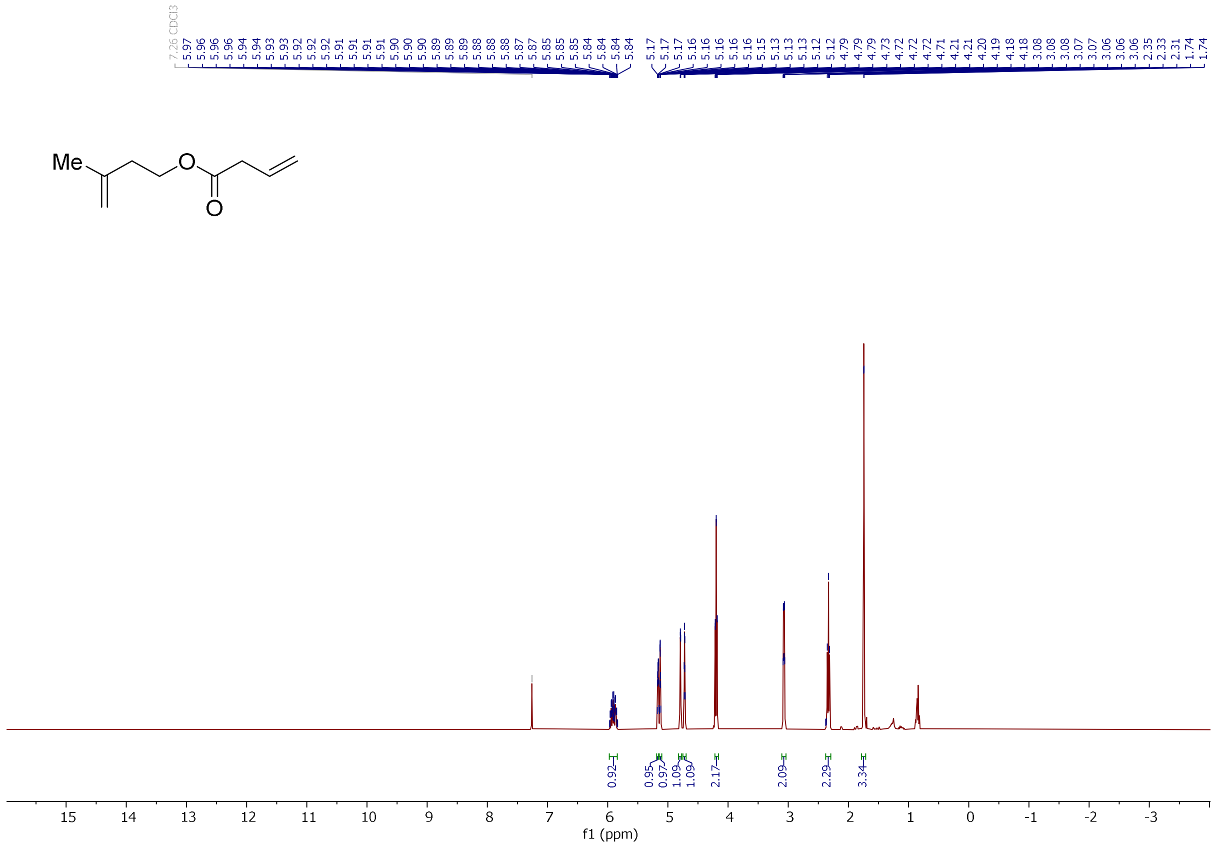

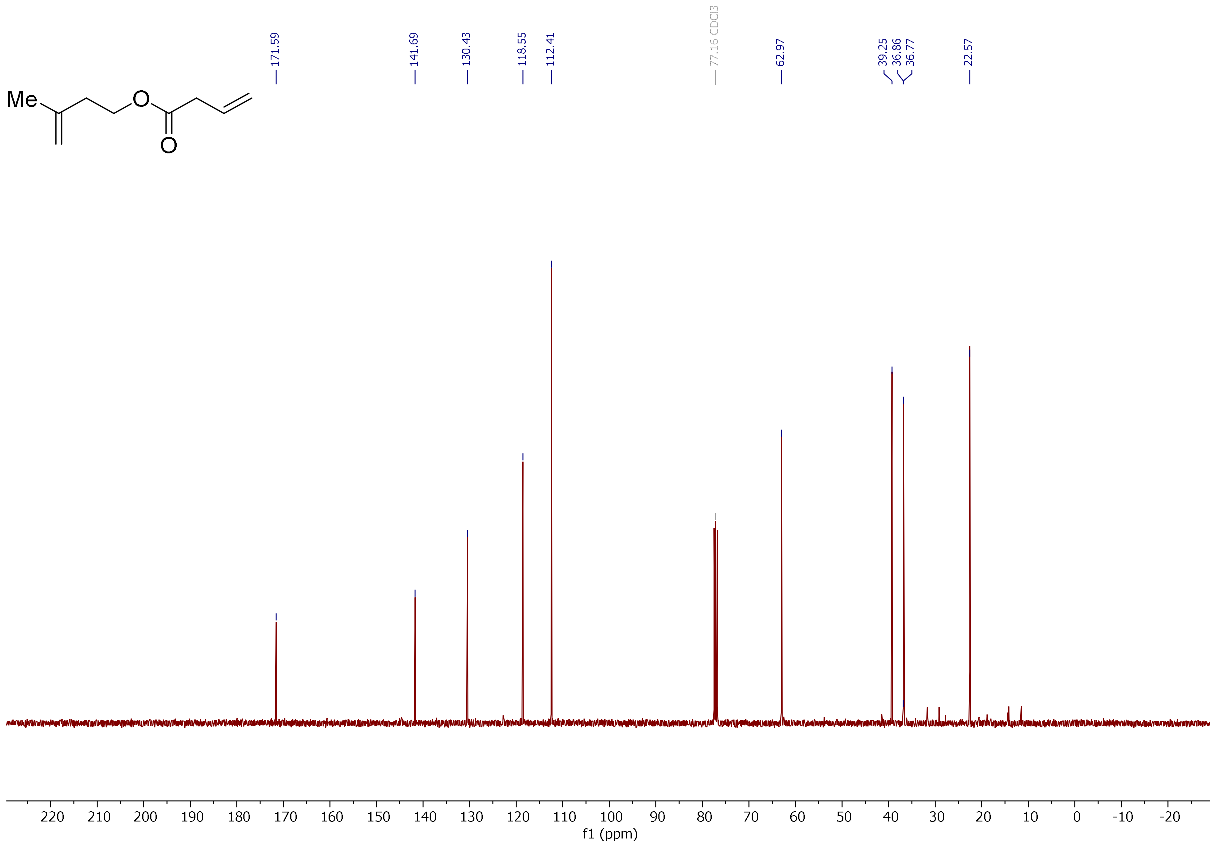

Tetrahydro-1H-spiro[pentalene-2,2'-[1,3]dioxolan]-5(3H)-one (O-33)

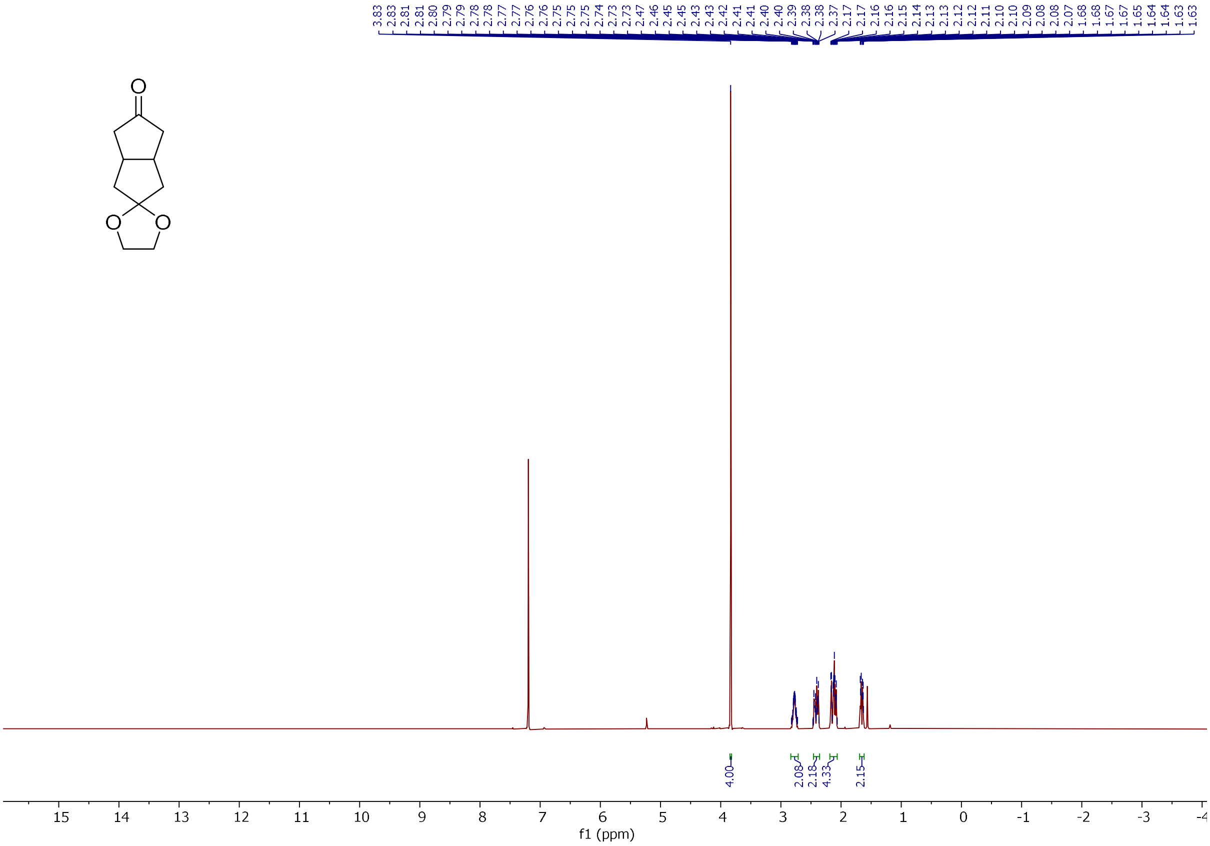

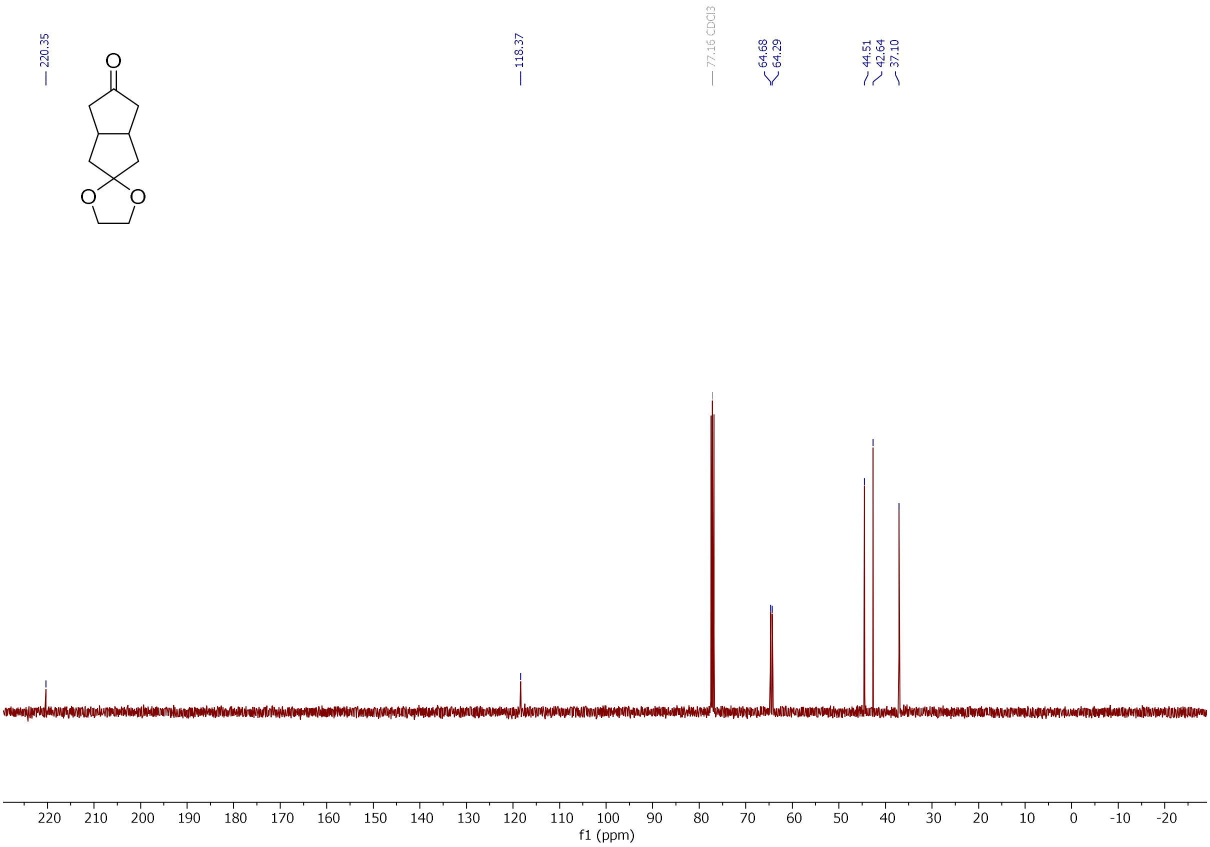

5-Methylenehexahydro-1H-spiro[pentalene-2,2'-[1,3]dioxolane] (O-34)

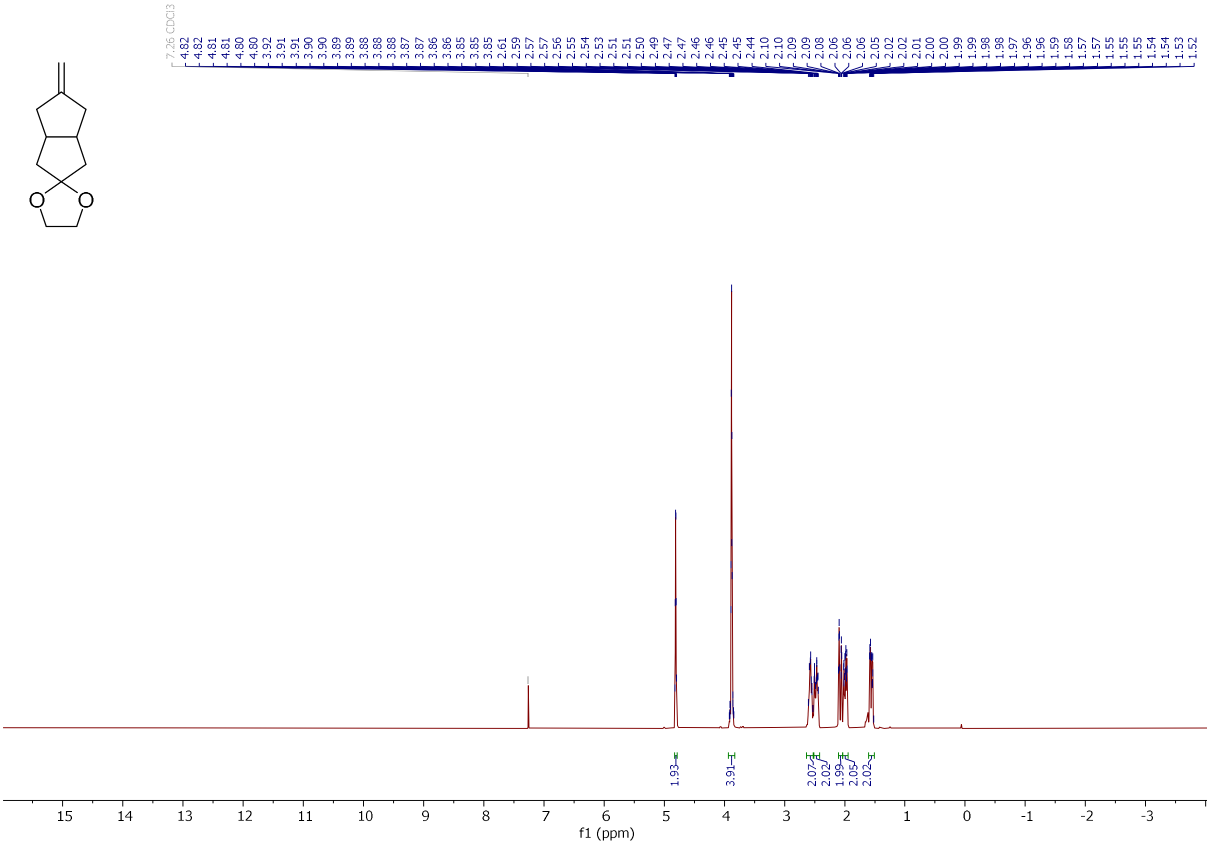

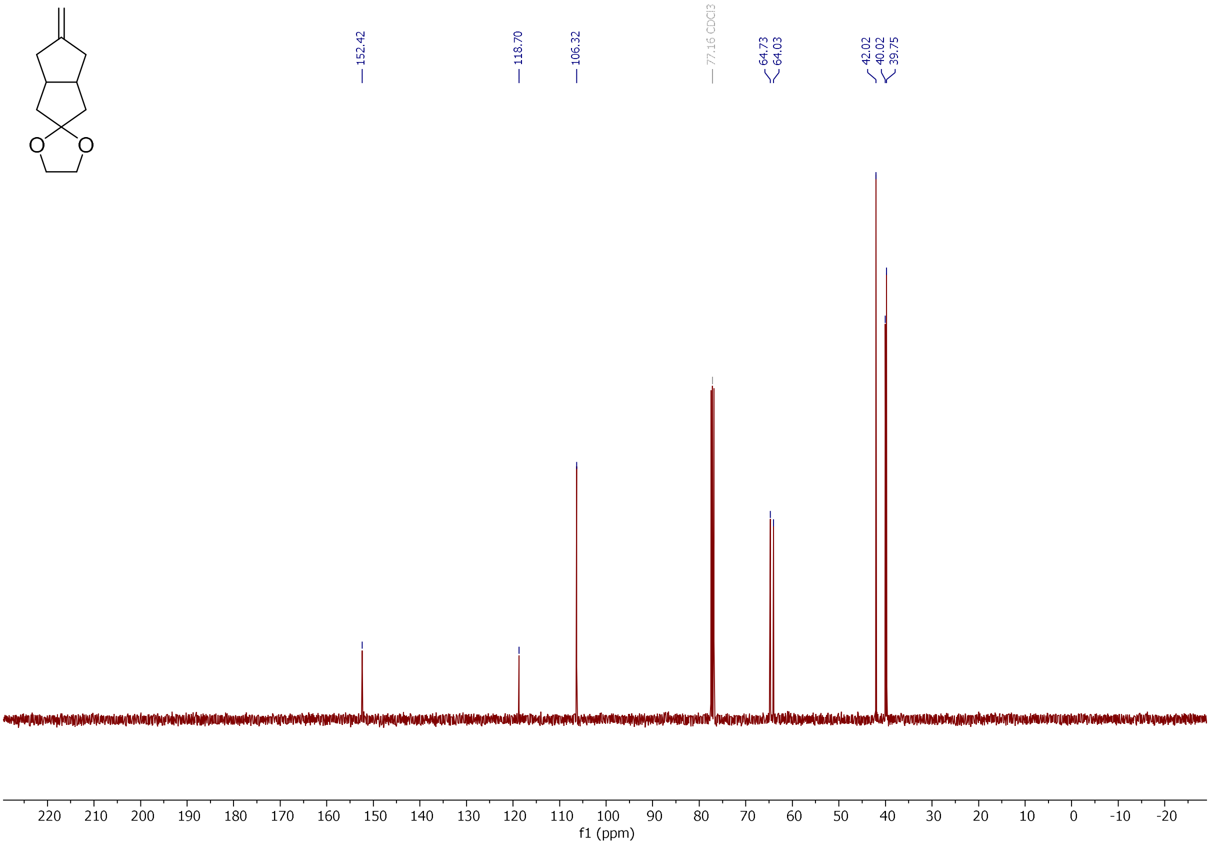

2,2,2-Trifluoro-1-(4-methylenepiperidin-1-yl)ethan-1-one (O-35)

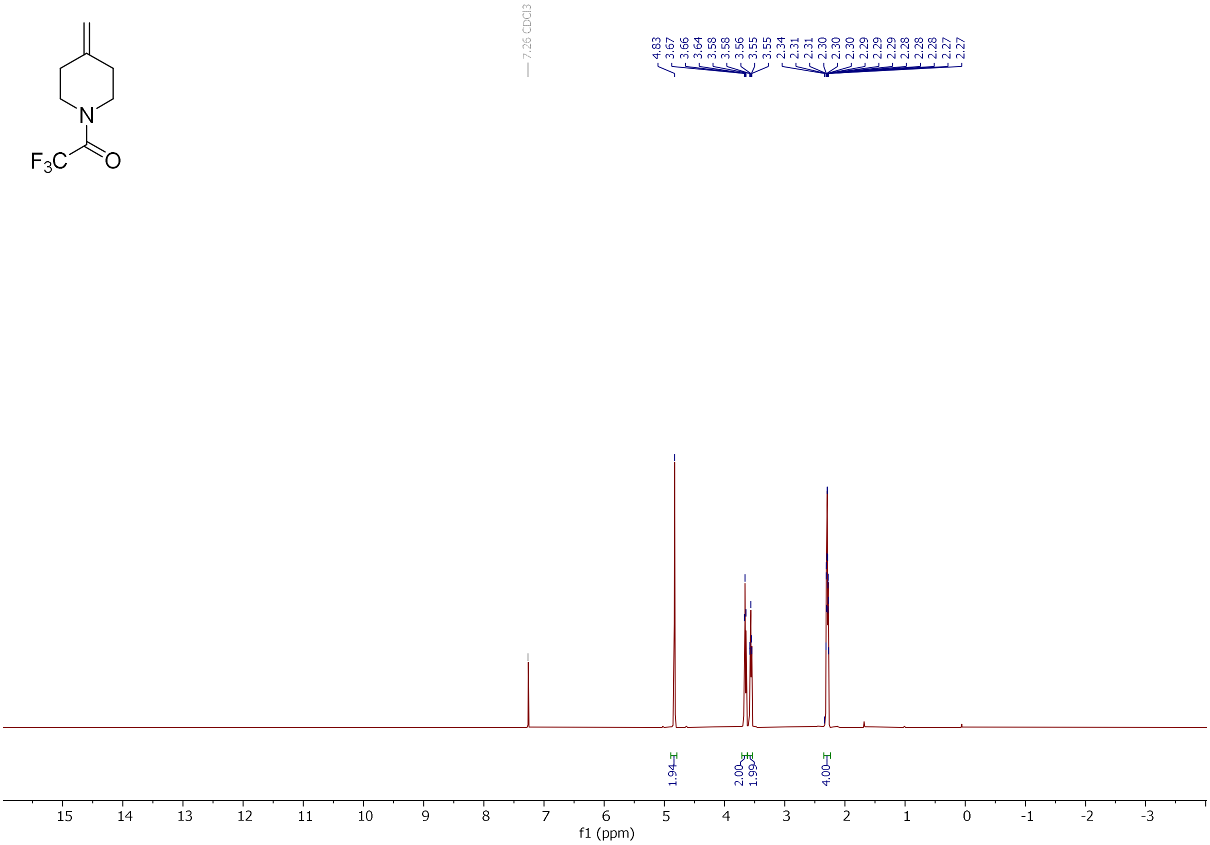

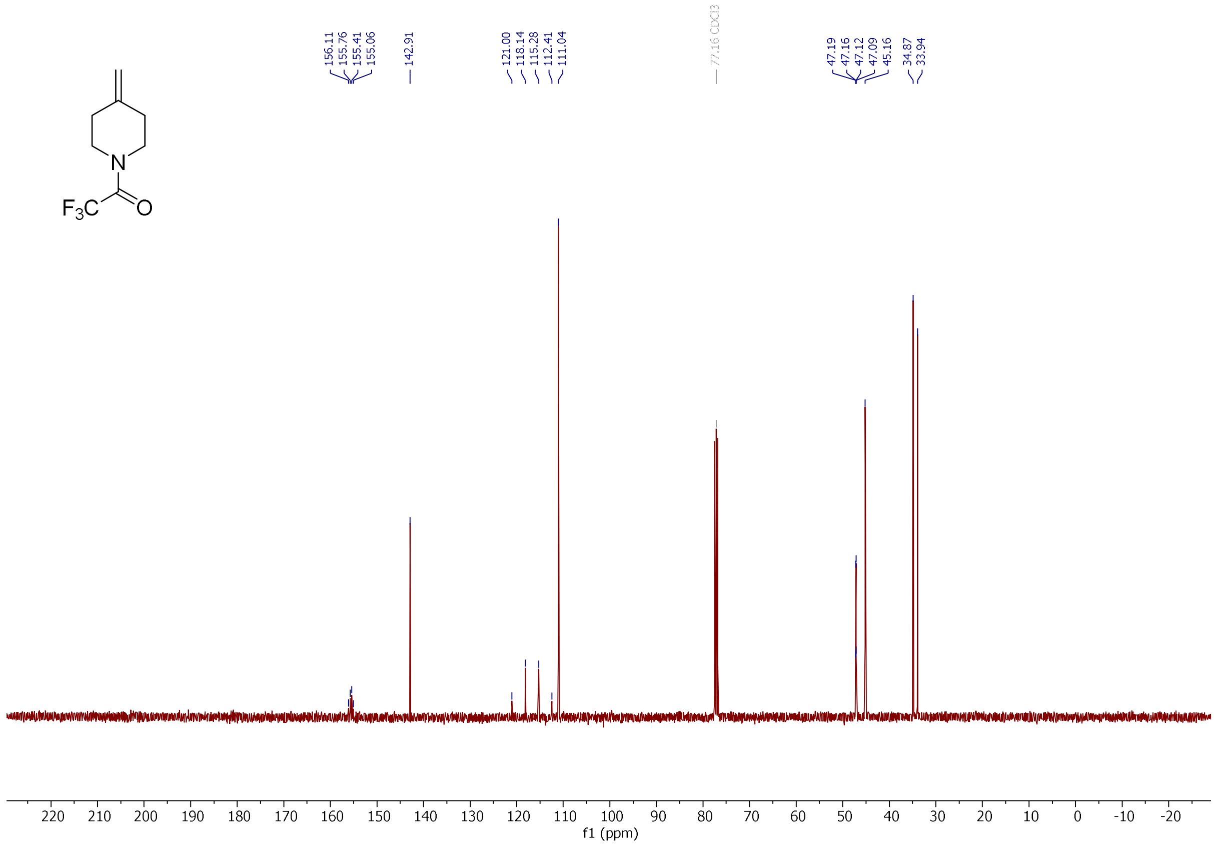
156.11
155.76
155.41
155.06
142.91
121.00
118.14
115.28
112.41
111.04
77.16 CDCl3
47.19
47.16
47.12
47.09
45.16
34.87
33.94
F3C
N
O
f1 (ppm)

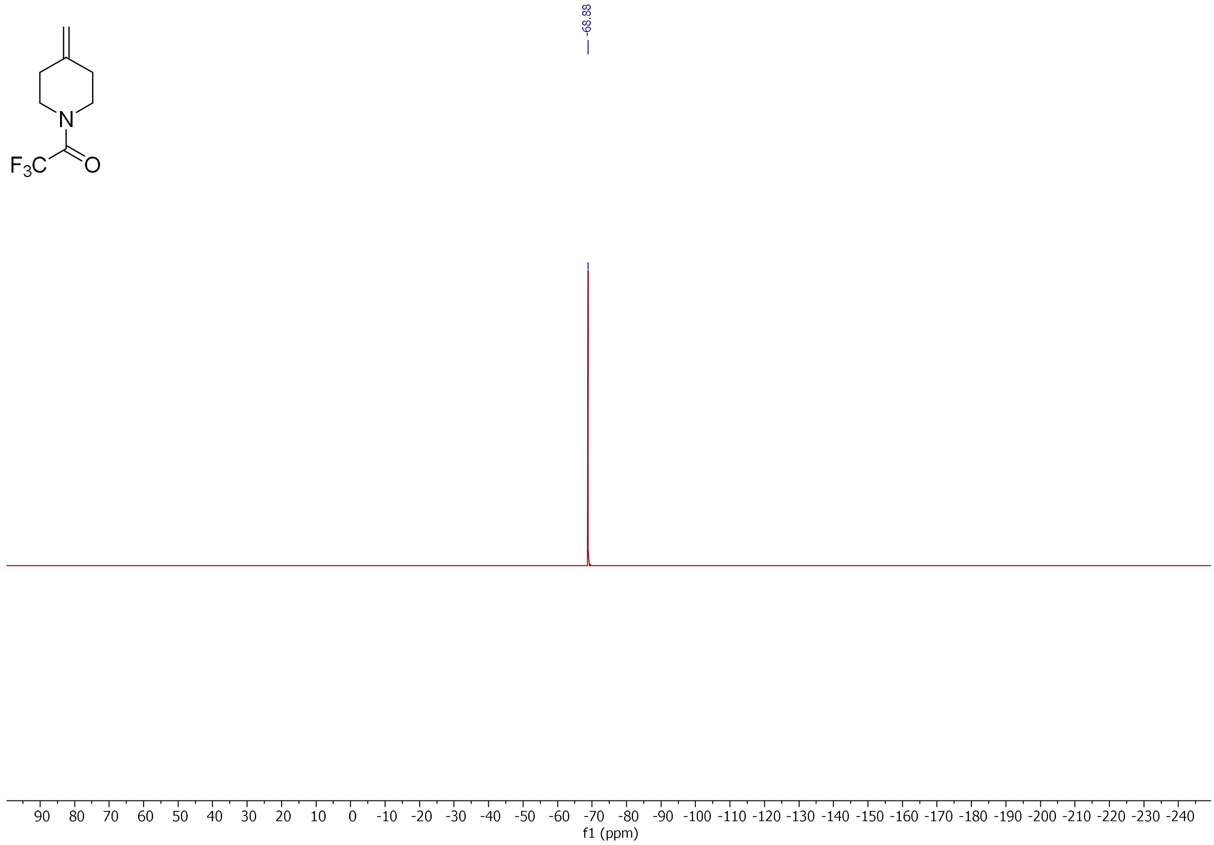
-68.88
F3C
N
O
f1 (ppm)

Tetrahydro-4H-thiopyran-4-one 1,1-dioxide (O-36)

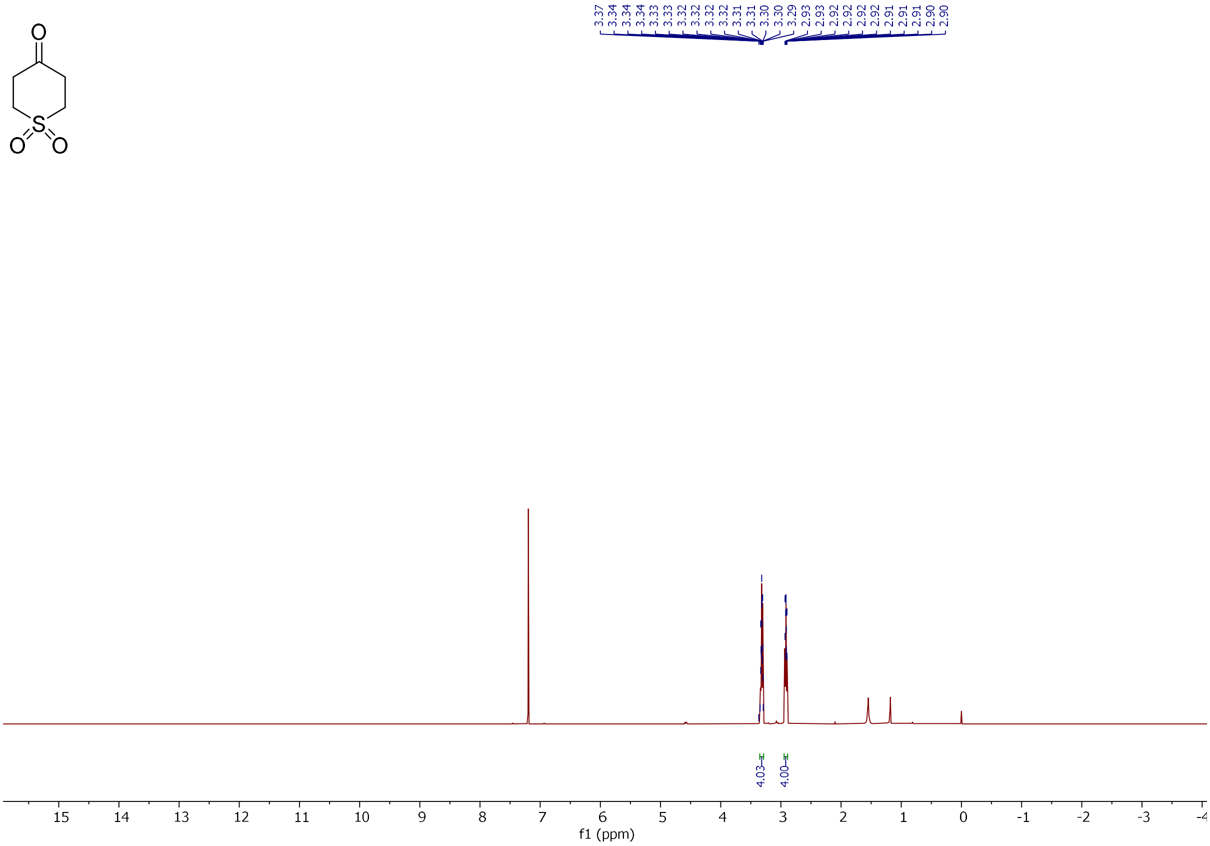

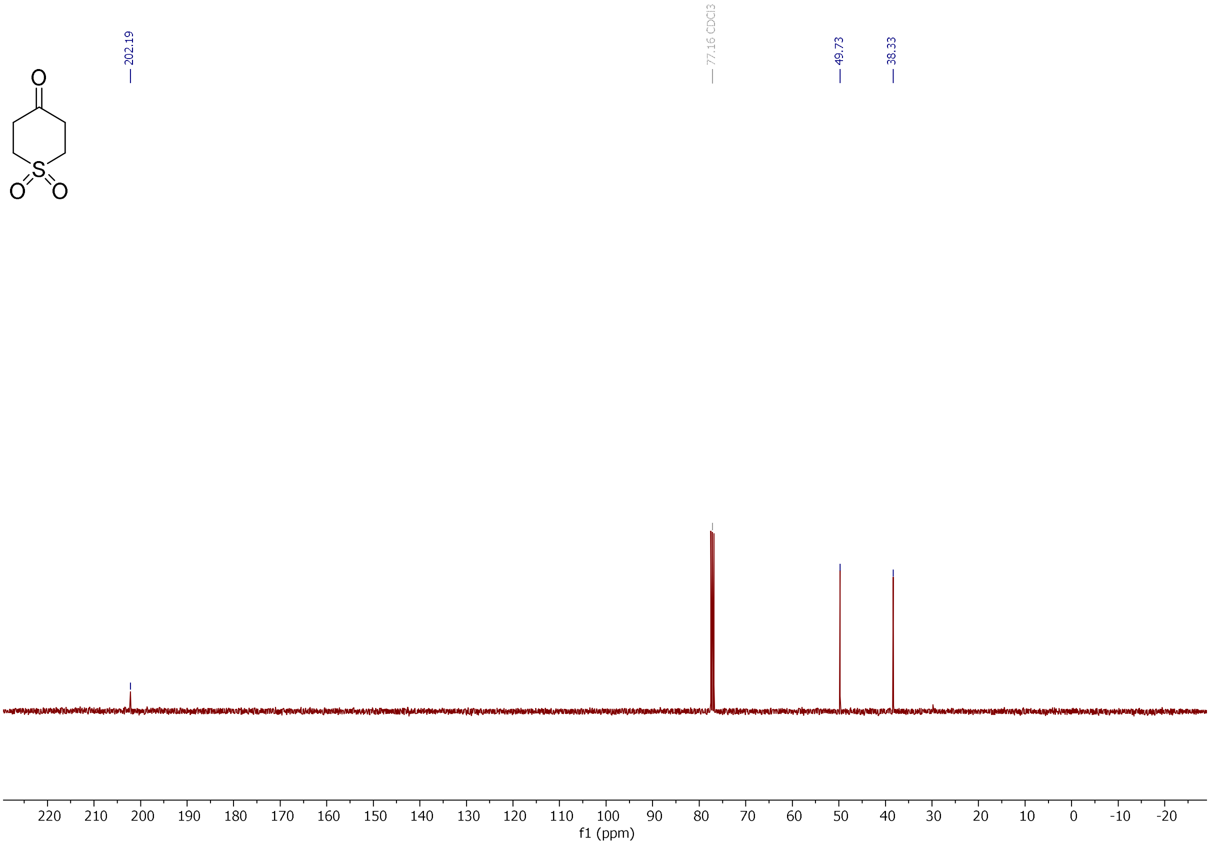

4-Methylenetetrahydro-2H-thiopyran 1,1-dioxide (O-37)

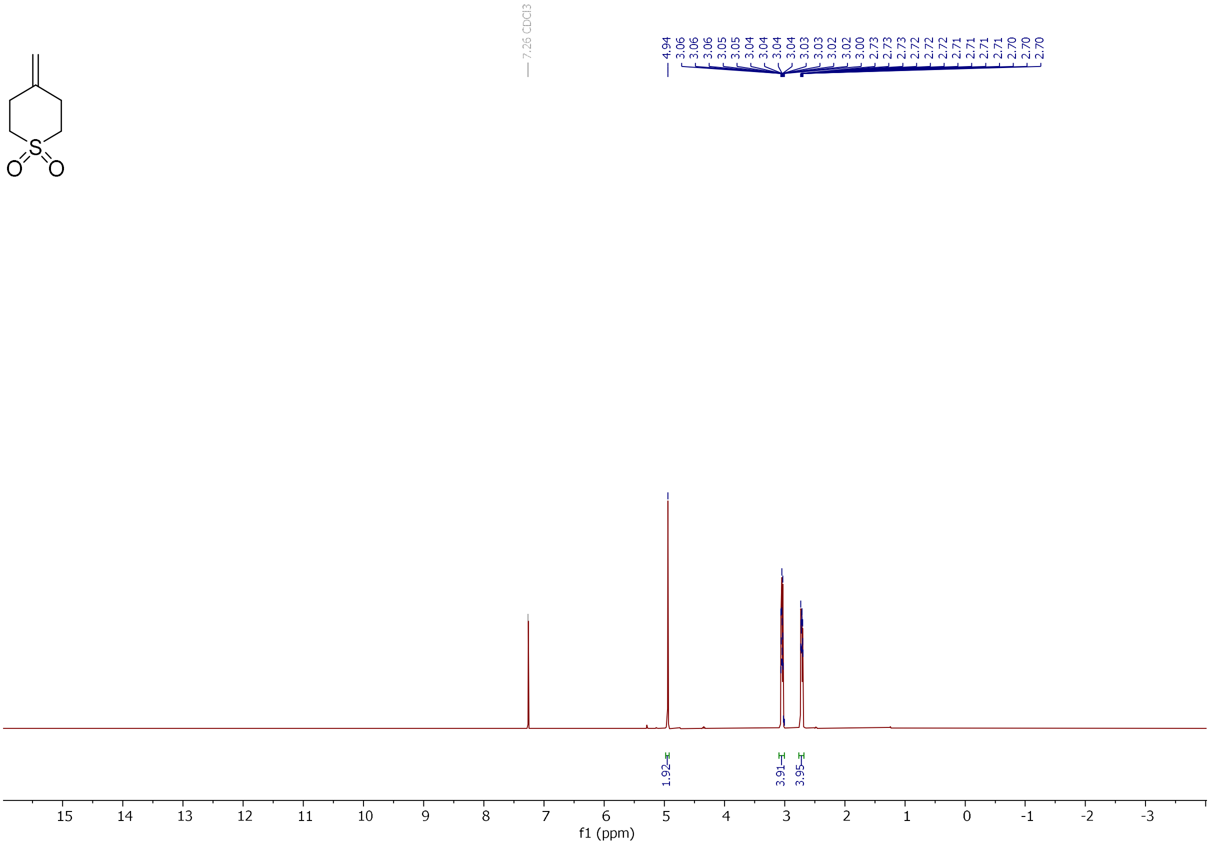

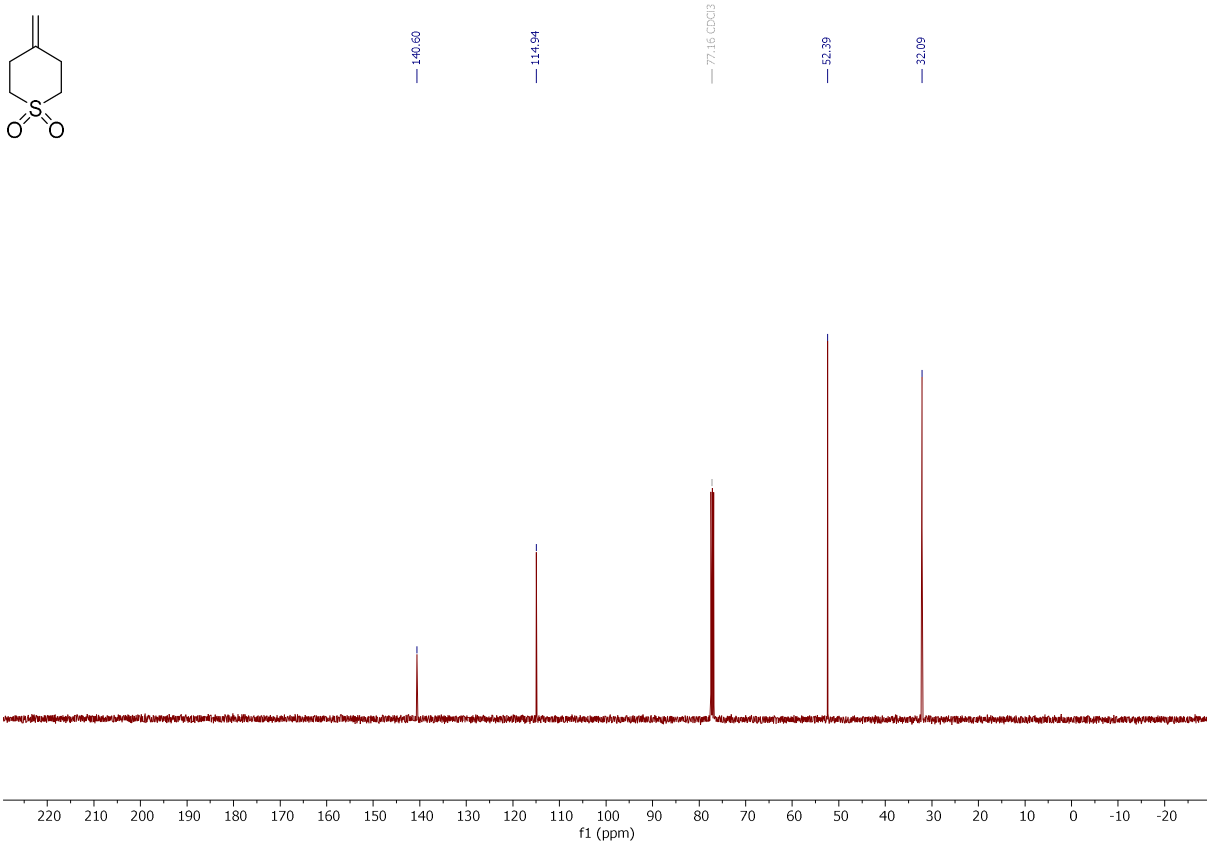

Tert-butyl (5-bromopyridin-2-yl)(tert-butoxycarbonyl)carbamate (O-38)

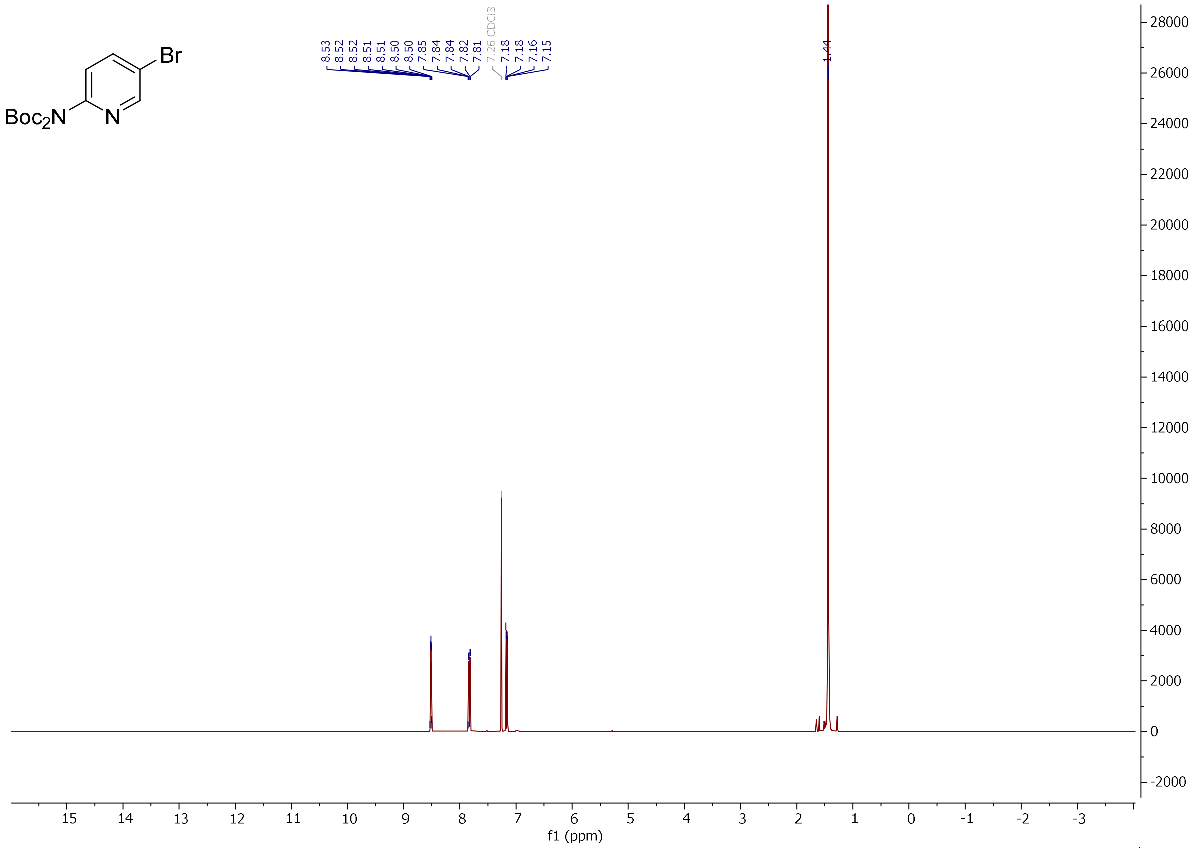

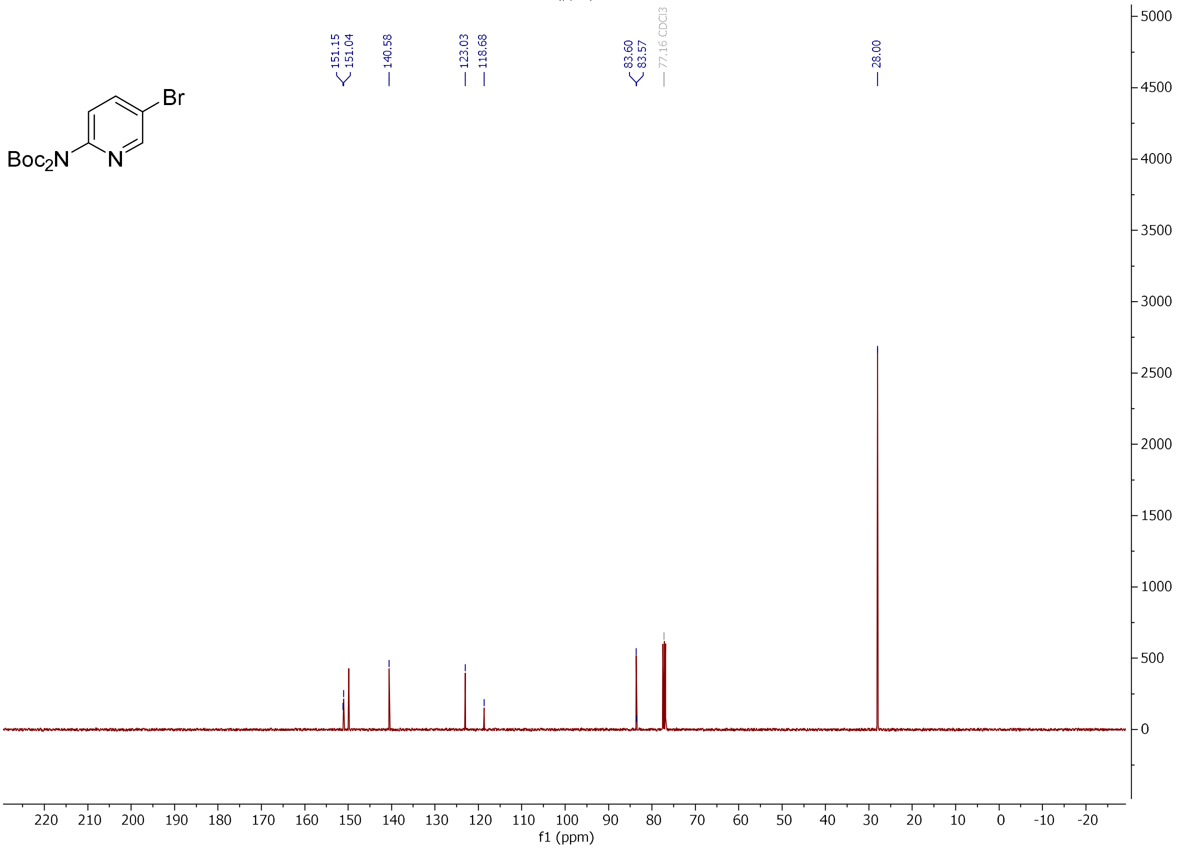

Tert-butyl (tert-butoxycarbonyl)(5-vinylpyridin-2-yl)carbamate (O-39)

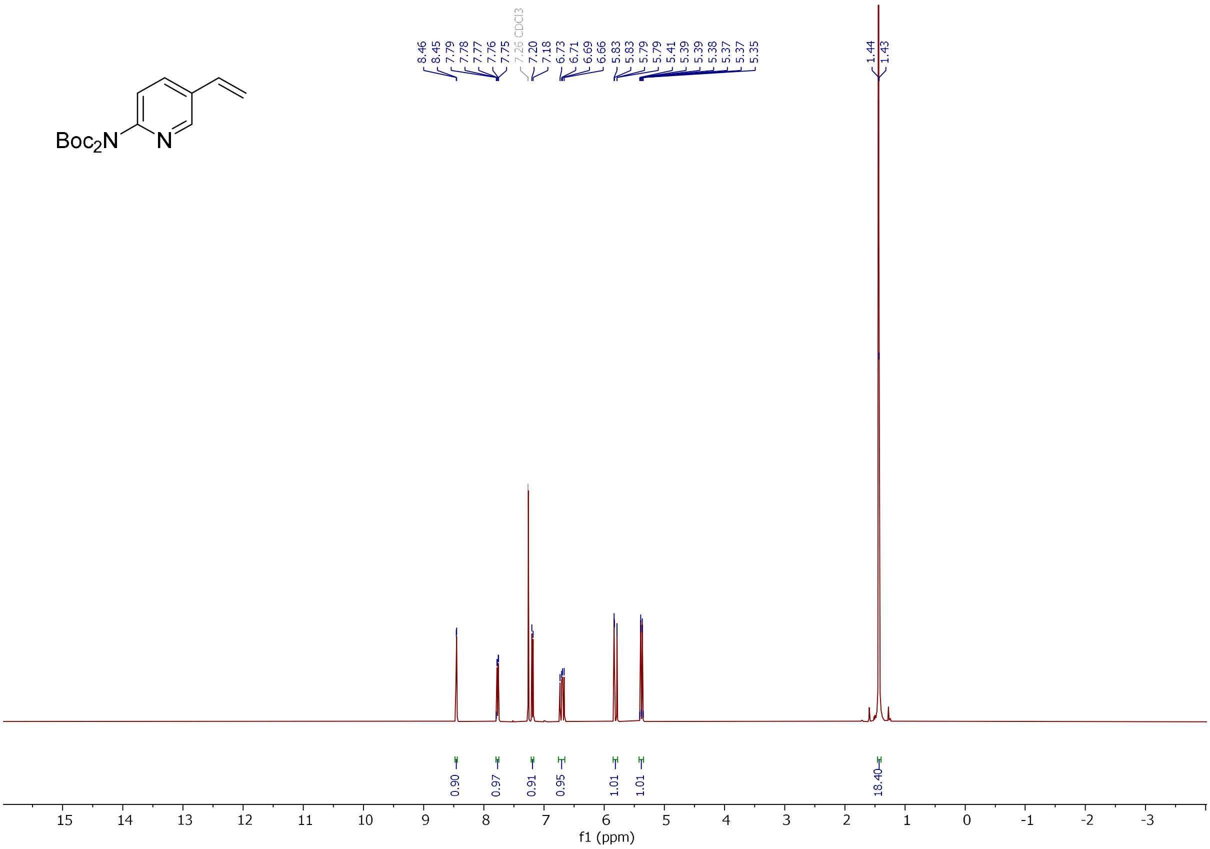

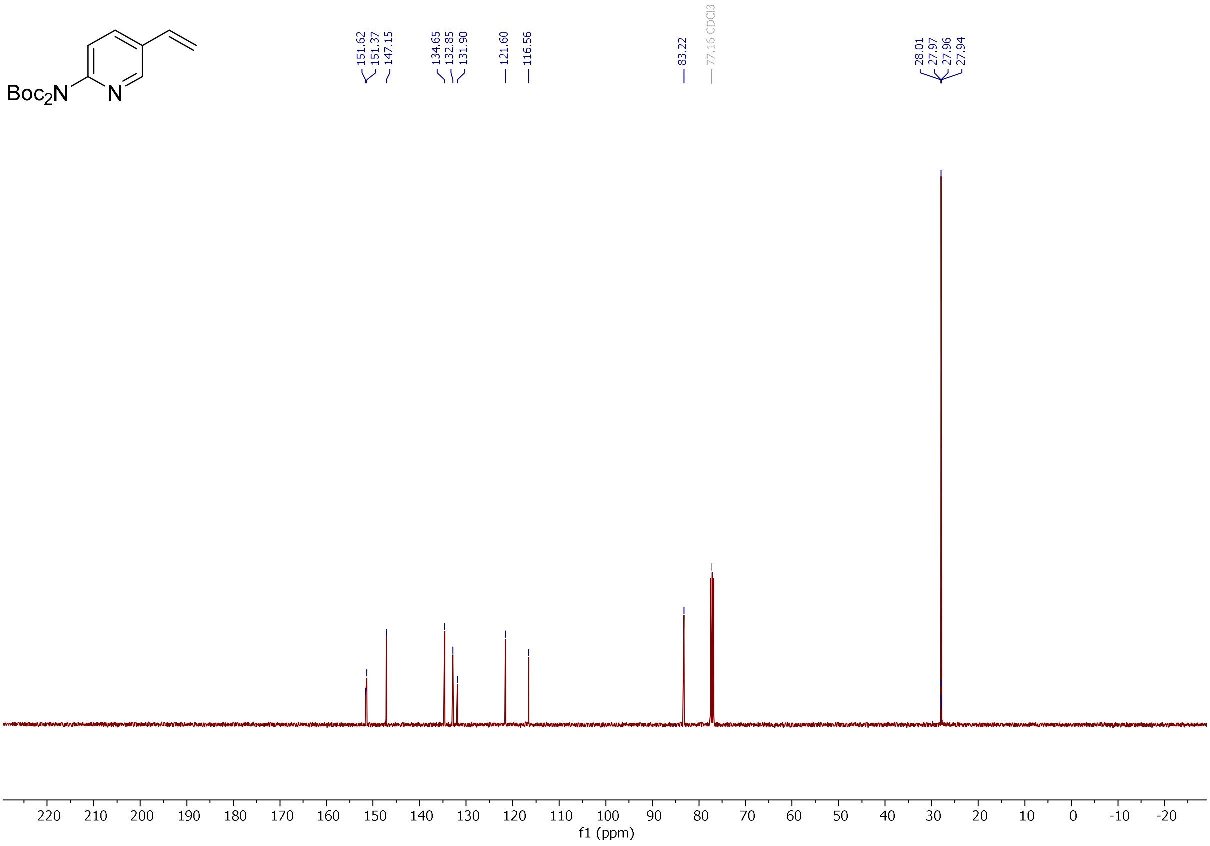

3,5-Dimethyl-1-vinyl-1H-pyrazole (O-40)

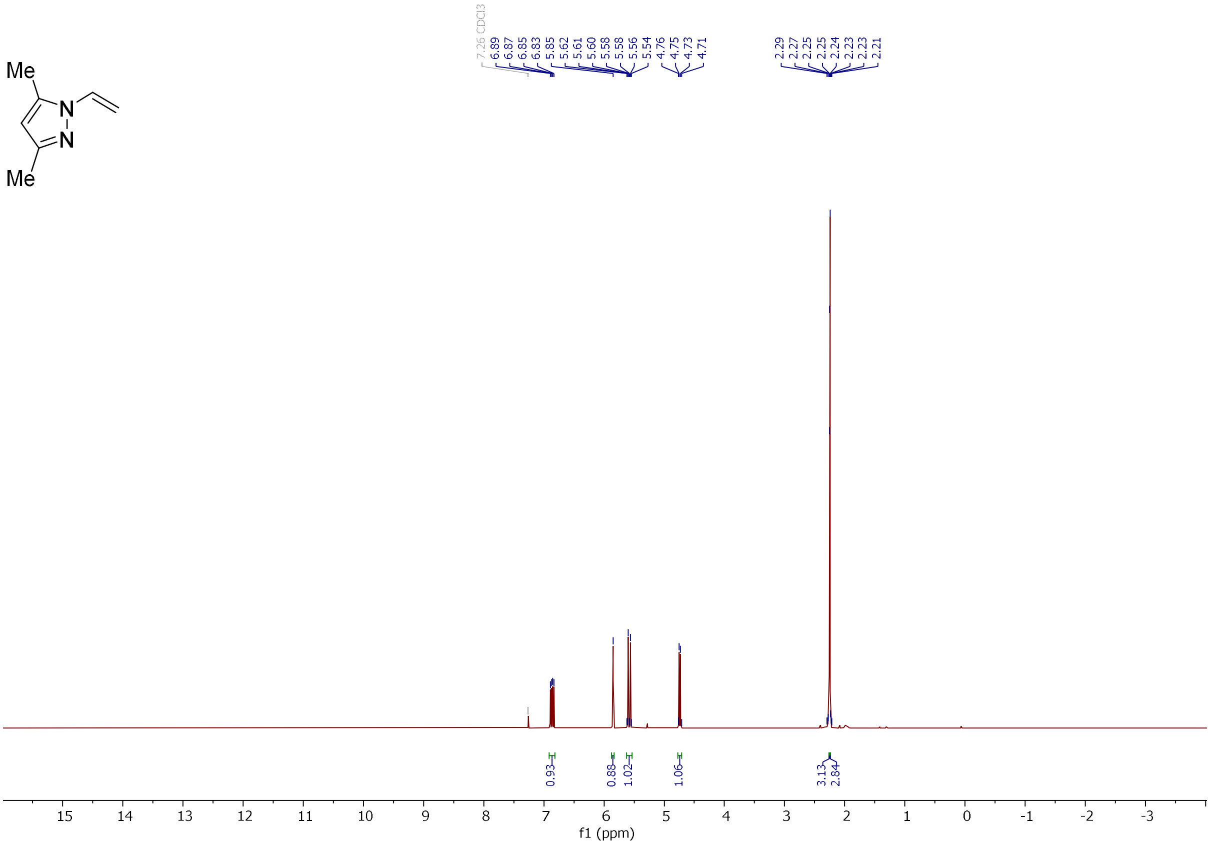

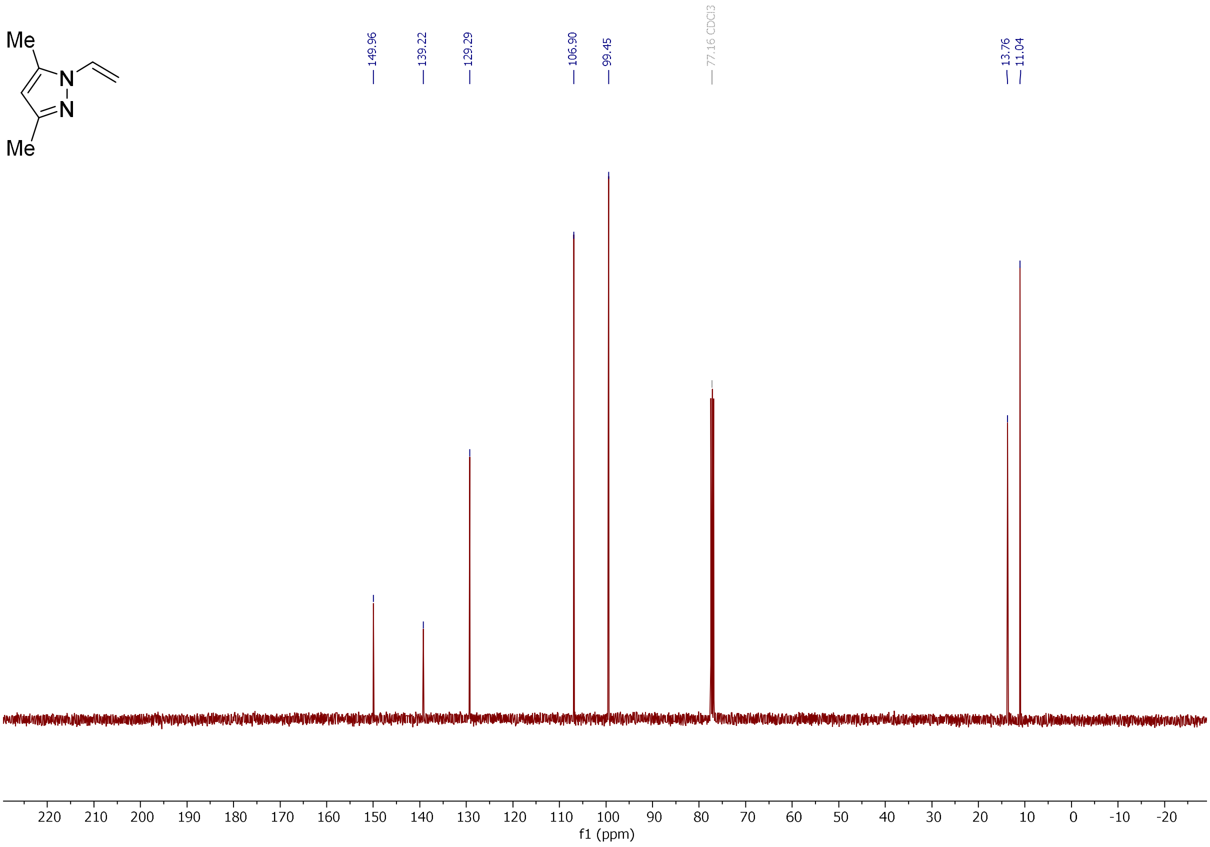

tert-butyl vinylcarbamate (O-41)

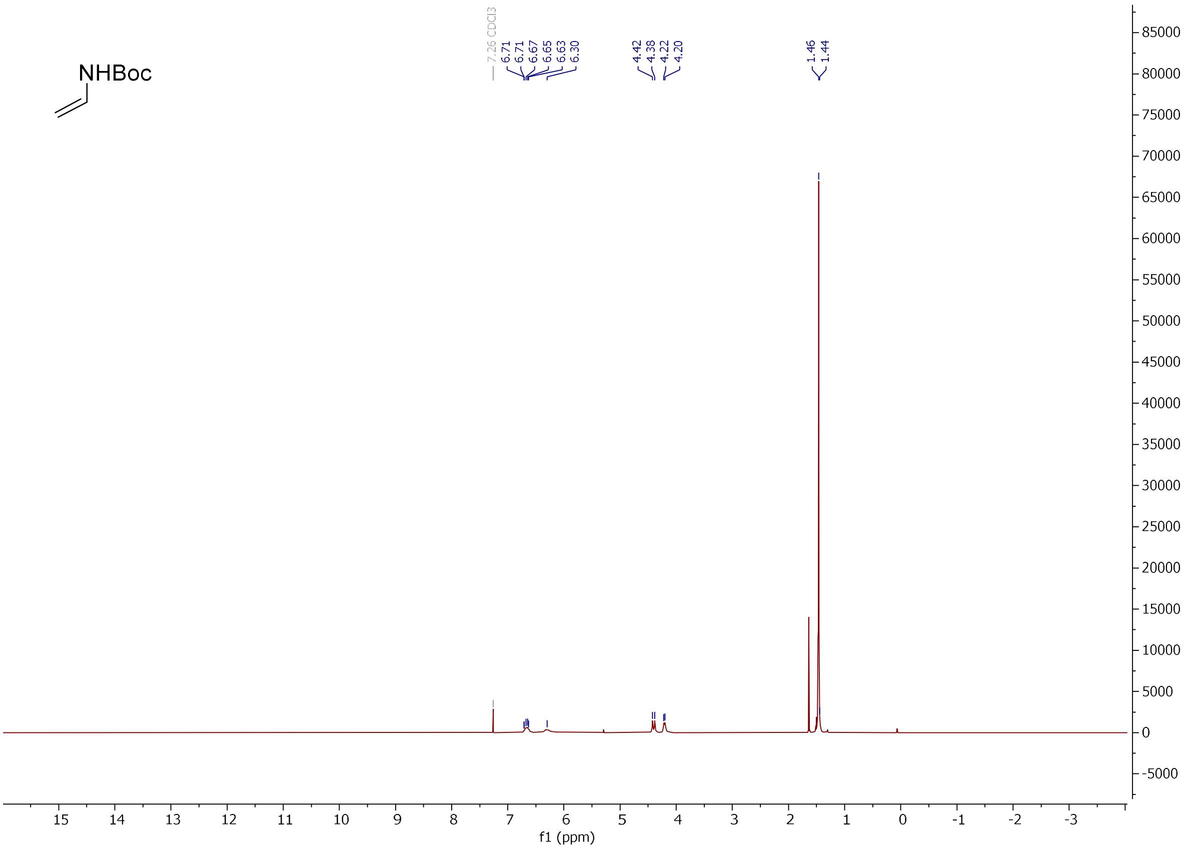

Di-tert-butyl vinyliminodicarbonate (O-42)

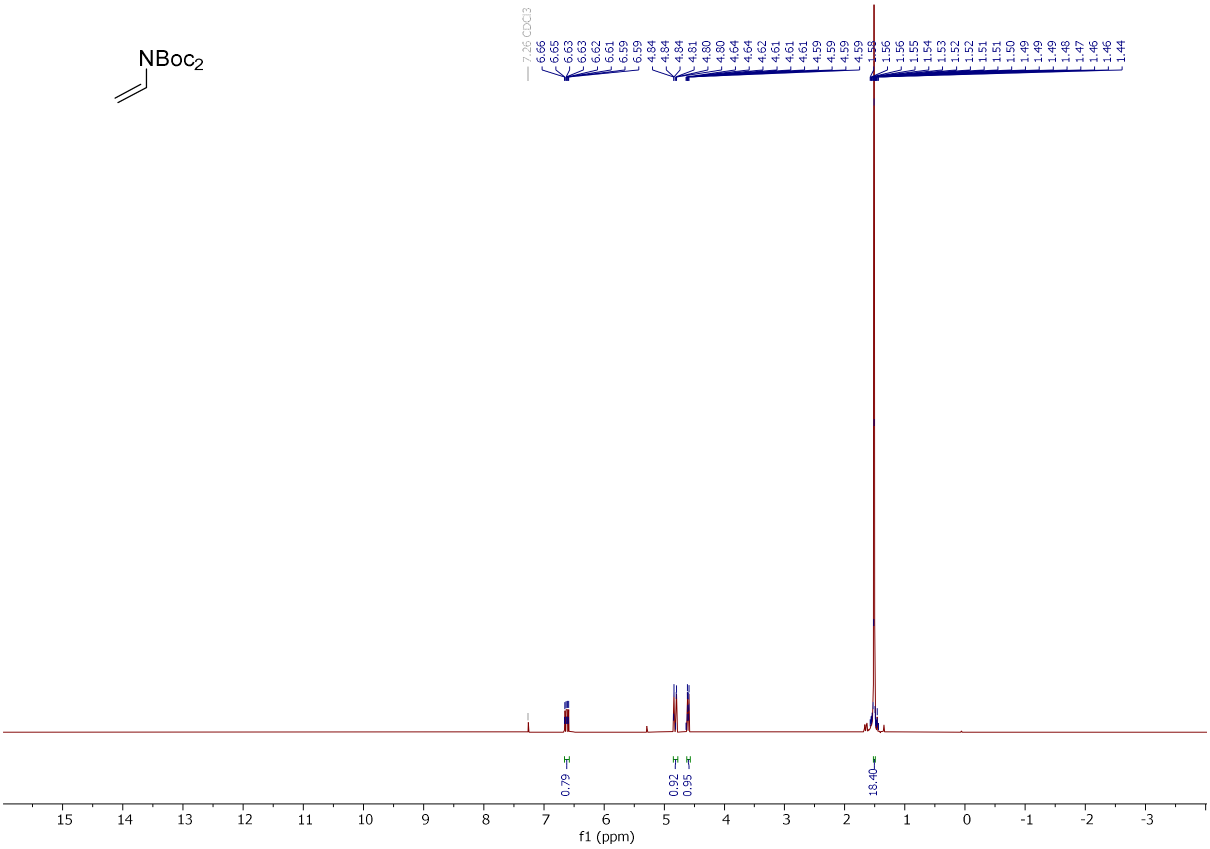

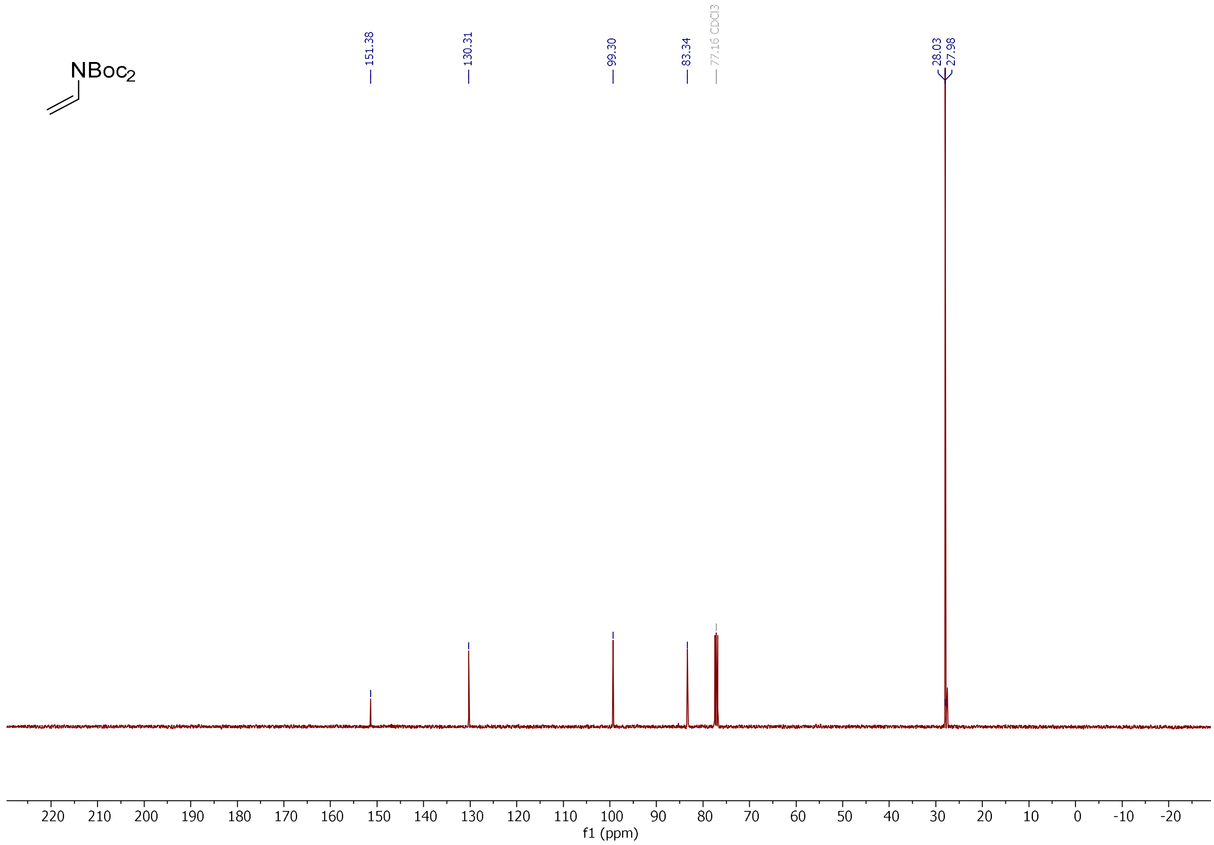

N-triflyl aldimine

1,1,1-trifluoro-N-(4-methoxybenzylidene)methanesulfonamide (I-43)

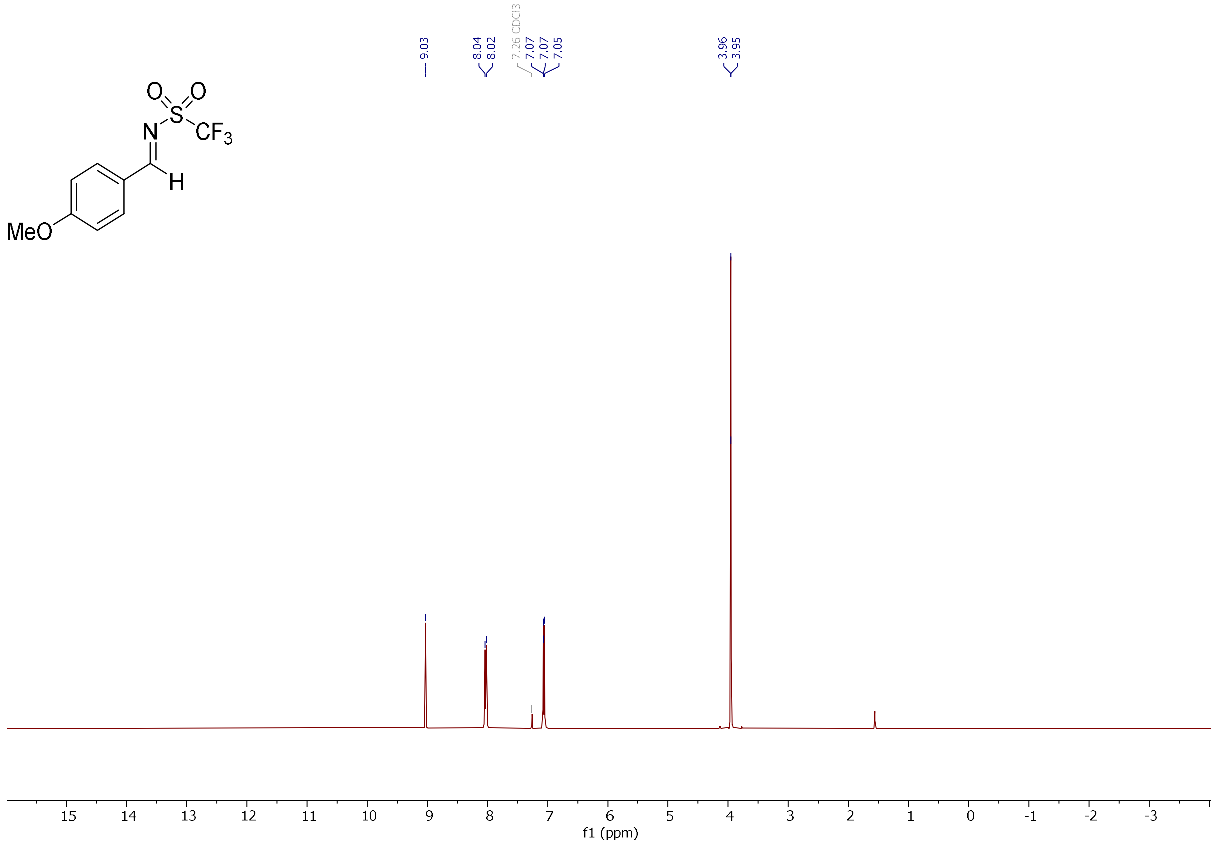

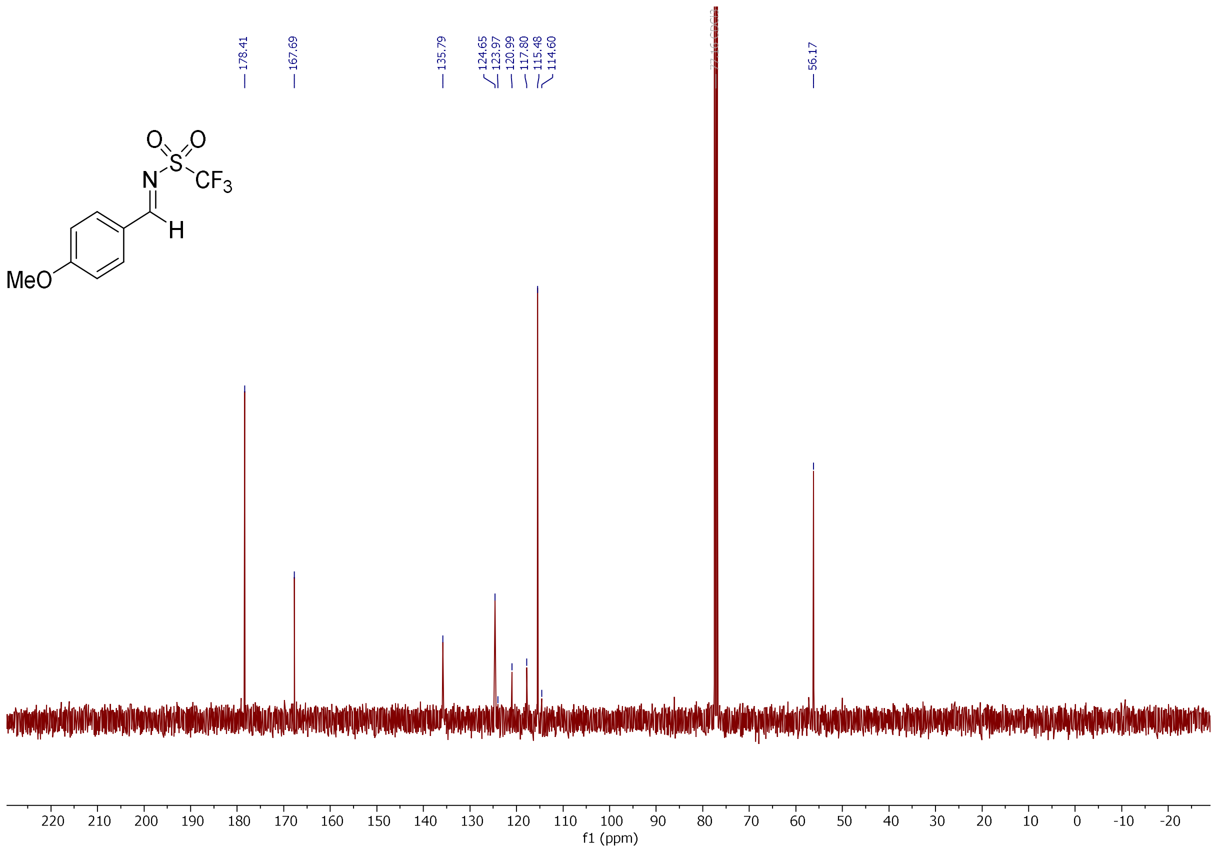
178.41
167.69
135.79
124.65
123.97
120.99
117.80
115.48
114.60
56.17
MeO
N
S
O
O
CF3
H
220 210 200 190 180 170 160 150 140 130 120 110 100 90 80 70 60 50 40 30 20 10 0 -10 -20
f1 (ppm)

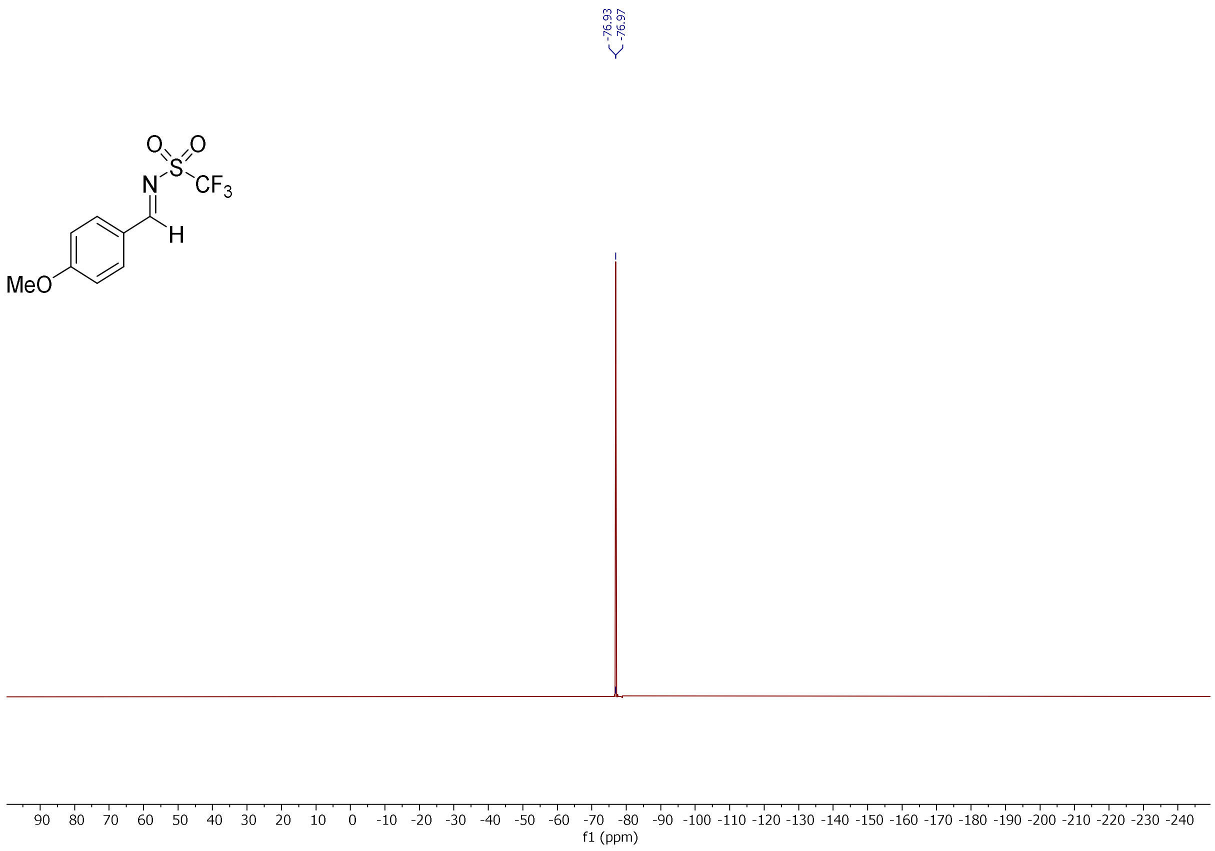
-76.93
-76.97
MeO
N
S
O
O
CF3
H
90 80 70 60 50 40 30 20 10 0 -10 -20 -30 -40 -50 -60 -70 -80 -90 -100 -110 -120 -130 -140 -150 -160 -170 -180 -190 -200 -210 -220 -230 -240
f1 (ppm)

Supplementary References

1 Juliá, F., Yan, J., Paulus, F. & Ritter, T. Vinyl Thianthrenium Tetrafluoroborate: A Practical and Versatile Vinylating Reagent Made from Ethylene. *J. Am. Chem. Soc.* **143**, 12992-12998 (2021). https://doi.org/10.1021/jacs.1c06632

2 Elliott, L. D., Kayal, S., George, M. W. & Booker-Milburn, K. Rational Design of Triplet Sensitizers for the Transfer of Excited State Photochemistry from UV to Visible. *J. Am. Chem. Soc.* **142**, 14947-14956 (2020). https://doi.org/10.1021/jacs.0c05069

3 Rubio-Presa, R., Suarez-Pantiga, S., Pedrosa, M. R. & Sanz, R. Molybdenum-Catalyzed Sustainable Friedländer Synthesis of Quinolines. *Adv. Synth. Catal.* **360**, 2216 - 2220 (2018). https://doi.org/10.1002/adsc.201800278

4 Neese, F. Software update: The ORCA program system—Version 5.0. *Wiley Interdiscip. Rev. Comput. Mol. Sci.* **12**, e1606 (2022). https://doi.org/https://doi.org/10.1002/wcms.1606

5 Neese, F. The ORCA program system. *Wiley Interdiscip. Rev. Comput. Mol. Sci.* **2**, 73-78 (2012). https://doi.org/https://doi.org/10.1002/wcms.81

6 Helmich-Paris, B., de Souza, B., Neese, F. & Izsák, R. An improved chain of spheres for exchange algorithm. *J. Chem. Phys.* **155** (2021). https://doi.org/10.1063/5.0058766

7 Izsák, R. & Neese, F. An overlap fitted chain of spheres exchange method. *J. Chem. Phys.* **135** (2011). https://doi.org/10.1063/1.3646921

8 Izsák, R., Neese, F. & Klopper, W. Robust fitting techniques in the chain of spheres approximation to the Fock exchange: The role of the complementary space. *J. Chem. Phys.* **139** (2013). https://doi.org/10.1063/1.4819264

9 Neese, F., Wennmohs, F., Hansen, A. & Becker, U. Efficient, approximate and parallel Hartree–Fock and hybrid DFT calculations. A 'chain-of-spheres' algorithm for the Hartree–Fock exchange. *Chem. Phys.* **356**, 98-109 (2009). https://doi.org/https://doi.org/10.1016/j.chemphys.2008.10.036

10 Marenich, A. V., Cramer, C. J. & Truhlar, D. G. Universal Solvation Model Based on Solute Electron Density and on a Continuum Model of the Solvent Defined by the Bulk Dielectric Constant and Atomic Surface Tensions. *J. Phys. Chem. B* **113**, 6378-6396 (2009). https://doi.org/10.1021/jp810292n

11 Zhao, Y. & Truhlar, D. G. The M06 suite of density functionals for main group thermochemistry, thermochemical kinetics, noncovalent interactions, excited states, and transition elements: two new functionals and systematic testing of four M06-class functionals and 12 other functionals. *Theor. Chem. Acc.* **120**, 215-241 (2008). https://doi.org/10.1007/s00214-007-0310-x

12 Weigend, F. & Ahlrichs, R. Balanced basis sets of split valence, triple zeta valence and quadruple zeta valence quality for H to Rn: Design and assessment of accuracy. *Phys. Chem. Chem. Phys.* **7**, 3297-3305 (2005).

13 Grimme, S., Antony, J., Ehrlich, S. & Krieg, H. A consistent and accurate ab initio parametrization of density functional dispersion correction (DFT-D) for the 94 elements H-Pu. *J. Chem. Phys.* **132**, 154104 (2010). https://doi.org/10.1063/1.3382344

14 Grimme, S., Ehrlich, S. & Goerigk, L. Effect of the damping function in dispersion corrected density functional theory. *J. Comput. Chem.* **32**, 1456-1465 (2011). https://doi.org/https://doi.org/10.1002/jcc.21759

15 Goerigk, L. & Grimme, S. A thorough benchmark of density functional methods for general main group thermochemistry, kinetics, and noncovalent interactions. *Phys. Chem. Chem. Phys.* **13**, 6670-6688 (2011). https://doi.org/10.1039/C0CP02984J

16 Goerigk, L. *et al.* A look at the density functional theory zoo with the advanced GMTKN55 database for general main group thermochemistry, kinetics and noncovalent interactions. *Phys. Chem. Chem. Phys.* **19**, 32184-32215 (2017). https://doi.org/10.1039/C7CP04913G

17 Korth, M. & Grimme, S. "Mindless" DFT Benchmarking. *J. Chem. Theory Comput.* **5**, 993-1003 (2009). https://doi.org/10.1021/ct800511q

18 Ishida, K., Morokuma, K. & Komornicki, A. The intrinsic reaction coordinate. An ab initio calculation for HNC→HCN and H−+CH4→CH4+H− *J. Chem. Phys.* **66**, 2153-2156 (1977). https://doi.org/10.1063/1.434152
19 Harvey, J. N., Aschi, M., Schwarz, H. & Koch, W. The singlet and triplet states of phenyl cation. A hybrid approach for locating minimum energy crossing points between non-interacting potential energy surfaces. *Theor. Chem. Acc.* **99**, 95-99 (1998). https://doi.org/10.1007/s002140050309
20 Yamaguchi, K., Jensen, F., Dorigo, A. & Houk, K. A spin correction procedure for unrestricted Hartree-Fock and Møller-Plesset wavefunctions for singlet diradicals and polyradicals. *Chem. Phys. Lett.* **149**, 537-542 (1988).
21 Martyna, G. J., Klein, M. L. & Tuckerman, M. Nosé–Hoover chains: The canonical ensemble via continuous dynamics. *J. Chem. Phys.* **97**, 2635-2643 (1992). https://doi.org/10.1063/1.463940
22 Martyna, G. J., Tuckerman, M. E., Tobias, D. J. & Klein, M. L. Explicit reversible integrators for extended systems dynamics. *Mol. Phys.* **87**, 1117-1157 (1996). https://doi.org/10.1080/00268979600100761
23 Bouayad-Gervais, S. *et al.* Access to Cyclic N-Trifluoromethyl Ureas through Photocatalytic Activation of Carbamoyl Azides. *J. Am. Chem. Soc.* **144**, 6100-6106 (2022). https://doi.org/10.1021/jacs.2c02004
24 NBO Version 3.1.
25 Gaussian 16 Rev. C.01 (Wallingford, CT, 2016).
26 CYLview20 (Université de Sherbrooke, 2020).
27 Li, Y., Wang, W. & Liu, F. Exploring the Mechanism of a Chiral N-Alkyl Imine-Based Light-Driven Molecular Rotary Motor at MS-CASPT2//CASSCF and MS-CASPT2//(TD) DFT Levels. *Chem. Eur. J.* **25**, 4194-4201 (2019). https://doi.org/https://doi.org/10.1002/chem.201806152
28 Uraguchi, D. *et al.* Unveiling Latent Photoreactivity of Imines. *Angew. Chem. Int. Ed.* **59**, 3665-3670 (2020). https://doi.org/https://doi.org/10.1002/anie.201913555
29 Tilby, M. J. *et al.* Photocatalytic Late-Stage Functionalization of Sulfonamides via Sulfonyl Radical Intermediates. *ACS Catal.* **12**, 6060-6067 (2022). https://doi.org/10.1021/acscatal.2c01442
30 Jung, H., Keum, H., Kweon, J. & Chang, S. Tuning Triplet Energy Transfer of Hydroxamates as the Nitrene Precursor for Intramolecular C(sp3)–H Amidation. *J. Am. Chem. Soc.* **142**, 5811-5818 (2020). https://doi.org/10.1021/jacs.0c00868
31 Baas, P. & Cerfontain, H. Photochemistry of α-oxo-oximes. Part 1. Photoisomerization of biacetyl mono-oxime ethyl ether. *J. Chem. Soc., Perkin Trans. 2*, 1351-1353 (1977). https://doi.org/10.1039/P29770001351
32 Chakraborty, D. & Chattaraj, P. K. Conceptual density functional theory based electronic structure principles. *Chem. Sci.* **12**, 6264-6279 (2021). https://doi.org/10.1039/D0SC07017C
33 Zhang, Y., Xue, J., Gao, Y., Fun, H.-K. & Xu, J.-H. Photoinduced [2+2] cycloadditions (the Paterno–Büchi reaction) of 1-acetylisatin with enol ethers—regioselectivity, diastereoselectivity and acid catalysed transformations of the spirooxetane products. *J. Chem. Soc., Perkin Trans. 1*, 345-353 (2002).
34 Franceschi, P., Cuadros, S., Goti, G. & Dell'Amico, L. Mechanisms and Synthetic Strategies in Visible Light-Driven [2+2]-Heterocycloadditions. *Angew. Chem. Int. Ed.* **62**, e202217210 (2023). https://doi.org/https://doi.org/10.1002/anie.202217210
35 Isegawa, M., Neese, F. & Pantazis, D. A. Ionization Energies and Aqueous Redox Potentials of Organic Molecules: Comparison of DFT, Correlated ab Initio Theory and Pair Natural Orbital Approaches. *J. Chem. Theory Comput.* **12**, 2272-2284 (2016). https://doi.org/10.1021/acs.jctc.6b00252
36 Ho, J. Are thermodynamic cycles necessary for continuum solvent calculation of pKas and reduction potentials? *Phys. Chem. Chem. Phys.* **17**, 2859-2868 (2015). https://doi.org/10.1039/C4CP04538F

37 Demissie, T. B., Ruud, K. & Hansen, J. H. DFT as a Powerful Predictive Tool in Photoredox Catalysis: Redox Potentials and Mechanistic Analysis. *Organometallics* **34**, 4218-4228 (2015). https://doi.org/10.1021/acs.organomet.5b00582
38 Roesky, H. W. & Tutkunkardes, S. Fluorsulfonylstickstoffverbindungen. *Z. Anorg. Allg. Chem.* **374**, 147-158 (1970). https://doi.org/https://doi.org/10.1002/zaac.19703740204
39 Clauβ, K., Friedrich, H.-J. & Jensen, H. Reaktionen der Aldehyde und Ketone mit Chlor- und Fluorsulfonylisocyanat. *Justus Liebigs Ann. Chem.* **1974**, 561-592 (1974). https://doi.org/https://doi.org/10.1002/jlac.197419740404
40 Barth, E. R. *et al.* Higher Carbon Analogues of 1,4-Dihydropyridines as Potent TGFβ/Smad Inhibitors. *Eur. J. Inorg. Chem.* **2020**, 176-181 (2020). https://doi.org/https://doi.org/10.1002/ejic.201901223
41 Ganguly, A., Chandrasekaran, R., Balamurugan, B. S. S. & Rasappan, R. Application of Solid Me3SiZnI for the Synthesis of Aryl and Alkyl Trimethylsilanes. *Adv. Synth. Catal.* **366**, 1442-1447 (2024). https://doi.org/https://doi.org/10.1002/adsc.202301298
42 Lindsey, J. S., Brown, P. A. & Siesel, D. A. Visible light-harvesting in covalently-linked porphyrin-cyanine dyes. *Tetrahedron* **45**, 4845-4866 (1989). https://doi.org/https://doi.org/10.1016/S0040-4020(01)85156-5
43 Díaz-Oviedo, C. D., Maji, R. & List, B. The Catalytic Asymmetric Intermolecular Prins Reaction. *J. Am. Chem. Soc.* **143**, 20598-20604 (2021). https://doi.org/10.1021/jacs.1c10245
44 Oka, N., Yamada, T., Sajiki, H., Akai, S. & Ikawa, T. Aryl Boronic Esters Are Stable on Silica Gel and Reactive under Suzuki–Miyaura Coupling Conditions. *Org. Lett.* **24**, 3510-3514 (2022). https://doi.org/10.1021/acs.orglett.2c01174
45 Xi, J. & Gu, Z. Palladium-Catalzyed Atroposelective 16-Membered Macrocyclization: Total Synthesis of Isoplagiochin D†. *Chin. J. Chem* . **38**, 1081-1085 (2020). https://doi.org/https://doi.org/10.1002/cjoc.202000051
46 Hameed P, S. *et al.* Novel N-Linked Aminopiperidine-Based Gyrase Inhibitors with Improved hERG and in Vivo Efficacy against Mycobacterium tuberculosis. *J. Med. Chem.* **57**, 4889-4905 (2014). https://doi.org/10.1021/jm500432n
47 Huang, H. *et al.* Synthesis of Aldehydes by Organocatalytic Formylation Reactions of Boronic Acids with Glyoxylic Acid. *Angew. Chem. Int. Ed.* **56**, 8201-8205 (2017). https://doi.org/https://doi.org/10.1002/anie.201703127
48 Ghosh, A. K. & Nicponski, D. R. Cu(II)-Catalyzed Olefin Migration and Prins Cyclization: Highly Diastereoselective Synthesis of Substituted Tetrahydropyrans. *Org. Lett.* **13**, 4328-4331 (2011). https://doi.org/10.1021/ol2016675
49 Bewley, C. A., Ray, S., Cohen, F., Collins, S. K. & Overman, L. E. Inhibition of HIV-1 Envelope-Mediated Fusion by Synthetic Batzelladine Analogues. *J. Nat. Prod.* **67**, 1319-1324 (2004). https://doi.org/10.1021/np049958o
50 Ellwood, A. R. & Porter, M. J. A direct and efficient preparation of 1-phenyltetrazol-5-yl sulfides from alcohols. *Org. Biomol. Chem.* **9**, 379-381 (2011). https://doi.org/10.1039/C0OB00863J
51 Trost, B. M., Amans, D., Seganish, W. M. & Chung, C. K. Evaluating Transition-Metal-Catalyzed Transformations for the Synthesis of Laulimalide. *J. Am. Chem. Soc.* **131**, 17087-17089 (2009). https://doi.org/10.1021/ja907924j
52 Bodenschatz, K., Stöckl, J., Winterer, M. & Schobert, R. A synthetic approach to 5/5/6-polycyclic tetramate macrolactams of the discodermide type. *Tetrahedron* **104**, 132113 (2022). https://doi.org/https://doi.org/10.1016/j.tet.2021.132113
53 Leonard, J. *et al.* Spiro epoxide fused cis bicyclo[3.3.0]octanes: enantioselective rearrangement and utilisation of the products in synthetic adventures. *Tetrahedron* **58**, 4681-4691 (2002). https://doi.org/https://doi.org/10.1016/S0040-4020(02)00375-7
54 COMPOUNDS AND COMPOSITIONS FOR TREATING CONDITIONS ASSOCIATED WITH STING ACTIVITY. WO2023/18781 (2023).

55 Yang, D., Yip, Y.-C., Jiao, G.-S. & Wong, M.-K. Design of Efficient Ketone Catalysts for Epoxidation by Using the Field Effect. *J. Org. Chem.* **63**, 8952-8956 (1998). https://doi.org/10.1021/jo981270r
56 Cullen, M., Bastos, C. M., Parks, D. & Munoz, B. PROTEASOME ACTIVITY ENHANCING COMPOUNDS. WO2020/6296 (2020).
57 Pinto, D. J. P. *et al.* NITROGEN CONTAINING HETEROAROMATICS AS FACTOR Xa INHIBITORS. EP946508 (2009).
58 Rodygin, K. S., Bogachenkov, A. S. & Ananikov, V. P. Vinylation of a Secondary Amine Core with Calcium Carbide for Efficient Post-Modification and Access to Polymeric Materials. *Molecules* **23**, 648 (2018).
59 Chanthamath, S., Nguyen, D. T., Shibatomi, K. & Iwasa, S. Highly Enantioselective Synthesis of Cyclopropylamine Derivatives via Ru(II)-Pheox-Catalyzed Direct Asymmetric Cyclopropanation of Vinylcarbamates. *Org. Lett.* **15**, 772-775 (2013). https://doi.org/10.1021/ol303404c
60 Morales, S., Guijarro, F. G., García Ruano, J. L. & Cid, M. B. A General Aminocatalytic Method for the Synthesis of Aldimines. *J. Am. Chem. Soc.* **136**, 1082-1089 (2014). https://doi.org/10.1021/ja4111418
61 Lee, E. C., Hodous, B. L., Bergin, E., Shih, C. & Fu, G. C. Catalytic Asymmetric Staudinger Reactions to Form β-Lactams: An Unanticipated Dependence of Diastereoselectivity on the Choice of the Nitrogen Substituent. *J. Am. Chem. Soc.* **127**, 11586-11587 (2005). https://doi.org/10.1021/ja052058p
